# Weighting strategy and selection analysis in the panel ‘Health in Germany‘: methods and results for the 2024 annual survey

**DOI:** 10.1186/s12874-025-02740-w

**Published:** 2025-12-15

**Authors:** Stefan Damerow, Ronny Kuhnert, Angelika Schaffrath Rosario, Johannes Lemcke

**Affiliations:** Department 2 Epidemiology and Health Monitoring, Robert Koch Institute, Gerichtstraße 27, 13347 Berlin, Germany

**Keywords:** Panel study, Panel recruitment, Nonresponse bias, Selection effects, Drop-out bias, Calibration weighting, Raking, Inverse probability weighting, Design weighting, Representative, Population-based

## Abstract

**Background:**

The panel `Health in Germany` has been established to gather nationwide health-related information, replacing cross-sectional surveys as primary data sources. However, panel designs involve multiple selection stages, potentially introducing additional nonresponse bias. This study aims to describe this drop-out bias and the weighting strategy used to improve representativeness.

**Methods:**

Panelists were recruited through a recruitment study. At completion of the recruitment questionnaire, participants were invited to register for the panel. Registered panelists were subsequently invited to the first annual survey in 2024, which was divided into three sub-waves. Each included one of four questionnaires with different topics. Logistic regression models are used to estimate the probability for panel registration and participation in panel 2024 questionnaires, using sociodemographic and health-related variables from the recruitment study to illustrate drop-out bias. The weighting scheme and techniques are described. To assess the potential for drop-out bias and how it is reduced through weighting, unweighted and weighted estimates are compared to internal and external reference distributions using standardized differences.

**Results:**

Drop-out analysis showed that sociodemographic characteristics (age, education, German citizenship) had a stronger association with panel registration than health-related parameters (e.g., self-rated health, smoking status, sport activity, chronic diseases) among the participants of the recruitment study. Similar patterns as for registration were found for participation in the 2024 questionnaires, except for age, which showed a reverse effect. No differences were observed across questionnaire types. Standardized differences confirmed the findings: sociodemographic characteristics—particularly education and German citizenship—showed larger deviations than health-related parameters. The largest deviations occurred in the recruitment study. The weighting procedure reduced most standardized differences to below 0.5% points. An exception is German citizenship, which showed only slight improvement.

**Conclusions:**

Drop-out within the first year of a newly established panel is mainly affected by sociodemographic variables, with minor effects due to health-related parameters. The additional recruitment steps did not lead to concerning deviations in sample composition compared to the recruitment study. Remaining differences were addressed through drop-out and calibration weighting, so the weighted panel 2024 sample does not substantially differ from what would be expected in a cross-sectional design such as the recruitment study. However, continued analyses are needed, as sample composition may change due to future panel attrition.

**Supplementary Information:**

The online version contains supplementary material available at 10.1186/s12874-025-02740-w.

## Contributions to the literature

This study advances the literature on nonresponse and drop-out bias as well as weighting methodologies in population-representative health panel surveys by examining patterns of selective participation across multiple recruitment steps. While the analysis does not allow for a comprehensive assessment of nonresponse bias, it provides empirical indications of when and how selective participation may arise. These insights contribute to a more nuanced understanding of potential sources of nonresponse-related distortions in designs with multiple recruitment steps.

Furthermore, the study documents a weighting strategy specifically tailored to the requirements and structural characteristics of the panel `Health in Germany`. Although the approach reflects features unique to this panel, the transparent description of the underlying rationale and the evaluation of the results can assist other panel studies facing comparable complexities. In this way, the study offers practical guidance and contributes to broader discussions on panel recruitment, representativeness, and data quality in health panel surveys.

## Introduction

Public health research relies on the availability of reliable and valid data to provide essential information for policymakers. In Germany, the Robert Koch Institute (RKI), Germany’s national public health institute, offers these centralized information services (Ziese et al., 2020). Historically, population-based cross-sectional studies for Germany were used as primary data sources e.g. the Study on the Health of Adults in Germany (DEGS) [1], German Health Update (GEDA) [2], and the Study on the Health of Children and Adolescents (KiGGS) [3]. These surveys were designed to generate prevalence data based on a cross-section of the population every two to six years.

However, it has become evident, especially during the COVID-19 pandemic, that there are pressing demands for continuous and rapid information, and that data collection infrastructure based on cross-sectional studies every two to six years cannot meet these requirements. Therefore, the RKI decided to establish a panel infrastructure (`Health in Germany`) to meet the demands for ad hoc data collection as well as long-term and standardized monitoring of the public health status of Germany’s population [4]. The shift from one-time cross-sectional studies, where participants are invited only once, to a panel design, where participants are involved frequently, needs to be evaluated in order to determine the effects due to changes in the data generation process. In the context of survey research, the Total Survey Error (TSE) framework serves as a comprehensive model for understanding and mitigating the various sources of error that can impact the validity and reliability of survey findings [5].

Drop-out of study participants over time is a well-known problem [6, 7]. A consequence of drop-out is not only reduced sample sizes, leading to lower statistical power, but also potential selective nonresponse (that means non-random nonresponse) which can bias results [8, 9]. In recent decades, a decline - independent of the survey mode - in willingness to participate in population-based studies has been observed [10–15]. For this reason, analyses regarding the nature of nonresponse and drop-out are of increasing importance. If subgroups of the population with specific characteristics are less willing to participate in the panel and are therefore underrepresented in the resulting samples, and if these characteristics are associated with the target variables, systematic differences will arise between respondents and nonrespondents, leading to nonresponse or drop-out bias.

A panel, whether established through random sampling or based on self-recruitment (as in the case of non-probability access panels), inherently contains more potential selection stages than one-time cross-sectional surveys [16]. Compared to a cross-sectional design, the panel ‘Health in Germany’ introduced two additional recruitment steps. First, after completing a brief recruitment questionnaire, participants were asked to register in the panel infrastructure. Second, registered panelists were subsequently invited to take part in regular annual surveys or ad-hoc studies focusing on specific topics and/or subgroups. These additional recruitment steps can introduce further nonresponse or drop-out bias due to selective participation over the course of the panel.

First findings for the initial recruitment study of the panel ‘Health in Germany’ on response and recruitment rates, basic selection analysis, and sample composition regarding sociodemographic characteristics will be published elsewhere soon.

This paper addresses two primary concerns in the context of the TSE components: drop-out bias and nonresponse bias. First, we describe potential drop-out bias within the panel ‘Health in Germany’. This is achieved by estimating logistic regression models that reveal the effects of sociodemographic and health-related indicators on both, the willingness to register in the panel infrastructure and participation in the first regular annual survey in 2024. Secondly, we focus on the application and evaluation of weighting techniques designed to reduce the potential for nonresponse and drop-out bias. Using the data from the 2024 annual survey, we aim to systematically examine how weighting strategies —encompassing design, drop-out (inverse probability) weighting, and calibration weighting —can substantially improve sample representativeness and reduce nonresponse-related error, thereby enhancing the overall quality of the collected data.

## Design and methods

### Sample and study design

The recruitment of panelists was done via a recruitment study from January 2024 to the end of May 2024. After completing the questionnaire of the recruitment study, the participants were asked to register in the panel infrastructure. Details on the sample and study design can be found in [4].

The recruitment study comprises individuals aged 16 and above having their main place of residence in Germany. The sample is based on a two-stage stratified (cluster) sample with 359 primary sampling units (PSUs) randomly selected proportional to population size from a list of all political municipalities in Germany, stratified by federal state and type of municipality (BIK classification) [17, 18]. Due to their large population size, several major cities were represented by multiple PSUs. Within the PSUs, addresses were randomly selected from the residents’ registration offices, stratified by age group. The size of the gross sample for each age group was selected according to anticipated age-specific response rates. In each PSU, the same number of addresses was drawn. To ensure a sufficient number of PSUs for smaller federal states, a minimum of 14 PSUs per state was selected. In the states of Berlin and Schleswig-Holstein, the number of addresses per PSU was increased to enable more detailed analyses. In cases where the number of available addresses within a PSU was too small or where selecting all addresses would have covered a substantial share of the local population, municipalities were merged to form synthetic PSUs to ensure data protection. As a result, a single PSU could include multiple municipalities.

The recruitment study followed a mixed-mode approach with a computer-assisted web interview and a paper questionnaire [19–22]. People up to 69 years received the online access data (link and QR code) with the postal invitation letter and the first reminder, the paper questionnaire was additionally offered only with the second (last) reminder. Due to the lower internet usage of elderly participants, for individuals aged 70 and above, both the online interview and the paper questionnaire were offered starting with the first invitation letter. In the regular panel operation, participants usually continued with the same survey mode chosen for the recruitment study. Participants received unconditional incentives with the invitation letter for the recruitment study. Conditional incentives were used for registration and participation in the panel sub-waves.

In the recruitment study, 62,556 questionnaires were received (AAPOR Response Rate 2: 37.6%). At the end of the questionnaire, a total of 47,863 participants registered for future participation in the panel (Recruitment Rate 28.7%).

Registered panelists are invited annually to the regular panel survey, which is divided into four separate sub-waves (quarterly), each focusing on different health-related topics. The panelists are randomly allocated to four subsamples, each of which receives the four quarterly questionnaires in a different order. For a detailed description of the rotation scheme, see [4].

The 2024 annual survey comprised three quarterly sub-waves instead of the usual four, resulting in a sample size that was approximately a quarter smaller for each questionnaire. The panel sub-waves were conducted during the following periods:

- Wave 1: May 28, 2024, to August 5, 2024 (38,212 participants, 81.3% response rate).
- Wave 2: August 12, 2024, to October 14, 2024 (36,134 participants, 77.1% response rate).
- Wave 3: October 28, 2024, to January 6, 2025 (35,786 participants, 75.3% response rate).

The 2024 annual survey is intended for analyses of participants aged 18 and above. Among the 46,863 registered panelists aged 18 or older, 41,376 took part in at least one of the three sub-waves, corresponding to a response rate of 88%.

### Drop-out analysis

To analyse selection effects related to panel registration, a logistic regression model was estimated for the willingness to register, to describe possible participation behaviour that can cause drop-out bias, if the variables affecting participation are associated with the target variables of the panel. The model was based on the participants of the recruitment study. The same model was used for the analysis of selection effects concerning participation in the regular panel study in 2024, but limited to the subpopulation of registered panellists, which allows for distinguishing between registration (yes/no) and participation in questionnaires A to D (yes/no). The model was run separately for each questionnaire because the final analyses are primarily conducted within each questionnaire, making potential biases within the various questionnaires of specific interest. A key consideration in selecting the independent variables was to include sociodemographic information as well as health-related indicators. The recruitment study’s questionnaire was specifically designed to cover a broad spectrum of health-related factors, and thus the following information was utilized:

- Age group: Age calculated from birth year and month to participation in the recruitment study and categorized into age groups (18–29 years, 30–39 years, 40–49 years, 50–59 years, 60–69 years, 70 + years).
- Sex: Officially recorded sex at birth (male, female).
- Education: Categorized as low, medium, high according to CASMIN classification [23].
- Household size: Single- versus multi-person household.
- Type of municipality (BIK classification, 2023): Municipalities in Germany are classified based on their size and commuter flows [18]. The official ten categories are summarized as follows:
  - BIK1: Municipality belonging to a BIK region < 20,000 inhabitants.
  - BIK2: Municipality belonging to a BIK region with 20,000 to < 50,000 inhabitants or a region including surroundings of a city with 50,000 to < 500,000 inhabitants.
  - BIK3: Municipality belonging to a BIK region including a core city with 50,000 to < 500,000 inhabitants or surroundings of a city with 500,000 + inhabitants.
  - BIK4: Municipality belonging to a BIK region including a core city with 500,000 + inhabitants.
- Region: Based on the federal state of residency, regions are categorized as follows:
  - Northeast: Berlin, Brandenburg, Mecklenburg-Western Pomerania.
  - Northwest: Schleswig-Holstein, Hamburg, Lower Saxony, Bremen.
  - Central-East: Saxony, Saxony-Anhalt, Thuringia.
  - Central-West: North Rhine-Westphalia, Hesse, Rhineland-Palatinate, Saarland.
  - South: Baden-Württemberg, Bavaria.
- German: Self-reported information on holding German citizenship (Yes/No).
- BMI: Body mass index (BMI) was calculated from self-reported weight (kg) and height (m) as BMI = weight in kg/(height in m)² and categorized as follows:
  - Underweight (BMI < 18.5).
  - Normal weight (18.5 < = BMI < 25).
  - Overweight (25 < = BMI < 30).
  - Obesity (BMI >= 30).
- Chronic disease: Self-reported chronic disease (Yes/No).
- Self-rated health: Categories from “very good” to “very bad”, grouped into very good/good/fair vs. bad/very bad.
- Self-rated mental health: Categories from “excellent” to “poor”, grouped into excellent/very good/good vs. fair/poor.
- Paying attention to health: Categories from “very strong” to “not at all”, grouped into not at all/less strong/moderate vs. strong/very strong.
- Satisfaction with life in general: Scale from 1 (completely dissatisfied) to 10 (completely satisfied) categorized as 1 to 3, 4 to 7, and 8 to 10.
- Consumption of red meat: Self-reported information in categories: Daily or several times a day, 4 to 6 times per week, 1 to 3 times per week, less than once per week, never.
- Consumption of sausage products: Self-reported information in categories: Daily or several times a day, 4 to 6 times per week, 1 to 3 times per week, less than once per week, never.
- Current smoking: Self-reported information on daily smoking, occasional smoking or non-smoking (combining never-smokers and ex-smokers).
- Sport: Weekly duration of sporting activity during the past three months, in the following categories: No sporting activity, less than 1 h per week, regularly 1 to less than 2 h per week, regularly 2 to less than 4 h per week, and regularly 4 or more hours per week.
- Subjective health risk due to climate change: Scale from 1 (not at all) to 10 (very much) categorized as 1 to 3, 4 to 7, and 8 to 10.
- Waited for appointment: Waited for medical appointment in the last 12 months (Yes/No).

The models were estimated in R (version 4.3.0 [24]), taking into account the survey design and the weighting factors calculated for the recruitment study, using svyglm from the survey package [25]. Odds ratios were calculated and graphically compared and evaluated using ggcoef_compare from the ggstats package [26]. A statistically significant effect is assumed if the calculated p-value is < 0.05. The analyses were based on data version 3 of the recruitment study and version 5 of 2024 annual survey.

### Weighting

#### Weighting scheme

**Fig. 1:**
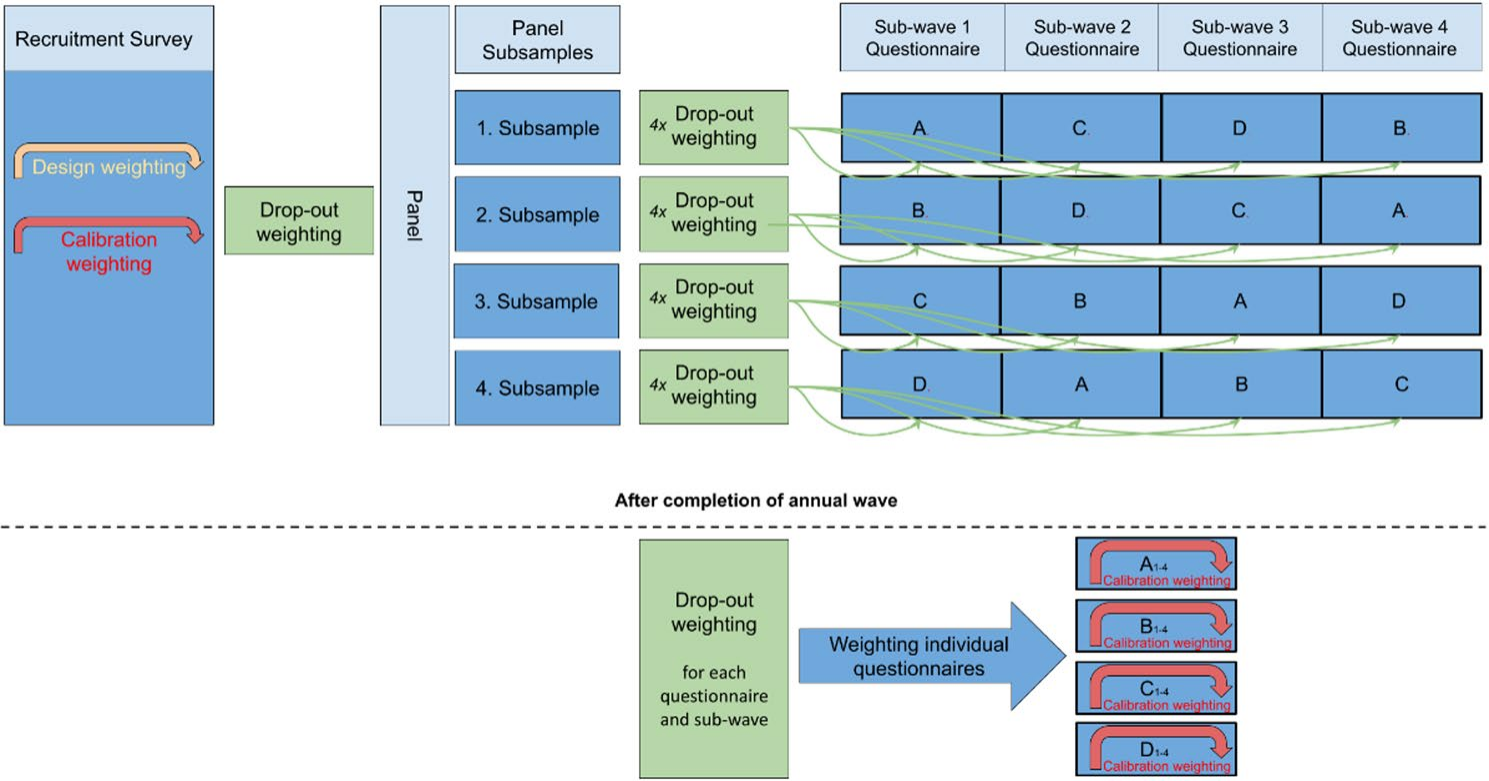
Weighting scheme for a regular annual panel survey with four sub-waves and questionnaire A-D – from recruitment study to panel registration to sub-wave participation

Figure 1 provides a schematic representation of the weighting process for a regular annual survey with four sub-waves. The first step is the weighting of the recruitment study, i.e. design weighting (taking account of the two-stage sampling as described below) and calibration weighting (adjusting the sample to known population totals from official statistics). In the subsequent weighting steps, which build upon the initial weighting factor, drop-out weights are calculated for both the registration process and subsequent participation in each survey sub-wave. As participation behaviour may vary between the questionnaires and sub-waves, the drop-out weighting is performed independently for each questionnaire and sub-wave. The final step, which produces the cross-sectional weights for each questionnaire, is again a calibration weighting based on official statistics.

The construction of the annual sample weights $\:weigh{t}^{Q}$ for questionnaires (*Q*) A to D is therefore given by, where 

$$\begin{aligned}\:weigh{t}^{Q}&=wDesign\times AFCalibratio{n}^{RS}\times AFDro{p}^{Reg}\\ &\quad \times\:AFDro{p}^{Q,t}\times AFCalibratio{n}^{Q}\end{aligned}$$

where $\:wDesign$ denotes the sampling design weight, $\:AFCalibratio{n}^{RS}$ the adjustment factor based on calibration weighting of the recruitment study, $\:AFDro{p}^{Reg}$ the adjustment factor accounting for drop-out during registration and $\:AFDro{p}^{Q,t}$ the adjustment factor accounting for drop-out in sub-wave *t* and questionnaire Q and $\:AFCalibratio{n}^{Q}\:\:$the adjustment factor based on calibration weighting for questionnaires A to D.

The following sections describe each weighting step in detail.

#### Design weighting

Due to the stratified two-stage sampling procedure, selection probabilities varied among participants. This imbalance is corrected by calculating a design weight [27].The design weight *wDesign* is determined as the inverse of the selection probability of each PSU ($\:{p}_{PSU}$), stratified by federal state, multiplied by the selection probability of the participant within each PSU ($\:{p}_{i,ag{e}^{j},PSU}$).

The selection probability of each PSU within a federal state is given by

$$\:{p}_{PSU}=\frac{{N}_{PSU}}{{N}_{FS}}$$

, with $\:{N}_{PSU}$ representing the population aged 16 and above in each PSU, and $\:{N}_{FS}$ representing the population size of the corresponding federal state, both at the time of sampling as of December 31, 2021.

The selection probability for persons within each sampled PSU is given by

$$\:{p}_{i,ag{e}^{j},PSU}=\frac{{n}_{resp,ag{e}^{j},PSU}}{{N}_{ag{e}^{j},PSU}}$$

, where $\:{n}_{resp,ag{e}^{j},\mathrm{P}\mathrm{S}\mathrm{U}}$ defines the number of participants in age group *j* in the sampled PSU and $\:{N}_{ag{e}^{j},PSU}$ the respective number of inhabitants in the sampled PSU.

To account for the number of sampled PSUs within each federal state, the design weight $\:wDesig{n}_{i}\:$is further adjusted by dividing by the number of sampled PSUs in each federal state ($\:\#PS{U}_{FS}$):

$$\:{wDesign}_{i,ag{e}^{j},PSU\:}=\frac{1}{{p}_{PSU}\times\:{p}_{i,ag{e}^{j},PSU}}\times\frac{1}{\#PS{U}_{FS}}$$

#### Drop-out weighting

Participant recruitment for regular annual survey involves several steps: initial participation in the recruitment study, followed by registration to participate in the panel, and ultimately, participation in each quarterly sub-wave. Unlike cross-sectional studies, the latter two steps are additional sources of bias (drop-out bias). However, the information on demographic variables, health status and health behaviour collected in the recruitment study allows for reducing these drop-out biases. This adjustment is achieved through drop-out weighting, employing the technique of inverse probability weighting (IPW) [8, 28]. In this approach, the participation probability ($\:p$) is estimated using logistic regression with registration/participation (yes/no) as the independent variable and the variables collected in the recruitment study as explanatory variables. In principle, participation probabilities could also be estimated using the gross sample. However, the information available in the gross sample is limited to age, sex, and place of residence, all of which are already incorporated in the design and calibration weighting. Consequently, estimating an additional model based on the gross sample would not provide any new information. Therefore, we did not pursue a separate estimation of participation probabilities for the gross sample.

Following the IPW method, the weighting factor ($\:wDro{p}_{i}$) for each (re-)participant ($\:i$) is calculated as the inverse of the registration/participation probability, multiplied by the weighting factor derived from the previous weighting step ($\:weight$):

$$\:wDro{p}_{i}=\frac{1}{{p}_{i}}\times\:weigh{t}_{i}$$

.

This calculation assumes that participation decisions are independent between respondents and that each individual appears only once in the panel. Variable selection for estimating the registration/participation probability is of central importance and was conducted using lasso regression [29], as for example [30] showed better performance than a-priori knowledge-based variable selection. All variables from the recruitment study were included (see variable list in additional file 1), along with interaction terms for age group (16–29, 30–39, … 70–79, 80 + years), sex, and education (CASMIN [23]: low, medium, high). The variable selection was performed using cv.glmnet from the glmnet package [31] in R (version 4.3.0 [24]), with 10-fold cross-validation. The final model was selected based on the regularization parameter that minimizes the cross-validation error. The variables sex, age group, and education were always included, as they represent key sociodemographic dimensions and are also part of interaction terms.

Lasso regression cannot directly handle weighting factors and the complex two-stage survey design. However, to accurately predict registration/participation probabilities, it is essential to incorporate the weights from previous steps. Therefore, the R survey package [25] was used to estimate the registration/participation probabilities, using the variables selected by the lasso regression, while appropriately accounting for the survey design and the corresponding weighting factors.

For the variable selection, missing values were replaced. Additional file 1 also presents the proportions of missing values for the variables included in the selection process, differentiated by registration and participation status for questionnaires A to D. The overall proportion of missing values was very low, with a maximum of 0.7%. Moreover, the proportions did not differ between registered and non-registered individuals, nor between participants and non-participants in any of the questionnaires A to D. Consequently, replacing missing values had only a negligible impact on the variable distributions. Therefore, complex imputation methods were deemed unnecessary. Missing values in explanatory variables were automatically replaced: continuous variables were replaced with their respective median, while categorical variables were replaced with the mode.

The results of the logistic model estimation for registration in the panel, based on the participants of the recruitment study, are available in additional file 1. This model was used to predict the probability of registration for each registered panelist ($\:{p}_{i}^{Reg}$). Following the IPW method, the weighting factor after accounting for the drop-out in registration ($\:wDro{p}_{i}^{Reg}$) was calculated by multiplying the weight from the recruitment study ($\:wRecrui{t}_{i})$ by the inverse of the predicted registration probability $\:{p}_{i}^{Reg}$:

$$\:wDro{p}_{i}^{Reg}=AFDro{p}_{i}^{Reg}\:\times\:wRecrui{t}_{i}=\:\frac{1}{{p}_{i}^{Reg}}\times\:wRecrui{t}_{i}$$

, where $\:wRecrui{t}_{i}\:$denotes the product of the design weight and the adjustment factor based on the calibration of the recruitment study.

The results of the 12 logistic regressions for participation in the 2024 annual survey, for questionnaire A to D in each sub-wave, with survey 2024 only covering the first to third sub-wave, are also available in additional file 1. The model is estimated based on the sample of registered panelists. The drop-out weight $\:wDro{p}_{i}^{Q,t}$ for participants (*i*) in the specific questionnaire (*Q*) and survey quarter (*t*) were calculated by multiplying the inverse of the predicted participation probability $\:{p}_{i}^{Q,t}$ by the drop-out weight from the registration stage ($\:wDro{p}_{i}^{Reg})$:

$$\:wDro{p}_{i}^{Q,t}=AFDro{p}_{i}^{Q,t}\times\:wDro{p}_{i}^{Reg}=\frac{1}{{p}_{i}^{Q,t}}\times\:wDro{p}_{i}^{Reg}$$

To prevent extreme values for the drop-out weights for registration as well as panel sub-wave and questionnaire participation, the weighting factors were trimmed to the 0.5th percentile (lower bound) and the 99.5th percentile (upper bound) of their distribution within each federal state.

#### Calibration weighting

In general, the willingness to participate is not uniform across population groups, but varies, for example, by region, age, gender, or educational level. In what is known in general form as calibration weighting, this varying willingness to participate is aligned by adjusting the net sample of participants to the population distribution of selected official statistics [27]. In contrast, the drop-out weighting described above uses information collected from the recruitment study itself to adjust for potential selective registration and re-participation, rather than relying on external data sources. Drop-out weighting addresses drop-out bias that may arise within the panel, regarding registration for the panel and participation in the 2024 sub-waves. The main purpose of calibration weighting, on the other hand, is to reduce nonresponse and coverage bias that may result from systematic differences between the sample and population distributions. By calibrating, the weighted sample reflects the population composition more accurately. In addition, calibration weighting may reduce random sampling bias, thus improving the precision of estimates if the calibration variables are correlated with the target variables.

A widely used iterative method for calibration weighting is raking [32, 33]. The raking algorithm calculates an adjustment factor ($\:AFCalibratio{n}^{RS}$and $\:AFCalibratio{n}^{Q}$) to the respondent weights in an iterative process, to make the sample totals within each raking dimension align with known population totals. Within each raking dimension, an adjustment to the weights is calculated as the ratio between the total of each weighting cell in the external reference distribution and the corresponding total in the sample, with the sample total weighted by the previously computed weights. The process iterates through five raking dimensions (for a detailed description, see below). Since adjusting the distribution in one dimension can distort the distributions in other dimensions, the process is repeated iteratively until the sum of differences between the weighting factors of successive iterations is minimized and falls below a predefined threshold (i.e., convergence is reached). Whereas the classical raking procedure [34] calibrates solely to univariate distributions, the approach applied here uses cross-classified parameters as calibration targets. This modification accounts for correlations and interaction effects among the included parameters (see also the modified raking ratio estimation by Oh and Scheuren [35]). Table 1 details the five raking dimensions applied in the calibration weighting for both the recruitment study and the four questionnaires in the 2024 annual survey. According to Särndal and Lundström [36], the selection of calibration variables should consider variables that are correlated with the outcome measures, describe response behavior, and represent important subgroups. In Germany, there is no comprehensive reference data specifically related to health that could be used for health surveys. Consequently, variable selection is largely limited to sociodemographic characteristics available from official statistics. In health studies, outcomes are often strongly associated with age, sex, education, and living situation (e.g., household size, urbanity), and participation behavior also depends on these variables. Moreover, health outcomes frequently vary across subgroups defined by, for example, education in combination with age and sex. Therefore, the raking dimensions include detailed cross-classifications to capture these patterns. Since the data is also intended for analyses at the federal state level, federal states or regions are incorporated in each dimension instead of performing the raking for each federal state separately as proposed in Battaglia et al. [37]. To ensure applicability of the weights in different types of analyses, the age cut-points were varied across these dimensions to capture diverse age categorizations. Due to the time gap between sampling and recruitment, almost all invited individuals had already passed the age of 16. Therefore, the calibration weighting in the recruitment study starts at a minimum age of 17. For the panel 2024 annual survey, the analysis population was defined as individuals aged 18 and older, therefore the calibration weighting was based on this age threshold. Population totals from the Microcensus 2021 [38] were applied for raking dimensions 2 and 4, while population figures as of 31/12/2023 served as the margins for the other dimensions [39]. To prevent inconsistencies between the different sets of control data, the Microcensus data was normalized to the population figures prior to raking. The raking dimensions used in the recruitment study and the 2024 annual survey were largely identical. The only difference is that, in dimension two, federal states are grouped into broader regions in 2024 annual survey due to a lower number of participants. Notably, Berlin and Schleswig-Holstein are treated as separate federal states within this dimension to account for their oversampling.

**Table 1:** Calibration weighting for the recruitment study and for each questionnaire of the 2024 annual survey: raking dimensions

Raking Dimension	Parameter
1	Federal State X Sex X Age Group 1
2	Federal State/Region^a^ X Sex X Age Group 2 X Education
3	Federal State X Age Group 3 X BIK Classification
4	Federal State X Age Group 4 X Household Size
5	Federal State X Sex X Age Group 5

Definitions	
Federal State:	Schleswig-Holstein, Hamburg, Lower Saxony, Bremen, North Rhine-Westphalia, Hesse, Rhineland-Palatinate, Baden-Württemberg, Bavaria, Saarland, Berlin, Brandenburg, Mecklenburg-Western Pomerania, Saxony, Saxony-Anhalt, Thuringia
Sex:	Officially recorded sex at birth (Male, Female)
Household Size:	Single-person household, Multi-person household
Education^b^:	CASMIN classification (Low, Medium, High)
Region:	North/Central-East, Northwest, Central-West, South, Berlin, Schleswig-Holstein
BIK Classification^c^:	BIK region < 20,000 inhabitants; BIK region 20,000 to < 50,000 inhabitants or BIK Region including surrounding area of city 50,000 to < 500,000 inhabitants; BIK region including core city 50,000 to < 500,000 inhabitants or surrounding area 500,000 + inhabitants; BIK region including core city 500,000 + inhabitants
Age Group 1^d^:	18–19 yrs, 20–24 yrs, 25–29 yrs, 30–39 yrs, 40–49 yrs, …, 80 + yrs
Age Group 2^d^:	18–19 yrs, 20–24 yrs, 25–34 yrs, 35–45 yrs, …, 65–79 yrs, 80 + yrs
Age Group 3^d^:	18–29 yrs, 30–44 yrs, 45–64 yrs, 65 + yrs
Age Group 4^d^:	18–24 yrs, 25–34 yrs, 35–49 yrs, 50–64 yrs, 65–79 yrs, 80 + yrs
Age Group 5^d^.	18–24 yrs, 25–34 yrs, 35–44 yrs, …, 85 + yrs

^a^ Recruitment study: federal state, 2024 annual survey: region

^b^ see Brauns et al. [23]; CASMIN set to “low” for ages below 20

^c^ see Behrens et al. [18]

^d^ In the recruitment study, weights are also calculated for participants under the age of 18. Accordingly, age groups start at 17

Raking dimension 2 and 4: Population totals Microcensus 2021 (35)

Raking dimension 1, 3 and 5: Population figures as of 31/12/2023 (34)

Raking requires that there are no missing values in the variables used, as otherwise, no weighting factor can be calculated for the affected observations. Missing values occurred in the variables education (*n* = 179 (0.3%)) and household size (*n* = 161 (0.3%)). Based on the distribution in the Microcensus 2021 [38], the missing values were substituted by random-draw single imputation within each federal state–age–sex group, thus preserving the observed marginal distributions.

To prevent extreme weighting values, after each raking iteration, the weighting factors were trimmed to the 0.5th percentile (lower bound) and the 99.5th percentile (upper bound) of their distribution within each federal state. Trimming distorts the adjusted weighted distribution, which in turn increases the number of iterations needed for convergence and results in slight discrepancies between the sample and the reference distributions. Furthermore, the calibration weights are normalized with respect to the total number of observations in the sample. For the calibration weights applied to each questionnaire in 2024 annual survey, the sample size was evenly allocated across the sub-waves in order to mitigate potential seasonal effects.

Raking was implemented using a self-written R function according to Lumley [40]. In each iteration, the procedure sequentially cycles through all raking dimensions (see Table 1) and adjusts the weighted sample totals within each group of the respective cross-classification. This adjustment is achieved by multiplying the individual weights by a factor defined as the ratio of the weighted sample total to the population total within the corresponding group of the current dimension. After completing one full iteration across all five dimensions, the resulting weights are trimmed as described above. The sum of absolute differences between the pre-iteration weights and the trimmed post-iteration weights is then computed. If this difference exceeds one, a new iteration is initiated using the adjusted weights from the previous iteration.

Due to a technical error in the sampling process, certain age groups in Berlin have very few observations, and no participants aged 85 or older are present for this state. As a consequence, the upper limit for raking dimension 5 in Berlin was set at 75 years.

To ensure the sample can be adjusted to the marginal distribution via raking, each cell in all raking dimensions must have at least one observation. Also, to avoid problems such as extreme weighting factors, cell counts should not be too small. Weighting cells were therefore merged (across age group or sex) if they were empty or had very few observations (*n* < 4). For the 2024 annual survey, the following cell merging were applied across all questionnaires: In raking dimension 1, sex was combined for Berlin within the 55–64 age range and for Brandenburg and Mecklenburg-Western Pomerania for participants aged 18 to 19. In raking dimension 2, sex was combined for individuals aged 20–34, with a specific aggregation for Berlin in the 55–64 age group. Within Rhineland-Palatinate at dimension 3, the 18–29 and 30–44 age groups were merged for the BIK classification “BIK region including core city with 500,000 + inhabitants”. No cell merging was required for the raking procedure in the recruitment study.

For individuals below the age of 20, classification by educational attainment according to CASMIN is not appropriate, as the majority of individuals in this age group have not yet completed their formal education. In this context, a more suitable indicator would be the current type of school attended in combination with the intended educational qualification. As this information is not available, differentiation by education is not applied for this age group. Accordingly, the CASMIN categories were collapsed into the lowest category in both the sample and the marginal distribution for individuals younger than 20 years.

## Evaluation of the sample composition

To assess the results and effects of the weighting process, reference distributions from official statistics [38, 39] are compared to the unweighted and weighted proportions in the samples of participants from the recruitment study, registered panelists, and the first annual survey. This external comparison is limited to variables for which marginal distributions are available. However, the recruitment study itself can serve as an internal reference in the drop-out analysis, since its cross-sectional design provides the best available benchmark at the time of the study for assessing possible drop-out bias. Comparing the distribution of health-related variables in the samples of registered panelists and participants of the first annual survey to the recruitment study can be used to understand at which point in the panel process selective drop-out occurs. To link the results to the drop-out analysis, all variables included in the drop-out analysis are incorporated into the evaluation table. In addition, federal state is included, as the calibration weighting is constructed using this stratification.

For the comparison, standardized differences ($\:\varDelta\:$) between proportions in the external reference and internal comparison samples are calculated for all weighting steps as follows:

$$\:\varDelta\:\:\:=\:\frac{{p}_{1}-{p}_{2}}{\sqrt{\left({p}_{1}\left(1-{p}_{1}\right)+{p}_{2}\left(1-{p}_{2}\right)\right)\times\:2}}\times\:100$$

, with $\:{p}_{1}$ as reference proportion and $\:{p}_{2}$ as the proportion to be compared. The calculation is based on Austin and Stuart [41]. However, for ease of interpretation we divide their standardized difference by 2, so that our standardized difference is approximately equal to the absolute difference for prevalences around 50%. For other prevalences, the standardized difference is higher than the absolute difference, e.g. a difference of 3% around a prevalence of 10% corresponds to a standardized difference of 5%. Austin and Stuart [41] suggest that a standardized difference of 10% is generally considered negligible, corresponding to a standardized difference below 5% with our modified definition. Furthermore, descriptive statistics (minimum, maximum, median, coefficient of variation) of the weighting factors are presented. Additionally, the effectiveness of the weights is determined. This allows for the assessment and comparison of the dispersion of the weights. The weighting effectiveness ($\:Eff$) is calculated by:

$$\:Eff=\frac{{\left(\sum\:_{i=1}^{n}{w}_{i}\right)}^{2}}{\sum\:_{i=1}^{n}{w}_{i}^{2}}\times\:\frac{100}{n}$$

, where the weight of subject $\:i$ is given by $\:{w}_{i}$, and $\:n$ represents the sample size.

## Results

### Drop-out analysis

**Fig. 2:**
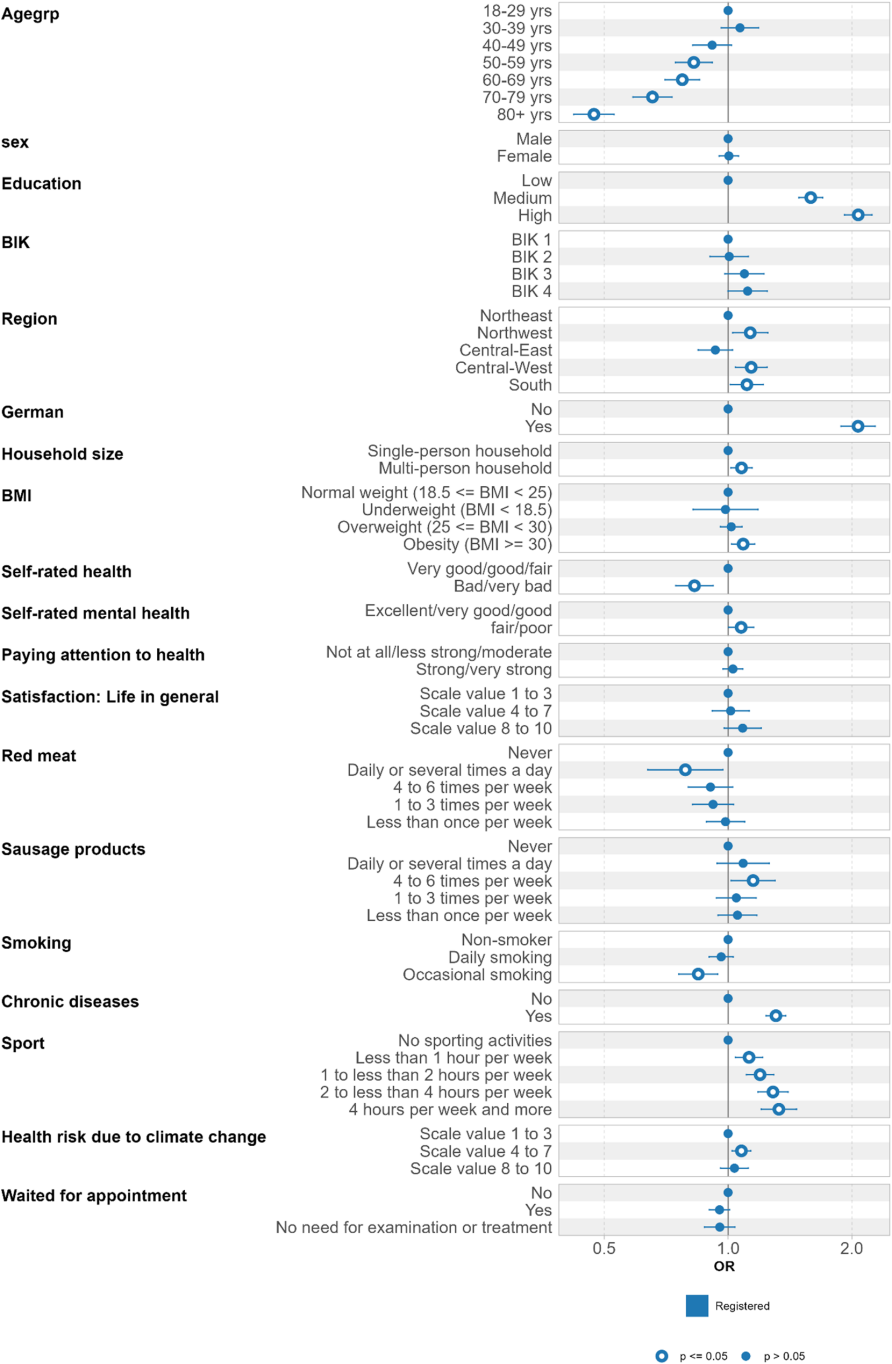
Panel registration (yes/no): Odds ratios (OR) and 95% confidence intervals from logistic regression (*n* = 59,580)

Figure 2 illustrates the results of the logistic regression for panel registration. Logistic regression shows that the willingness to register decreases with increasing age. Regarding region and household size, individuals from western and southern federal states and from multi-person households are more likely to register for the panel. German citizenship and education have a notable impact on the willingness for registration, while sex and BIK-classifications have none. Concerning health-related aspects, individuals with bad/very bad self-rated health register less frequently. Conversely, individuals with chronic disease, fair/poor self-rated mental health and more sport activity register more often. A slight tendency is observed for individuals with higher red meat consumption and (occasional) smoking behaviour to register less frequently. There are none or only minor effects on registration behaviour regarding BMI categories, overall life satisfaction, consumption of sausage products, concerns on health risk due to climate change and waiting for a medical appointment.

**Fig. 3:**
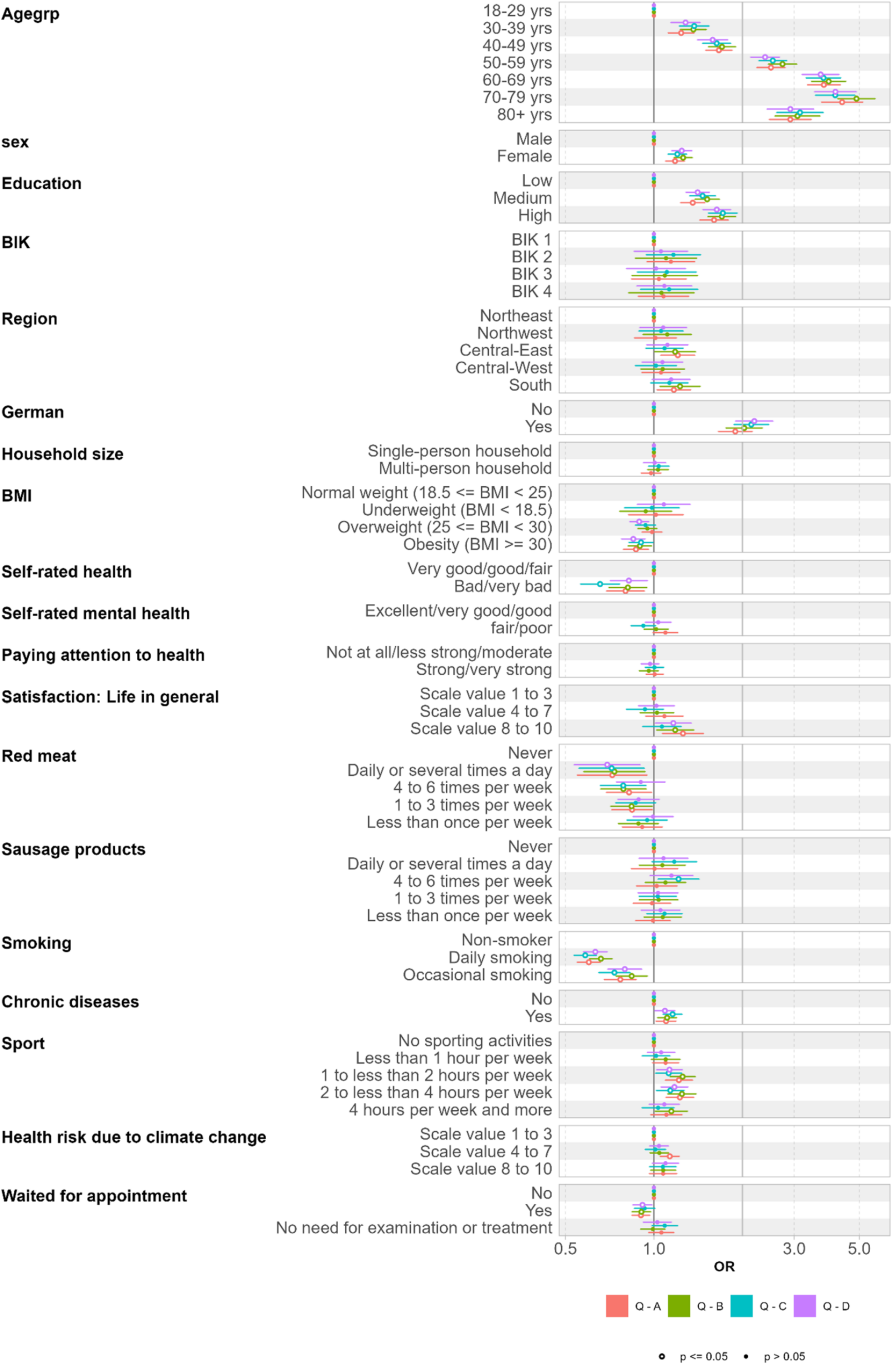
Participation in questionnaire (Q) A to D (yes/no): Odds ratios (OR) and 95% confidence intervals from logistic regression (Q-A: *n* = 34,176; Q-B: *n* = 34,213; Q-C: *n* = 34,062; Q-D: *n* = 34,196)

Figure 3 illustrates the results of the logistic regression for participation in the 2024 annual survey, stratified by questionnaires A to D. The logistic regression indicates that participation willingness increases with age. Female panelists, those with German citizenship and medium or high education level are more likely to participate. For BIK classification, regions, and household size, no or only minor effects were observed. Regarding health-related aspects, the participation pattern corresponds in several aspects to the observations made for the registration: individuals with more sport activities are more likely to participate, whereas those reporting bad/very bad self-rated health participate less frequently. Again, a slight tendency can be observed indicating that individuals with higher red meat consumption are less likely to participate. For smoking, a clearer effect is evident: daily and occasional smokers participate less frequently. In contrast to the registration process, no participation effect was observed for self-rated mental health. Similar to registration, there are none or only minor effects on participation behaviour for BMI categories, overall life satisfaction, consumption of sausage products, concerns on health risk due to climate change and waiting for a medical appointment. For all analyzed parameters, no differences were observed between the questionnaires A to D.

### Evaluation of the sample composition

In Table 2, the distributions of selected variables in the recruitment study, the panel sample (registered panelists) and participants in questionnaire A of the 2024 annual survey are compared to external and internal reference distributions using standardized differences. The standardized differences are calculated unweighed as well as using the weighting factors up until the respective weighting step. Questionnaires B to D, as well as the results stratified by sex, are omitted from the table for presentation purposes. The findings from these questionnaires are consistent with those of questionnaire A. Similarly, stratification by sex does not reveal substantial differences from the main effects. Both sets of results can be accessed in additional file 2.

In the sample of the recruitment study, the major differences from the external references occur in education, German citizenship and federal states. The design weighting effectively adjusts the sample’s distribution across federal states and provides a partial correction for age. As intended, the calibration weighting brings the distributions of the sociodemographic parameters included in raking into congruence with the reference data.

For the population of registered panelists, it is apparent that the design and calibration weighting – calculated for the entire recruitment study population rather than solely for registered panelists – already resolves most of the initial deviations from the reference values. Notably, an improvement is observed even for variables not included in the calibration weighting. Subsequently, the drop-out weighting for registration reduces the standardized deviations to predominantly below 0.5% points for almost all variables.

Almost the same can be observed for participants of the 2024 annual survey completing questionnaire A: The application of the recruitment study’s calibration weight (CW) or the weighting factor correcting for drop-out in the registration process corrected most of the initial deviations. The subsequent drop-out and calibration weighting steps further ensured that deviations from the external and internal reference data were predominantly resolved.

German citizenship, which is not included in the calibration weighting, is the only exception of the analyzed parameters. The deviation from the external reference value decreases only slightly after each weighting step and remains substantially large for all samples (recruitment study, registration, participation questionnaire A).

**Table 2:** Standardized differences to external and internal references (Δ^a,^ %-points) by weighting factor and sample: recruitment, registration, questionnaire A

		Recruitment Study			Registration				Participation Questionnaire A					
Parameter	External/internal reference^b^	Unweighted	Design-weighted	Calibration-weighted (Recruitment Study)	Unweighted	Design-weighted	Calibration-weighted (Recruitment Study)	Drop-out-weighted (Registration)	Unweighted	Design-weighted	Calibration-weighted (Recruitment Study)	Drop-out-weighted (Registration)	Drop-out-weighted (Questionnaire A)	Calibration-weighted (Questionnaire A)
	[%]	[Δ]	[Δ]	[Δ]	[Δ]	[Δ]	[Δ]	[Δ]	[Δ]	[Δ]	[Δ]	[Δ]	[Δ]	[Δ]
Sex														
Male	48.9	−1.9	−1.9	0.0	−2.2	−2.1	0.0	0.0	−3.5	−3.2	−1.3	−1.4	−0.1	0.0
Female	51.1	1.9	1.9	0.0	2.2	2.1	0.0	0.0	3.5	3.2	1.3	1.4	0.1	0.0
Agegrp														
18–29 yrs	16.0	0.7	−0.2	−0.1	1.7	0.7	0.9	−0.3	−2.4	−2.9	−3.2	−4.4	−0.8	0.2
30–39 yrs	15.8	−0.8	−0.1	0.1	0.3	1.1	1.4	0.1	−2.1	−1.4	−1.3	−2.6	−0.1	0.1
40–49 yrs	14.7	−3.8	−0.2	0.1	−3.3	0.4	0.7	0.1	−4.1	−0.3	−0.1	−0.9	0.1	−0.2
50–59 yrs	17.6	0.3	0.6	0.3	0.8	0.9	0.7	0.4	2.0	2.3	2.2	1.8	0.7	0.4
60–69 yrs	16.5	−0.1	−0.2	−0.1	−0.1	−0.2	−0.2	0.0	2.3	2.2	2.7	2.8	0.1	−0.2
70–79 yrs	10.7	3.4	1.6	−0.3	2.3	0.4	−1.4	−0.1	5.1	3.0	1.2	2.5	0.0	−0.1
80 + yrs	8.7	0.5	−1.8	−0.1	−2.3	−4.6	−3.2	−0.1	−1.4	−3.8	−2.3	0.8	−0.1	−0.4
Federal State ^c^														
Schleswig-Holstein	3.5	12.5	0.0	0.0	12.6	0.1	0.2	0.1	12.9	0.2	0.2	0.2	0.5	0.0
Hamburg	2.2	3.9	−0.1	0.0	4.2	0.3	0.4	0.2	4.0	0.1	0.1	−0.1	0.2	0.0
Lower Saxony	9.6	−3.8	0.0	0.0	−3.9	0.0	0.0	−0.1	−4.0	−0.2	−0.4	−0.5	−0.4	0.0
Bremen	0.8	8.4	0.0	0.0	8.6	0.1	0.1	0.1	8.5	0.0	−0.1	−0.1	0.2	0.0
North Rhine-Westphalia	21.4	−7.2	0.0	0.0	−7.2	0.2	0.1	−0.2	−7.2	0.2	0.0	−0.3	−0.3	0.0
Hesse	7.6	−4.4	0.0	0.0	−4.5	−0.2	−0.2	−0.2	−5.6	−1.6	−1.6	−1.7	−1.4	0.0
Rhineland-Palatinate	4.9	−3.5	−0.1	0.0	−3.2	0.5	0.5	0.5	−2.9	0.9	0.9	1.0	0.9	0.0
Baden-Württemberg	13.3	−5.9	−0.1	0.0	−5.6	0.1	0.1	−0.1	−5.8	−0.1	−0.2	−0.3	−0.8	0.0
Bavaria	15.9	−5.7	0.0	0.0	−5.7	−0.1	−0.2	0.0	−5.1	0.8	0.7	0.7	0.4	0.0
Saarland	1.2	7.0	0.0	0.0	6.9	0.0	0.0	0.1	7.1	0.2	0.2	0.3	0.3	0.0
Berlin	4.5	10.5	0.0	0.0	11.0	0.3	0.4	0.3	10.5	−0.1	0.3	0.1	0.9	0.0
Brandenburg	3.1	0.8	0.0	−0.1	0.6	−0.2	−0.2	−0.1	0.4	−0.4	−0.3	−0.2	0.0	0.0
Mecklenburg-Western Pomerania	2.0	3.9	0.0	0.0	3.5	−0.3	−0.3	−0.2	3.4	−0.3	−0.3	−0.2	0.0	0.0
Saxony	4.9	−2.7	0.0	0.0	−3.0	−0.4	−0.3	0.0	−2.5	0.2	0.4	0.8	0.3	0.0
Saxony-Anhalt	2.6	1.8	0.0	0.0	1.4	−0.3	−0.2	0.2	1.7	−0.1	0.0	0.5	0.4	0.0
Thuringia	2.5	2.0	0.1	0.0	1.3	−0.5	−0.5	−0.1	1.7	−0.1	−0.1	0.4	0.3	0.0
BIK														
BIK 1	10.8	−0.8	−0.4	0.0	−1.4	−0.9	−0.5	0.0	−1.7	−1.1	−0.6	0.0	0.3	0.0
BIK 2	34.6	−1.2	1.1	0.0	−1.8	0.6	−0.6	0.1	−1.1	1.5	0.2	0.9	0.1	0.0
BIK 3	26.8	−2.4	−0.6	0.0	−2.2	−0.4	0.2	−0.1	−2.4	−0.8	−0.2	−0.5	−0.2	0.0
BIK 4	27.9	4.1	−0.3	0.0	4.9	0.3	0.7	0.0	4.6	0.0	0.3	−0.5	−0.1	0.0
German														
Yes	85.0	14.0	13.6	12.5	16.0	15.7	15.0	12.6	18.9	18.7	18.2	16.3	13.6	13.6
No	15.0	−14.0	−13.6	−12.5	−16.0	−15.7	−15.0	−12.6	−18.9	−18.7	−18.2	−16.3	−13.6	−13.6
Education														
Low	34.4	−12.3	−12.8	−1.3	−16.2	−16.8	−5.8	−1.3	−16.9	−17.6	−7.3	−2.8	−2.6	−1.0
Medium	45.3	2.4	2.5	1.3	3.3	3.5	3.4	1.5	2.5	2.8	3.4	1.5	2.3	1.1
High	20.3	10.2	10.4	−0.2	12.6	12.9	2.3	−0.3	14.1	14.4	4.1	1.4	0.2	−0.1
Household size														
Single-person household	25.4	−3.9	−4.8	0.2	−5.0	−5.8	−0.9	−0.2	−5.0	−5.6	−0.9	−0.1	−0.4	0.3
Multi-person household	74.6	3.9	4.8	−0.2	5.0	5.8	0.9	0.2	5.0	5.6	0.9	0.1	0.4	−0.3
Health-related parameters – Internal reference values from the recruitment study														
BMI														
Normal weight (18.5 < = BMI < 25)	40.7	2.0	1.9	0.0	2.5	2.5	0.4	−0.1	2.3	2.4	0.4	−0.1	−0.1	−0.2
Underweight (BMI < 18.5)	2.0	0.2	−0.1	0.0	0.2	−0.1	−0.1	−0.1	0.1	−0.4	−0.4	−0.4	−0.1	0.1
Overweight (25 < = BMI < 30)	35.2	−0.3	−0.3	0.0	−0.7	−0.7	−0.4	0.1	0.0	−0.2	0.2	0.7	0.1	0.0
Obesity (BMI > = 30)	22.1	−2.2	−1.9	0.0	−2.3	−2.1	−0.1	0.0	−2.8	−2.5	−0.6	−0.5	0.1	0.2
Self-rated health														
Very good/good/fair	93.1	0.9	1.3	0.0	2.4	2.7	1.5	0.0	2.8	3.1	2.0	0.7	0.5	0.7
Bad/very bad	6.9	−0.9	−1.3	0.0	−2.4	−2.7	−1.5	0.0	−2.8	−3.1	−2.0	−0.7	−0.5	−0.7
Self-rated mental health														
Excellent/very good/good	78.5	1.3	1.6	0.0	1.7	1.9	0.3	0.0	2.3	2.7	1.1	0.7	0.0	0.0
fair/poor	21.5	−1.3	−1.6	0.0	−1.7	−1.9	−0.3	0.0	−2.3	−2.7	−1.1	−0.7	0.0	0.0
Paying attention to health														
Not at all/less strong/moderate	51.6	−1.7	−1.6	0.0	−2.2	−2.2	−0.5	0.0	−3.7	−3.7	−2.1	−1.4	−0.1	0.2
Strong/very strong	48.4	1.7	1.6	0.0	2.2	2.2	0.5	0.0	3.7	3.7	2.1	1.4	0.1	−0.2
Satisfaction: Life in general														
Scale value 1 to 3	8.5	−1.4	−1.5	0.0	−2.0	−2.1	−0.5	−0.1	−3.1	−3.2	−1.7	−1.3	−0.3	0.0
Scale value 4 to 7	45.1	−0.9	−1.0	0.0	−1.3	−1.4	−0.5	0.1	−2.3	−2.4	−1.5	−1.0	0.1	0.1
Scale value 8 to 10	46.4	1.7	1.8	0.0	2.4	2.5	0.7	0.0	3.9	4.1	2.4	1.7	0.1	−0.1
Red meat														
Never	9.3	1.2	0.6	0.0	1.7	1.0	0.4	0.0	1.7	1.1	0.5	0.0	−0.3	−0.1
Daily or several times a day	2.8	−1.6	−1.1	0.0	−2.1	−1.7	−0.4	0.0	−3.3	−3.0	−1.9	−1.7	−0.4	−0.2
4 to 6 times per week	12.2	−1.4	−0.4	0.0	−1.6	−0.5	0.1	0.0	−2.8	−1.7	−1.0	−1.0	−0.2	0.0
1 to 3 times per week	46.1	−0.6	−0.1	0.0	−1.1	−0.5	−0.4	0.0	−0.8	−0.3	−0.3	0.0	0.0	−0.2
Less than once per week	29.6	1.4	0.4	0.0	1.9	0.9	0.3	0.0	2.7	1.7	1.4	1.2	0.5	0.3
Sausage products														
Never	10.3	1.5	1.1	0.0	2.1	1.6	0.4	−0.1	1.7	1.2	−0.1	−0.7	−0.5	−0.5
Daily or several times a day	10.0	−0.7	−1.0	0.0	−1.2	−1.4	−0.3	0.0	−1.8	−2.1	−0.9	−0.6	−0.1	−0.1
4 to 6 times per week	19.4	−0.6	−0.2	0.0	−0.3	0.1	0.4	0.0	−0.2	0.3	0.5	0.2	0.2	0.1
1 to 3 times per week	35.6	−0.7	−0.3	0.0	−1.3	−0.8	−0.4	0.0	−0.9	−0.4	−0.1	0.4	0.0	0.0
Less than once per week	24.8	0.8	0.4	0.0	1.0	0.6	0.1	0.1	1.2	0.7	0.3	0.3	0.2	0.3
Smoking														
Non-smoker	76.2	3.5	3.2	0.0	4.1	3.8	0.5	0.0	7.4	7.1	4.4	4.1	0.8	0.6
Daily smoking	17.9	−3.9	−3.5	0.0	−4.5	−4.1	−0.5	−0.1	−7.5	−7.1	−4.2	−3.9	−0.9	−0.7
Occasional smoking	5.9	−0.1	−0.1	0.0	−0.1	−0.2	−0.1	0.0	−1.4	−1.5	−1.3	−1.1	−0.1	0.0
Chronic diseases														
No	45.0	−0.1	0.7	0.0	−0.3	0.4	−0.4	−0.1	−2.2	−1.3	−2.1	−1.8	−0.3	−0.1
Yes	55.0	0.1	−0.7	0.0	0.3	−0.4	0.4	0.1	2.2	1.3	2.1	1.8	0.3	0.1
Sport														
No sporting activities	23.0	−2.6	−3.1	0.0	−4.7	−5.2	−2.3	−0.1	−5.7	−6.4	−3.7	−1.5	−0.3	−0.2
Less than 1 h per week	19.7	−0.9	−0.5	0.0	−1.0	−0.7	−0.1	0.0	−1.8	−1.3	−0.9	−0.7	−0.1	−0.1
1 to less than 2 h per week	26.5	1.0	1.0	0.0	1.4	1.4	0.5	0.1	2.4	2.5	1.6	1.2	0.4	0.2
2 to less than 4 h per week	18.9	1.7	1.8	0.0	2.8	3.0	1.2	0.0	3.6	3.7	2.3	1.1	0.1	0.0
4 h per week and more	11.9	0.8	0.9	0.0	1.6	1.8	0.9	0.0	1.3	1.5	0.8	−0.2	−0.1	0.0
Health risk due to climate change														
Scale value 1 to 3	34.8	−0.3	−0.4	0.0	−0.8	−0.9	−0.4	0.0	−0.9	−1.0	−0.9	−0.6	0.0	−0.1
Scale value 4 to 7	49.0	0.1	0.1	0.0	0.1	0.2	0.2	0.0	0.6	0.5	0.8	0.7	0.1	0.3
Scale value 8 to 10	16.1	0.3	0.4	0.0	0.8	0.8	0.3	−0.1	0.4	0.6	0.2	−0.2	−0.1	−0.2
Waited for appointment														
No	54.8	0.9	0.3	0.0	0.6	−0.1	−0.5	0.0	2.4	1.8	1.4	1.9	0.4	0.4
Yes	33.5	−0.7	−0.3	0.0	−0.5	−0.2	0.3	0.0	−1.9	−1.7	−1.2	−1.5	−0.3	−0.3
No need for examination or treatment	11.7	−0.4	0.1	0.0	−0.1	0.3	0.3	0.0	−1.0	−0.3	−0.4	−0.8	−0.2	−0.1

^a^Negative values in the standardized differences [Δ] indicate an underrepresentation whereas positive values indicate an overrepresentation in the sample

^b^External references for sex, agegrp, federal states, BIK, German: population figures as of 31/12/2023 [39]; external references for education, household size: Microcensus 2021 [38]; internal reference for health-related parameters: recruitment study

^c^Oversampling of smaller federal states was applied in the sampling process. In the states of Berlin and Schleswig-Holstein, the number of addresses per primary sampling unit was additionally increased

Descriptive statistics and the effectiveness of the weighting factors are summarized in Table 3. A clear trend is observed, where extreme values (minimum and maximum) increase with each subsequent weighting step, a pattern broken only by the design weight due to the absence of trimming these weights.

The major loss in effectiveness occurs between the unweighted sample and the design weighting step, and with the calibration weighting of the recruitment study. Nevertheless, there is a substantial decline in effectiveness from 65% in the calibrated recruitment study to ~ 53% in the calibrated 2024 annual survey for questionnaire A to D, after accounting for drop-out and aligning with the 2024 marginal distributions.

**Table 3:** Descriptive statistics and effectiveness of weighting factors

Weighting factor	*n*. Obs.	Minimum	Median	Maximum	CV^a^	Effectiveness
Unweighted Recruitment study	61,102	1.00	1.00	1.00	0.0	100.0
Design weight Recruitment study	61,102	0.10	1.04	30.79	58.1	74.8
CW Recruitment study^b^	61,102	0.05	0.88	6.28	73.4	65.0
Drop-out weight Registration	46,863	0.06	0.84	5.76	78.9	61.6
Drop-out weight Questionnaire A	27,080	0.09	1.03	7.35	84.0	58.6
Drop-out weight Questionnaire B	27,040	0.09	1.03	7.48	85.0	58.1
Drop-out weight Questionnaire C	26,978	0.09	1.03	7.50	84.8	58.1
Drop-out weight Questionnaire D	27,179	0.09	1.03	7.33	83.9	58.7
CW Questionnaire A^b^	27,080	0.03	0.77	9.12	95.0	52.6
CW Questionnaire B^b^	27,040	0.05	0.77	8.15	93.5	53.3
CW Questionnaire C^b^	26,978	0.05	0.78	8.52	93.6	53.3
CW Questionnaire D^b^	27,179	0.05	0.77	7.94	92.6	53.8

^a^*CV* Coefficient of variation

^b^*CW* Calibration weight

## Discussion

The results illustrate how selective participation affects the sample composition of the panel ‘Health in Germany‘ and how the applied sample weighting methodology, combining design weighting, drop-out (inverse probability) weighting and calibration weighting, reduces the resulting drop-out bias in the observed parameters. The drop-out analysis indicates that sociodemographic as well as health-related factors play a role in the willingness to participate, both when registering for the panel and when taking part in the 2024 annual survey. The impact of sociodemographic parameters is considerably stronger. Notably, regarding age, younger individuals are more likely to register for the panel, whereas older panelists show a higher participation willingness in the 2024 annual survey. For the other examined parameters, the direction of selection effects observed at registration largely also persists regarding participation in the 2024 annual survey. For example, individuals who smoke or do not hold German citizenship show a lower tendency to register for the panel and, if registered, are less likely to participate in the 2024 annual survey. Consequently, these selection effects accumulate, leading to increased changes in sample composition compared to the initial recruitment study, which was used as an internal reference.

Examining the sample composition over time, reported as standardized differences, reveals at which point in the survey process drop-out has the highest effect on sample composition. As expected, distributions of parameters with negligible or minor effects in the drop-out analyses remain relatively stable from registration for the panel to participation in the 2024 annual survey, while deviations from the reference values for parameters with substantial effects increase. Notably, a significant portion of the deviations from the recruitment study observed in the samples of registered panelists and participants in the 2024 annual survey are reduced by applying the calibration weight of the recruitment study. Any remaining deviations at this weighting step (column “Calibration-weighted (Recruitment Study)” in Table 2) can be interpreted as the additional drop-out bias introduced by the panel design when compared to the (weighted) cross-sectional survey. Substantial deviations are mainly observed in the sociodemographic parameters German citizenship and education. However, for most health-related parameters, the standardized differences largely remain at a considerably lower level, typically below 3% points. Exceptions include smoking and sports, where differences range between three and five % points. While the remaining deviations are effectively reduced through drop-out weighting and final calibration weighting, resulting in standardized differences that are almost all around zero. It must be noted that these results relate to parameters which are included in the weighting. For the general case of health-related indicators which are not part of the recruitment study and thus cannot be used for weighting, the results on drop-out bias give an indication of the range of drop-out bias that might in general be expected. To what extent this drop-out bias can be reduced through the weighting depends on the correlation of the health-related indicator of interest with the variables included in the weighting.

For those variables that were included in the calibration weighting, some deviations from the external reference distributions persist even after calibration. Several factors contribute to this. First, trimming extreme weights inevitably introduces slight shifts away from the exact calibration targets. Second, due to low cell counts in some calibration categories, it was necessary to merge cells. These merged categories do not fully correspond to the more detailed structure of the reference distributions. Third, for household size and education, calibration is performed using imputed values, and education is set to the lowest category for individuals under the age of 20. In contrast, the comparisons with the reference distributions rely on data in which missing values are not imputed and education is not recoded for individuals under the age of 20. These mismatches can also lead to small remaining deviations from the reference distributions, however, the impact on the target variables of interest will be negligible.

The stability of the results for future annual panel surveys cannot be taken for granted. Since recruitment to the panel and participation in the 2024 annual survey occurred within the same year, the time lag was minimal. In later surveys, panel attrition and panel conditioning effects may alter the results, especially for health-related indicators. However, it becomes very clear that most of the change in sample composition—particularly regarding education and German citizenship, where the largest deviations are observed—already occurs during the initial recruitment study. This result is not new in the context of panel studies. Rather, it underscores how crucial it is to achieve a well-balanced sample at the very beginning of a panel study [42]. Notably, this finding is not a specific issue of panel design but also applies to cross-sectional studies. The sample adjustment through weighting to reduce nonresponse bias is limited in both designs to the available population figures. These figures are used for calibration weighting in both the recruitment study sample and the sample of 2024 annual survey participants.

An exception is German citizenship. Even after calibration and drop-out weighting, significant deviations persist for this parameter. While population figures for German citizenship are available and could be used as a separate raking dimension in calibration weighting, we decided against using it. As per design, panel participation requires adequate German language proficiency to answer German-only questionnaires, hence, a specific sub-population is systematically excluded. However, the systematically excluded population without adequate German language proficiency is not congruent with the population without German citizenship. Hence, using citizenship in the calibration weighting would introduce additional bias, as the population without German citizenship includes both: those with good as well as those with insufficient German language proficiency. Calibrating the sample to the marginal distribution of German citizenship under these conditions could assign disproportionately high weights to individuals without German citizenship and, paradoxically, may amplify existing nonresponse bias rather than reducing it.

The descriptive statistics and the effectiveness of the sample weights highlight the inherent “cost” of correcting for selective drop-out. By reducing the observed biases stemming from the sampling design and selective nonresponse during recruitment, the variance of sample estimates increases, leading to greater statistical uncertainty. For participation in the 2024 survey wave, the calculated effectiveness is around 53%. The primary reduction in effectiveness occurs during the recruitment study, where it decreases by 35%-points – nearly three times the loss due to weighting the samples (drop-out and calibration) in the 2024 annual survey. It is important to note that a significant portion of this effectiveness loss is not attributable to nonresponse in the initial sample but rather to the sampling design, which oversampled smaller federal states, with two states receiving additional oversampling. Consequently, the design weighting alone reduces effectiveness from 100% to 74.8%, with a considerable portion of this reduction stemming from the oversampling itself, which accounts for a decline to 84% when considering oversampling only. For comparison, in the most recent cross-sectional health surveys conducted at the RKI using a dual-frame telephone design (GEDA [2]), the overall effectiveness is 43%. In that study, oversampling was applied for Berlin and Saarland, but not for other smaller federal states.

### Strengths and limitations

The results demonstrate that the chosen weighting scheme effectively reduces drop-out bias caused by selective participation after the initial recruitment study, as far as it is due to variables included in the recruitment study. The weighting procedure has thus proved its potential to reduce sources of error in the TSE, particularly the drop-out error. However, the effects of selective drop-out during the survey can only be analyzed for parameters available through the recruitment study. It is possible that other important factors influencing registration/participation exist, which could potentially affect the final results. It also needs to be mentioned that the drop-out analyses do not include any stratifications, for example by age or sex. It is possible that results in subgroups might differ.

Moreover, the evaluation of sample composition is subject to the availability of valid external data sources. For health-related parameters, such an evaluation is not possible, as there are no official valid data sources available for comparison or for use in weighting. However, this is not a problem unique to the panel design. It also applies to cross-sectional studies, where individuals with certain health characteristics may be more or less willing to participate in a health survey, resulting in nonresponse bias that cannot be quantified.

The chosen method facilitates the integration of weighting factor calculations into existing data workflows and extends easily to ad-hoc studies. For future annual panel surveys, the approach can also be transferred. However, additional challenges are expected due to increasing panel attrition and the need to integrate refresher samples to maintain representativeness and consistency within the panel structure.

Researchers can easily apply the weighting factors, ensuring that their results are controlled, as far as possible, for initial nonresponse bias with respect to the external reference distributions used in calibration weighting, for drop-out bias and for the complex survey design. Incorporating them into analyses does not require extensive statistical expertise - provided that survey procedures included in all major statistical analysis packages or other procedures calculating robust standard errors are used in the analysis. The procedures used should also take the clustering in PSUs resulting from the two-stage sampling design into account to obtain valid variance estimates.

The calibration weighting uses detailed cross-classifications across five raking dimensions. As noted by Brick et al. [43], extensive raking dimensions and fine-grained cross-classifications can produce small raking cells, increasing iteration counts and potentially causing non-convergence—particularly when dimensions are correlated, as in our case with federal state and age group. These conditions also increase weight variability. To mitigate these risks, raking cells were collapsed and weight trimming was applied, ensuring convergence and maintaining acceptable coefficients of variation of the final weights (see Table 3). Convergence required 160–200 iterations for the 2024 survey questionnaires, mainly because federal state (or region) enters every raking dimension. Given the relevance of federal-state–level health policy in Germany, retaining this dimension in the calibration was essential, even at the cost of increased computational time.

It should be noted that there are differences in the regional raking dimensions between the recruitment study and the 2024 annual survey. Due to the lower number of observations in the 2024 annual survey, broader regions (North/Central-East, Northwest, Central-West, South, Berlin, Schleswig-Holstein) rather than individual federal states are used for the calibration of education. Preliminary analyses indicate that deviations from the marginal distribution at the federal state level are minor. In addition, some raking cells in the calibration weighting for the 2024 annual survey were collapsed due to small cell sizes.

For individuals under 20 years of age, educational attainment was uniformly assigned to the lowest CASMIN category, since not enough information on ongoing education in this age group was available. Consequently, researchers should be aware that, for this age group, potential nonresponse bias related to education is not accounted for by the calibration weighting.

The weighting is applied by replacing missing values through imputation during both the drop-out and calibration steps. This ensures that the weighting procedures can be applied to all observations. However, for researchers, the data is provided without the imputed values. As a consequence, bias may occur if missing values are not missing at random. Since the proportion of item missingness is very low, this distortion is considered negligible. Nevertheless, researchers must be aware that if a parameter being analyzed has missing values, the remaining complete cases might be biased, even after applying the weighting factors. Therefore, a missing data analysis is crucial before proceeding with any analysis. If significant deviations are found, additional steps must be taken to address selective nonresponse specifically for that analysis.

The automated variable selection in drop-out weighting significantly simplifies its integration into the data workflow. By including all available recruitment study variables, plus their interactions with age, sex, and education, we cover a broad spectrum of potential selection effects, including health-related variables. It should be noted, however, that potential factors influencing registration or participation which were not captured in the initial recruitment study cannot be included in the variable selection. Furthermore, interactions between variables other than age, sex, and education are not considered, meaning specific drop-out biases in the registration and annual survey participation process may remain unaccounted for. Manually checking for these additional interactions for every study or subpopulation is impractical, hindering fast implementation for other studies. Similarly, integrating more sophisticated variable selection methods (like random forest, which automatically tests for interactions) is challenging due to the current data workflow’s limitations regarding comprehensive computational methods.

### Outlook

The current weighting scheme will be applied in subsequent annual surveys, maintaining the structure of four separate quarterly sub-waves. However, the methodology will be adapted as more information on registered panelists becomes available from prior years’ regular annual surveys. This additional data can be used in the drop-out weighting to improve the estimation of re-participation. This is particularly important because the parameters from the initial recruitment study will become outdated for future weighting, and the long-term validity of this information for drop-out weighting is uncertain. Furthermore, the weighting methods, including drop-out and calibration weighting, will also be applied in the ad-hoc studies. These studies can be conducted several times a year and are designed as short interviews focusing on specific topics or particular subpopulations within the panel. It is straightforward to utilize the existing panelist information for drop-out weighting and subsequently perform calibration weighting using marginal distributions relevant to the ad-hoc study’s population. Notably, marginal distributions from the panel or the recruitment study itself can also serve as raking dimension in the calibration weighting.

Certain analyses necessitate the standardization of prevalence estimates for comparisons between specified groups (e.g., federal states) or across various time points. This procedure mitigates the impact of alterations in population composition that could confound the results. Accordingly, the final weighting factors will be standardized to the European standard population [44], employing stratification by: sex and age, federal state, sex, age and federal state, sex, education (CASMIN).

In the forthcoming year, a panel revision process will be initiated to remove registered panelists who have not participated in any regular annual survey. A detailed analysis of the attrition pattern is deemed critical. This analysis aims to inform the development of robust methodological approaches for mitigating potential drop-out biases stemming from such attrition, as this paper already showed. This process will also clarify the optimal timing for recruiting a new refreshment sample to compensate for any resulting underrepresentation within specific population strata. These new refreshment studies can then attempt to address at least some of the limitations of the first recruitment study (such as the lack of multilingual questionnaires).

## Conclusion

In this paper, we have highlighted the selection effects caused by selective drop-out, tracing them from the recruiting study to the first regular annual survey. We have shown that our chosen sample weighting methods substantially reduce the drop-out bias observed over the course of the panel for parameters collected in the recruitment study. For most health-related parameters, the estimated drop-out bias is considerably low, ranging from 1% to 3% in standardized differences. This indicates that the resulting data remain largely comparable to what would be expected from a cross-sectional study design. This needs to be further analyzed in forthcoming annual panel surveys, as the sample composition may change due to future panel attrition.

## Supplementary Information

Supplementary Material 1.

Supplementary Material 2.

## Data Availability

The data from surveys conducted by the Robert Koch Institute is available free of charge to use by the scientific community as Scientific Use Files. Each Scientific Use File is comprised of the respective data record along with documentation and a description of the study, sample survey documents, code plan and user instructions. The data records can be obtained by submitting an application form through the Research Data Centre (Robert Koch Institute, MF4), which can be accessed at www.rki.de/fdz.
