# Supplementary material for: Weighting strategy and selection analysis in the panel ‘Health in Germany‘: methods and results for the 2024 annual survey
Source: BMC Med Res Methodol. 2025 Dec 15;26:8. doi: 10.1186/s12874-025-02740-w (PMC12822093; doi:10.1186/s12874-025-02740-w)
Supplement: Supplementary file 1 — Supplementary Material 1. [file 12874_2025_2740_MOESM1_ESM.html]

Additional File 1 - Model estimation


- Additional File 1 - Model estimation

- Selection analysis
  - Variables
  - Result model
    estimation: Registration
  - Result model
    estimation: Questionaire A
  - Result model
    estimation: Questionaire B
  - Result model
    estimation: Questionaire C
  - Result model
    estimation: Questionaire D
- Drop-out weighting
  - Variables
  - Result model
    estimation: Registration
  - Result model
    estimation: Questionaire A
  - Result model
    estimation: Questionaire B
  - Result model
    estimation: Questionaire C
  - Result model
    estimation: Questionaire D

- 2025-10-21

# Additional File 1 - Model estimation

# Selection analysis

## Variables

| Characteristic | n.Obs | Percent |
| --- | --- | --- |
| Registered |  |  |
| 0 | 14,239 | 23.3% |
| 1 | 46,863 | 76.7% |
| Age group |  |  |
| 18-29 yrs | 10,021 | 16.4% |
| 30-39 yrs | 9,277 | 15.2% |
| 40-49 yrs | 7,391 | 12.1% |
| 50-59 yrs | 10,919 | 17.9% |
| 60-69 yrs | 10,025 | 16.4% |
| 70-79 yrs | 7,934 | 13.0% |
| 80+ yrs | 5,535 | 9.1% |
| Sex |  |  |
| Male | 28,695 | 47.0% |
| Female | 32,407 | 53.0% |
| Education: Casmin |  |  |
| Low | 13,929 | 22.8% |
| Medium | 29,003 | 47.5% |
| High | 17,991 | 29.4% |
| Missing | 179 | 0.3% |
| BIK community size (categorized) |  |  |
| BIK 1 | 6,273 | 10.3% |
| BIK 2 | 20,409 | 33.4% |
| BIK 3 | 15,060 | 24.6% |
| BIK 4 | 19,360 | 31.7% |
| Region |  |  |
| Northeast | 10,018 | 16.4% |
| Northwest | 14,581 | 23.9% |
| Central-East | 6,270 | 10.3% |
| Central-West | 17,082 | 28.0% |
| South | 13,151 | 21.5% |
| German Citizenship |  |  |
| No | 3,863 | 6.3% |
| Yes | 57,006 | 93.3% |
| Missing | 233 | 0.4% |
| Household size |  |  |
| Single-person household | 13,681 | 22.4% |
| Multi-person household | 47,260 | 77.4% |
| Missing | 161 | 0.3% |
| BMI category |  |  |
| Normal weight (18.5 <= BMI < 25) | 25,930 | 42.4% |
| Underweight (BMI < 18.5) | 1,272 | 2.1% |
| Overweight (25 <= BMI < 30) | 21,169 | 34.6% |
| Obesity (BMI >= 30) | 12,297 | 20.1% |
| Missing | 434 | 0.7% |
| Self-rated health |  |  |
| Very good/good/fair | 57,125 | 93.5% |
| Bad/very bad | 3,906 | 6.4% |
| Missing | 71 | 0.1% |
| Self-rated mental health |  |  |
| Excellent/very good/good | 48,511 | 79.4% |
| fair/poor | 12,494 | 20.4% |
| Missing | 97 | 0.2% |
| Paying attention to health |  |  |
| Not at all/less strong/moderate | 30,495 | 49.9% |
| Strong/very strong | 30,557 | 50.0% |
| Missing | 50 | 0.1% |
| Satisfaction: Life in general |  |  |
| Scale value 1 to 3 | 4,689 | 7.7% |
| Scale value 4 to 7 | 26,960 | 44.1% |
| Scale value 8 to 10 | 29,352 | 48.0% |
| Missing | 101 | 0.2% |
| Red meat |  |  |
| Never | 6,115 | 10.0% |
| Daily or several times a day | 1,398 | 2.3% |
| 4 to 6 times per week | 6,881 | 11.3% |
| 1 to 3 times per week | 27,799 | 45.5% |
| Less than once per week | 18,848 | 30.8% |
| Missing | 61 | 0.1% |
| Sausage products |  |  |
| Never | 6,855 | 11.2% |
| Daily or several times a day | 5,823 | 9.5% |
| 4 to 6 times per week | 11,535 | 18.9% |
| 1 to 3 times per week | 21,297 | 34.8% |
| Less than once per week | 15,532 | 25.4% |
| Missing | 60 | 0.1% |
| Smoking |  |  |
| Non-smoker | 48,274 | 79.0% |
| Daily smoking | 9,134 | 15.0% |
| Occasional smoking | 3,599 | 5.9% |
| Missing | 95 | 0.2% |
| Chronic diseases |  |  |
| No | 27,367 | 44.8% |
| Yes | 33,585 | 55.0% |
| Missing | 150 | 0.2% |
| Sport |  |  |
| No sporting activities | 12,756 | 20.9% |
| Less than 1 hour per week | 11,589 | 19.0% |
| 1 to less than 2 hours per week | 16,684 | 27.3% |
| 2 to less than 4 hours per week | 12,347 | 20.2% |
| 4 hours per week and more | 7,561 | 12.4% |
| Missing | 165 | 0.3% |
| Health risk due to climate change |  |  |
| Scale value 1 to 3 | 21,059 | 34.5% |
| Scale value 4 to 7 | 29,968 | 49.0% |
| Scale value 8 to 10 | 9,950 | 16.3% |
| Missing | 125 | 0.2% |
| Waited for appointment |  |  |
| No | 33,929 | 55.5% |
| Yes | 20,000 | 32.7% |
| No need for examination or treatment | 7,003 | 11.5% |
| Missing | 170 | 0.3% |

## Result model estimation: Registration

| Characteristic | log(OR) | SE | 95% CI | p-value | p-value (global) |
| --- | --- | --- | --- | --- | --- |
| Agegrp |  |  |  |  | <0.001 |
| 18-29 yrs | — | — | — |  |  |
| 30-39 yrs | 0.07 | 0.053 | -0.04, 0.17 | 0.2 |  |
| 40-49 yrs | -0.09 | 0.055 | -0.20, 0.02 | 0.11 |  |
| 50-59 yrs | -0.19 | 0.052 | -0.29, -0.09 | <0.001 |  |
| 60-69 yrs | -0.26 | 0.049 | -0.35, -0.16 | <0.001 |  |
| 70-79 yrs | -0.42 | 0.056 | -0.53, -0.31 | <0.001 |  |
| 80+ yrs | -0.75 | 0.058 | -0.86, -0.64 | <0.001 |  |
| Sex |  |  |  |  | 0.871 |
| Male | — | — | — |  |  |
| Female | 0.00 | 0.027 | -0.05, 0.06 | 0.9 |  |
| Education |  |  |  |  | <0.001 |
| Low | — | — | — |  |  |
| Medium | 0.46 | 0.034 | 0.40, 0.53 | <0.001 |  |
| High | 0.73 | 0.039 | 0.65, 0.80 | <0.001 |  |
| BIK |  |  |  |  | 0.013 |
| BIK 1 | — | — | — |  |  |
| BIK 2 | 0.01 | 0.055 | -0.10, 0.11 | >0.9 |  |
| BIK 3 | 0.09 | 0.056 | -0.02, 0.20 | 0.10 |  |
| BIK 4 | 0.11 | 0.056 | 0.00, 0.22 | 0.051 |  |
| Region |  |  |  |  | <0.001 |
| Northeast | — | — | — |  |  |
| Northwest | 0.12 | 0.050 | 0.03, 0.22 | 0.014 |  |
| Central-East | -0.07 | 0.049 | -0.17, 0.02 | 0.15 |  |
| Central-West | 0.13 | 0.045 | 0.04, 0.22 | 0.004 |  |
| South | 0.11 | 0.047 | 0.01, 0.20 | 0.026 |  |
| German |  |  |  |  | <0.001 |
| No | — | — | — |  |  |
| Yes | 0.73 | 0.049 | 0.63, 0.82 | <0.001 |  |
| Household size |  |  |  |  |  |
| Single-person household | — | — | — |  |  |
| Multi-person household | 0.08 | 0.030 | 0.02, 0.13 | 0.014 |  |
| BMI |  |  |  |  | 0.075 |
| Normal weight (18.5 <= BMI < 25) | — | — | — |  |  |
| Underweight (BMI < 18.5) | -0.01 | 0.092 | -0.20, 0.17 | 0.9 |  |
| Overweight (25 <= BMI < 30) | 0.02 | 0.031 | -0.04, 0.08 | 0.6 |  |
| Obesity (BMI >= 30) | 0.08 | 0.033 | 0.02, 0.15 | 0.011 |  |
| Self-rated health |  |  |  |  | <0.001 |
| Very good/good/fair | — | — | — |  |  |
| Bad/very bad | -0.19 | 0.053 | -0.29, -0.08 | <0.001 |  |
| Self-rated mental health |  |  |  |  | 0.04 |
| Excellent/very good/good | — | — | — |  |  |
| fair/poor | 0.07 | 0.036 | 0.00, 0.14 | 0.041 |  |
| Paying attention to health |  |  |  |  | 0.332 |
| Not at all/less strong/moderate | — | — | — |  |  |
| Strong/very strong | 0.03 | 0.028 | -0.03, 0.08 | 0.3 |  |
| Satisfaction: Life in general |  |  |  |  | 0.03 |
| Scale value 1 to 3 | — | — | — |  |  |
| Scale value 4 to 7 | 0.01 | 0.052 | -0.09, 0.12 | 0.8 |  |
| Scale value 8 to 10 | 0.08 | 0.053 | -0.02, 0.19 | 0.12 |  |
| Red meat |  |  |  |  | 0.033 |
| Never | — | — | — |  |  |
| Daily or several times a day | -0.24 | 0.107 | -0.45, -0.03 | 0.026 |  |
| 4 to 6 times per week | -0.10 | 0.063 | -0.22, 0.03 | 0.12 |  |
| 1 to 3 times per week | -0.08 | 0.058 | -0.20, 0.03 | 0.2 |  |
| Less than once per week | -0.01 | 0.054 | -0.12, 0.09 | 0.8 |  |
| Sausage products |  |  |  |  | 0.065 |
| Never | — | — | — |  |  |
| Daily or several times a day | 0.08 | 0.074 | -0.06, 0.23 | 0.3 |  |
| 4 to 6 times per week | 0.14 | 0.062 | 0.02, 0.26 | 0.025 |  |
| 1 to 3 times per week | 0.05 | 0.056 | -0.07, 0.16 | 0.4 |  |
| Less than once per week | 0.05 | 0.055 | -0.06, 0.16 | 0.3 |  |
| Smoking |  |  |  |  | 0.008 |
| Non-smoker | — | — | — |  |  |
| Daily smoking | -0.04 | 0.034 | -0.11, 0.03 | 0.3 |  |
| Occasional smoking | -0.17 | 0.055 | -0.28, -0.06 | 0.003 |  |
| Chronic diseases |  |  |  |  | <0.001 |
| No | — | — | — |  |  |
| Yes | 0.27 | 0.028 | 0.21, 0.32 | <0.001 |  |
| Sport |  |  |  |  | <0.001 |
| No sporting activities | — | — | — |  |  |
| Less than 1 hour per week | 0.12 | 0.039 | 0.04, 0.19 | 0.003 |  |
| 1 to less than 2 hours per week | 0.18 | 0.039 | 0.10, 0.26 | <0.001 |  |
| 2 to less than 4 hours per week | 0.25 | 0.043 | 0.17, 0.34 | <0.001 |  |
| 4 hours per week and more | 0.28 | 0.050 | 0.19, 0.38 | <0.001 |  |
| Health risk due to climate change |  |  |  |  | 0.019 |
| Scale value 1 to 3 | — | — | — |  |  |
| Scale value 4 to 7 | 0.07 | 0.027 | 0.02, 0.13 | 0.006 |  |
| Scale value 8 to 10 | 0.04 | 0.039 | -0.04, 0.11 | 0.4 |  |
| Waited for medical examination date in the last 12 months |  |  |  |  | 0.222 |
| No | — | — | — |  |  |
| Yes | -0.05 | 0.029 | -0.10, 0.01 | 0.10 |  |
| No need for examination or treatment | -0.05 | 0.043 | -0.13, 0.04 | 0.3 |  |
| N.Obs | 59,580 |  |  |  |  |
| N.Cluster | 359 |  |  |  |  |
|  |  |  |  |  |  |
| --- | --- | --- | --- | --- | --- |
| Abbreviations: CI = Confidence Interval, OR = Odds Ratio, SE = Standard Error | | | | | |

## Result model estimation: Questionaire A

| Characteristic | log(OR) | SE | 95% CI | p-value | p-value (global) |
| --- | --- | --- | --- | --- | --- |
| Agegrp |  |  |  |  | <0.001 |
| 18-29 yrs | — | — | — |  |  |
| 30-39 yrs | 0.21 | 0.053 | 0.11, 0.32 | <0.001 |  |
| 40-49 yrs | 0.51 | 0.053 | 0.40, 0.61 | <0.001 |  |
| 50-59 yrs | 0.92 | 0.057 | 0.80, 1.0 | <0.001 |  |
| 60-69 yrs | 1.3 | 0.067 | 1.2, 1.5 | <0.001 |  |
| 70-79 yrs | 1.5 | 0.083 | 1.3, 1.6 | <0.001 |  |
| 80+ yrs | 1.1 | 0.084 | 0.90, 1.2 | <0.001 |  |
| Sex |  |  |  |  | <0.001 |
| Male | — | — | — |  |  |
| Female | 0.17 | 0.038 | 0.09, 0.24 | <0.001 |  |
| Education |  |  |  |  | <0.001 |
| Low | — | — | — |  |  |
| Medium | 0.30 | 0.049 | 0.21, 0.40 | <0.001 |  |
| High | 0.47 | 0.058 | 0.36, 0.59 | <0.001 |  |
| BIK |  |  |  |  | 0.35 |
| BIK 1 | — | — | — |  |  |
| BIK 2 | 0.13 | 0.096 | -0.06, 0.32 | 0.2 |  |
| BIK 3 | 0.04 | 0.110 | -0.18, 0.26 | 0.7 |  |
| BIK 4 | 0.07 | 0.101 | -0.12, 0.27 | 0.5 |  |
| Region |  |  |  |  | 0.021 |
| Northeast | — | — | — |  |  |
| Northwest | 0.01 | 0.084 | -0.15, 0.18 | 0.9 |  |
| Central-East | 0.19 | 0.069 | 0.05, 0.32 | 0.007 |  |
| Central-West | 0.06 | 0.077 | -0.10, 0.21 | 0.5 |  |
| South | 0.16 | 0.068 | 0.02, 0.29 | 0.022 |  |
| German |  |  |  |  | <0.001 |
| No | — | — | — |  |  |
| Yes | 0.64 | 0.068 | 0.50, 0.77 | <0.001 |  |
| Household size |  |  |  |  |  |
| Single-person household | — | — | — |  |  |
| Multi-person household | -0.02 | 0.040 | -0.10, 0.06 | 0.6 |  |
| BMI |  |  |  |  | 0.042 |
| Normal weight (18.5 <= BMI < 25) | — | — | — |  |  |
| Underweight (BMI < 18.5) | 0.02 | 0.109 | -0.20, 0.23 | 0.9 |  |
| Overweight (25 <= BMI < 30) | -0.01 | 0.040 | -0.09, 0.06 | 0.7 |  |
| Obesity (BMI >= 30) | -0.14 | 0.051 | -0.24, -0.04 | 0.006 |  |
| Self-rated health |  |  |  |  | 0.003 |
| Very good/good/fair | — | — | — |  |  |
| Bad/very bad | -0.22 | 0.076 | -0.37, -0.07 | 0.004 |  |
| Self-rated mental health |  |  |  |  | 0.074 |
| Excellent/very good/good | — | — | — |  |  |
| fair/poor | 0.09 | 0.051 | -0.01, 0.19 | 0.075 |  |
| Paying attention to health |  |  |  |  | 0.86 |
| Not at all/less strong/moderate | — | — | — |  |  |
| Strong/very strong | 0.01 | 0.035 | -0.06, 0.08 | 0.9 |  |
| Satisfaction: Life in general |  |  |  |  | <0.001 |
| Scale value 1 to 3 | — | — | — |  |  |
| Scale value 4 to 7 | 0.08 | 0.076 | -0.07, 0.23 | 0.3 |  |
| Scale value 8 to 10 | 0.23 | 0.082 | 0.07, 0.39 | 0.006 |  |
| Red meat |  |  |  |  | 0.05 |
| Never | — | — | — |  |  |
| Daily or several times a day | -0.33 | 0.139 | -0.60, -0.05 | 0.020 |  |
| 4 to 6 times per week | -0.19 | 0.090 | -0.37, -0.02 | 0.032 |  |
| 1 to 3 times per week | -0.17 | 0.082 | -0.33, -0.01 | 0.037 |  |
| Less than once per week | -0.09 | 0.080 | -0.25, 0.07 | 0.3 |  |
| Sausage products |  |  |  |  | >0.9 |
| Never | — | — | — |  |  |
| Daily or several times a day | 0.01 | 0.094 | -0.18, 0.19 | >0.9 |  |
| 4 to 6 times per week | 0.02 | 0.081 | -0.14, 0.18 | 0.8 |  |
| 1 to 3 times per week | -0.01 | 0.075 | -0.16, 0.13 | 0.8 |  |
| Less than once per week | -0.01 | 0.070 | -0.15, 0.13 | >0.9 |  |
| Smoking |  |  |  |  | <0.001 |
| Non-smoker | — | — | — |  |  |
| Daily smoking | -0.51 | 0.047 | -0.60, -0.42 | <0.001 |  |
| Occasional smoking | -0.26 | 0.065 | -0.39, -0.14 | <0.001 |  |
| Chronic diseases |  |  |  |  | 0.019 |
| No | — | — | — |  |  |
| Yes | 0.10 | 0.041 | 0.02, 0.17 | 0.020 |  |
| Sport |  |  |  |  | 0.002 |
| No sporting activities | — | — | — |  |  |
| Less than 1 hour per week | 0.09 | 0.053 | -0.01, 0.20 | 0.087 |  |
| 1 to less than 2 hours per week | 0.20 | 0.054 | 0.09, 0.30 | <0.001 |  |
| 2 to less than 4 hours per week | 0.20 | 0.056 | 0.09, 0.31 | <0.001 |  |
| 4 hours per week and more | 0.10 | 0.063 | -0.03, 0.22 | 0.13 |  |
| Health risk due to climate change |  |  |  |  | 0.006 |
| Scale value 1 to 3 | — | — | — |  |  |
| Scale value 4 to 7 | 0.12 | 0.039 | 0.05, 0.20 | 0.002 |  |
| Scale value 8 to 10 | 0.07 | 0.055 | -0.04, 0.18 | 0.2 |  |
| Waited for medical examination date in the last 12 months |  |  |  |  | 0.001 |
| No | — | — | — |  |  |
| Yes | -0.10 | 0.036 | -0.17, -0.03 | 0.005 |  |
| No need for examination or treatment | 0.06 | 0.051 | -0.04, 0.16 | 0.3 |  |
| N.Obs | 34,176 |  |  |  |  |
| N.Cluster | 359 |  |  |  |  |
|  |  |  |  |  |  |
| --- | --- | --- | --- | --- | --- |
| Abbreviations: CI = Confidence Interval, OR = Odds Ratio, SE = Standard Error | | | | | |

## Result model estimation: Questionaire B

| Characteristic | log(OR) | SE | 95% CI | p-value | p-value (global) |
| --- | --- | --- | --- | --- | --- |
| Agegrp |  |  |  |  | <0.001 |
| 18-29 yrs | — | — | — |  |  |
| 30-39 yrs | 0.31 | 0.052 | 0.21, 0.41 | <0.001 |  |
| 40-49 yrs | 0.53 | 0.055 | 0.43, 0.64 | <0.001 |  |
| 50-59 yrs | 1.0 | 0.057 | 0.89, 1.1 | <0.001 |  |
| 60-69 yrs | 1.4 | 0.069 | 1.2, 1.5 | <0.001 |  |
| 70-79 yrs | 1.6 | 0.075 | 1.4, 1.7 | <0.001 |  |
| 80+ yrs | 1.1 | 0.090 | 0.95, 1.3 | <0.001 |  |
| Sex |  |  |  |  | <0.001 |
| Male | — | — | — |  |  |
| Female | 0.23 | 0.037 | 0.16, 0.30 | <0.001 |  |
| Education |  |  |  |  | <0.001 |
| Low | — | — | — |  |  |
| Medium | 0.42 | 0.050 | 0.32, 0.51 | <0.001 |  |
| High | 0.53 | 0.057 | 0.42, 0.64 | <0.001 |  |
| BIK |  |  |  |  | 0.86 |
| BIK 1 | — | — | — |  |  |
| BIK 2 | 0.09 | 0.123 | -0.15, 0.34 | 0.4 |  |
| BIK 3 | 0.09 | 0.131 | -0.17, 0.34 | 0.5 |  |
| BIK 4 | 0.06 | 0.131 | -0.20, 0.32 | 0.7 |  |
| Region |  |  |  |  | 0.066 |
| Northeast | — | — | — |  |  |
| Northwest | 0.10 | 0.097 | -0.09, 0.30 | 0.3 |  |
| Central-East | 0.17 | 0.082 | 0.00, 0.33 | 0.045 |  |
| Central-West | 0.07 | 0.088 | -0.10, 0.24 | 0.4 |  |
| South | 0.20 | 0.081 | 0.05, 0.36 | 0.012 |  |
| German |  |  |  |  | <0.001 |
| No | — | — | — |  |  |
| Yes | 0.71 | 0.073 | 0.56, 0.85 | <0.001 |  |
| Household size |  |  |  |  |  |
| Single-person household | — | — | — |  |  |
| Multi-person household | 0.03 | 0.042 | -0.05, 0.11 | 0.4 |  |
| BMI |  |  |  |  | 0.139 |
| Normal weight (18.5 <= BMI < 25) | — | — | — |  |  |
| Underweight (BMI < 18.5) | -0.06 | 0.104 | -0.27, 0.14 | 0.5 |  |
| Overweight (25 <= BMI < 30) | -0.05 | 0.039 | -0.13, 0.03 | 0.2 |  |
| Obesity (BMI >= 30) | -0.11 | 0.048 | -0.20, -0.01 | 0.023 |  |
| Self-rated health |  |  |  |  | 0.008 |
| Very good/good/fair | — | — | — |  |  |
| Bad/very bad | -0.20 | 0.077 | -0.35, -0.05 | 0.008 |  |
| Self-rated mental health |  |  |  |  | 0.721 |
| Excellent/very good/good | — | — | — |  |  |
| fair/poor | 0.02 | 0.049 | -0.08, 0.11 | 0.7 |  |
| Paying attention to health |  |  |  |  | 0.305 |
| Not at all/less strong/moderate | — | — | — |  |  |
| Strong/very strong | -0.04 | 0.039 | -0.12, 0.04 | 0.3 |  |
| Satisfaction: Life in general |  |  |  |  | <0.001 |
| Scale value 1 to 3 | — | — | — |  |  |
| Scale value 4 to 7 | 0.02 | 0.068 | -0.11, 0.16 | 0.7 |  |
| Scale value 8 to 10 | 0.17 | 0.074 | 0.02, 0.31 | 0.026 |  |
| Red meat |  |  |  |  | 0.031 |
| Never | — | — | — |  |  |
| Daily or several times a day | -0.31 | 0.122 | -0.55, -0.07 | 0.012 |  |
| 4 to 6 times per week | -0.24 | 0.091 | -0.42, -0.06 | 0.009 |  |
| 1 to 3 times per week | -0.18 | 0.084 | -0.34, -0.01 | 0.038 |  |
| Less than once per week | -0.12 | 0.080 | -0.28, 0.04 | 0.13 |  |
| Sausage products |  |  |  |  | 0.718 |
| Never | — | — | — |  |  |
| Daily or several times a day | 0.07 | 0.092 | -0.12, 0.25 | 0.5 |  |
| 4 to 6 times per week | 0.09 | 0.081 | -0.07, 0.25 | 0.3 |  |
| 1 to 3 times per week | 0.04 | 0.078 | -0.12, 0.19 | 0.6 |  |
| Less than once per week | 0.07 | 0.076 | -0.08, 0.22 | 0.4 |  |
| Smoking |  |  |  |  | <0.001 |
| Non-smoker | — | — | — |  |  |
| Daily smoking | -0.42 | 0.046 | -0.51, -0.33 | <0.001 |  |
| Occasional smoking | -0.17 | 0.063 | -0.30, -0.05 | 0.006 |  |
| Chronic diseases |  |  |  |  | 0.007 |
| No | — | — | — |  |  |
| Yes | 0.10 | 0.038 | 0.03, 0.18 | 0.007 |  |
| Sport |  |  |  |  | <0.001 |
| No sporting activities | — | — | — |  |  |
| Less than 1 hour per week | 0.09 | 0.058 | -0.02, 0.21 | 0.11 |  |
| 1 to less than 2 hours per week | 0.22 | 0.051 | 0.13, 0.32 | <0.001 |  |
| 2 to less than 4 hours per week | 0.22 | 0.058 | 0.11, 0.33 | <0.001 |  |
| 4 hours per week and more | 0.14 | 0.065 | 0.01, 0.27 | 0.038 |  |
| Health risk due to climate change |  |  |  |  | 0.318 |
| Scale value 1 to 3 | — | — | — |  |  |
| Scale value 4 to 7 | 0.04 | 0.038 | -0.03, 0.12 | 0.3 |  |
| Scale value 8 to 10 | 0.07 | 0.050 | -0.03, 0.17 | 0.2 |  |
| Waited for medical examination date in the last 12 months |  |  |  |  | 0.027 |
| No | — | — | — |  |  |
| Yes | -0.10 | 0.038 | -0.17, -0.02 | 0.010 |  |
| No need for examination or treatment | -0.01 | 0.049 | -0.11, 0.09 | 0.9 |  |
| N.Obs | 34,213 |  |  |  |  |
| N.Cluster | 359 |  |  |  |  |
|  |  |  |  |  |  |
| --- | --- | --- | --- | --- | --- |
| Abbreviations: CI = Confidence Interval, OR = Odds Ratio, SE = Standard Error | | | | | |

## Result model estimation: Questionaire C

| Characteristic | log(OR) | SE | 95% CI | p-value | p-value (global) |
| --- | --- | --- | --- | --- | --- |
| Agegrp |  |  |  |  | <0.001 |
| 18-29 yrs | — | — | — |  |  |
| 30-39 yrs | 0.32 | 0.059 | 0.20, 0.43 | <0.001 |  |
| 40-49 yrs | 0.49 | 0.056 | 0.38, 0.60 | <0.001 |  |
| 50-59 yrs | 0.93 | 0.057 | 0.82, 1.0 | <0.001 |  |
| 60-69 yrs | 1.3 | 0.069 | 1.2, 1.5 | <0.001 |  |
| 70-79 yrs | 1.4 | 0.079 | 1.3, 1.6 | <0.001 |  |
| 80+ yrs | 1.1 | 0.093 | 0.96, 1.3 | <0.001 |  |
| Sex |  |  |  |  | <0.001 |
| Male | — | — | — |  |  |
| Female | 0.18 | 0.038 | 0.11, 0.26 | <0.001 |  |
| Education |  |  |  |  | <0.001 |
| Low | — | — | — |  |  |
| Medium | 0.38 | 0.053 | 0.28, 0.48 | <0.001 |  |
| High | 0.54 | 0.058 | 0.43, 0.65 | <0.001 |  |
| BIK |  |  |  |  | 0.52 |
| BIK 1 | — | — | — |  |  |
| BIK 2 | 0.15 | 0.109 | -0.06, 0.37 | 0.2 |  |
| BIK 3 | 0.10 | 0.118 | -0.13, 0.33 | 0.4 |  |
| BIK 4 | 0.12 | 0.115 | -0.11, 0.35 | 0.3 |  |
| Region |  |  |  |  | 0.403 |
| Northeast | — | — | — |  |  |
| Northwest | 0.06 | 0.089 | -0.12, 0.23 | 0.5 |  |
| Central-East | 0.08 | 0.075 | -0.07, 0.23 | 0.3 |  |
| Central-West | 0.02 | 0.083 | -0.15, 0.18 | 0.8 |  |
| South | 0.12 | 0.074 | -0.03, 0.27 | 0.10 |  |
| German |  |  |  |  | <0.001 |
| No | — | — | — |  |  |
| Yes | 0.76 | 0.070 | 0.63, 0.90 | <0.001 |  |
| Household size |  |  |  |  |  |
| Single-person household | — | — | — |  |  |
| Multi-person household | 0.04 | 0.042 | -0.04, 0.12 | 0.4 |  |
| BMI |  |  |  |  | 0.18 |
| Normal weight (18.5 <= BMI < 25) | — | — | — |  |  |
| Underweight (BMI < 18.5) | -0.02 | 0.109 | -0.23, 0.20 | 0.9 |  |
| Overweight (25 <= BMI < 30) | -0.07 | 0.041 | -0.15, 0.01 | 0.11 |  |
| Obesity (BMI >= 30) | -0.10 | 0.049 | -0.20, 0.00 | 0.041 |  |
| Self-rated health |  |  |  |  | <0.001 |
| Very good/good/fair | — | — | — |  |  |
| Bad/very bad | -0.42 | 0.079 | -0.58, -0.27 | <0.001 |  |
| Self-rated mental health |  |  |  |  | 0.095 |
| Excellent/very good/good | — | — | — |  |  |
| fair/poor | -0.08 | 0.050 | -0.18, 0.01 | 0.10 |  |
| Paying attention to health |  |  |  |  | >0.9 |
| Not at all/less strong/moderate | — | — | — |  |  |
| Strong/very strong | 0.00 | 0.037 | -0.07, 0.08 | >0.9 |  |
| Satisfaction: Life in general |  |  |  |  | 0.002 |
| Scale value 1 to 3 | — | — | — |  |  |
| Scale value 4 to 7 | -0.07 | 0.074 | -0.22, 0.08 | 0.3 |  |
| Scale value 8 to 10 | 0.06 | 0.077 | -0.09, 0.21 | 0.4 |  |
| Red meat |  |  |  |  | 0.003 |
| Never | — | — | — |  |  |
| Daily or several times a day | -0.33 | 0.130 | -0.59, -0.07 | 0.012 |  |
| 4 to 6 times per week | -0.24 | 0.092 | -0.42, -0.06 | 0.009 |  |
| 1 to 3 times per week | -0.14 | 0.081 | -0.30, 0.02 | 0.079 |  |
| Less than once per week | -0.05 | 0.080 | -0.21, 0.10 | 0.5 |  |
| Sausage products |  |  |  |  | 0.006 |
| Never | — | — | — |  |  |
| Daily or several times a day | 0.16 | 0.090 | -0.02, 0.34 | 0.077 |  |
| 4 to 6 times per week | 0.19 | 0.082 | 0.03, 0.35 | 0.020 |  |
| 1 to 3 times per week | 0.03 | 0.075 | -0.12, 0.18 | 0.7 |  |
| Less than once per week | 0.08 | 0.071 | -0.06, 0.22 | 0.2 |  |
| Smoking |  |  |  |  | <0.001 |
| Non-smoker | — | — | — |  |  |
| Daily smoking | -0.54 | 0.046 | -0.63, -0.45 | <0.001 |  |
| Occasional smoking | -0.31 | 0.063 | -0.43, -0.19 | <0.001 |  |
| Chronic diseases |  |  |  |  | <0.001 |
| No | — | — | — |  |  |
| Yes | 0.15 | 0.038 | 0.07, 0.22 | <0.001 |  |
| Sport |  |  |  |  | 0.05 |
| No sporting activities | — | — | — |  |  |
| Less than 1 hour per week | 0.02 | 0.056 | -0.09, 0.13 | 0.8 |  |
| 1 to less than 2 hours per week | 0.12 | 0.052 | 0.01, 0.22 | 0.028 |  |
| 2 to less than 4 hours per week | 0.13 | 0.056 | 0.02, 0.24 | 0.023 |  |
| 4 hours per week and more | 0.03 | 0.064 | -0.09, 0.16 | 0.6 |  |
| Health risk due to climate change |  |  |  |  | 0.408 |
| Scale value 1 to 3 | — | — | — |  |  |
| Scale value 4 to 7 | 0.01 | 0.041 | -0.07, 0.09 | 0.8 |  |
| Scale value 8 to 10 | 0.07 | 0.054 | -0.04, 0.18 | 0.2 |  |
| Waited for medical examination date in the last 12 months |  |  |  |  | 0.017 |
| No | — | — | — |  |  |
| Yes | -0.07 | 0.042 | -0.16, 0.01 | 0.087 |  |
| No need for examination or treatment | 0.08 | 0.052 | -0.02, 0.19 | 0.11 |  |
| N.Obs | 34,062 |  |  |  |  |
| N.Cluster | 359 |  |  |  |  |
|  |  |  |  |  |  |
| --- | --- | --- | --- | --- | --- |
| Abbreviations: CI = Confidence Interval, OR = Odds Ratio, SE = Standard Error | | | | | |

## Result model estimation: Questionaire D

| Characteristic | log(OR) | SE | 95% CI | p-value | p-value (global) |
| --- | --- | --- | --- | --- | --- |
| Agegrp |  |  |  |  | <0.001 |
| 18-29 yrs | — | — | — |  |  |
| 30-39 yrs | 0.25 | 0.059 | 0.13, 0.36 | <0.001 |  |
| 40-49 yrs | 0.46 | 0.061 | 0.34, 0.58 | <0.001 |  |
| 50-59 yrs | 0.87 | 0.058 | 0.76, 0.99 | <0.001 |  |
| 60-69 yrs | 1.3 | 0.074 | 1.2, 1.5 | <0.001 |  |
| 70-79 yrs | 1.4 | 0.084 | 1.3, 1.6 | <0.001 |  |
| 80+ yrs | 1.1 | 0.094 | 0.88, 1.3 | <0.001 |  |
| Sex |  |  |  |  | <0.001 |
| Male | — | — | — |  |  |
| Female | 0.22 | 0.041 | 0.14, 0.30 | <0.001 |  |
| Education |  |  |  |  | <0.001 |
| Low | — | — | — |  |  |
| Medium | 0.34 | 0.047 | 0.25, 0.44 | <0.001 |  |
| High | 0.49 | 0.056 | 0.38, 0.60 | <0.001 |  |
| BIK |  |  |  |  | 0.792 |
| BIK 1 | — | — | — |  |  |
| BIK 2 | 0.05 | 0.107 | -0.16, 0.27 | 0.6 |  |
| BIK 3 | 0.02 | 0.118 | -0.22, 0.25 | 0.9 |  |
| BIK 4 | 0.08 | 0.110 | -0.14, 0.30 | 0.5 |  |
| Region |  |  |  |  | 0.479 |
| Northeast | — | — | — |  |  |
| Northwest | 0.07 | 0.094 | -0.11, 0.26 | 0.4 |  |
| Central-East | 0.11 | 0.083 | -0.06, 0.27 | 0.2 |  |
| Central-West | 0.07 | 0.082 | -0.09, 0.23 | 0.4 |  |
| South | 0.14 | 0.076 | -0.01, 0.29 | 0.076 |  |
| German |  |  |  |  | <0.001 |
| No | — | — | — |  |  |
| Yes | 0.79 | 0.075 | 0.64, 0.93 | <0.001 |  |
| Household size |  |  |  |  |  |
| Single-person household | — | — | — |  |  |
| Multi-person household | 0.01 | 0.045 | -0.08, 0.10 | 0.9 |  |
| BMI |  |  |  |  | 0.002 |
| Normal weight (18.5 <= BMI < 25) | — | — | — |  |  |
| Underweight (BMI < 18.5) | 0.08 | 0.106 | -0.13, 0.29 | 0.5 |  |
| Overweight (25 <= BMI < 30) | -0.11 | 0.039 | -0.19, -0.04 | 0.003 |  |
| Obesity (BMI >= 30) | -0.16 | 0.048 | -0.26, -0.07 | <0.001 |  |
| Self-rated health |  |  |  |  | 0.008 |
| Very good/good/fair | — | — | — |  |  |
| Bad/very bad | -0.20 | 0.074 | -0.34, -0.05 | 0.009 |  |
| Self-rated mental health |  |  |  |  | 0.514 |
| Excellent/very good/good | — | — | — |  |  |
| fair/poor | 0.03 | 0.052 | -0.07, 0.14 | 0.5 |  |
| Paying attention to health |  |  |  |  | 0.39 |
| Not at all/less strong/moderate | — | — | — |  |  |
| Strong/very strong | -0.03 | 0.036 | -0.10, 0.04 | 0.4 |  |
| Satisfaction: Life in general |  |  |  |  | 0.001 |
| Scale value 1 to 3 | — | — | — |  |  |
| Scale value 4 to 7 | 0.02 | 0.072 | -0.12, 0.16 | 0.8 |  |
| Scale value 8 to 10 | 0.15 | 0.073 | 0.01, 0.30 | 0.037 |  |
| Red meat |  |  |  |  | 0.006 |
| Never | — | — | — |  |  |
| Daily or several times a day | -0.37 | 0.132 | -0.63, -0.11 | 0.006 |  |
| 4 to 6 times per week | -0.10 | 0.098 | -0.30, 0.09 | 0.3 |  |
| 1 to 3 times per week | -0.12 | 0.083 | -0.28, 0.04 | 0.15 |  |
| Less than once per week | -0.01 | 0.080 | -0.16, 0.15 | >0.9 |  |
| Sausage products |  |  |  |  | 0.225 |
| Never | — | — | — |  |  |
| Daily or several times a day | 0.07 | 0.098 | -0.12, 0.27 | 0.4 |  |
| 4 to 6 times per week | 0.14 | 0.087 | -0.03, 0.31 | 0.11 |  |
| 1 to 3 times per week | 0.03 | 0.080 | -0.13, 0.19 | 0.7 |  |
| Less than once per week | 0.05 | 0.078 | -0.10, 0.21 | 0.5 |  |
| Smoking |  |  |  |  | <0.001 |
| Non-smoker | — | — | — |  |  |
| Daily smoking | -0.46 | 0.048 | -0.55, -0.37 | <0.001 |  |
| Occasional smoking | -0.23 | 0.068 | -0.36, -0.09 | <0.001 |  |
| Chronic diseases |  |  |  |  | 0.033 |
| No | — | — | — |  |  |
| Yes | 0.09 | 0.041 | 0.01, 0.17 | 0.034 |  |
| Sport |  |  |  |  | 0.034 |
| No sporting activities | — | — | — |  |  |
| Less than 1 hour per week | 0.06 | 0.056 | -0.05, 0.17 | 0.3 |  |
| 1 to less than 2 hours per week | 0.12 | 0.052 | 0.02, 0.22 | 0.020 |  |
| 2 to less than 4 hours per week | 0.16 | 0.054 | 0.05, 0.27 | 0.003 |  |
| 4 hours per week and more | 0.08 | 0.061 | -0.04, 0.20 | 0.2 |  |
| Health risk due to climate change |  |  |  |  | 0.223 |
| Scale value 1 to 3 | — | — | — |  |  |
| Scale value 4 to 7 | 0.04 | 0.038 | -0.03, 0.11 | 0.3 |  |
| Scale value 8 to 10 | 0.09 | 0.053 | -0.01, 0.20 | 0.089 |  |
| Waited for medical examination date in the last 12 months |  |  |  |  | 0.026 |
| No | — | — | — |  |  |
| Yes | -0.09 | 0.039 | -0.17, -0.01 | 0.023 |  |
| No need for examination or treatment | 0.03 | 0.057 | -0.09, 0.14 | 0.6 |  |
| N.Obs | 34,196 |  |  |  |  |
| N.Cluster | 359 |  |  |  |  |
|  |  |  |  |  |  |
| --- | --- | --- | --- | --- | --- |
| Abbreviations: CI = Confidence Interval, OR = Odds Ratio, SE = Standard Error | | | | | |

# Drop-out weighting

## Variables

Following variables were part of the variable selection performed by
Lasso Regression.

| Variables used in variable selection - Missing and imputed missing values | | | |
| --- | --- | --- | --- |
| Variable | Variable (imputed)1 | n.Obs | Percent |
| Age group |  |  |  |
| 16-29 yrs | 16-29 yrs | 11,088 | 17.8% |
| 30-39 yrs | 30-39 yrs | 9,277 | 14.9% |
| 40-49 yrs | 40-49 yrs | 7,391 | 11.9% |
| 50-59 yrs | 50-59 yrs | 10,919 | 17.6% |
| 60-69 yrs | 60-69 yrs | 10,025 | 16.1% |
| 70-79 yrs | 70-79 yrs | 7,934 | 12.8% |
| 80+ | 80+ | 5,535 | 8.9% |
| BIK community size (categorized) |  |  |  |
| BIK region <20,000 inhabitants | BIK region <20,000 inhabitants | 6,381 | 10.3% |
| BIK region 20,000 to <50,000 inhabitants OR surroundings 50,000 to <500,000 inhabitants | BIK region 20,000 to <50,000 inhabitants OR surroundings 50,000 to <500,000 inhabitants | 20,741 | 33.4% |
| Core city 50,000 to <500,000 inhabitants OR surroundings 500,000+ inhabitants | Core city 50,000 to <500,000 inhabitants OR surroundings 500,000+ inhabitants | 15,339 | 24.7% |
| Core city 500,000+ inhabitants | Core city 500,000+ inhabitants | 19,708 | 31.7% |
| Chronic diseases |  |  |  |
| Yes | Yes | 33,845 | 54.4% |
| No | No | 28,177 | 45.3% |
| Missing | Yes | 147 | 0.2% |
| Country of birth |  |  |  |
| In Germany (within current borders) | In Germany (within current borders) | 54,451 | 87.6% |
| In another country | In another country | 7,557 | 12.2% |
| Missing | In Germany (within current borders) | 161 | 0.3% |
| Current life situation |  |  |  |
| Full-time employed | Full-time employed | 24,633 | 39.6% |
| Part-time employed | Part-time employed | 10,185 | 16.4% |
| Unemployed | Unemployed | 2,100 | 3.4% |
| Retired or early retired | Retired or early retired | 18,934 | 30.5% |
| Not employed for other reasons (student, volunteer service, homemaker) | Not employed for other reasons (student, volunteer service, homemaker) | 6,019 | 9.7% |
| Missing | Full-time employed | 298 | 0.5% |
| Education: Casmin |  |  |  |
| Low | Low | 14,435 | 23.2% |
| Medium | Medium | 29,558 | 47.5% |
| High | High | 18,000 | 29.0% |
| Missing | Medium | 176 | 0.3% |
| Education: ISCED (2011) |  |  |  |
| Low | Low | 6,573 | 10.6% |
| Medium | Medium | 30,010 | 48.3% |
| High | High | 25,410 | 40.9% |
| Missing | Medium | 176 | 0.3% |
| Foreign nationality (registration office) |  |  |  |
| Foreign national | Foreign national | 9,026 | 14.5% |
| Not foreign national | Not foreign national | 53,143 | 85.5% |
| German Citizenship |  |  |  |
| Yes | Yes | 57,979 | 93.3% |
| No | No | 3,958 | 6.4% |
| Missing | Yes | 232 | 0.4% |
| Health risk due to climate change |  |  |  |
| Scale value 1 to 3 | Scale value 1 to 3 | 21,345 | 34.3% |
| Scale value 4 to 7 | Scale value 4 to 7 | 30,600 | 49.2% |
| Scale value 8 to 10 | Scale value 8 to 10 | 10,102 | 16.2% |
| Missing | Scale value 4 to 7 | 122 | 0.2% |
| Household size |  |  |  |
| Single-person household | Single-person household | 13,696 | 22.0% |
| Multi-person household | Multi-person household | 48,314 | 77.7% |
| Missing | Multi-person household | 159 | 0.3% |
| Normal weight |  |  |  |
| Normal weight (18.5 <= BMI < 25) | Normal weight (18.5 <= BMI < 25) | 26,622 | 42.8% |
| Not normal weight (BMI < 18.5 or BMI >= 25) | Not normal weight (BMI < 18.5 or BMI >= 25) | 35,106 | 56.5% |
| Missing | Not normal weight (BMI < 18.5 or BMI >= 25) | 441 | 0.7% |
| Obesity |  |  |  |
| Yes | Yes | 12,378 | 19.9% |
| No | No | 49,350 | 79.4% |
| Missing | No | 441 | 0.7% |
| Overweight |  |  |  |
| Yes | Yes | 33,701 | 54.2% |
| No | No | 28,027 | 45.1% |
| Missing | Yes | 441 | 0.7% |
| Paying attention to health |  |  |  |
| Not at all/less strong/moderate | Not at all/less strong/moderate | 31,088 | 50.0% |
| Strong/very strong | Strong/very strong | 31,034 | 49.9% |
| Missing | Not at all/less strong/moderate | 47 | 0.1% |
| Red meat |  |  |  |
| Daily or several times a day | Daily or several times a day | 1,461 | 2.4% |
| 4 to 6 times per week | 4 to 6 times per week | 7,029 | 11.3% |
| 1 to 3 times per week | 1 to 3 times per week | 28,261 | 45.5% |
| Less than once per week | Less than once per week | 19,083 | 30.7% |
| Never | Never | 6,277 | 10.1% |
| Missing | 1 to 3 times per week | 58 | 0.1% |
| Region |  |  |  |
| Northeast | Northeast | 10,214 | 16.4% |
| Northwest | Northwest | 14,846 | 23.9% |
| Central-East | Central-East | 6,355 | 10.2% |
| Central-West | Central-West | 17,376 | 27.9% |
| South | South | 13,378 | 21.5% |
| Satisfaction: Life in general |  |  |  |
| Scale value 1 to 3 | Scale value 1 to 3 | 4,779 | 7.7% |
| Scale value 4 to 7 | Scale value 4 to 7 | 27,440 | 44.1% |
| Scale value 8 to 10 | Scale value 8 to 10 | 29,852 | 48.0% |
| Missing | Scale value 8 to 10 | 98 | 0.2% |
| Sausage products |  |  |  |
| Daily or several times a day | Daily or several times a day | 5,965 | 9.6% |
| 4 to 6 times per week | 4 to 6 times per week | 11,711 | 18.8% |
| 1 to 3 times per week | 1 to 3 times per week | 21,615 | 34.8% |
| Less than once per week | Less than once per week | 15,766 | 25.4% |
| Never | Never | 7,055 | 11.3% |
| Missing | 1 to 3 times per week | 57 | 0.1% |
| Self-rated health |  |  |  |
| Very good/good/fair | Very good/good/fair | 58,181 | 93.6% |
| Bad/very bad | Bad/very bad | 3,918 | 6.3% |
| Missing | Very good/good/fair | 70 | 0.1% |
| Self-rated mental health |  |  |  |
| Excellent/very good/good | Excellent/very good/good | 49,345 | 79.4% |
| fair/poor | fair/poor | 12,730 | 20.5% |
| Missing | Excellent/very good/good | 94 | 0.2% |
| Sex |  |  |  |
| Male | Male | 29,206 | 47.0% |
| Female | Female | 32,963 | 53.0% |
| Smoking |  |  |  |
| Daily smoking | Daily smoking | 9,212 | 14.8% |
| Occasional smoking | Occasional smoking | 3,705 | 6.0% |
| Non-smoker | Non-smoker | 49,158 | 79.1% |
| Missing | Non-smoker | 94 | 0.2% |
| Sport |  |  |  |
| No sporting activities | No sporting activities | 12,842 | 20.7% |
| Less than 1 hour per week | Less than 1 hour per week | 11,737 | 18.9% |
| 1 to less than 2 hours per week | 1 to less than 2 hours per week | 16,949 | 27.3% |
| 2 to less than 4 hours per week | 2 to less than 4 hours per week | 12,602 | 20.3% |
| 4 hours per week and more | 4 hours per week and more | 7,877 | 12.7% |
| Missing | 1 to less than 2 hours per week | 162 | 0.3% |
| Survey Mode |  |  |  |
| Computer Assisted Web Interview | Computer Assisted Web Interview | 46,207 | 74.3% |
| Paper and Pencil Interview | Paper and Pencil Interview | 15,962 | 25.7% |
| Waited for appointment |  |  |  |
| Yes | Yes | 20,321 | 32.7% |
| No | No | 34,460 | 55.4% |
| No need for examination or treatment | No need for examination or treatment | 7,221 | 11.6% |
| Missing | No | 167 | 0.3% |
|  |  |  |  |
| --- | --- | --- | --- |
| 1Continuous variables were replaced with their respective median, while categorical variables were replaced with the mode. | | | |

### Missing values by participation status

| Proportion of Missing Values by Panel-registration and Panel wave 2024 participation (Questionaire(Q) A to D) | | | | | | | | | | |
| --- | --- | --- | --- | --- | --- | --- | --- | --- | --- | --- |
| Characteristic | Not registered % (n.Obs) | Registered % (n.Obs) | Non-Participant Q-A % (n.Obs) | Participant Q-A % (n.Obs) | Non-Participant Q-B % (n.Obs) | Participant Q-B % (n.Obs) | Non-Participant Q-C % (n.Obs) | Participant Q-C % (n.Obs) | Non-Participant Q-D % (n.Obs) | Participant Q-D % (n.Obs) |
| Education: Casmin | 0.1% (70) | 0.2% (106) | 0.1% (33) | 0.2% (58) | 0.1% (33) | 0.1% (40) | 0.1% (31) | 0.1% (53) | 0.1% (23) | 0.1% (49) |
| Education: ISCED (2011) | 0.1% (70) | 0.2% (106) | 0.1% (33) | 0.2% (58) | 0.1% (33) | 0.1% (40) | 0.1% (31) | 0.1% (53) | 0.1% (23) | 0.1% (49) |
| Current life situation | 0.2% (119) | 0.3% (179) | 0.1% (46) | 0.3% (92) | 0.1% (44) | 0.2% (82) | 0.1% (51) | 0.2% (76) | 0.1% (49) | 0.3% (94) |
| Normal weight | 0.3% (166) | 0.4% (275) | 0.1% (40) | 0.4% (150) | 0.1% (52) | 0.5% (164) | 0.2% (54) | 0.4% (149) | 0.1% (40) | 0.5% (177) |
| Obesity | 0.3% (166) | 0.4% (275) | 0.1% (40) | 0.4% (150) | 0.1% (52) | 0.5% (164) | 0.2% (54) | 0.4% (149) | 0.1% (40) | 0.5% (177) |
| Overweight | 0.3% (166) | 0.4% (275) | 0.1% (40) | 0.4% (150) | 0.1% (52) | 0.5% (164) | 0.2% (54) | 0.4% (149) | 0.1% (40) | 0.5% (177) |
| Self-rated health | 0.0% (29) | 0.1% (41) | 0.0% (2) | 0.1% (29) | 0.0% (9) | 0.1% (23) | 0.0% (6) | 0.1% (27) | 0.0% (10) | 0.1% (19) |
| Chronic diseases | 0.1% (47) | 0.2% (100) | 0.0% (10) | 0.1% (53) | 0.1% (21) | 0.2% (66) | 0.1% (26) | 0.1% (49) | 0.0% (12) | 0.2% (65) |
| Satisfaction: Life in general | 0.1% (35) | 0.1% (63) | 0.0% (10) | 0.1% (42) | 0.0% (6) | 0.1% (37) | 0.0% (9) | 0.1% (29) | 0.0% (9) | 0.1% (41) |
| Health risk due to climate change | 0.1% (46) | 0.1% (76) | 0.0% (10) | 0.1% (40) | 0.1% (19) | 0.1% (41) | 0.1% (20) | 0.1% (39) | 0.0% (14) | 0.1% (43) |
| Paying attention to health | 0.0% (22) | 0.0% (25) | 0.0% (5) | 0.0% (13) | 0.0% (4) | 0.0% (15) | 0.0% (5) | 0.0% (17) | 0.0% (6) | 0.0% (12) |
| Self-rated mental health | 0.1% (36) | 0.1% (58) | 0.0% (8) | 0.1% (44) | 0.0% (3) | 0.1% (32) | 0.0% (10) | 0.1% (42) | 0.0% (5) | 0.1% (32) |
| Smoking | 0.1% (35) | 0.1% (59) | 0.0% (11) | 0.1% (36) | 0.0% (4) | 0.1% (41) | 0.0% (13) | 0.1% (33) | 0.0% (8) | 0.1% (33) |
| Sausage products | 0.0% (21) | 0.1% (36) | 0.0% (10) | 0.1% (26) | 0.0% (1) | 0.1% (20) | 0.0% (14) | 0.1% (18) | 0.0% (7) | 0.0% (14) |
| Red meat | 0.0% (22) | 0.1% (36) | 0.0% (7) | 0.1% (30) | 0.0% (1) | 0.1% (22) | 0.0% (9) | 0.1% (23) | 0.0% (2) | 0.0% (16) |
| Sport | 0.1% (66) | 0.2% (96) | 0.1% (19) | 0.2% (60) | 0.0% (9) | 0.2% (58) | 0.1% (28) | 0.1% (47) | 0.0% (16) | 0.1% (52) |
| Waited for appointment | 0.1% (62) | 0.2% (105) | 0.0% (17) | 0.2% (61) | 0.1% (24) | 0.1% (53) | 0.0% (17) | 0.2% (54) | 0.1% (22) | 0.2% (67) |
| Country of birth | 0.1% (64) | 0.2% (97) | 0.1% (18) | 0.2% (55) | 0.1% (31) | 0.1% (43) | 0.1% (20) | 0.1% (52) | 0.1% (22) | 0.1% (47) |
| German Citizenship | 0.3% (185) | 0.1% (47) | 0.0% (10) | 0.1% (37) | 0.0% (5) | 0.1% (23) | 0.0% (5) | 0.1% (28) | 0.0% (8) | 0.1% (22) |
| Household size | 0.1% (63) | 0.2% (96) | 0.1% (23) | 0.1% (43) | 0.1% (24) | 0.1% (53) | 0.1% (25) | 0.2% (54) | 0.1% (19) | 0.1% (46) |

## Result model estimation: Registration

| Characteristic | log(OR) | SE | 95% CI | p-value | p-value (global) |
| --- | --- | --- | --- | --- | --- |
| Sex |  |  |  |  | 0.035 |
| Male | — | — | — |  |  |
| Female | 0.38 | 0.180 |  |  |  |
| Age |  |  |  |  | 0.027 |
| 16-29 yrs | — | — | — |  |  |
| 30-39 yrs | 0.20 | 0.486 |  |  |  |
| 40-49 yrs | 0.00 | 0.470 |  |  |  |
| 50-59 yrs | -0.62 | 0.451 |  |  |  |
| 60-69 yrs | -1.2 | 0.436 |  |  |  |
| 70-79 yrs | -0.98 | 0.513 |  |  |  |
| 80+ | -0.26 | 0.579 |  |  |  |
| Education: CASMIN |  |  |  |  | 0.423 |
| Low | — | — | — |  |  |
| Medium | 0.37 | 0.332 |  |  |  |
| High | -0.01 | 0.427 |  |  |  |
| Self-rated health |  |  |  |  | 0.161 |
| Very good/good/fair | — | — | — |  |  |
| Bad/very bad | -0.35 | 0.253 |  |  |  |
| Chronic diseases |  |  |  |  | 0.004 |
| Yes | — | — | — |  |  |
| No | -0.22 | 0.077 |  |  |  |
| Satisfaction: Life in general |  |  |  |  | >0.9 |
| Scale value 1 to 3 | — | — | — |  |  |
| Scale value 4 to 7 | -0.07 | 0.160 |  |  |  |
| Scale value 8 to 10 | -0.07 | 0.172 |  |  |  |
| Paying attention to health |  |  |  |  | 0.057 |
| Not at all/less strong/moderate | — | — | — |  |  |
| Strong/very strong | 0.09 | 0.047 |  |  |  |
| Smoking |  |  |  |  | 0.738 |
| Daily smoking | — | — | — |  |  |
| Occasional smoking | 0.13 | 0.176 |  |  |  |
| Non-smoker | 0.02 | 0.112 |  |  |  |
| Sport |  |  |  |  | 0.359 |
| No sporting activities | — | — | — |  |  |
| Less than 1 hour per week | 0.07 | 0.130 |  |  |  |
| 1 to less than 2 hours per week | -0.01 | 0.112 |  |  |  |
| 2 to less than 4 hours per week | 0.15 | 0.119 |  |  |  |
| 4 hours per week and more | 0.14 | 0.120 |  |  |  |
| Interview type |  |  |  |  | 0.1 |
| Computer Assisted Web Interview | — | — | — |  |  |
| Paper and Pencil Interview | -0.22 | 0.134 |  |  |  |
| Regions |  |  |  |  | 0.759 |
| Northeast | — | — | — |  |  |
| Northwest | 0.04 | 0.146 |  |  |  |
| Central-East | 0.04 | 0.161 |  |  |  |
| Central-West | 0.16 | 0.142 |  |  |  |
| South | 0.04 | 0.148 |  |  |  |
| Country of birth |  |  |  |  | 0.051 |
| In Germany (within current borders) | — | — | — |  |  |
| In another country | -0.35 | 0.181 |  |  |  |
| German nationality |  |  |  |  | 0.184 |
| Yes | — | — | — |  |  |
| No | -0.31 | 0.237 |  |  |  |
| Household size |  |  |  |  | 0.037 |
| Single-person household | — | — | — |  |  |
| Multi-person household | 0.06 | 0.029 |  |  |  |
| Education: ISCED (2011) |  |  |  |  | 0.032 |
| ISCED 1 | — | — | — |  |  |
| ISCED 2 | 0.26 | 0.195 |  |  |  |
| ISCED 3 | 0.10 | 0.177 |  |  |  |
| ISCED 4 | 0.36 | 0.190 |  |  |  |
| ISCED 6 | 0.21 | 0.197 |  |  |  |
| ISCED 7 | 0.49 | 0.246 |  |  |  |
| ISCED 8 | -0.32 | 0.546 |  |  |  |
| Current living situation |  |  |  |  | 0.336 |
| Full-time employed | — | — | — |  |  |
| Part-time employed | 0.09 | 0.150 |  |  |  |
| Unemployed | 0.29 | 0.200 |  |  |  |
| Retired or early retired | -0.42 | 0.728 |  |  |  |
| Not employed for other reasons (student, volunteer service, homemaker) | 0.26 | 0.142 |  |  |  |
| Obesity |  |  |  |  | 0.742 |
| Yes | — | — | — |  |  |
| No | -0.04 | 0.136 |  |  |  |
| Overweight |  |  |  |  | 0.404 |
| Yes | — | — | — |  |  |
| No | -0.09 | 0.103 |  |  |  |
| Health risk due to climate change |  |  |  |  | 0.354 |
| Scale value 1 to 3 | — | — | — |  |  |
| Scale value 4 to 7 | 0.14 | 0.098 |  |  |  |
| Scale value 8 to 10 | 0.05 | 0.128 |  |  |  |
| Self-rated mental health |  |  |  |  | 0.143 |
| Excellent/very good/good | — | — | — |  |  |
| fair/poor | 0.17 | 0.117 |  |  |  |
| Sausage products |  |  |  |  | >0.9 |
| Daily or several times a day | — | — | — |  |  |
| 4 to 6 times per week | -0.04 | 0.159 |  |  |  |
| 1 to 3 times per week | 0.05 | 0.155 |  |  |  |
| Less than once per week | 0.01 | 0.166 |  |  |  |
| Never | 0.05 | 0.235 |  |  |  |
| Red meat |  |  |  |  | 0.302 |
| Daily or several times a day | — | — | — |  |  |
| 4 to 6 times per week | 0.21 | 0.234 |  |  |  |
| 1 to 3 times per week | -0.02 | 0.218 |  |  |  |
| Less than once per week | -0.06 | 0.218 |  |  |  |
| Never | 0.17 | 0.272 |  |  |  |
| Waited for medical examination date in the last 12 months |  |  |  |  | 0.65 |
| Yes | — | — | — |  |  |
| No | 0.05 | 0.086 |  |  |  |
| No need for examination or treatment | -0.05 | 0.124 |  |  |  |
| BIK community size (categorized) |  |  |  |  | 0.703 |
| BIK region <20,000 inhabitants | — | — | — |  |  |
| BIK region 20,000 to <50,000 inhabitants OR surroundings 50,000 to <500,000 inhabitants | -0.02 | 0.147 |  |  |  |
| Core city 50,000 to <500,000 inhabitants OR surroundings 500,000+ inhabitants | -0.04 | 0.163 |  |  |  |
| Core city 500,000+ inhabitants | 0.10 | 0.160 |  |  |  |
| Foreign nationality |  |  |  |  | 0.822 |
| Foreign national | — | — | — |  |  |
| Not foreign national | 0.02 | 0.086 |  |  |  |
| Underweight |  |  |  |  | 0.472 |
| Underweight (BMI < 18.5) | — | — | — |  |  |
| No Underweight (BMI >= 18.5) | 0.11 | 0.155 |  |  |  |
| Age \* Obesity |  |  |  |  | 0.45 |
| 30-39 yrs \* No | -0.14 | 0.151 |  |  |  |
| 40-49 yrs \* No | 0.12 | 0.152 |  |  |  |
| 50-59 yrs \* No | -0.04 | 0.147 |  |  |  |
| 60-69 yrs \* No | -0.02 | 0.149 |  |  |  |
| 70-79 yrs \* No | -0.07 | 0.148 |  |  |  |
| 80+ \* No | -0.17 | 0.160 |  |  |  |
| Age \* Overweight |  |  |  |  | 0.577 |
| 30-39 yrs \* No | -0.04 | 0.118 |  |  |  |
| 40-49 yrs \* No | -0.06 | 0.124 |  |  |  |
| 50-59 yrs \* No | 0.07 | 0.110 |  |  |  |
| 60-69 yrs \* No | 0.09 | 0.114 |  |  |  |
| 70-79 yrs \* No | -0.02 | 0.110 |  |  |  |
| 80+ \* No | -0.10 | 0.120 |  |  |  |
| Age \* Self-rated health |  |  |  |  | 0.499 |
| 30-39 yrs \* Bad/very bad | 0.10 | 0.318 |  |  |  |
| 40-49 yrs \* Bad/very bad | 0.17 | 0.330 |  |  |  |
| 50-59 yrs \* Bad/very bad | 0.16 | 0.276 |  |  |  |
| 60-69 yrs \* Bad/very bad | 0.44 | 0.278 |  |  |  |
| 70-79 yrs \* Bad/very bad | 0.30 | 0.276 |  |  |  |
| 80+ \* Bad/very bad | 0.15 | 0.283 |  |  |  |
| Age \* Chronic diseases |  |  |  |  | 0.379 |
| 30-39 yrs \* No | 0.17 | 0.104 |  |  |  |
| 40-49 yrs \* No | 0.03 | 0.113 |  |  |  |
| 50-59 yrs \* No | -0.06 | 0.091 |  |  |  |
| 60-69 yrs \* No | 0.02 | 0.093 |  |  |  |
| 70-79 yrs \* No | -0.01 | 0.104 |  |  |  |
| 80+ \* No | 0.08 | 0.109 |  |  |  |
| Age \* Satisfaction: Life in general |  |  |  |  | 0.014 |
| 30-39 yrs \* Scale value 4 to 7 | -0.09 | 0.184 |  |  |  |
| 40-49 yrs \* Scale value 4 to 7 | -0.12 | 0.206 |  |  |  |
| 50-59 yrs \* Scale value 4 to 7 | -0.04 | 0.177 |  |  |  |
| 60-69 yrs \* Scale value 4 to 7 | 0.12 | 0.182 |  |  |  |
| 70-79 yrs \* Scale value 4 to 7 | -0.08 | 0.201 |  |  |  |
| 80+ \* Scale value 4 to 7 | 0.33 | 0.189 |  |  |  |
| 30-39 yrs \* Scale value 8 to 10 | -0.07 | 0.198 |  |  |  |
| 40-49 yrs \* Scale value 8 to 10 | -0.05 | 0.213 |  |  |  |
| 50-59 yrs \* Scale value 8 to 10 | 0.01 | 0.181 |  |  |  |
| 60-69 yrs \* Scale value 8 to 10 | 0.26 | 0.181 |  |  |  |
| 70-79 yrs \* Scale value 8 to 10 | 0.25 | 0.217 |  |  |  |
| 80+ \* Scale value 8 to 10 | 0.45 | 0.207 |  |  |  |
| Age \* Health risk due to climate change |  |  |  |  | 0.188 |
| 30-39 yrs \* Scale value 4 to 7 | 0.09 | 0.110 |  |  |  |
| 40-49 yrs \* Scale value 4 to 7 | 0.01 | 0.109 |  |  |  |
| 50-59 yrs \* Scale value 4 to 7 | -0.04 | 0.105 |  |  |  |
| 60-69 yrs \* Scale value 4 to 7 | -0.15 | 0.108 |  |  |  |
| 70-79 yrs \* Scale value 4 to 7 | -0.06 | 0.116 |  |  |  |
| 80+ \* Scale value 4 to 7 | -0.05 | 0.111 |  |  |  |
| 30-39 yrs \* Scale value 8 to 10 | -0.17 | 0.142 |  |  |  |
| 40-49 yrs \* Scale value 8 to 10 | -0.24 | 0.146 |  |  |  |
| 50-59 yrs \* Scale value 8 to 10 | 0.02 | 0.140 |  |  |  |
| 60-69 yrs \* Scale value 8 to 10 | -0.14 | 0.136 |  |  |  |
| 70-79 yrs \* Scale value 8 to 10 | -0.05 | 0.154 |  |  |  |
| 80+ \* Scale value 8 to 10 | -0.01 | 0.160 |  |  |  |
| Age \* Self-rated mental health |  |  |  |  | 0.151 |
| 30-39 yrs \* fair/poor | 0.06 | 0.137 |  |  |  |
| 40-49 yrs \* fair/poor | -0.10 | 0.153 |  |  |  |
| 50-59 yrs \* fair/poor | -0.19 | 0.129 |  |  |  |
| 60-69 yrs \* fair/poor | -0.05 | 0.130 |  |  |  |
| 70-79 yrs \* fair/poor | -0.27 | 0.136 |  |  |  |
| 80+ \* fair/poor | -0.23 | 0.143 |  |  |  |
| Age \* Smoking |  |  |  |  | 0.009 |
| 30-39 yrs \* Occasional smoking | -0.29 | 0.207 |  |  |  |
| 40-49 yrs \* Occasional smoking | -0.13 | 0.194 |  |  |  |
| 50-59 yrs \* Occasional smoking | -0.02 | 0.190 |  |  |  |
| 60-69 yrs \* Occasional smoking | -0.44 | 0.194 |  |  |  |
| 70-79 yrs \* Occasional smoking | -0.54 | 0.276 |  |  |  |
| 80+ \* Occasional smoking | -0.46 | 0.402 |  |  |  |
| 30-39 yrs \* Non-smoker | -0.21 | 0.138 |  |  |  |
| 40-49 yrs \* Non-smoker | -0.11 | 0.122 |  |  |  |
| 50-59 yrs \* Non-smoker | 0.09 | 0.113 |  |  |  |
| 60-69 yrs \* Non-smoker | -0.03 | 0.125 |  |  |  |
| 70-79 yrs \* Non-smoker | -0.26 | 0.142 |  |  |  |
| 80+ \* Non-smoker | -0.61 | 0.224 |  |  |  |
| Age \* Sausage products |  |  |  |  | >0.9 |
| 30-39 yrs \* 4 to 6 times per week | 0.05 | 0.180 |  |  |  |
| 40-49 yrs \* 4 to 6 times per week | 0.10 | 0.196 |  |  |  |
| 50-59 yrs \* 4 to 6 times per week | 0.16 | 0.168 |  |  |  |
| 60-69 yrs \* 4 to 6 times per week | 0.24 | 0.174 |  |  |  |
| 70-79 yrs \* 4 to 6 times per week | 0.02 | 0.186 |  |  |  |
| 80+ \* 4 to 6 times per week | 0.01 | 0.195 |  |  |  |
| 30-39 yrs \* 1 to 3 times per week | 0.02 | 0.176 |  |  |  |
| 40-49 yrs \* 1 to 3 times per week | 0.07 | 0.178 |  |  |  |
| 50-59 yrs \* 1 to 3 times per week | -0.02 | 0.162 |  |  |  |
| 60-69 yrs \* 1 to 3 times per week | -0.04 | 0.167 |  |  |  |
| 70-79 yrs \* 1 to 3 times per week | -0.07 | 0.170 |  |  |  |
| 80+ \* 1 to 3 times per week | -0.08 | 0.181 |  |  |  |
| 30-39 yrs \* Less than once per week | -0.11 | 0.183 |  |  |  |
| 40-49 yrs \* Less than once per week | -0.05 | 0.198 |  |  |  |
| 50-59 yrs \* Less than once per week | -0.06 | 0.167 |  |  |  |
| 60-69 yrs \* Less than once per week | -0.06 | 0.181 |  |  |  |
| 70-79 yrs \* Less than once per week | -0.04 | 0.188 |  |  |  |
| 80+ \* Less than once per week | -0.12 | 0.203 |  |  |  |
| 30-39 yrs \* Never | -0.01 | 0.235 |  |  |  |
| 40-49 yrs \* Never | -0.05 | 0.239 |  |  |  |
| 50-59 yrs \* Never | 0.11 | 0.232 |  |  |  |
| 60-69 yrs \* Never | 0.00 | 0.236 |  |  |  |
| 70-79 yrs \* Never | 0.20 | 0.257 |  |  |  |
| 80+ \* Never | 0.15 | 0.283 |  |  |  |
| Age \* Red meat |  |  |  |  | 0.094 |
| 30-39 yrs \* 4 to 6 times per week | -0.27 | 0.285 |  |  |  |
| 40-49 yrs \* 4 to 6 times per week | 0.13 | 0.315 |  |  |  |
| 50-59 yrs \* 4 to 6 times per week | -0.10 | 0.263 |  |  |  |
| 60-69 yrs \* 4 to 6 times per week | -0.14 | 0.296 |  |  |  |
| 70-79 yrs \* 4 to 6 times per week | 0.13 | 0.373 |  |  |  |
| 80+ \* 4 to 6 times per week | -0.78 | 0.398 |  |  |  |
| 30-39 yrs \* 1 to 3 times per week | -0.24 | 0.271 |  |  |  |
| 40-49 yrs \* 1 to 3 times per week | 0.05 | 0.290 |  |  |  |
| 50-59 yrs \* 1 to 3 times per week | 0.13 | 0.246 |  |  |  |
| 60-69 yrs \* 1 to 3 times per week | 0.19 | 0.273 |  |  |  |
| 70-79 yrs \* 1 to 3 times per week | 0.37 | 0.348 |  |  |  |
| 80+ \* 1 to 3 times per week | -0.48 | 0.367 |  |  |  |
| 30-39 yrs \* Less than once per week | -0.18 | 0.273 |  |  |  |
| 40-49 yrs \* Less than once per week | -0.02 | 0.295 |  |  |  |
| 50-59 yrs \* Less than once per week | 0.03 | 0.252 |  |  |  |
| 60-69 yrs \* Less than once per week | 0.18 | 0.276 |  |  |  |
| 70-79 yrs \* Less than once per week | 0.39 | 0.353 |  |  |  |
| 80+ \* Less than once per week | -0.43 | 0.374 |  |  |  |
| 30-39 yrs \* Never | -0.21 | 0.319 |  |  |  |
| 40-49 yrs \* Never | -0.21 | 0.325 |  |  |  |
| 50-59 yrs \* Never | -0.20 | 0.308 |  |  |  |
| 60-69 yrs \* Never | 0.03 | 0.328 |  |  |  |
| 70-79 yrs \* Never | -0.06 | 0.392 |  |  |  |
| 80+ \* Never | -0.61 | 0.396 |  |  |  |
| Age \* Sport |  |  |  |  | 0.125 |
| 30-39 yrs \* Less than 1 hour per week | -0.03 | 0.160 |  |  |  |
| 40-49 yrs \* Less than 1 hour per week | 0.07 | 0.152 |  |  |  |
| 50-59 yrs \* Less than 1 hour per week | -0.26 | 0.155 |  |  |  |
| 60-69 yrs \* Less than 1 hour per week | -0.03 | 0.150 |  |  |  |
| 70-79 yrs \* Less than 1 hour per week | -0.12 | 0.161 |  |  |  |
| 80+ \* Less than 1 hour per week | 0.03 | 0.150 |  |  |  |
| 30-39 yrs \* 1 to less than 2 hours per week | -0.03 | 0.150 |  |  |  |
| 40-49 yrs \* 1 to less than 2 hours per week | 0.14 | 0.152 |  |  |  |
| 50-59 yrs \* 1 to less than 2 hours per week | -0.05 | 0.138 |  |  |  |
| 60-69 yrs \* 1 to less than 2 hours per week | 0.13 | 0.135 |  |  |  |
| 70-79 yrs \* 1 to less than 2 hours per week | 0.17 | 0.135 |  |  |  |
| 80+ \* 1 to less than 2 hours per week | 0.19 | 0.146 |  |  |  |
| 30-39 yrs \* 2 to less than 4 hours per week | -0.28 | 0.150 |  |  |  |
| 40-49 yrs \* 2 to less than 4 hours per week | -0.14 | 0.157 |  |  |  |
| 50-59 yrs \* 2 to less than 4 hours per week | -0.13 | 0.150 |  |  |  |
| 60-69 yrs \* 2 to less than 4 hours per week | -0.05 | 0.151 |  |  |  |
| 70-79 yrs \* 2 to less than 4 hours per week | 0.10 | 0.161 |  |  |  |
| 80+ \* 2 to less than 4 hours per week | 0.04 | 0.158 |  |  |  |
| 30-39 yrs \* 4 hours per week and more | -0.04 | 0.166 |  |  |  |
| 40-49 yrs \* 4 hours per week and more | -0.02 | 0.190 |  |  |  |
| 50-59 yrs \* 4 hours per week and more | -0.12 | 0.164 |  |  |  |
| 60-69 yrs \* 4 hours per week and more | 0.11 | 0.161 |  |  |  |
| 70-79 yrs \* 4 hours per week and more | 0.24 | 0.167 |  |  |  |
| 80+ \* 4 hours per week and more | 0.38 | 0.202 |  |  |  |
| Age \* Waited for medical examination date in the last 12 months |  |  |  |  | 0.431 |
| 30-39 yrs \* No | 0.02 | 0.107 |  |  |  |
| 40-49 yrs \* No | -0.21 | 0.113 |  |  |  |
| 50-59 yrs \* No | -0.06 | 0.096 |  |  |  |
| 60-69 yrs \* No | -0.11 | 0.092 |  |  |  |
| 70-79 yrs \* No | -0.06 | 0.107 |  |  |  |
| 80+ \* No | -0.25 | 0.119 |  |  |  |
| 30-39 yrs \* No need for examination or treatment | -0.03 | 0.129 |  |  |  |
| 40-49 yrs \* No need for examination or treatment | 0.02 | 0.137 |  |  |  |
| 50-59 yrs \* No need for examination or treatment | -0.12 | 0.144 |  |  |  |
| 60-69 yrs \* No need for examination or treatment | -0.03 | 0.151 |  |  |  |
| 70-79 yrs \* No need for examination or treatment | -0.04 | 0.172 |  |  |  |
| 80+ \* No need for examination or treatment | -0.14 | 0.245 |  |  |  |
| Age \* Interview type |  |  |  |  | 0.416 |
| 30-39 yrs \* Paper and Pencil Interview | 0.10 | 0.183 |  |  |  |
| 40-49 yrs \* Paper and Pencil Interview | 0.10 | 0.165 |  |  |  |
| 50-59 yrs \* Paper and Pencil Interview | 0.22 | 0.139 |  |  |  |
| 60-69 yrs \* Paper and Pencil Interview | 0.12 | 0.145 |  |  |  |
| 70-79 yrs \* Paper and Pencil Interview | 0.21 | 0.144 |  |  |  |
| 80+ \* Paper and Pencil Interview | 0.29 | 0.159 |  |  |  |
| Age \* BIK community size (categorized) |  |  |  |  | 0.444 |
| 30-39 yrs \* BIK region 20,000 to <50,000 inhabitants OR surroundings 50,000 to <500,000 inhabitants | 0.01 | 0.153 |  |  |  |
| 40-49 yrs \* BIK region 20,000 to <50,000 inhabitants OR surroundings 50,000 to <500,000 inhabitants | 0.16 | 0.159 |  |  |  |
| 50-59 yrs \* BIK region 20,000 to <50,000 inhabitants OR surroundings 50,000 to <500,000 inhabitants | -0.11 | 0.142 |  |  |  |
| 60-69 yrs \* BIK region 20,000 to <50,000 inhabitants OR surroundings 50,000 to <500,000 inhabitants | 0.11 | 0.137 |  |  |  |
| 70-79 yrs \* BIK region 20,000 to <50,000 inhabitants OR surroundings 50,000 to <500,000 inhabitants | 0.19 | 0.167 |  |  |  |
| 80+ \* BIK region 20,000 to <50,000 inhabitants OR surroundings 50,000 to <500,000 inhabitants | 0.04 | 0.150 |  |  |  |
| 30-39 yrs \* Core city 50,000 to <500,000 inhabitants OR surroundings 500,000+ inhabitants | 0.05 | 0.164 |  |  |  |
| 40-49 yrs \* Core city 50,000 to <500,000 inhabitants OR surroundings 500,000+ inhabitants | 0.37 | 0.186 |  |  |  |
| 50-59 yrs \* Core city 50,000 to <500,000 inhabitants OR surroundings 500,000+ inhabitants | 0.17 | 0.161 |  |  |  |
| 60-69 yrs \* Core city 50,000 to <500,000 inhabitants OR surroundings 500,000+ inhabitants | 0.20 | 0.144 |  |  |  |
| 70-79 yrs \* Core city 50,000 to <500,000 inhabitants OR surroundings 500,000+ inhabitants | 0.14 | 0.175 |  |  |  |
| 80+ \* Core city 50,000 to <500,000 inhabitants OR surroundings 500,000+ inhabitants | 0.22 | 0.168 |  |  |  |
| 30-39 yrs \* Core city 500,000+ inhabitants | -0.05 | 0.164 |  |  |  |
| 40-49 yrs \* Core city 500,000+ inhabitants | 0.18 | 0.172 |  |  |  |
| 50-59 yrs \* Core city 500,000+ inhabitants | -0.13 | 0.157 |  |  |  |
| 60-69 yrs \* Core city 500,000+ inhabitants | 0.12 | 0.152 |  |  |  |
| 70-79 yrs \* Core city 500,000+ inhabitants | 0.14 | 0.184 |  |  |  |
| 80+ \* Core city 500,000+ inhabitants | 0.08 | 0.163 |  |  |  |
| Age \* Regions |  |  |  |  | 0.065 |
| 30-39 yrs \* Northwest | 0.02 | 0.142 |  |  |  |
| 40-49 yrs \* Northwest | -0.10 | 0.159 |  |  |  |
| 50-59 yrs \* Northwest | 0.10 | 0.147 |  |  |  |
| 60-69 yrs \* Northwest | 0.21 | 0.136 |  |  |  |
| 70-79 yrs \* Northwest | 0.07 | 0.159 |  |  |  |
| 80+ \* Northwest | -0.06 | 0.171 |  |  |  |
| 30-39 yrs \* Central-East | 0.01 | 0.173 |  |  |  |
| 40-49 yrs \* Central-East | -0.10 | 0.180 |  |  |  |
| 50-59 yrs \* Central-East | -0.23 | 0.203 |  |  |  |
| 60-69 yrs \* Central-East | -0.20 | 0.164 |  |  |  |
| 70-79 yrs \* Central-East | -0.24 | 0.179 |  |  |  |
| 80+ \* Central-East | -0.27 | 0.202 |  |  |  |
| 30-39 yrs \* Central-West | 0.08 | 0.148 |  |  |  |
| 40-49 yrs \* Central-West | 0.08 | 0.146 |  |  |  |
| 50-59 yrs \* Central-West | -0.08 | 0.135 |  |  |  |
| 60-69 yrs \* Central-West | 0.19 | 0.136 |  |  |  |
| 70-79 yrs \* Central-West | 0.01 | 0.154 |  |  |  |
| 80+ \* Central-West | -0.01 | 0.166 |  |  |  |
| 30-39 yrs \* South | 0.15 | 0.143 |  |  |  |
| 40-49 yrs \* South | 0.01 | 0.163 |  |  |  |
| 50-59 yrs \* South | 0.09 | 0.146 |  |  |  |
| 60-69 yrs \* South | 0.18 | 0.139 |  |  |  |
| 70-79 yrs \* South | 0.01 | 0.158 |  |  |  |
| 80+ \* South | -0.10 | 0.173 |  |  |  |
| Age \* Country of birth |  |  |  |  | 0.005 |
| 30-39 yrs \* In another country | 0.02 | 0.193 |  |  |  |
| 40-49 yrs \* In another country | -0.04 | 0.192 |  |  |  |
| 50-59 yrs \* In another country | 0.01 | 0.178 |  |  |  |
| 60-69 yrs \* In another country | -0.27 | 0.201 |  |  |  |
| 70-79 yrs \* In another country | 0.27 | 0.204 |  |  |  |
| 80+ \* In another country | 0.37 | 0.195 |  |  |  |
| Age \* German nationality |  |  |  |  | 0.091 |
| 30-39 yrs \* No | -0.16 | 0.243 |  |  |  |
| 40-49 yrs \* No | -0.14 | 0.224 |  |  |  |
| 50-59 yrs \* No | 0.10 | 0.225 |  |  |  |
| 60-69 yrs \* No | 0.44 | 0.301 |  |  |  |
| 70-79 yrs \* No | 0.07 | 0.295 |  |  |  |
| 80+ \* No | 0.54 | 0.369 |  |  |  |
| Age \* Education: ISCED (2011) |  |  |  |  | <0.001 |
| 30-39 yrs \* ISCED 2 | 0.43 | 0.318 |  |  |  |
| 40-49 yrs \* ISCED 2 | -0.27 | 0.321 |  |  |  |
| 50-59 yrs \* ISCED 2 | 0.37 | 0.318 |  |  |  |
| 60-69 yrs \* ISCED 2 | 0.52 | 0.262 |  |  |  |
| 70-79 yrs \* ISCED 2 | 0.40 | 0.260 |  |  |  |
| 80+ \* ISCED 2 | 0.14 | 0.239 |  |  |  |
| 30-39 yrs \* ISCED 3 | 0.49 | 0.278 |  |  |  |
| 40-49 yrs \* ISCED 3 | 0.06 | 0.289 |  |  |  |
| 50-59 yrs \* ISCED 3 | 0.77 | 0.289 |  |  |  |
| 60-69 yrs \* ISCED 3 | 0.67 | 0.259 |  |  |  |
| 70-79 yrs \* ISCED 3 | 0.47 | 0.236 |  |  |  |
| 80+ \* ISCED 3 | 0.46 | 0.235 |  |  |  |
| 30-39 yrs \* ISCED 4 | 0.26 | 0.292 |  |  |  |
| 40-49 yrs \* ISCED 4 | -0.02 | 0.301 |  |  |  |
| 50-59 yrs \* ISCED 4 | 0.69 | 0.295 |  |  |  |
| 60-69 yrs \* ISCED 4 | 0.69 | 0.271 |  |  |  |
| 70-79 yrs \* ISCED 4 | 0.53 | 0.281 |  |  |  |
| 80+ \* ISCED 4 | 0.66 | 0.299 |  |  |  |
| 30-39 yrs \* ISCED 6 | 0.39 | 0.283 |  |  |  |
| 40-49 yrs \* ISCED 6 | -0.06 | 0.308 |  |  |  |
| 50-59 yrs \* ISCED 6 | 0.69 | 0.299 |  |  |  |
| 60-69 yrs \* ISCED 6 | 0.74 | 0.271 |  |  |  |
| 70-79 yrs \* ISCED 6 | 0.65 | 0.260 |  |  |  |
| 80+ \* ISCED 6 | 0.59 | 0.247 |  |  |  |
| 30-39 yrs \* ISCED 7 | 0.14 | 0.311 |  |  |  |
| 40-49 yrs \* ISCED 7 | -0.08 | 0.329 |  |  |  |
| 50-59 yrs \* ISCED 7 | 0.47 | 0.328 |  |  |  |
| 60-69 yrs \* ISCED 7 | 0.53 | 0.312 |  |  |  |
| 70-79 yrs \* ISCED 7 | 0.36 | 0.301 |  |  |  |
| 80+ \* ISCED 7 | 0.35 | 0.306 |  |  |  |
| 30-39 yrs \* ISCED 8 | 1.3 | 0.610 |  |  |  |
| 40-49 yrs \* ISCED 8 | -0.09 | 0.598 |  |  |  |
| 50-59 yrs \* ISCED 8 | 1.2 | 0.584 |  |  |  |
| 60-69 yrs \* ISCED 8 | 1.5 | 0.615 |  |  |  |
| 70-79 yrs \* ISCED 8 | 0.93 | 0.572 |  |  |  |
| 80+ \* ISCED 8 | 0.87 | 0.587 |  |  |  |
| Age \* Current living situation |  |  |  |  | <0.001 |
| 30-39 yrs \* Part-time employed | 0.09 | 0.141 |  |  |  |
| 40-49 yrs \* Part-time employed | 0.04 | 0.145 |  |  |  |
| 50-59 yrs \* Part-time employed | 0.07 | 0.133 |  |  |  |
| 60-69 yrs \* Part-time employed | 0.16 | 0.147 |  |  |  |
| 70-79 yrs \* Part-time employed | 0.14 | 0.359 |  |  |  |
| 80+ \* Part-time employed | 0.55 | 0.598 |  |  |  |
| 30-39 yrs \* Unemployed | 0.25 | 0.232 |  |  |  |
| 40-49 yrs \* Unemployed | 0.07 | 0.240 |  |  |  |
| 50-59 yrs \* Unemployed | 0.30 | 0.224 |  |  |  |
| 60-69 yrs \* Unemployed | 0.18 | 0.229 |  |  |  |
| 70-79 yrs \* Unemployed | -3.2 | 1.17 |  |  |  |
| 80+ \* Unemployed | 9.6 | 0.763 |  |  |  |
| 30-39 yrs \* Retired or early retired | 0.70 | 0.906 |  |  |  |
| 40-49 yrs \* Retired or early retired | 1.4 | 0.847 |  |  |  |
| 50-59 yrs \* Retired or early retired | 0.66 | 0.746 |  |  |  |
| 60-69 yrs \* Retired or early retired | 0.57 | 0.737 |  |  |  |
| 70-79 yrs \* Retired or early retired | 0.63 | 0.741 |  |  |  |
| 80+ \* Retired or early retired | 0.87 | 0.756 |  |  |  |
| 30-39 yrs \* Not employed for other reasons (student, volunteer service, homemaker) | 0.18 | 0.164 |  |  |  |
| 40-49 yrs \* Not employed for other reasons (student, volunteer service, homemaker) | -0.16 | 0.207 |  |  |  |
| 50-59 yrs \* Not employed for other reasons (student, volunteer service, homemaker) | -0.30 | 0.152 |  |  |  |
| 60-69 yrs \* Not employed for other reasons (student, volunteer service, homemaker) | -0.31 | 0.167 |  |  |  |
| 70-79 yrs \* Not employed for other reasons (student, volunteer service, homemaker) | -0.34 | 0.362 |  |  |  |
| 80+ \* Not employed for other reasons (student, volunteer service, homemaker) | 0.34 | 0.321 |  |  |  |
| Sex \* Chronic diseases |  |  |  |  | 0.027 |
| Female \* No | -0.11 | 0.048 |  |  |  |
| Sex \* Sausage products |  |  |  |  | 0.284 |
| Female \* 4 to 6 times per week | 0.02 | 0.112 |  |  |  |
| Female \* 1 to 3 times per week | -0.09 | 0.103 |  |  |  |
| Female \* Less than once per week | 0.02 | 0.111 |  |  |  |
| Female \* Never | -0.14 | 0.130 |  |  |  |
| Sex \* Sport |  |  |  |  | 0.048 |
| Female \* Less than 1 hour per week | 0.14 | 0.082 |  |  |  |
| Female \* 1 to less than 2 hours per week | 0.15 | 0.076 |  |  |  |
| Female \* 2 to less than 4 hours per week | 0.26 | 0.084 |  |  |  |
| Female \* 4 hours per week and more | 0.14 | 0.091 |  |  |  |
| Sex \* Regions |  |  |  |  | 0.456 |
| Female \* Northwest | 0.02 | 0.077 |  |  |  |
| Female \* Central-East | 0.00 | 0.092 |  |  |  |
| Female \* Central-West | -0.10 | 0.074 |  |  |  |
| Female \* South | -0.02 | 0.076 |  |  |  |
| Sex \* Country of birth |  |  |  |  | 0.327 |
| Female \* In another country | -0.07 | 0.070 |  |  |  |
| Sex \* Education: ISCED (2011) |  |  |  |  | 0.105 |
| Female \* ISCED 2 | -0.36 | 0.151 |  |  |  |
| Female \* ISCED 3 | -0.23 | 0.136 |  |  |  |
| Female \* ISCED 4 | -0.27 | 0.148 |  |  |  |
| Female \* ISCED 6 | -0.34 | 0.142 |  |  |  |
| Female \* ISCED 7 | -0.32 | 0.156 |  |  |  |
| Female \* ISCED 8 | -0.08 | 0.225 |  |  |  |
| Sex \* Current living situation |  |  |  |  | 0.001 |
| Female \* Part-time employed | -0.17 | 0.097 |  |  |  |
| Female \* Unemployed | -0.08 | 0.146 |  |  |  |
| Female \* Retired or early retired | -0.25 | 0.064 |  |  |  |
| Female \* Not employed for other reasons (student, volunteer service, homemaker) | 0.00 | 0.107 |  |  |  |
| Education: CASMIN \* Obesity |  |  |  |  | 0.623 |
| Medium \* No | 0.04 | 0.079 |  |  |  |
| High \* No | -0.05 | 0.107 |  |  |  |
| Education: CASMIN \* Overweight |  |  |  |  | 0.328 |
| Medium \* No | 0.08 | 0.070 |  |  |  |
| High \* No | 0.12 | 0.078 |  |  |  |
| Education: CASMIN \* Satisfaction: Life in general |  |  |  |  | 0.26 |
| Medium \* Scale value 4 to 7 | 0.10 | 0.111 |  |  |  |
| High \* Scale value 4 to 7 | 0.27 | 0.131 |  |  |  |
| Medium \* Scale value 8 to 10 | 0.07 | 0.116 |  |  |  |
| High \* Scale value 8 to 10 | 0.18 | 0.137 |  |  |  |
| Education: CASMIN \* Health risk due to climate change |  |  |  |  | 0.078 |
| Medium \* Scale value 4 to 7 | -0.02 | 0.063 |  |  |  |
| High \* Scale value 4 to 7 | -0.12 | 0.071 |  |  |  |
| Medium \* Scale value 8 to 10 | 0.10 | 0.094 |  |  |  |
| High \* Scale value 8 to 10 | 0.15 | 0.106 |  |  |  |
| Education: CASMIN \* Paying attention to health |  |  |  |  | 0.059 |
| Medium \* Strong/very strong | -0.11 | 0.059 |  |  |  |
| High \* Strong/very strong | 0.02 | 0.068 |  |  |  |
| Education: CASMIN \* Self-rated mental health |  |  |  |  | 0.868 |
| Medium \* fair/poor | 0.00 | 0.077 |  |  |  |
| High \* fair/poor | -0.05 | 0.103 |  |  |  |
| Education: CASMIN \* Smoking |  |  |  |  | 0.098 |
| Medium \* Occasional smoking | -0.12 | 0.152 |  |  |  |
| High \* Occasional smoking | 0.05 | 0.197 |  |  |  |
| Medium \* Non-smoker | 0.16 | 0.079 |  |  |  |
| High \* Non-smoker | 0.15 | 0.121 |  |  |  |
| Education: CASMIN \* Sausage products |  |  |  |  | 0.553 |
| Medium \* 4 to 6 times per week | 0.05 | 0.113 |  |  |  |
| High \* 4 to 6 times per week | -0.21 | 0.139 |  |  |  |
| Medium \* 1 to 3 times per week | -0.03 | 0.109 |  |  |  |
| High \* 1 to 3 times per week | -0.23 | 0.134 |  |  |  |
| Medium \* Less than once per week | 0.04 | 0.117 |  |  |  |
| High \* Less than once per week | -0.17 | 0.148 |  |  |  |
| Medium \* Never | -0.12 | 0.174 |  |  |  |
| High \* Never | -0.31 | 0.193 |  |  |  |
| Education: CASMIN \* Red meat |  |  |  |  | 0.058 |
| Medium \* 4 to 6 times per week | -0.04 | 0.193 |  |  |  |
| High \* 4 to 6 times per week | 0.18 | 0.239 |  |  |  |
| Medium \* 1 to 3 times per week | 0.10 | 0.186 |  |  |  |
| High \* 1 to 3 times per week | 0.37 | 0.236 |  |  |  |
| Medium \* Less than once per week | 0.26 | 0.184 |  |  |  |
| High \* Less than once per week | 0.56 | 0.236 |  |  |  |
| Medium \* Never | 0.23 | 0.218 |  |  |  |
| High \* Never | 0.44 | 0.275 |  |  |  |
| Education: CASMIN \* Waited for medical examination date in the last 12 months |  |  |  |  | 0.404 |
| Medium \* No | 0.12 | 0.067 |  |  |  |
| High \* No | 0.13 | 0.086 |  |  |  |
| Medium \* No need for examination or treatment | 0.14 | 0.112 |  |  |  |
| High \* No need for examination or treatment | 0.11 | 0.120 |  |  |  |
| Education: CASMIN \* Interview type |  |  |  |  | <0.001 |
| Medium \* Paper and Pencil Interview | -0.38 | 0.073 |  |  |  |
| High \* Paper and Pencil Interview | -0.45 | 0.086 |  |  |  |
| Education: CASMIN \* Foreign nationality |  |  |  |  | 0.278 |
| Medium \* Not foreign national | -0.05 | 0.106 |  |  |  |
| High \* Not foreign national | 0.11 | 0.113 |  |  |  |
| Education: CASMIN \* BIK community size (categorized) |  |  |  |  | 0.249 |
| Medium \* BIK region 20,000 to <50,000 inhabitants OR surroundings 50,000 to <500,000 inhabitants | -0.03 | 0.102 |  |  |  |
| High \* BIK region 20,000 to <50,000 inhabitants OR surroundings 50,000 to <500,000 inhabitants | 0.08 | 0.117 |  |  |  |
| Medium \* Core city 50,000 to <500,000 inhabitants OR surroundings 500,000+ inhabitants | 0.02 | 0.116 |  |  |  |
| High \* Core city 50,000 to <500,000 inhabitants OR surroundings 500,000+ inhabitants | -0.09 | 0.124 |  |  |  |
| Medium \* Core city 500,000+ inhabitants | 0.00 | 0.114 |  |  |  |
| High \* Core city 500,000+ inhabitants | 0.00 | 0.125 |  |  |  |
| Education: CASMIN \* Regions |  |  |  |  | 0.858 |
| Medium \* Northwest | 0.02 | 0.107 |  |  |  |
| High \* Northwest | 0.04 | 0.121 |  |  |  |
| Medium \* Central-East | 0.03 | 0.140 |  |  |  |
| High \* Central-East | 0.11 | 0.162 |  |  |  |
| Medium \* Central-West | -0.04 | 0.105 |  |  |  |
| High \* Central-West | -0.08 | 0.113 |  |  |  |
| Medium \* South | 0.04 | 0.108 |  |  |  |
| High \* South | -0.03 | 0.112 |  |  |  |
| Education: CASMIN \* Country of birth |  |  |  |  | 0.295 |
| Medium \* In another country | -0.13 | 0.096 |  |  |  |
| High \* In another country | -0.17 | 0.124 |  |  |  |
| Education: CASMIN \* German nationality |  |  |  |  | 0.118 |
| Medium \* No | -0.11 | 0.167 |  |  |  |
| High \* No | 0.26 | 0.183 |  |  |  |
| Education: CASMIN \* Current living situation |  |  |  |  | 0.06 |
| Medium \* Part-time employed | 0.03 | 0.105 |  |  |  |
| High \* Part-time employed | 0.09 | 0.123 |  |  |  |
| Medium \* Unemployed | -0.24 | 0.163 |  |  |  |
| High \* Unemployed | -0.14 | 0.219 |  |  |  |
| Medium \* Retired or early retired | 0.06 | 0.084 |  |  |  |
| High \* Retired or early retired | -0.12 | 0.109 |  |  |  |
| Medium \* Not employed for other reasons (student, volunteer service, homemaker) | 0.06 | 0.135 |  |  |  |
| High \* Not employed for other reasons (student, volunteer service, homemaker) | -0.26 | 0.159 |  |  |  |
| Education: CASMIN \* Underweight |  |  |  |  | 0.157 |
| Medium \* No Underweight (BMI >= 18.5) | -0.26 | 0.195 |  |  |  |
| High \* No Underweight (BMI >= 18.5) | 0.11 | 0.230 |  |  |  |
| N.Obs | 62,169 |  |  |  |  |
| N.Cluster | 359 |  |  |  |  |
|  |  |  |  |  |  |
| --- | --- | --- | --- | --- | --- |
| Abbreviations: CI = Confidence Interval, OR = Odds Ratio, SE = Standard Error | | | | | |

## Result model estimation: Questionaire A

### Quarter 2

| Characteristic | log(OR) | SE | 95% CI | p-value | p-value (global) |
| --- | --- | --- | --- | --- | --- |
| Sex |  |  |  |  | 0.598 |
| Male | — | — | — |  |  |
| Female | 0.08 | 0.159 | -0.23, 0.40 | 0.6 |  |
| Age |  |  |  |  | 0.163 |
| 16-29 yrs | — | — | — |  |  |
| 30-39 yrs | -0.72 | 0.805 | -2.3, 0.89 | 0.4 |  |
| 40-49 yrs | 0.12 | 0.906 | -1.7, 1.9 | 0.9 |  |
| 50-59 yrs | 0.39 | 0.871 | -1.4, 2.1 | 0.7 |  |
| 60-69 yrs | 2.1 | 1.11 | -0.13, 4.3 | 0.065 |  |
| 70-79 yrs | 1.4 | 1.34 | -1.3, 4.1 | 0.3 |  |
| 80+ | -1.4 | 1.67 | -4.8, 1.9 | 0.4 |  |
| Smoking |  |  |  |  | <0.001 |
| Daily smoking | — | — | — |  |  |
| Occasional smoking | 0.12 | 0.385 | -0.65, 0.89 | 0.8 |  |
| Non-smoker | 0.91 | 0.241 | 0.42, 1.4 | <0.001 |  |
| Red meat |  |  |  |  | 0.49 |
| Daily or several times a day | — | — | — |  |  |
| 4 to 6 times per week | -0.03 | 0.395 | -0.82, 0.76 | >0.9 |  |
| 1 to 3 times per week | -0.06 | 0.376 | -0.81, 0.69 | 0.9 |  |
| Less than once per week | -0.10 | 0.376 | -0.85, 0.65 | 0.8 |  |
| Never | 0.35 | 0.416 | -0.48, 1.2 | 0.4 |  |
| Waited for medical examination date in the last 12 months |  |  |  |  | 0.091 |
| Yes | — | — | — |  |  |
| No | -0.28 | 0.138 | -0.56, -0.01 | 0.044 |  |
| No need for examination or treatment | -0.28 | 0.169 | -0.61, 0.06 | 0.11 |  |
| BIK community size (categorized) |  |  |  |  | 0.543 |
| BIK region <20,000 inhabitants | — | — | — |  |  |
| BIK region 20,000 to <50,000 inhabitants OR surroundings 50,000 to <500,000 inhabitants | 0.21 | 0.323 | -0.43, 0.86 | 0.5 |  |
| Core city 50,000 to <500,000 inhabitants OR surroundings 500,000+ inhabitants | 0.14 | 0.348 | -0.56, 0.84 | 0.7 |  |
| Core city 500,000+ inhabitants | -0.13 | 0.339 | -0.81, 0.55 | 0.7 |  |
| German nationality |  |  |  |  | >0.9 |
| Yes | — | — | — |  |  |
| No | -0.04 | 0.330 | -0.70, 0.62 | >0.9 |  |
| Household size |  |  |  |  | 0.022 |
| Single-person household | — | — | — |  |  |
| Multi-person household | -0.18 | 0.079 | -0.34, -0.02 | 0.026 |  |
| Obesity |  |  |  |  | 0.53 |
| Yes | — | — | — |  |  |
| No | 0.17 | 0.270 | -0.37, 0.71 | 0.5 |  |
| Chronic diseases |  |  |  |  | 0.882 |
| Yes | — | — | — |  |  |
| No | 0.02 | 0.144 | -0.27, 0.31 | 0.9 |  |
| Satisfaction: Life in general |  |  |  |  | 0.356 |
| Scale value 1 to 3 | — | — | — |  |  |
| Scale value 4 to 7 | 0.16 | 0.247 | -0.34, 0.65 | 0.5 |  |
| Scale value 8 to 10 | 0.31 | 0.253 | -0.20, 0.81 | 0.2 |  |
| Health risk due to climate change |  |  |  |  | 0.372 |
| Scale value 1 to 3 | — | — | — |  |  |
| Scale value 4 to 7 | 0.10 | 0.143 | -0.19, 0.38 | 0.5 |  |
| Scale value 8 to 10 | -0.12 | 0.178 | -0.47, 0.24 | 0.5 |  |
| Sausage products |  |  |  |  | 0.763 |
| Daily or several times a day | — | — | — |  |  |
| 4 to 6 times per week | 0.02 | 0.355 | -0.69, 0.73 | >0.9 |  |
| 1 to 3 times per week | -0.17 | 0.330 | -0.83, 0.49 | 0.6 |  |
| Less than once per week | 0.17 | 0.327 | -0.49, 0.82 | 0.6 |  |
| Never | 0.09 | 0.421 | -0.75, 0.94 | 0.8 |  |
| Sport |  |  |  |  | 0.773 |
| No sporting activities | — | — | — |  |  |
| Less than 1 hour per week | 0.07 | 0.215 | -0.36, 0.50 | 0.7 |  |
| 1 to less than 2 hours per week | 0.19 | 0.220 | -0.25, 0.63 | 0.4 |  |
| 2 to less than 4 hours per week | 0.20 | 0.227 | -0.26, 0.65 | 0.4 |  |
| 4 hours per week and more | 0.27 | 0.217 | -0.17, 0.70 | 0.2 |  |
| Interview type |  |  |  |  | 0.011 |
| Computer Assisted Web Interview | — | — | — |  |  |
| Paper and Pencil Interview | -0.65 | 0.257 | -1.2, -0.14 | 0.014 |  |
| Regions |  |  |  |  | 0.489 |
| Northeast | — | — | — |  |  |
| Northwest | -0.48 | 0.324 | -1.1, 0.17 | 0.14 |  |
| Central-East | -0.25 | 0.397 | -1.0, 0.55 | 0.5 |  |
| Central-West | -0.31 | 0.306 | -0.92, 0.30 | 0.3 |  |
| South | -0.07 | 0.310 | -0.69, 0.55 | 0.8 |  |
| Country of birth |  |  |  |  | 0.722 |
| In Germany (within current borders) | — | — | — |  |  |
| In another country | -0.13 | 0.360 | -0.85, 0.59 | 0.7 |  |
| Education: ISCED (2011) |  |  |  |  | 0.032 |
| Low | — | — | — |  |  |
| Medium | 0.29 | 0.187 | -0.08, 0.66 | 0.13 |  |
| High | -0.16 | 0.238 | -0.63, 0.32 | 0.5 |  |
| Education: CASMIN |  |  |  |  | 0.251 |
| Low | — | — | — |  |  |
| Medium | 0.29 | 0.378 | -0.47, 1.0 | 0.4 |  |
| High | 0.84 | 0.505 | -0.17, 1.8 | 0.10 |  |
| Overweight |  |  |  |  | 0.114 |
| Yes | — | — | — |  |  |
| No | -0.23 | 0.145 | -0.52, 0.06 | 0.12 |  |
| Current living situation |  |  |  |  | 0.046 |
| Full-time employed | — | — | — |  |  |
| Part-time employed | -0.13 | 0.225 | -0.58, 0.32 | 0.6 |  |
| Unemployed | 0.24 | 0.289 | -0.33, 0.82 | 0.4 |  |
| Retired or early retired | 0.49 | 0.199 | 0.09, 0.89 | 0.017 |  |
| Not employed for other reasons (student, volunteer service, homemaker) | -0.03 | 0.240 | -0.51, 0.45 | >0.9 |  |
| Age \* Obesity |  |  |  |  | 0.505 |
| 30-39 yrs \* No | -0.02 | 0.250 | -0.52, 0.48 | >0.9 |  |
| 40-49 yrs \* No | 0.07 | 0.290 | -0.51, 0.65 | 0.8 |  |
| 50-59 yrs \* No | -0.11 | 0.269 | -0.65, 0.43 | 0.7 |  |
| 60-69 yrs \* No | 0.07 | 0.299 | -0.53, 0.66 | 0.8 |  |
| 70-79 yrs \* No | -0.63 | 0.374 | -1.4, 0.12 | 0.10 |  |
| 80+ \* No | 0.20 | 0.439 | -0.68, 1.1 | 0.6 |  |
| Age \* Chronic diseases |  |  |  |  | 0.01 |
| 30-39 yrs \* No | -0.49 | 0.214 | -0.91, -0.06 | 0.026 |  |
| 40-49 yrs \* No | 0.48 | 0.219 | 0.04, 0.92 | 0.033 |  |
| 50-59 yrs \* No | -0.11 | 0.228 | -0.57, 0.34 | 0.6 |  |
| 60-69 yrs \* No | 0.10 | 0.224 | -0.35, 0.55 | 0.7 |  |
| 70-79 yrs \* No | -0.07 | 0.300 | -0.67, 0.53 | 0.8 |  |
| 80+ \* No | -0.08 | 0.338 | -0.76, 0.59 | 0.8 |  |
| Age \* Satisfaction: Life in general |  |  |  |  | 0.344 |
| 30-39 yrs \* Scale value 4 to 7 | 0.46 | 0.362 | -0.26, 1.2 | 0.2 |  |
| 40-49 yrs \* Scale value 4 to 7 | -0.32 | 0.397 | -1.1, 0.47 | 0.4 |  |
| 50-59 yrs \* Scale value 4 to 7 | 0.07 | 0.380 | -0.69, 0.83 | 0.9 |  |
| 60-69 yrs \* Scale value 4 to 7 | -0.32 | 0.400 | -1.1, 0.48 | 0.4 |  |
| 70-79 yrs \* Scale value 4 to 7 | -0.21 | 0.489 | -1.2, 0.77 | 0.7 |  |
| 80+ \* Scale value 4 to 7 | 0.64 | 0.498 | -0.35, 1.6 | 0.2 |  |
| 30-39 yrs \* Scale value 8 to 10 | 0.33 | 0.370 | -0.41, 1.1 | 0.4 |  |
| 40-49 yrs \* Scale value 8 to 10 | -0.58 | 0.391 | -1.4, 0.21 | 0.15 |  |
| 50-59 yrs \* Scale value 8 to 10 | 0.10 | 0.377 | -0.66, 0.85 | 0.8 |  |
| 60-69 yrs \* Scale value 8 to 10 | -0.11 | 0.411 | -0.93, 0.72 | 0.8 |  |
| 70-79 yrs \* Scale value 8 to 10 | 0.01 | 0.502 | -0.99, 1.0 | >0.9 |  |
| 80+ \* Scale value 8 to 10 | 0.34 | 0.507 | -0.68, 1.4 | 0.5 |  |
| Age \* Health risk due to climate change |  |  |  |  | 0.248 |
| 30-39 yrs \* Scale value 4 to 7 | 0.16 | 0.203 | -0.25, 0.56 | 0.4 |  |
| 40-49 yrs \* Scale value 4 to 7 | -0.04 | 0.224 | -0.49, 0.40 | 0.8 |  |
| 50-59 yrs \* Scale value 4 to 7 | -0.08 | 0.225 | -0.53, 0.37 | 0.7 |  |
| 60-69 yrs \* Scale value 4 to 7 | -0.22 | 0.232 | -0.69, 0.24 | 0.3 |  |
| 70-79 yrs \* Scale value 4 to 7 | 0.24 | 0.272 | -0.31, 0.78 | 0.4 |  |
| 80+ \* Scale value 4 to 7 | 0.05 | 0.292 | -0.53, 0.64 | 0.9 |  |
| 30-39 yrs \* Scale value 8 to 10 | 0.29 | 0.272 | -0.26, 0.83 | 0.3 |  |
| 40-49 yrs \* Scale value 8 to 10 | 0.58 | 0.280 | 0.02, 1.1 | 0.044 |  |
| 50-59 yrs \* Scale value 8 to 10 | -0.22 | 0.283 | -0.79, 0.35 | 0.4 |  |
| 60-69 yrs \* Scale value 8 to 10 | 0.09 | 0.304 | -0.51, 0.70 | 0.8 |  |
| 70-79 yrs \* Scale value 8 to 10 | 0.17 | 0.398 | -0.62, 0.97 | 0.7 |  |
| 80+ \* Scale value 8 to 10 | 0.35 | 0.399 | -0.45, 1.1 | 0.4 |  |
| Age \* Smoking |  |  |  |  | 0.044 |
| 30-39 yrs \* Occasional smoking | -0.13 | 0.386 | -0.91, 0.64 | 0.7 |  |
| 40-49 yrs \* Occasional smoking | 0.42 | 0.425 | -0.43, 1.3 | 0.3 |  |
| 50-59 yrs \* Occasional smoking | 0.09 | 0.467 | -0.84, 1.0 | 0.8 |  |
| 60-69 yrs \* Occasional smoking | -0.22 | 0.536 | -1.3, 0.85 | 0.7 |  |
| 70-79 yrs \* Occasional smoking | 0.85 | 1.16 | -1.5, 3.2 | 0.5 |  |
| 80+ \* Occasional smoking | 0.68 | 0.956 | -1.2, 2.6 | 0.5 |  |
| 30-39 yrs \* Non-smoker | -0.36 | 0.260 | -0.88, 0.16 | 0.2 |  |
| 40-49 yrs \* Non-smoker | 0.11 | 0.276 | -0.45, 0.66 | 0.7 |  |
| 50-59 yrs \* Non-smoker | -0.54 | 0.264 | -1.1, -0.02 | 0.044 |  |
| 60-69 yrs \* Non-smoker | -0.32 | 0.274 | -0.87, 0.23 | 0.3 |  |
| 70-79 yrs \* Non-smoker | -0.89 | 0.364 | -1.6, -0.16 | 0.017 |  |
| 80+ \* Non-smoker | 0.88 | 0.651 | -0.42, 2.2 | 0.2 |  |
| Age \* Sausage products |  |  |  |  | 0.066 |
| 30-39 yrs \* 4 to 6 times per week | -0.26 | 0.403 | -1.1, 0.54 | 0.5 |  |
| 40-49 yrs \* 4 to 6 times per week | 0.15 | 0.382 | -0.61, 0.92 | 0.7 |  |
| 50-59 yrs \* 4 to 6 times per week | 0.71 | 0.366 | -0.03, 1.4 | 0.059 |  |
| 60-69 yrs \* 4 to 6 times per week | 0.42 | 0.436 | -0.45, 1.3 | 0.3 |  |
| 70-79 yrs \* 4 to 6 times per week | -0.80 | 0.520 | -1.8, 0.24 | 0.13 |  |
| 80+ \* 4 to 6 times per week | 0.51 | 0.590 | -0.67, 1.7 | 0.4 |  |
| 30-39 yrs \* 1 to 3 times per week | -0.13 | 0.364 | -0.85, 0.60 | 0.7 |  |
| 40-49 yrs \* 1 to 3 times per week | 0.63 | 0.357 | -0.09, 1.3 | 0.084 |  |
| 50-59 yrs \* 1 to 3 times per week | 0.74 | 0.349 | 0.04, 1.4 | 0.039 |  |
| 60-69 yrs \* 1 to 3 times per week | 0.46 | 0.384 | -0.30, 1.2 | 0.2 |  |
| 70-79 yrs \* 1 to 3 times per week | -0.20 | 0.492 | -1.2, 0.78 | 0.7 |  |
| 80+ \* 1 to 3 times per week | -0.03 | 0.522 | -1.1, 1.0 | >0.9 |  |
| 30-39 yrs \* Less than once per week | -0.03 | 0.384 | -0.80, 0.73 | >0.9 |  |
| 40-49 yrs \* Less than once per week | 0.24 | 0.378 | -0.52, 1.0 | 0.5 |  |
| 50-59 yrs \* Less than once per week | 0.51 | 0.365 | -0.22, 1.2 | 0.2 |  |
| 60-69 yrs \* Less than once per week | 0.29 | 0.438 | -0.59, 1.2 | 0.5 |  |
| 70-79 yrs \* Less than once per week | -0.37 | 0.520 | -1.4, 0.67 | 0.5 |  |
| 80+ \* Less than once per week | 0.04 | 0.569 | -1.1, 1.2 | >0.9 |  |
| 30-39 yrs \* Never | -0.34 | 0.483 | -1.3, 0.63 | 0.5 |  |
| 40-49 yrs \* Never | -0.54 | 0.509 | -1.6, 0.48 | 0.3 |  |
| 50-59 yrs \* Never | 0.83 | 0.473 | -0.11, 1.8 | 0.083 |  |
| 60-69 yrs \* Never | 0.64 | 0.612 | -0.59, 1.9 | 0.3 |  |
| 70-79 yrs \* Never | -0.34 | 0.674 | -1.7, 1.0 | 0.6 |  |
| 80+ \* Never | 0.51 | 0.772 | -1.0, 2.1 | 0.5 |  |
| Age \* Red meat |  |  |  |  | 0.096 |
| 30-39 yrs \* 4 to 6 times per week | 0.11 | 0.570 | -1.0, 1.3 | 0.8 |  |
| 40-49 yrs \* 4 to 6 times per week | -0.23 | 0.677 | -1.6, 1.1 | 0.7 |  |
| 50-59 yrs \* 4 to 6 times per week | 0.57 | 0.614 | -0.66, 1.8 | 0.4 |  |
| 60-69 yrs \* 4 to 6 times per week | -0.84 | 0.971 | -2.8, 1.1 | 0.4 |  |
| 70-79 yrs \* 4 to 6 times per week | 0.04 | 1.02 | -2.0, 2.1 | >0.9 |  |
| 80+ \* 4 to 6 times per week | 0.61 | 1.28 | -1.9, 3.2 | 0.6 |  |
| 30-39 yrs \* 1 to 3 times per week | 0.24 | 0.549 | -0.86, 1.3 | 0.7 |  |
| 40-49 yrs \* 1 to 3 times per week | -0.04 | 0.624 | -1.3, 1.2 | >0.9 |  |
| 50-59 yrs \* 1 to 3 times per week | 0.27 | 0.576 | -0.88, 1.4 | 0.6 |  |
| 60-69 yrs \* 1 to 3 times per week | -1.1 | 0.930 | -3.0, 0.76 | 0.2 |  |
| 70-79 yrs \* 1 to 3 times per week | 0.19 | 0.963 | -1.7, 2.1 | 0.8 |  |
| 80+ \* 1 to 3 times per week | -0.29 | 1.15 | -2.6, 2.0 | 0.8 |  |
| 30-39 yrs \* Less than once per week | 0.48 | 0.568 | -0.66, 1.6 | 0.4 |  |
| 40-49 yrs \* Less than once per week | -0.28 | 0.653 | -1.6, 1.0 | 0.7 |  |
| 50-59 yrs \* Less than once per week | 0.56 | 0.577 | -0.60, 1.7 | 0.3 |  |
| 60-69 yrs \* Less than once per week | -0.53 | 0.963 | -2.5, 1.4 | 0.6 |  |
| 70-79 yrs \* Less than once per week | -0.10 | 0.996 | -2.1, 1.9 | >0.9 |  |
| 80+ \* Less than once per week | 0.16 | 1.17 | -2.2, 2.5 | 0.9 |  |
| 30-39 yrs \* Never | 0.17 | 0.671 | -1.2, 1.5 | 0.8 |  |
| 40-49 yrs \* Never | -0.16 | 0.798 | -1.8, 1.4 | 0.8 |  |
| 50-59 yrs \* Never | 0.13 | 0.654 | -1.2, 1.4 | 0.8 |  |
| 60-69 yrs \* Never | -1.7 | 1.03 | -3.8, 0.34 | 0.10 |  |
| 70-79 yrs \* Never | 0.07 | 1.06 | -2.1, 2.2 | >0.9 |  |
| 80+ \* Never | -1.2 | 1.20 | -3.6, 1.2 | 0.3 |  |
| Age \* Sport |  |  |  |  | 0.1 |
| 30-39 yrs \* Less than 1 hour per week | 0.17 | 0.304 | -0.44, 0.78 | 0.6 |  |
| 40-49 yrs \* Less than 1 hour per week | 0.07 | 0.303 | -0.54, 0.67 | 0.8 |  |
| 50-59 yrs \* Less than 1 hour per week | 0.10 | 0.337 | -0.57, 0.78 | 0.8 |  |
| 60-69 yrs \* Less than 1 hour per week | -0.12 | 0.365 | -0.85, 0.61 | 0.7 |  |
| 70-79 yrs \* Less than 1 hour per week | 0.33 | 0.383 | -0.44, 1.1 | 0.4 |  |
| 80+ \* Less than 1 hour per week | -0.18 | 0.413 | -1.0, 0.65 | 0.7 |  |
| 30-39 yrs \* 1 to less than 2 hours per week | -0.09 | 0.297 | -0.68, 0.50 | 0.8 |  |
| 40-49 yrs \* 1 to less than 2 hours per week | -0.13 | 0.319 | -0.77, 0.51 | 0.7 |  |
| 50-59 yrs \* 1 to less than 2 hours per week | -0.01 | 0.326 | -0.66, 0.64 | >0.9 |  |
| 60-69 yrs \* 1 to less than 2 hours per week | -0.24 | 0.347 | -0.93, 0.46 | 0.5 |  |
| 70-79 yrs \* 1 to less than 2 hours per week | -0.08 | 0.338 | -0.76, 0.60 | 0.8 |  |
| 80+ \* 1 to less than 2 hours per week | -0.09 | 0.436 | -0.97, 0.78 | 0.8 |  |
| 30-39 yrs \* 2 to less than 4 hours per week | 0.09 | 0.336 | -0.58, 0.76 | 0.8 |  |
| 40-49 yrs \* 2 to less than 4 hours per week | -0.32 | 0.350 | -1.0, 0.38 | 0.4 |  |
| 50-59 yrs \* 2 to less than 4 hours per week | -0.28 | 0.351 | -0.98, 0.42 | 0.4 |  |
| 60-69 yrs \* 2 to less than 4 hours per week | -0.10 | 0.365 | -0.83, 0.63 | 0.8 |  |
| 70-79 yrs \* 2 to less than 4 hours per week | 0.44 | 0.365 | -0.29, 1.2 | 0.2 |  |
| 80+ \* 2 to less than 4 hours per week | -0.02 | 0.488 | -0.99, 0.96 | >0.9 |  |
| 30-39 yrs \* 4 hours per week and more | -0.66 | 0.327 | -1.3, 0.00 | 0.049 |  |
| 40-49 yrs \* 4 hours per week and more | -0.05 | 0.400 | -0.85, 0.75 | >0.9 |  |
| 50-59 yrs \* 4 hours per week and more | -0.67 | 0.369 | -1.4, 0.07 | 0.075 |  |
| 60-69 yrs \* 4 hours per week and more | -0.46 | 0.417 | -1.3, 0.38 | 0.3 |  |
| 70-79 yrs \* 4 hours per week and more | 0.79 | 0.437 | -0.08, 1.7 | 0.075 |  |
| 80+ \* 4 hours per week and more | 1.2 | 0.688 | -0.22, 2.5 | 0.10 |  |
| Age \* Waited for medical examination date in the last 12 months |  |  |  |  | 0.559 |
| 30-39 yrs \* No | 0.17 | 0.214 | -0.26, 0.60 | 0.4 |  |
| 40-49 yrs \* No | 0.31 | 0.222 | -0.13, 0.76 | 0.2 |  |
| 50-59 yrs \* No | 0.28 | 0.193 | -0.11, 0.67 | 0.2 |  |
| 60-69 yrs \* No | 0.35 | 0.239 | -0.13, 0.83 | 0.2 |  |
| 70-79 yrs \* No | 0.39 | 0.288 | -0.19, 0.96 | 0.2 |  |
| 80+ \* No | 0.41 | 0.339 | -0.26, 1.1 | 0.2 |  |
| 30-39 yrs \* No need for examination or treatment | 0.21 | 0.261 | -0.31, 0.73 | 0.4 |  |
| 40-49 yrs \* No need for examination or treatment | 0.37 | 0.307 | -0.25, 0.98 | 0.2 |  |
| 50-59 yrs \* No need for examination or treatment | -0.06 | 0.296 | -0.65, 0.54 | 0.9 |  |
| 60-69 yrs \* No need for examination or treatment | 0.93 | 0.402 | 0.13, 1.7 | 0.024 |  |
| 70-79 yrs \* No need for examination or treatment | 0.31 | 0.627 | -0.94, 1.6 | 0.6 |  |
| 80+ \* No need for examination or treatment | 0.43 | 0.887 | -1.3, 2.2 | 0.6 |  |
| Age \* Interview type |  |  |  |  | <0.001 |
| 30-39 yrs \* Paper and Pencil Interview | 0.19 | 0.389 | -0.59, 0.97 | 0.6 |  |
| 40-49 yrs \* Paper and Pencil Interview | -0.11 | 0.366 | -0.84, 0.63 | 0.8 |  |
| 50-59 yrs \* Paper and Pencil Interview | 0.05 | 0.306 | -0.56, 0.66 | 0.9 |  |
| 60-69 yrs \* Paper and Pencil Interview | 0.50 | 0.341 | -0.18, 1.2 | 0.14 |  |
| 70-79 yrs \* Paper and Pencil Interview | 1.2 | 0.345 | 0.50, 1.9 | 0.001 |  |
| 80+ \* Paper and Pencil Interview | 0.83 | 0.431 | -0.03, 1.7 | 0.060 |  |
| Age \* BIK community size (categorized) |  |  |  |  | 0.056 |
| 30-39 yrs \* BIK region 20,000 to <50,000 inhabitants OR surroundings 50,000 to <500,000 inhabitants | 0.53 | 0.351 | -0.17, 1.2 | 0.14 |  |
| 40-49 yrs \* BIK region 20,000 to <50,000 inhabitants OR surroundings 50,000 to <500,000 inhabitants | 0.24 | 0.395 | -0.55, 1.0 | 0.6 |  |
| 50-59 yrs \* BIK region 20,000 to <50,000 inhabitants OR surroundings 50,000 to <500,000 inhabitants | 0.23 | 0.378 | -0.52, 0.99 | 0.5 |  |
| 60-69 yrs \* BIK region 20,000 to <50,000 inhabitants OR surroundings 50,000 to <500,000 inhabitants | 1.0 | 0.393 | 0.24, 1.8 | 0.012 |  |
| 70-79 yrs \* BIK region 20,000 to <50,000 inhabitants OR surroundings 50,000 to <500,000 inhabitants | -0.32 | 0.424 | -1.2, 0.53 | 0.5 |  |
| 80+ \* BIK region 20,000 to <50,000 inhabitants OR surroundings 50,000 to <500,000 inhabitants | -0.26 | 0.628 | -1.5, 1.0 | 0.7 |  |
| 30-39 yrs \* Core city 50,000 to <500,000 inhabitants OR surroundings 500,000+ inhabitants | 0.50 | 0.364 | -0.22, 1.2 | 0.2 |  |
| 40-49 yrs \* Core city 50,000 to <500,000 inhabitants OR surroundings 500,000+ inhabitants | 0.11 | 0.409 | -0.71, 0.93 | 0.8 |  |
| 50-59 yrs \* Core city 50,000 to <500,000 inhabitants OR surroundings 500,000+ inhabitants | -0.05 | 0.400 | -0.85, 0.75 | 0.9 |  |
| 60-69 yrs \* Core city 50,000 to <500,000 inhabitants OR surroundings 500,000+ inhabitants | 0.56 | 0.442 | -0.33, 1.4 | 0.2 |  |
| 70-79 yrs \* Core city 50,000 to <500,000 inhabitants OR surroundings 500,000+ inhabitants | -0.11 | 0.470 | -1.0, 0.83 | 0.8 |  |
| 80+ \* Core city 50,000 to <500,000 inhabitants OR surroundings 500,000+ inhabitants | -0.15 | 0.654 | -1.5, 1.2 | 0.8 |  |
| 30-39 yrs \* Core city 500,000+ inhabitants | 0.69 | 0.354 | -0.01, 1.4 | 0.054 |  |
| 40-49 yrs \* Core city 500,000+ inhabitants | 0.54 | 0.401 | -0.26, 1.3 | 0.2 |  |
| 50-59 yrs \* Core city 500,000+ inhabitants | 0.57 | 0.394 | -0.22, 1.4 | 0.2 |  |
| 60-69 yrs \* Core city 500,000+ inhabitants | 0.63 | 0.422 | -0.22, 1.5 | 0.14 |  |
| 70-79 yrs \* Core city 500,000+ inhabitants | 0.21 | 0.469 | -0.73, 1.1 | 0.7 |  |
| 80+ \* Core city 500,000+ inhabitants | -0.19 | 0.622 | -1.4, 1.1 | 0.8 |  |
| Age \* Regions |  |  |  |  | 0.076 |
| 30-39 yrs \* Northwest | 0.28 | 0.324 | -0.37, 0.93 | 0.4 |  |
| 40-49 yrs \* Northwest | 0.07 | 0.343 | -0.61, 0.76 | 0.8 |  |
| 50-59 yrs \* Northwest | 0.43 | 0.368 | -0.31, 1.2 | 0.2 |  |
| 60-69 yrs \* Northwest | -0.04 | 0.412 | -0.86, 0.79 | >0.9 |  |
| 70-79 yrs \* Northwest | 0.34 | 0.463 | -0.58, 1.3 | 0.5 |  |
| 80+ \* Northwest | 0.38 | 0.614 | -0.85, 1.6 | 0.5 |  |
| 30-39 yrs \* Central-East | 0.30 | 0.420 | -0.54, 1.1 | 0.5 |  |
| 40-49 yrs \* Central-East | 0.11 | 0.490 | -0.87, 1.1 | 0.8 |  |
| 50-59 yrs \* Central-East | -0.22 | 0.445 | -1.1, 0.67 | 0.6 |  |
| 60-69 yrs \* Central-East | -0.34 | 0.552 | -1.4, 0.76 | 0.5 |  |
| 70-79 yrs \* Central-East | -1.3 | 0.482 | -2.2, -0.30 | 0.011 |  |
| 80+ \* Central-East | -0.05 | 0.665 | -1.4, 1.3 | >0.9 |  |
| 30-39 yrs \* Central-West | 0.22 | 0.290 | -0.36, 0.81 | 0.4 |  |
| 40-49 yrs \* Central-West | 0.20 | 0.334 | -0.47, 0.86 | 0.6 |  |
| 50-59 yrs \* Central-West | 0.37 | 0.346 | -0.32, 1.1 | 0.3 |  |
| 60-69 yrs \* Central-West | -0.17 | 0.418 | -1.0, 0.67 | 0.7 |  |
| 70-79 yrs \* Central-West | -0.14 | 0.381 | -0.90, 0.63 | 0.7 |  |
| 80+ \* Central-West | 0.14 | 0.571 | -1.0, 1.3 | 0.8 |  |
| 30-39 yrs \* South | 0.22 | 0.304 | -0.39, 0.83 | 0.5 |  |
| 40-49 yrs \* South | -0.15 | 0.336 | -0.83, 0.52 | 0.6 |  |
| 50-59 yrs \* South | -0.02 | 0.328 | -0.68, 0.63 | >0.9 |  |
| 60-69 yrs \* South | -0.57 | 0.402 | -1.4, 0.23 | 0.2 |  |
| 70-79 yrs \* South | -0.37 | 0.388 | -1.1, 0.41 | 0.3 |  |
| 80+ \* South | 0.41 | 0.588 | -0.77, 1.6 | 0.5 |  |
| Age \* Country of birth |  |  |  |  | 0.228 |
| 30-39 yrs \* In another country | 0.28 | 0.432 | -0.58, 1.1 | 0.5 |  |
| 40-49 yrs \* In another country | -0.21 | 0.368 | -0.95, 0.53 | 0.6 |  |
| 50-59 yrs \* In another country | 0.40 | 0.437 | -0.47, 1.3 | 0.4 |  |
| 60-69 yrs \* In another country | -0.21 | 0.534 | -1.3, 0.86 | 0.7 |  |
| 70-79 yrs \* In another country | -0.56 | 0.450 | -1.5, 0.34 | 0.2 |  |
| 80+ \* In another country | 0.33 | 0.459 | -0.58, 1.3 | 0.5 |  |
| Age \* German nationality |  |  |  |  | 0.28 |
| 30-39 yrs \* No | 0.08 | 0.485 | -0.89, 1.1 | 0.9 |  |
| 40-49 yrs \* No | -0.56 | 0.471 | -1.5, 0.38 | 0.2 |  |
| 50-59 yrs \* No | -1.1 | 0.480 | -2.1, -0.17 | 0.022 |  |
| 60-69 yrs \* No | -0.23 | 0.563 | -1.4, 0.90 | 0.7 |  |
| 70-79 yrs \* No | -0.59 | 0.658 | -1.9, 0.72 | 0.4 |  |
| 80+ \* No | -0.02 | 1.01 | -2.0, 2.0 | >0.9 |  |
| Age \* Education: ISCED (2011) |  |  |  |  | 0.075 |
| 30-39 yrs \* Medium | -0.19 | 0.318 | -0.82, 0.45 | 0.6 |  |
| 40-49 yrs \* Medium | -0.11 | 0.348 | -0.81, 0.59 | 0.8 |  |
| 50-59 yrs \* Medium | -0.49 | 0.392 | -1.3, 0.30 | 0.2 |  |
| 60-69 yrs \* Medium | -0.71 | 0.418 | -1.5, 0.13 | 0.10 |  |
| 70-79 yrs \* Medium | 0.58 | 0.380 | -0.17, 1.3 | 0.13 |  |
| 80+ \* Medium | -0.28 | 0.419 | -1.1, 0.56 | 0.5 |  |
| 30-39 yrs \* High | 0.29 | 0.356 | -0.42, 1.0 | 0.4 |  |
| 40-49 yrs \* High | 0.01 | 0.380 | -0.75, 0.78 | >0.9 |  |
| 50-59 yrs \* High | 0.08 | 0.407 | -0.74, 0.89 | 0.9 |  |
| 60-69 yrs \* High | -0.28 | 0.432 | -1.1, 0.58 | 0.5 |  |
| 70-79 yrs \* High | 0.97 | 0.416 | 0.14, 1.8 | 0.023 |  |
| 80+ \* High | 0.31 | 0.462 | -0.62, 1.2 | 0.5 |  |
| Sex \* Regions |  |  |  |  | 0.412 |
| Female \* Northwest | 0.14 | 0.210 | -0.28, 0.56 | 0.5 |  |
| Female \* Central-East | 0.38 | 0.224 | -0.07, 0.83 | 0.10 |  |
| Female \* Central-West | 0.03 | 0.180 | -0.33, 0.39 | 0.9 |  |
| Female \* South | 0.10 | 0.195 | -0.30, 0.49 | 0.6 |  |
| Sex \* Country of birth |  |  |  |  | >0.9 |
| Female \* In another country | -0.02 | 0.184 | -0.39, 0.35 | >0.9 |  |
| Obesity \* Education: CASMIN |  |  |  |  | 0.327 |
| No \* Medium | -0.09 | 0.182 | -0.46, 0.27 | 0.6 |  |
| No \* High | 0.21 | 0.231 | -0.25, 0.68 | 0.4 |  |
| Education: CASMIN \* Overweight |  |  |  |  | 0.073 |
| Medium \* No | 0.38 | 0.168 | 0.05, 0.72 | 0.027 |  |
| High \* No | 0.34 | 0.198 | -0.06, 0.73 | 0.10 |  |
| Smoking \* Education: CASMIN |  |  |  |  | 0.052 |
| Occasional smoking \* Medium | 0.32 | 0.362 | -0.41, 1.0 | 0.4 |  |
| Non-smoker \* Medium | 0.08 | 0.202 | -0.32, 0.48 | 0.7 |  |
| Occasional smoking \* High | -0.28 | 0.412 | -1.1, 0.55 | 0.5 |  |
| Non-smoker \* High | -0.57 | 0.248 | -1.1, -0.07 | 0.026 |  |
| Sausage products \* Education: CASMIN |  |  |  |  | 0.601 |
| 4 to 6 times per week \* Medium | -0.05 | 0.284 | -0.62, 0.52 | 0.9 |  |
| 1 to 3 times per week \* Medium | -0.02 | 0.260 | -0.54, 0.50 | >0.9 |  |
| Less than once per week \* Medium | -0.25 | 0.280 | -0.81, 0.31 | 0.4 |  |
| Never \* Medium | -0.46 | 0.359 | -1.2, 0.25 | 0.2 |  |
| 4 to 6 times per week \* High | 0.05 | 0.346 | -0.64, 0.74 | 0.9 |  |
| 1 to 3 times per week \* High | -0.14 | 0.317 | -0.77, 0.50 | 0.7 |  |
| Less than once per week \* High | -0.28 | 0.354 | -0.99, 0.43 | 0.4 |  |
| Never \* High | -0.20 | 0.403 | -1.0, 0.60 | 0.6 |  |
| BIK community size (categorized) \* Education: CASMIN |  |  |  |  | 0.794 |
| BIK region 20,000 to <50,000 inhabitants OR surroundings 50,000 to <500,000 inhabitants \* Medium | -0.25 | 0.241 | -0.73, 0.23 | 0.3 |  |
| Core city 50,000 to <500,000 inhabitants OR surroundings 500,000+ inhabitants \* Medium | -0.31 | 0.243 | -0.80, 0.17 | 0.2 |  |
| Core city 500,000+ inhabitants \* Medium | -0.09 | 0.260 | -0.61, 0.43 | 0.7 |  |
| BIK region 20,000 to <50,000 inhabitants OR surroundings 50,000 to <500,000 inhabitants \* High | -0.24 | 0.301 | -0.84, 0.37 | 0.4 |  |
| Core city 50,000 to <500,000 inhabitants OR surroundings 500,000+ inhabitants \* High | -0.34 | 0.305 | -0.95, 0.27 | 0.3 |  |
| Core city 500,000+ inhabitants \* High | -0.24 | 0.316 | -0.88, 0.39 | 0.4 |  |
| Regions \* Education: CASMIN |  |  |  |  | 0.208 |
| Northwest \* Medium | 0.07 | 0.298 | -0.52, 0.67 | 0.8 |  |
| Central-East \* Medium | 0.16 | 0.368 | -0.57, 0.90 | 0.7 |  |
| Central-West \* Medium | 0.15 | 0.275 | -0.40, 0.71 | 0.6 |  |
| South \* Medium | -0.01 | 0.268 | -0.55, 0.53 | >0.9 |  |
| Northwest \* High | 0.29 | 0.324 | -0.36, 0.93 | 0.4 |  |
| Central-East \* High | 0.80 | 0.386 | 0.02, 1.6 | 0.043 |  |
| Central-West \* High | 0.07 | 0.276 | -0.49, 0.62 | 0.8 |  |
| South \* High | 0.23 | 0.252 | -0.27, 0.74 | 0.4 |  |
| Country of birth \* Education: CASMIN |  |  |  |  | 0.216 |
| In another country \* Medium | -0.31 | 0.238 | -0.78, 0.17 | 0.2 |  |
| In another country \* High | 0.01 | 0.245 | -0.49, 0.50 | >0.9 |  |
| Education: CASMIN \* Current living situation |  |  |  |  | >0.9 |
| Medium \* Part-time employed | 0.28 | 0.259 | -0.23, 0.80 | 0.3 |  |
| High \* Part-time employed | 0.06 | 0.281 | -0.51, 0.62 | 0.8 |  |
| Medium \* Unemployed | 0.15 | 0.376 | -0.60, 0.90 | 0.7 |  |
| High \* Unemployed | -0.05 | 0.439 | -0.93, 0.83 | >0.9 |  |
| Medium \* Retired or early retired | 0.00 | 0.220 | -0.44, 0.44 | >0.9 |  |
| High \* Retired or early retired | -0.19 | 0.312 | -0.81, 0.44 | 0.6 |  |
| Medium \* Not employed for other reasons (student, volunteer service, homemaker) | 0.14 | 0.261 | -0.38, 0.66 | 0.6 |  |
| High \* Not employed for other reasons (student, volunteer service, homemaker) | -0.02 | 0.345 | -0.71, 0.67 | >0.9 |  |
| N.Obs | 11,765 |  |  |  |  |
| N.Cluster | 359 |  |  |  |  |
|  |  |  |  |  |  |
| --- | --- | --- | --- | --- | --- |
| Abbreviations: CI = Confidence Interval, OR = Odds Ratio, SE = Standard Error | | | | | |

### Quarter 3

| Characteristic | log(OR) | SE | 95% CI | p-value | p-value (global) |
| --- | --- | --- | --- | --- | --- |
| Sex |  |  |  |  | 0.2 |
| Male | — | — | — |  |  |
| Female | -0.56 | 0.440 | -1.4, 0.31 | 0.2 |  |
| Age |  |  |  |  | 0.683 |
| 16-29 yrs | — | — | — |  |  |
| 30-39 yrs | -0.60 | 0.704 | -2.0, 0.80 | 0.4 |  |
| 40-49 yrs | -0.05 | 0.809 | -1.7, 1.6 | >0.9 |  |
| 50-59 yrs | 0.22 | 0.836 | -1.4, 1.9 | 0.8 |  |
| 60-69 yrs | 0.64 | 0.875 | -1.1, 2.4 | 0.5 |  |
| 70-79 yrs | -0.69 | 1.24 | -3.2, 1.8 | 0.6 |  |
| 80+ | 2.1 | 1.72 | -1.3, 5.6 | 0.2 |  |
| Obesity |  |  |  |  | 0.07 |
| Yes | — | — | — |  |  |
| No | 0.14 | 0.080 | -0.01, 0.30 | 0.074 |  |
| Satisfaction: Life in general |  |  |  |  | >0.9 |
| Scale value 1 to 3 | — | — | — |  |  |
| Scale value 4 to 7 | 0.06 | 0.313 | -0.56, 0.68 | 0.8 |  |
| Scale value 8 to 10 | 0.12 | 0.311 | -0.50, 0.74 | 0.7 |  |
| Health risk due to climate change |  |  |  |  | 0.214 |
| Scale value 1 to 3 | — | — | — |  |  |
| Scale value 4 to 7 | 0.36 | 0.210 | -0.06, 0.78 | 0.089 |  |
| Scale value 8 to 10 | 0.28 | 0.287 | -0.29, 0.85 | 0.3 |  |
| Smoking |  |  |  |  | <0.001 |
| Daily smoking | — | — | — |  |  |
| Occasional smoking | 0.20 | 0.129 | -0.05, 0.46 | 0.12 |  |
| Non-smoker | 0.37 | 0.085 | 0.20, 0.54 | <0.001 |  |
| Sport |  |  |  |  | 0.411 |
| No sporting activities | — | — | — |  |  |
| Less than 1 hour per week | -0.32 | 0.237 | -0.79, 0.15 | 0.2 |  |
| 1 to less than 2 hours per week | -0.27 | 0.215 | -0.70, 0.16 | 0.2 |  |
| 2 to less than 4 hours per week | -0.40 | 0.240 | -0.87, 0.08 | 0.10 |  |
| 4 hours per week and more | -0.40 | 0.208 | -0.81, 0.02 | 0.060 |  |
| Regions |  |  |  |  | 0.1 |
| Northeast | — | — | — |  |  |
| Northwest | 0.01 | 0.371 | -0.73, 0.75 | >0.9 |  |
| Central-East | 0.20 | 0.447 | -0.69, 1.1 | 0.6 |  |
| Central-West | 0.67 | 0.359 | -0.05, 1.4 | 0.066 |  |
| South | 0.22 | 0.357 | -0.49, 0.93 | 0.5 |  |
| Country of birth |  |  |  |  | 0.396 |
| In Germany (within current borders) | — | — | — |  |  |
| In another country | -0.24 | 0.283 | -0.81, 0.32 | 0.4 |  |
| German nationality |  |  |  |  | 0.124 |
| Yes | — | — | — |  |  |
| No | -0.55 | 0.356 | -1.3, 0.16 | 0.13 |  |
| Education: ISCED (2011) |  |  |  |  | 0.084 |
| Low | — | — | — |  |  |
| Medium | -0.28 | 0.167 | -0.61, 0.06 | 0.10 |  |
| High | 0.00 | 0.211 | -0.42, 0.42 | >0.9 |  |
| Current living situation |  |  |  |  | 0.141 |
| Full-time employed | — | — | — |  |  |
| Part-time employed | -0.23 | 0.156 | -0.54, 0.08 | 0.15 |  |
| Unemployed | -0.29 | 0.194 | -0.68, 0.09 | 0.14 |  |
| Retired or early retired | 0.19 | 0.151 | -0.11, 0.49 | 0.2 |  |
| Not employed for other reasons (student, volunteer service, homemaker) | 0.02 | 0.154 | -0.28, 0.33 | 0.9 |  |
| Self-rated health |  |  |  |  | 0.666 |
| Very good/good/fair | — | — | — |  |  |
| Bad/very bad | -0.23 | 0.537 | -1.3, 0.84 | 0.7 |  |
| Sausage products |  |  |  |  | 0.39 |
| Daily or several times a day | — | — | — |  |  |
| 4 to 6 times per week | -0.34 | 0.389 | -1.1, 0.43 | 0.4 |  |
| 1 to 3 times per week | 0.00 | 0.341 | -0.68, 0.68 | >0.9 |  |
| Less than once per week | 0.27 | 0.357 | -0.44, 0.98 | 0.5 |  |
| Never | 0.13 | 0.456 | -0.78, 1.0 | 0.8 |  |
| Red meat |  |  |  |  | 0.564 |
| Daily or several times a day | — | — | — |  |  |
| 4 to 6 times per week | 0.37 | 0.338 | -0.31, 1.0 | 0.3 |  |
| 1 to 3 times per week | 0.52 | 0.317 | -0.11, 1.1 | 0.11 |  |
| Less than once per week | 0.46 | 0.334 | -0.20, 1.1 | 0.2 |  |
| Never | 0.56 | 0.409 | -0.25, 1.4 | 0.2 |  |
| Interview type |  |  |  |  | 0.074 |
| Computer Assisted Web Interview | — | — | — |  |  |
| Paper and Pencil Interview | -0.51 | 0.284 | -1.1, 0.06 | 0.078 |  |
| BIK community size (categorized) |  |  |  |  | 0.344 |
| BIK region <20,000 inhabitants | — | — | — |  |  |
| BIK region 20,000 to <50,000 inhabitants OR surroundings 50,000 to <500,000 inhabitants | -0.30 | 0.300 | -0.89, 0.30 | 0.3 |  |
| Core city 50,000 to <500,000 inhabitants OR surroundings 500,000+ inhabitants | 0.02 | 0.303 | -0.58, 0.62 | >0.9 |  |
| Core city 500,000+ inhabitants | -0.35 | 0.321 | -0.99, 0.29 | 0.3 |  |
| Waited for medical examination date in the last 12 months |  |  |  |  | 0.492 |
| Yes | — | — | — |  |  |
| No | 0.18 | 0.152 | -0.12, 0.48 | 0.2 |  |
| No need for examination or treatment | 0.08 | 0.261 | -0.44, 0.60 | 0.8 |  |
| Education: CASMIN |  |  |  |  | 0.255 |
| Low | — | — | — |  |  |
| Medium | 0.67 | 0.510 | -0.35, 1.7 | 0.2 |  |
| High | 0.97 | 0.612 | -0.25, 2.2 | 0.12 |  |
| Paying attention to health |  |  |  |  | 0.047 |
| Not at all/less strong/moderate | — | — | — |  |  |
| Strong/very strong | -0.25 | 0.127 | -0.50, 0.00 | 0.051 |  |
| Foreign nationality |  |  |  |  | 0.574 |
| Foreign national | — | — | — |  |  |
| Not foreign national | -0.11 | 0.197 | -0.50, 0.28 | 0.6 |  |
| Household size |  |  |  |  | 0.642 |
| Single-person household | — | — | — |  |  |
| Multi-person household | -0.07 | 0.152 | -0.37, 0.23 | 0.6 |  |
| Age \* Self-rated health |  |  |  |  | 0.199 |
| 30-39 yrs \* Bad/very bad | 1.1 | 0.689 | -0.29, 2.5 | 0.12 |  |
| 40-49 yrs \* Bad/very bad | 0.25 | 0.840 | -1.4, 1.9 | 0.8 |  |
| 50-59 yrs \* Bad/very bad | -0.31 | 0.589 | -1.5, 0.87 | 0.6 |  |
| 60-69 yrs \* Bad/very bad | -0.08 | 0.606 | -1.3, 1.1 | 0.9 |  |
| 70-79 yrs \* Bad/very bad | -0.07 | 0.611 | -1.3, 1.2 | >0.9 |  |
| 80+ \* Bad/very bad | 0.16 | 0.646 | -1.1, 1.4 | 0.8 |  |
| Age \* Satisfaction: Life in general |  |  |  |  | 0.184 |
| 30-39 yrs \* Scale value 4 to 7 | -0.10 | 0.378 | -0.85, 0.65 | 0.8 |  |
| 40-49 yrs \* Scale value 4 to 7 | -0.85 | 0.371 | -1.6, -0.11 | 0.025 |  |
| 50-59 yrs \* Scale value 4 to 7 | 0.17 | 0.355 | -0.54, 0.87 | 0.6 |  |
| 60-69 yrs \* Scale value 4 to 7 | -0.17 | 0.428 | -1.0, 0.68 | 0.7 |  |
| 70-79 yrs \* Scale value 4 to 7 | 0.49 | 0.483 | -0.47, 1.5 | 0.3 |  |
| 80+ \* Scale value 4 to 7 | 0.44 | 0.517 | -0.59, 1.5 | 0.4 |  |
| 30-39 yrs \* Scale value 8 to 10 | 0.23 | 0.382 | -0.53, 0.99 | 0.6 |  |
| 40-49 yrs \* Scale value 8 to 10 | -0.75 | 0.377 | -1.5, 0.00 | 0.050 |  |
| 50-59 yrs \* Scale value 8 to 10 | 0.19 | 0.363 | -0.54, 0.91 | 0.6 |  |
| 60-69 yrs \* Scale value 8 to 10 | 0.10 | 0.426 | -0.75, 0.94 | 0.8 |  |
| 70-79 yrs \* Scale value 8 to 10 | 0.43 | 0.493 | -0.55, 1.4 | 0.4 |  |
| 80+ \* Scale value 8 to 10 | 0.15 | 0.539 | -0.92, 1.2 | 0.8 |  |
| Age \* Health risk due to climate change |  |  |  |  | >0.9 |
| 30-39 yrs \* Scale value 4 to 7 | -0.20 | 0.217 | -0.63, 0.23 | 0.4 |  |
| 40-49 yrs \* Scale value 4 to 7 | -0.14 | 0.220 | -0.57, 0.30 | 0.5 |  |
| 50-59 yrs \* Scale value 4 to 7 | -0.17 | 0.209 | -0.59, 0.24 | 0.4 |  |
| 60-69 yrs \* Scale value 4 to 7 | -0.16 | 0.241 | -0.64, 0.33 | 0.5 |  |
| 70-79 yrs \* Scale value 4 to 7 | -0.14 | 0.303 | -0.74, 0.47 | 0.7 |  |
| 80+ \* Scale value 4 to 7 | 0.05 | 0.338 | -0.62, 0.73 | 0.9 |  |
| 30-39 yrs \* Scale value 8 to 10 | -0.04 | 0.250 | -0.54, 0.46 | 0.9 |  |
| 40-49 yrs \* Scale value 8 to 10 | -0.03 | 0.305 | -0.63, 0.58 | >0.9 |  |
| 50-59 yrs \* Scale value 8 to 10 | -0.16 | 0.294 | -0.75, 0.43 | 0.6 |  |
| 60-69 yrs \* Scale value 8 to 10 | -0.19 | 0.324 | -0.83, 0.46 | 0.6 |  |
| 70-79 yrs \* Scale value 8 to 10 | -0.14 | 0.389 | -0.92, 0.63 | 0.7 |  |
| 80+ \* Scale value 8 to 10 | -0.61 | 0.468 | -1.5, 0.32 | 0.2 |  |
| Age \* Sausage products |  |  |  |  | 0.33 |
| 30-39 yrs \* 4 to 6 times per week | 0.27 | 0.352 | -0.44, 0.97 | 0.5 |  |
| 40-49 yrs \* 4 to 6 times per week | 0.47 | 0.371 | -0.27, 1.2 | 0.2 |  |
| 50-59 yrs \* 4 to 6 times per week | 0.21 | 0.381 | -0.55, 0.97 | 0.6 |  |
| 60-69 yrs \* 4 to 6 times per week | 0.38 | 0.449 | -0.52, 1.3 | 0.4 |  |
| 70-79 yrs \* 4 to 6 times per week | 0.53 | 0.528 | -0.52, 1.6 | 0.3 |  |
| 80+ \* 4 to 6 times per week | 0.32 | 0.609 | -0.89, 1.5 | 0.6 |  |
| 30-39 yrs \* 1 to 3 times per week | 0.59 | 0.358 | -0.12, 1.3 | 0.10 |  |
| 40-49 yrs \* 1 to 3 times per week | 0.44 | 0.358 | -0.28, 1.1 | 0.2 |  |
| 50-59 yrs \* 1 to 3 times per week | 0.18 | 0.352 | -0.52, 0.88 | 0.6 |  |
| 60-69 yrs \* 1 to 3 times per week | -0.09 | 0.395 | -0.88, 0.69 | 0.8 |  |
| 70-79 yrs \* 1 to 3 times per week | 0.56 | 0.451 | -0.34, 1.5 | 0.2 |  |
| 80+ \* 1 to 3 times per week | 0.30 | 0.520 | -0.73, 1.3 | 0.6 |  |
| 30-39 yrs \* Less than once per week | 0.68 | 0.359 | -0.03, 1.4 | 0.060 |  |
| 40-49 yrs \* Less than once per week | 0.27 | 0.380 | -0.49, 1.0 | 0.5 |  |
| 50-59 yrs \* Less than once per week | 0.28 | 0.358 | -0.43, 1.0 | 0.4 |  |
| 60-69 yrs \* Less than once per week | 0.32 | 0.426 | -0.53, 1.2 | 0.5 |  |
| 70-79 yrs \* Less than once per week | 0.72 | 0.501 | -0.27, 1.7 | 0.2 |  |
| 80+ \* Less than once per week | -0.55 | 0.566 | -1.7, 0.57 | 0.3 |  |
| 30-39 yrs \* Never | 0.72 | 0.491 | -0.26, 1.7 | 0.15 |  |
| 40-49 yrs \* Never | 0.14 | 0.503 | -0.86, 1.1 | 0.8 |  |
| 50-59 yrs \* Never | 0.06 | 0.515 | -0.96, 1.1 | >0.9 |  |
| 60-69 yrs \* Never | -0.17 | 0.589 | -1.3, 1.0 | 0.8 |  |
| 70-79 yrs \* Never | 0.18 | 0.781 | -1.4, 1.7 | 0.8 |  |
| 80+ \* Never | -0.48 | 0.763 | -2.0, 1.0 | 0.5 |  |
| Age \* Red meat |  |  |  |  | 0.296 |
| 30-39 yrs \* 4 to 6 times per week | -0.34 | 0.532 | -1.4, 0.72 | 0.5 |  |
| 40-49 yrs \* 4 to 6 times per week | -0.19 | 0.545 | -1.3, 0.89 | 0.7 |  |
| 50-59 yrs \* 4 to 6 times per week | -0.60 | 0.670 | -1.9, 0.74 | 0.4 |  |
| 60-69 yrs \* 4 to 6 times per week | -0.33 | 0.739 | -1.8, 1.1 | 0.7 |  |
| 70-79 yrs \* 4 to 6 times per week | -0.20 | 1.00 | -2.2, 1.8 | 0.8 |  |
| 80+ \* 4 to 6 times per week | -3.0 | 1.43 | -5.9, -0.17 | 0.038 |  |
| 30-39 yrs \* 1 to 3 times per week | -0.83 | 0.502 | -1.8, 0.17 | 0.10 |  |
| 40-49 yrs \* 1 to 3 times per week | -0.50 | 0.531 | -1.6, 0.55 | 0.3 |  |
| 50-59 yrs \* 1 to 3 times per week | -1.2 | 0.659 | -2.5, 0.11 | 0.072 |  |
| 60-69 yrs \* 1 to 3 times per week | -0.54 | 0.738 | -2.0, 0.93 | 0.5 |  |
| 70-79 yrs \* 1 to 3 times per week | -0.07 | 0.914 | -1.9, 1.8 | >0.9 |  |
| 80+ \* 1 to 3 times per week | -3.8 | 1.40 | -6.6, -1.1 | 0.008 |  |
| 30-39 yrs \* Less than once per week | -0.86 | 0.507 | -1.9, 0.15 | 0.094 |  |
| 40-49 yrs \* Less than once per week | -0.03 | 0.538 | -1.1, 1.0 | >0.9 |  |
| 50-59 yrs \* Less than once per week | -0.77 | 0.642 | -2.1, 0.51 | 0.2 |  |
| 60-69 yrs \* Less than once per week | -0.33 | 0.784 | -1.9, 1.2 | 0.7 |  |
| 70-79 yrs \* Less than once per week | 0.04 | 0.943 | -1.8, 1.9 | >0.9 |  |
| 80+ \* Less than once per week | -3.5 | 1.43 | -6.4, -0.66 | 0.016 |  |
| 30-39 yrs \* Never | -1.1 | 0.631 | -2.3, 0.17 | 0.090 |  |
| 40-49 yrs \* Never | -0.46 | 0.608 | -1.7, 0.75 | 0.4 |  |
| 50-59 yrs \* Never | -1.5 | 0.698 | -2.8, -0.06 | 0.041 |  |
| 60-69 yrs \* Never | -0.88 | 0.873 | -2.6, 0.86 | 0.3 |  |
| 70-79 yrs \* Never | -0.07 | 1.02 | -2.1, 2.0 | >0.9 |  |
| 80+ \* Never | -3.8 | 1.49 | -6.8, -0.86 | 0.012 |  |
| Age \* Sport |  |  |  |  | 0.046 |
| 30-39 yrs \* Less than 1 hour per week | 0.41 | 0.307 | -0.20, 1.0 | 0.2 |  |
| 40-49 yrs \* Less than 1 hour per week | 0.44 | 0.296 | -0.15, 1.0 | 0.15 |  |
| 50-59 yrs \* Less than 1 hour per week | 0.49 | 0.299 | -0.11, 1.1 | 0.11 |  |
| 60-69 yrs \* Less than 1 hour per week | 0.05 | 0.347 | -0.64, 0.74 | 0.9 |  |
| 70-79 yrs \* Less than 1 hour per week | 0.52 | 0.439 | -0.35, 1.4 | 0.2 |  |
| 80+ \* Less than 1 hour per week | 1.4 | 0.472 | 0.43, 2.3 | 0.005 |  |
| 30-39 yrs \* 1 to less than 2 hours per week | 0.38 | 0.295 | -0.20, 0.97 | 0.2 |  |
| 40-49 yrs \* 1 to less than 2 hours per week | 0.62 | 0.307 | 0.01, 1.2 | 0.046 |  |
| 50-59 yrs \* 1 to less than 2 hours per week | 0.41 | 0.269 | -0.13, 0.95 | 0.13 |  |
| 60-69 yrs \* 1 to less than 2 hours per week | 0.39 | 0.298 | -0.20, 0.99 | 0.2 |  |
| 70-79 yrs \* 1 to less than 2 hours per week | 0.33 | 0.378 | -0.42, 1.1 | 0.4 |  |
| 80+ \* 1 to less than 2 hours per week | 1.0 | 0.366 | 0.32, 1.8 | 0.005 |  |
| 30-39 yrs \* 2 to less than 4 hours per week | 0.27 | 0.331 | -0.38, 0.93 | 0.4 |  |
| 40-49 yrs \* 2 to less than 4 hours per week | 0.70 | 0.323 | 0.05, 1.3 | 0.034 |  |
| 50-59 yrs \* 2 to less than 4 hours per week | 0.31 | 0.306 | -0.30, 0.92 | 0.3 |  |
| 60-69 yrs \* 2 to less than 4 hours per week | 0.28 | 0.377 | -0.47, 1.0 | 0.5 |  |
| 70-79 yrs \* 2 to less than 4 hours per week | 0.40 | 0.406 | -0.41, 1.2 | 0.3 |  |
| 80+ \* 2 to less than 4 hours per week | 1.5 | 0.443 | 0.61, 2.4 | 0.001 |  |
| 30-39 yrs \* 4 hours per week and more | 0.32 | 0.322 | -0.32, 0.96 | 0.3 |  |
| 40-49 yrs \* 4 hours per week and more | 0.98 | 0.351 | 0.28, 1.7 | 0.007 |  |
| 50-59 yrs \* 4 hours per week and more | 0.28 | 0.327 | -0.38, 0.93 | 0.4 |  |
| 60-69 yrs \* 4 hours per week and more | 0.63 | 0.383 | -0.13, 1.4 | 0.10 |  |
| 70-79 yrs \* 4 hours per week and more | 0.38 | 0.444 | -0.51, 1.3 | 0.4 |  |
| 80+ \* 4 hours per week and more | 2.5 | 0.651 | 1.2, 3.8 | <0.001 |  |
| Age \* Interview type |  |  |  |  | 0.171 |
| 30-39 yrs \* Paper and Pencil Interview | 0.96 | 0.392 | 0.18, 1.7 | 0.017 |  |
| 40-49 yrs \* Paper and Pencil Interview | 1.1 | 0.430 | 0.20, 1.9 | 0.016 |  |
| 50-59 yrs \* Paper and Pencil Interview | 0.70 | 0.354 | 0.00, 1.4 | 0.051 |  |
| 60-69 yrs \* Paper and Pencil Interview | 0.60 | 0.364 | -0.12, 1.3 | 0.10 |  |
| 70-79 yrs \* Paper and Pencil Interview | 0.74 | 0.367 | 0.01, 1.5 | 0.046 |  |
| 80+ \* Paper and Pencil Interview | 1.0 | 0.466 | 0.08, 1.9 | 0.033 |  |
| Age \* BIK community size (categorized) |  |  |  |  | 0.179 |
| 30-39 yrs \* BIK region 20,000 to <50,000 inhabitants OR surroundings 50,000 to <500,000 inhabitants | 0.56 | 0.286 | -0.01, 1.1 | 0.055 |  |
| 40-49 yrs \* BIK region 20,000 to <50,000 inhabitants OR surroundings 50,000 to <500,000 inhabitants | 0.34 | 0.356 | -0.37, 1.1 | 0.3 |  |
| 50-59 yrs \* BIK region 20,000 to <50,000 inhabitants OR surroundings 50,000 to <500,000 inhabitants | 0.56 | 0.298 | -0.03, 1.2 | 0.065 |  |
| 60-69 yrs \* BIK region 20,000 to <50,000 inhabitants OR surroundings 50,000 to <500,000 inhabitants | 0.45 | 0.381 | -0.31, 1.2 | 0.2 |  |
| 70-79 yrs \* BIK region 20,000 to <50,000 inhabitants OR surroundings 50,000 to <500,000 inhabitants | 0.71 | 0.415 | -0.11, 1.5 | 0.089 |  |
| 80+ \* BIK region 20,000 to <50,000 inhabitants OR surroundings 50,000 to <500,000 inhabitants | 0.01 | 0.447 | -0.88, 0.90 | >0.9 |  |
| 30-39 yrs \* Core city 50,000 to <500,000 inhabitants OR surroundings 500,000+ inhabitants | 0.23 | 0.298 | -0.37, 0.82 | 0.5 |  |
| 40-49 yrs \* Core city 50,000 to <500,000 inhabitants OR surroundings 500,000+ inhabitants | 0.35 | 0.372 | -0.39, 1.1 | 0.3 |  |
| 50-59 yrs \* Core city 50,000 to <500,000 inhabitants OR surroundings 500,000+ inhabitants | 0.27 | 0.318 | -0.36, 0.91 | 0.4 |  |
| 60-69 yrs \* Core city 50,000 to <500,000 inhabitants OR surroundings 500,000+ inhabitants | 0.05 | 0.396 | -0.74, 0.83 | >0.9 |  |
| 70-79 yrs \* Core city 50,000 to <500,000 inhabitants OR surroundings 500,000+ inhabitants | 0.02 | 0.404 | -0.78, 0.82 | >0.9 |  |
| 80+ \* Core city 50,000 to <500,000 inhabitants OR surroundings 500,000+ inhabitants | 0.17 | 0.452 | -0.73, 1.1 | 0.7 |  |
| 30-39 yrs \* Core city 500,000+ inhabitants | 0.39 | 0.298 | -0.20, 0.98 | 0.2 |  |
| 40-49 yrs \* Core city 500,000+ inhabitants | 0.65 | 0.360 | -0.07, 1.4 | 0.075 |  |
| 50-59 yrs \* Core city 500,000+ inhabitants | 0.29 | 0.317 | -0.34, 0.92 | 0.4 |  |
| 60-69 yrs \* Core city 500,000+ inhabitants | 0.48 | 0.385 | -0.28, 1.2 | 0.2 |  |
| 70-79 yrs \* Core city 500,000+ inhabitants | 0.23 | 0.422 | -0.61, 1.1 | 0.6 |  |
| 80+ \* Core city 500,000+ inhabitants | 0.95 | 0.483 | -0.01, 1.9 | 0.052 |  |
| Age \* Regions |  |  |  |  | 0.555 |
| 30-39 yrs \* Northwest | 0.23 | 0.282 | -0.33, 0.79 | 0.4 |  |
| 40-49 yrs \* Northwest | 0.42 | 0.365 | -0.31, 1.1 | 0.3 |  |
| 50-59 yrs \* Northwest | 0.55 | 0.317 | -0.08, 1.2 | 0.085 |  |
| 60-69 yrs \* Northwest | 0.51 | 0.363 | -0.21, 1.2 | 0.2 |  |
| 70-79 yrs \* Northwest | 0.17 | 0.475 | -0.77, 1.1 | 0.7 |  |
| 80+ \* Northwest | 0.45 | 0.511 | -0.57, 1.5 | 0.4 |  |
| 30-39 yrs \* Central-East | 0.14 | 0.373 | -0.60, 0.89 | 0.7 |  |
| 40-49 yrs \* Central-East | 0.51 | 0.400 | -0.28, 1.3 | 0.2 |  |
| 50-59 yrs \* Central-East | -0.12 | 0.359 | -0.84, 0.59 | 0.7 |  |
| 60-69 yrs \* Central-East | 0.50 | 0.480 | -0.46, 1.5 | 0.3 |  |
| 70-79 yrs \* Central-East | -0.34 | 0.563 | -1.5, 0.78 | 0.5 |  |
| 80+ \* Central-East | 0.64 | 0.653 | -0.66, 1.9 | 0.3 |  |
| 30-39 yrs \* Central-West | -0.02 | 0.264 | -0.55, 0.51 | >0.9 |  |
| 40-49 yrs \* Central-West | 0.25 | 0.305 | -0.36, 0.85 | 0.4 |  |
| 50-59 yrs \* Central-West | 0.29 | 0.277 | -0.26, 0.84 | 0.3 |  |
| 60-69 yrs \* Central-West | 0.22 | 0.344 | -0.47, 0.91 | 0.5 |  |
| 70-79 yrs \* Central-West | -0.04 | 0.441 | -0.92, 0.84 | >0.9 |  |
| 80+ \* Central-West | -0.25 | 0.480 | -1.2, 0.71 | 0.6 |  |
| 30-39 yrs \* South | 0.08 | 0.278 | -0.47, 0.64 | 0.8 |  |
| 40-49 yrs \* South | 0.07 | 0.343 | -0.61, 0.75 | 0.8 |  |
| 50-59 yrs \* South | 0.23 | 0.303 | -0.37, 0.84 | 0.4 |  |
| 60-69 yrs \* South | 0.25 | 0.335 | -0.42, 0.92 | 0.5 |  |
| 70-79 yrs \* South | 0.79 | 0.492 | -0.19, 1.8 | 0.11 |  |
| 80+ \* South | 0.22 | 0.539 | -0.85, 1.3 | 0.7 |  |
| Age \* Country of birth |  |  |  |  | 0.235 |
| 30-39 yrs \* In another country | 0.29 | 0.399 | -0.50, 1.1 | 0.5 |  |
| 40-49 yrs \* In another country | -0.20 | 0.383 | -0.96, 0.56 | 0.6 |  |
| 50-59 yrs \* In another country | -0.32 | 0.394 | -1.1, 0.46 | 0.4 |  |
| 60-69 yrs \* In another country | -0.71 | 0.452 | -1.6, 0.20 | 0.12 |  |
| 70-79 yrs \* In another country | -0.74 | 0.446 | -1.6, 0.14 | 0.10 |  |
| 80+ \* In another country | -0.12 | 0.473 | -1.1, 0.82 | 0.8 |  |
| Age \* German nationality |  |  |  |  | 0.574 |
| 30-39 yrs \* No | -0.33 | 0.487 | -1.3, 0.64 | 0.5 |  |
| 40-49 yrs \* No | -0.47 | 0.490 | -1.4, 0.51 | 0.3 |  |
| 50-59 yrs \* No | 0.16 | 0.523 | -0.88, 1.2 | 0.8 |  |
| 60-69 yrs \* No | 0.62 | 0.653 | -0.69, 1.9 | 0.3 |  |
| 70-79 yrs \* No | 0.24 | 0.652 | -1.1, 1.5 | 0.7 |  |
| 80+ \* No | 1.0 | 0.959 | -0.91, 2.9 | 0.3 |  |
| Age \* Education: ISCED (2011) |  |  |  |  | 0.142 |
| 30-39 yrs \* Medium | 0.59 | 0.346 | -0.10, 1.3 | 0.094 |  |
| 40-49 yrs \* Medium | 0.45 | 0.345 | -0.23, 1.1 | 0.2 |  |
| 50-59 yrs \* Medium | 0.73 | 0.326 | 0.08, 1.4 | 0.028 |  |
| 60-69 yrs \* Medium | 0.48 | 0.353 | -0.22, 1.2 | 0.2 |  |
| 70-79 yrs \* Medium | 0.68 | 0.406 | -0.13, 1.5 | 0.10 |  |
| 80+ \* Medium | 0.84 | 0.336 | 0.18, 1.5 | 0.014 |  |
| 30-39 yrs \* High | 0.20 | 0.367 | -0.53, 0.93 | 0.6 |  |
| 40-49 yrs \* High | 0.23 | 0.355 | -0.48, 0.94 | 0.5 |  |
| 50-59 yrs \* High | 0.34 | 0.359 | -0.37, 1.1 | 0.3 |  |
| 60-69 yrs \* High | 0.45 | 0.393 | -0.33, 1.2 | 0.3 |  |
| 70-79 yrs \* High | 0.53 | 0.449 | -0.36, 1.4 | 0.2 |  |
| 80+ \* High | 0.70 | 0.401 | -0.10, 1.5 | 0.087 |  |
| Sex \* Red meat |  |  |  |  | 0.359 |
| Female \* 4 to 6 times per week | 0.68 | 0.441 | -0.20, 1.6 | 0.13 |  |
| Female \* 1 to 3 times per week | 0.64 | 0.414 | -0.18, 1.5 | 0.12 |  |
| Female \* Less than once per week | 0.55 | 0.419 | -0.28, 1.4 | 0.2 |  |
| Female \* Never | 0.83 | 0.445 | -0.06, 1.7 | 0.066 |  |
| Sex \* Sport |  |  |  |  | 0.347 |
| Female \* Less than 1 hour per week | -0.06 | 0.181 | -0.42, 0.30 | 0.8 |  |
| Female \* 1 to less than 2 hours per week | 0.16 | 0.178 | -0.19, 0.52 | 0.4 |  |
| Female \* 2 to less than 4 hours per week | 0.31 | 0.193 | -0.07, 0.69 | 0.11 |  |
| Female \* 4 hours per week and more | 0.06 | 0.204 | -0.34, 0.47 | 0.8 |  |
| Sex \* Waited for medical examination date in the last 12 months |  |  |  |  | 0.155 |
| Female \* No | 0.01 | 0.126 | -0.24, 0.26 | >0.9 |  |
| Female \* No need for examination or treatment | 0.37 | 0.204 | -0.04, 0.78 | 0.074 |  |
| Sex \* Regions |  |  |  |  | 0.051 |
| Female \* Northwest | 0.07 | 0.191 | -0.31, 0.45 | 0.7 |  |
| Female \* Central-East | 0.42 | 0.214 | 0.00, 0.85 | 0.052 |  |
| Female \* Central-West | -0.11 | 0.170 | -0.45, 0.22 | 0.5 |  |
| Female \* South | 0.21 | 0.172 | -0.13, 0.55 | 0.2 |  |
| Sex \* Current living situation |  |  |  |  | 0.62 |
| Female \* Part-time employed | -0.01 | 0.215 | -0.44, 0.42 | >0.9 |  |
| Female \* Unemployed | -0.35 | 0.300 | -0.95, 0.24 | 0.2 |  |
| Female \* Retired or early retired | -0.16 | 0.171 | -0.50, 0.18 | 0.4 |  |
| Female \* Not employed for other reasons (student, volunteer service, homemaker) | -0.20 | 0.204 | -0.61, 0.20 | 0.3 |  |
| Satisfaction: Life in general \* Education: CASMIN |  |  |  |  | >0.9 |
| Scale value 4 to 7 \* Medium | 0.07 | 0.258 | -0.45, 0.58 | 0.8 |  |
| Scale value 8 to 10 \* Medium | -0.07 | 0.251 | -0.57, 0.43 | 0.8 |  |
| Scale value 4 to 7 \* High | 0.07 | 0.325 | -0.58, 0.71 | 0.8 |  |
| Scale value 8 to 10 \* High | -0.10 | 0.309 | -0.71, 0.52 | 0.8 |  |
| Health risk due to climate change \* Education: CASMIN |  |  |  |  | 0.786 |
| Scale value 4 to 7 \* Medium | -0.08 | 0.180 | -0.44, 0.28 | 0.6 |  |
| Scale value 8 to 10 \* Medium | -0.08 | 0.254 | -0.58, 0.43 | 0.8 |  |
| Scale value 4 to 7 \* High | -0.23 | 0.189 | -0.61, 0.14 | 0.2 |  |
| Scale value 8 to 10 \* High | -0.12 | 0.269 | -0.65, 0.42 | 0.7 |  |
| Education: CASMIN \* Paying attention to health |  |  |  |  | 0.134 |
| Medium \* Strong/very strong | 0.26 | 0.150 | -0.04, 0.56 | 0.089 |  |
| High \* Strong/very strong | 0.29 | 0.156 | -0.02, 0.60 | 0.069 |  |
| Sausage products \* Education: CASMIN |  |  |  |  | 0.166 |
| 4 to 6 times per week \* Medium | 0.24 | 0.314 | -0.39, 0.86 | 0.5 |  |
| 1 to 3 times per week \* Medium | -0.16 | 0.295 | -0.75, 0.42 | 0.6 |  |
| Less than once per week \* Medium | -0.49 | 0.312 | -1.1, 0.13 | 0.12 |  |
| Never \* Medium | 0.06 | 0.384 | -0.71, 0.82 | 0.9 |  |
| 4 to 6 times per week \* High | -0.10 | 0.353 | -0.80, 0.60 | 0.8 |  |
| 1 to 3 times per week \* High | -0.16 | 0.325 | -0.81, 0.49 | 0.6 |  |
| Less than once per week \* High | -0.56 | 0.342 | -1.2, 0.12 | 0.11 |  |
| Never \* High | -0.14 | 0.438 | -1.0, 0.73 | 0.7 |  |
| Waited for medical examination date in the last 12 months \* Education: CASMIN |  |  |  |  | >0.9 |
| No \* Medium | -0.10 | 0.156 | -0.42, 0.21 | 0.5 |  |
| No need for examination or treatment \* Medium | -0.02 | 0.266 | -0.55, 0.51 | >0.9 |  |
| No \* High | -0.07 | 0.186 | -0.44, 0.31 | 0.7 |  |
| No need for examination or treatment \* High | -0.08 | 0.307 | -0.70, 0.53 | 0.8 |  |
| Education: CASMIN \* Foreign nationality |  |  |  |  | 0.474 |
| Medium \* Not foreign national | -0.19 | 0.230 | -0.64, 0.27 | 0.4 |  |
| High \* Not foreign national | 0.06 | 0.250 | -0.44, 0.56 | 0.8 |  |
| BIK community size (categorized) \* Education: CASMIN |  |  |  |  | 0.722 |
| BIK region 20,000 to <50,000 inhabitants OR surroundings 50,000 to <500,000 inhabitants \* Medium | 0.19 | 0.238 | -0.29, 0.66 | 0.4 |  |
| Core city 50,000 to <500,000 inhabitants OR surroundings 500,000+ inhabitants \* Medium | 0.03 | 0.260 | -0.48, 0.55 | 0.9 |  |
| Core city 500,000+ inhabitants \* Medium | 0.37 | 0.274 | -0.18, 0.91 | 0.2 |  |
| BIK region 20,000 to <50,000 inhabitants OR surroundings 50,000 to <500,000 inhabitants \* High | -0.01 | 0.258 | -0.52, 0.51 | >0.9 |  |
| Core city 50,000 to <500,000 inhabitants OR surroundings 500,000+ inhabitants \* High | -0.15 | 0.273 | -0.69, 0.39 | 0.6 |  |
| Core city 500,000+ inhabitants \* High | 0.03 | 0.288 | -0.54, 0.61 | >0.9 |  |
| Regions \* Education: CASMIN |  |  |  |  | 0.091 |
| Northwest \* Medium | -0.27 | 0.293 | -0.85, 0.32 | 0.4 |  |
| Central-East \* Medium | -0.24 | 0.355 | -0.94, 0.47 | 0.5 |  |
| Central-West \* Medium | -0.56 | 0.288 | -1.1, 0.01 | 0.055 |  |
| South \* Medium | -0.25 | 0.306 | -0.86, 0.36 | 0.4 |  |
| Northwest \* High | -0.21 | 0.328 | -0.87, 0.44 | 0.5 |  |
| Central-East \* High | -0.30 | 0.426 | -1.1, 0.55 | 0.5 |  |
| Central-West \* High | -0.97 | 0.344 | -1.7, -0.28 | 0.006 |  |
| South \* High | -0.28 | 0.341 | -0.96, 0.40 | 0.4 |  |
| Education: CASMIN \* Household size |  |  |  |  | 0.554 |
| Medium \* Multi-person household | 0.04 | 0.173 | -0.31, 0.38 | 0.8 |  |
| High \* Multi-person household | 0.21 | 0.221 | -0.23, 0.65 | 0.3 |  |
| N.Obs | 11,863 |  |  |  |  |
| N.Cluster | 359 |  |  |  |  |
|  |  |  |  |  |  |
| --- | --- | --- | --- | --- | --- |
| Abbreviations: CI = Confidence Interval, OR = Odds Ratio, SE = Standard Error | | | | | |

### Quarter 4

| Characteristic | log(OR) | SE | 95% CI | p-value | p-value (global) |
| --- | --- | --- | --- | --- | --- |
| Sex |  |  |  |  | 0.108 |
| Male | — | — | — |  |  |
| Female | 0.82 | 0.510 | -0.80, 2.4 | 0.2 |  |
| Age |  |  |  |  | 0.104 |
| 16-29 yrs | — | — | — |  |  |
| 30-39 yrs | -0.94 | 0.850 | -3.7, 1.8 | 0.3 |  |
| 40-49 yrs | 0.14 | 0.933 | -2.8, 3.1 | 0.9 |  |
| 50-59 yrs | -0.38 | 0.788 | -2.9, 2.1 | 0.7 |  |
| 60-69 yrs | -2.5 | 0.947 | -5.5, 0.54 | 0.080 |  |
| 70-79 yrs | 0.55 | 1.32 | -3.6, 4.7 | 0.7 |  |
| 80+ | -0.99 | 1.30 | -5.1, 3.1 | 0.5 |  |
| Obesity |  |  |  |  | >0.9 |
| Yes | — | — | — |  |  |
| No | 0.00 | 0.149 | -0.48, 0.47 | >0.9 |  |
| Self-rated health |  |  |  |  | 0.045 |
| Very good/good/fair | — | — | — |  |  |
| Bad/very bad | -0.87 | 0.435 | -2.3, 0.51 | 0.14 |  |
| Chronic diseases |  |  |  |  | 0.334 |
| Yes | — | — | — |  |  |
| No | -0.12 | 0.128 | -0.53, 0.28 | 0.4 |  |
| Satisfaction: Life in general |  |  |  |  | 0.464 |
| Scale value 1 to 3 | — | — | — |  |  |
| Scale value 4 to 7 | -0.35 | 0.291 | -1.3, 0.58 | 0.3 |  |
| Scale value 8 to 10 | -0.37 | 0.327 | -1.4, 0.67 | 0.3 |  |
| Health risk due to climate change |  |  |  |  | 0.876 |
| Scale value 1 to 3 | — | — | — |  |  |
| Scale value 4 to 7 | 0.03 | 0.211 | -0.65, 0.70 | >0.9 |  |
| Scale value 8 to 10 | -0.09 | 0.250 | -0.88, 0.70 | 0.7 |  |
| Paying attention to health |  |  |  |  | 0.109 |
| Not at all/less strong/moderate | — | — | — |  |  |
| Strong/very strong | 0.31 | 0.194 | -0.31, 0.93 | 0.2 |  |
| Smoking |  |  |  |  | 0.002 |
| Daily smoking | — | — | — |  |  |
| Occasional smoking | 0.15 | 0.249 | -0.65, 0.94 | 0.6 |  |
| Non-smoker | 0.57 | 0.176 | 0.01, 1.1 | 0.047 |  |
| Red meat |  |  |  |  | >0.9 |
| Daily or several times a day | — | — | — |  |  |
| 4 to 6 times per week | 0.30 | 0.540 | -1.4, 2.0 | 0.6 |  |
| 1 to 3 times per week | 0.12 | 0.490 | -1.4, 1.7 | 0.8 |  |
| Less than once per week | 0.21 | 0.519 | -1.4, 1.9 | 0.7 |  |
| Never | 0.14 | 0.592 | -1.7, 2.0 | 0.8 |  |
| Sport |  |  |  |  | 0.58 |
| No sporting activities | — | — | — |  |  |
| Less than 1 hour per week | 0.18 | 0.286 | -0.73, 1.1 | 0.6 |  |
| 1 to less than 2 hours per week | 0.34 | 0.306 | -0.63, 1.3 | 0.3 |  |
| 2 to less than 4 hours per week | 0.49 | 0.312 | -0.51, 1.5 | 0.2 |  |
| 4 hours per week and more | 0.12 | 0.332 | -0.94, 1.2 | 0.7 |  |
| Waited for medical examination date in the last 12 months |  |  |  |  | 0.104 |
| Yes | — | — | — |  |  |
| No | 0.33 | 0.217 | -0.36, 1.0 | 0.2 |  |
| No need for examination or treatment | 0.58 | 0.313 | -0.42, 1.6 | 0.2 |  |
| Interview type |  |  |  |  | 0.058 |
| Computer Assisted Web Interview | — | — | — |  |  |
| Paper and Pencil Interview | 0.49 | 0.257 | -0.33, 1.3 | 0.2 |  |
| Regions |  |  |  |  | 0.619 |
| Northeast | — | — | — |  |  |
| Northwest | -0.09 | 0.316 | -1.1, 0.92 | 0.8 |  |
| Central-East | -0.19 | 0.389 | -1.4, 1.0 | 0.7 |  |
| Central-West | 0.09 | 0.302 | -0.87, 1.1 | 0.8 |  |
| South | 0.24 | 0.301 | -0.72, 1.2 | 0.5 |  |
| Country of birth |  |  |  |  | 0.338 |
| In Germany (within current borders) | — | — | — |  |  |
| In another country | -0.24 | 0.255 | -1.1, 0.57 | 0.4 |  |
| German nationality |  |  |  |  | <0.001 |
| Yes | — | — | — |  |  |
| No | -0.71 | 0.173 | -1.3, -0.16 | 0.026 |  |
| Education: ISCED (2011) |  |  |  |  | 0.529 |
| Low | — | — | — |  |  |
| Medium | 0.07 | 0.177 | -0.49, 0.64 | 0.7 |  |
| High | 0.25 | 0.233 | -0.49, 0.99 | 0.4 |  |
| Current living situation |  |  |  |  | 0.085 |
| Full-time employed | — | — | — |  |  |
| Part-time employed | -0.18 | 0.215 | -0.86, 0.51 | 0.5 |  |
| Unemployed | -0.26 | 0.253 | -1.1, 0.55 | 0.4 |  |
| Retired or early retired | 0.37 | 0.192 | -0.24, 0.98 | 0.15 |  |
| Not employed for other reasons (student, volunteer service, homemaker) | -0.03 | 0.227 | -0.75, 0.69 | >0.9 |  |
| Overweight |  |  |  |  | >0.9 |
| Yes | — | — | — |  |  |
| No | 0.01 | 0.127 | -0.39, 0.41 | >0.9 |  |
| Self-rated mental health |  |  |  |  | 0.212 |
| Excellent/very good/good | — | — | — |  |  |
| fair/poor | 0.20 | 0.156 | -0.30, 0.69 | 0.3 |  |
| Sausage products |  |  |  |  | 0.201 |
| Daily or several times a day | — | — | — |  |  |
| 4 to 6 times per week | -0.74 | 0.350 | -1.9, 0.37 | 0.12 |  |
| 1 to 3 times per week | -0.51 | 0.319 | -1.5, 0.50 | 0.2 |  |
| Less than once per week | -0.30 | 0.343 | -1.4, 0.79 | 0.4 |  |
| Never | -0.07 | 0.421 | -1.4, 1.3 | 0.9 |  |
| BIK community size (categorized) |  |  |  |  | 0.511 |
| BIK region <20,000 inhabitants | — | — | — |  |  |
| BIK region 20,000 to <50,000 inhabitants OR surroundings 50,000 to <500,000 inhabitants | -0.19 | 0.312 | -1.2, 0.80 | 0.6 |  |
| Core city 50,000 to <500,000 inhabitants OR surroundings 500,000+ inhabitants | -0.28 | 0.316 | -1.3, 0.72 | 0.4 |  |
| Core city 500,000+ inhabitants | -0.45 | 0.324 | -1.5, 0.58 | 0.3 |  |
| Household size |  |  |  |  | 0.843 |
| Single-person household | — | — | — |  |  |
| Multi-person household | 0.05 | 0.263 | -0.79, 0.89 | 0.9 |  |
| Education: CASMIN |  |  |  |  | 0.213 |
| Low | — | — | — |  |  |
| Medium | -0.73 | 0.578 | -2.6, 1.1 | 0.3 |  |
| High | 0.39 | 0.760 | -2.0, 2.8 | 0.6 |  |
| Foreign nationality |  |  |  |  | 0.578 |
| Foreign national | — | — | — |  |  |
| Not foreign national | -0.11 | 0.190 | -0.71, 0.50 | 0.6 |  |
| Age \* Overweight |  |  |  |  | 0.341 |
| 30-39 yrs \* No | -0.16 | 0.180 | -0.73, 0.41 | 0.4 |  |
| 40-49 yrs \* No | -0.04 | 0.200 | -0.68, 0.59 | 0.8 |  |
| 50-59 yrs \* No | 0.10 | 0.196 | -0.53, 0.72 | 0.7 |  |
| 60-69 yrs \* No | 0.36 | 0.228 | -0.36, 1.1 | 0.2 |  |
| 70-79 yrs \* No | -0.19 | 0.270 | -1.0, 0.68 | 0.5 |  |
| 80+ \* No | 0.01 | 0.280 | -0.88, 0.90 | >0.9 |  |
| Age \* Self-rated health |  |  |  |  | 0.005 |
| 30-39 yrs \* Bad/very bad | 1.3 | 0.620 | -0.69, 3.3 | 0.13 |  |
| 40-49 yrs \* Bad/very bad | 1.5 | 0.649 | -0.57, 3.6 | 0.10 |  |
| 50-59 yrs \* Bad/very bad | 0.77 | 0.502 | -0.82, 2.4 | 0.2 |  |
| 60-69 yrs \* Bad/very bad | 0.61 | 0.509 | -1.0, 2.2 | 0.3 |  |
| 70-79 yrs \* Bad/very bad | -0.33 | 0.548 | -2.1, 1.4 | 0.6 |  |
| 80+ \* Bad/very bad | 0.01 | 0.568 | -1.8, 1.8 | >0.9 |  |
| Age \* Chronic diseases |  |  |  |  | 0.383 |
| 30-39 yrs \* No | -0.16 | 0.209 | -0.82, 0.51 | 0.5 |  |
| 40-49 yrs \* No | 0.05 | 0.201 | -0.59, 0.69 | 0.8 |  |
| 50-59 yrs \* No | -0.04 | 0.176 | -0.60, 0.52 | 0.8 |  |
| 60-69 yrs \* No | -0.36 | 0.239 | -1.1, 0.40 | 0.2 |  |
| 70-79 yrs \* No | 0.00 | 0.277 | -0.88, 0.88 | >0.9 |  |
| 80+ \* No | -0.57 | 0.302 | -1.5, 0.39 | 0.2 |  |
| Age \* Satisfaction: Life in general |  |  |  |  | 0.047 |
| 30-39 yrs \* Scale value 4 to 7 | 0.53 | 0.410 | -0.78, 1.8 | 0.3 |  |
| 40-49 yrs \* Scale value 4 to 7 | -0.05 | 0.371 | -1.2, 1.1 | 0.9 |  |
| 50-59 yrs \* Scale value 4 to 7 | 0.43 | 0.338 | -0.65, 1.5 | 0.3 |  |
| 60-69 yrs \* Scale value 4 to 7 | 1.0 | 0.355 | -0.10, 2.2 | 0.062 |  |
| 70-79 yrs \* Scale value 4 to 7 | -0.30 | 0.495 | -1.9, 1.3 | 0.6 |  |
| 80+ \* Scale value 4 to 7 | 0.48 | 0.563 | -1.3, 2.3 | 0.5 |  |
| 30-39 yrs \* Scale value 8 to 10 | 0.44 | 0.449 | -0.98, 1.9 | 0.4 |  |
| 40-49 yrs \* Scale value 8 to 10 | 0.05 | 0.436 | -1.3, 1.4 | >0.9 |  |
| 50-59 yrs \* Scale value 8 to 10 | 0.55 | 0.363 | -0.61, 1.7 | 0.2 |  |
| 60-69 yrs \* Scale value 8 to 10 | 1.5 | 0.407 | 0.18, 2.8 | 0.036 |  |
| 70-79 yrs \* Scale value 8 to 10 | -0.20 | 0.537 | -1.9, 1.5 | 0.7 |  |
| 80+ \* Scale value 8 to 10 | 0.77 | 0.594 | -1.1, 2.7 | 0.3 |  |
| Age \* Health risk due to climate change |  |  |  |  | 0.054 |
| 30-39 yrs \* Scale value 4 to 7 | 0.28 | 0.193 | -0.33, 0.89 | 0.2 |  |
| 40-49 yrs \* Scale value 4 to 7 | 0.22 | 0.226 | -0.50, 0.94 | 0.4 |  |
| 50-59 yrs \* Scale value 4 to 7 | 0.04 | 0.208 | -0.62, 0.71 | 0.9 |  |
| 60-69 yrs \* Scale value 4 to 7 | 0.53 | 0.221 | -0.17, 1.2 | 0.094 |  |
| 70-79 yrs \* Scale value 4 to 7 | -0.21 | 0.279 | -1.1, 0.68 | 0.5 |  |
| 80+ \* Scale value 4 to 7 | 0.42 | 0.317 | -0.59, 1.4 | 0.3 |  |
| 30-39 yrs \* Scale value 8 to 10 | -0.35 | 0.265 | -1.2, 0.50 | 0.3 |  |
| 40-49 yrs \* Scale value 8 to 10 | -0.02 | 0.276 | -0.89, 0.86 | >0.9 |  |
| 50-59 yrs \* Scale value 8 to 10 | 0.26 | 0.275 | -0.61, 1.1 | 0.4 |  |
| 60-69 yrs \* Scale value 8 to 10 | 0.16 | 0.284 | -0.74, 1.1 | 0.6 |  |
| 70-79 yrs \* Scale value 8 to 10 | 0.15 | 0.420 | -1.2, 1.5 | 0.8 |  |
| 80+ \* Scale value 8 to 10 | -0.04 | 0.430 | -1.4, 1.3 | >0.9 |  |
| Age \* Paying attention to health |  |  |  |  | 0.573 |
| 30-39 yrs \* Strong/very strong | -0.04 | 0.198 | -0.67, 0.59 | 0.9 |  |
| 40-49 yrs \* Strong/very strong | 0.16 | 0.199 | -0.47, 0.80 | 0.5 |  |
| 50-59 yrs \* Strong/very strong | -0.14 | 0.201 | -0.78, 0.50 | 0.5 |  |
| 60-69 yrs \* Strong/very strong | -0.32 | 0.228 | -1.0, 0.41 | 0.3 |  |
| 70-79 yrs \* Strong/very strong | -0.17 | 0.261 | -1.0, 0.66 | 0.6 |  |
| 80+ \* Strong/very strong | 0.10 | 0.292 | -0.83, 1.0 | 0.8 |  |
| Age \* Self-rated mental health |  |  |  |  | 0.752 |
| 30-39 yrs \* fair/poor | 0.08 | 0.234 | -0.66, 0.83 | 0.7 |  |
| 40-49 yrs \* fair/poor | -0.30 | 0.266 | -1.1, 0.55 | 0.3 |  |
| 50-59 yrs \* fair/poor | -0.07 | 0.274 | -0.94, 0.80 | 0.8 |  |
| 60-69 yrs \* fair/poor | -0.14 | 0.301 | -1.1, 0.81 | 0.7 |  |
| 70-79 yrs \* fair/poor | -0.10 | 0.327 | -1.1, 0.94 | 0.8 |  |
| 80+ \* fair/poor | -0.45 | 0.337 | -1.5, 0.62 | 0.3 |  |
| Age \* Smoking |  |  |  |  | 0.074 |
| 30-39 yrs \* Occasional smoking | -0.01 | 0.355 | -1.1, 1.1 | >0.9 |  |
| 40-49 yrs \* Occasional smoking | -0.29 | 0.360 | -1.4, 0.86 | 0.5 |  |
| 50-59 yrs \* Occasional smoking | 0.26 | 0.383 | -0.96, 1.5 | 0.5 |  |
| 60-69 yrs \* Occasional smoking | 0.80 | 0.465 | -0.68, 2.3 | 0.2 |  |
| 70-79 yrs \* Occasional smoking | 0.80 | 1.17 | -2.9, 4.5 | 0.5 |  |
| 80+ \* Occasional smoking | -0.47 | 1.32 | -4.7, 3.7 | 0.7 |  |
| 30-39 yrs \* Non-smoker | -0.20 | 0.248 | -0.99, 0.59 | 0.5 |  |
| 40-49 yrs \* Non-smoker | 0.02 | 0.250 | -0.77, 0.81 | >0.9 |  |
| 50-59 yrs \* Non-smoker | 0.04 | 0.239 | -0.72, 0.80 | 0.9 |  |
| 60-69 yrs \* Non-smoker | -0.03 | 0.257 | -0.85, 0.79 | >0.9 |  |
| 70-79 yrs \* Non-smoker | -1.1 | 0.432 | -2.5, 0.25 | 0.080 |  |
| 80+ \* Non-smoker | 0.11 | 0.556 | -1.7, 1.9 | 0.9 |  |
| Age \* Sausage products |  |  |  |  | 0.771 |
| 30-39 yrs \* 4 to 6 times per week | 0.24 | 0.400 | -1.0, 1.5 | 0.6 |  |
| 40-49 yrs \* 4 to 6 times per week | 0.15 | 0.381 | -1.1, 1.4 | 0.7 |  |
| 50-59 yrs \* 4 to 6 times per week | 0.37 | 0.376 | -0.83, 1.6 | 0.4 |  |
| 60-69 yrs \* 4 to 6 times per week | 0.81 | 0.406 | -0.48, 2.1 | 0.14 |  |
| 70-79 yrs \* 4 to 6 times per week | 0.07 | 0.455 | -1.4, 1.5 | 0.9 |  |
| 80+ \* 4 to 6 times per week | 1.1 | 0.530 | -0.54, 2.8 | 0.12 |  |
| 30-39 yrs \* 1 to 3 times per week | 0.07 | 0.394 | -1.2, 1.3 | 0.9 |  |
| 40-49 yrs \* 1 to 3 times per week | 0.02 | 0.358 | -1.1, 1.2 | >0.9 |  |
| 50-59 yrs \* 1 to 3 times per week | 0.39 | 0.354 | -0.74, 1.5 | 0.4 |  |
| 60-69 yrs \* 1 to 3 times per week | 0.30 | 0.382 | -0.91, 1.5 | 0.5 |  |
| 70-79 yrs \* 1 to 3 times per week | -0.02 | 0.432 | -1.4, 1.4 | >0.9 |  |
| 80+ \* 1 to 3 times per week | 0.37 | 0.471 | -1.1, 1.9 | 0.5 |  |
| 30-39 yrs \* Less than once per week | 0.23 | 0.400 | -1.0, 1.5 | 0.6 |  |
| 40-49 yrs \* Less than once per week | 0.14 | 0.392 | -1.1, 1.4 | 0.7 |  |
| 50-59 yrs \* Less than once per week | 0.13 | 0.403 | -1.1, 1.4 | 0.8 |  |
| 60-69 yrs \* Less than once per week | 0.11 | 0.387 | -1.1, 1.3 | 0.8 |  |
| 70-79 yrs \* Less than once per week | -0.28 | 0.461 | -1.7, 1.2 | 0.6 |  |
| 80+ \* Less than once per week | 0.16 | 0.524 | -1.5, 1.8 | 0.8 |  |
| 30-39 yrs \* Never | 0.02 | 0.453 | -1.4, 1.5 | >0.9 |  |
| 40-49 yrs \* Never | -0.34 | 0.518 | -2.0, 1.3 | 0.6 |  |
| 50-59 yrs \* Never | -0.12 | 0.505 | -1.7, 1.5 | 0.8 |  |
| 60-69 yrs \* Never | 0.32 | 0.584 | -1.5, 2.2 | 0.6 |  |
| 70-79 yrs \* Never | -0.02 | 0.811 | -2.6, 2.6 | >0.9 |  |
| 80+ \* Never | -0.71 | 0.684 | -2.9, 1.5 | 0.4 |  |
| Age \* Red meat |  |  |  |  | 0.032 |
| 30-39 yrs \* 4 to 6 times per week | 0.22 | 0.547 | -1.5, 2.0 | 0.7 |  |
| 40-49 yrs \* 4 to 6 times per week | 0.63 | 0.541 | -1.1, 2.4 | 0.3 |  |
| 50-59 yrs \* 4 to 6 times per week | 0.28 | 0.630 | -1.7, 2.3 | 0.7 |  |
| 60-69 yrs \* 4 to 6 times per week | 2.3 | 0.764 | -0.16, 4.7 | 0.059 |  |
| 70-79 yrs \* 4 to 6 times per week | 0.42 | 1.04 | -2.9, 3.7 | 0.7 |  |
| 80+ \* 4 to 6 times per week | -1.3 | 0.953 | -4.4, 1.7 | 0.3 |  |
| 30-39 yrs \* 1 to 3 times per week | 0.26 | 0.511 | -1.4, 1.9 | 0.7 |  |
| 40-49 yrs \* 1 to 3 times per week | 0.62 | 0.506 | -0.99, 2.2 | 0.3 |  |
| 50-59 yrs \* 1 to 3 times per week | 0.23 | 0.593 | -1.7, 2.1 | 0.7 |  |
| 60-69 yrs \* 1 to 3 times per week | 1.5 | 0.660 | -0.60, 3.6 | 0.11 |  |
| 70-79 yrs \* 1 to 3 times per week | 0.69 | 0.998 | -2.5, 3.9 | 0.5 |  |
| 80+ \* 1 to 3 times per week | 0.19 | 0.886 | -2.6, 3.0 | 0.8 |  |
| 30-39 yrs \* Less than once per week | 0.15 | 0.540 | -1.6, 1.9 | 0.8 |  |
| 40-49 yrs \* Less than once per week | 0.48 | 0.544 | -1.3, 2.2 | 0.4 |  |
| 50-59 yrs \* Less than once per week | 0.45 | 0.601 | -1.5, 2.4 | 0.5 |  |
| 60-69 yrs \* Less than once per week | 1.8 | 0.713 | -0.45, 4.1 | 0.084 |  |
| 70-79 yrs \* Less than once per week | 0.82 | 1.03 | -2.4, 4.1 | 0.5 |  |
| 80+ \* Less than once per week | -0.04 | 0.932 | -3.0, 2.9 | >0.9 |  |
| 30-39 yrs \* Never | 0.06 | 0.608 | -1.9, 2.0 | >0.9 |  |
| 40-49 yrs \* Never | 0.21 | 0.619 | -1.8, 2.2 | 0.8 |  |
| 50-59 yrs \* Never | 0.17 | 0.704 | -2.1, 2.4 | 0.8 |  |
| 60-69 yrs \* Never | 1.2 | 0.756 | -1.2, 3.6 | 0.2 |  |
| 70-79 yrs \* Never | 1.4 | 1.16 | -2.3, 5.1 | 0.3 |  |
| 80+ \* Never | -0.45 | 0.954 | -3.5, 2.6 | 0.7 |  |
| Age \* Sport |  |  |  |  | 0.353 |
| 30-39 yrs \* Less than 1 hour per week | 0.24 | 0.292 | -0.69, 1.2 | 0.5 |  |
| 40-49 yrs \* Less than 1 hour per week | -0.11 | 0.301 | -1.1, 0.85 | 0.7 |  |
| 50-59 yrs \* Less than 1 hour per week | 0.00 | 0.292 | -0.93, 0.93 | >0.9 |  |
| 60-69 yrs \* Less than 1 hour per week | -0.39 | 0.318 | -1.4, 0.63 | 0.3 |  |
| 70-79 yrs \* Less than 1 hour per week | 0.24 | 0.400 | -1.0, 1.5 | 0.6 |  |
| 80+ \* Less than 1 hour per week | -0.45 | 0.384 | -1.7, 0.77 | 0.3 |  |
| 30-39 yrs \* 1 to less than 2 hours per week | -0.14 | 0.299 | -1.1, 0.82 | 0.7 |  |
| 40-49 yrs \* 1 to less than 2 hours per week | -0.20 | 0.297 | -1.1, 0.74 | 0.5 |  |
| 50-59 yrs \* 1 to less than 2 hours per week | -0.23 | 0.292 | -1.2, 0.70 | 0.5 |  |
| 60-69 yrs \* 1 to less than 2 hours per week | -0.50 | 0.305 | -1.5, 0.47 | 0.2 |  |
| 70-79 yrs \* 1 to less than 2 hours per week | 0.14 | 0.366 | -1.0, 1.3 | 0.7 |  |
| 80+ \* 1 to less than 2 hours per week | -0.28 | 0.393 | -1.5, 0.97 | 0.5 |  |
| 30-39 yrs \* 2 to less than 4 hours per week | -0.24 | 0.324 | -1.3, 0.79 | 0.5 |  |
| 40-49 yrs \* 2 to less than 4 hours per week | -0.73 | 0.323 | -1.8, 0.30 | 0.11 |  |
| 50-59 yrs \* 2 to less than 4 hours per week | -0.20 | 0.311 | -1.2, 0.79 | 0.6 |  |
| 60-69 yrs \* 2 to less than 4 hours per week | -0.14 | 0.379 | -1.3, 1.1 | 0.7 |  |
| 70-79 yrs \* 2 to less than 4 hours per week | 0.65 | 0.441 | -0.75, 2.0 | 0.2 |  |
| 80+ \* 2 to less than 4 hours per week | -0.09 | 0.434 | -1.5, 1.3 | 0.8 |  |
| 30-39 yrs \* 4 hours per week and more | -0.13 | 0.321 | -1.1, 0.89 | 0.7 |  |
| 40-49 yrs \* 4 hours per week and more | -0.57 | 0.357 | -1.7, 0.56 | 0.2 |  |
| 50-59 yrs \* 4 hours per week and more | 0.08 | 0.351 | -1.0, 1.2 | 0.8 |  |
| 60-69 yrs \* 4 hours per week and more | -0.47 | 0.393 | -1.7, 0.78 | 0.3 |  |
| 70-79 yrs \* 4 hours per week and more | 0.25 | 0.443 | -1.2, 1.7 | 0.6 |  |
| 80+ \* 4 hours per week and more | -0.08 | 0.534 | -1.8, 1.6 | 0.9 |  |
| Age \* Waited for medical examination date in the last 12 months |  |  |  |  | 0.402 |
| 30-39 yrs \* No | -0.19 | 0.201 | -0.83, 0.45 | 0.4 |  |
| 40-49 yrs \* No | -0.04 | 0.203 | -0.69, 0.61 | 0.9 |  |
| 50-59 yrs \* No | -0.15 | 0.211 | -0.82, 0.52 | 0.5 |  |
| 60-69 yrs \* No | -0.14 | 0.235 | -0.89, 0.61 | 0.6 |  |
| 70-79 yrs \* No | -0.30 | 0.312 | -1.3, 0.69 | 0.4 |  |
| 80+ \* No | -0.36 | 0.336 | -1.4, 0.71 | 0.4 |  |
| 30-39 yrs \* No need for examination or treatment | -0.21 | 0.257 | -1.0, 0.61 | 0.5 |  |
| 40-49 yrs \* No need for examination or treatment | -0.11 | 0.297 | -1.1, 0.84 | 0.7 |  |
| 50-59 yrs \* No need for examination or treatment | -0.28 | 0.314 | -1.3, 0.72 | 0.4 |  |
| 60-69 yrs \* No need for examination or treatment | 0.62 | 0.455 | -0.83, 2.1 | 0.3 |  |
| 70-79 yrs \* No need for examination or treatment | 0.12 | 0.704 | -2.1, 2.4 | 0.9 |  |
| 80+ \* No need for examination or treatment | -1.5 | 0.662 | -3.6, 0.61 | 0.11 |  |
| Age \* Interview type |  |  |  |  | 0.018 |
| 30-39 yrs \* Paper and Pencil Interview | -0.35 | 0.368 | -1.5, 0.82 | 0.4 |  |
| 40-49 yrs \* Paper and Pencil Interview | -0.52 | 0.340 | -1.6, 0.56 | 0.2 |  |
| 50-59 yrs \* Paper and Pencil Interview | -0.78 | 0.313 | -1.8, 0.21 | 0.088 |  |
| 60-69 yrs \* Paper and Pencil Interview | -0.13 | 0.324 | -1.2, 0.90 | 0.7 |  |
| 70-79 yrs \* Paper and Pencil Interview | 0.17 | 0.344 | -0.92, 1.3 | 0.6 |  |
| 80+ \* Paper and Pencil Interview | -0.19 | 0.412 | -1.5, 1.1 | 0.7 |  |
| Age \* BIK community size (categorized) |  |  |  |  | 0.287 |
| 30-39 yrs \* BIK region 20,000 to <50,000 inhabitants OR surroundings 50,000 to <500,000 inhabitants | -0.24 | 0.329 | -1.3, 0.81 | 0.5 |  |
| 40-49 yrs \* BIK region 20,000 to <50,000 inhabitants OR surroundings 50,000 to <500,000 inhabitants | 0.12 | 0.349 | -0.99, 1.2 | 0.7 |  |
| 50-59 yrs \* BIK region 20,000 to <50,000 inhabitants OR surroundings 50,000 to <500,000 inhabitants | -0.21 | 0.381 | -1.4, 1.0 | 0.6 |  |
| 60-69 yrs \* BIK region 20,000 to <50,000 inhabitants OR surroundings 50,000 to <500,000 inhabitants | 0.24 | 0.353 | -0.88, 1.4 | 0.5 |  |
| 70-79 yrs \* BIK region 20,000 to <50,000 inhabitants OR surroundings 50,000 to <500,000 inhabitants | 0.46 | 0.344 | -0.63, 1.6 | 0.3 |  |
| 80+ \* BIK region 20,000 to <50,000 inhabitants OR surroundings 50,000 to <500,000 inhabitants | 0.71 | 0.444 | -0.70, 2.1 | 0.2 |  |
| 30-39 yrs \* Core city 50,000 to <500,000 inhabitants OR surroundings 500,000+ inhabitants | 0.07 | 0.353 | -1.1, 1.2 | 0.9 |  |
| 40-49 yrs \* Core city 50,000 to <500,000 inhabitants OR surroundings 500,000+ inhabitants | 0.09 | 0.366 | -1.1, 1.3 | 0.8 |  |
| 50-59 yrs \* Core city 50,000 to <500,000 inhabitants OR surroundings 500,000+ inhabitants | 0.10 | 0.391 | -1.1, 1.3 | 0.8 |  |
| 60-69 yrs \* Core city 50,000 to <500,000 inhabitants OR surroundings 500,000+ inhabitants | 0.43 | 0.375 | -0.76, 1.6 | 0.3 |  |
| 70-79 yrs \* Core city 50,000 to <500,000 inhabitants OR surroundings 500,000+ inhabitants | 0.65 | 0.383 | -0.57, 1.9 | 0.2 |  |
| 80+ \* Core city 50,000 to <500,000 inhabitants OR surroundings 500,000+ inhabitants | 1.2 | 0.496 | -0.41, 2.7 | 0.10 |  |
| 30-39 yrs \* Core city 500,000+ inhabitants | -0.29 | 0.357 | -1.4, 0.85 | 0.5 |  |
| 40-49 yrs \* Core city 500,000+ inhabitants | -0.08 | 0.357 | -1.2, 1.1 | 0.8 |  |
| 50-59 yrs \* Core city 500,000+ inhabitants | 0.23 | 0.395 | -1.0, 1.5 | 0.6 |  |
| 60-69 yrs \* Core city 500,000+ inhabitants | 0.49 | 0.386 | -0.74, 1.7 | 0.3 |  |
| 70-79 yrs \* Core city 500,000+ inhabitants | 0.32 | 0.380 | -0.89, 1.5 | 0.5 |  |
| 80+ \* Core city 500,000+ inhabitants | 1.0 | 0.469 | -0.48, 2.5 | 0.12 |  |
| Age \* Regions |  |  |  |  | >0.9 |
| 30-39 yrs \* Northwest | -0.39 | 0.299 | -1.3, 0.56 | 0.3 |  |
| 40-49 yrs \* Northwest | -0.18 | 0.316 | -1.2, 0.82 | 0.6 |  |
| 50-59 yrs \* Northwest | 0.06 | 0.305 | -0.91, 1.0 | 0.9 |  |
| 60-69 yrs \* Northwest | 0.12 | 0.370 | -1.1, 1.3 | 0.8 |  |
| 70-79 yrs \* Northwest | 0.15 | 0.369 | -1.0, 1.3 | 0.7 |  |
| 80+ \* Northwest | -0.44 | 0.438 | -1.8, 0.96 | 0.4 |  |
| 30-39 yrs \* Central-East | -0.68 | 0.370 | -1.9, 0.50 | 0.2 |  |
| 40-49 yrs \* Central-East | -0.50 | 0.374 | -1.7, 0.69 | 0.3 |  |
| 50-59 yrs \* Central-East | 0.18 | 0.355 | -0.95, 1.3 | 0.6 |  |
| 60-69 yrs \* Central-East | -0.28 | 0.406 | -1.6, 1.0 | 0.5 |  |
| 70-79 yrs \* Central-East | -0.04 | 0.399 | -1.3, 1.2 | >0.9 |  |
| 80+ \* Central-East | 0.45 | 0.572 | -1.4, 2.3 | 0.5 |  |
| 30-39 yrs \* Central-West | -0.31 | 0.276 | -1.2, 0.57 | 0.3 |  |
| 40-49 yrs \* Central-West | -0.13 | 0.296 | -1.1, 0.81 | 0.7 |  |
| 50-59 yrs \* Central-West | -0.06 | 0.267 | -0.91, 0.79 | 0.8 |  |
| 60-69 yrs \* Central-West | 0.02 | 0.317 | -0.99, 1.0 | >0.9 |  |
| 70-79 yrs \* Central-West | -0.02 | 0.366 | -1.2, 1.1 | >0.9 |  |
| 80+ \* Central-West | -0.20 | 0.390 | -1.4, 1.0 | 0.6 |  |
| 30-39 yrs \* South | -0.17 | 0.281 | -1.1, 0.73 | 0.6 |  |
| 40-49 yrs \* South | 0.01 | 0.323 | -1.0, 1.0 | >0.9 |  |
| 50-59 yrs \* South | 0.16 | 0.294 | -0.78, 1.1 | 0.6 |  |
| 60-69 yrs \* South | -0.13 | 0.335 | -1.2, 0.93 | 0.7 |  |
| 70-79 yrs \* South | 0.07 | 0.400 | -1.2, 1.3 | 0.9 |  |
| 80+ \* South | 0.04 | 0.428 | -1.3, 1.4 | >0.9 |  |
| Age \* Country of birth |  |  |  |  | 0.395 |
| 30-39 yrs \* In another country | 0.20 | 0.283 | -0.71, 1.1 | 0.5 |  |
| 40-49 yrs \* In another country | -0.17 | 0.306 | -1.1, 0.81 | 0.6 |  |
| 50-59 yrs \* In another country | 0.40 | 0.318 | -0.61, 1.4 | 0.3 |  |
| 60-69 yrs \* In another country | 0.07 | 0.352 | -1.1, 1.2 | 0.9 |  |
| 70-79 yrs \* In another country | -0.23 | 0.414 | -1.5, 1.1 | 0.6 |  |
| 80+ \* In another country | 0.58 | 0.390 | -0.66, 1.8 | 0.2 |  |
| Age \* Household size |  |  |  |  | 0.514 |
| 30-39 yrs \* Multi-person household | 0.41 | 0.230 | -0.33, 1.1 | 0.2 |  |
| 40-49 yrs \* Multi-person household | -0.08 | 0.266 | -0.92, 0.77 | 0.8 |  |
| 50-59 yrs \* Multi-person household | 0.13 | 0.251 | -0.67, 0.93 | 0.6 |  |
| 60-69 yrs \* Multi-person household | 0.18 | 0.274 | -0.69, 1.1 | 0.6 |  |
| 70-79 yrs \* Multi-person household | 0.18 | 0.307 | -0.79, 1.2 | 0.6 |  |
| 80+ \* Multi-person household | 0.36 | 0.329 | -0.68, 1.4 | 0.4 |  |
| Age \* Education: ISCED (2011) |  |  |  |  | 0.005 |
| 30-39 yrs \* Medium | 0.90 | 0.361 | -0.25, 2.0 | 0.089 |  |
| 40-49 yrs \* Medium | 0.26 | 0.368 | -0.92, 1.4 | 0.5 |  |
| 50-59 yrs \* Medium | 0.66 | 0.318 | -0.35, 1.7 | 0.13 |  |
| 60-69 yrs \* Medium | 0.34 | 0.291 | -0.59, 1.3 | 0.3 |  |
| 70-79 yrs \* Medium | 0.51 | 0.337 | -0.56, 1.6 | 0.2 |  |
| 80+ \* Medium | 0.02 | 0.346 | -1.1, 1.1 | >0.9 |  |
| 30-39 yrs \* High | 1.2 | 0.365 | 0.03, 2.4 | 0.047 |  |
| 40-49 yrs \* High | 0.29 | 0.378 | -0.91, 1.5 | 0.5 |  |
| 50-59 yrs \* High | 0.18 | 0.325 | -0.86, 1.2 | 0.6 |  |
| 60-69 yrs \* High | 0.32 | 0.361 | -0.83, 1.5 | 0.4 |  |
| 70-79 yrs \* High | 0.35 | 0.428 | -1.0, 1.7 | 0.5 |  |
| 80+ \* High | -0.18 | 0.420 | -1.5, 1.2 | 0.7 |  |
| Sex \* Self-rated health |  |  |  |  | 0.598 |
| Female \* Bad/very bad | 0.13 | 0.243 | -0.65, 0.90 | 0.6 |  |
| Sex \* Smoking |  |  |  |  | 0.618 |
| Female \* Occasional smoking | 0.22 | 0.233 | -0.52, 0.96 | 0.4 |  |
| Female \* Non-smoker | 0.03 | 0.147 | -0.43, 0.50 | 0.8 |  |
| Sex \* Red meat |  |  |  |  | 0.53 |
| Female \* 4 to 6 times per week | -0.78 | 0.509 | -2.4, 0.83 | 0.2 |  |
| Female \* 1 to 3 times per week | -0.68 | 0.474 | -2.2, 0.83 | 0.2 |  |
| Female \* Less than once per week | -0.71 | 0.493 | -2.3, 0.86 | 0.2 |  |
| Female \* Never | -0.86 | 0.506 | -2.5, 0.75 | 0.2 |  |
| Sex \* Sport |  |  |  |  | 0.082 |
| Female \* Less than 1 hour per week | 0.09 | 0.171 | -0.45, 0.63 | 0.6 |  |
| Female \* 1 to less than 2 hours per week | -0.12 | 0.172 | -0.67, 0.43 | 0.5 |  |
| Female \* 2 to less than 4 hours per week | 0.25 | 0.195 | -0.37, 0.87 | 0.3 |  |
| Female \* 4 hours per week and more | 0.39 | 0.211 | -0.29, 1.1 | 0.2 |  |
| Sex \* Waited for medical examination date in the last 12 months |  |  |  |  | 0.05 |
| Female \* No | -0.08 | 0.116 | -0.45, 0.29 | 0.6 |  |
| Female \* No need for examination or treatment | 0.36 | 0.180 | -0.21, 0.93 | 0.14 |  |
| Sex \* Country of birth |  |  |  |  | 0.423 |
| Female \* In another country | -0.15 | 0.193 | -0.77, 0.46 | 0.5 |  |
| Obesity \* Education: CASMIN |  |  |  |  | 0.405 |
| No \* Medium | 0.24 | 0.184 | -0.34, 0.83 | 0.3 |  |
| No \* High | 0.23 | 0.219 | -0.47, 0.92 | 0.4 |  |
| Satisfaction: Life in general \* Education: CASMIN |  |  |  |  | 0.145 |
| Scale value 4 to 7 \* Medium | 0.25 | 0.264 | -0.59, 1.1 | 0.4 |  |
| Scale value 8 to 10 \* Medium | 0.37 | 0.270 | -0.49, 1.2 | 0.3 |  |
| Scale value 4 to 7 \* High | -0.30 | 0.349 | -1.4, 0.82 | 0.5 |  |
| Scale value 8 to 10 \* High | -0.01 | 0.340 | -1.1, 1.1 | >0.9 |  |
| Health risk due to climate change \* Education: CASMIN |  |  |  |  | 0.462 |
| Scale value 4 to 7 \* Medium | -0.05 | 0.184 | -0.63, 0.54 | 0.8 |  |
| Scale value 8 to 10 \* Medium | 0.26 | 0.235 | -0.49, 1.0 | 0.4 |  |
| Scale value 4 to 7 \* High | -0.21 | 0.213 | -0.89, 0.47 | 0.4 |  |
| Scale value 8 to 10 \* High | 0.11 | 0.267 | -0.74, 0.96 | 0.7 |  |
| Paying attention to health \* Education: CASMIN |  |  |  |  | 0.234 |
| Strong/very strong \* Medium | -0.27 | 0.163 | -0.79, 0.25 | 0.2 |  |
| Strong/very strong \* High | -0.14 | 0.182 | -0.72, 0.44 | 0.5 |  |
| Sausage products \* Education: CASMIN |  |  |  |  | 0.191 |
| 4 to 6 times per week \* Medium | 0.50 | 0.284 | -0.40, 1.4 | 0.2 |  |
| 1 to 3 times per week \* Medium | 0.30 | 0.248 | -0.49, 1.1 | 0.3 |  |
| Less than once per week \* Medium | -0.06 | 0.275 | -0.94, 0.81 | 0.8 |  |
| Never \* Medium | 0.03 | 0.395 | -1.2, 1.3 | >0.9 |  |
| 4 to 6 times per week \* High | 0.42 | 0.360 | -0.73, 1.6 | 0.3 |  |
| 1 to 3 times per week \* High | 0.00 | 0.308 | -0.98, 0.98 | >0.9 |  |
| Less than once per week \* High | -0.15 | 0.341 | -1.2, 0.93 | 0.7 |  |
| Never \* High | -0.54 | 0.465 | -2.0, 0.94 | 0.3 |  |
| Red meat \* Education: CASMIN |  |  |  |  | 0.493 |
| 4 to 6 times per week \* Medium | -0.38 | 0.436 | -1.8, 1.0 | 0.4 |  |
| 1 to 3 times per week \* Medium | 0.11 | 0.401 | -1.2, 1.4 | 0.8 |  |
| Less than once per week \* Medium | 0.07 | 0.406 | -1.2, 1.4 | 0.9 |  |
| Never \* Medium | 0.55 | 0.487 | -1.0, 2.1 | 0.3 |  |
| 4 to 6 times per week \* High | -0.48 | 0.574 | -2.3, 1.3 | 0.5 |  |
| 1 to 3 times per week \* High | -0.19 | 0.546 | -1.9, 1.6 | 0.8 |  |
| Less than once per week \* High | -0.27 | 0.560 | -2.0, 1.5 | 0.7 |  |
| Never \* High | 0.39 | 0.646 | -1.7, 2.4 | 0.6 |  |
| Sport \* Education: CASMIN |  |  |  |  | 0.691 |
| Less than 1 hour per week \* Medium | -0.23 | 0.230 | -0.96, 0.51 | 0.4 |  |
| 1 to less than 2 hours per week \* Medium | 0.03 | 0.214 | -0.65, 0.71 | >0.9 |  |
| 2 to less than 4 hours per week \* Medium | -0.24 | 0.262 | -1.1, 0.60 | 0.4 |  |
| 4 hours per week and more \* Medium | -0.22 | 0.273 | -1.1, 0.65 | 0.5 |  |
| Less than 1 hour per week \* High | -0.25 | 0.274 | -1.1, 0.62 | 0.4 |  |
| 1 to less than 2 hours per week \* High | 0.09 | 0.283 | -0.82, 0.99 | 0.8 |  |
| 2 to less than 4 hours per week \* High | -0.25 | 0.304 | -1.2, 0.71 | 0.5 |  |
| 4 hours per week and more \* High | 0.09 | 0.331 | -0.97, 1.1 | 0.8 |  |
| Waited for medical examination date in the last 12 months \* Education: CASMIN |  |  |  |  | 0.468 |
| No \* Medium | -0.04 | 0.179 | -0.61, 0.53 | 0.8 |  |
| No need for examination or treatment \* Medium | -0.40 | 0.313 | -1.4, 0.60 | 0.3 |  |
| No \* High | 0.15 | 0.187 | -0.45, 0.74 | 0.5 |  |
| No need for examination or treatment \* High | -0.20 | 0.319 | -1.2, 0.81 | 0.6 |  |
| Education: CASMIN \* Foreign nationality |  |  |  |  | 0.816 |
| Medium \* Not foreign national | 0.14 | 0.214 | -0.55, 0.82 | 0.6 |  |
| High \* Not foreign national | 0.08 | 0.214 | -0.60, 0.76 | 0.7 |  |
| BIK community size (categorized) \* Education: CASMIN |  |  |  |  | 0.334 |
| BIK region 20,000 to <50,000 inhabitants OR surroundings 50,000 to <500,000 inhabitants \* Medium | 0.12 | 0.229 | -0.61, 0.85 | 0.6 |  |
| Core city 50,000 to <500,000 inhabitants OR surroundings 500,000+ inhabitants \* Medium | -0.12 | 0.237 | -0.87, 0.64 | 0.7 |  |
| Core city 500,000+ inhabitants \* Medium | 0.26 | 0.251 | -0.54, 1.1 | 0.4 |  |
| BIK region 20,000 to <50,000 inhabitants OR surroundings 50,000 to <500,000 inhabitants \* High | 0.41 | 0.261 | -0.42, 1.2 | 0.2 |  |
| Core city 50,000 to <500,000 inhabitants OR surroundings 500,000+ inhabitants \* High | 0.30 | 0.283 | -0.60, 1.2 | 0.4 |  |
| Core city 500,000+ inhabitants \* High | 0.44 | 0.280 | -0.45, 1.3 | 0.2 |  |
| Regions \* Education: CASMIN |  |  |  |  | 0.22 |
| Northwest \* Medium | 0.47 | 0.260 | -0.36, 1.3 | 0.2 |  |
| Central-East \* Medium | 0.65 | 0.369 | -0.52, 1.8 | 0.2 |  |
| Central-West \* Medium | 0.26 | 0.244 | -0.51, 1.0 | 0.4 |  |
| South \* Medium | 0.15 | 0.253 | -0.65, 0.95 | 0.6 |  |
| Northwest \* High | 0.21 | 0.317 | -0.80, 1.2 | 0.6 |  |
| Central-East \* High | 0.75 | 0.455 | -0.70, 2.2 | 0.2 |  |
| Central-West \* High | -0.06 | 0.303 | -1.0, 0.91 | 0.9 |  |
| South \* High | 0.11 | 0.306 | -0.86, 1.1 | 0.7 |  |
| Household size \* Education: CASMIN |  |  |  |  | 0.17 |
| Multi-person household \* Medium | -0.09 | 0.203 | -0.74, 0.56 | 0.7 |  |
| Multi-person household \* High | -0.37 | 0.234 | -1.1, 0.38 | 0.2 |  |
| Current living situation \* Education: CASMIN |  |  |  |  | 0.783 |
| Part-time employed \* Medium | 0.32 | 0.234 | -0.42, 1.1 | 0.3 |  |
| Unemployed \* Medium | 0.19 | 0.316 | -0.82, 1.2 | 0.6 |  |
| Retired or early retired \* Medium | 0.29 | 0.198 | -0.35, 0.92 | 0.2 |  |
| Not employed for other reasons (student, volunteer service, homemaker) \* Medium | 0.20 | 0.232 | -0.54, 0.94 | 0.5 |  |
| Part-time employed \* High | 0.32 | 0.242 | -0.46, 1.1 | 0.3 |  |
| Unemployed \* High | -0.14 | 0.391 | -1.4, 1.1 | 0.7 |  |
| Retired or early retired \* High | 0.35 | 0.301 | -0.60, 1.3 | 0.3 |  |
| Not employed for other reasons (student, volunteer service, homemaker) \* High | -0.02 | 0.287 | -0.93, 0.90 | >0.9 |  |
| N.Obs | 11,778 |  |  |  |  |
| N.Cluster | 359 |  |  |  |  |
|  |  |  |  |  |  |
| --- | --- | --- | --- | --- | --- |
| Abbreviations: CI = Confidence Interval, OR = Odds Ratio, SE = Standard Error | | | | | |

## Result model estimation: Questionaire B

### Quarter 2

| Characteristic | log(OR) | SE | 95% CI | p-value | p-value (global) |
| --- | --- | --- | --- | --- | --- |
| Sex |  |  |  |  | 0.161 |
| Male | — | — | — |  |  |
| Female | 0.76 | 0.543 | -0.36, 1.9 | 0.2 |  |
| Age |  |  |  |  | 0.277 |
| 16-29 yrs | — | — | — |  |  |
| 30-39 yrs | 0.92 | 0.891 | -0.91, 2.8 | 0.3 |  |
| 40-49 yrs | 0.72 | 0.908 | -1.2, 2.6 | 0.4 |  |
| 50-59 yrs | 0.75 | 1.04 | -1.4, 2.9 | 0.5 |  |
| 60-69 yrs | 1.7 | 0.934 | -0.26, 3.6 | 0.088 |  |
| 70-79 yrs | 1.6 | 1.27 | -1.1, 4.2 | 0.2 |  |
| 80+ | -1.8 | 1.59 | -5.1, 1.4 | 0.3 |  |
| Education: CASMIN |  |  |  |  | 0.531 |
| Low | — | — | — |  |  |
| Medium | 0.61 | 0.566 | -0.55, 1.8 | 0.3 |  |
| High | 0.31 | 0.700 | -1.1, 1.8 | 0.7 |  |
| Satisfaction: Life in general |  |  |  |  | >0.9 |
| Scale value 1 to 3 | — | — | — |  |  |
| Scale value 4 to 7 | -0.07 | 0.275 | -0.64, 0.49 | 0.8 |  |
| Scale value 8 to 10 | -0.02 | 0.291 | -0.62, 0.58 | >0.9 |  |
| Smoking |  |  |  |  | 0.002 |
| Daily smoking | — | — | — |  |  |
| Occasional smoking | 1.2 | 0.376 | 0.39, 1.9 | 0.005 |  |
| Non-smoker | 0.72 | 0.239 | 0.23, 1.2 | 0.006 |  |
| Interview type |  |  |  |  | 0.478 |
| Computer Assisted Web Interview | — | — | — |  |  |
| Paper and Pencil Interview | -0.20 | 0.288 | -0.80, 0.39 | 0.5 |  |
| Country of birth |  |  |  |  | 0.227 |
| In Germany (within current borders) | — | — | — |  |  |
| In another country | -0.32 | 0.262 | -0.86, 0.22 | 0.2 |  |
| German nationality |  |  |  |  | 0.862 |
| Yes | — | — | — |  |  |
| No | 0.06 | 0.348 | -0.66, 0.78 | 0.9 |  |
| Education: ISCED (2011) |  |  |  |  | 0.157 |
| Low | — | — | — |  |  |
| Medium | 0.39 | 0.209 | -0.04, 0.82 | 0.073 |  |
| High | 0.41 | 0.250 | -0.11, 0.92 | 0.12 |  |
| Current living situation |  |  |  |  | 0.149 |
| Full-time employed | — | — | — |  |  |
| Part-time employed | -0.18 | 0.223 | -0.64, 0.28 | 0.4 |  |
| Unemployed | -0.60 | 0.263 | -1.1, -0.06 | 0.032 |  |
| Retired or early retired | 0.08 | 0.203 | -0.34, 0.50 | 0.7 |  |
| Not employed for other reasons (student, volunteer service, homemaker) | -0.31 | 0.231 | -0.79, 0.17 | 0.2 |  |
| Normal weight |  |  |  |  | 0.623 |
| Normal weight (18.5 <= BMI < 25) | — | — | — |  |  |
| Not normal weight (BMI < 18.5 or BMI >= 25) | -0.09 | 0.186 | -0.47, 0.29 | 0.6 |  |
| Overweight |  |  |  |  | 0.835 |
| Yes | — | — | — |  |  |
| No | 0.05 | 0.217 | -0.40, 0.49 | 0.8 |  |
| Self-rated health |  |  |  |  | 0.321 |
| Very good/good/fair | — | — | — |  |  |
| Bad/very bad | 0.45 | 0.448 | -0.48, 1.4 | 0.3 |  |
| Health risk due to climate change |  |  |  |  | 0.656 |
| Scale value 1 to 3 | — | — | — |  |  |
| Scale value 4 to 7 | 0.04 | 0.223 | -0.42, 0.50 | 0.9 |  |
| Scale value 8 to 10 | -0.19 | 0.256 | -0.71, 0.34 | 0.5 |  |
| Sausage products |  |  |  |  | 0.413 |
| Daily or several times a day | — | — | — |  |  |
| 4 to 6 times per week | -0.37 | 0.379 | -1.2, 0.41 | 0.3 |  |
| 1 to 3 times per week | -0.41 | 0.371 | -1.2, 0.35 | 0.3 |  |
| Less than once per week | -0.39 | 0.385 | -1.2, 0.40 | 0.3 |  |
| Never | -1.0 | 0.514 | -2.1, 0.04 | 0.059 |  |
| Red meat |  |  |  |  | 0.284 |
| Daily or several times a day | — | — | — |  |  |
| 4 to 6 times per week | 0.34 | 0.593 | -0.88, 1.6 | 0.6 |  |
| 1 to 3 times per week | 0.37 | 0.528 | -0.71, 1.5 | 0.5 |  |
| Less than once per week | 0.59 | 0.568 | -0.58, 1.8 | 0.3 |  |
| Never | 1.2 | 0.661 | -0.19, 2.5 | 0.089 |  |
| Sport |  |  |  |  | 0.706 |
| No sporting activities | — | — | — |  |  |
| Less than 1 hour per week | 0.04 | 0.295 | -0.57, 0.64 | 0.9 |  |
| 1 to less than 2 hours per week | 0.19 | 0.300 | -0.42, 0.81 | 0.5 |  |
| 2 to less than 4 hours per week | 0.39 | 0.299 | -0.23, 1.0 | 0.2 |  |
| 4 hours per week and more | 0.09 | 0.360 | -0.65, 0.83 | 0.8 |  |
| Waited for medical examination date in the last 12 months |  |  |  |  | 0.464 |
| Yes | — | — | — |  |  |
| No | 0.13 | 0.149 | -0.18, 0.43 | 0.4 |  |
| No need for examination or treatment | -0.07 | 0.164 | -0.41, 0.26 | 0.7 |  |
| BIK community size (categorized) |  |  |  |  | 0.655 |
| BIK region <20,000 inhabitants | — | — | — |  |  |
| BIK region 20,000 to <50,000 inhabitants OR surroundings 50,000 to <500,000 inhabitants | 0.39 | 0.414 | -0.46, 1.2 | 0.4 |  |
| Core city 50,000 to <500,000 inhabitants OR surroundings 500,000+ inhabitants | 0.14 | 0.415 | -0.71, 1.0 | 0.7 |  |
| Core city 500,000+ inhabitants | 0.32 | 0.418 | -0.54, 1.2 | 0.5 |  |
| Regions |  |  |  |  | 0.596 |
| Northeast | — | — | — |  |  |
| Northwest | 0.18 | 0.377 | -0.59, 0.96 | 0.6 |  |
| Central-East | -0.36 | 0.459 | -1.3, 0.58 | 0.4 |  |
| Central-West | 0.24 | 0.352 | -0.48, 0.97 | 0.5 |  |
| South | 0.19 | 0.363 | -0.56, 0.93 | 0.6 |  |
| Household size |  |  |  |  | 0.42 |
| Single-person household | — | — | — |  |  |
| Multi-person household | -0.14 | 0.169 | -0.48, 0.21 | 0.4 |  |
| Self-rated mental health |  |  |  |  | 0.381 |
| Excellent/very good/good | — | — | — |  |  |
| fair/poor | 0.11 | 0.128 | -0.15, 0.38 | 0.4 |  |
| Age \* Overweight |  |  |  |  | 0.39 |
| 30-39 yrs \* No | -0.14 | 0.205 | -0.56, 0.28 | 0.5 |  |
| 40-49 yrs \* No | 0.06 | 0.217 | -0.38, 0.51 | 0.8 |  |
| 50-59 yrs \* No | -0.19 | 0.227 | -0.65, 0.28 | 0.4 |  |
| 60-69 yrs \* No | 0.24 | 0.249 | -0.28, 0.75 | 0.4 |  |
| 70-79 yrs \* No | -0.26 | 0.277 | -0.83, 0.31 | 0.4 |  |
| 80+ \* No | 0.31 | 0.300 | -0.31, 0.93 | 0.3 |  |
| Age \* Self-rated health |  |  |  |  | 0.323 |
| 30-39 yrs \* Bad/very bad | -0.91 | 0.608 | -2.2, 0.34 | 0.15 |  |
| 40-49 yrs \* Bad/very bad | 0.03 | 0.633 | -1.3, 1.3 | >0.9 |  |
| 50-59 yrs \* Bad/very bad | -0.51 | 0.544 | -1.6, 0.61 | 0.4 |  |
| 60-69 yrs \* Bad/very bad | -0.47 | 0.594 | -1.7, 0.76 | 0.4 |  |
| 70-79 yrs \* Bad/very bad | -0.97 | 0.501 | -2.0, 0.07 | 0.065 |  |
| 80+ \* Bad/very bad | -0.75 | 0.570 | -1.9, 0.42 | 0.2 |  |
| Age \* Satisfaction: Life in general |  |  |  |  | 0.021 |
| 30-39 yrs \* Scale value 4 to 7 | -0.25 | 0.410 | -1.1, 0.60 | 0.5 |  |
| 40-49 yrs \* Scale value 4 to 7 | 0.73 | 0.392 | -0.08, 1.5 | 0.076 |  |
| 50-59 yrs \* Scale value 4 to 7 | -0.29 | 0.448 | -1.2, 0.63 | 0.5 |  |
| 60-69 yrs \* Scale value 4 to 7 | -0.51 | 0.478 | -1.5, 0.48 | 0.3 |  |
| 70-79 yrs \* Scale value 4 to 7 | -0.40 | 0.501 | -1.4, 0.63 | 0.4 |  |
| 80+ \* Scale value 4 to 7 | 0.66 | 0.479 | -0.33, 1.6 | 0.2 |  |
| 30-39 yrs \* Scale value 8 to 10 | -0.27 | 0.421 | -1.1, 0.60 | 0.5 |  |
| 40-49 yrs \* Scale value 8 to 10 | 0.86 | 0.360 | 0.12, 1.6 | 0.025 |  |
| 50-59 yrs \* Scale value 8 to 10 | -0.20 | 0.452 | -1.1, 0.73 | 0.7 |  |
| 60-69 yrs \* Scale value 8 to 10 | -0.46 | 0.476 | -1.4, 0.52 | 0.3 |  |
| 70-79 yrs \* Scale value 8 to 10 | 0.04 | 0.493 | -0.98, 1.1 | >0.9 |  |
| 80+ \* Scale value 8 to 10 | 0.67 | 0.516 | -0.39, 1.7 | 0.2 |  |
| Age \* Health risk due to climate change |  |  |  |  | 0.27 |
| 30-39 yrs \* Scale value 4 to 7 | -0.63 | 0.226 | -1.1, -0.16 | 0.010 |  |
| 40-49 yrs \* Scale value 4 to 7 | -0.24 | 0.248 | -0.75, 0.27 | 0.3 |  |
| 50-59 yrs \* Scale value 4 to 7 | -0.35 | 0.251 | -0.87, 0.17 | 0.2 |  |
| 60-69 yrs \* Scale value 4 to 7 | 0.03 | 0.264 | -0.51, 0.58 | >0.9 |  |
| 70-79 yrs \* Scale value 4 to 7 | 0.00 | 0.291 | -0.60, 0.60 | >0.9 |  |
| 80+ \* Scale value 4 to 7 | -0.12 | 0.338 | -0.82, 0.57 | 0.7 |  |
| 30-39 yrs \* Scale value 8 to 10 | -0.17 | 0.273 | -0.73, 0.40 | 0.6 |  |
| 40-49 yrs \* Scale value 8 to 10 | 0.02 | 0.282 | -0.57, 0.60 | >0.9 |  |
| 50-59 yrs \* Scale value 8 to 10 | -0.07 | 0.297 | -0.68, 0.55 | 0.8 |  |
| 60-69 yrs \* Scale value 8 to 10 | 0.23 | 0.323 | -0.44, 0.89 | 0.5 |  |
| 70-79 yrs \* Scale value 8 to 10 | 0.26 | 0.392 | -0.55, 1.1 | 0.5 |  |
| 80+ \* Scale value 8 to 10 | -0.23 | 0.413 | -1.1, 0.62 | 0.6 |  |
| Age \* Smoking |  |  |  |  | 0.303 |
| 30-39 yrs \* Occasional smoking | -0.34 | 0.365 | -1.1, 0.41 | 0.4 |  |
| 40-49 yrs \* Occasional smoking | 0.11 | 0.492 | -0.90, 1.1 | 0.8 |  |
| 50-59 yrs \* Occasional smoking | -0.48 | 0.423 | -1.4, 0.39 | 0.3 |  |
| 60-69 yrs \* Occasional smoking | -0.23 | 0.630 | -1.5, 1.1 | 0.7 |  |
| 70-79 yrs \* Occasional smoking | -1.7 | 0.663 | -3.0, -0.31 | 0.018 |  |
| 80+ \* Occasional smoking | -0.91 | 1.23 | -3.4, 1.6 | 0.5 |  |
| 30-39 yrs \* Non-smoker | -0.30 | 0.267 | -0.85, 0.25 | 0.3 |  |
| 40-49 yrs \* Non-smoker | -0.11 | 0.290 | -0.71, 0.48 | 0.7 |  |
| 50-59 yrs \* Non-smoker | -0.22 | 0.271 | -0.78, 0.33 | 0.4 |  |
| 60-69 yrs \* Non-smoker | -0.57 | 0.287 | -1.2, 0.02 | 0.057 |  |
| 70-79 yrs \* Non-smoker | -0.45 | 0.429 | -1.3, 0.44 | 0.3 |  |
| 80+ \* Non-smoker | 0.32 | 0.641 | -1.0, 1.6 | 0.6 |  |
| Age \* Sausage products |  |  |  |  | 0.587 |
| 30-39 yrs \* 4 to 6 times per week | 0.29 | 0.399 | -0.53, 1.1 | 0.5 |  |
| 40-49 yrs \* 4 to 6 times per week | 0.55 | 0.405 | -0.28, 1.4 | 0.2 |  |
| 50-59 yrs \* 4 to 6 times per week | 0.91 | 0.425 | 0.04, 1.8 | 0.042 |  |
| 60-69 yrs \* 4 to 6 times per week | 0.34 | 0.409 | -0.50, 1.2 | 0.4 |  |
| 70-79 yrs \* 4 to 6 times per week | 0.64 | 0.480 | -0.35, 1.6 | 0.2 |  |
| 80+ \* 4 to 6 times per week | 0.76 | 0.595 | -0.47, 2.0 | 0.2 |  |
| 30-39 yrs \* 1 to 3 times per week | 0.30 | 0.396 | -0.51, 1.1 | 0.5 |  |
| 40-49 yrs \* 1 to 3 times per week | 0.37 | 0.393 | -0.44, 1.2 | 0.4 |  |
| 50-59 yrs \* 1 to 3 times per week | 0.94 | 0.374 | 0.17, 1.7 | 0.019 |  |
| 60-69 yrs \* 1 to 3 times per week | 0.59 | 0.401 | -0.24, 1.4 | 0.2 |  |
| 70-79 yrs \* 1 to 3 times per week | 0.55 | 0.481 | -0.44, 1.5 | 0.3 |  |
| 80+ \* 1 to 3 times per week | 0.23 | 0.556 | -0.92, 1.4 | 0.7 |  |
| 30-39 yrs \* Less than once per week | 0.52 | 0.428 | -0.36, 1.4 | 0.2 |  |
| 40-49 yrs \* Less than once per week | 0.49 | 0.401 | -0.34, 1.3 | 0.2 |  |
| 50-59 yrs \* Less than once per week | 0.99 | 0.433 | 0.09, 1.9 | 0.032 |  |
| 60-69 yrs \* Less than once per week | 0.30 | 0.415 | -0.56, 1.1 | 0.5 |  |
| 70-79 yrs \* Less than once per week | 0.26 | 0.508 | -0.79, 1.3 | 0.6 |  |
| 80+ \* Less than once per week | 1.1 | 0.635 | -0.24, 2.4 | 0.11 |  |
| 30-39 yrs \* Never | 0.76 | 0.547 | -0.36, 1.9 | 0.2 |  |
| 40-49 yrs \* Never | 0.32 | 0.506 | -0.72, 1.4 | 0.5 |  |
| 50-59 yrs \* Never | 1.1 | 0.514 | 0.04, 2.2 | 0.043 |  |
| 60-69 yrs \* Never | 0.82 | 0.579 | -0.37, 2.0 | 0.2 |  |
| 70-79 yrs \* Never | 0.46 | 0.675 | -0.93, 1.8 | 0.5 |  |
| 80+ \* Never | 0.61 | 0.723 | -0.88, 2.1 | 0.4 |  |
| Age \* Red meat |  |  |  |  | 0.628 |
| 30-39 yrs \* 4 to 6 times per week | 0.02 | 0.560 | -1.1, 1.2 | >0.9 |  |
| 40-49 yrs \* 4 to 6 times per week | 0.05 | 0.607 | -1.2, 1.3 | >0.9 |  |
| 50-59 yrs \* 4 to 6 times per week | -0.20 | 0.769 | -1.8, 1.4 | 0.8 |  |
| 60-69 yrs \* 4 to 6 times per week | -0.61 | 0.709 | -2.1, 0.85 | 0.4 |  |
| 70-79 yrs \* 4 to 6 times per week | -1.0 | 0.888 | -2.8, 0.81 | 0.3 |  |
| 80+ \* 4 to 6 times per week | 0.99 | 1.33 | -1.7, 3.7 | 0.5 |  |
| 30-39 yrs \* 1 to 3 times per week | 0.13 | 0.546 | -1.0, 1.3 | 0.8 |  |
| 40-49 yrs \* 1 to 3 times per week | 0.00 | 0.542 | -1.1, 1.1 | >0.9 |  |
| 50-59 yrs \* 1 to 3 times per week | 0.09 | 0.722 | -1.4, 1.6 | >0.9 |  |
| 60-69 yrs \* 1 to 3 times per week | 0.00 | 0.646 | -1.3, 1.3 | >0.9 |  |
| 70-79 yrs \* 1 to 3 times per week | -0.05 | 0.801 | -1.7, 1.6 | >0.9 |  |
| 80+ \* 1 to 3 times per week | 0.93 | 1.26 | -1.7, 3.5 | 0.5 |  |
| 30-39 yrs \* Less than once per week | 0.19 | 0.577 | -1.0, 1.4 | 0.7 |  |
| 40-49 yrs \* Less than once per week | 0.18 | 0.570 | -0.99, 1.4 | 0.7 |  |
| 50-59 yrs \* Less than once per week | -0.07 | 0.748 | -1.6, 1.5 | >0.9 |  |
| 60-69 yrs \* Less than once per week | 0.05 | 0.740 | -1.5, 1.6 | >0.9 |  |
| 70-79 yrs \* Less than once per week | -0.34 | 0.812 | -2.0, 1.3 | 0.7 |  |
| 80+ \* Less than once per week | 0.66 | 1.30 | -2.0, 3.3 | 0.6 |  |
| 30-39 yrs \* Never | 0.28 | 0.736 | -1.2, 1.8 | 0.7 |  |
| 40-49 yrs \* Never | 0.23 | 0.722 | -1.3, 1.7 | 0.8 |  |
| 50-59 yrs \* Never | -0.13 | 0.834 | -1.9, 1.6 | 0.9 |  |
| 60-69 yrs \* Never | -0.56 | 0.800 | -2.2, 1.1 | 0.5 |  |
| 70-79 yrs \* Never | -0.96 | 0.969 | -3.0, 1.0 | 0.3 |  |
| 80+ \* Never | 0.06 | 1.34 | -2.7, 2.8 | >0.9 |  |
| Age \* Sport |  |  |  |  | 0.444 |
| 30-39 yrs \* Less than 1 hour per week | 0.33 | 0.343 | -0.37, 1.0 | 0.3 |  |
| 40-49 yrs \* Less than 1 hour per week | -0.01 | 0.361 | -0.75, 0.73 | >0.9 |  |
| 50-59 yrs \* Less than 1 hour per week | 0.19 | 0.345 | -0.52, 0.90 | 0.6 |  |
| 60-69 yrs \* Less than 1 hour per week | 0.65 | 0.350 | -0.07, 1.4 | 0.074 |  |
| 70-79 yrs \* Less than 1 hour per week | 0.46 | 0.421 | -0.41, 1.3 | 0.3 |  |
| 80+ \* Less than 1 hour per week | -0.50 | 0.427 | -1.4, 0.38 | 0.3 |  |
| 30-39 yrs \* 1 to less than 2 hours per week | 0.28 | 0.321 | -0.38, 0.94 | 0.4 |  |
| 40-49 yrs \* 1 to less than 2 hours per week | 0.10 | 0.339 | -0.59, 0.80 | 0.8 |  |
| 50-59 yrs \* 1 to less than 2 hours per week | 0.22 | 0.350 | -0.50, 0.94 | 0.5 |  |
| 60-69 yrs \* 1 to less than 2 hours per week | 0.51 | 0.349 | -0.20, 1.2 | 0.2 |  |
| 70-79 yrs \* 1 to less than 2 hours per week | 0.15 | 0.394 | -0.66, 0.96 | 0.7 |  |
| 80+ \* 1 to less than 2 hours per week | -0.60 | 0.395 | -1.4, 0.21 | 0.14 |  |
| 30-39 yrs \* 2 to less than 4 hours per week | 0.20 | 0.320 | -0.46, 0.86 | 0.5 |  |
| 40-49 yrs \* 2 to less than 4 hours per week | 0.10 | 0.357 | -0.63, 0.84 | 0.8 |  |
| 50-59 yrs \* 2 to less than 4 hours per week | 0.12 | 0.353 | -0.60, 0.85 | 0.7 |  |
| 60-69 yrs \* 2 to less than 4 hours per week | 0.62 | 0.337 | -0.08, 1.3 | 0.079 |  |
| 70-79 yrs \* 2 to less than 4 hours per week | 0.05 | 0.384 | -0.74, 0.84 | >0.9 |  |
| 80+ \* 2 to less than 4 hours per week | -0.51 | 0.491 | -1.5, 0.50 | 0.3 |  |
| 30-39 yrs \* 4 hours per week and more | 0.08 | 0.376 | -0.69, 0.86 | 0.8 |  |
| 40-49 yrs \* 4 hours per week and more | -0.07 | 0.393 | -0.88, 0.74 | 0.9 |  |
| 50-59 yrs \* 4 hours per week and more | 0.39 | 0.417 | -0.47, 1.2 | 0.4 |  |
| 60-69 yrs \* 4 hours per week and more | 1.1 | 0.392 | 0.32, 1.9 | 0.008 |  |
| 70-79 yrs \* 4 hours per week and more | 0.47 | 0.489 | -0.54, 1.5 | 0.3 |  |
| 80+ \* 4 hours per week and more | 0.53 | 0.700 | -0.91, 2.0 | 0.5 |  |
| Age \* Waited for medical examination date in the last 12 months |  |  |  |  | 0.382 |
| 30-39 yrs \* No | 0.10 | 0.210 | -0.34, 0.53 | 0.7 |  |
| 40-49 yrs \* No | -0.35 | 0.225 | -0.82, 0.11 | 0.13 |  |
| 50-59 yrs \* No | 0.20 | 0.220 | -0.25, 0.65 | 0.4 |  |
| 60-69 yrs \* No | -0.22 | 0.259 | -0.76, 0.31 | 0.4 |  |
| 70-79 yrs \* No | 0.10 | 0.315 | -0.55, 0.75 | 0.8 |  |
| 80+ \* No | 0.35 | 0.324 | -0.31, 1.0 | 0.3 |  |
| 30-39 yrs \* No need for examination or treatment | 0.15 | 0.233 | -0.33, 0.63 | 0.5 |  |
| 40-49 yrs \* No need for examination or treatment | -0.13 | 0.292 | -0.73, 0.47 | 0.7 |  |
| 50-59 yrs \* No need for examination or treatment | 0.42 | 0.328 | -0.26, 1.1 | 0.2 |  |
| 60-69 yrs \* No need for examination or treatment | -0.08 | 0.354 | -0.81, 0.65 | 0.8 |  |
| 70-79 yrs \* No need for examination or treatment | 0.32 | 0.537 | -0.78, 1.4 | 0.6 |  |
| 80+ \* No need for examination or treatment | 1.3 | 0.780 | -0.27, 2.9 | 0.10 |  |
| Age \* Interview type |  |  |  |  | 0.366 |
| 30-39 yrs \* Paper and Pencil Interview | 0.02 | 0.402 | -0.81, 0.85 | >0.9 |  |
| 40-49 yrs \* Paper and Pencil Interview | -0.56 | 0.373 | -1.3, 0.21 | 0.15 |  |
| 50-59 yrs \* Paper and Pencil Interview | -0.08 | 0.363 | -0.82, 0.67 | 0.8 |  |
| 60-69 yrs \* Paper and Pencil Interview | -0.09 | 0.348 | -0.81, 0.62 | 0.8 |  |
| 70-79 yrs \* Paper and Pencil Interview | 0.31 | 0.386 | -0.49, 1.1 | 0.4 |  |
| 80+ \* Paper and Pencil Interview | -0.05 | 0.431 | -0.94, 0.84 | >0.9 |  |
| Age \* BIK community size (categorized) |  |  |  |  | 0.166 |
| 30-39 yrs \* BIK region 20,000 to <50,000 inhabitants OR surroundings 50,000 to <500,000 inhabitants | -0.63 | 0.395 | -1.4, 0.18 | 0.12 |  |
| 40-49 yrs \* BIK region 20,000 to <50,000 inhabitants OR surroundings 50,000 to <500,000 inhabitants | -1.0 | 0.443 | -1.9, -0.09 | 0.033 |  |
| 50-59 yrs \* BIK region 20,000 to <50,000 inhabitants OR surroundings 50,000 to <500,000 inhabitants | -0.39 | 0.375 | -1.2, 0.38 | 0.3 |  |
| 60-69 yrs \* BIK region 20,000 to <50,000 inhabitants OR surroundings 50,000 to <500,000 inhabitants | -0.58 | 0.412 | -1.4, 0.27 | 0.2 |  |
| 70-79 yrs \* BIK region 20,000 to <50,000 inhabitants OR surroundings 50,000 to <500,000 inhabitants | -0.39 | 0.443 | -1.3, 0.53 | 0.4 |  |
| 80+ \* BIK region 20,000 to <50,000 inhabitants OR surroundings 50,000 to <500,000 inhabitants | -0.16 | 0.560 | -1.3, 0.99 | 0.8 |  |
| 30-39 yrs \* Core city 50,000 to <500,000 inhabitants OR surroundings 500,000+ inhabitants | -0.20 | 0.399 | -1.0, 0.62 | 0.6 |  |
| 40-49 yrs \* Core city 50,000 to <500,000 inhabitants OR surroundings 500,000+ inhabitants | -0.44 | 0.457 | -1.4, 0.51 | 0.3 |  |
| 50-59 yrs \* Core city 50,000 to <500,000 inhabitants OR surroundings 500,000+ inhabitants | 0.29 | 0.391 | -0.51, 1.1 | 0.5 |  |
| 60-69 yrs \* Core city 50,000 to <500,000 inhabitants OR surroundings 500,000+ inhabitants | -0.49 | 0.453 | -1.4, 0.45 | 0.3 |  |
| 70-79 yrs \* Core city 50,000 to <500,000 inhabitants OR surroundings 500,000+ inhabitants | -0.32 | 0.448 | -1.2, 0.60 | 0.5 |  |
| 80+ \* Core city 50,000 to <500,000 inhabitants OR surroundings 500,000+ inhabitants | 0.24 | 0.560 | -0.91, 1.4 | 0.7 |  |
| 30-39 yrs \* Core city 500,000+ inhabitants | -0.61 | 0.393 | -1.4, 0.19 | 0.13 |  |
| 40-49 yrs \* Core city 500,000+ inhabitants | -0.66 | 0.446 | -1.6, 0.26 | 0.2 |  |
| 50-59 yrs \* Core city 500,000+ inhabitants | -0.46 | 0.375 | -1.2, 0.31 | 0.2 |  |
| 60-69 yrs \* Core city 500,000+ inhabitants | -0.64 | 0.427 | -1.5, 0.24 | 0.14 |  |
| 70-79 yrs \* Core city 500,000+ inhabitants | -0.41 | 0.488 | -1.4, 0.59 | 0.4 |  |
| 80+ \* Core city 500,000+ inhabitants | -0.28 | 0.565 | -1.4, 0.89 | 0.6 |  |
| Age \* Regions |  |  |  |  | 0.011 |
| 30-39 yrs \* Northwest | -0.43 | 0.289 | -1.0, 0.17 | 0.15 |  |
| 40-49 yrs \* Northwest | 0.02 | 0.358 | -0.72, 0.76 | >0.9 |  |
| 50-59 yrs \* Northwest | -0.27 | 0.335 | -0.96, 0.42 | 0.4 |  |
| 60-69 yrs \* Northwest | -0.01 | 0.484 | -1.0, 0.99 | >0.9 |  |
| 70-79 yrs \* Northwest | -0.44 | 0.544 | -1.6, 0.68 | 0.4 |  |
| 80+ \* Northwest | 0.39 | 0.508 | -0.66, 1.4 | 0.4 |  |
| 30-39 yrs \* Central-East | -0.54 | 0.380 | -1.3, 0.24 | 0.2 |  |
| 40-49 yrs \* Central-East | -0.19 | 0.405 | -1.0, 0.64 | 0.6 |  |
| 50-59 yrs \* Central-East | -0.22 | 0.471 | -1.2, 0.75 | 0.6 |  |
| 60-69 yrs \* Central-East | 0.42 | 0.460 | -0.53, 1.4 | 0.4 |  |
| 70-79 yrs \* Central-East | -1.1 | 0.608 | -2.3, 0.18 | 0.089 |  |
| 80+ \* Central-East | 0.00 | 0.615 | -1.3, 1.3 | >0.9 |  |
| 30-39 yrs \* Central-West | -0.33 | 0.296 | -0.94, 0.28 | 0.3 |  |
| 40-49 yrs \* Central-West | 0.18 | 0.327 | -0.50, 0.85 | 0.6 |  |
| 50-59 yrs \* Central-West | -0.09 | 0.313 | -0.74, 0.55 | 0.8 |  |
| 60-69 yrs \* Central-West | -0.22 | 0.374 | -0.99, 0.55 | 0.6 |  |
| 70-79 yrs \* Central-West | -0.28 | 0.528 | -1.4, 0.81 | 0.6 |  |
| 80+ \* Central-West | -0.08 | 0.465 | -1.0, 0.88 | 0.9 |  |
| 30-39 yrs \* South | 0.18 | 0.301 | -0.44, 0.79 | 0.6 |  |
| 40-49 yrs \* South | 0.10 | 0.321 | -0.56, 0.77 | 0.7 |  |
| 50-59 yrs \* South | -0.05 | 0.330 | -0.73, 0.63 | 0.9 |  |
| 60-69 yrs \* South | -0.05 | 0.385 | -0.85, 0.74 | 0.9 |  |
| 70-79 yrs \* South | -0.51 | 0.518 | -1.6, 0.55 | 0.3 |  |
| 80+ \* South | -0.49 | 0.501 | -1.5, 0.54 | 0.3 |  |
| Age \* Country of birth |  |  |  |  | 0.192 |
| 30-39 yrs \* In another country | 0.21 | 0.366 | -0.55, 0.96 | 0.6 |  |
| 40-49 yrs \* In another country | -0.59 | 0.375 | -1.4, 0.18 | 0.13 |  |
| 50-59 yrs \* In another country | 0.44 | 0.414 | -0.41, 1.3 | 0.3 |  |
| 60-69 yrs \* In another country | -0.19 | 0.429 | -1.1, 0.70 | 0.7 |  |
| 70-79 yrs \* In another country | -0.30 | 0.543 | -1.4, 0.82 | 0.6 |  |
| 80+ \* In another country | -0.01 | 0.419 | -0.88, 0.85 | >0.9 |  |
| Age \* German nationality |  |  |  |  | 0.004 |
| 30-39 yrs \* No | -0.32 | 0.444 | -1.2, 0.59 | 0.5 |  |
| 40-49 yrs \* No | 0.46 | 0.462 | -0.49, 1.4 | 0.3 |  |
| 50-59 yrs \* No | -0.85 | 0.439 | -1.7, 0.06 | 0.065 |  |
| 60-69 yrs \* No | -1.4 | 0.578 | -2.6, -0.24 | 0.020 |  |
| 70-79 yrs \* No | -0.38 | 0.648 | -1.7, 0.95 | 0.6 |  |
| 80+ \* No | 0.02 | 1.03 | -2.1, 2.1 | >0.9 |  |
| Age \* Household size |  |  |  |  | 0.848 |
| 30-39 yrs \* Multi-person household | 0.20 | 0.264 | -0.34, 0.75 | 0.5 |  |
| 40-49 yrs \* Multi-person household | 0.05 | 0.269 | -0.50, 0.61 | 0.8 |  |
| 50-59 yrs \* Multi-person household | -0.07 | 0.263 | -0.61, 0.47 | 0.8 |  |
| 60-69 yrs \* Multi-person household | 0.18 | 0.259 | -0.36, 0.71 | 0.5 |  |
| 70-79 yrs \* Multi-person household | 0.28 | 0.304 | -0.34, 0.91 | 0.4 |  |
| 80+ \* Multi-person household | 0.29 | 0.291 | -0.31, 0.89 | 0.3 |  |
| Age \* Education: ISCED (2011) |  |  |  |  | 0.587 |
| 30-39 yrs \* Medium | -0.13 | 0.371 | -0.90, 0.63 | 0.7 |  |
| 40-49 yrs \* Medium | -0.61 | 0.333 | -1.3, 0.08 | 0.080 |  |
| 50-59 yrs \* Medium | -0.09 | 0.363 | -0.84, 0.66 | 0.8 |  |
| 60-69 yrs \* Medium | -0.16 | 0.317 | -0.82, 0.49 | 0.6 |  |
| 70-79 yrs \* Medium | 0.14 | 0.345 | -0.57, 0.85 | 0.7 |  |
| 80+ \* Medium | 0.35 | 0.348 | -0.37, 1.1 | 0.3 |  |
| 30-39 yrs \* High | -0.09 | 0.379 | -0.87, 0.69 | 0.8 |  |
| 40-49 yrs \* High | -0.60 | 0.347 | -1.3, 0.11 | 0.095 |  |
| 50-59 yrs \* High | 0.00 | 0.373 | -0.77, 0.77 | >0.9 |  |
| 60-69 yrs \* High | 0.02 | 0.359 | -0.72, 0.76 | >0.9 |  |
| 70-79 yrs \* High | 0.21 | 0.431 | -0.68, 1.1 | 0.6 |  |
| 80+ \* High | 0.69 | 0.402 | -0.14, 1.5 | 0.10 |  |
| Sex \* Self-rated mental health |  |  |  |  | 0.083 |
| Female \* fair/poor | -0.29 | 0.167 | -0.63, 0.05 | 0.10 |  |
| Sex \* Sausage products |  |  |  |  | 0.179 |
| Female \* 4 to 6 times per week | -0.22 | 0.241 | -0.72, 0.28 | 0.4 |  |
| Female \* 1 to 3 times per week | -0.49 | 0.223 | -0.95, -0.03 | 0.039 |  |
| Female \* Less than once per week | -0.25 | 0.247 | -0.76, 0.25 | 0.3 |  |
| Female \* Never | -0.31 | 0.301 | -0.93, 0.31 | 0.3 |  |
| Sex \* Red meat |  |  |  |  | 0.275 |
| Female \* 4 to 6 times per week | 0.18 | 0.509 | -0.87, 1.2 | 0.7 |  |
| Female \* 1 to 3 times per week | -0.22 | 0.466 | -1.2, 0.73 | 0.6 |  |
| Female \* Less than once per week | -0.22 | 0.478 | -1.2, 0.76 | 0.6 |  |
| Female \* Never | 0.22 | 0.539 | -0.89, 1.3 | 0.7 |  |
| Sex \* BIK community size (categorized) |  |  |  |  | 0.138 |
| Female \* BIK region 20,000 to <50,000 inhabitants OR surroundings 50,000 to <500,000 inhabitants | 0.47 | 0.209 | 0.04, 0.90 | 0.034 |  |
| Female \* Core city 50,000 to <500,000 inhabitants OR surroundings 500,000+ inhabitants | 0.23 | 0.201 | -0.18, 0.65 | 0.3 |  |
| Female \* Core city 500,000+ inhabitants | 0.27 | 0.205 | -0.15, 0.69 | 0.2 |  |
| Sex \* Regions |  |  |  |  | <0.001 |
| Female \* Northwest | -0.09 | 0.223 | -0.55, 0.37 | 0.7 |  |
| Female \* Central-East | -0.10 | 0.239 | -0.59, 0.40 | 0.7 |  |
| Female \* Central-West | -0.63 | 0.194 | -1.0, -0.22 | 0.004 |  |
| Female \* South | -0.28 | 0.208 | -0.71, 0.15 | 0.2 |  |
| Sex \* German nationality |  |  |  |  | 0.017 |
| Female \* No | -0.54 | 0.226 | -1.0, -0.08 | 0.025 |  |
| Sex \* Education: ISCED (2011) |  |  |  |  | 0.133 |
| Female \* Medium | 0.20 | 0.183 | -0.17, 0.58 | 0.3 |  |
| Female \* High | -0.05 | 0.189 | -0.44, 0.34 | 0.8 |  |
| Education: CASMIN \* Health risk due to climate change |  |  |  |  | 0.142 |
| Medium \* Scale value 4 to 7 | 0.19 | 0.175 | -0.17, 0.55 | 0.3 |  |
| High \* Scale value 4 to 7 | 0.24 | 0.209 | -0.19, 0.67 | 0.3 |  |
| Medium \* Scale value 8 to 10 | 0.41 | 0.238 | -0.08, 0.90 | 0.10 |  |
| High \* Scale value 8 to 10 | 0.05 | 0.248 | -0.46, 0.56 | 0.8 |  |
| Education: CASMIN \* Smoking |  |  |  |  | 0.077 |
| Medium \* Occasional smoking | -0.92 | 0.331 | -1.6, -0.24 | 0.010 |  |
| High \* Occasional smoking | -0.94 | 0.429 | -1.8, -0.05 | 0.038 |  |
| Medium \* Non-smoker | -0.19 | 0.189 | -0.58, 0.20 | 0.3 |  |
| High \* Non-smoker | -0.33 | 0.271 | -0.88, 0.23 | 0.2 |  |
| Education: CASMIN \* Sausage products |  |  |  |  | 0.842 |
| Medium \* 4 to 6 times per week | -0.19 | 0.324 | -0.86, 0.48 | 0.6 |  |
| High \* 4 to 6 times per week | -0.26 | 0.362 | -1.0, 0.48 | 0.5 |  |
| Medium \* 1 to 3 times per week | 0.02 | 0.304 | -0.61, 0.65 | >0.9 |  |
| High \* 1 to 3 times per week | -0.16 | 0.334 | -0.85, 0.53 | 0.6 |  |
| Medium \* Less than once per week | -0.09 | 0.332 | -0.77, 0.60 | 0.8 |  |
| High \* Less than once per week | 0.01 | 0.363 | -0.74, 0.75 | >0.9 |  |
| Medium \* Never | 0.30 | 0.423 | -0.57, 1.2 | 0.5 |  |
| High \* Never | 0.17 | 0.456 | -0.77, 1.1 | 0.7 |  |
| Education: CASMIN \* Red meat |  |  |  |  | 0.431 |
| Medium \* 4 to 6 times per week | 0.04 | 0.497 | -0.99, 1.1 | >0.9 |  |
| High \* 4 to 6 times per week | 0.12 | 0.554 | -1.0, 1.3 | 0.8 |  |
| Medium \* 1 to 3 times per week | -0.15 | 0.443 | -1.1, 0.76 | 0.7 |  |
| High \* 1 to 3 times per week | -0.11 | 0.515 | -1.2, 0.95 | 0.8 |  |
| Medium \* Less than once per week | -0.26 | 0.475 | -1.2, 0.72 | 0.6 |  |
| High \* Less than once per week | -0.31 | 0.535 | -1.4, 0.79 | 0.6 |  |
| Medium \* Never | -0.97 | 0.578 | -2.2, 0.22 | 0.10 |  |
| High \* Never | -0.84 | 0.633 | -2.1, 0.46 | 0.2 |  |
| Education: CASMIN \* Sport |  |  |  |  | 0.294 |
| Medium \* Less than 1 hour per week | -0.39 | 0.230 | -0.87, 0.08 | 0.10 |  |
| High \* Less than 1 hour per week | -0.48 | 0.285 | -1.1, 0.10 | 0.10 |  |
| Medium \* 1 to less than 2 hours per week | -0.47 | 0.226 | -0.94, -0.01 | 0.048 |  |
| High \* 1 to less than 2 hours per week | -0.36 | 0.275 | -0.93, 0.20 | 0.2 |  |
| Medium \* 2 to less than 4 hours per week | -0.64 | 0.270 | -1.2, -0.08 | 0.027 |  |
| High \* 2 to less than 4 hours per week | -0.69 | 0.297 | -1.3, -0.08 | 0.028 |  |
| Medium \* 4 hours per week and more | -0.50 | 0.317 | -1.2, 0.15 | 0.12 |  |
| High \* 4 hours per week and more | -0.35 | 0.344 | -1.1, 0.36 | 0.3 |  |
| Education: CASMIN \* BIK community size (categorized) |  |  |  |  | 0.051 |
| Medium \* BIK region 20,000 to <50,000 inhabitants OR surroundings 50,000 to <500,000 inhabitants | -0.18 | 0.283 | -0.76, 0.40 | 0.5 |  |
| High \* BIK region 20,000 to <50,000 inhabitants OR surroundings 50,000 to <500,000 inhabitants | 0.67 | 0.292 | 0.06, 1.3 | 0.032 |  |
| Medium \* Core city 50,000 to <500,000 inhabitants OR surroundings 500,000+ inhabitants | -0.21 | 0.283 | -0.79, 0.37 | 0.5 |  |
| High \* Core city 50,000 to <500,000 inhabitants OR surroundings 500,000+ inhabitants | 0.49 | 0.289 | -0.11, 1.1 | 0.10 |  |
| Medium \* Core city 500,000+ inhabitants | -0.13 | 0.302 | -0.75, 0.49 | 0.7 |  |
| High \* Core city 500,000+ inhabitants | 0.77 | 0.296 | 0.16, 1.4 | 0.015 |  |
| Education: CASMIN \* Regions |  |  |  |  | 0.059 |
| Medium \* Northwest | 0.04 | 0.311 | -0.60, 0.69 | 0.9 |  |
| High \* Northwest | -0.28 | 0.363 | -1.0, 0.47 | 0.5 |  |
| Medium \* Central-East | 0.82 | 0.375 | 0.05, 1.6 | 0.038 |  |
| High \* Central-East | 0.82 | 0.377 | 0.04, 1.6 | 0.040 |  |
| Medium \* Central-West | 0.21 | 0.290 | -0.38, 0.81 | 0.5 |  |
| High \* Central-West | -0.10 | 0.337 | -0.79, 0.59 | 0.8 |  |
| Medium \* South | 0.06 | 0.292 | -0.55, 0.66 | 0.8 |  |
| High \* South | -0.14 | 0.322 | -0.80, 0.53 | 0.7 |  |
| Education: CASMIN \* Current living situation |  |  |  |  | 0.669 |
| Medium \* Part-time employed | 0.25 | 0.246 | -0.26, 0.75 | 0.3 |  |
| High \* Part-time employed | 0.24 | 0.283 | -0.34, 0.82 | 0.4 |  |
| Medium \* Unemployed | 0.44 | 0.347 | -0.28, 1.2 | 0.2 |  |
| High \* Unemployed | 0.29 | 0.426 | -0.59, 1.2 | 0.5 |  |
| Medium \* Retired or early retired | 0.27 | 0.218 | -0.18, 0.72 | 0.2 |  |
| High \* Retired or early retired | 0.20 | 0.283 | -0.38, 0.79 | 0.5 |  |
| Medium \* Not employed for other reasons (student, volunteer service, homemaker) | 0.55 | 0.273 | -0.01, 1.1 | 0.054 |  |
| High \* Not employed for other reasons (student, volunteer service, homemaker) | 0.61 | 0.318 | -0.05, 1.3 | 0.067 |  |
| N.Obs | 11,845 |  |  |  |  |
| N.Cluster | 359 |  |  |  |  |
|  |  |  |  |  |  |
| --- | --- | --- | --- | --- | --- |
| Abbreviations: CI = Confidence Interval, OR = Odds Ratio, SE = Standard Error | | | | | |

### Quarter 3

| Characteristic | log(OR) | SE | 95% CI | p-value | p-value (global) |
| --- | --- | --- | --- | --- | --- |
| Sex |  |  |  |  | 0.675 |
| Male | — | — | — |  |  |
| Female | 0.24 | 0.576 |  |  |  |
| Age |  |  |  |  | 0.344 |
| 16-29 yrs | — | — | — |  |  |
| 30-39 yrs | -0.76 | 0.888 |  |  |  |
| 40-49 yrs | 0.22 | 0.928 |  |  |  |
| 50-59 yrs | 0.01 | 0.912 |  |  |  |
| 60-69 yrs | -1.3 | 1.04 |  |  |  |
| 70-79 yrs | 1.5 | 1.35 |  |  |  |
| 80+ | -1.8 | 1.39 |  |  |  |
| Education: CASMIN |  |  |  |  | 0.071 |
| Low | — | — | — |  |  |
| Medium | 1.8 | 0.776 |  |  |  |
| High | 1.3 | 0.978 |  |  |  |
| Obesity |  |  |  |  | >0.9 |
| Yes | — | — | — |  |  |
| No | 0.01 | 0.162 |  |  |  |
| Self-rated health |  |  |  |  | 0.247 |
| Very good/good/fair | — | — | — |  |  |
| Bad/very bad | -0.61 | 0.528 |  |  |  |
| Chronic diseases |  |  |  |  | 0.159 |
| Yes | — | — | — |  |  |
| No | -0.19 | 0.134 |  |  |  |
| Satisfaction: Life in general |  |  |  |  | 0.044 |
| Scale value 1 to 3 | — | — | — |  |  |
| Scale value 4 to 7 | -0.49 | 0.222 |  |  |  |
| Scale value 8 to 10 | -0.29 | 0.244 |  |  |  |
| Health risk due to climate change |  |  |  |  | >0.9 |
| Scale value 1 to 3 | — | — | — |  |  |
| Scale value 4 to 7 | 0.06 | 0.205 |  |  |  |
| Scale value 8 to 10 | 0.04 | 0.264 |  |  |  |
| Smoking |  |  |  |  | <0.001 |
| Daily smoking | — | — | — |  |  |
| Occasional smoking | 0.50 | 0.378 |  |  |  |
| Non-smoker | 1.1 | 0.246 |  |  |  |
| Red meat |  |  |  |  | 0.465 |
| Daily or several times a day | — | — | — |  |  |
| 4 to 6 times per week | 0.11 | 0.564 |  |  |  |
| 1 to 3 times per week | -0.27 | 0.522 |  |  |  |
| Less than once per week | -0.46 | 0.538 |  |  |  |
| Never | -0.52 | 0.599 |  |  |  |
| Sport |  |  |  |  | 0.195 |
| No sporting activities | — | — | — |  |  |
| Less than 1 hour per week | 0.34 | 0.272 |  |  |  |
| 1 to less than 2 hours per week | 0.01 | 0.272 |  |  |  |
| 2 to less than 4 hours per week | 0.58 | 0.315 |  |  |  |
| 4 hours per week and more | 0.38 | 0.312 |  |  |  |
| Interview type |  |  |  |  | 0.323 |
| Computer Assisted Web Interview | — | — | — |  |  |
| Paper and Pencil Interview | 0.28 | 0.280 |  |  |  |
| Foreign nationality |  |  |  |  | 0.084 |
| Foreign national | — | — | — |  |  |
| Not foreign national | -0.40 | 0.230 |  |  |  |
| BIK community size (categorized) |  |  |  |  | 0.222 |
| BIK region <20,000 inhabitants | — | — | — |  |  |
| BIK region 20,000 to <50,000 inhabitants OR surroundings 50,000 to <500,000 inhabitants | 0.59 | 0.333 |  |  |  |
| Core city 50,000 to <500,000 inhabitants OR surroundings 500,000+ inhabitants | 0.26 | 0.349 |  |  |  |
| Core city 500,000+ inhabitants | 0.25 | 0.350 |  |  |  |
| Regions |  |  |  |  | 0.66 |
| Northeast | — | — | — |  |  |
| Northwest | -0.13 | 0.342 |  |  |  |
| Central-East | -0.36 | 0.457 |  |  |  |
| Central-West | 0.06 | 0.356 |  |  |  |
| South | 0.13 | 0.342 |  |  |  |
| Country of birth |  |  |  |  | 0.861 |
| In Germany (within current borders) | — | — | — |  |  |
| In another country | 0.05 | 0.283 |  |  |  |
| German nationality |  |  |  |  | 0.013 |
| Yes | — | — | — |  |  |
| No | -0.98 | 0.395 |  |  |  |
| Education: ISCED (2011) |  |  |  |  | 0.45 |
| Low | — | — | — |  |  |
| Medium | 0.17 | 0.168 |  |  |  |
| High | 0.27 | 0.220 |  |  |  |
| Current living situation |  |  |  |  | 0.072 |
| Full-time employed | — | — | — |  |  |
| Part-time employed | -0.26 | 0.213 |  |  |  |
| Unemployed | -0.39 | 0.272 |  |  |  |
| Retired or early retired | 0.30 | 0.202 |  |  |  |
| Not employed for other reasons (student, volunteer service, homemaker) | -0.01 | 0.229 |  |  |  |
| Overweight |  |  |  |  | 0.175 |
| Yes | — | — | — |  |  |
| No | 0.61 | 0.446 |  |  |  |
| Paying attention to health |  |  |  |  | 0.515 |
| Not at all/less strong/moderate | — | — | — |  |  |
| Strong/very strong | -0.09 | 0.136 |  |  |  |
| Self-rated mental health |  |  |  |  | 0.852 |
| Excellent/very good/good | — | — | — |  |  |
| fair/poor | 0.03 | 0.158 |  |  |  |
| Sausage products |  |  |  |  | 0.401 |
| Daily or several times a day | — | — | — |  |  |
| 4 to 6 times per week | 0.52 | 0.361 |  |  |  |
| 1 to 3 times per week | 0.47 | 0.332 |  |  |  |
| Less than once per week | 0.63 | 0.359 |  |  |  |
| Never | 0.79 | 0.447 |  |  |  |
| Waited for medical examination date in the last 12 months |  |  |  |  | >0.9 |
| Yes | — | — | — |  |  |
| No | -0.05 | 0.223 |  |  |  |
| No need for examination or treatment | -0.02 | 0.307 |  |  |  |
| Household size |  |  |  |  | 0.225 |
| Single-person household | — | — | — |  |  |
| Multi-person household | -0.29 | 0.238 |  |  |  |
| Normal weight |  |  |  |  | 0.083 |
| Normal weight (18.5 <= BMI < 25) | — | — | — |  |  |
| Not normal weight (BMI < 18.5 or BMI >= 25) | 0.75 | 0.432 |  |  |  |
| Age \* Overweight |  |  |  |  | 0.059 |
| 30-39 yrs \* No | -0.06 | 0.182 |  |  |  |
| 40-49 yrs \* No | -0.28 | 0.203 |  |  |  |
| 50-59 yrs \* No | -0.18 | 0.192 |  |  |  |
| 60-69 yrs \* No | 0.43 | 0.220 |  |  |  |
| 70-79 yrs \* No | -0.23 | 0.250 |  |  |  |
| 80+ \* No | 0.15 | 0.289 |  |  |  |
| Age \* Self-rated health |  |  |  |  | <0.001 |
| 30-39 yrs \* Bad/very bad | 1.1 | 0.593 |  |  |  |
| 40-49 yrs \* Bad/very bad | 1.3 | 0.645 |  |  |  |
| 50-59 yrs \* Bad/very bad | 0.60 | 0.551 |  |  |  |
| 60-69 yrs \* Bad/very bad | 0.74 | 0.568 |  |  |  |
| 70-79 yrs \* Bad/very bad | -0.22 | 0.599 |  |  |  |
| 80+ \* Bad/very bad | -0.71 | 0.606 |  |  |  |
| Age \* Chronic diseases |  |  |  |  | 0.688 |
| 30-39 yrs \* No | -0.01 | 0.199 |  |  |  |
| 40-49 yrs \* No | 0.20 | 0.210 |  |  |  |
| 50-59 yrs \* No | 0.11 | 0.176 |  |  |  |
| 60-69 yrs \* No | -0.03 | 0.236 |  |  |  |
| 70-79 yrs \* No | 0.00 | 0.268 |  |  |  |
| 80+ \* No | -0.40 | 0.333 |  |  |  |
| Age \* Satisfaction: Life in general |  |  |  |  | 0.249 |
| 30-39 yrs \* Scale value 4 to 7 | 0.89 | 0.360 |  |  |  |
| 40-49 yrs \* Scale value 4 to 7 | 0.32 | 0.380 |  |  |  |
| 50-59 yrs \* Scale value 4 to 7 | 0.77 | 0.352 |  |  |  |
| 60-69 yrs \* Scale value 4 to 7 | 0.71 | 0.375 |  |  |  |
| 70-79 yrs \* Scale value 4 to 7 | 0.10 | 0.520 |  |  |  |
| 80+ \* Scale value 4 to 7 | 0.51 | 0.560 |  |  |  |
| 30-39 yrs \* Scale value 8 to 10 | 0.79 | 0.392 |  |  |  |
| 40-49 yrs \* Scale value 8 to 10 | 0.27 | 0.416 |  |  |  |
| 50-59 yrs \* Scale value 8 to 10 | 0.64 | 0.367 |  |  |  |
| 60-69 yrs \* Scale value 8 to 10 | 0.86 | 0.414 |  |  |  |
| 70-79 yrs \* Scale value 8 to 10 | 0.18 | 0.553 |  |  |  |
| 80+ \* Scale value 8 to 10 | 0.88 | 0.609 |  |  |  |
| Age \* Health risk due to climate change |  |  |  |  | 0.031 |
| 30-39 yrs \* Scale value 4 to 7 | 0.27 | 0.201 |  |  |  |
| 40-49 yrs \* Scale value 4 to 7 | 0.24 | 0.228 |  |  |  |
| 50-59 yrs \* Scale value 4 to 7 | -0.34 | 0.210 |  |  |  |
| 60-69 yrs \* Scale value 4 to 7 | 0.37 | 0.228 |  |  |  |
| 70-79 yrs \* Scale value 4 to 7 | -0.29 | 0.295 |  |  |  |
| 80+ \* Scale value 4 to 7 | 0.36 | 0.329 |  |  |  |
| 30-39 yrs \* Scale value 8 to 10 | -0.20 | 0.264 |  |  |  |
| 40-49 yrs \* Scale value 8 to 10 | -0.17 | 0.259 |  |  |  |
| 50-59 yrs \* Scale value 8 to 10 | 0.03 | 0.268 |  |  |  |
| 60-69 yrs \* Scale value 8 to 10 | 0.21 | 0.304 |  |  |  |
| 70-79 yrs \* Scale value 8 to 10 | 0.04 | 0.432 |  |  |  |
| 80+ \* Scale value 8 to 10 | 0.19 | 0.441 |  |  |  |
| Age \* Paying attention to health |  |  |  |  | 0.014 |
| 30-39 yrs \* Strong/very strong | 0.21 | 0.199 |  |  |  |
| 40-49 yrs \* Strong/very strong | 0.47 | 0.206 |  |  |  |
| 50-59 yrs \* Strong/very strong | 0.14 | 0.204 |  |  |  |
| 60-69 yrs \* Strong/very strong | -0.27 | 0.226 |  |  |  |
| 70-79 yrs \* Strong/very strong | -0.07 | 0.252 |  |  |  |
| 80+ \* Strong/very strong | 0.65 | 0.274 |  |  |  |
| Age \* Self-rated mental health |  |  |  |  | 0.82 |
| 30-39 yrs \* fair/poor | 0.06 | 0.242 |  |  |  |
| 40-49 yrs \* fair/poor | -0.13 | 0.259 |  |  |  |
| 50-59 yrs \* fair/poor | 0.16 | 0.264 |  |  |  |
| 60-69 yrs \* fair/poor | -0.31 | 0.272 |  |  |  |
| 70-79 yrs \* fair/poor | 0.03 | 0.310 |  |  |  |
| 80+ \* fair/poor | 0.08 | 0.350 |  |  |  |
| Age \* Smoking |  |  |  |  | <0.001 |
| 30-39 yrs \* Occasional smoking | -0.03 | 0.351 |  |  |  |
| 40-49 yrs \* Occasional smoking | 0.69 | 0.374 |  |  |  |
| 50-59 yrs \* Occasional smoking | 0.55 | 0.397 |  |  |  |
| 60-69 yrs \* Occasional smoking | 0.16 | 0.473 |  |  |  |
| 70-79 yrs \* Occasional smoking | 0.72 | 0.997 |  |  |  |
| 80+ \* Occasional smoking | -0.21 | 1.64 |  |  |  |
| 30-39 yrs \* Non-smoker | -0.66 | 0.250 |  |  |  |
| 40-49 yrs \* Non-smoker | -0.06 | 0.266 |  |  |  |
| 50-59 yrs \* Non-smoker | -0.25 | 0.246 |  |  |  |
| 60-69 yrs \* Non-smoker | -0.28 | 0.271 |  |  |  |
| 70-79 yrs \* Non-smoker | -1.8 | 0.482 |  |  |  |
| 80+ \* Non-smoker | -0.63 | 0.573 |  |  |  |
| Age \* Sausage products |  |  |  |  | 0.16 |
| 30-39 yrs \* 4 to 6 times per week | -0.70 | 0.370 |  |  |  |
| 40-49 yrs \* 4 to 6 times per week | -0.31 | 0.418 |  |  |  |
| 50-59 yrs \* 4 to 6 times per week | -0.49 | 0.375 |  |  |  |
| 60-69 yrs \* 4 to 6 times per week | -0.26 | 0.413 |  |  |  |
| 70-79 yrs \* 4 to 6 times per week | -0.95 | 0.471 |  |  |  |
| 80+ \* 4 to 6 times per week | 0.36 | 0.595 |  |  |  |
| 30-39 yrs \* 1 to 3 times per week | -0.22 | 0.367 |  |  |  |
| 40-49 yrs \* 1 to 3 times per week | -0.02 | 0.364 |  |  |  |
| 50-59 yrs \* 1 to 3 times per week | -0.02 | 0.350 |  |  |  |
| 60-69 yrs \* 1 to 3 times per week | -0.09 | 0.392 |  |  |  |
| 70-79 yrs \* 1 to 3 times per week | -0.65 | 0.441 |  |  |  |
| 80+ \* 1 to 3 times per week | -0.58 | 0.524 |  |  |  |
| 30-39 yrs \* Less than once per week | 0.01 | 0.373 |  |  |  |
| 40-49 yrs \* Less than once per week | 0.21 | 0.396 |  |  |  |
| 50-59 yrs \* Less than once per week | -0.14 | 0.382 |  |  |  |
| 60-69 yrs \* Less than once per week | -0.29 | 0.416 |  |  |  |
| 70-79 yrs \* Less than once per week | -0.79 | 0.475 |  |  |  |
| 80+ \* Less than once per week | -0.53 | 0.590 |  |  |  |
| 30-39 yrs \* Never | -0.52 | 0.427 |  |  |  |
| 40-49 yrs \* Never | -0.45 | 0.530 |  |  |  |
| 50-59 yrs \* Never | -0.47 | 0.536 |  |  |  |
| 60-69 yrs \* Never | -0.38 | 0.581 |  |  |  |
| 70-79 yrs \* Never | -1.4 | 0.722 |  |  |  |
| 80+ \* Never | -1.2 | 0.826 |  |  |  |
| Age \* Red meat |  |  |  |  | 0.006 |
| 30-39 yrs \* 4 to 6 times per week | 0.12 | 0.628 |  |  |  |
| 40-49 yrs \* 4 to 6 times per week | 0.18 | 0.536 |  |  |  |
| 50-59 yrs \* 4 to 6 times per week | 0.21 | 0.709 |  |  |  |
| 60-69 yrs \* 4 to 6 times per week | 1.8 | 0.733 |  |  |  |
| 70-79 yrs \* 4 to 6 times per week | 0.59 | 0.937 |  |  |  |
| 80+ \* 4 to 6 times per week | -0.59 | 0.995 |  |  |  |
| 30-39 yrs \* 1 to 3 times per week | 0.09 | 0.599 |  |  |  |
| 40-49 yrs \* 1 to 3 times per week | 0.47 | 0.488 |  |  |  |
| 50-59 yrs \* 1 to 3 times per week | 0.20 | 0.677 |  |  |  |
| 60-69 yrs \* 1 to 3 times per week | 1.5 | 0.636 |  |  |  |
| 70-79 yrs \* 1 to 3 times per week | 1.3 | 0.941 |  |  |  |
| 80+ \* 1 to 3 times per week | 1.0 | 0.928 |  |  |  |
| 30-39 yrs \* Less than once per week | -0.13 | 0.626 |  |  |  |
| 40-49 yrs \* Less than once per week | 0.10 | 0.518 |  |  |  |
| 50-59 yrs \* Less than once per week | 0.49 | 0.670 |  |  |  |
| 60-69 yrs \* Less than once per week | 1.9 | 0.675 |  |  |  |
| 70-79 yrs \* Less than once per week | 1.4 | 0.934 |  |  |  |
| 80+ \* Less than once per week | 1.3 | 0.963 |  |  |  |
| 30-39 yrs \* Never | 0.24 | 0.691 |  |  |  |
| 40-49 yrs \* Never | 0.33 | 0.635 |  |  |  |
| 50-59 yrs \* Never | 0.38 | 0.779 |  |  |  |
| 60-69 yrs \* Never | 1.2 | 0.736 |  |  |  |
| 70-79 yrs \* Never | 2.1 | 1.11 |  |  |  |
| 80+ \* Never | 0.68 | 1.00 |  |  |  |
| Age \* Sport |  |  |  |  | 0.207 |
| 30-39 yrs \* Less than 1 hour per week | 0.01 | 0.287 |  |  |  |
| 40-49 yrs \* Less than 1 hour per week | 0.26 | 0.286 |  |  |  |
| 50-59 yrs \* Less than 1 hour per week | -0.13 | 0.290 |  |  |  |
| 60-69 yrs \* Less than 1 hour per week | -0.02 | 0.345 |  |  |  |
| 70-79 yrs \* Less than 1 hour per week | -0.15 | 0.409 |  |  |  |
| 80+ \* Less than 1 hour per week | -0.50 | 0.405 |  |  |  |
| 30-39 yrs \* 1 to less than 2 hours per week | 0.02 | 0.288 |  |  |  |
| 40-49 yrs \* 1 to less than 2 hours per week | 0.12 | 0.302 |  |  |  |
| 50-59 yrs \* 1 to less than 2 hours per week | -0.09 | 0.290 |  |  |  |
| 60-69 yrs \* 1 to less than 2 hours per week | -0.33 | 0.320 |  |  |  |
| 70-79 yrs \* 1 to less than 2 hours per week | 0.07 | 0.372 |  |  |  |
| 80+ \* 1 to less than 2 hours per week | 0.23 | 0.416 |  |  |  |
| 30-39 yrs \* 2 to less than 4 hours per week | 0.11 | 0.303 |  |  |  |
| 40-49 yrs \* 2 to less than 4 hours per week | -0.39 | 0.328 |  |  |  |
| 50-59 yrs \* 2 to less than 4 hours per week | -0.15 | 0.308 |  |  |  |
| 60-69 yrs \* 2 to less than 4 hours per week | 0.38 | 0.380 |  |  |  |
| 70-79 yrs \* 2 to less than 4 hours per week | 0.04 | 0.440 |  |  |  |
| 80+ \* 2 to less than 4 hours per week | 0.26 | 0.490 |  |  |  |
| 30-39 yrs \* 4 hours per week and more | 0.13 | 0.313 |  |  |  |
| 40-49 yrs \* 4 hours per week and more | -0.08 | 0.343 |  |  |  |
| 50-59 yrs \* 4 hours per week and more | 0.11 | 0.347 |  |  |  |
| 60-69 yrs \* 4 hours per week and more | -0.42 | 0.347 |  |  |  |
| 70-79 yrs \* 4 hours per week and more | -0.11 | 0.464 |  |  |  |
| 80+ \* 4 hours per week and more | 0.13 | 0.595 |  |  |  |
| Age \* Waited for medical examination date in the last 12 months |  |  |  |  | 0.058 |
| 30-39 yrs \* No | -0.18 | 0.218 |  |  |  |
| 40-49 yrs \* No | -0.09 | 0.205 |  |  |  |
| 50-59 yrs \* No | 0.02 | 0.213 |  |  |  |
| 60-69 yrs \* No | 0.19 | 0.235 |  |  |  |
| 70-79 yrs \* No | 0.12 | 0.300 |  |  |  |
| 80+ \* No | -0.41 | 0.341 |  |  |  |
| 30-39 yrs \* No need for examination or treatment | -0.33 | 0.252 |  |  |  |
| 40-49 yrs \* No need for examination or treatment | -0.42 | 0.268 |  |  |  |
| 50-59 yrs \* No need for examination or treatment | -0.49 | 0.332 |  |  |  |
| 60-69 yrs \* No need for examination or treatment | 0.98 | 0.481 |  |  |  |
| 70-79 yrs \* No need for examination or treatment | 0.91 | 0.664 |  |  |  |
| 80+ \* No need for examination or treatment | -1.8 | 0.673 |  |  |  |
| Age \* Interview type |  |  |  |  | 0.002 |
| 30-39 yrs \* Paper and Pencil Interview | 0.01 | 0.392 |  |  |  |
| 40-49 yrs \* Paper and Pencil Interview | -0.66 | 0.387 |  |  |  |
| 50-59 yrs \* Paper and Pencil Interview | -0.48 | 0.340 |  |  |  |
| 60-69 yrs \* Paper and Pencil Interview | 0.03 | 0.350 |  |  |  |
| 70-79 yrs \* Paper and Pencil Interview | 0.44 | 0.345 |  |  |  |
| 80+ \* Paper and Pencil Interview | 0.54 | 0.467 |  |  |  |
| Age \* Foreign nationality |  |  |  |  | 0.406 |
| 30-39 yrs \* Not foreign national | -0.03 | 0.318 |  |  |  |
| 40-49 yrs \* Not foreign national | -0.24 | 0.377 |  |  |  |
| 50-59 yrs \* Not foreign national | 0.35 | 0.351 |  |  |  |
| 60-69 yrs \* Not foreign national | 0.51 | 0.421 |  |  |  |
| 70-79 yrs \* Not foreign national | -0.31 | 0.687 |  |  |  |
| 80+ \* Not foreign national | 0.68 | 0.519 |  |  |  |
| Age \* BIK community size (categorized) |  |  |  |  | 0.043 |
| 30-39 yrs \* BIK region 20,000 to <50,000 inhabitants OR surroundings 50,000 to <500,000 inhabitants | 0.04 | 0.281 |  |  |  |
| 40-49 yrs \* BIK region 20,000 to <50,000 inhabitants OR surroundings 50,000 to <500,000 inhabitants | -0.60 | 0.291 |  |  |  |
| 50-59 yrs \* BIK region 20,000 to <50,000 inhabitants OR surroundings 50,000 to <500,000 inhabitants | -0.35 | 0.336 |  |  |  |
| 60-69 yrs \* BIK region 20,000 to <50,000 inhabitants OR surroundings 50,000 to <500,000 inhabitants | -0.37 | 0.287 |  |  |  |
| 70-79 yrs \* BIK region 20,000 to <50,000 inhabitants OR surroundings 50,000 to <500,000 inhabitants | -0.03 | 0.382 |  |  |  |
| 80+ \* BIK region 20,000 to <50,000 inhabitants OR surroundings 50,000 to <500,000 inhabitants | 0.00 | 0.493 |  |  |  |
| 30-39 yrs \* Core city 50,000 to <500,000 inhabitants OR surroundings 500,000+ inhabitants | 0.63 | 0.301 |  |  |  |
| 40-49 yrs \* Core city 50,000 to <500,000 inhabitants OR surroundings 500,000+ inhabitants | -0.48 | 0.315 |  |  |  |
| 50-59 yrs \* Core city 50,000 to <500,000 inhabitants OR surroundings 500,000+ inhabitants | -0.17 | 0.346 |  |  |  |
| 60-69 yrs \* Core city 50,000 to <500,000 inhabitants OR surroundings 500,000+ inhabitants | 0.25 | 0.332 |  |  |  |
| 70-79 yrs \* Core city 50,000 to <500,000 inhabitants OR surroundings 500,000+ inhabitants | 0.50 | 0.400 |  |  |  |
| 80+ \* Core city 50,000 to <500,000 inhabitants OR surroundings 500,000+ inhabitants | 0.70 | 0.538 |  |  |  |
| 30-39 yrs \* Core city 500,000+ inhabitants | 0.00 | 0.303 |  |  |  |
| 40-49 yrs \* Core city 500,000+ inhabitants | -0.62 | 0.308 |  |  |  |
| 50-59 yrs \* Core city 500,000+ inhabitants | -0.22 | 0.346 |  |  |  |
| 60-69 yrs \* Core city 500,000+ inhabitants | 0.01 | 0.343 |  |  |  |
| 70-79 yrs \* Core city 500,000+ inhabitants | -0.16 | 0.378 |  |  |  |
| 80+ \* Core city 500,000+ inhabitants | 0.35 | 0.517 |  |  |  |
| Age \* Regions |  |  |  |  | >0.9 |
| 30-39 yrs \* Northwest | -0.02 | 0.278 |  |  |  |
| 40-49 yrs \* Northwest | 0.16 | 0.317 |  |  |  |
| 50-59 yrs \* Northwest | 0.24 | 0.324 |  |  |  |
| 60-69 yrs \* Northwest | 0.13 | 0.360 |  |  |  |
| 70-79 yrs \* Northwest | 0.13 | 0.406 |  |  |  |
| 80+ \* Northwest | -0.04 | 0.488 |  |  |  |
| 30-39 yrs \* Central-East | -0.01 | 0.362 |  |  |  |
| 40-49 yrs \* Central-East | 0.06 | 0.391 |  |  |  |
| 50-59 yrs \* Central-East | 0.24 | 0.364 |  |  |  |
| 60-69 yrs \* Central-East | -0.11 | 0.447 |  |  |  |
| 70-79 yrs \* Central-East | -0.03 | 0.470 |  |  |  |
| 80+ \* Central-East | 1.1 | 0.632 |  |  |  |
| 30-39 yrs \* Central-West | -0.06 | 0.261 |  |  |  |
| 40-49 yrs \* Central-West | -0.05 | 0.305 |  |  |  |
| 50-59 yrs \* Central-West | 0.16 | 0.275 |  |  |  |
| 60-69 yrs \* Central-West | -0.11 | 0.339 |  |  |  |
| 70-79 yrs \* Central-West | -0.10 | 0.380 |  |  |  |
| 80+ \* Central-West | 0.25 | 0.439 |  |  |  |
| 30-39 yrs \* South | -0.26 | 0.265 |  |  |  |
| 40-49 yrs \* South | -0.17 | 0.324 |  |  |  |
| 50-59 yrs \* South | -0.10 | 0.309 |  |  |  |
| 60-69 yrs \* South | -0.26 | 0.354 |  |  |  |
| 70-79 yrs \* South | -0.25 | 0.417 |  |  |  |
| 80+ \* South | 0.44 | 0.486 |  |  |  |
| Age \* Country of birth |  |  |  |  | 0.097 |
| 30-39 yrs \* In another country | -0.07 | 0.383 |  |  |  |
| 40-49 yrs \* In another country | -0.81 | 0.372 |  |  |  |
| 50-59 yrs \* In another country | 0.00 | 0.420 |  |  |  |
| 60-69 yrs \* In another country | -0.61 | 0.445 |  |  |  |
| 70-79 yrs \* In another country | -0.51 | 0.556 |  |  |  |
| 80+ \* In another country | 0.11 | 0.464 |  |  |  |
| Age \* German nationality |  |  |  |  | >0.9 |
| 30-39 yrs \* No | -0.07 | 0.486 |  |  |  |
| 40-49 yrs \* No | 0.17 | 0.571 |  |  |  |
| 50-59 yrs \* No | 0.14 | 0.623 |  |  |  |
| 60-69 yrs \* No | 0.38 | 0.679 |  |  |  |
| 70-79 yrs \* No | -0.02 | 0.920 |  |  |  |
| 80+ \* No | -0.52 | 1.07 |  |  |  |
| Age \* Household size |  |  |  |  | 0.456 |
| 30-39 yrs \* Multi-person household | 0.27 | 0.225 |  |  |  |
| 40-49 yrs \* Multi-person household | 0.01 | 0.255 |  |  |  |
| 50-59 yrs \* Multi-person household | 0.06 | 0.247 |  |  |  |
| 60-69 yrs \* Multi-person household | 0.27 | 0.273 |  |  |  |
| 70-79 yrs \* Multi-person household | 0.39 | 0.312 |  |  |  |
| 80+ \* Multi-person household | 0.57 | 0.298 |  |  |  |
| Age \* Education: ISCED (2011) |  |  |  |  | 0.065 |
| 30-39 yrs \* Medium | 0.77 | 0.349 |  |  |  |
| 40-49 yrs \* Medium | 0.67 | 0.352 |  |  |  |
| 50-59 yrs \* Medium | 0.58 | 0.318 |  |  |  |
| 60-69 yrs \* Medium | -0.10 | 0.308 |  |  |  |
| 70-79 yrs \* Medium | 0.54 | 0.330 |  |  |  |
| 80+ \* Medium | -0.29 | 0.334 |  |  |  |
| 30-39 yrs \* High | 0.76 | 0.359 |  |  |  |
| 40-49 yrs \* High | 0.45 | 0.392 |  |  |  |
| 50-59 yrs \* High | 0.36 | 0.341 |  |  |  |
| 60-69 yrs \* High | 0.00 | 0.353 |  |  |  |
| 70-79 yrs \* High | 0.63 | 0.366 |  |  |  |
| 80+ \* High | 0.34 | 0.399 |  |  |  |
| Sex \* Self-rated health |  |  |  |  | 0.708 |
| Female \* Bad/very bad | 0.09 | 0.244 |  |  |  |
| Sex \* Health risk due to climate change |  |  |  |  | 0.175 |
| Female \* Scale value 4 to 7 | 0.12 | 0.124 |  |  |  |
| Female \* Scale value 8 to 10 | -0.18 | 0.181 |  |  |  |
| Sex \* Smoking |  |  |  |  | 0.491 |
| Female \* Occasional smoking | 0.20 | 0.240 |  |  |  |
| Female \* Non-smoker | -0.07 | 0.161 |  |  |  |
| Sex \* Red meat |  |  |  |  | 0.731 |
| Female \* 4 to 6 times per week | -0.27 | 0.540 |  |  |  |
| Female \* 1 to 3 times per week | -0.06 | 0.529 |  |  |  |
| Female \* Less than once per week | -0.22 | 0.525 |  |  |  |
| Female \* Never | -0.18 | 0.549 |  |  |  |
| Sex \* Waited for medical examination date in the last 12 months |  |  |  |  | 0.023 |
| Female \* No | -0.09 | 0.129 |  |  |  |
| Female \* No need for examination or treatment | 0.41 | 0.187 |  |  |  |
| Sex \* BIK community size (categorized) |  |  |  |  | 0.57 |
| Female \* BIK region 20,000 to <50,000 inhabitants OR surroundings 50,000 to <500,000 inhabitants | 0.12 | 0.194 |  |  |  |
| Female \* Core city 50,000 to <500,000 inhabitants OR surroundings 500,000+ inhabitants | 0.27 | 0.203 |  |  |  |
| Female \* Core city 500,000+ inhabitants | 0.18 | 0.187 |  |  |  |
| Sex \* Regions |  |  |  |  | 0.397 |
| Female \* Northwest | 0.29 | 0.204 |  |  |  |
| Female \* Central-East | 0.13 | 0.231 |  |  |  |
| Female \* Central-West | -0.07 | 0.181 |  |  |  |
| Female \* South | 0.08 | 0.185 |  |  |  |
| Education: CASMIN \* Obesity |  |  |  |  | 0.748 |
| Medium \* No | 0.14 | 0.202 |  |  |  |
| High \* No | 0.15 | 0.241 |  |  |  |
| Education: CASMIN \* Overweight |  |  |  |  | 0.245 |
| Medium \* No | -0.84 | 0.503 |  |  |  |
| High \* No | -0.63 | 0.619 |  |  |  |
| Education: CASMIN \* Self-rated health |  |  |  |  | 0.262 |
| Medium \* Bad/very bad | -0.22 | 0.272 |  |  |  |
| High \* Bad/very bad | -0.58 | 0.354 |  |  |  |
| Education: CASMIN \* Health risk due to climate change |  |  |  |  | 0.37 |
| Medium \* Scale value 4 to 7 | -0.04 | 0.176 |  |  |  |
| High \* Scale value 4 to 7 | -0.20 | 0.196 |  |  |  |
| Medium \* Scale value 8 to 10 | 0.30 | 0.244 |  |  |  |
| High \* Scale value 8 to 10 | 0.24 | 0.268 |  |  |  |
| Education: CASMIN \* Smoking |  |  |  |  | 0.03 |
| Medium \* Occasional smoking | -0.48 | 0.339 |  |  |  |
| High \* Occasional smoking | -0.89 | 0.428 |  |  |  |
| Medium \* Non-smoker | -0.44 | 0.191 |  |  |  |
| High \* Non-smoker | -0.27 | 0.251 |  |  |  |
| Education: CASMIN \* Sausage products |  |  |  |  | 0.061 |
| Medium \* 4 to 6 times per week | -0.06 | 0.295 |  |  |  |
| High \* 4 to 6 times per week | -0.39 | 0.340 |  |  |  |
| Medium \* 1 to 3 times per week | -0.48 | 0.272 |  |  |  |
| High \* 1 to 3 times per week | -0.83 | 0.338 |  |  |  |
| Medium \* Less than once per week | -0.64 | 0.304 |  |  |  |
| High \* Less than once per week | -1.2 | 0.346 |  |  |  |
| Medium \* Never | -0.45 | 0.405 |  |  |  |
| High \* Never | -0.97 | 0.490 |  |  |  |
| Education: CASMIN \* Red meat |  |  |  |  | 0.438 |
| Medium \* 4 to 6 times per week | -0.49 | 0.468 |  |  |  |
| High \* 4 to 6 times per week | -0.01 | 0.600 |  |  |  |
| Medium \* 1 to 3 times per week | -0.06 | 0.436 |  |  |  |
| High \* 1 to 3 times per week | 0.32 | 0.559 |  |  |  |
| Medium \* Less than once per week | 0.22 | 0.452 |  |  |  |
| High \* Less than once per week | 0.62 | 0.570 |  |  |  |
| Medium \* Never | 0.33 | 0.526 |  |  |  |
| High \* Never | 0.77 | 0.649 |  |  |  |
| Education: CASMIN \* Sport |  |  |  |  | 0.075 |
| Medium \* Less than 1 hour per week | -0.47 | 0.229 |  |  |  |
| High \* Less than 1 hour per week | -0.35 | 0.281 |  |  |  |
| Medium \* 1 to less than 2 hours per week | 0.08 | 0.207 |  |  |  |
| High \* 1 to less than 2 hours per week | -0.07 | 0.267 |  |  |  |
| Medium \* 2 to less than 4 hours per week | -0.41 | 0.265 |  |  |  |
| High \* 2 to less than 4 hours per week | -0.51 | 0.310 |  |  |  |
| Medium \* 4 hours per week and more | -0.55 | 0.275 |  |  |  |
| High \* 4 hours per week and more | -0.26 | 0.337 |  |  |  |
| Education: CASMIN \* Waited for medical examination date in the last 12 months |  |  |  |  | 0.346 |
| Medium \* No | 0.20 | 0.175 |  |  |  |
| High \* No | 0.38 | 0.201 |  |  |  |
| Medium \* No need for examination or treatment | 0.11 | 0.295 |  |  |  |
| High \* No need for examination or treatment | 0.40 | 0.305 |  |  |  |
| Education: CASMIN \* BIK community size (categorized) |  |  |  |  | 0.564 |
| Medium \* BIK region 20,000 to <50,000 inhabitants OR surroundings 50,000 to <500,000 inhabitants | -0.23 | 0.253 |  |  |  |
| High \* BIK region 20,000 to <50,000 inhabitants OR surroundings 50,000 to <500,000 inhabitants | 0.10 | 0.262 |  |  |  |
| Medium \* Core city 50,000 to <500,000 inhabitants OR surroundings 500,000+ inhabitants | -0.39 | 0.278 |  |  |  |
| High \* Core city 50,000 to <500,000 inhabitants OR surroundings 500,000+ inhabitants | -0.15 | 0.280 |  |  |  |
| Medium \* Core city 500,000+ inhabitants | -0.11 | 0.282 |  |  |  |
| High \* Core city 500,000+ inhabitants | 0.20 | 0.259 |  |  |  |
| Education: CASMIN \* Regions |  |  |  |  | 0.364 |
| Medium \* Northwest | 0.11 | 0.306 |  |  |  |
| High \* Northwest | 0.01 | 0.328 |  |  |  |
| Medium \* Central-East | 0.48 | 0.454 |  |  |  |
| High \* Central-East | 0.64 | 0.462 |  |  |  |
| Medium \* Central-West | 0.06 | 0.288 |  |  |  |
| High \* Central-West | -0.08 | 0.326 |  |  |  |
| Medium \* South | 0.18 | 0.278 |  |  |  |
| High \* South | 0.39 | 0.309 |  |  |  |
| Education: CASMIN \* Household size |  |  |  |  | 0.253 |
| Medium \* Multi-person household | 0.30 | 0.192 |  |  |  |
| High \* Multi-person household | 0.17 | 0.226 |  |  |  |
| Education: CASMIN \* Current living situation |  |  |  |  | 0.524 |
| Medium \* Part-time employed | 0.43 | 0.236 |  |  |  |
| High \* Part-time employed | 0.45 | 0.244 |  |  |  |
| Medium \* Unemployed | -0.04 | 0.327 |  |  |  |
| High \* Unemployed | 0.32 | 0.414 |  |  |  |
| Medium \* Retired or early retired | 0.16 | 0.220 |  |  |  |
| High \* Retired or early retired | 0.06 | 0.279 |  |  |  |
| Medium \* Not employed for other reasons (student, volunteer service, homemaker) | 0.12 | 0.241 |  |  |  |
| High \* Not employed for other reasons (student, volunteer service, homemaker) | -0.12 | 0.312 |  |  |  |
| Education: CASMIN \* Normal weight |  |  |  |  | 0.075 |
| Medium \* Not normal weight (BMI < 18.5 or BMI >= 25) | -1.1 | 0.502 |  |  |  |
| High \* Not normal weight (BMI < 18.5 or BMI >= 25) | -0.74 | 0.621 |  |  |  |
| N.Obs | 11,806 |  |  |  |  |
| N.Cluster | 359 |  |  |  |  |
|  |  |  |  |  |  |
| --- | --- | --- | --- | --- | --- |
| Abbreviations: CI = Confidence Interval, OR = Odds Ratio, SE = Standard Error | | | | | |

### Quarter 4

| Characteristic | log(OR) | SE | 95% CI | p-value | p-value (global) |
| --- | --- | --- | --- | --- | --- |
| Sex |  |  |  |  | 0.566 |
| Male | — | — | — |  |  |
| Female | -0.23 | 0.399 | -1.0, 0.56 | 0.6 |  |
| Age |  |  |  |  | 0.444 |
| 16-29 yrs | — | — | — |  |  |
| 30-39 yrs | -0.60 | 0.510 | -1.6, 0.41 | 0.2 |  |
| 40-49 yrs | -0.46 | 0.551 | -1.5, 0.63 | 0.4 |  |
| 50-59 yrs | 0.14 | 0.490 | -0.83, 1.1 | 0.8 |  |
| 60-69 yrs | 0.05 | 0.544 | -1.0, 1.1 | >0.9 |  |
| 70-79 yrs | 0.00 | 0.632 | -1.2, 1.3 | >0.9 |  |
| 80+ | -1.4 | 0.687 | -2.7, 0.00 | 0.050 |  |
| Education: CASMIN |  |  |  |  | 0.138 |
| Low | — | — | — |  |  |
| Medium | -0.26 | 0.572 | -1.4, 0.87 | 0.7 |  |
| High | 0.98 | 0.760 | -0.52, 2.5 | 0.2 |  |
| Smoking |  |  |  |  | 0.253 |
| Daily smoking | — | — | — |  |  |
| Occasional smoking | 0.17 | 0.328 | -0.48, 0.81 | 0.6 |  |
| Non-smoker | 0.26 | 0.158 | -0.05, 0.57 | 0.10 |  |
| Sport |  |  |  |  | >0.9 |
| No sporting activities | — | — | — |  |  |
| Less than 1 hour per week | -0.18 | 0.248 | -0.67, 0.31 | 0.5 |  |
| 1 to less than 2 hours per week | -0.05 | 0.219 | -0.48, 0.38 | 0.8 |  |
| 2 to less than 4 hours per week | -0.16 | 0.241 | -0.64, 0.32 | 0.5 |  |
| 4 hours per week and more | -0.09 | 0.204 | -0.50, 0.31 | 0.6 |  |
| BIK community size (categorized) |  |  |  |  | 0.063 |
| BIK region <20,000 inhabitants | — | — | — |  |  |
| BIK region 20,000 to <50,000 inhabitants OR surroundings 50,000 to <500,000 inhabitants | -0.76 | 0.297 | -1.4, -0.18 | 0.011 |  |
| Core city 50,000 to <500,000 inhabitants OR surroundings 500,000+ inhabitants | -0.39 | 0.309 | -1.0, 0.22 | 0.2 |  |
| Core city 500,000+ inhabitants | -0.57 | 0.303 | -1.2, 0.03 | 0.064 |  |
| Country of birth |  |  |  |  | 0.567 |
| In Germany (within current borders) | — | — | — |  |  |
| In another country | -0.19 | 0.332 | -0.84, 0.47 | 0.6 |  |
| German nationality |  |  |  |  | 0.223 |
| Yes | — | — | — |  |  |
| No | -0.45 | 0.367 | -1.2, 0.28 | 0.2 |  |
| Education: ISCED (2011) |  |  |  |  | 0.211 |
| Low | — | — | — |  |  |
| Medium | -0.25 | 0.197 | -0.63, 0.14 | 0.2 |  |
| High | 0.00 | 0.228 | -0.45, 0.45 | >0.9 |  |
| Current living situation |  |  |  |  | 0.009 |
| Full-time employed | — | — | — |  |  |
| Part-time employed | -0.12 | 0.227 | -0.57, 0.33 | 0.6 |  |
| Unemployed | -0.22 | 0.255 | -0.72, 0.29 | 0.4 |  |
| Retired or early retired | 0.58 | 0.204 | 0.18, 0.98 | 0.005 |  |
| Not employed for other reasons (student, volunteer service, homemaker) | 0.17 | 0.279 | -0.39, 0.72 | 0.6 |  |
| Self-rated health |  |  |  |  | >0.9 |
| Very good/good/fair | — | — | — |  |  |
| Bad/very bad | 0.01 | 0.487 | -0.95, 0.98 | >0.9 |  |
| Interview type |  |  |  |  | 0.04 |
| Computer Assisted Web Interview | — | — | — |  |  |
| Paper and Pencil Interview | -0.53 | 0.258 | -1.0, -0.02 | 0.042 |  |
| Regions |  |  |  |  | 0.365 |
| Northeast | — | — | — |  |  |
| Northwest | 0.36 | 0.353 | -0.34, 1.1 | 0.3 |  |
| Central-East | -0.11 | 0.417 | -0.93, 0.72 | 0.8 |  |
| Central-West | 0.47 | 0.314 | -0.15, 1.1 | 0.14 |  |
| South | 0.16 | 0.325 | -0.48, 0.80 | 0.6 |  |
| Normal weight |  |  |  |  | 0.347 |
| Normal weight (18.5 <= BMI < 25) | — | — | — |  |  |
| Not normal weight (BMI < 18.5 or BMI >= 25) | -0.19 | 0.206 | -0.60, 0.21 | 0.3 |  |
| Red meat |  |  |  |  | 0.144 |
| Daily or several times a day | — | — | — |  |  |
| 4 to 6 times per week | -0.72 | 0.401 | -1.5, 0.07 | 0.075 |  |
| 1 to 3 times per week | -0.55 | 0.353 | -1.2, 0.15 | 0.12 |  |
| Less than once per week | -0.22 | 0.361 | -0.94, 0.49 | 0.5 |  |
| Never | -0.62 | 0.444 | -1.5, 0.26 | 0.2 |  |
| Obesity |  |  |  |  | 0.271 |
| Yes | — | — | — |  |  |
| No | 0.17 | 0.157 | -0.14, 0.48 | 0.3 |  |
| Overweight |  |  |  |  | 0.659 |
| Yes | — | — | — |  |  |
| No | -0.11 | 0.242 | -0.58, 0.37 | 0.7 |  |
| Satisfaction: Life in general |  |  |  |  | 0.663 |
| Scale value 1 to 3 | — | — | — |  |  |
| Scale value 4 to 7 | 0.20 | 0.219 | -0.24, 0.63 | 0.4 |  |
| Scale value 8 to 10 | 0.17 | 0.215 | -0.25, 0.60 | 0.4 |  |
| Sausage products |  |  |  |  | 0.203 |
| Daily or several times a day | — | — | — |  |  |
| 4 to 6 times per week | 0.25 | 0.232 | -0.20, 0.71 | 0.3 |  |
| 1 to 3 times per week | 0.36 | 0.206 | -0.04, 0.77 | 0.081 |  |
| Less than once per week | 0.43 | 0.249 | -0.07, 0.92 | 0.089 |  |
| Never | -0.03 | 0.344 | -0.71, 0.65 | >0.9 |  |
| Foreign nationality |  |  |  |  | 0.782 |
| Foreign national | — | — | — |  |  |
| Not foreign national | -0.06 | 0.231 | -0.52, 0.39 | 0.8 |  |
| Age \* Self-rated health |  |  |  |  | 0.073 |
| 30-39 yrs \* Bad/very bad | 1.2 | 0.727 | -0.24, 2.6 | 0.10 |  |
| 40-49 yrs \* Bad/very bad | 0.80 | 0.720 | -0.63, 2.2 | 0.3 |  |
| 50-59 yrs \* Bad/very bad | -0.44 | 0.557 | -1.5, 0.66 | 0.4 |  |
| 60-69 yrs \* Bad/very bad | -0.53 | 0.532 | -1.6, 0.53 | 0.3 |  |
| 70-79 yrs \* Bad/very bad | -0.27 | 0.569 | -1.4, 0.86 | 0.6 |  |
| 80+ \* Bad/very bad | -0.31 | 0.575 | -1.4, 0.82 | 0.6 |  |
| Age \* Sport |  |  |  |  | 0.031 |
| 30-39 yrs \* Less than 1 hour per week | 0.34 | 0.328 | -0.30, 0.99 | 0.3 |  |
| 40-49 yrs \* Less than 1 hour per week | 0.38 | 0.322 | -0.25, 1.0 | 0.2 |  |
| 50-59 yrs \* Less than 1 hour per week | 0.08 | 0.348 | -0.61, 0.77 | 0.8 |  |
| 60-69 yrs \* Less than 1 hour per week | 0.07 | 0.357 | -0.63, 0.78 | 0.8 |  |
| 70-79 yrs \* Less than 1 hour per week | 0.15 | 0.432 | -0.71, 1.0 | 0.7 |  |
| 80+ \* Less than 1 hour per week | 1.1 | 0.430 | 0.22, 1.9 | 0.014 |  |
| 30-39 yrs \* 1 to less than 2 hours per week | 0.23 | 0.321 | -0.41, 0.86 | 0.5 |  |
| 40-49 yrs \* 1 to less than 2 hours per week | 0.33 | 0.331 | -0.32, 0.99 | 0.3 |  |
| 50-59 yrs \* 1 to less than 2 hours per week | 0.29 | 0.290 | -0.28, 0.87 | 0.3 |  |
| 60-69 yrs \* 1 to less than 2 hours per week | 0.38 | 0.328 | -0.27, 1.0 | 0.2 |  |
| 70-79 yrs \* 1 to less than 2 hours per week | 0.30 | 0.374 | -0.44, 1.0 | 0.4 |  |
| 80+ \* 1 to less than 2 hours per week | 1.1 | 0.356 | 0.42, 1.8 | 0.002 |  |
| 30-39 yrs \* 2 to less than 4 hours per week | 0.26 | 0.340 | -0.41, 0.93 | 0.4 |  |
| 40-49 yrs \* 2 to less than 4 hours per week | 0.64 | 0.351 | -0.06, 1.3 | 0.071 |  |
| 50-59 yrs \* 2 to less than 4 hours per week | -0.02 | 0.344 | -0.70, 0.66 | >0.9 |  |
| 60-69 yrs \* 2 to less than 4 hours per week | 0.58 | 0.370 | -0.15, 1.3 | 0.12 |  |
| 70-79 yrs \* 2 to less than 4 hours per week | 1.0 | 0.489 | 0.08, 2.0 | 0.035 |  |
| 80+ \* 2 to less than 4 hours per week | 1.1 | 0.419 | 0.25, 1.9 | 0.011 |  |
| 30-39 yrs \* 4 hours per week and more | 0.23 | 0.337 | -0.44, 0.90 | 0.5 |  |
| 40-49 yrs \* 4 hours per week and more | 0.38 | 0.342 | -0.30, 1.1 | 0.3 |  |
| 50-59 yrs \* 4 hours per week and more | -0.08 | 0.332 | -0.74, 0.58 | 0.8 |  |
| 60-69 yrs \* 4 hours per week and more | 0.81 | 0.406 | 0.00, 1.6 | 0.049 |  |
| 70-79 yrs \* 4 hours per week and more | 0.55 | 0.506 | -0.45, 1.5 | 0.3 |  |
| 80+ \* 4 hours per week and more | 0.92 | 0.504 | -0.08, 1.9 | 0.071 |  |
| Age \* Interview type |  |  |  |  | 0.066 |
| 30-39 yrs \* Paper and Pencil Interview | 0.90 | 0.381 | 0.15, 1.7 | 0.019 |  |
| 40-49 yrs \* Paper and Pencil Interview | 1.1 | 0.397 | 0.36, 1.9 | 0.005 |  |
| 50-59 yrs \* Paper and Pencil Interview | 0.40 | 0.341 | -0.27, 1.1 | 0.2 |  |
| 60-69 yrs \* Paper and Pencil Interview | 0.73 | 0.336 | 0.07, 1.4 | 0.031 |  |
| 70-79 yrs \* Paper and Pencil Interview | 0.63 | 0.361 | -0.08, 1.3 | 0.081 |  |
| 80+ \* Paper and Pencil Interview | 0.94 | 0.432 | 0.09, 1.8 | 0.031 |  |
| Age \* BIK community size (categorized) |  |  |  |  | 0.608 |
| 30-39 yrs \* BIK region 20,000 to <50,000 inhabitants OR surroundings 50,000 to <500,000 inhabitants | 0.36 | 0.277 | -0.19, 0.91 | 0.2 |  |
| 40-49 yrs \* BIK region 20,000 to <50,000 inhabitants OR surroundings 50,000 to <500,000 inhabitants | 0.16 | 0.347 | -0.53, 0.85 | 0.6 |  |
| 50-59 yrs \* BIK region 20,000 to <50,000 inhabitants OR surroundings 50,000 to <500,000 inhabitants | 0.43 | 0.291 | -0.15, 1.0 | 0.14 |  |
| 60-69 yrs \* BIK region 20,000 to <50,000 inhabitants OR surroundings 50,000 to <500,000 inhabitants | 0.39 | 0.349 | -0.30, 1.1 | 0.3 |  |
| 70-79 yrs \* BIK region 20,000 to <50,000 inhabitants OR surroundings 50,000 to <500,000 inhabitants | 0.67 | 0.491 | -0.30, 1.6 | 0.2 |  |
| 80+ \* BIK region 20,000 to <50,000 inhabitants OR surroundings 50,000 to <500,000 inhabitants | 0.53 | 0.430 | -0.32, 1.4 | 0.2 |  |
| 30-39 yrs \* Core city 50,000 to <500,000 inhabitants OR surroundings 500,000+ inhabitants | 0.17 | 0.283 | -0.39, 0.73 | 0.6 |  |
| 40-49 yrs \* Core city 50,000 to <500,000 inhabitants OR surroundings 500,000+ inhabitants | 0.13 | 0.348 | -0.55, 0.82 | 0.7 |  |
| 50-59 yrs \* Core city 50,000 to <500,000 inhabitants OR surroundings 500,000+ inhabitants | 0.09 | 0.311 | -0.53, 0.70 | 0.8 |  |
| 60-69 yrs \* Core city 50,000 to <500,000 inhabitants OR surroundings 500,000+ inhabitants | -0.01 | 0.371 | -0.74, 0.72 | >0.9 |  |
| 70-79 yrs \* Core city 50,000 to <500,000 inhabitants OR surroundings 500,000+ inhabitants | 0.20 | 0.519 | -0.83, 1.2 | 0.7 |  |
| 80+ \* Core city 50,000 to <500,000 inhabitants OR surroundings 500,000+ inhabitants | 0.18 | 0.442 | -0.70, 1.1 | 0.7 |  |
| 30-39 yrs \* Core city 500,000+ inhabitants | 0.34 | 0.289 | -0.23, 0.91 | 0.2 |  |
| 40-49 yrs \* Core city 500,000+ inhabitants | 0.29 | 0.342 | -0.38, 0.97 | 0.4 |  |
| 50-59 yrs \* Core city 500,000+ inhabitants | 0.07 | 0.295 | -0.51, 0.65 | 0.8 |  |
| 60-69 yrs \* Core city 500,000+ inhabitants | 0.20 | 0.348 | -0.49, 0.89 | 0.6 |  |
| 70-79 yrs \* Core city 500,000+ inhabitants | -0.27 | 0.505 | -1.3, 0.73 | 0.6 |  |
| 80+ \* Core city 500,000+ inhabitants | 0.40 | 0.459 | -0.50, 1.3 | 0.4 |  |
| Age \* Regions |  |  |  |  | 0.66 |
| 30-39 yrs \* Northwest | -0.15 | 0.265 | -0.68, 0.37 | 0.6 |  |
| 40-49 yrs \* Northwest | -0.01 | 0.327 | -0.66, 0.63 | >0.9 |  |
| 50-59 yrs \* Northwest | 0.33 | 0.303 | -0.27, 0.93 | 0.3 |  |
| 60-69 yrs \* Northwest | 0.46 | 0.341 | -0.22, 1.1 | 0.2 |  |
| 70-79 yrs \* Northwest | 0.26 | 0.382 | -0.49, 1.0 | 0.5 |  |
| 80+ \* Northwest | -0.15 | 0.416 | -0.97, 0.67 | 0.7 |  |
| 30-39 yrs \* Central-East | 0.11 | 0.347 | -0.57, 0.80 | 0.7 |  |
| 40-49 yrs \* Central-East | 0.55 | 0.397 | -0.24, 1.3 | 0.2 |  |
| 50-59 yrs \* Central-East | 0.09 | 0.366 | -0.63, 0.82 | 0.8 |  |
| 60-69 yrs \* Central-East | 0.45 | 0.416 | -0.38, 1.3 | 0.3 |  |
| 70-79 yrs \* Central-East | 0.14 | 0.515 | -0.88, 1.2 | 0.8 |  |
| 80+ \* Central-East | 0.42 | 0.529 | -0.62, 1.5 | 0.4 |  |
| 30-39 yrs \* Central-West | 0.00 | 0.254 | -0.50, 0.51 | >0.9 |  |
| 40-49 yrs \* Central-West | 0.44 | 0.312 | -0.17, 1.1 | 0.2 |  |
| 50-59 yrs \* Central-West | 0.36 | 0.253 | -0.14, 0.86 | 0.2 |  |
| 60-69 yrs \* Central-West | 0.33 | 0.322 | -0.30, 0.97 | 0.3 |  |
| 70-79 yrs \* Central-West | 0.37 | 0.370 | -0.36, 1.1 | 0.3 |  |
| 80+ \* Central-West | -0.34 | 0.385 | -1.1, 0.42 | 0.4 |  |
| 30-39 yrs \* South | 0.02 | 0.261 | -0.49, 0.54 | >0.9 |  |
| 40-49 yrs \* South | 0.23 | 0.318 | -0.40, 0.85 | 0.5 |  |
| 50-59 yrs \* South | 0.49 | 0.283 | -0.07, 1.0 | 0.089 |  |
| 60-69 yrs \* South | 0.55 | 0.325 | -0.09, 1.2 | 0.090 |  |
| 70-79 yrs \* South | 0.56 | 0.417 | -0.26, 1.4 | 0.2 |  |
| 80+ \* South | 0.35 | 0.470 | -0.58, 1.3 | 0.5 |  |
| Age \* Country of birth |  |  |  |  | 0.19 |
| 30-39 yrs \* In another country | 0.28 | 0.377 | -0.47, 1.0 | 0.5 |  |
| 40-49 yrs \* In another country | -0.24 | 0.367 | -0.96, 0.49 | 0.5 |  |
| 50-59 yrs \* In another country | -0.58 | 0.399 | -1.4, 0.21 | 0.15 |  |
| 60-69 yrs \* In another country | -0.65 | 0.475 | -1.6, 0.29 | 0.2 |  |
| 70-79 yrs \* In another country | -0.67 | 0.474 | -1.6, 0.26 | 0.2 |  |
| 80+ \* In another country | -0.03 | 0.451 | -0.92, 0.86 | >0.9 |  |
| Age \* German nationality |  |  |  |  | 0.343 |
| 30-39 yrs \* No | -0.28 | 0.433 | -1.1, 0.58 | 0.5 |  |
| 40-49 yrs \* No | -0.23 | 0.466 | -1.2, 0.69 | 0.6 |  |
| 50-59 yrs \* No | 0.08 | 0.534 | -0.97, 1.1 | 0.9 |  |
| 60-69 yrs \* No | 1.2 | 0.626 | -0.06, 2.4 | 0.061 |  |
| 70-79 yrs \* No | -0.12 | 0.665 | -1.4, 1.2 | 0.9 |  |
| 80+ \* No | 0.47 | 0.804 | -1.1, 2.1 | 0.6 |  |
| Age \* Education: ISCED (2011) |  |  |  |  | 0.437 |
| 30-39 yrs \* Medium | 0.50 | 0.350 | -0.19, 1.2 | 0.2 |  |
| 40-49 yrs \* Medium | 0.47 | 0.380 | -0.29, 1.2 | 0.2 |  |
| 50-59 yrs \* Medium | 0.68 | 0.347 | 0.00, 1.4 | 0.051 |  |
| 60-69 yrs \* Medium | 0.46 | 0.347 | -0.22, 1.1 | 0.2 |  |
| 70-79 yrs \* Medium | 0.87 | 0.365 | 0.14, 1.6 | 0.019 |  |
| 80+ \* Medium | 0.82 | 0.338 | 0.15, 1.5 | 0.017 |  |
| 30-39 yrs \* High | 0.18 | 0.358 | -0.53, 0.89 | 0.6 |  |
| 40-49 yrs \* High | 0.21 | 0.399 | -0.57, 1.0 | 0.6 |  |
| 50-59 yrs \* High | 0.54 | 0.371 | -0.20, 1.3 | 0.15 |  |
| 60-69 yrs \* High | 0.31 | 0.362 | -0.40, 1.0 | 0.4 |  |
| 70-79 yrs \* High | 0.54 | 0.416 | -0.28, 1.4 | 0.2 |  |
| 80+ \* High | 0.60 | 0.412 | -0.21, 1.4 | 0.15 |  |
| Age \* Normal weight |  |  |  |  | 0.312 |
| 30-39 yrs \* Not normal weight (BMI < 18.5 or BMI >= 25) | 0.03 | 0.185 | -0.34, 0.39 | 0.9 |  |
| 40-49 yrs \* Not normal weight (BMI < 18.5 or BMI >= 25) | -0.07 | 0.187 | -0.44, 0.29 | 0.7 |  |
| 50-59 yrs \* Not normal weight (BMI < 18.5 or BMI >= 25) | -0.20 | 0.203 | -0.60, 0.20 | 0.3 |  |
| 60-69 yrs \* Not normal weight (BMI < 18.5 or BMI >= 25) | 0.15 | 0.231 | -0.30, 0.61 | 0.5 |  |
| 70-79 yrs \* Not normal weight (BMI < 18.5 or BMI >= 25) | 0.12 | 0.305 | -0.48, 0.73 | 0.7 |  |
| 80+ \* Not normal weight (BMI < 18.5 or BMI >= 25) | 0.44 | 0.248 | -0.05, 0.93 | 0.080 |  |
| Sex \* Red meat |  |  |  |  | 0.001 |
| Female \* 4 to 6 times per week | 0.70 | 0.413 | -0.12, 1.5 | 0.093 |  |
| Female \* 1 to 3 times per week | 0.25 | 0.396 | -0.54, 1.0 | 0.5 |  |
| Female \* Less than once per week | 0.08 | 0.400 | -0.71, 0.87 | 0.8 |  |
| Female \* Never | 0.87 | 0.418 | 0.04, 1.7 | 0.040 |  |
| Sex \* Regions |  |  |  |  | 0.049 |
| Female \* Northwest | 0.14 | 0.195 | -0.25, 0.52 | 0.5 |  |
| Female \* Central-East | 0.36 | 0.195 | -0.02, 0.75 | 0.066 |  |
| Female \* Central-West | -0.06 | 0.159 | -0.37, 0.26 | 0.7 |  |
| Female \* South | 0.31 | 0.164 | -0.02, 0.63 | 0.062 |  |
| Education: CASMIN \* Obesity |  |  |  |  | 0.356 |
| Medium \* No | -0.03 | 0.181 | -0.39, 0.33 | 0.9 |  |
| High \* No | -0.31 | 0.247 | -0.80, 0.18 | 0.2 |  |
| Education: CASMIN \* Overweight |  |  |  |  | 0.354 |
| Medium \* No | -0.13 | 0.191 | -0.51, 0.24 | 0.5 |  |
| High \* No | 0.07 | 0.205 | -0.33, 0.48 | 0.7 |  |
| Education: CASMIN \* Satisfaction: Life in general |  |  |  |  | 0.898 |
| Medium \* Scale value 4 to 7 | -0.03 | 0.256 | -0.54, 0.48 | >0.9 |  |
| High \* Scale value 4 to 7 | -0.27 | 0.302 | -0.87, 0.33 | 0.4 |  |
| Medium \* Scale value 8 to 10 | 0.02 | 0.251 | -0.47, 0.52 | >0.9 |  |
| High \* Scale value 8 to 10 | -0.19 | 0.297 | -0.78, 0.40 | 0.5 |  |
| Education: CASMIN \* Smoking |  |  |  |  | 0.74 |
| Medium \* Occasional smoking | -0.01 | 0.373 | -0.74, 0.73 | >0.9 |  |
| High \* Occasional smoking | -0.24 | 0.398 | -1.0, 0.55 | 0.6 |  |
| Medium \* Non-smoker | 0.18 | 0.179 | -0.17, 0.54 | 0.3 |  |
| High \* Non-smoker | -0.01 | 0.235 | -0.47, 0.46 | >0.9 |  |
| Education: CASMIN \* Sausage products |  |  |  |  | 0.235 |
| Medium \* 4 to 6 times per week | -0.12 | 0.279 | -0.68, 0.43 | 0.7 |  |
| High \* 4 to 6 times per week | -0.52 | 0.357 | -1.2, 0.18 | 0.15 |  |
| Medium \* 1 to 3 times per week | -0.31 | 0.256 | -0.82, 0.20 | 0.2 |  |
| High \* 1 to 3 times per week | -0.50 | 0.303 | -1.1, 0.10 | 0.10 |  |
| Medium \* Less than once per week | -0.53 | 0.300 | -1.1, 0.06 | 0.080 |  |
| High \* Less than once per week | -0.72 | 0.360 | -1.4, -0.01 | 0.046 |  |
| Medium \* Never | 0.28 | 0.424 | -0.56, 1.1 | 0.5 |  |
| High \* Never | -0.31 | 0.457 | -1.2, 0.59 | 0.5 |  |
| Education: CASMIN \* Red meat |  |  |  |  | 0.192 |
| Medium \* 4 to 6 times per week | 1.0 | 0.444 | 0.15, 1.9 | 0.022 |  |
| High \* 4 to 6 times per week | 0.44 | 0.557 | -0.66, 1.5 | 0.4 |  |
| Medium \* 1 to 3 times per week | 0.84 | 0.405 | 0.04, 1.6 | 0.040 |  |
| High \* 1 to 3 times per week | 0.76 | 0.505 | -0.24, 1.8 | 0.14 |  |
| Medium \* Less than once per week | 0.78 | 0.412 | -0.04, 1.6 | 0.061 |  |
| High \* Less than once per week | 0.56 | 0.519 | -0.46, 1.6 | 0.3 |  |
| Medium \* Never | 0.72 | 0.509 | -0.28, 1.7 | 0.2 |  |
| High \* Never | 0.76 | 0.596 | -0.41, 1.9 | 0.2 |  |
| Education: CASMIN \* Foreign nationality |  |  |  |  | 0.753 |
| Medium \* Not foreign national | -0.09 | 0.245 | -0.57, 0.40 | 0.7 |  |
| High \* Not foreign national | 0.06 | 0.280 | -0.49, 0.62 | 0.8 |  |
| Education: CASMIN \* BIK community size (categorized) |  |  |  |  | 0.286 |
| Medium \* BIK region 20,000 to <50,000 inhabitants OR surroundings 50,000 to <500,000 inhabitants | 0.45 | 0.242 | -0.03, 0.93 | 0.063 |  |
| High \* BIK region 20,000 to <50,000 inhabitants OR surroundings 50,000 to <500,000 inhabitants | 0.44 | 0.316 | -0.18, 1.1 | 0.2 |  |
| Medium \* Core city 50,000 to <500,000 inhabitants OR surroundings 500,000+ inhabitants | 0.48 | 0.248 | -0.01, 0.97 | 0.054 |  |
| High \* Core city 50,000 to <500,000 inhabitants OR surroundings 500,000+ inhabitants | 0.28 | 0.310 | -0.33, 0.89 | 0.4 |  |
| Medium \* Core city 500,000+ inhabitants | 0.53 | 0.255 | 0.02, 1.0 | 0.041 |  |
| High \* Core city 500,000+ inhabitants | 0.27 | 0.321 | -0.37, 0.90 | 0.4 |  |
| Education: CASMIN \* Regions |  |  |  |  | 0.516 |
| Medium \* Northwest | -0.27 | 0.299 | -0.86, 0.32 | 0.4 |  |
| High \* Northwest | -0.41 | 0.342 | -1.1, 0.27 | 0.2 |  |
| Medium \* Central-East | -0.10 | 0.355 | -0.80, 0.60 | 0.8 |  |
| High \* Central-East | 0.03 | 0.411 | -0.78, 0.85 | >0.9 |  |
| Medium \* Central-West | -0.40 | 0.265 | -0.92, 0.13 | 0.14 |  |
| High \* Central-West | -0.50 | 0.307 | -1.1, 0.11 | 0.11 |  |
| Medium \* South | -0.20 | 0.297 | -0.79, 0.38 | 0.5 |  |
| High \* South | -0.03 | 0.343 | -0.71, 0.65 | >0.9 |  |
| Education: CASMIN \* Country of birth |  |  |  |  | 0.568 |
| Medium \* In another country | 0.00 | 0.244 | -0.48, 0.49 | >0.9 |  |
| High \* In another country | -0.21 | 0.281 | -0.77, 0.34 | 0.4 |  |
| Education: CASMIN \* Current living situation |  |  |  |  | 0.178 |
| Medium \* Part-time employed | 0.10 | 0.249 | -0.40, 0.59 | 0.7 |  |
| High \* Part-time employed | 0.02 | 0.262 | -0.49, 0.54 | >0.9 |  |
| Medium \* Unemployed | 0.09 | 0.322 | -0.55, 0.73 | 0.8 |  |
| High \* Unemployed | 0.09 | 0.394 | -0.69, 0.87 | 0.8 |  |
| Medium \* Retired or early retired | -0.47 | 0.212 | -0.89, -0.05 | 0.029 |  |
| High \* Retired or early retired | 0.10 | 0.297 | -0.48, 0.69 | 0.7 |  |
| Medium \* Not employed for other reasons (student, volunteer service, homemaker) | -0.05 | 0.311 | -0.66, 0.56 | 0.9 |  |
| High \* Not employed for other reasons (student, volunteer service, homemaker) | -0.33 | 0.336 | -0.99, 0.33 | 0.3 |  |
| N.Obs | 11,897 |  |  |  |  |
| N.Cluster | 359 |  |  |  |  |
|  |  |  |  |  |  |
| --- | --- | --- | --- | --- | --- |
| Abbreviations: CI = Confidence Interval, OR = Odds Ratio, SE = Standard Error | | | | | |

## Result model estimation: Questionaire C

### Quarter 2

| Characteristic | log(OR) | SE | 95% CI | p-value | p-value (global) |
| --- | --- | --- | --- | --- | --- |
| Sex |  |  |  |  | 0.767 |
| Male | — | — | — |  |  |
| Female | 0.07 | 0.236 | -0.40, 0.54 | 0.8 |  |
| Age |  |  |  |  | 0.542 |
| 16-29 yrs | — | — | — |  |  |
| 30-39 yrs | -0.14 | 0.764 | -1.7, 1.4 | 0.9 |  |
| 40-49 yrs | 0.86 | 0.766 | -0.67, 2.4 | 0.3 |  |
| 50-59 yrs | 0.26 | 0.812 | -1.4, 1.9 | 0.7 |  |
| 60-69 yrs | -0.16 | 0.819 | -1.8, 1.5 | 0.8 |  |
| 70-79 yrs | -1.0 | 1.04 | -3.1, 1.0 | 0.3 |  |
| 80+ | -1.5 | 1.06 | -3.6, 0.66 | 0.2 |  |
| Self-rated health |  |  |  |  | 0.076 |
| Very good/good/fair | — | — | — |  |  |
| Bad/very bad | -0.73 | 0.412 | -1.6, 0.09 | 0.080 |  |
| Chronic diseases |  |  |  |  | <0.001 |
| Yes | — | — | — |  |  |
| No | -0.36 | 0.071 | -0.50, -0.22 | <0.001 |  |
| Satisfaction: Life in general |  |  |  |  | 0.171 |
| Scale value 1 to 3 | — | — | — |  |  |
| Scale value 4 to 7 | -0.25 | 0.210 | -0.67, 0.17 | 0.2 |  |
| Scale value 8 to 10 | -0.04 | 0.234 | -0.51, 0.42 | 0.9 |  |
| Self-rated mental health |  |  |  |  | 0.225 |
| Excellent/very good/good | — | — | — |  |  |
| fair/poor | -0.13 | 0.110 | -0.35, 0.09 | 0.2 |  |
| Smoking |  |  |  |  | <0.001 |
| Daily smoking | — | — | — |  |  |
| Occasional smoking | 0.32 | 0.303 | -0.29, 0.92 | 0.3 |  |
| Non-smoker | 0.92 | 0.174 | 0.57, 1.3 | <0.001 |  |
| Red meat |  |  |  |  | 0.37 |
| Daily or several times a day | — | — | — |  |  |
| 4 to 6 times per week | -0.41 | 0.349 | -1.1, 0.28 | 0.2 |  |
| 1 to 3 times per week | -0.17 | 0.332 | -0.83, 0.49 | 0.6 |  |
| Less than once per week | -0.24 | 0.329 | -0.89, 0.42 | 0.5 |  |
| Never | -0.54 | 0.383 | -1.3, 0.22 | 0.2 |  |
| Regions |  |  |  |  | 0.037 |
| Northeast | — | — | — |  |  |
| Northwest | -0.64 | 0.302 | -1.2, -0.04 | 0.036 |  |
| Central-East | -0.87 | 0.420 | -1.7, -0.03 | 0.042 |  |
| Central-West | -0.73 | 0.312 | -1.4, -0.11 | 0.021 |  |
| South | -0.22 | 0.325 | -0.87, 0.43 | 0.5 |  |
| German nationality |  |  |  |  | 0.006 |
| Yes | — | — | — |  |  |
| No | -0.43 | 0.155 | -0.74, -0.12 | 0.007 |  |
| Education: ISCED (2011) |  |  |  |  | 0.546 |
| Low | — | — | — |  |  |
| Medium | 0.17 | 0.175 | -0.18, 0.52 | 0.3 |  |
| High | 0.22 | 0.223 | -0.22, 0.67 | 0.3 |  |
| Current living situation |  |  |  |  | 0.022 |
| Full-time employed | — | — | — |  |  |
| Part-time employed | -0.49 | 0.228 | -0.95, -0.04 | 0.034 |  |
| Unemployed | -0.25 | 0.261 | -0.77, 0.27 | 0.3 |  |
| Retired or early retired | 0.24 | 0.206 | -0.17, 0.65 | 0.2 |  |
| Not employed for other reasons (student, volunteer service, homemaker) | 0.10 | 0.246 | -0.39, 0.59 | 0.7 |  |
| Normal weight |  |  |  |  | 0.1 |
| Normal weight (18.5 <= BMI < 25) | — | — | — |  |  |
| Not normal weight (BMI < 18.5 or BMI >= 25) | -0.12 | 0.071 | -0.26, 0.02 | 0.10 |  |
| Health risk due to climate change |  |  |  |  | <0.001 |
| Scale value 1 to 3 | — | — | — |  |  |
| Scale value 4 to 7 | 0.83 | 0.208 | 0.42, 1.2 | <0.001 |  |
| Scale value 8 to 10 | 0.96 | 0.271 | 0.42, 1.5 | <0.001 |  |
| Sausage products |  |  |  |  | 0.192 |
| Daily or several times a day | — | — | — |  |  |
| 4 to 6 times per week | 0.29 | 0.283 | -0.27, 0.86 | 0.3 |  |
| 1 to 3 times per week | 0.08 | 0.264 | -0.45, 0.61 | 0.8 |  |
| Less than once per week | -0.05 | 0.269 | -0.59, 0.48 | 0.8 |  |
| Never | 0.45 | 0.326 | -0.19, 1.1 | 0.2 |  |
| Sport |  |  |  |  | 0.335 |
| No sporting activities | — | — | — |  |  |
| Less than 1 hour per week | -0.38 | 0.334 | -1.0, 0.29 | 0.3 |  |
| 1 to less than 2 hours per week | -0.68 | 0.319 | -1.3, -0.04 | 0.037 |  |
| 2 to less than 4 hours per week | -0.40 | 0.362 | -1.1, 0.32 | 0.3 |  |
| 4 hours per week and more | -0.35 | 0.351 | -1.0, 0.35 | 0.3 |  |
| Waited for medical examination date in the last 12 months |  |  |  |  | 0.647 |
| Yes | — | — | — |  |  |
| No | -0.16 | 0.215 | -0.59, 0.27 | 0.5 |  |
| No need for examination or treatment | 0.05 | 0.314 | -0.57, 0.68 | 0.9 |  |
| Interview type |  |  |  |  | 0.809 |
| Computer Assisted Web Interview | — | — | — |  |  |
| Paper and Pencil Interview | -0.07 | 0.297 | -0.66, 0.52 | 0.8 |  |
| BIK community size (categorized) |  |  |  |  | 0.116 |
| BIK region <20,000 inhabitants | — | — | — |  |  |
| BIK region 20,000 to <50,000 inhabitants OR surroundings 50,000 to <500,000 inhabitants | 0.82 | 0.396 | 0.03, 1.6 | 0.042 |  |
| Core city 50,000 to <500,000 inhabitants OR surroundings 500,000+ inhabitants | 0.76 | 0.400 | -0.04, 1.6 | 0.062 |  |
| Core city 500,000+ inhabitants | 0.97 | 0.402 | 0.17, 1.8 | 0.018 |  |
| Country of birth |  |  |  |  | 0.183 |
| In Germany (within current borders) | — | — | — |  |  |
| In another country | -0.31 | 0.231 | -0.77, 0.15 | 0.2 |  |
| Education: CASMIN |  |  |  |  | 0.865 |
| Low | — | — | — |  |  |
| Medium | -0.20 | 0.497 | -1.2, 0.79 | 0.7 |  |
| High | -0.31 | 0.581 | -1.5, 0.85 | 0.6 |  |
| Obesity |  |  |  |  | 0.327 |
| Yes | — | — | — |  |  |
| No | -0.15 | 0.152 | -0.45, 0.15 | 0.3 |  |
| Paying attention to health |  |  |  |  | 0.232 |
| Not at all/less strong/moderate | — | — | — |  |  |
| Strong/very strong | -0.15 | 0.129 | -0.41, 0.10 | 0.2 |  |
| Age \* Self-rated health |  |  |  |  | 0.837 |
| 30-39 yrs \* Bad/very bad | 0.77 | 0.600 | -0.43, 2.0 | 0.2 |  |
| 40-49 yrs \* Bad/very bad | 0.10 | 0.537 | -0.97, 1.2 | 0.9 |  |
| 50-59 yrs \* Bad/very bad | 0.23 | 0.479 | -0.72, 1.2 | 0.6 |  |
| 60-69 yrs \* Bad/very bad | 0.20 | 0.490 | -0.78, 1.2 | 0.7 |  |
| 70-79 yrs \* Bad/very bad | 0.20 | 0.564 | -0.93, 1.3 | 0.7 |  |
| 80+ \* Bad/very bad | -0.10 | 0.535 | -1.2, 0.97 | 0.9 |  |
| Age \* Health risk due to climate change |  |  |  |  | 0.009 |
| 30-39 yrs \* Scale value 4 to 7 | -0.52 | 0.211 | -0.94, -0.10 | 0.017 |  |
| 40-49 yrs \* Scale value 4 to 7 | -0.28 | 0.229 | -0.73, 0.18 | 0.2 |  |
| 50-59 yrs \* Scale value 4 to 7 | -0.65 | 0.207 | -1.1, -0.24 | 0.002 |  |
| 60-69 yrs \* Scale value 4 to 7 | -0.50 | 0.238 | -0.97, -0.02 | 0.040 |  |
| 70-79 yrs \* Scale value 4 to 7 | -0.85 | 0.285 | -1.4, -0.29 | 0.004 |  |
| 80+ \* Scale value 4 to 7 | -0.45 | 0.300 | -1.0, 0.15 | 0.14 |  |
| 30-39 yrs \* Scale value 8 to 10 | -0.66 | 0.278 | -1.2, -0.10 | 0.021 |  |
| 40-49 yrs \* Scale value 8 to 10 | -0.47 | 0.328 | -1.1, 0.18 | 0.2 |  |
| 50-59 yrs \* Scale value 8 to 10 | -0.26 | 0.292 | -0.84, 0.32 | 0.4 |  |
| 60-69 yrs \* Scale value 8 to 10 | -0.44 | 0.348 | -1.1, 0.26 | 0.2 |  |
| 70-79 yrs \* Scale value 8 to 10 | -0.94 | 0.409 | -1.8, -0.12 | 0.025 |  |
| 80+ \* Scale value 8 to 10 | -1.2 | 0.395 | -2.0, -0.44 | 0.003 |  |
| Age \* Sausage products |  |  |  |  | 0.099 |
| 30-39 yrs \* 4 to 6 times per week | -0.38 | 0.382 | -1.1, 0.38 | 0.3 |  |
| 40-49 yrs \* 4 to 6 times per week | -0.47 | 0.441 | -1.3, 0.41 | 0.3 |  |
| 50-59 yrs \* 4 to 6 times per week | -0.48 | 0.394 | -1.3, 0.30 | 0.2 |  |
| 60-69 yrs \* 4 to 6 times per week | -0.47 | 0.412 | -1.3, 0.35 | 0.3 |  |
| 70-79 yrs \* 4 to 6 times per week | -0.37 | 0.498 | -1.4, 0.62 | 0.5 |  |
| 80+ \* 4 to 6 times per week | 0.29 | 0.532 | -0.77, 1.3 | 0.6 |  |
| 30-39 yrs \* 1 to 3 times per week | -0.01 | 0.388 | -0.78, 0.77 | >0.9 |  |
| 40-49 yrs \* 1 to 3 times per week | -0.28 | 0.392 | -1.1, 0.50 | 0.5 |  |
| 50-59 yrs \* 1 to 3 times per week | -0.17 | 0.368 | -0.90, 0.57 | 0.7 |  |
| 60-69 yrs \* 1 to 3 times per week | -0.47 | 0.385 | -1.2, 0.29 | 0.2 |  |
| 70-79 yrs \* 1 to 3 times per week | -0.29 | 0.437 | -1.2, 0.58 | 0.5 |  |
| 80+ \* 1 to 3 times per week | -0.21 | 0.449 | -1.1, 0.68 | 0.6 |  |
| 30-39 yrs \* Less than once per week | 0.16 | 0.380 | -0.59, 0.92 | 0.7 |  |
| 40-49 yrs \* Less than once per week | -0.06 | 0.393 | -0.84, 0.73 | 0.9 |  |
| 50-59 yrs \* Less than once per week | -0.21 | 0.391 | -0.99, 0.57 | 0.6 |  |
| 60-69 yrs \* Less than once per week | -0.58 | 0.398 | -1.4, 0.22 | 0.2 |  |
| 70-79 yrs \* Less than once per week | -0.78 | 0.446 | -1.7, 0.11 | 0.086 |  |
| 80+ \* Less than once per week | 0.43 | 0.483 | -0.53, 1.4 | 0.4 |  |
| 30-39 yrs \* Never | -0.48 | 0.452 | -1.4, 0.42 | 0.3 |  |
| 40-49 yrs \* Never | -0.74 | 0.528 | -1.8, 0.31 | 0.2 |  |
| 50-59 yrs \* Never | -0.93 | 0.539 | -2.0, 0.14 | 0.089 |  |
| 60-69 yrs \* Never | -0.99 | 0.569 | -2.1, 0.15 | 0.087 |  |
| 70-79 yrs \* Never | -1.1 | 0.669 | -2.4, 0.24 | 0.11 |  |
| 80+ \* Never | -1.5 | 0.662 | -2.8, -0.15 | 0.030 |  |
| Age \* Red meat |  |  |  |  | 0.045 |
| 30-39 yrs \* 4 to 6 times per week | 0.32 | 0.633 | -0.94, 1.6 | 0.6 |  |
| 40-49 yrs \* 4 to 6 times per week | 0.70 | 0.583 | -0.46, 1.9 | 0.2 |  |
| 50-59 yrs \* 4 to 6 times per week | 0.54 | 0.733 | -0.92, 2.0 | 0.5 |  |
| 60-69 yrs \* 4 to 6 times per week | 1.7 | 0.744 | 0.23, 3.2 | 0.024 |  |
| 70-79 yrs \* 4 to 6 times per week | 1.5 | 0.893 | -0.24, 3.3 | 0.090 |  |
| 80+ \* 4 to 6 times per week | 0.25 | 0.898 | -1.5, 2.0 | 0.8 |  |
| 30-39 yrs \* 1 to 3 times per week | -0.02 | 0.611 | -1.2, 1.2 | >0.9 |  |
| 40-49 yrs \* 1 to 3 times per week | 0.87 | 0.530 | -0.19, 1.9 | 0.11 |  |
| 50-59 yrs \* 1 to 3 times per week | 0.27 | 0.720 | -1.2, 1.7 | 0.7 |  |
| 60-69 yrs \* 1 to 3 times per week | 1.7 | 0.642 | 0.45, 3.0 | 0.009 |  |
| 70-79 yrs \* 1 to 3 times per week | 1.9 | 0.865 | 0.22, 3.7 | 0.028 |  |
| 80+ \* 1 to 3 times per week | 1.0 | 0.826 | -0.62, 2.7 | 0.2 |  |
| 30-39 yrs \* Less than once per week | -0.27 | 0.626 | -1.5, 0.97 | 0.7 |  |
| 40-49 yrs \* Less than once per week | 0.77 | 0.544 | -0.31, 1.9 | 0.2 |  |
| 50-59 yrs \* Less than once per week | 0.54 | 0.710 | -0.87, 2.0 | 0.4 |  |
| 60-69 yrs \* Less than once per week | 1.9 | 0.666 | 0.57, 3.2 | 0.006 |  |
| 70-79 yrs \* Less than once per week | 1.9 | 0.867 | 0.17, 3.6 | 0.031 |  |
| 80+ \* Less than once per week | 1.1 | 0.827 | -0.57, 2.7 | 0.2 |  |
| 30-39 yrs \* Never | 0.59 | 0.692 | -0.78, 2.0 | 0.4 |  |
| 40-49 yrs \* Never | 0.97 | 0.678 | -0.38, 2.3 | 0.2 |  |
| 50-59 yrs \* Never | 0.82 | 0.824 | -0.82, 2.5 | 0.3 |  |
| 60-69 yrs \* Never | 1.3 | 0.742 | -0.15, 2.8 | 0.079 |  |
| 70-79 yrs \* Never | 2.0 | 0.978 | 0.01, 3.9 | 0.049 |  |
| 80+ \* Never | 1.1 | 0.957 | -0.78, 3.0 | 0.2 |  |
| Age \* Sport |  |  |  |  | 0.496 |
| 30-39 yrs \* Less than 1 hour per week | 0.17 | 0.331 | -0.49, 0.83 | 0.6 |  |
| 40-49 yrs \* Less than 1 hour per week | -0.12 | 0.328 | -0.77, 0.54 | 0.7 |  |
| 50-59 yrs \* Less than 1 hour per week | 0.37 | 0.339 | -0.30, 1.0 | 0.3 |  |
| 60-69 yrs \* Less than 1 hour per week | 0.23 | 0.392 | -0.55, 1.0 | 0.6 |  |
| 70-79 yrs \* Less than 1 hour per week | 0.06 | 0.431 | -0.80, 0.92 | 0.9 |  |
| 80+ \* Less than 1 hour per week | -0.06 | 0.408 | -0.87, 0.75 | 0.9 |  |
| 30-39 yrs \* 1 to less than 2 hours per week | 0.13 | 0.323 | -0.52, 0.77 | 0.7 |  |
| 40-49 yrs \* 1 to less than 2 hours per week | 0.24 | 0.356 | -0.47, 0.95 | 0.5 |  |
| 50-59 yrs \* 1 to less than 2 hours per week | 0.43 | 0.315 | -0.20, 1.1 | 0.2 |  |
| 60-69 yrs \* 1 to less than 2 hours per week | 0.24 | 0.356 | -0.47, 0.95 | 0.5 |  |
| 70-79 yrs \* 1 to less than 2 hours per week | 0.74 | 0.405 | -0.07, 1.5 | 0.074 |  |
| 80+ \* 1 to less than 2 hours per week | 0.98 | 0.413 | 0.16, 1.8 | 0.020 |  |
| 30-39 yrs \* 2 to less than 4 hours per week | 0.31 | 0.331 | -0.35, 0.97 | 0.3 |  |
| 40-49 yrs \* 2 to less than 4 hours per week | 0.05 | 0.376 | -0.70, 0.80 | 0.9 |  |
| 50-59 yrs \* 2 to less than 4 hours per week | 0.83 | 0.346 | 0.14, 1.5 | 0.018 |  |
| 60-69 yrs \* 2 to less than 4 hours per week | 0.49 | 0.420 | -0.35, 1.3 | 0.3 |  |
| 70-79 yrs \* 2 to less than 4 hours per week | 0.49 | 0.445 | -0.40, 1.4 | 0.3 |  |
| 80+ \* 2 to less than 4 hours per week | 0.99 | 0.443 | 0.11, 1.9 | 0.028 |  |
| 30-39 yrs \* 4 hours per week and more | 0.08 | 0.358 | -0.64, 0.79 | 0.8 |  |
| 40-49 yrs \* 4 hours per week and more | -0.10 | 0.377 | -0.86, 0.65 | 0.8 |  |
| 50-59 yrs \* 4 hours per week and more | 0.32 | 0.376 | -0.43, 1.1 | 0.4 |  |
| 60-69 yrs \* 4 hours per week and more | 0.22 | 0.444 | -0.67, 1.1 | 0.6 |  |
| 70-79 yrs \* 4 hours per week and more | 0.21 | 0.528 | -0.85, 1.3 | 0.7 |  |
| 80+ \* 4 hours per week and more | 0.12 | 0.543 | -0.96, 1.2 | 0.8 |  |
| Age \* Waited for medical examination date in the last 12 months |  |  |  |  | 0.006 |
| 30-39 yrs \* No | 0.01 | 0.194 | -0.37, 0.40 | >0.9 |  |
| 40-49 yrs \* No | -0.18 | 0.230 | -0.64, 0.28 | 0.4 |  |
| 50-59 yrs \* No | 0.14 | 0.211 | -0.28, 0.56 | 0.5 |  |
| 60-69 yrs \* No | 0.02 | 0.239 | -0.45, 0.50 | >0.9 |  |
| 70-79 yrs \* No | 0.33 | 0.312 | -0.29, 0.95 | 0.3 |  |
| 80+ \* No | -0.24 | 0.331 | -0.90, 0.42 | 0.5 |  |
| 30-39 yrs \* No need for examination or treatment | 0.14 | 0.251 | -0.36, 0.64 | 0.6 |  |
| 40-49 yrs \* No need for examination or treatment | -0.57 | 0.306 | -1.2, 0.03 | 0.064 |  |
| 50-59 yrs \* No need for examination or treatment | -0.17 | 0.289 | -0.75, 0.40 | 0.6 |  |
| 60-69 yrs \* No need for examination or treatment | 0.79 | 0.451 | -0.11, 1.7 | 0.084 |  |
| 70-79 yrs \* No need for examination or treatment | 0.62 | 0.653 | -0.68, 1.9 | 0.3 |  |
| 80+ \* No need for examination or treatment | -2.1 | 0.569 | -3.2, -0.95 | <0.001 |  |
| Age \* Interview type |  |  |  |  | 0.081 |
| 30-39 yrs \* Paper and Pencil Interview | -0.38 | 0.371 | -1.1, 0.36 | 0.3 |  |
| 40-49 yrs \* Paper and Pencil Interview | -0.43 | 0.385 | -1.2, 0.34 | 0.3 |  |
| 50-59 yrs \* Paper and Pencil Interview | -0.44 | 0.361 | -1.2, 0.27 | 0.2 |  |
| 60-69 yrs \* Paper and Pencil Interview | -0.26 | 0.365 | -0.98, 0.47 | 0.5 |  |
| 70-79 yrs \* Paper and Pencil Interview | 0.31 | 0.385 | -0.45, 1.1 | 0.4 |  |
| 80+ \* Paper and Pencil Interview | 0.29 | 0.437 | -0.58, 1.2 | 0.5 |  |
| Age \* BIK community size (categorized) |  |  |  |  | 0.014 |
| 30-39 yrs \* BIK region 20,000 to <50,000 inhabitants OR surroundings 50,000 to <500,000 inhabitants | -0.46 | 0.311 | -1.1, 0.16 | 0.14 |  |
| 40-49 yrs \* BIK region 20,000 to <50,000 inhabitants OR surroundings 50,000 to <500,000 inhabitants | -0.85 | 0.312 | -1.5, -0.23 | 0.008 |  |
| 50-59 yrs \* BIK region 20,000 to <50,000 inhabitants OR surroundings 50,000 to <500,000 inhabitants | -0.49 | 0.342 | -1.2, 0.19 | 0.2 |  |
| 60-69 yrs \* BIK region 20,000 to <50,000 inhabitants OR surroundings 50,000 to <500,000 inhabitants | -0.66 | 0.330 | -1.3, 0.00 | 0.049 |  |
| 70-79 yrs \* BIK region 20,000 to <50,000 inhabitants OR surroundings 50,000 to <500,000 inhabitants | -0.59 | 0.381 | -1.4, 0.16 | 0.12 |  |
| 80+ \* BIK region 20,000 to <50,000 inhabitants OR surroundings 50,000 to <500,000 inhabitants | -0.19 | 0.533 | -1.2, 0.87 | 0.7 |  |
| 30-39 yrs \* Core city 50,000 to <500,000 inhabitants OR surroundings 500,000+ inhabitants | -0.01 | 0.319 | -0.65, 0.62 | >0.9 |  |
| 40-49 yrs \* Core city 50,000 to <500,000 inhabitants OR surroundings 500,000+ inhabitants | -0.99 | 0.355 | -1.7, -0.28 | 0.007 |  |
| 50-59 yrs \* Core city 50,000 to <500,000 inhabitants OR surroundings 500,000+ inhabitants | -0.46 | 0.350 | -1.2, 0.24 | 0.2 |  |
| 60-69 yrs \* Core city 50,000 to <500,000 inhabitants OR surroundings 500,000+ inhabitants | -0.32 | 0.347 | -1.0, 0.37 | 0.4 |  |
| 70-79 yrs \* Core city 50,000 to <500,000 inhabitants OR surroundings 500,000+ inhabitants | -0.31 | 0.397 | -1.1, 0.48 | 0.4 |  |
| 80+ \* Core city 50,000 to <500,000 inhabitants OR surroundings 500,000+ inhabitants | 0.14 | 0.551 | -0.96, 1.2 | 0.8 |  |
| 30-39 yrs \* Core city 500,000+ inhabitants | -0.53 | 0.317 | -1.2, 0.11 | 0.10 |  |
| 40-49 yrs \* Core city 500,000+ inhabitants | -1.2 | 0.319 | -1.8, -0.54 | <0.001 |  |
| 50-59 yrs \* Core city 500,000+ inhabitants | -0.67 | 0.343 | -1.4, 0.01 | 0.054 |  |
| 60-69 yrs \* Core city 500,000+ inhabitants | -0.81 | 0.351 | -1.5, -0.12 | 0.023 |  |
| 70-79 yrs \* Core city 500,000+ inhabitants | -0.77 | 0.410 | -1.6, 0.04 | 0.063 |  |
| 80+ \* Core city 500,000+ inhabitants | 0.11 | 0.556 | -1.0, 1.2 | 0.8 |  |
| Age \* Regions |  |  |  |  | 0.068 |
| 30-39 yrs \* Northwest | 0.04 | 0.311 | -0.58, 0.66 | 0.9 |  |
| 40-49 yrs \* Northwest | 0.04 | 0.325 | -0.61, 0.69 | >0.9 |  |
| 50-59 yrs \* Northwest | 0.43 | 0.315 | -0.20, 1.1 | 0.2 |  |
| 60-69 yrs \* Northwest | 0.55 | 0.387 | -0.22, 1.3 | 0.2 |  |
| 70-79 yrs \* Northwest | 0.49 | 0.398 | -0.30, 1.3 | 0.2 |  |
| 80+ \* Northwest | 0.07 | 0.414 | -0.76, 0.89 | 0.9 |  |
| 30-39 yrs \* Central-East | 0.01 | 0.360 | -0.71, 0.72 | >0.9 |  |
| 40-49 yrs \* Central-East | -0.33 | 0.439 | -1.2, 0.54 | 0.5 |  |
| 50-59 yrs \* Central-East | 0.26 | 0.379 | -0.49, 1.0 | 0.5 |  |
| 60-69 yrs \* Central-East | -0.01 | 0.440 | -0.88, 0.87 | >0.9 |  |
| 70-79 yrs \* Central-East | 0.81 | 0.503 | -0.20, 1.8 | 0.11 |  |
| 80+ \* Central-East | 1.0 | 0.533 | -0.01, 2.1 | 0.053 |  |
| 30-39 yrs \* Central-West | 0.34 | 0.289 | -0.23, 0.92 | 0.2 |  |
| 40-49 yrs \* Central-West | 0.03 | 0.321 | -0.61, 0.67 | >0.9 |  |
| 50-59 yrs \* Central-West | 0.57 | 0.281 | 0.01, 1.1 | 0.045 |  |
| 60-69 yrs \* Central-West | 0.25 | 0.350 | -0.45, 0.94 | 0.5 |  |
| 70-79 yrs \* Central-West | 0.76 | 0.410 | -0.06, 1.6 | 0.069 |  |
| 80+ \* Central-West | 0.81 | 0.382 | 0.05, 1.6 | 0.037 |  |
| 30-39 yrs \* South | -0.10 | 0.291 | -0.68, 0.48 | 0.7 |  |
| 40-49 yrs \* South | -0.04 | 0.352 | -0.74, 0.66 | >0.9 |  |
| 50-59 yrs \* South | 0.11 | 0.300 | -0.49, 0.71 | 0.7 |  |
| 60-69 yrs \* South | -0.08 | 0.364 | -0.81, 0.64 | 0.8 |  |
| 70-79 yrs \* South | -0.24 | 0.397 | -1.0, 0.56 | 0.6 |  |
| 80+ \* South | 0.68 | 0.417 | -0.16, 1.5 | 0.11 |  |
| Age \* Country of birth |  |  |  |  | 0.208 |
| 30-39 yrs \* In another country | 0.30 | 0.299 | -0.30, 0.89 | 0.3 |  |
| 40-49 yrs \* In another country | -0.41 | 0.292 | -0.99, 0.17 | 0.2 |  |
| 50-59 yrs \* In another country | 0.35 | 0.323 | -0.30, 0.99 | 0.3 |  |
| 60-69 yrs \* In another country | -0.02 | 0.368 | -0.76, 0.71 | >0.9 |  |
| 70-79 yrs \* In another country | 0.13 | 0.421 | -0.71, 0.96 | 0.8 |  |
| 80+ \* In another country | 0.35 | 0.396 | -0.44, 1.1 | 0.4 |  |
| Age \* Education: ISCED (2011) |  |  |  |  | 0.015 |
| 30-39 yrs \* Medium | 0.69 | 0.329 | 0.03, 1.3 | 0.041 |  |
| 40-49 yrs \* Medium | 0.47 | 0.302 | -0.13, 1.1 | 0.12 |  |
| 50-59 yrs \* Medium | 0.59 | 0.324 | -0.05, 1.2 | 0.071 |  |
| 60-69 yrs \* Medium | 0.14 | 0.319 | -0.49, 0.78 | 0.7 |  |
| 70-79 yrs \* Medium | 0.28 | 0.366 | -0.45, 1.0 | 0.5 |  |
| 80+ \* Medium | 0.16 | 0.318 | -0.48, 0.79 | 0.6 |  |
| 30-39 yrs \* High | 0.98 | 0.326 | 0.33, 1.6 | 0.003 |  |
| 40-49 yrs \* High | 0.52 | 0.334 | -0.14, 1.2 | 0.12 |  |
| 50-59 yrs \* High | 0.15 | 0.330 | -0.50, 0.81 | 0.6 |  |
| 60-69 yrs \* High | 0.51 | 0.358 | -0.20, 1.2 | 0.2 |  |
| 70-79 yrs \* High | 0.43 | 0.409 | -0.39, 1.2 | 0.3 |  |
| 80+ \* High | 0.37 | 0.393 | -0.42, 1.1 | 0.4 |  |
| Sex \* Self-rated mental health |  |  |  |  | 0.712 |
| Female \* fair/poor | -0.05 | 0.138 | -0.32, 0.22 | 0.7 |  |
| Sex \* Smoking |  |  |  |  | 0.066 |
| Female \* Occasional smoking | 0.31 | 0.270 | -0.23, 0.84 | 0.3 |  |
| Female \* Non-smoker | -0.19 | 0.160 | -0.51, 0.12 | 0.2 |  |
| Sex \* Sausage products |  |  |  |  | 0.27 |
| Female \* 4 to 6 times per week | 0.15 | 0.245 | -0.33, 0.64 | 0.5 |  |
| Female \* 1 to 3 times per week | 0.06 | 0.227 | -0.39, 0.52 | 0.8 |  |
| Female \* Less than once per week | 0.35 | 0.241 | -0.13, 0.83 | 0.2 |  |
| Female \* Never | 0.39 | 0.272 | -0.15, 0.94 | 0.2 |  |
| Sex \* Sport |  |  |  |  | 0.796 |
| Female \* Less than 1 hour per week | 0.15 | 0.183 | -0.21, 0.52 | 0.4 |  |
| Female \* 1 to less than 2 hours per week | 0.10 | 0.183 | -0.26, 0.47 | 0.6 |  |
| Female \* 2 to less than 4 hours per week | 0.12 | 0.197 | -0.28, 0.51 | 0.6 |  |
| Female \* 4 hours per week and more | 0.25 | 0.212 | -0.17, 0.67 | 0.2 |  |
| Education: CASMIN \* Obesity |  |  |  |  | 0.774 |
| Medium \* No | 0.13 | 0.184 | -0.24, 0.50 | 0.5 |  |
| High \* No | 0.11 | 0.242 | -0.37, 0.59 | 0.7 |  |
| Satisfaction: Life in general \* Education: CASMIN |  |  |  |  | >0.9 |
| Scale value 4 to 7 \* Medium | 0.11 | 0.278 | -0.45, 0.66 | 0.7 |  |
| Scale value 8 to 10 \* Medium | 0.07 | 0.291 | -0.51, 0.65 | 0.8 |  |
| Scale value 4 to 7 \* High | -0.01 | 0.369 | -0.75, 0.73 | >0.9 |  |
| Scale value 8 to 10 \* High | -0.07 | 0.376 | -0.82, 0.68 | 0.9 |  |
| Health risk due to climate change \* Education: CASMIN |  |  |  |  | 0.035 |
| Scale value 4 to 7 \* Medium | -0.28 | 0.185 | -0.65, 0.09 | 0.13 |  |
| Scale value 8 to 10 \* Medium | -0.50 | 0.242 | -0.98, -0.02 | 0.042 |  |
| Scale value 4 to 7 \* High | -0.49 | 0.212 | -0.92, -0.07 | 0.022 |  |
| Scale value 8 to 10 \* High | -0.27 | 0.275 | -0.82, 0.28 | 0.3 |  |
| Education: CASMIN \* Paying attention to health |  |  |  |  | 0.185 |
| Medium \* Strong/very strong | 0.27 | 0.155 | -0.03, 0.58 | 0.081 |  |
| High \* Strong/very strong | 0.25 | 0.177 | -0.10, 0.60 | 0.2 |  |
| Smoking \* Education: CASMIN |  |  |  |  | 0.133 |
| Occasional smoking \* Medium | -0.23 | 0.303 | -0.83, 0.38 | 0.5 |  |
| Non-smoker \* Medium | -0.37 | 0.180 | -0.73, -0.01 | 0.042 |  |
| Occasional smoking \* High | -0.70 | 0.394 | -1.5, 0.09 | 0.081 |  |
| Non-smoker \* High | -0.53 | 0.254 | -1.0, -0.02 | 0.041 |  |
| Sport \* Education: CASMIN |  |  |  |  | 0.52 |
| Less than 1 hour per week \* Medium | -0.03 | 0.246 | -0.52, 0.46 | >0.9 |  |
| 1 to less than 2 hours per week \* Medium | 0.37 | 0.244 | -0.11, 0.86 | 0.13 |  |
| 2 to less than 4 hours per week \* Medium | -0.01 | 0.284 | -0.57, 0.56 | >0.9 |  |
| 4 hours per week and more \* Medium | -0.13 | 0.300 | -0.73, 0.47 | 0.7 |  |
| Less than 1 hour per week \* High | 0.16 | 0.314 | -0.47, 0.79 | 0.6 |  |
| 1 to less than 2 hours per week \* High | 0.29 | 0.302 | -0.31, 0.89 | 0.3 |  |
| 2 to less than 4 hours per week \* High | -0.14 | 0.335 | -0.80, 0.53 | 0.7 |  |
| 4 hours per week and more \* High | 0.10 | 0.356 | -0.61, 0.80 | 0.8 |  |
| Waited for medical examination date in the last 12 months \* Education: CASMIN |  |  |  |  | 0.554 |
| No \* Medium | 0.27 | 0.193 | -0.11, 0.65 | 0.2 |  |
| No need for examination or treatment \* Medium | 0.13 | 0.311 | -0.49, 0.75 | 0.7 |  |
| No \* High | 0.36 | 0.216 | -0.07, 0.79 | 0.10 |  |
| No need for examination or treatment \* High | 0.24 | 0.326 | -0.41, 0.89 | 0.5 |  |
| BIK community size (categorized) \* Education: CASMIN |  |  |  |  | 0.668 |
| BIK region 20,000 to <50,000 inhabitants OR surroundings 50,000 to <500,000 inhabitants \* Medium | -0.27 | 0.304 | -0.88, 0.34 | 0.4 |  |
| Core city 50,000 to <500,000 inhabitants OR surroundings 500,000+ inhabitants \* Medium | -0.40 | 0.327 | -1.1, 0.25 | 0.2 |  |
| Core city 500,000+ inhabitants \* Medium | -0.40 | 0.316 | -1.0, 0.23 | 0.2 |  |
| BIK region 20,000 to <50,000 inhabitants OR surroundings 50,000 to <500,000 inhabitants \* High | 0.20 | 0.302 | -0.41, 0.80 | 0.5 |  |
| Core city 50,000 to <500,000 inhabitants OR surroundings 500,000+ inhabitants \* High | 0.05 | 0.332 | -0.61, 0.71 | 0.9 |  |
| Core city 500,000+ inhabitants \* High | 0.06 | 0.327 | -0.60, 0.71 | 0.9 |  |
| Regions \* Education: CASMIN |  |  |  |  | 0.309 |
| Northwest \* Medium | 0.53 | 0.296 | -0.06, 1.1 | 0.078 |  |
| Central-East \* Medium | 0.83 | 0.413 | 0.01, 1.7 | 0.048 |  |
| Central-West \* Medium | 0.47 | 0.266 | -0.06, 1.0 | 0.082 |  |
| South \* Medium | 0.35 | 0.283 | -0.21, 0.92 | 0.2 |  |
| Northwest \* High | 0.60 | 0.332 | -0.06, 1.3 | 0.073 |  |
| Central-East \* High | 1.2 | 0.493 | 0.19, 2.2 | 0.020 |  |
| Central-West \* High | 0.39 | 0.337 | -0.28, 1.1 | 0.2 |  |
| South \* High | 0.56 | 0.330 | -0.10, 1.2 | 0.093 |  |
| Current living situation \* Education: CASMIN |  |  |  |  | 0.06 |
| Part-time employed \* Medium | 0.66 | 0.268 | 0.13, 1.2 | 0.015 |  |
| Unemployed \* Medium | -0.15 | 0.323 | -0.79, 0.50 | 0.7 |  |
| Retired or early retired \* Medium | 0.19 | 0.236 | -0.27, 0.66 | 0.4 |  |
| Not employed for other reasons (student, volunteer service, homemaker) \* Medium | 0.11 | 0.257 | -0.40, 0.62 | 0.7 |  |
| Part-time employed \* High | 0.80 | 0.274 | 0.25, 1.3 | 0.005 |  |
| Unemployed \* High | -0.10 | 0.394 | -0.89, 0.68 | 0.8 |  |
| Retired or early retired \* High | 0.22 | 0.279 | -0.34, 0.77 | 0.4 |  |
| Not employed for other reasons (student, volunteer service, homemaker) \* High | -0.28 | 0.325 | -0.93, 0.37 | 0.4 |  |
| N.Obs | 11,806 |  |  |  |  |
| N.Cluster | 359 |  |  |  |  |
|  |  |  |  |  |  |
| --- | --- | --- | --- | --- | --- |
| Abbreviations: CI = Confidence Interval, OR = Odds Ratio, SE = Standard Error | | | | | |

### Quarter 3

| Characteristic | log(OR) | SE | 95% CI | p-value | p-value (global) |
| --- | --- | --- | --- | --- | --- |
| Sex |  |  |  |  | 0.14 |
| Male | — | — | — |  |  |
| Female | 0.24 | 0.161 | -0.08, 0.56 | 0.14 |  |
| Age |  |  |  |  | 0.153 |
| 16-29 yrs | — | — | — |  |  |
| 30-39 yrs | 0.26 | 0.528 | -0.78, 1.3 | 0.6 |  |
| 40-49 yrs | 0.50 | 0.564 | -0.61, 1.6 | 0.4 |  |
| 50-59 yrs | 0.92 | 0.588 | -0.24, 2.1 | 0.12 |  |
| 60-69 yrs | 1.4 | 0.640 | 0.14, 2.7 | 0.029 |  |
| 70-79 yrs | 1.2 | 0.679 | -0.12, 2.6 | 0.075 |  |
| 80+ | -0.89 | 1.14 | -3.1, 1.4 | 0.4 |  |
| Self-rated health |  |  |  |  | 0.258 |
| Very good/good/fair | — | — | — |  |  |
| Bad/very bad | -0.56 | 0.495 | -1.5, 0.42 | 0.3 |  |
| Satisfaction: Life in general |  |  |  |  | 0.549 |
| Scale value 1 to 3 | — | — | — |  |  |
| Scale value 4 to 7 | -0.20 | 0.248 | -0.68, 0.29 | 0.4 |  |
| Scale value 8 to 10 | -0.08 | 0.260 | -0.60, 0.43 | 0.8 |  |
| Smoking |  |  |  |  | 0.088 |
| Daily smoking | — | — | — |  |  |
| Occasional smoking | 0.54 | 0.384 | -0.22, 1.3 | 0.2 |  |
| Non-smoker | 0.53 | 0.243 | 0.05, 1.0 | 0.031 |  |
| Sausage products |  |  |  |  | 0.764 |
| Daily or several times a day | — | — | — |  |  |
| 4 to 6 times per week | 0.10 | 0.262 | -0.42, 0.62 | 0.7 |  |
| 1 to 3 times per week | 0.01 | 0.250 | -0.49, 0.50 | >0.9 |  |
| Less than once per week | 0.18 | 0.253 | -0.32, 0.68 | 0.5 |  |
| Never | 0.20 | 0.265 | -0.32, 0.72 | 0.5 |  |
| BIK community size (categorized) |  |  |  |  | 0.502 |
| BIK region <20,000 inhabitants | — | — | — |  |  |
| BIK region 20,000 to <50,000 inhabitants OR surroundings 50,000 to <500,000 inhabitants | 0.08 | 0.145 | -0.21, 0.37 | 0.6 |  |
| Core city 50,000 to <500,000 inhabitants OR surroundings 500,000+ inhabitants | -0.05 | 0.156 | -0.35, 0.26 | 0.8 |  |
| Core city 500,000+ inhabitants | 0.11 | 0.153 | -0.19, 0.41 | 0.5 |  |
| German nationality |  |  |  |  | 0.155 |
| Yes | — | — | — |  |  |
| No | -0.46 | 0.322 | -1.1, 0.18 | 0.2 |  |
| Education: ISCED (2011) |  |  |  |  | 0.412 |
| Low | — | — | — |  |  |
| Medium | 0.23 | 0.178 | -0.12, 0.58 | 0.2 |  |
| High | 0.13 | 0.198 | -0.27, 0.52 | 0.5 |  |
| Obesity |  |  |  |  | 0.306 |
| Yes | — | — | — |  |  |
| No | 0.29 | 0.286 | -0.27, 0.86 | 0.3 |  |
| Overweight |  |  |  |  | 0.285 |
| Yes | — | — | — |  |  |
| No | -0.23 | 0.213 | -0.65, 0.19 | 0.3 |  |
| Chronic diseases |  |  |  |  | 0.678 |
| Yes | — | — | — |  |  |
| No | -0.06 | 0.133 | -0.32, 0.21 | 0.7 |  |
| Sport |  |  |  |  | 0.769 |
| No sporting activities | — | — | — |  |  |
| Less than 1 hour per week | -0.32 | 0.288 | -0.89, 0.25 | 0.3 |  |
| 1 to less than 2 hours per week | -0.05 | 0.305 | -0.65, 0.55 | 0.9 |  |
| 2 to less than 4 hours per week | -0.03 | 0.291 | -0.60, 0.55 | >0.9 |  |
| 4 hours per week and more | -0.21 | 0.343 | -0.89, 0.47 | 0.5 |  |
| Interview type |  |  |  |  | 0.371 |
| Computer Assisted Web Interview | — | — | — |  |  |
| Paper and Pencil Interview | -0.24 | 0.272 | -0.78, 0.29 | 0.4 |  |
| Country of birth |  |  |  |  | 0.888 |
| In Germany (within current borders) | — | — | — |  |  |
| In another country | -0.04 | 0.307 | -0.65, 0.56 | 0.9 |  |
| Regions |  |  |  |  | >0.9 |
| Northeast | — | — | — |  |  |
| Northwest | 0.03 | 0.160 | -0.28, 0.35 | 0.8 |  |
| Central-East | -0.07 | 0.168 | -0.40, 0.26 | 0.7 |  |
| Central-West | 0.04 | 0.157 | -0.27, 0.35 | 0.8 |  |
| South | 0.00 | 0.147 | -0.29, 0.29 | >0.9 |  |
| Education: CASMIN |  |  |  |  | 0.55 |
| Low | — | — | — |  |  |
| Medium | -0.15 | 0.232 | -0.61, 0.31 | 0.5 |  |
| High | 0.10 | 0.332 | -0.55, 0.76 | 0.8 |  |
| Age \* Obesity |  |  |  |  | 0.704 |
| 30-39 yrs \* No | 0.15 | 0.301 | -0.44, 0.75 | 0.6 |  |
| 40-49 yrs \* No | -0.10 | 0.315 | -0.72, 0.52 | 0.7 |  |
| 50-59 yrs \* No | -0.16 | 0.297 | -0.75, 0.42 | 0.6 |  |
| 60-69 yrs \* No | -0.23 | 0.312 | -0.85, 0.39 | 0.5 |  |
| 70-79 yrs \* No | -0.45 | 0.355 | -1.1, 0.25 | 0.2 |  |
| 80+ \* No | -0.07 | 0.452 | -0.97, 0.82 | 0.9 |  |
| Age \* Overweight |  |  |  |  | 0.364 |
| 30-39 yrs \* No | -0.21 | 0.214 | -0.63, 0.21 | 0.3 |  |
| 40-49 yrs \* No | -0.17 | 0.222 | -0.61, 0.27 | 0.5 |  |
| 50-59 yrs \* No | -0.15 | 0.217 | -0.58, 0.28 | 0.5 |  |
| 60-69 yrs \* No | 0.08 | 0.230 | -0.37, 0.54 | 0.7 |  |
| 70-79 yrs \* No | -0.23 | 0.275 | -0.78, 0.31 | 0.4 |  |
| 80+ \* No | 0.54 | 0.337 | -0.13, 1.2 | 0.11 |  |
| Age \* Self-rated health |  |  |  |  | 0.281 |
| 30-39 yrs \* Bad/very bad | 0.45 | 0.608 | -0.75, 1.7 | 0.5 |  |
| 40-49 yrs \* Bad/very bad | 0.72 | 0.622 | -0.51, 1.9 | 0.2 |  |
| 50-59 yrs \* Bad/very bad | 0.57 | 0.589 | -0.59, 1.7 | 0.3 |  |
| 60-69 yrs \* Bad/very bad | -0.23 | 0.564 | -1.3, 0.89 | 0.7 |  |
| 70-79 yrs \* Bad/very bad | 0.34 | 0.658 | -0.96, 1.6 | 0.6 |  |
| 80+ \* Bad/very bad | 0.08 | 0.625 | -1.2, 1.3 | >0.9 |  |
| Age \* Chronic diseases |  |  |  |  | 0.312 |
| 30-39 yrs \* No | -0.02 | 0.195 | -0.41, 0.36 | >0.9 |  |
| 40-49 yrs \* No | 0.48 | 0.223 | 0.04, 0.92 | 0.034 |  |
| 50-59 yrs \* No | -0.05 | 0.201 | -0.45, 0.34 | 0.8 |  |
| 60-69 yrs \* No | 0.09 | 0.224 | -0.36, 0.53 | 0.7 |  |
| 70-79 yrs \* No | 0.15 | 0.279 | -0.40, 0.70 | 0.6 |  |
| 80+ \* No | 0.04 | 0.338 | -0.63, 0.71 | >0.9 |  |
| Age \* Satisfaction: Life in general |  |  |  |  | 0.043 |
| 30-39 yrs \* Scale value 4 to 7 | 0.74 | 0.341 | 0.07, 1.4 | 0.031 |  |
| 40-49 yrs \* Scale value 4 to 7 | -0.49 | 0.402 | -1.3, 0.31 | 0.2 |  |
| 50-59 yrs \* Scale value 4 to 7 | -0.02 | 0.361 | -0.73, 0.69 | >0.9 |  |
| 60-69 yrs \* Scale value 4 to 7 | 0.09 | 0.366 | -0.63, 0.81 | 0.8 |  |
| 70-79 yrs \* Scale value 4 to 7 | 0.17 | 0.445 | -0.71, 1.0 | 0.7 |  |
| 80+ \* Scale value 4 to 7 | 0.77 | 0.548 | -0.31, 1.8 | 0.2 |  |
| 30-39 yrs \* Scale value 8 to 10 | 0.78 | 0.354 | 0.08, 1.5 | 0.030 |  |
| 40-49 yrs \* Scale value 8 to 10 | -0.55 | 0.416 | -1.4, 0.27 | 0.2 |  |
| 50-59 yrs \* Scale value 8 to 10 | 0.10 | 0.359 | -0.61, 0.81 | 0.8 |  |
| 60-69 yrs \* Scale value 8 to 10 | 0.57 | 0.392 | -0.20, 1.3 | 0.15 |  |
| 70-79 yrs \* Scale value 8 to 10 | 0.31 | 0.447 | -0.57, 1.2 | 0.5 |  |
| 80+ \* Scale value 8 to 10 | 0.56 | 0.559 | -0.55, 1.7 | 0.3 |  |
| Age \* Smoking |  |  |  |  | 0.336 |
| 30-39 yrs \* Occasional smoking | -0.01 | 0.382 | -0.76, 0.74 | >0.9 |  |
| 40-49 yrs \* Occasional smoking | 0.14 | 0.431 | -0.72, 0.99 | 0.8 |  |
| 50-59 yrs \* Occasional smoking | 0.14 | 0.400 | -0.65, 0.93 | 0.7 |  |
| 60-69 yrs \* Occasional smoking | -0.16 | 0.490 | -1.1, 0.81 | 0.7 |  |
| 70-79 yrs \* Occasional smoking | 2.1 | 1.26 | -0.35, 4.6 | 0.091 |  |
| 80+ \* Occasional smoking | 0.32 | 0.877 | -1.4, 2.1 | 0.7 |  |
| 30-39 yrs \* Non-smoker | -0.10 | 0.250 | -0.59, 0.40 | 0.7 |  |
| 40-49 yrs \* Non-smoker | 0.07 | 0.271 | -0.46, 0.60 | 0.8 |  |
| 50-59 yrs \* Non-smoker | -0.22 | 0.249 | -0.71, 0.27 | 0.4 |  |
| 60-69 yrs \* Non-smoker | -0.28 | 0.291 | -0.85, 0.29 | 0.3 |  |
| 70-79 yrs \* Non-smoker | -0.49 | 0.362 | -1.2, 0.23 | 0.2 |  |
| 80+ \* Non-smoker | 0.90 | 0.709 | -0.50, 2.3 | 0.2 |  |
| Age \* Sausage products |  |  |  |  | 0.12 |
| 30-39 yrs \* 4 to 6 times per week | -0.49 | 0.390 | -1.3, 0.28 | 0.2 |  |
| 40-49 yrs \* 4 to 6 times per week | 0.33 | 0.378 | -0.41, 1.1 | 0.4 |  |
| 50-59 yrs \* 4 to 6 times per week | 0.54 | 0.347 | -0.15, 1.2 | 0.12 |  |
| 60-69 yrs \* 4 to 6 times per week | -0.29 | 0.395 | -1.1, 0.49 | 0.5 |  |
| 70-79 yrs \* 4 to 6 times per week | 0.01 | 0.433 | -0.85, 0.86 | >0.9 |  |
| 80+ \* 4 to 6 times per week | 0.39 | 0.530 | -0.65, 1.4 | 0.5 |  |
| 30-39 yrs \* 1 to 3 times per week | -0.60 | 0.371 | -1.3, 0.14 | 0.11 |  |
| 40-49 yrs \* 1 to 3 times per week | 0.33 | 0.372 | -0.41, 1.1 | 0.4 |  |
| 50-59 yrs \* 1 to 3 times per week | 0.29 | 0.333 | -0.37, 0.95 | 0.4 |  |
| 60-69 yrs \* 1 to 3 times per week | -0.29 | 0.365 | -1.0, 0.43 | 0.4 |  |
| 70-79 yrs \* 1 to 3 times per week | -0.01 | 0.419 | -0.84, 0.81 | >0.9 |  |
| 80+ \* 1 to 3 times per week | -0.22 | 0.481 | -1.2, 0.73 | 0.6 |  |
| 30-39 yrs \* Less than once per week | -0.52 | 0.380 | -1.3, 0.23 | 0.2 |  |
| 40-49 yrs \* Less than once per week | -0.13 | 0.379 | -0.88, 0.61 | 0.7 |  |
| 50-59 yrs \* Less than once per week | 0.40 | 0.337 | -0.26, 1.1 | 0.2 |  |
| 60-69 yrs \* Less than once per week | -0.28 | 0.395 | -1.1, 0.50 | 0.5 |  |
| 70-79 yrs \* Less than once per week | 0.26 | 0.448 | -0.62, 1.1 | 0.6 |  |
| 80+ \* Less than once per week | -0.12 | 0.541 | -1.2, 0.95 | 0.8 |  |
| 30-39 yrs \* Never | -0.75 | 0.404 | -1.5, 0.05 | 0.065 |  |
| 40-49 yrs \* Never | -0.54 | 0.466 | -1.5, 0.38 | 0.2 |  |
| 50-59 yrs \* Never | 0.19 | 0.414 | -0.63, 1.0 | 0.7 |  |
| 60-69 yrs \* Never | -0.50 | 0.499 | -1.5, 0.49 | 0.3 |  |
| 70-79 yrs \* Never | -0.82 | 0.572 | -1.9, 0.31 | 0.2 |  |
| 80+ \* Never | 0.35 | 0.661 | -0.95, 1.7 | 0.6 |  |
| Age \* Sport |  |  |  |  | 0.008 |
| 30-39 yrs \* Less than 1 hour per week | 0.40 | 0.294 | -0.18, 0.98 | 0.2 |  |
| 40-49 yrs \* Less than 1 hour per week | 0.05 | 0.282 | -0.51, 0.60 | 0.9 |  |
| 50-59 yrs \* Less than 1 hour per week | 0.35 | 0.311 | -0.26, 0.96 | 0.3 |  |
| 60-69 yrs \* Less than 1 hour per week | 0.41 | 0.366 | -0.31, 1.1 | 0.3 |  |
| 70-79 yrs \* Less than 1 hour per week | 0.37 | 0.374 | -0.37, 1.1 | 0.3 |  |
| 80+ \* Less than 1 hour per week | 0.19 | 0.407 | -0.61, 0.99 | 0.6 |  |
| 30-39 yrs \* 1 to less than 2 hours per week | 0.19 | 0.296 | -0.39, 0.78 | 0.5 |  |
| 40-49 yrs \* 1 to less than 2 hours per week | 0.14 | 0.300 | -0.45, 0.74 | 0.6 |  |
| 50-59 yrs \* 1 to less than 2 hours per week | 0.54 | 0.299 | -0.05, 1.1 | 0.072 |  |
| 60-69 yrs \* 1 to less than 2 hours per week | 0.20 | 0.336 | -0.46, 0.86 | 0.6 |  |
| 70-79 yrs \* 1 to less than 2 hours per week | -0.30 | 0.349 | -0.98, 0.39 | 0.4 |  |
| 80+ \* 1 to less than 2 hours per week | -0.04 | 0.440 | -0.91, 0.83 | >0.9 |  |
| 30-39 yrs \* 2 to less than 4 hours per week | 0.20 | 0.321 | -0.44, 0.83 | 0.5 |  |
| 40-49 yrs \* 2 to less than 4 hours per week | 0.13 | 0.330 | -0.52, 0.79 | 0.7 |  |
| 50-59 yrs \* 2 to less than 4 hours per week | 0.22 | 0.312 | -0.39, 0.84 | 0.5 |  |
| 60-69 yrs \* 2 to less than 4 hours per week | 0.27 | 0.339 | -0.40, 0.94 | 0.4 |  |
| 70-79 yrs \* 2 to less than 4 hours per week | 0.32 | 0.399 | -0.47, 1.1 | 0.4 |  |
| 80+ \* 2 to less than 4 hours per week | 0.04 | 0.492 | -0.93, 1.0 | >0.9 |  |
| 30-39 yrs \* 4 hours per week and more | -0.18 | 0.335 | -0.84, 0.48 | 0.6 |  |
| 40-49 yrs \* 4 hours per week and more | 0.63 | 0.391 | -0.14, 1.4 | 0.11 |  |
| 50-59 yrs \* 4 hours per week and more | -0.27 | 0.338 | -0.93, 0.40 | 0.4 |  |
| 60-69 yrs \* 4 hours per week and more | 0.13 | 0.393 | -0.64, 0.91 | 0.7 |  |
| 70-79 yrs \* 4 hours per week and more | 1.2 | 0.476 | 0.26, 2.1 | 0.013 |  |
| 80+ \* 4 hours per week and more | 1.3 | 0.670 | 0.03, 2.7 | 0.046 |  |
| Age \* Interview type |  |  |  |  | 0.311 |
| 30-39 yrs \* Paper and Pencil Interview | 0.28 | 0.379 | -0.47, 1.0 | 0.5 |  |
| 40-49 yrs \* Paper and Pencil Interview | 0.46 | 0.374 | -0.28, 1.2 | 0.2 |  |
| 50-59 yrs \* Paper and Pencil Interview | 0.17 | 0.318 | -0.46, 0.79 | 0.6 |  |
| 60-69 yrs \* Paper and Pencil Interview | 0.30 | 0.341 | -0.37, 0.97 | 0.4 |  |
| 70-79 yrs \* Paper and Pencil Interview | 0.74 | 0.337 | 0.07, 1.4 | 0.030 |  |
| 80+ \* Paper and Pencil Interview | 0.59 | 0.419 | -0.24, 1.4 | 0.2 |  |
| Age \* Country of birth |  |  |  |  | 0.259 |
| 30-39 yrs \* In another country | -0.24 | 0.402 | -1.0, 0.55 | 0.6 |  |
| 40-49 yrs \* In another country | -0.23 | 0.422 | -1.1, 0.60 | 0.6 |  |
| 50-59 yrs \* In another country | -0.23 | 0.422 | -1.1, 0.60 | 0.6 |  |
| 60-69 yrs \* In another country | -0.37 | 0.502 | -1.4, 0.62 | 0.5 |  |
| 70-79 yrs \* In another country | -0.61 | 0.408 | -1.4, 0.20 | 0.14 |  |
| 80+ \* In another country | 0.64 | 0.488 | -0.32, 1.6 | 0.2 |  |
| Age \* German nationality |  |  |  |  | 0.803 |
| 30-39 yrs \* No | -0.06 | 0.472 | -0.99, 0.87 | >0.9 |  |
| 40-49 yrs \* No | -0.55 | 0.460 | -1.5, 0.36 | 0.2 |  |
| 50-59 yrs \* No | -0.60 | 0.501 | -1.6, 0.39 | 0.2 |  |
| 60-69 yrs \* No | 0.10 | 0.608 | -1.1, 1.3 | 0.9 |  |
| 70-79 yrs \* No | -0.15 | 0.568 | -1.3, 0.98 | 0.8 |  |
| 80+ \* No | -0.43 | 0.867 | -2.1, 1.3 | 0.6 |  |
| Age \* Education: ISCED (2011) |  |  |  |  | 0.655 |
| 30-39 yrs \* Medium | -0.39 | 0.307 | -0.99, 0.22 | 0.2 |  |
| 40-49 yrs \* Medium | 0.05 | 0.327 | -0.60, 0.69 | 0.9 |  |
| 50-59 yrs \* Medium | -0.32 | 0.365 | -1.0, 0.40 | 0.4 |  |
| 60-69 yrs \* Medium | 0.00 | 0.338 | -0.67, 0.66 | >0.9 |  |
| 70-79 yrs \* Medium | 0.34 | 0.350 | -0.35, 1.0 | 0.3 |  |
| 80+ \* Medium | 0.08 | 0.403 | -0.72, 0.87 | 0.9 |  |
| 30-39 yrs \* High | -0.06 | 0.337 | -0.73, 0.61 | 0.9 |  |
| 40-49 yrs \* High | 0.30 | 0.334 | -0.36, 0.96 | 0.4 |  |
| 50-59 yrs \* High | 0.07 | 0.373 | -0.66, 0.81 | 0.9 |  |
| 60-69 yrs \* High | 0.10 | 0.358 | -0.61, 0.81 | 0.8 |  |
| 70-79 yrs \* High | 0.65 | 0.378 | -0.10, 1.4 | 0.087 |  |
| 80+ \* High | 0.35 | 0.399 | -0.43, 1.1 | 0.4 |  |
| Sex \* Regions |  |  |  |  | 0.299 |
| Female \* Northwest | -0.07 | 0.209 | -0.48, 0.34 | 0.7 |  |
| Female \* Central-East | 0.31 | 0.247 | -0.17, 0.80 | 0.2 |  |
| Female \* Central-West | -0.15 | 0.191 | -0.53, 0.23 | 0.4 |  |
| Female \* South | -0.04 | 0.195 | -0.43, 0.34 | 0.8 |  |
| Sex \* Country of birth |  |  |  |  | >0.9 |
| Female \* In another country | 0.01 | 0.186 | -0.36, 0.38 | >0.9 |  |
| Obesity \* Education: CASMIN |  |  |  |  | 0.629 |
| No \* Medium | -0.13 | 0.189 | -0.50, 0.24 | 0.5 |  |
| No \* High | 0.02 | 0.240 | -0.45, 0.50 | >0.9 |  |
| Overweight \* Education: CASMIN |  |  |  |  | 0.053 |
| No \* Medium | 0.39 | 0.165 | 0.07, 0.72 | 0.018 |  |
| No \* High | 0.39 | 0.196 | 0.00, 0.77 | 0.051 |  |
| Smoking \* Education: CASMIN |  |  |  |  | 0.22 |
| Occasional smoking \* Medium | -0.44 | 0.366 | -1.2, 0.28 | 0.2 |  |
| Non-smoker \* Medium | 0.16 | 0.180 | -0.19, 0.52 | 0.4 |  |
| Occasional smoking \* High | -0.57 | 0.444 | -1.4, 0.31 | 0.2 |  |
| Non-smoker \* High | -0.17 | 0.245 | -0.65, 0.31 | 0.5 |  |
| Sport \* Education: CASMIN |  |  |  |  | 0.143 |
| Less than 1 hour per week \* Medium | 0.44 | 0.223 | 0.00, 0.88 | 0.052 |  |
| 1 to less than 2 hours per week \* Medium | 0.32 | 0.243 | -0.16, 0.80 | 0.2 |  |
| 2 to less than 4 hours per week \* Medium | 0.06 | 0.245 | -0.42, 0.55 | 0.8 |  |
| 4 hours per week and more \* Medium | 0.33 | 0.302 | -0.27, 0.92 | 0.3 |  |
| Less than 1 hour per week \* High | 0.14 | 0.250 | -0.35, 0.63 | 0.6 |  |
| 1 to less than 2 hours per week \* High | 0.09 | 0.259 | -0.42, 0.60 | 0.7 |  |
| 2 to less than 4 hours per week \* High | 0.18 | 0.279 | -0.37, 0.73 | 0.5 |  |
| 4 hours per week and more \* High | 0.53 | 0.320 | -0.11, 1.2 | 0.10 |  |
| N.Obs | 11,800 |  |  |  |  |
| N.Cluster | 359 |  |  |  |  |
|  |  |  |  |  |  |
| --- | --- | --- | --- | --- | --- |
| Abbreviations: CI = Confidence Interval, OR = Odds Ratio, SE = Standard Error | | | | | |

### Quarter 4

| Characteristic | log(OR) | SE | 95% CI | p-value | p-value (global) |
| --- | --- | --- | --- | --- | --- |
| Sex |  |  |  |  | 0.506 |
| Male | — | — | — |  |  |
| Female | 0.22 | 0.323 | -0.44, 0.87 | 0.5 |  |
| Age |  |  |  |  | 0.56 |
| 16-29 yrs | — | — | — |  |  |
| 30-39 yrs | -0.05 | 0.795 | -1.7, 1.6 | >0.9 |  |
| 40-49 yrs | 0.38 | 0.892 | -1.4, 2.2 | 0.7 |  |
| 50-59 yrs | 0.26 | 0.938 | -1.6, 2.1 | 0.8 |  |
| 60-69 yrs | 1.4 | 0.930 | -0.49, 3.3 | 0.14 |  |
| 70-79 yrs | 2.2 | 1.40 | -0.67, 5.0 | 0.13 |  |
| 80+ | 0.20 | 1.49 | -2.8, 3.2 | 0.9 |  |
| Education: CASMIN |  |  |  |  | 0.331 |
| Low | — | — | — |  |  |
| Medium | 0.51 | 0.678 | -0.85, 1.9 | 0.5 |  |
| High | -0.41 | 0.753 | -1.9, 1.1 | 0.6 |  |
| Overweight |  |  |  |  | 0.227 |
| Yes | — | — | — |  |  |
| No | 0.26 | 0.211 | -0.17, 0.68 | 0.2 |  |
| Health risk due to climate change |  |  |  |  | 0.701 |
| Scale value 1 to 3 | — | — | — |  |  |
| Scale value 4 to 7 | -0.11 | 0.137 | -0.38, 0.17 | 0.4 |  |
| Scale value 8 to 10 | -0.01 | 0.218 | -0.45, 0.43 | >0.9 |  |
| Smoking |  |  |  |  | 0.14 |
| Daily smoking | — | — | — |  |  |
| Occasional smoking | 0.47 | 0.382 | -0.30, 1.2 | 0.2 |  |
| Non-smoker | 0.50 | 0.257 | -0.02, 1.0 | 0.059 |  |
| Red meat |  |  |  |  | 0.286 |
| Daily or several times a day | — | — | — |  |  |
| 4 to 6 times per week | 0.69 | 0.497 | -0.31, 1.7 | 0.2 |  |
| 1 to 3 times per week | 0.74 | 0.428 | -0.12, 1.6 | 0.090 |  |
| Less than once per week | 0.95 | 0.441 | 0.06, 1.8 | 0.037 |  |
| Never | 1.0 | 0.534 | -0.07, 2.1 | 0.067 |  |
| Sport |  |  |  |  | 0.895 |
| No sporting activities | — | — | — |  |  |
| Less than 1 hour per week | -0.24 | 0.341 | -0.92, 0.45 | 0.5 |  |
| 1 to less than 2 hours per week | -0.20 | 0.315 | -0.83, 0.43 | 0.5 |  |
| 2 to less than 4 hours per week | -0.06 | 0.323 | -0.71, 0.59 | 0.8 |  |
| 4 hours per week and more | -0.28 | 0.345 | -0.98, 0.41 | 0.4 |  |
| Waited for medical examination date in the last 12 months |  |  |  |  | 0.082 |
| Yes | — | — | — |  |  |
| No | 0.10 | 0.134 | -0.17, 0.37 | 0.5 |  |
| No need for examination or treatment | 0.56 | 0.253 | 0.05, 1.1 | 0.032 |  |
| Country of birth |  |  |  |  | <0.001 |
| In Germany (within current borders) | — | — | — |  |  |
| In another country | -1.1 | 0.330 | -1.8, -0.46 | 0.001 |  |
| German nationality |  |  |  |  | 0.821 |
| Yes | — | — | — |  |  |
| No | -0.07 | 0.316 | -0.71, 0.57 | 0.8 |  |
| Household size |  |  |  |  | 0.267 |
| Single-person household | — | — | — |  |  |
| Multi-person household | 0.09 | 0.078 | -0.07, 0.24 | 0.3 |  |
| Education: ISCED (2011) |  |  |  |  | <0.001 |
| Low | — | — | — |  |  |
| Medium | 0.53 | 0.198 | 0.13, 0.93 | 0.011 |  |
| High | 1.0 | 0.230 | 0.54, 1.5 | <0.001 |  |
| Current living situation |  |  |  |  | >0.9 |
| Full-time employed | — | — | — |  |  |
| Part-time employed | -0.14 | 0.238 | -0.62, 0.34 | 0.6 |  |
| Unemployed | -0.15 | 0.278 | -0.71, 0.41 | 0.6 |  |
| Retired or early retired | 0.05 | 0.200 | -0.35, 0.45 | 0.8 |  |
| Not employed for other reasons (student, volunteer service, homemaker) | 0.01 | 0.248 | -0.49, 0.51 | >0.9 |  |
| Self-rated health |  |  |  |  | 0.541 |
| Very good/good/fair | — | — | — |  |  |
| Bad/very bad | 0.28 | 0.456 | -0.64, 1.2 | 0.5 |  |
| Chronic diseases |  |  |  |  | 0.054 |
| Yes | — | — | — |  |  |
| No | -0.26 | 0.134 | -0.53, 0.01 | 0.061 |  |
| Satisfaction: Life in general |  |  |  |  | 0.838 |
| Scale value 1 to 3 | — | — | — |  |  |
| Scale value 4 to 7 | 0.07 | 0.343 | -0.62, 0.76 | 0.8 |  |
| Scale value 8 to 10 | -0.04 | 0.343 | -0.73, 0.65 | 0.9 |  |
| Self-rated mental health |  |  |  |  | 0.462 |
| Excellent/very good/good | — | — | — |  |  |
| fair/poor | -0.14 | 0.191 | -0.52, 0.24 | 0.5 |  |
| Sausage products |  |  |  |  | 0.21 |
| Daily or several times a day | — | — | — |  |  |
| 4 to 6 times per week | -0.46 | 0.284 | -1.0, 0.11 | 0.11 |  |
| 1 to 3 times per week | -0.27 | 0.263 | -0.80, 0.25 | 0.3 |  |
| Less than once per week | -0.18 | 0.263 | -0.71, 0.35 | 0.5 |  |
| Never | -0.64 | 0.319 | -1.3, 0.00 | 0.051 |  |
| BIK community size (categorized) |  |  |  |  | 0.671 |
| BIK region <20,000 inhabitants | — | — | — |  |  |
| BIK region 20,000 to <50,000 inhabitants OR surroundings 50,000 to <500,000 inhabitants | 0.09 | 0.326 | -0.57, 0.75 | 0.8 |  |
| Core city 50,000 to <500,000 inhabitants OR surroundings 500,000+ inhabitants | 0.32 | 0.331 | -0.35, 0.98 | 0.3 |  |
| Core city 500,000+ inhabitants | 0.29 | 0.331 | -0.38, 0.95 | 0.4 |  |
| Regions |  |  |  |  | >0.9 |
| Northeast | — | — | — |  |  |
| Northwest | 0.05 | 0.376 | -0.70, 0.81 | 0.9 |  |
| Central-East | -0.24 | 0.485 | -1.2, 0.74 | 0.6 |  |
| Central-West | 0.04 | 0.354 | -0.67, 0.76 | >0.9 |  |
| South | 0.04 | 0.356 | -0.68, 0.76 | >0.9 |  |
| Foreign nationality |  |  |  |  | 0.131 |
| Foreign national | — | — | — |  |  |
| Not foreign national | -0.33 | 0.216 | -0.76, 0.11 | 0.14 |  |
| Normal weight |  |  |  |  | 0.463 |
| Normal weight (18.5 <= BMI < 25) | — | — | — |  |  |
| Not normal weight (BMI < 18.5 or BMI >= 25) | -0.16 | 0.216 | -0.59, 0.28 | 0.5 |  |
| Age \* Overweight |  |  |  |  | 0.152 |
| 30-39 yrs \* No | 0.24 | 0.181 | -0.12, 0.61 | 0.2 |  |
| 40-49 yrs \* No | 0.18 | 0.188 | -0.20, 0.56 | 0.3 |  |
| 50-59 yrs \* No | 0.33 | 0.207 | -0.08, 0.75 | 0.12 |  |
| 60-69 yrs \* No | 0.17 | 0.224 | -0.28, 0.62 | 0.5 |  |
| 70-79 yrs \* No | -0.42 | 0.268 | -0.96, 0.12 | 0.13 |  |
| 80+ \* No | -0.29 | 0.286 | -0.87, 0.29 | 0.3 |  |
| Age \* Self-rated health |  |  |  |  | 0.244 |
| 30-39 yrs \* Bad/very bad | -0.36 | 0.633 | -1.6, 0.91 | 0.6 |  |
| 40-49 yrs \* Bad/very bad | -0.56 | 0.567 | -1.7, 0.58 | 0.3 |  |
| 50-59 yrs \* Bad/very bad | -0.48 | 0.516 | -1.5, 0.56 | 0.4 |  |
| 60-69 yrs \* Bad/very bad | -0.72 | 0.563 | -1.9, 0.42 | 0.2 |  |
| 70-79 yrs \* Bad/very bad | -1.3 | 0.532 | -2.4, -0.23 | 0.018 |  |
| 80+ \* Bad/very bad | -0.81 | 0.549 | -1.9, 0.30 | 0.15 |  |
| Age \* Chronic diseases |  |  |  |  | 0.118 |
| 30-39 yrs \* No | 0.11 | 0.194 | -0.28, 0.51 | 0.6 |  |
| 40-49 yrs \* No | 0.08 | 0.210 | -0.35, 0.50 | 0.7 |  |
| 50-59 yrs \* No | 0.18 | 0.203 | -0.23, 0.59 | 0.4 |  |
| 60-69 yrs \* No | 0.65 | 0.228 | 0.19, 1.1 | 0.007 |  |
| 70-79 yrs \* No | 0.18 | 0.269 | -0.36, 0.73 | 0.5 |  |
| 80+ \* No | -0.18 | 0.330 | -0.84, 0.49 | 0.6 |  |
| Age \* Satisfaction: Life in general |  |  |  |  | 0.43 |
| 30-39 yrs \* Scale value 4 to 7 | -0.07 | 0.382 | -0.84, 0.70 | 0.9 |  |
| 40-49 yrs \* Scale value 4 to 7 | 0.28 | 0.413 | -0.55, 1.1 | 0.5 |  |
| 50-59 yrs \* Scale value 4 to 7 | -0.12 | 0.382 | -0.89, 0.65 | 0.7 |  |
| 60-69 yrs \* Scale value 4 to 7 | -0.03 | 0.455 | -0.94, 0.89 | >0.9 |  |
| 70-79 yrs \* Scale value 4 to 7 | -0.25 | 0.485 | -1.2, 0.72 | 0.6 |  |
| 80+ \* Scale value 4 to 7 | 0.96 | 0.462 | 0.03, 1.9 | 0.044 |  |
| 30-39 yrs \* Scale value 8 to 10 | 0.04 | 0.432 | -0.84, 0.91 | >0.9 |  |
| 40-49 yrs \* Scale value 8 to 10 | 0.38 | 0.442 | -0.51, 1.3 | 0.4 |  |
| 50-59 yrs \* Scale value 8 to 10 | -0.25 | 0.410 | -1.1, 0.58 | 0.5 |  |
| 60-69 yrs \* Scale value 8 to 10 | 0.18 | 0.477 | -0.78, 1.1 | 0.7 |  |
| 70-79 yrs \* Scale value 8 to 10 | 0.00 | 0.503 | -1.0, 1.0 | >0.9 |  |
| 80+ \* Scale value 8 to 10 | 0.83 | 0.518 | -0.22, 1.9 | 0.12 |  |
| Age \* Self-rated mental health |  |  |  |  | 0.049 |
| 30-39 yrs \* fair/poor | 0.33 | 0.277 | -0.23, 0.89 | 0.2 |  |
| 40-49 yrs \* fair/poor | -0.06 | 0.277 | -0.62, 0.49 | 0.8 |  |
| 50-59 yrs \* fair/poor | 0.09 | 0.279 | -0.47, 0.65 | 0.7 |  |
| 60-69 yrs \* fair/poor | 0.79 | 0.302 | 0.19, 1.4 | 0.012 |  |
| 70-79 yrs \* fair/poor | 0.35 | 0.340 | -0.33, 1.0 | 0.3 |  |
| 80+ \* fair/poor | -0.42 | 0.384 | -1.2, 0.36 | 0.3 |  |
| Age \* Smoking |  |  |  |  | 0.304 |
| 30-39 yrs \* Occasional smoking | -0.21 | 0.380 | -0.97, 0.56 | 0.6 |  |
| 40-49 yrs \* Occasional smoking | 0.54 | 0.482 | -0.44, 1.5 | 0.3 |  |
| 50-59 yrs \* Occasional smoking | 0.10 | 0.437 | -0.78, 0.98 | 0.8 |  |
| 60-69 yrs \* Occasional smoking | 0.14 | 0.655 | -1.2, 1.5 | 0.8 |  |
| 70-79 yrs \* Occasional smoking | 0.24 | 0.782 | -1.3, 1.8 | 0.8 |  |
| 80+ \* Occasional smoking | -0.29 | 1.23 | -2.8, 2.2 | 0.8 |  |
| 30-39 yrs \* Non-smoker | 0.04 | 0.287 | -0.54, 0.62 | 0.9 |  |
| 40-49 yrs \* Non-smoker | 0.05 | 0.280 | -0.51, 0.61 | 0.9 |  |
| 50-59 yrs \* Non-smoker | 0.03 | 0.265 | -0.50, 0.57 | 0.9 |  |
| 60-69 yrs \* Non-smoker | -0.60 | 0.312 | -1.2, 0.03 | 0.060 |  |
| 70-79 yrs \* Non-smoker | 0.15 | 0.369 | -0.59, 0.89 | 0.7 |  |
| 80+ \* Non-smoker | -0.01 | 0.698 | -1.4, 1.4 | >0.9 |  |
| Age \* Sausage products |  |  |  |  | 0.404 |
| 30-39 yrs \* 4 to 6 times per week | 0.12 | 0.381 | -0.65, 0.88 | 0.8 |  |
| 40-49 yrs \* 4 to 6 times per week | 0.80 | 0.397 | 0.00, 1.6 | 0.050 |  |
| 50-59 yrs \* 4 to 6 times per week | 0.49 | 0.409 | -0.33, 1.3 | 0.2 |  |
| 60-69 yrs \* 4 to 6 times per week | -0.07 | 0.425 | -0.92, 0.79 | 0.9 |  |
| 70-79 yrs \* 4 to 6 times per week | 0.87 | 0.451 | -0.04, 1.8 | 0.061 |  |
| 80+ \* 4 to 6 times per week | 0.51 | 0.535 | -0.57, 1.6 | 0.3 |  |
| 30-39 yrs \* 1 to 3 times per week | -0.22 | 0.357 | -0.94, 0.50 | 0.5 |  |
| 40-49 yrs \* 1 to 3 times per week | 0.31 | 0.365 | -0.42, 1.0 | 0.4 |  |
| 50-59 yrs \* 1 to 3 times per week | 0.19 | 0.364 | -0.54, 0.93 | 0.6 |  |
| 60-69 yrs \* 1 to 3 times per week | -0.24 | 0.404 | -1.1, 0.57 | 0.6 |  |
| 70-79 yrs \* 1 to 3 times per week | 0.25 | 0.445 | -0.64, 1.1 | 0.6 |  |
| 80+ \* 1 to 3 times per week | -0.08 | 0.493 | -1.1, 0.92 | 0.9 |  |
| 30-39 yrs \* Less than once per week | -0.57 | 0.373 | -1.3, 0.18 | 0.13 |  |
| 40-49 yrs \* Less than once per week | 0.08 | 0.391 | -0.71, 0.87 | 0.8 |  |
| 50-59 yrs \* Less than once per week | 0.09 | 0.395 | -0.71, 0.88 | 0.8 |  |
| 60-69 yrs \* Less than once per week | -0.47 | 0.419 | -1.3, 0.38 | 0.3 |  |
| 70-79 yrs \* Less than once per week | -0.13 | 0.480 | -1.1, 0.84 | 0.8 |  |
| 80+ \* Less than once per week | -0.04 | 0.577 | -1.2, 1.1 | >0.9 |  |
| 30-39 yrs \* Never | -0.60 | 0.461 | -1.5, 0.33 | 0.2 |  |
| 40-49 yrs \* Never | 0.57 | 0.525 | -0.49, 1.6 | 0.3 |  |
| 50-59 yrs \* Never | 0.31 | 0.511 | -0.72, 1.3 | 0.5 |  |
| 60-69 yrs \* Never | -0.27 | 0.563 | -1.4, 0.86 | 0.6 |  |
| 70-79 yrs \* Never | 0.30 | 0.654 | -1.0, 1.6 | 0.7 |  |
| 80+ \* Never | 1.0 | 0.692 | -0.35, 2.4 | 0.14 |  |
| Age \* Red meat |  |  |  |  | 0.569 |
| 30-39 yrs \* 4 to 6 times per week | 0.16 | 0.529 | -0.91, 1.2 | 0.8 |  |
| 40-49 yrs \* 4 to 6 times per week | -0.35 | 0.545 | -1.4, 0.75 | 0.5 |  |
| 50-59 yrs \* 4 to 6 times per week | -0.73 | 0.707 | -2.2, 0.69 | 0.3 |  |
| 60-69 yrs \* 4 to 6 times per week | -0.44 | 0.815 | -2.1, 1.2 | 0.6 |  |
| 70-79 yrs \* 4 to 6 times per week | -1.3 | 1.20 | -3.8, 1.1 | 0.3 |  |
| 80+ \* 4 to 6 times per week | 0.76 | 1.18 | -1.6, 3.1 | 0.5 |  |
| 30-39 yrs \* 1 to 3 times per week | 0.11 | 0.491 | -0.88, 1.1 | 0.8 |  |
| 40-49 yrs \* 1 to 3 times per week | -0.55 | 0.496 | -1.5, 0.45 | 0.3 |  |
| 50-59 yrs \* 1 to 3 times per week | -0.51 | 0.674 | -1.9, 0.84 | 0.5 |  |
| 60-69 yrs \* 1 to 3 times per week | 0.04 | 0.763 | -1.5, 1.6 | >0.9 |  |
| 70-79 yrs \* 1 to 3 times per week | -1.1 | 1.14 | -3.4, 1.2 | 0.4 |  |
| 80+ \* 1 to 3 times per week | 0.38 | 1.06 | -1.8, 2.5 | 0.7 |  |
| 30-39 yrs \* Less than once per week | 0.41 | 0.508 | -0.61, 1.4 | 0.4 |  |
| 40-49 yrs \* Less than once per week | -0.28 | 0.497 | -1.3, 0.72 | 0.6 |  |
| 50-59 yrs \* Less than once per week | -0.75 | 0.692 | -2.1, 0.64 | 0.3 |  |
| 60-69 yrs \* Less than once per week | 0.00 | 0.783 | -1.6, 1.6 | >0.9 |  |
| 70-79 yrs \* Less than once per week | -1.2 | 1.18 | -3.5, 1.2 | 0.3 |  |
| 80+ \* Less than once per week | 0.73 | 1.12 | -1.5, 3.0 | 0.5 |  |
| 30-39 yrs \* Never | 0.84 | 0.623 | -0.41, 2.1 | 0.2 |  |
| 40-49 yrs \* Never | -0.25 | 0.615 | -1.5, 0.99 | 0.7 |  |
| 50-59 yrs \* Never | -0.65 | 0.802 | -2.3, 0.97 | 0.4 |  |
| 60-69 yrs \* Never | -0.09 | 0.915 | -1.9, 1.8 | >0.9 |  |
| 70-79 yrs \* Never | -1.7 | 1.22 | -4.1, 0.77 | 0.2 |  |
| 80+ \* Never | 0.06 | 1.20 | -2.4, 2.5 | >0.9 |  |
| Age \* Sport |  |  |  |  | 0.274 |
| 30-39 yrs \* Less than 1 hour per week | 0.69 | 0.309 | 0.07, 1.3 | 0.031 |  |
| 40-49 yrs \* Less than 1 hour per week | 0.32 | 0.356 | -0.39, 1.0 | 0.4 |  |
| 50-59 yrs \* Less than 1 hour per week | 0.40 | 0.342 | -0.29, 1.1 | 0.2 |  |
| 60-69 yrs \* Less than 1 hour per week | 0.54 | 0.365 | -0.19, 1.3 | 0.14 |  |
| 70-79 yrs \* Less than 1 hour per week | 0.35 | 0.415 | -0.49, 1.2 | 0.4 |  |
| 80+ \* Less than 1 hour per week | 0.03 | 0.468 | -0.91, 0.98 | >0.9 |  |
| 30-39 yrs \* 1 to less than 2 hours per week | 0.47 | 0.299 | -0.13, 1.1 | 0.12 |  |
| 40-49 yrs \* 1 to less than 2 hours per week | 0.28 | 0.333 | -0.39, 0.96 | 0.4 |  |
| 50-59 yrs \* 1 to less than 2 hours per week | 0.41 | 0.317 | -0.22, 1.1 | 0.2 |  |
| 60-69 yrs \* 1 to less than 2 hours per week | 0.59 | 0.343 | -0.10, 1.3 | 0.094 |  |
| 70-79 yrs \* 1 to less than 2 hours per week | 0.40 | 0.402 | -0.42, 1.2 | 0.3 |  |
| 80+ \* 1 to less than 2 hours per week | -0.03 | 0.415 | -0.87, 0.80 | >0.9 |  |
| 30-39 yrs \* 2 to less than 4 hours per week | 0.55 | 0.308 | -0.07, 1.2 | 0.079 |  |
| 40-49 yrs \* 2 to less than 4 hours per week | 0.46 | 0.339 | -0.23, 1.1 | 0.2 |  |
| 50-59 yrs \* 2 to less than 4 hours per week | 0.15 | 0.329 | -0.51, 0.81 | 0.6 |  |
| 60-69 yrs \* 2 to less than 4 hours per week | 0.38 | 0.353 | -0.33, 1.1 | 0.3 |  |
| 70-79 yrs \* 2 to less than 4 hours per week | 0.43 | 0.389 | -0.35, 1.2 | 0.3 |  |
| 80+ \* 2 to less than 4 hours per week | 0.21 | 0.526 | -0.85, 1.3 | 0.7 |  |
| 30-39 yrs \* 4 hours per week and more | 0.58 | 0.348 | -0.12, 1.3 | 0.10 |  |
| 40-49 yrs \* 4 hours per week and more | 0.21 | 0.344 | -0.48, 0.91 | 0.5 |  |
| 50-59 yrs \* 4 hours per week and more | 1.0 | 0.395 | 0.23, 1.8 | 0.013 |  |
| 60-69 yrs \* 4 hours per week and more | 1.6 | 0.453 | 0.69, 2.5 | <0.001 |  |
| 70-79 yrs \* 4 hours per week and more | 0.46 | 0.448 | -0.44, 1.4 | 0.3 |  |
| 80+ \* 4 hours per week and more | 0.45 | 0.686 | -0.93, 1.8 | 0.5 |  |
| Age \* BIK community size (categorized) |  |  |  |  | 0.023 |
| 30-39 yrs \* BIK region 20,000 to <50,000 inhabitants OR surroundings 50,000 to <500,000 inhabitants | 0.20 | 0.323 | -0.45, 0.85 | 0.5 |  |
| 40-49 yrs \* BIK region 20,000 to <50,000 inhabitants OR surroundings 50,000 to <500,000 inhabitants | -0.40 | 0.366 | -1.1, 0.34 | 0.3 |  |
| 50-59 yrs \* BIK region 20,000 to <50,000 inhabitants OR surroundings 50,000 to <500,000 inhabitants | 0.26 | 0.357 | -0.46, 0.98 | 0.5 |  |
| 60-69 yrs \* BIK region 20,000 to <50,000 inhabitants OR surroundings 50,000 to <500,000 inhabitants | -0.36 | 0.351 | -1.1, 0.34 | 0.3 |  |
| 70-79 yrs \* BIK region 20,000 to <50,000 inhabitants OR surroundings 50,000 to <500,000 inhabitants | -0.25 | 0.428 | -1.1, 0.62 | 0.6 |  |
| 80+ \* BIK region 20,000 to <50,000 inhabitants OR surroundings 50,000 to <500,000 inhabitants | 0.19 | 0.613 | -1.0, 1.4 | 0.8 |  |
| 30-39 yrs \* Core city 50,000 to <500,000 inhabitants OR surroundings 500,000+ inhabitants | -0.29 | 0.335 | -0.97, 0.38 | 0.4 |  |
| 40-49 yrs \* Core city 50,000 to <500,000 inhabitants OR surroundings 500,000+ inhabitants | -0.42 | 0.380 | -1.2, 0.34 | 0.3 |  |
| 50-59 yrs \* Core city 50,000 to <500,000 inhabitants OR surroundings 500,000+ inhabitants | 0.41 | 0.360 | -0.31, 1.1 | 0.3 |  |
| 60-69 yrs \* Core city 50,000 to <500,000 inhabitants OR surroundings 500,000+ inhabitants | -0.48 | 0.371 | -1.2, 0.27 | 0.2 |  |
| 70-79 yrs \* Core city 50,000 to <500,000 inhabitants OR surroundings 500,000+ inhabitants | -0.57 | 0.465 | -1.5, 0.37 | 0.2 |  |
| 80+ \* Core city 50,000 to <500,000 inhabitants OR surroundings 500,000+ inhabitants | 0.00 | 0.628 | -1.3, 1.3 | >0.9 |  |
| 30-39 yrs \* Core city 500,000+ inhabitants | -0.44 | 0.333 | -1.1, 0.23 | 0.2 |  |
| 40-49 yrs \* Core city 500,000+ inhabitants | -0.13 | 0.376 | -0.89, 0.63 | 0.7 |  |
| 50-59 yrs \* Core city 500,000+ inhabitants | 0.14 | 0.369 | -0.61, 0.88 | 0.7 |  |
| 60-69 yrs \* Core city 500,000+ inhabitants | -0.50 | 0.364 | -1.2, 0.24 | 0.2 |  |
| 70-79 yrs \* Core city 500,000+ inhabitants | -0.46 | 0.466 | -1.4, 0.48 | 0.3 |  |
| 80+ \* Core city 500,000+ inhabitants | -0.65 | 0.611 | -1.9, 0.58 | 0.3 |  |
| Age \* Regions |  |  |  |  | 0.176 |
| 30-39 yrs \* Northwest | 0.11 | 0.317 | -0.53, 0.75 | 0.7 |  |
| 40-49 yrs \* Northwest | 0.44 | 0.360 | -0.29, 1.2 | 0.2 |  |
| 50-59 yrs \* Northwest | 0.32 | 0.349 | -0.38, 1.0 | 0.4 |  |
| 60-69 yrs \* Northwest | 0.32 | 0.348 | -0.38, 1.0 | 0.4 |  |
| 70-79 yrs \* Northwest | -0.01 | 0.410 | -0.84, 0.82 | >0.9 |  |
| 80+ \* Northwest | 0.19 | 0.538 | -0.90, 1.3 | 0.7 |  |
| 30-39 yrs \* Central-East | -0.56 | 0.373 | -1.3, 0.20 | 0.14 |  |
| 40-49 yrs \* Central-East | 0.06 | 0.411 | -0.76, 0.89 | 0.9 |  |
| 50-59 yrs \* Central-East | 0.51 | 0.422 | -0.34, 1.4 | 0.2 |  |
| 60-69 yrs \* Central-East | 0.06 | 0.434 | -0.82, 0.93 | 0.9 |  |
| 70-79 yrs \* Central-East | -0.25 | 0.489 | -1.2, 0.73 | 0.6 |  |
| 80+ \* Central-East | -0.26 | 0.637 | -1.5, 1.0 | 0.7 |  |
| 30-39 yrs \* Central-West | 0.24 | 0.289 | -0.34, 0.83 | 0.4 |  |
| 40-49 yrs \* Central-West | 0.58 | 0.343 | -0.11, 1.3 | 0.10 |  |
| 50-59 yrs \* Central-West | 0.45 | 0.321 | -0.20, 1.1 | 0.2 |  |
| 60-69 yrs \* Central-West | 0.47 | 0.337 | -0.21, 1.1 | 0.2 |  |
| 70-79 yrs \* Central-West | 0.31 | 0.402 | -0.50, 1.1 | 0.5 |  |
| 80+ \* Central-West | -0.04 | 0.488 | -1.0, 0.94 | >0.9 |  |
| 30-39 yrs \* South | 0.06 | 0.305 | -0.55, 0.68 | 0.8 |  |
| 40-49 yrs \* South | 0.22 | 0.358 | -0.50, 0.94 | 0.5 |  |
| 50-59 yrs \* South | 0.80 | 0.350 | 0.10, 1.5 | 0.027 |  |
| 60-69 yrs \* South | 0.08 | 0.347 | -0.62, 0.78 | 0.8 |  |
| 70-79 yrs \* South | -0.15 | 0.396 | -0.95, 0.64 | 0.7 |  |
| 80+ \* South | -0.28 | 0.531 | -1.3, 0.79 | 0.6 |  |
| Age \* Country of birth |  |  |  |  | 0.479 |
| 30-39 yrs \* In another country | 0.49 | 0.384 | -0.29, 1.3 | 0.2 |  |
| 40-49 yrs \* In another country | 0.07 | 0.354 | -0.64, 0.79 | 0.8 |  |
| 50-59 yrs \* In another country | 0.51 | 0.394 | -0.28, 1.3 | 0.2 |  |
| 60-69 yrs \* In another country | 0.04 | 0.425 | -0.81, 0.90 | >0.9 |  |
| 70-79 yrs \* In another country | 0.48 | 0.523 | -0.57, 1.5 | 0.4 |  |
| 80+ \* In another country | 0.66 | 0.444 | -0.24, 1.6 | 0.14 |  |
| Age \* German nationality |  |  |  |  | 0.009 |
| 30-39 yrs \* No | -0.29 | 0.422 | -1.1, 0.56 | 0.5 |  |
| 40-49 yrs \* No | 0.07 | 0.426 | -0.78, 0.93 | 0.9 |  |
| 50-59 yrs \* No | -1.3 | 0.415 | -2.2, -0.49 | 0.003 |  |
| 60-69 yrs \* No | -0.92 | 0.606 | -2.1, 0.30 | 0.13 |  |
| 70-79 yrs \* No | -0.97 | 0.587 | -2.1, 0.21 | 0.11 |  |
| 80+ \* No | -0.88 | 0.989 | -2.9, 1.1 | 0.4 |  |
| Age \* Education: ISCED (2011) |  |  |  |  | 0.843 |
| 30-39 yrs \* Medium | -0.15 | 0.327 | -0.81, 0.51 | 0.7 |  |
| 40-49 yrs \* Medium | -0.52 | 0.349 | -1.2, 0.18 | 0.14 |  |
| 50-59 yrs \* Medium | 0.07 | 0.323 | -0.58, 0.72 | 0.8 |  |
| 60-69 yrs \* Medium | 0.01 | 0.330 | -0.65, 0.68 | >0.9 |  |
| 70-79 yrs \* Medium | 0.21 | 0.327 | -0.45, 0.87 | 0.5 |  |
| 80+ \* Medium | -0.03 | 0.365 | -0.77, 0.70 | >0.9 |  |
| 30-39 yrs \* High | -0.32 | 0.352 | -1.0, 0.39 | 0.4 |  |
| 40-49 yrs \* High | -0.81 | 0.353 | -1.5, -0.10 | 0.027 |  |
| 50-59 yrs \* High | -0.01 | 0.332 | -0.68, 0.66 | >0.9 |  |
| 60-69 yrs \* High | -0.16 | 0.348 | -0.86, 0.54 | 0.7 |  |
| 70-79 yrs \* High | 0.01 | 0.382 | -0.76, 0.78 | >0.9 |  |
| 80+ \* High | -0.04 | 0.427 | -0.90, 0.82 | >0.9 |  |
| Sex \* Self-rated health |  |  |  |  | >0.9 |
| Female \* Bad/very bad | 0.03 | 0.261 | -0.49, 0.56 | >0.9 |  |
| Sex \* Self-rated mental health |  |  |  |  | 0.389 |
| Female \* fair/poor | -0.14 | 0.157 | -0.45, 0.18 | 0.4 |  |
| Sex \* Sausage products |  |  |  |  | 0.147 |
| Female \* 4 to 6 times per week | 0.17 | 0.253 | -0.34, 0.68 | 0.5 |  |
| Female \* 1 to 3 times per week | -0.05 | 0.240 | -0.53, 0.43 | 0.8 |  |
| Female \* Less than once per week | 0.28 | 0.240 | -0.20, 0.76 | 0.3 |  |
| Female \* Never | 0.26 | 0.267 | -0.28, 0.80 | 0.3 |  |
| Sex \* Sport |  |  |  |  | 0.467 |
| Female \* Less than 1 hour per week | -0.19 | 0.191 | -0.57, 0.20 | 0.3 |  |
| Female \* 1 to less than 2 hours per week | 0.14 | 0.174 | -0.21, 0.49 | 0.4 |  |
| Female \* 2 to less than 4 hours per week | 0.10 | 0.194 | -0.29, 0.49 | 0.6 |  |
| Female \* 4 hours per week and more | 0.05 | 0.214 | -0.38, 0.48 | 0.8 |  |
| Sex \* Regions |  |  |  |  | 0.006 |
| Female \* Northwest | 0.21 | 0.161 | -0.11, 0.54 | 0.2 |  |
| Female \* Central-East | 0.07 | 0.180 | -0.29, 0.44 | 0.7 |  |
| Female \* Central-West | -0.36 | 0.146 | -0.65, -0.06 | 0.019 |  |
| Female \* South | -0.07 | 0.156 | -0.38, 0.25 | 0.7 |  |
| Sex \* Education: ISCED (2011) |  |  |  |  | 0.001 |
| Female \* Medium | 0.12 | 0.189 | -0.27, 0.50 | 0.5 |  |
| Female \* High | -0.35 | 0.194 | -0.74, 0.04 | 0.079 |  |
| Education: CASMIN \* Satisfaction: Life in general |  |  |  |  | 0.544 |
| Medium \* Scale value 4 to 7 | -0.19 | 0.285 | -0.76, 0.39 | 0.5 |  |
| High \* Scale value 4 to 7 | 0.05 | 0.320 | -0.59, 0.70 | 0.9 |  |
| Medium \* Scale value 8 to 10 | -0.13 | 0.290 | -0.71, 0.46 | 0.7 |  |
| High \* Scale value 8 to 10 | 0.26 | 0.326 | -0.40, 0.92 | 0.4 |  |
| Education: CASMIN \* Health risk due to climate change |  |  |  |  | 0.794 |
| Medium \* Scale value 4 to 7 | 0.20 | 0.164 | -0.13, 0.53 | 0.2 |  |
| High \* Scale value 4 to 7 | 0.13 | 0.175 | -0.23, 0.48 | 0.5 |  |
| Medium \* Scale value 8 to 10 | 0.16 | 0.249 | -0.34, 0.67 | 0.5 |  |
| High \* Scale value 8 to 10 | 0.02 | 0.247 | -0.48, 0.52 | >0.9 |  |
| Education: CASMIN \* Smoking |  |  |  |  | 0.548 |
| Medium \* Occasional smoking | -0.30 | 0.361 | -1.0, 0.42 | 0.4 |  |
| High \* Occasional smoking | -0.57 | 0.403 | -1.4, 0.24 | 0.2 |  |
| Medium \* Non-smoker | 0.09 | 0.195 | -0.31, 0.48 | 0.7 |  |
| High \* Non-smoker | -0.14 | 0.239 | -0.62, 0.34 | 0.6 |  |
| Education: CASMIN \* Red meat |  |  |  |  | 0.655 |
| Medium \* 4 to 6 times per week | -0.33 | 0.483 | -1.3, 0.64 | 0.5 |  |
| High \* 4 to 6 times per week | -0.68 | 0.528 | -1.7, 0.38 | 0.2 |  |
| Medium \* 1 to 3 times per week | -0.44 | 0.442 | -1.3, 0.45 | 0.3 |  |
| High \* 1 to 3 times per week | -0.50 | 0.481 | -1.5, 0.47 | 0.3 |  |
| Medium \* Less than once per week | -0.64 | 0.443 | -1.5, 0.25 | 0.2 |  |
| High \* Less than once per week | -0.66 | 0.490 | -1.6, 0.33 | 0.2 |  |
| Medium \* Never | -0.38 | 0.516 | -1.4, 0.66 | 0.5 |  |
| High \* Never | -0.49 | 0.544 | -1.6, 0.61 | 0.4 |  |
| Education: CASMIN \* Sport |  |  |  |  | 0.574 |
| Medium \* Less than 1 hour per week | -0.14 | 0.248 | -0.64, 0.36 | 0.6 |  |
| High \* Less than 1 hour per week | 0.19 | 0.274 | -0.36, 0.74 | 0.5 |  |
| Medium \* 1 to less than 2 hours per week | -0.35 | 0.224 | -0.80, 0.10 | 0.12 |  |
| High \* 1 to less than 2 hours per week | -0.11 | 0.252 | -0.62, 0.40 | 0.7 |  |
| Medium \* 2 to less than 4 hours per week | -0.22 | 0.275 | -0.77, 0.33 | 0.4 |  |
| High \* 2 to less than 4 hours per week | -0.13 | 0.281 | -0.70, 0.43 | 0.6 |  |
| Medium \* 4 hours per week and more | -0.37 | 0.301 | -0.97, 0.24 | 0.2 |  |
| High \* 4 hours per week and more | -0.18 | 0.316 | -0.81, 0.46 | 0.6 |  |
| Education: CASMIN \* Waited for medical examination date in the last 12 months |  |  |  |  | 0.272 |
| Medium \* No | -0.06 | 0.163 | -0.39, 0.27 | 0.7 |  |
| High \* No | 0.11 | 0.171 | -0.23, 0.46 | 0.5 |  |
| Medium \* No need for examination or treatment | -0.30 | 0.282 | -0.87, 0.26 | 0.3 |  |
| High \* No need for examination or treatment | -0.44 | 0.309 | -1.1, 0.19 | 0.2 |  |
| Education: CASMIN \* Foreign nationality |  |  |  |  | 0.374 |
| Medium \* Not foreign national | 0.08 | 0.248 | -0.42, 0.58 | 0.7 |  |
| High \* Not foreign national | 0.32 | 0.270 | -0.23, 0.86 | 0.2 |  |
| Education: CASMIN \* BIK community size (categorized) |  |  |  |  | 0.247 |
| Medium \* BIK region 20,000 to <50,000 inhabitants OR surroundings 50,000 to <500,000 inhabitants | -0.07 | 0.261 | -0.60, 0.45 | 0.8 |  |
| High \* BIK region 20,000 to <50,000 inhabitants OR surroundings 50,000 to <500,000 inhabitants | 0.51 | 0.285 | -0.06, 1.1 | 0.079 |  |
| Medium \* Core city 50,000 to <500,000 inhabitants OR surroundings 500,000+ inhabitants | -0.17 | 0.262 | -0.70, 0.36 | 0.5 |  |
| High \* Core city 50,000 to <500,000 inhabitants OR surroundings 500,000+ inhabitants | 0.40 | 0.284 | -0.17, 0.98 | 0.2 |  |
| Medium \* Core city 500,000+ inhabitants | -0.11 | 0.285 | -0.68, 0.47 | 0.7 |  |
| High \* Core city 500,000+ inhabitants | 0.49 | 0.297 | -0.10, 1.1 | 0.10 |  |
| Education: CASMIN \* Regions |  |  |  |  | 0.363 |
| Medium \* Northwest | -0.18 | 0.316 | -0.82, 0.45 | 0.6 |  |
| High \* Northwest | -0.43 | 0.327 | -1.1, 0.22 | 0.2 |  |
| Medium \* Central-East | 0.49 | 0.383 | -0.28, 1.3 | 0.2 |  |
| High \* Central-East | 0.46 | 0.376 | -0.30, 1.2 | 0.2 |  |
| Medium \* Central-West | 0.15 | 0.306 | -0.47, 0.76 | 0.6 |  |
| High \* Central-West | -0.01 | 0.299 | -0.62, 0.59 | >0.9 |  |
| Medium \* South | 0.19 | 0.308 | -0.43, 0.82 | 0.5 |  |
| High \* South | 0.05 | 0.305 | -0.56, 0.66 | 0.9 |  |
| Education: CASMIN \* Country of birth |  |  |  |  | 0.142 |
| Medium \* In another country | 0.49 | 0.263 | -0.04, 1.0 | 0.067 |  |
| High \* In another country | 0.47 | 0.286 | -0.10, 1.0 | 0.11 |  |
| Education: CASMIN \* Current living situation |  |  |  |  | 0.461 |
| Medium \* Part-time employed | 0.09 | 0.261 | -0.43, 0.62 | 0.7 |  |
| High \* Part-time employed | 0.30 | 0.281 | -0.27, 0.86 | 0.3 |  |
| Medium \* Unemployed | -0.53 | 0.360 | -1.3, 0.20 | 0.2 |  |
| High \* Unemployed | -0.15 | 0.414 | -0.98, 0.69 | 0.7 |  |
| Medium \* Retired or early retired | 0.21 | 0.210 | -0.22, 0.63 | 0.3 |  |
| High \* Retired or early retired | 0.27 | 0.280 | -0.30, 0.83 | 0.3 |  |
| Medium \* Not employed for other reasons (student, volunteer service, homemaker) | 0.09 | 0.283 | -0.48, 0.66 | 0.7 |  |
| High \* Not employed for other reasons (student, volunteer service, homemaker) | 0.47 | 0.317 | -0.17, 1.1 | 0.15 |  |
| Education: CASMIN \* Normal weight |  |  |  |  | 0.002 |
| Medium \* Not normal weight (BMI < 18.5 or BMI >= 25) | 0.43 | 0.166 | 0.10, 0.77 | 0.013 |  |
| High \* Not normal weight (BMI < 18.5 or BMI >= 25) | 0.64 | 0.184 | 0.27, 1.0 | 0.001 |  |
| N.Obs | 11,845 |  |  |  |  |
| N.Cluster | 359 |  |  |  |  |
|  |  |  |  |  |  |
| --- | --- | --- | --- | --- | --- |
| Abbreviations: CI = Confidence Interval, OR = Odds Ratio, SE = Standard Error | | | | | |

## Result model estimation: Questionaire D

### Quarter 2

| Characteristic | log(OR) | SE | 95% CI | p-value | p-value (global) |
| --- | --- | --- | --- | --- | --- |
| Sex |  |  |  |  | 0.69 |
| Male | — | — | — |  |  |
| Female | 0.10 | 0.243 | -0.39, 0.59 | 0.7 |  |
| Age |  |  |  |  | <0.001 |
| 16-29 yrs | — | — | — |  |  |
| 30-39 yrs | 0.26 | 0.813 | -1.4, 1.9 | 0.8 |  |
| 40-49 yrs | -0.54 | 0.901 | -2.4, 1.3 | 0.6 |  |
| 50-59 yrs | 1.3 | 1.01 | -0.75, 3.3 | 0.2 |  |
| 60-69 yrs | 1.2 | 1.06 | -0.93, 3.4 | 0.3 |  |
| 70-79 yrs | 0.78 | 1.22 | -1.7, 3.2 | 0.5 |  |
| 80+ | 13 | 1.31 | 10, 16 | <0.001 |  |
| Education: CASMIN |  |  |  |  | 0.425 |
| Low | — | — | — |  |  |
| Medium | 0.36 | 0.614 | -0.87, 1.6 | 0.6 |  |
| High | 1.0 | 0.806 | -0.59, 2.7 | 0.2 |  |
| Smoking |  |  |  |  | 0.021 |
| Daily smoking | — | — | — |  |  |
| Occasional smoking | 0.56 | 0.429 | -0.30, 1.4 | 0.2 |  |
| Non-smoker | 0.70 | 0.250 | 0.19, 1.2 | 0.008 |  |
| Red meat |  |  |  |  | 0.779 |
| Daily or several times a day | — | — | — |  |  |
| 4 to 6 times per week | -0.29 | 0.510 | -1.3, 0.74 | 0.6 |  |
| 1 to 3 times per week | -0.47 | 0.479 | -1.4, 0.50 | 0.3 |  |
| Less than once per week | -0.24 | 0.540 | -1.3, 0.85 | 0.7 |  |
| Never | -0.39 | 0.618 | -1.6, 0.86 | 0.5 |  |
| Interview type |  |  |  |  | 0.049 |
| Computer Assisted Web Interview | — | — | — |  |  |
| Paper and Pencil Interview | -0.67 | 0.338 | -1.3, 0.02 | 0.055 |  |
| Regions |  |  |  |  | 0.08 |
| Northeast | — | — | — |  |  |
| Northwest | 0.58 | 0.289 | 0.00, 1.2 | 0.052 |  |
| Central-East | 0.71 | 0.305 | 0.10, 1.3 | 0.024 |  |
| Central-West | 0.69 | 0.253 | 0.18, 1.2 | 0.009 |  |
| South | 0.63 | 0.260 | 0.11, 1.2 | 0.019 |  |
| Country of birth |  |  |  |  | 0.101 |
| In Germany (within current borders) | — | — | — |  |  |
| In another country | -0.48 | 0.290 | -1.1, 0.11 | 0.11 |  |
| German nationality |  |  |  |  | 0.298 |
| Yes | — | — | — |  |  |
| No | 0.38 | 0.367 | -0.36, 1.1 | 0.3 |  |
| Household size |  |  |  |  | 0.678 |
| Single-person household | — | — | — |  |  |
| Multi-person household | -0.11 | 0.255 | -0.62, 0.41 | 0.7 |  |
| Education: ISCED (2011) |  |  |  |  | >0.9 |
| Low | — | — | — |  |  |
| Medium | 0.01 | 0.192 | -0.38, 0.40 | >0.9 |  |
| High | 0.03 | 0.236 | -0.44, 0.51 | 0.9 |  |
| Current living situation |  |  |  |  | 0.107 |
| Full-time employed | — | — | — |  |  |
| Part-time employed | -0.38 | 0.251 | -0.89, 0.13 | 0.14 |  |
| Unemployed | -0.40 | 0.304 | -1.0, 0.22 | 0.2 |  |
| Retired or early retired | 0.24 | 0.221 | -0.20, 0.69 | 0.3 |  |
| Not employed for other reasons (student, volunteer service, homemaker) | 0.25 | 0.286 | -0.33, 0.82 | 0.4 |  |
| Self-rated health |  |  |  |  | >0.9 |
| Very good/good/fair | — | — | — |  |  |
| Bad/very bad | -0.02 | 0.535 | -1.1, 1.1 | >0.9 |  |
| Chronic diseases |  |  |  |  | 0.226 |
| Yes | — | — | — |  |  |
| No | -0.18 | 0.148 | -0.48, 0.12 | 0.2 |  |
| Satisfaction: Life in general |  |  |  |  | 0.251 |
| Scale value 1 to 3 | — | — | — |  |  |
| Scale value 4 to 7 | 0.46 | 0.369 | -0.28, 1.2 | 0.2 |  |
| Scale value 8 to 10 | 0.18 | 0.381 | -0.59, 0.95 | 0.6 |  |
| Health risk due to climate change |  |  |  |  | 0.279 |
| Scale value 1 to 3 | — | — | — |  |  |
| Scale value 4 to 7 | 0.32 | 0.218 | -0.12, 0.76 | 0.15 |  |
| Scale value 8 to 10 | 0.34 | 0.288 | -0.24, 0.92 | 0.2 |  |
| Self-rated mental health |  |  |  |  | 0.228 |
| Excellent/very good/good | — | — | — |  |  |
| fair/poor | 0.22 | 0.184 | -0.15, 0.59 | 0.2 |  |
| Sausage products |  |  |  |  | 0.687 |
| Daily or several times a day | — | — | — |  |  |
| 4 to 6 times per week | 0.20 | 0.371 | -0.55, 0.95 | 0.6 |  |
| 1 to 3 times per week | 0.36 | 0.322 | -0.29, 1.0 | 0.3 |  |
| Less than once per week | 0.49 | 0.360 | -0.24, 1.2 | 0.2 |  |
| Never | 0.48 | 0.497 | -0.52, 1.5 | 0.3 |  |
| Sport |  |  |  |  | 0.782 |
| No sporting activities | — | — | — |  |  |
| Less than 1 hour per week | -0.25 | 0.248 | -0.75, 0.25 | 0.3 |  |
| 1 to less than 2 hours per week | -0.25 | 0.233 | -0.72, 0.22 | 0.3 |  |
| 2 to less than 4 hours per week | -0.30 | 0.249 | -0.80, 0.20 | 0.2 |  |
| 4 hours per week and more | -0.29 | 0.245 | -0.78, 0.21 | 0.2 |  |
| Waited for medical examination date in the last 12 months |  |  |  |  | 0.187 |
| Yes | — | — | — |  |  |
| No | 0.35 | 0.209 | -0.07, 0.77 | 0.10 |  |
| No need for examination or treatment | 0.35 | 0.296 | -0.24, 0.95 | 0.2 |  |
| BIK community size (categorized) |  |  |  |  | 0.03 |
| BIK region <20,000 inhabitants | — | — | — |  |  |
| BIK region 20,000 to <50,000 inhabitants OR surroundings 50,000 to <500,000 inhabitants | -0.62 | 0.266 | -1.2, -0.09 | 0.024 |  |
| Core city 50,000 to <500,000 inhabitants OR surroundings 500,000+ inhabitants | -0.07 | 0.261 | -0.59, 0.46 | 0.8 |  |
| Core city 500,000+ inhabitants | -0.51 | 0.292 | -1.1, 0.08 | 0.088 |  |
| Overweight |  |  |  |  | 0.826 |
| Yes | — | — | — |  |  |
| No | -0.03 | 0.140 | -0.31, 0.25 | 0.8 |  |
| Age \* Self-rated health |  |  |  |  | 0.039 |
| 30-39 yrs \* Bad/very bad | 1.3 | 0.766 | -0.21, 2.9 | 0.088 |  |
| 40-49 yrs \* Bad/very bad | 0.40 | 0.820 | -1.3, 2.0 | 0.6 |  |
| 50-59 yrs \* Bad/very bad | -0.74 | 0.616 | -2.0, 0.50 | 0.2 |  |
| 60-69 yrs \* Bad/very bad | -0.01 | 0.660 | -1.3, 1.3 | >0.9 |  |
| 70-79 yrs \* Bad/very bad | -0.11 | 0.652 | -1.4, 1.2 | 0.9 |  |
| 80+ \* Bad/very bad | 0.06 | 0.663 | -1.3, 1.4 | >0.9 |  |
| Age \* Chronic diseases |  |  |  |  | 0.246 |
| 30-39 yrs \* No | 0.13 | 0.206 | -0.28, 0.55 | 0.5 |  |
| 40-49 yrs \* No | -0.14 | 0.233 | -0.61, 0.33 | 0.5 |  |
| 50-59 yrs \* No | 0.21 | 0.232 | -0.26, 0.68 | 0.4 |  |
| 60-69 yrs \* No | 0.45 | 0.261 | -0.08, 0.97 | 0.093 |  |
| 70-79 yrs \* No | 0.40 | 0.315 | -0.24, 1.0 | 0.2 |  |
| 80+ \* No | 0.51 | 0.343 | -0.18, 1.2 | 0.14 |  |
| Age \* Satisfaction: Life in general |  |  |  |  | 0.046 |
| 30-39 yrs \* Scale value 4 to 7 | -0.74 | 0.404 | -1.6, 0.07 | 0.073 |  |
| 40-49 yrs \* Scale value 4 to 7 | -0.71 | 0.423 | -1.6, 0.14 | 0.10 |  |
| 50-59 yrs \* Scale value 4 to 7 | 0.11 | 0.422 | -0.74, 0.96 | 0.8 |  |
| 60-69 yrs \* Scale value 4 to 7 | -0.42 | 0.503 | -1.4, 0.60 | 0.4 |  |
| 70-79 yrs \* Scale value 4 to 7 | 0.22 | 0.532 | -0.85, 1.3 | 0.7 |  |
| 80+ \* Scale value 4 to 7 | -0.03 | 0.547 | -1.1, 1.1 | >0.9 |  |
| 30-39 yrs \* Scale value 8 to 10 | -0.41 | 0.441 | -1.3, 0.48 | 0.4 |  |
| 40-49 yrs \* Scale value 8 to 10 | -0.41 | 0.450 | -1.3, 0.50 | 0.4 |  |
| 50-59 yrs \* Scale value 8 to 10 | 0.58 | 0.439 | -0.31, 1.5 | 0.2 |  |
| 60-69 yrs \* Scale value 8 to 10 | 0.18 | 0.527 | -0.88, 1.2 | 0.7 |  |
| 70-79 yrs \* Scale value 8 to 10 | 0.47 | 0.547 | -0.63, 1.6 | 0.4 |  |
| 80+ \* Scale value 8 to 10 | -0.37 | 0.598 | -1.6, 0.83 | 0.5 |  |
| Age \* Health risk due to climate change |  |  |  |  | 0.543 |
| 30-39 yrs \* Scale value 4 to 7 | -0.36 | 0.215 | -0.79, 0.07 | 0.10 |  |
| 40-49 yrs \* Scale value 4 to 7 | -0.04 | 0.214 | -0.47, 0.39 | 0.9 |  |
| 50-59 yrs \* Scale value 4 to 7 | -0.26 | 0.234 | -0.73, 0.21 | 0.3 |  |
| 60-69 yrs \* Scale value 4 to 7 | -0.12 | 0.247 | -0.62, 0.37 | 0.6 |  |
| 70-79 yrs \* Scale value 4 to 7 | -0.23 | 0.290 | -0.81, 0.36 | 0.4 |  |
| 80+ \* Scale value 4 to 7 | -0.16 | 0.314 | -0.79, 0.47 | 0.6 |  |
| 30-39 yrs \* Scale value 8 to 10 | 0.22 | 0.249 | -0.28, 0.72 | 0.4 |  |
| 40-49 yrs \* Scale value 8 to 10 | -0.06 | 0.271 | -0.60, 0.49 | 0.8 |  |
| 50-59 yrs \* Scale value 8 to 10 | 0.11 | 0.314 | -0.52, 0.74 | 0.7 |  |
| 60-69 yrs \* Scale value 8 to 10 | 0.06 | 0.320 | -0.59, 0.70 | 0.9 |  |
| 70-79 yrs \* Scale value 8 to 10 | 0.02 | 0.441 | -0.87, 0.90 | >0.9 |  |
| 80+ \* Scale value 8 to 10 | -0.58 | 0.466 | -1.5, 0.36 | 0.2 |  |
| Age \* Self-rated mental health |  |  |  |  | 0.008 |
| 30-39 yrs \* fair/poor | -0.27 | 0.262 | -0.80, 0.26 | 0.3 |  |
| 40-49 yrs \* fair/poor | 0.00 | 0.308 | -0.62, 0.62 | >0.9 |  |
| 50-59 yrs \* fair/poor | 0.47 | 0.304 | -0.15, 1.1 | 0.13 |  |
| 60-69 yrs \* fair/poor | 0.18 | 0.336 | -0.50, 0.85 | 0.6 |  |
| 70-79 yrs \* fair/poor | -0.88 | 0.357 | -1.6, -0.16 | 0.017 |  |
| 80+ \* fair/poor | -0.50 | 0.380 | -1.3, 0.27 | 0.2 |  |
| Age \* Smoking |  |  |  |  | 0.12 |
| 30-39 yrs \* Occasional smoking | -0.02 | 0.354 | -0.73, 0.70 | >0.9 |  |
| 40-49 yrs \* Occasional smoking | -1.2 | 0.424 | -2.0, -0.30 | 0.009 |  |
| 50-59 yrs \* Occasional smoking | -0.59 | 0.438 | -1.5, 0.29 | 0.2 |  |
| 60-69 yrs \* Occasional smoking | -0.82 | 0.491 | -1.8, 0.17 | 0.10 |  |
| 70-79 yrs \* Occasional smoking | -1.5 | 0.708 | -2.9, -0.03 | 0.046 |  |
| 80+ \* Occasional smoking | -2.2 | 1.40 | -5.0, 0.61 | 0.12 |  |
| 30-39 yrs \* Non-smoker | -0.30 | 0.245 | -0.79, 0.20 | 0.2 |  |
| 40-49 yrs \* Non-smoker | -0.86 | 0.279 | -1.4, -0.30 | 0.004 |  |
| 50-59 yrs \* Non-smoker | -0.56 | 0.275 | -1.1, -0.01 | 0.047 |  |
| 60-69 yrs \* Non-smoker | -0.34 | 0.278 | -0.90, 0.22 | 0.2 |  |
| 70-79 yrs \* Non-smoker | -0.72 | 0.424 | -1.6, 0.13 | 0.10 |  |
| 80+ \* Non-smoker | -1.2 | 0.683 | -2.6, 0.19 | 0.088 |  |
| Age \* Sausage products |  |  |  |  | 0.009 |
| 30-39 yrs \* 4 to 6 times per week | 0.13 | 0.382 | -0.64, 0.90 | 0.7 |  |
| 40-49 yrs \* 4 to 6 times per week | -0.03 | 0.354 | -0.74, 0.68 | >0.9 |  |
| 50-59 yrs \* 4 to 6 times per week | -0.48 | 0.401 | -1.3, 0.33 | 0.2 |  |
| 60-69 yrs \* 4 to 6 times per week | -0.29 | 0.556 | -1.4, 0.83 | 0.6 |  |
| 70-79 yrs \* 4 to 6 times per week | -0.07 | 0.520 | -1.1, 0.98 | 0.9 |  |
| 80+ \* 4 to 6 times per week | -0.19 | 0.547 | -1.3, 0.92 | 0.7 |  |
| 30-39 yrs \* 1 to 3 times per week | 0.10 | 0.369 | -0.64, 0.84 | 0.8 |  |
| 40-49 yrs \* 1 to 3 times per week | 0.28 | 0.349 | -0.42, 0.98 | 0.4 |  |
| 50-59 yrs \* 1 to 3 times per week | -0.75 | 0.394 | -1.5, 0.04 | 0.063 |  |
| 60-69 yrs \* 1 to 3 times per week | -1.3 | 0.491 | -2.3, -0.29 | 0.013 |  |
| 70-79 yrs \* 1 to 3 times per week | 0.05 | 0.464 | -0.89, 0.98 | >0.9 |  |
| 80+ \* 1 to 3 times per week | -0.37 | 0.473 | -1.3, 0.59 | 0.4 |  |
| 30-39 yrs \* Less than once per week | 0.25 | 0.386 | -0.53, 1.0 | 0.5 |  |
| 40-49 yrs \* Less than once per week | 0.15 | 0.383 | -0.62, 0.92 | 0.7 |  |
| 50-59 yrs \* Less than once per week | -0.48 | 0.399 | -1.3, 0.32 | 0.2 |  |
| 60-69 yrs \* Less than once per week | -0.67 | 0.548 | -1.8, 0.43 | 0.2 |  |
| 70-79 yrs \* Less than once per week | 0.21 | 0.524 | -0.85, 1.3 | 0.7 |  |
| 80+ \* Less than once per week | -0.39 | 0.509 | -1.4, 0.63 | 0.4 |  |
| 30-39 yrs \* Never | 0.37 | 0.507 | -0.66, 1.4 | 0.5 |  |
| 40-49 yrs \* Never | -0.14 | 0.533 | -1.2, 0.94 | 0.8 |  |
| 50-59 yrs \* Never | -0.85 | 0.488 | -1.8, 0.13 | 0.087 |  |
| 60-69 yrs \* Never | -2.1 | 0.649 | -3.4, -0.75 | 0.003 |  |
| 70-79 yrs \* Never | -0.56 | 0.742 | -2.1, 0.94 | 0.5 |  |
| 80+ \* Never | -0.91 | 0.667 | -2.3, 0.44 | 0.2 |  |
| Age \* Red meat |  |  |  |  | <0.001 |
| 30-39 yrs \* 4 to 6 times per week | -0.39 | 0.558 | -1.5, 0.74 | 0.5 |  |
| 40-49 yrs \* 4 to 6 times per week | 0.56 | 0.604 | -0.65, 1.8 | 0.4 |  |
| 50-59 yrs \* 4 to 6 times per week | -0.54 | 0.700 | -1.9, 0.87 | 0.4 |  |
| 60-69 yrs \* 4 to 6 times per week | 0.14 | 0.892 | -1.7, 1.9 | 0.9 |  |
| 70-79 yrs \* 4 to 6 times per week | 0.80 | 0.967 | -1.1, 2.7 | 0.4 |  |
| 80+ \* 4 to 6 times per week | -12 | 0.842 | -13, -10 | <0.001 |  |
| 30-39 yrs \* 1 to 3 times per week | -0.51 | 0.534 | -1.6, 0.56 | 0.3 |  |
| 40-49 yrs \* 1 to 3 times per week | 0.65 | 0.545 | -0.45, 1.7 | 0.2 |  |
| 50-59 yrs \* 1 to 3 times per week | -0.84 | 0.696 | -2.2, 0.56 | 0.2 |  |
| 60-69 yrs \* 1 to 3 times per week | 0.22 | 0.906 | -1.6, 2.0 | 0.8 |  |
| 70-79 yrs \* 1 to 3 times per week | 0.55 | 0.857 | -1.2, 2.3 | 0.5 |  |
| 80+ \* 1 to 3 times per week | -12 | 0.773 | -13, -10 | <0.001 |  |
| 30-39 yrs \* Less than once per week | -0.48 | 0.535 | -1.6, 0.60 | 0.4 |  |
| 40-49 yrs \* Less than once per week | 0.52 | 0.586 | -0.66, 1.7 | 0.4 |  |
| 50-59 yrs \* Less than once per week | -0.62 | 0.700 | -2.0, 0.79 | 0.4 |  |
| 60-69 yrs \* Less than once per week | 0.44 | 0.949 | -1.5, 2.4 | 0.6 |  |
| 70-79 yrs \* Less than once per week | 0.79 | 0.896 | -1.0, 2.6 | 0.4 |  |
| 80+ \* Less than once per week | -12 | 0.835 | -14, -11 | <0.001 |  |
| 30-39 yrs \* Never | -0.85 | 0.678 | -2.2, 0.52 | 0.2 |  |
| 40-49 yrs \* Never | 0.61 | 0.725 | -0.85, 2.1 | 0.4 |  |
| 50-59 yrs \* Never | -1.0 | 0.814 | -2.7, 0.62 | 0.2 |  |
| 60-69 yrs \* Never | -0.12 | 1.02 | -2.2, 1.9 | >0.9 |  |
| 70-79 yrs \* Never | 0.91 | 1.02 | -1.2, 3.0 | 0.4 |  |
| 80+ \* Never | -13 | 0.917 | -15, -11 | <0.001 |  |
| Age \* Sport |  |  |  |  | 0.171 |
| 30-39 yrs \* Less than 1 hour per week | 0.38 | 0.341 | -0.31, 1.1 | 0.3 |  |
| 40-49 yrs \* Less than 1 hour per week | 0.27 | 0.347 | -0.43, 0.97 | 0.4 |  |
| 50-59 yrs \* Less than 1 hour per week | 0.21 | 0.333 | -0.46, 0.88 | 0.5 |  |
| 60-69 yrs \* Less than 1 hour per week | -0.22 | 0.390 | -1.0, 0.57 | 0.6 |  |
| 70-79 yrs \* Less than 1 hour per week | 0.03 | 0.484 | -0.94, 1.0 | >0.9 |  |
| 80+ \* Less than 1 hour per week | 1.3 | 0.498 | 0.27, 2.3 | 0.014 |  |
| 30-39 yrs \* 1 to less than 2 hours per week | 0.16 | 0.327 | -0.50, 0.82 | 0.6 |  |
| 40-49 yrs \* 1 to less than 2 hours per week | 0.33 | 0.332 | -0.34, 1.0 | 0.3 |  |
| 50-59 yrs \* 1 to less than 2 hours per week | 0.30 | 0.300 | -0.31, 0.90 | 0.3 |  |
| 60-69 yrs \* 1 to less than 2 hours per week | 0.29 | 0.342 | -0.40, 0.98 | 0.4 |  |
| 70-79 yrs \* 1 to less than 2 hours per week | 0.01 | 0.393 | -0.78, 0.81 | >0.9 |  |
| 80+ \* 1 to less than 2 hours per week | 1.1 | 0.375 | 0.36, 1.9 | 0.005 |  |
| 30-39 yrs \* 2 to less than 4 hours per week | 0.49 | 0.344 | -0.20, 1.2 | 0.2 |  |
| 40-49 yrs \* 2 to less than 4 hours per week | 0.61 | 0.356 | -0.11, 1.3 | 0.094 |  |
| 50-59 yrs \* 2 to less than 4 hours per week | 0.31 | 0.364 | -0.43, 1.0 | 0.4 |  |
| 60-69 yrs \* 2 to less than 4 hours per week | 0.38 | 0.409 | -0.45, 1.2 | 0.4 |  |
| 70-79 yrs \* 2 to less than 4 hours per week | 0.04 | 0.430 | -0.82, 0.91 | >0.9 |  |
| 80+ \* 2 to less than 4 hours per week | 1.4 | 0.427 | 0.53, 2.2 | 0.002 |  |
| 30-39 yrs \* 4 hours per week and more | 0.30 | 0.367 | -0.44, 1.0 | 0.4 |  |
| 40-49 yrs \* 4 hours per week and more | 0.45 | 0.368 | -0.29, 1.2 | 0.2 |  |
| 50-59 yrs \* 4 hours per week and more | 0.49 | 0.403 | -0.33, 1.3 | 0.2 |  |
| 60-69 yrs \* 4 hours per week and more | 0.28 | 0.409 | -0.54, 1.1 | 0.5 |  |
| 70-79 yrs \* 4 hours per week and more | 0.24 | 0.516 | -0.80, 1.3 | 0.6 |  |
| 80+ \* 4 hours per week and more | 1.5 | 0.546 | 0.39, 2.6 | 0.009 |  |
| Age \* Waited for medical examination date in the last 12 months |  |  |  |  | 0.02 |
| 30-39 yrs \* No | -0.08 | 0.217 | -0.52, 0.35 | 0.7 |  |
| 40-49 yrs \* No | -0.02 | 0.210 | -0.44, 0.40 | >0.9 |  |
| 50-59 yrs \* No | 0.04 | 0.221 | -0.41, 0.49 | 0.9 |  |
| 60-69 yrs \* No | -0.35 | 0.247 | -0.85, 0.14 | 0.2 |  |
| 70-79 yrs \* No | -0.73 | 0.300 | -1.3, -0.12 | 0.019 |  |
| 80+ \* No | -0.46 | 0.346 | -1.2, 0.24 | 0.2 |  |
| 30-39 yrs \* No need for examination or treatment | 0.23 | 0.263 | -0.30, 0.76 | 0.4 |  |
| 40-49 yrs \* No need for examination or treatment | -0.09 | 0.298 | -0.69, 0.51 | 0.8 |  |
| 50-59 yrs \* No need for examination or treatment | -0.09 | 0.356 | -0.80, 0.63 | 0.8 |  |
| 60-69 yrs \* No need for examination or treatment | -0.09 | 0.467 | -1.0, 0.86 | 0.9 |  |
| 70-79 yrs \* No need for examination or treatment | 0.36 | 0.751 | -1.2, 1.9 | 0.6 |  |
| 80+ \* No need for examination or treatment | -1.9 | 0.629 | -3.1, -0.61 | 0.005 |  |
| Age \* Interview type |  |  |  |  | 0.218 |
| 30-39 yrs \* Paper and Pencil Interview | 0.37 | 0.375 | -0.39, 1.1 | 0.3 |  |
| 40-49 yrs \* Paper and Pencil Interview | 0.33 | 0.409 | -0.49, 1.2 | 0.4 |  |
| 50-59 yrs \* Paper and Pencil Interview | 0.52 | 0.368 | -0.23, 1.3 | 0.2 |  |
| 60-69 yrs \* Paper and Pencil Interview | 0.75 | 0.345 | 0.05, 1.4 | 0.036 |  |
| 70-79 yrs \* Paper and Pencil Interview | 0.56 | 0.389 | -0.22, 1.3 | 0.2 |  |
| 80+ \* Paper and Pencil Interview | 1.1 | 0.452 | 0.24, 2.1 | 0.015 |  |
| Age \* BIK community size (categorized) |  |  |  |  | 0.003 |
| 30-39 yrs \* BIK region 20,000 to <50,000 inhabitants OR surroundings 50,000 to <500,000 inhabitants | 0.42 | 0.320 | -0.22, 1.1 | 0.2 |  |
| 40-49 yrs \* BIK region 20,000 to <50,000 inhabitants OR surroundings 50,000 to <500,000 inhabitants | 1.1 | 0.410 | 0.32, 2.0 | 0.008 |  |
| 50-59 yrs \* BIK region 20,000 to <50,000 inhabitants OR surroundings 50,000 to <500,000 inhabitants | 0.84 | 0.334 | 0.17, 1.5 | 0.016 |  |
| 60-69 yrs \* BIK region 20,000 to <50,000 inhabitants OR surroundings 50,000 to <500,000 inhabitants | 0.68 | 0.371 | -0.07, 1.4 | 0.074 |  |
| 70-79 yrs \* BIK region 20,000 to <50,000 inhabitants OR surroundings 50,000 to <500,000 inhabitants | 0.41 | 0.429 | -0.46, 1.3 | 0.4 |  |
| 80+ \* BIK region 20,000 to <50,000 inhabitants OR surroundings 50,000 to <500,000 inhabitants | -0.19 | 0.464 | -1.1, 0.75 | 0.7 |  |
| 30-39 yrs \* Core city 50,000 to <500,000 inhabitants OR surroundings 500,000+ inhabitants | 0.38 | 0.340 | -0.31, 1.1 | 0.3 |  |
| 40-49 yrs \* Core city 50,000 to <500,000 inhabitants OR surroundings 500,000+ inhabitants | 0.85 | 0.412 | 0.02, 1.7 | 0.045 |  |
| 50-59 yrs \* Core city 50,000 to <500,000 inhabitants OR surroundings 500,000+ inhabitants | 0.22 | 0.338 | -0.46, 0.91 | 0.5 |  |
| 60-69 yrs \* Core city 50,000 to <500,000 inhabitants OR surroundings 500,000+ inhabitants | 0.34 | 0.403 | -0.47, 1.2 | 0.4 |  |
| 70-79 yrs \* Core city 50,000 to <500,000 inhabitants OR surroundings 500,000+ inhabitants | 0.22 | 0.441 | -0.67, 1.1 | 0.6 |  |
| 80+ \* Core city 50,000 to <500,000 inhabitants OR surroundings 500,000+ inhabitants | -0.26 | 0.481 | -1.2, 0.71 | 0.6 |  |
| 30-39 yrs \* Core city 500,000+ inhabitants | 0.71 | 0.332 | 0.04, 1.4 | 0.039 |  |
| 40-49 yrs \* Core city 500,000+ inhabitants | 1.4 | 0.393 | 0.65, 2.2 | <0.001 |  |
| 50-59 yrs \* Core city 500,000+ inhabitants | 0.60 | 0.323 | -0.06, 1.2 | 0.072 |  |
| 60-69 yrs \* Core city 500,000+ inhabitants | 1.1 | 0.389 | 0.31, 1.9 | 0.007 |  |
| 70-79 yrs \* Core city 500,000+ inhabitants | 0.22 | 0.437 | -0.66, 1.1 | 0.6 |  |
| 80+ \* Core city 500,000+ inhabitants | 0.71 | 0.503 | -0.30, 1.7 | 0.2 |  |
| Age \* Country of birth |  |  |  |  | 0.162 |
| 30-39 yrs \* In another country | 0.71 | 0.399 | -0.10, 1.5 | 0.083 |  |
| 40-49 yrs \* In another country | 0.22 | 0.394 | -0.57, 1.0 | 0.6 |  |
| 50-59 yrs \* In another country | -0.04 | 0.425 | -0.90, 0.82 | >0.9 |  |
| 60-69 yrs \* In another country | -0.51 | 0.471 | -1.5, 0.44 | 0.3 |  |
| 70-79 yrs \* In another country | -0.10 | 0.452 | -1.0, 0.81 | 0.8 |  |
| 80+ \* In another country | 0.37 | 0.454 | -0.55, 1.3 | 0.4 |  |
| Age \* German nationality |  |  |  |  | 0.33 |
| 30-39 yrs \* No | -0.92 | 0.487 | -1.9, 0.06 | 0.065 |  |
| 40-49 yrs \* No | -0.67 | 0.464 | -1.6, 0.26 | 0.2 |  |
| 50-59 yrs \* No | -0.59 | 0.545 | -1.7, 0.51 | 0.3 |  |
| 60-69 yrs \* No | 0.16 | 0.603 | -1.1, 1.4 | 0.8 |  |
| 70-79 yrs \* No | -0.94 | 0.641 | -2.2, 0.36 | 0.2 |  |
| 80+ \* No | -0.48 | 0.825 | -2.1, 1.2 | 0.6 |  |
| Age \* Household size |  |  |  |  | 0.616 |
| 30-39 yrs \* Multi-person household | 0.08 | 0.246 | -0.41, 0.58 | 0.7 |  |
| 40-49 yrs \* Multi-person household | -0.38 | 0.312 | -1.0, 0.25 | 0.2 |  |
| 50-59 yrs \* Multi-person household | -0.02 | 0.256 | -0.53, 0.50 | >0.9 |  |
| 60-69 yrs \* Multi-person household | -0.04 | 0.298 | -0.64, 0.57 | >0.9 |  |
| 70-79 yrs \* Multi-person household | -0.05 | 0.319 | -0.69, 0.59 | 0.9 |  |
| 80+ \* Multi-person household | 0.41 | 0.325 | -0.24, 1.1 | 0.2 |  |
| Age \* Education: ISCED (2011) |  |  |  |  | >0.9 |
| 30-39 yrs \* Medium | 0.16 | 0.396 | -0.64, 0.96 | 0.7 |  |
| 40-49 yrs \* Medium | 0.43 | 0.361 | -0.30, 1.2 | 0.2 |  |
| 50-59 yrs \* Medium | 0.08 | 0.366 | -0.66, 0.81 | 0.8 |  |
| 60-69 yrs \* Medium | 0.18 | 0.358 | -0.54, 0.90 | 0.6 |  |
| 70-79 yrs \* Medium | 0.49 | 0.399 | -0.32, 1.3 | 0.2 |  |
| 80+ \* Medium | 0.14 | 0.360 | -0.59, 0.87 | 0.7 |  |
| 30-39 yrs \* High | 0.11 | 0.415 | -0.73, 0.94 | 0.8 |  |
| 40-49 yrs \* High | 0.58 | 0.399 | -0.23, 1.4 | 0.2 |  |
| 50-59 yrs \* High | -0.03 | 0.392 | -0.82, 0.76 | >0.9 |  |
| 60-69 yrs \* High | 0.31 | 0.404 | -0.50, 1.1 | 0.4 |  |
| 70-79 yrs \* High | 0.77 | 0.457 | -0.15, 1.7 | 0.10 |  |
| 80+ \* High | 0.18 | 0.422 | -0.68, 1.0 | 0.7 |  |
| Sex \* Smoking |  |  |  |  | 0.012 |
| Female \* Occasional smoking | -0.10 | 0.283 | -0.67, 0.47 | 0.7 |  |
| Female \* Non-smoker | 0.37 | 0.165 | 0.04, 0.70 | 0.029 |  |
| Sex \* Interview type |  |  |  |  | 0.573 |
| Female \* Paper and Pencil Interview | -0.10 | 0.178 | -0.46, 0.26 | 0.6 |  |
| Sex \* Regions |  |  |  |  | 0.469 |
| Female \* Northwest | -0.16 | 0.232 | -0.63, 0.30 | 0.5 |  |
| Female \* Central-East | 0.01 | 0.242 | -0.48, 0.49 | >0.9 |  |
| Female \* Central-West | -0.13 | 0.209 | -0.55, 0.29 | 0.5 |  |
| Female \* South | 0.14 | 0.222 | -0.31, 0.59 | 0.5 |  |
| Sex \* German nationality |  |  |  |  | 0.043 |
| Female \* No | -0.42 | 0.206 | -0.83, 0.00 | 0.049 |  |
| Sex \* Current living situation |  |  |  |  | 0.633 |
| Female \* Part-time employed | 0.20 | 0.223 | -0.25, 0.65 | 0.4 |  |
| Female \* Unemployed | -0.23 | 0.336 | -0.90, 0.45 | 0.5 |  |
| Female \* Retired or early retired | -0.09 | 0.186 | -0.47, 0.28 | 0.6 |  |
| Female \* Not employed for other reasons (student, volunteer service, homemaker) | -0.15 | 0.225 | -0.60, 0.30 | 0.5 |  |
| Education: CASMIN \* Overweight |  |  |  |  | 0.396 |
| Medium \* No | 0.22 | 0.160 | -0.11, 0.54 | 0.2 |  |
| High \* No | 0.13 | 0.177 | -0.23, 0.49 | 0.5 |  |
| Education: CASMIN \* Satisfaction: Life in general |  |  |  |  | 0.786 |
| Medium \* Scale value 4 to 7 | -0.22 | 0.277 | -0.78, 0.34 | 0.4 |  |
| High \* Scale value 4 to 7 | -0.09 | 0.334 | -0.76, 0.59 | 0.8 |  |
| Medium \* Scale value 8 to 10 | -0.07 | 0.293 | -0.66, 0.52 | 0.8 |  |
| High \* Scale value 8 to 10 | 0.13 | 0.336 | -0.55, 0.80 | 0.7 |  |
| Education: CASMIN \* Health risk due to climate change |  |  |  |  | 0.868 |
| Medium \* Scale value 4 to 7 | -0.06 | 0.187 | -0.43, 0.32 | 0.8 |  |
| High \* Scale value 4 to 7 | -0.14 | 0.213 | -0.57, 0.29 | 0.5 |  |
| Medium \* Scale value 8 to 10 | -0.26 | 0.265 | -0.79, 0.27 | 0.3 |  |
| High \* Scale value 8 to 10 | -0.28 | 0.300 | -0.89, 0.32 | 0.4 |  |
| Education: CASMIN \* Smoking |  |  |  |  | 0.875 |
| Medium \* Occasional smoking | 0.16 | 0.374 | -0.59, 0.92 | 0.7 |  |
| High \* Occasional smoking | 0.10 | 0.433 | -0.77, 0.97 | 0.8 |  |
| Medium \* Non-smoker | 0.06 | 0.198 | -0.34, 0.46 | 0.8 |  |
| High \* Non-smoker | -0.16 | 0.288 | -0.74, 0.42 | 0.6 |  |
| Education: CASMIN \* Sausage products |  |  |  |  | 0.88 |
| Medium \* 4 to 6 times per week | -0.02 | 0.299 | -0.63, 0.58 | >0.9 |  |
| High \* 4 to 6 times per week | 0.16 | 0.390 | -0.62, 0.95 | 0.7 |  |
| Medium \* 1 to 3 times per week | -0.12 | 0.265 | -0.66, 0.41 | 0.6 |  |
| High \* 1 to 3 times per week | -0.03 | 0.348 | -0.73, 0.67 | >0.9 |  |
| Medium \* Less than once per week | -0.39 | 0.298 | -0.99, 0.22 | 0.2 |  |
| High \* Less than once per week | -0.31 | 0.360 | -1.0, 0.41 | 0.4 |  |
| Medium \* Never | -0.21 | 0.433 | -1.1, 0.66 | 0.6 |  |
| High \* Never | -0.08 | 0.483 | -1.0, 0.90 | 0.9 |  |
| Education: CASMIN \* Red meat |  |  |  |  | 0.312 |
| Medium \* 4 to 6 times per week | 0.50 | 0.478 | -0.46, 1.5 | 0.3 |  |
| High \* 4 to 6 times per week | -0.13 | 0.581 | -1.3, 1.0 | 0.8 |  |
| Medium \* 1 to 3 times per week | 0.75 | 0.472 | -0.21, 1.7 | 0.12 |  |
| High \* 1 to 3 times per week | 0.42 | 0.571 | -0.73, 1.6 | 0.5 |  |
| Medium \* Less than once per week | 0.64 | 0.507 | -0.38, 1.7 | 0.2 |  |
| High \* Less than once per week | 0.07 | 0.605 | -1.2, 1.3 | >0.9 |  |
| Medium \* Never | 0.87 | 0.559 | -0.25, 2.0 | 0.13 |  |
| High \* Never | 0.49 | 0.620 | -0.76, 1.7 | 0.4 |  |
| Education: CASMIN \* Waited for medical examination date in the last 12 months |  |  |  |  | 0.713 |
| Medium \* No | -0.12 | 0.179 | -0.48, 0.24 | 0.5 |  |
| High \* No | -0.27 | 0.214 | -0.70, 0.16 | 0.2 |  |
| Medium \* No need for examination or treatment | -0.20 | 0.281 | -0.77, 0.36 | 0.5 |  |
| High \* No need for examination or treatment | -0.24 | 0.332 | -0.91, 0.42 | 0.5 |  |
| Education: CASMIN \* Interview type |  |  |  |  | 0.135 |
| Medium \* Paper and Pencil Interview | -0.42 | 0.211 | -0.85, 0.00 | 0.052 |  |
| High \* Paper and Pencil Interview | -0.22 | 0.266 | -0.76, 0.31 | 0.4 |  |
| Education: CASMIN \* BIK community size (categorized) |  |  |  |  | 0.119 |
| Medium \* BIK region 20,000 to <50,000 inhabitants OR surroundings 50,000 to <500,000 inhabitants | 0.41 | 0.218 | -0.03, 0.85 | 0.067 |  |
| High \* BIK region 20,000 to <50,000 inhabitants OR surroundings 50,000 to <500,000 inhabitants | 0.18 | 0.276 | -0.37, 0.74 | 0.5 |  |
| Medium \* Core city 50,000 to <500,000 inhabitants OR surroundings 500,000+ inhabitants | -0.08 | 0.233 | -0.55, 0.39 | 0.7 |  |
| High \* Core city 50,000 to <500,000 inhabitants OR surroundings 500,000+ inhabitants | 0.01 | 0.280 | -0.56, 0.57 | >0.9 |  |
| Medium \* Core city 500,000+ inhabitants | 0.17 | 0.258 | -0.35, 0.69 | 0.5 |  |
| High \* Core city 500,000+ inhabitants | -0.10 | 0.292 | -0.69, 0.49 | 0.7 |  |
| Education: CASMIN \* Regions |  |  |  |  | 0.172 |
| Medium \* Northwest | -0.58 | 0.275 | -1.1, -0.03 | 0.040 |  |
| High \* Northwest | -0.64 | 0.279 | -1.2, -0.07 | 0.027 |  |
| Medium \* Central-East | -0.62 | 0.351 | -1.3, 0.08 | 0.082 |  |
| High \* Central-East | -0.58 | 0.380 | -1.3, 0.19 | 0.13 |  |
| Medium \* Central-West | -0.69 | 0.283 | -1.3, -0.12 | 0.019 |  |
| High \* Central-West | -1.0 | 0.319 | -1.6, -0.36 | 0.003 |  |
| Medium \* South | -0.56 | 0.282 | -1.1, 0.01 | 0.055 |  |
| High \* South | -0.54 | 0.300 | -1.1, 0.06 | 0.077 |  |
| Education: CASMIN \* Household size |  |  |  |  | 0.64 |
| Medium \* Multi-person household | -0.06 | 0.207 | -0.48, 0.36 | 0.8 |  |
| High \* Multi-person household | 0.12 | 0.245 | -0.38, 0.61 | 0.6 |  |
| Education: CASMIN \* Current living situation |  |  |  |  | 0.489 |
| Medium \* Part-time employed | 0.22 | 0.242 | -0.27, 0.71 | 0.4 |  |
| High \* Part-time employed | 0.45 | 0.255 | -0.07, 0.96 | 0.085 |  |
| Medium \* Unemployed | 0.46 | 0.348 | -0.24, 1.2 | 0.2 |  |
| High \* Unemployed | 0.23 | 0.473 | -0.73, 1.2 | 0.6 |  |
| Medium \* Retired or early retired | -0.02 | 0.245 | -0.52, 0.47 | >0.9 |  |
| High \* Retired or early retired | 0.13 | 0.315 | -0.51, 0.76 | 0.7 |  |
| Medium \* Not employed for other reasons (student, volunteer service, homemaker) | -0.03 | 0.287 | -0.61, 0.55 | >0.9 |  |
| High \* Not employed for other reasons (student, volunteer service, homemaker) | -0.20 | 0.342 | -0.89, 0.49 | 0.6 |  |
| N.Obs | 11,897 |  |  |  |  |
| N.Cluster | 359 |  |  |  |  |
|  |  |  |  |  |  |
| --- | --- | --- | --- | --- | --- |
| Abbreviations: CI = Confidence Interval, OR = Odds Ratio, SE = Standard Error | | | | | |

### Quarter 3

| Characteristic | log(OR) | SE | 95% CI | p-value | p-value (global) |
| --- | --- | --- | --- | --- | --- |
| Sex |  |  |  |  | 0.034 |
| Male | — | — | — |  |  |
| Female | 0.85 | 0.402 | -0.27, 2.0 | 0.10 |  |
| Age |  |  |  |  | 0.243 |
| 16-29 yrs | — | — | — |  |  |
| 30-39 yrs | 1.1 | 0.857 | -1.2, 3.5 | 0.3 |  |
| 40-49 yrs | 1.5 | 0.878 | -0.95, 3.9 | 0.2 |  |
| 50-59 yrs | 1.9 | 0.984 | -0.87, 4.6 | 0.13 |  |
| 60-69 yrs | 1.4 | 0.892 | -1.1, 3.8 | 0.2 |  |
| 70-79 yrs | 3.5 | 1.48 | -0.63, 7.6 | 0.078 |  |
| 80+ | 1.1 | 1.48 | -3.0, 5.2 | 0.5 |  |
| Overweight |  |  |  |  | 0.752 |
| Yes | — | — | — |  |  |
| No | 0.07 | 0.211 | -0.52, 0.65 | 0.8 |  |
| Self-rated health |  |  |  |  | 0.075 |
| Very good/good/fair | — | — | — |  |  |
| Bad/very bad | 0.82 | 0.459 | -0.46, 2.1 | 0.15 |  |
| Satisfaction: Life in general |  |  |  |  | 0.157 |
| Scale value 1 to 3 | — | — | — |  |  |
| Scale value 4 to 7 | 0.81 | 0.423 | -0.37, 2.0 | 0.13 |  |
| Scale value 8 to 10 | 0.80 | 0.433 | -0.40, 2.0 | 0.14 |  |
| Smoking |  |  |  |  | 0.021 |
| Daily smoking | — | — | — |  |  |
| Occasional smoking | 0.39 | 0.373 | -0.65, 1.4 | 0.4 |  |
| Non-smoker | 0.66 | 0.235 | 0.00, 1.3 | 0.050 |  |
| Red meat |  |  |  |  | 0.154 |
| Daily or several times a day | — | — | — |  |  |
| 4 to 6 times per week | 0.82 | 0.484 | -0.52, 2.2 | 0.2 |  |
| 1 to 3 times per week | 0.92 | 0.426 | -0.26, 2.1 | 0.10 |  |
| Less than once per week | 1.0 | 0.427 | -0.14, 2.2 | 0.070 |  |
| Never | 1.2 | 0.533 | -0.31, 2.7 | 0.093 |  |
| Sport |  |  |  |  | >0.9 |
| No sporting activities | — | — | — |  |  |
| Less than 1 hour per week | -0.02 | 0.319 | -0.91, 0.87 | >0.9 |  |
| 1 to less than 2 hours per week | -0.22 | 0.317 | -1.1, 0.66 | 0.5 |  |
| 2 to less than 4 hours per week | -0.17 | 0.339 | -1.1, 0.77 | 0.6 |  |
| 4 hours per week and more | -0.04 | 0.342 | -0.99, 0.91 | >0.9 |  |
| Country of birth |  |  |  |  | 0.153 |
| In Germany (within current borders) | — | — | — |  |  |
| In another country | -0.40 | 0.278 | -1.2, 0.37 | 0.2 |  |
| German nationality |  |  |  |  | 0.857 |
| Yes | — | — | — |  |  |
| No | -0.06 | 0.325 | -0.96, 0.84 | 0.9 |  |
| Education: ISCED (2011) |  |  |  |  | 0.006 |
| Low | — | — | — |  |  |
| Medium | 0.21 | 0.209 | -0.37, 0.79 | 0.4 |  |
| High | 0.72 | 0.255 | 0.01, 1.4 | 0.048 |  |
| Current living situation |  |  |  |  | 0.69 |
| Full-time employed | — | — | — |  |  |
| Part-time employed | 0.00 | 0.215 | -0.60, 0.60 | >0.9 |  |
| Unemployed | -0.26 | 0.266 | -1.0, 0.48 | 0.4 |  |
| Retired or early retired | 0.04 | 0.196 | -0.51, 0.58 | 0.9 |  |
| Not employed for other reasons (student, volunteer service, homemaker) | -0.28 | 0.254 | -0.98, 0.43 | 0.3 |  |
| Normal weight |  |  |  |  | 0.874 |
| Normal weight (18.5 <= BMI < 25) | — | — | — |  |  |
| Not normal weight (BMI < 18.5 or BMI >= 25) | 0.03 | 0.179 | -0.47, 0.53 | 0.9 |  |
| Obesity |  |  |  |  | >0.9 |
| Yes | — | — | — |  |  |
| No | 0.02 | 0.267 | -0.72, 0.76 | >0.9 |  |
| Chronic diseases |  |  |  |  | 0.481 |
| Yes | — | — | — |  |  |
| No | 0.10 | 0.148 | -0.31, 0.51 | 0.5 |  |
| Health risk due to climate change |  |  |  |  | >0.9 |
| Scale value 1 to 3 | — | — | — |  |  |
| Scale value 4 to 7 | -0.10 | 0.213 | -0.69, 0.50 | 0.7 |  |
| Scale value 8 to 10 | -0.04 | 0.286 | -0.83, 0.76 | 0.9 |  |
| Self-rated mental health |  |  |  |  | 0.78 |
| Excellent/very good/good | — | — | — |  |  |
| fair/poor | -0.07 | 0.252 | -0.77, 0.63 | 0.8 |  |
| Sausage products |  |  |  |  | 0.361 |
| Daily or several times a day | — | — | — |  |  |
| 4 to 6 times per week | -0.37 | 0.379 | -1.4, 0.68 | 0.4 |  |
| 1 to 3 times per week | -0.41 | 0.356 | -1.4, 0.58 | 0.3 |  |
| Less than once per week | -0.50 | 0.348 | -1.5, 0.47 | 0.2 |  |
| Never | -0.89 | 0.438 | -2.1, 0.33 | 0.11 |  |
| BIK community size (categorized) |  |  |  |  | 0.641 |
| BIK region <20,000 inhabitants | — | — | — |  |  |
| BIK region 20,000 to <50,000 inhabitants OR surroundings 50,000 to <500,000 inhabitants | 0.13 | 0.350 | -0.84, 1.1 | 0.7 |  |
| Core city 50,000 to <500,000 inhabitants OR surroundings 500,000+ inhabitants | 0.19 | 0.370 | -0.84, 1.2 | 0.6 |  |
| Core city 500,000+ inhabitants | 0.38 | 0.363 | -0.62, 1.4 | 0.3 |  |
| Regions |  |  |  |  | 0.616 |
| Northeast | — | — | — |  |  |
| Northwest | 0.45 | 0.335 | -0.48, 1.4 | 0.2 |  |
| Central-East | 0.32 | 0.397 | -0.78, 1.4 | 0.5 |  |
| Central-West | 0.49 | 0.316 | -0.39, 1.4 | 0.2 |  |
| South | 0.32 | 0.327 | -0.59, 1.2 | 0.4 |  |
| Household size |  |  |  |  | 0.734 |
| Single-person household | — | — | — |  |  |
| Multi-person household | -0.05 | 0.161 | -0.50, 0.39 | 0.8 |  |
| Education: CASMIN |  |  |  |  | 0.124 |
| Low | — | — | — |  |  |
| Medium | 1.3 | 0.644 | -0.52, 3.1 | 0.12 |  |
| High | 0.59 | 0.790 | -1.6, 2.8 | 0.5 |  |
| Paying attention to health |  |  |  |  | 0.53 |
| Not at all/less strong/moderate | — | — | — |  |  |
| Strong/very strong | -0.07 | 0.116 | -0.40, 0.25 | 0.6 |  |
| Waited for medical examination date in the last 12 months |  |  |  |  | 0.136 |
| Yes | — | — | — |  |  |
| No | 0.12 | 0.134 | -0.25, 0.49 | 0.4 |  |
| No need for examination or treatment | 0.49 | 0.248 | -0.20, 1.2 | 0.12 |  |
| Interview type |  |  |  |  | 0.29 |
| Computer Assisted Web Interview | — | — | — |  |  |
| Paper and Pencil Interview | 0.16 | 0.149 | -0.26, 0.57 | 0.4 |  |
| Age \* Obesity |  |  |  |  | 0.606 |
| 30-39 yrs \* No | 0.04 | 0.283 | -0.74, 0.83 | 0.9 |  |
| 40-49 yrs \* No | 0.02 | 0.289 | -0.78, 0.83 | >0.9 |  |
| 50-59 yrs \* No | 0.13 | 0.277 | -0.64, 0.90 | 0.7 |  |
| 60-69 yrs \* No | -0.08 | 0.278 | -0.85, 0.69 | 0.8 |  |
| 70-79 yrs \* No | 0.32 | 0.340 | -0.63, 1.3 | 0.4 |  |
| 80+ \* No | -0.57 | 0.450 | -1.8, 0.68 | 0.3 |  |
| Age \* Overweight |  |  |  |  | 0.048 |
| 30-39 yrs \* No | 0.06 | 0.210 | -0.53, 0.64 | 0.8 |  |
| 40-49 yrs \* No | 0.26 | 0.207 | -0.31, 0.84 | 0.3 |  |
| 50-59 yrs \* No | 0.29 | 0.214 | -0.31, 0.88 | 0.3 |  |
| 60-69 yrs \* No | 0.70 | 0.246 | 0.02, 1.4 | 0.046 |  |
| 70-79 yrs \* No | -0.16 | 0.289 | -0.96, 0.65 | 0.6 |  |
| 80+ \* No | 0.40 | 0.315 | -0.47, 1.3 | 0.3 |  |
| Age \* Self-rated health |  |  |  |  | 0.164 |
| 30-39 yrs \* Bad/very bad | -0.75 | 0.610 | -2.4, 0.95 | 0.3 |  |
| 40-49 yrs \* Bad/very bad | -0.59 | 0.572 | -2.2, 1.0 | 0.4 |  |
| 50-59 yrs \* Bad/very bad | -0.27 | 0.546 | -1.8, 1.2 | 0.7 |  |
| 60-69 yrs \* Bad/very bad | -0.63 | 0.557 | -2.2, 0.92 | 0.3 |  |
| 70-79 yrs \* Bad/very bad | -1.1 | 0.532 | -2.6, 0.37 | 0.11 |  |
| 80+ \* Bad/very bad | -1.4 | 0.564 | -2.9, 0.20 | 0.073 |  |
| Age \* Chronic diseases |  |  |  |  | 0.286 |
| 30-39 yrs \* No | -0.09 | 0.183 | -0.59, 0.42 | 0.7 |  |
| 40-49 yrs \* No | -0.16 | 0.206 | -0.73, 0.41 | 0.5 |  |
| 50-59 yrs \* No | -0.33 | 0.198 | -0.88, 0.22 | 0.2 |  |
| 60-69 yrs \* No | 0.03 | 0.214 | -0.57, 0.62 | >0.9 |  |
| 70-79 yrs \* No | -0.12 | 0.268 | -0.86, 0.63 | 0.7 |  |
| 80+ \* No | -0.69 | 0.325 | -1.6, 0.21 | 0.10 |  |
| Age \* Satisfaction: Life in general |  |  |  |  | >0.9 |
| 30-39 yrs \* Scale value 4 to 7 | -0.40 | 0.376 | -1.4, 0.64 | 0.3 |  |
| 40-49 yrs \* Scale value 4 to 7 | -0.43 | 0.418 | -1.6, 0.73 | 0.4 |  |
| 50-59 yrs \* Scale value 4 to 7 | -0.79 | 0.446 | -2.0, 0.45 | 0.2 |  |
| 60-69 yrs \* Scale value 4 to 7 | -0.66 | 0.493 | -2.0, 0.71 | 0.3 |  |
| 70-79 yrs \* Scale value 4 to 7 | -0.68 | 0.510 | -2.1, 0.73 | 0.3 |  |
| 80+ \* Scale value 4 to 7 | -0.22 | 0.548 | -1.7, 1.3 | 0.7 |  |
| 30-39 yrs \* Scale value 8 to 10 | -0.39 | 0.408 | -1.5, 0.75 | 0.4 |  |
| 40-49 yrs \* Scale value 8 to 10 | -0.31 | 0.425 | -1.5, 0.87 | 0.5 |  |
| 50-59 yrs \* Scale value 8 to 10 | -0.87 | 0.472 | -2.2, 0.44 | 0.14 |  |
| 60-69 yrs \* Scale value 8 to 10 | -0.59 | 0.507 | -2.0, 0.81 | 0.3 |  |
| 70-79 yrs \* Scale value 8 to 10 | -0.46 | 0.543 | -2.0, 1.0 | 0.4 |  |
| 80+ \* Scale value 8 to 10 | -0.30 | 0.619 | -2.0, 1.4 | 0.7 |  |
| Age \* Health risk due to climate change |  |  |  |  | >0.9 |
| 30-39 yrs \* Scale value 4 to 7 | -0.30 | 0.209 | -0.88, 0.28 | 0.2 |  |
| 40-49 yrs \* Scale value 4 to 7 | -0.42 | 0.235 | -1.1, 0.23 | 0.15 |  |
| 50-59 yrs \* Scale value 4 to 7 | -0.20 | 0.224 | -0.82, 0.42 | 0.4 |  |
| 60-69 yrs \* Scale value 4 to 7 | -0.10 | 0.247 | -0.79, 0.58 | 0.7 |  |
| 70-79 yrs \* Scale value 4 to 7 | -0.06 | 0.299 | -0.89, 0.77 | 0.8 |  |
| 80+ \* Scale value 4 to 7 | -0.10 | 0.335 | -1.0, 0.83 | 0.8 |  |
| 30-39 yrs \* Scale value 8 to 10 | -0.28 | 0.256 | -0.99, 0.42 | 0.3 |  |
| 40-49 yrs \* Scale value 8 to 10 | -0.52 | 0.281 | -1.3, 0.26 | 0.14 |  |
| 50-59 yrs \* Scale value 8 to 10 | -0.36 | 0.302 | -1.2, 0.48 | 0.3 |  |
| 60-69 yrs \* Scale value 8 to 10 | -0.27 | 0.325 | -1.2, 0.64 | 0.5 |  |
| 70-79 yrs \* Scale value 8 to 10 | -0.07 | 0.388 | -1.1, 1.0 | 0.9 |  |
| 80+ \* Scale value 8 to 10 | -0.31 | 0.464 | -1.6, 0.97 | 0.5 |  |
| Age \* Self-rated mental health |  |  |  |  | 0.129 |
| 30-39 yrs \* fair/poor | 0.13 | 0.283 | -0.66, 0.91 | 0.7 |  |
| 40-49 yrs \* fair/poor | -0.36 | 0.274 | -1.1, 0.40 | 0.3 |  |
| 50-59 yrs \* fair/poor | 0.02 | 0.286 | -0.78, 0.81 | >0.9 |  |
| 60-69 yrs \* fair/poor | 0.45 | 0.300 | -0.38, 1.3 | 0.2 |  |
| 70-79 yrs \* fair/poor | -0.16 | 0.333 | -1.1, 0.77 | 0.7 |  |
| 80+ \* fair/poor | -0.54 | 0.394 | -1.6, 0.56 | 0.2 |  |
| Age \* Smoking |  |  |  |  | 0.463 |
| 30-39 yrs \* Occasional smoking | 0.04 | 0.357 | -0.95, 1.0 | >0.9 |  |
| 40-49 yrs \* Occasional smoking | 0.62 | 0.459 | -0.65, 1.9 | 0.2 |  |
| 50-59 yrs \* Occasional smoking | 0.40 | 0.416 | -0.75, 1.6 | 0.4 |  |
| 60-69 yrs \* Occasional smoking | 0.49 | 0.582 | -1.1, 2.1 | 0.4 |  |
| 70-79 yrs \* Occasional smoking | 0.66 | 0.747 | -1.4, 2.7 | 0.4 |  |
| 80+ \* Occasional smoking | -0.16 | 1.09 | -3.2, 2.9 | 0.9 |  |
| 30-39 yrs \* Non-smoker | 0.00 | 0.265 | -0.73, 0.74 | >0.9 |  |
| 40-49 yrs \* Non-smoker | 0.15 | 0.267 | -0.59, 0.89 | 0.6 |  |
| 50-59 yrs \* Non-smoker | 0.03 | 0.263 | -0.70, 0.76 | >0.9 |  |
| 60-69 yrs \* Non-smoker | -0.36 | 0.296 | -1.2, 0.47 | 0.3 |  |
| 70-79 yrs \* Non-smoker | -0.34 | 0.393 | -1.4, 0.75 | 0.4 |  |
| 80+ \* Non-smoker | 0.48 | 0.604 | -1.2, 2.2 | 0.5 |  |
| Age \* Sausage products |  |  |  |  | 0.529 |
| 30-39 yrs \* 4 to 6 times per week | 0.18 | 0.359 | -0.81, 1.2 | 0.6 |  |
| 40-49 yrs \* 4 to 6 times per week | 0.87 | 0.399 | -0.23, 2.0 | 0.093 |  |
| 50-59 yrs \* 4 to 6 times per week | 0.92 | 0.409 | -0.21, 2.1 | 0.088 |  |
| 60-69 yrs \* 4 to 6 times per week | 0.57 | 0.398 | -0.53, 1.7 | 0.2 |  |
| 70-79 yrs \* 4 to 6 times per week | 0.49 | 0.460 | -0.79, 1.8 | 0.3 |  |
| 80+ \* 4 to 6 times per week | 0.66 | 0.659 | -1.2, 2.5 | 0.4 |  |
| 30-39 yrs \* 1 to 3 times per week | -0.16 | 0.352 | -1.1, 0.81 | 0.7 |  |
| 40-49 yrs \* 1 to 3 times per week | 0.83 | 0.353 | -0.15, 1.8 | 0.078 |  |
| 50-59 yrs \* 1 to 3 times per week | 0.66 | 0.356 | -0.33, 1.6 | 0.14 |  |
| 60-69 yrs \* 1 to 3 times per week | 0.44 | 0.369 | -0.58, 1.5 | 0.3 |  |
| 70-79 yrs \* 1 to 3 times per week | 0.35 | 0.469 | -0.95, 1.7 | 0.5 |  |
| 80+ \* 1 to 3 times per week | -0.02 | 0.546 | -1.5, 1.5 | >0.9 |  |
| 30-39 yrs \* Less than once per week | 0.01 | 0.386 | -1.1, 1.1 | >0.9 |  |
| 40-49 yrs \* Less than once per week | 0.42 | 0.363 | -0.59, 1.4 | 0.3 |  |
| 50-59 yrs \* Less than once per week | 0.54 | 0.386 | -0.53, 1.6 | 0.2 |  |
| 60-69 yrs \* Less than once per week | 0.31 | 0.386 | -0.77, 1.4 | 0.5 |  |
| 70-79 yrs \* Less than once per week | 0.33 | 0.485 | -1.0, 1.7 | 0.5 |  |
| 80+ \* Less than once per week | 0.28 | 0.595 | -1.4, 1.9 | 0.7 |  |
| 30-39 yrs \* Never | -0.24 | 0.469 | -1.5, 1.1 | 0.6 |  |
| 40-49 yrs \* Never | 0.73 | 0.479 | -0.60, 2.1 | 0.2 |  |
| 50-59 yrs \* Never | 0.66 | 0.524 | -0.80, 2.1 | 0.3 |  |
| 60-69 yrs \* Never | 0.86 | 0.567 | -0.72, 2.4 | 0.2 |  |
| 70-79 yrs \* Never | 0.04 | 0.659 | -1.8, 1.9 | >0.9 |  |
| 80+ \* Never | 0.12 | 0.742 | -1.9, 2.2 | 0.9 |  |
| Age \* Red meat |  |  |  |  | 0.19 |
| 30-39 yrs \* 4 to 6 times per week | 0.24 | 0.513 | -1.2, 1.7 | 0.7 |  |
| 40-49 yrs \* 4 to 6 times per week | -0.36 | 0.500 | -1.7, 1.0 | 0.5 |  |
| 50-59 yrs \* 4 to 6 times per week | -1.2 | 0.708 | -3.1, 0.78 | 0.2 |  |
| 60-69 yrs \* 4 to 6 times per week | -0.61 | 0.636 | -2.4, 1.2 | 0.4 |  |
| 70-79 yrs \* 4 to 6 times per week | -1.1 | 1.13 | -4.2, 2.0 | 0.4 |  |
| 80+ \* 4 to 6 times per week | 1.2 | 1.15 | -2.0, 4.4 | 0.3 |  |
| 30-39 yrs \* 1 to 3 times per week | -0.13 | 0.486 | -1.5, 1.2 | 0.8 |  |
| 40-49 yrs \* 1 to 3 times per week | -0.73 | 0.475 | -2.0, 0.59 | 0.2 |  |
| 50-59 yrs \* 1 to 3 times per week | -1.0 | 0.669 | -2.9, 0.81 | 0.2 |  |
| 60-69 yrs \* 1 to 3 times per week | -0.25 | 0.575 | -1.8, 1.3 | 0.7 |  |
| 70-79 yrs \* 1 to 3 times per week | -1.1 | 1.05 | -4.0, 1.8 | 0.3 |  |
| 80+ \* 1 to 3 times per week | 0.36 | 0.994 | -2.4, 3.1 | 0.7 |  |
| 30-39 yrs \* Less than once per week | 0.22 | 0.486 | -1.1, 1.6 | 0.7 |  |
| 40-49 yrs \* Less than once per week | -0.46 | 0.485 | -1.8, 0.88 | 0.4 |  |
| 50-59 yrs \* Less than once per week | -1.2 | 0.700 | -3.1, 0.77 | 0.2 |  |
| 60-69 yrs \* Less than once per week | -0.40 | 0.621 | -2.1, 1.3 | 0.6 |  |
| 70-79 yrs \* Less than once per week | -0.94 | 1.08 | -3.9, 2.1 | 0.4 |  |
| 80+ \* Less than once per week | 0.54 | 1.05 | -2.4, 3.4 | 0.6 |  |
| 30-39 yrs \* Never | 0.64 | 0.642 | -1.1, 2.4 | 0.4 |  |
| 40-49 yrs \* Never | -0.41 | 0.616 | -2.1, 1.3 | 0.5 |  |
| 50-59 yrs \* Never | -1.2 | 0.826 | -3.5, 1.1 | 0.2 |  |
| 60-69 yrs \* Never | -0.60 | 0.713 | -2.6, 1.4 | 0.4 |  |
| 70-79 yrs \* Never | -2.0 | 1.13 | -5.1, 1.2 | 0.2 |  |
| 80+ \* Never | 0.69 | 1.18 | -2.6, 4.0 | 0.6 |  |
| Age \* Sport |  |  |  |  | 0.457 |
| 30-39 yrs \* Less than 1 hour per week | 0.73 | 0.324 | -0.17, 1.6 | 0.089 |  |
| 40-49 yrs \* Less than 1 hour per week | 0.32 | 0.355 | -0.67, 1.3 | 0.4 |  |
| 50-59 yrs \* Less than 1 hour per week | 0.20 | 0.339 | -0.75, 1.1 | 0.6 |  |
| 60-69 yrs \* Less than 1 hour per week | 0.31 | 0.349 | -0.66, 1.3 | 0.4 |  |
| 70-79 yrs \* Less than 1 hour per week | 0.28 | 0.420 | -0.89, 1.4 | 0.5 |  |
| 80+ \* Less than 1 hour per week | 0.28 | 0.450 | -0.97, 1.5 | 0.6 |  |
| 30-39 yrs \* 1 to less than 2 hours per week | 0.60 | 0.310 | -0.26, 1.5 | 0.13 |  |
| 40-49 yrs \* 1 to less than 2 hours per week | 0.51 | 0.347 | -0.45, 1.5 | 0.2 |  |
| 50-59 yrs \* 1 to less than 2 hours per week | 0.47 | 0.308 | -0.39, 1.3 | 0.2 |  |
| 60-69 yrs \* 1 to less than 2 hours per week | 0.85 | 0.343 | -0.10, 1.8 | 0.069 |  |
| 70-79 yrs \* 1 to less than 2 hours per week | 0.39 | 0.391 | -0.70, 1.5 | 0.4 |  |
| 80+ \* 1 to less than 2 hours per week | 0.14 | 0.426 | -1.0, 1.3 | 0.8 |  |
| 30-39 yrs \* 2 to less than 4 hours per week | 0.45 | 0.304 | -0.39, 1.3 | 0.2 |  |
| 40-49 yrs \* 2 to less than 4 hours per week | 0.36 | 0.355 | -0.63, 1.3 | 0.4 |  |
| 50-59 yrs \* 2 to less than 4 hours per week | 0.29 | 0.340 | -0.66, 1.2 | 0.4 |  |
| 60-69 yrs \* 2 to less than 4 hours per week | 0.31 | 0.345 | -0.65, 1.3 | 0.4 |  |
| 70-79 yrs \* 2 to less than 4 hours per week | 0.02 | 0.389 | -1.1, 1.1 | >0.9 |  |
| 80+ \* 2 to less than 4 hours per week | 0.42 | 0.501 | -0.97, 1.8 | 0.4 |  |
| 30-39 yrs \* 4 hours per week and more | 0.48 | 0.338 | -0.46, 1.4 | 0.2 |  |
| 40-49 yrs \* 4 hours per week and more | 0.26 | 0.368 | -0.76, 1.3 | 0.5 |  |
| 50-59 yrs \* 4 hours per week and more | 0.58 | 0.370 | -0.45, 1.6 | 0.2 |  |
| 60-69 yrs \* 4 hours per week and more | 1.1 | 0.384 | 0.05, 2.2 | 0.044 |  |
| 70-79 yrs \* 4 hours per week and more | 0.63 | 0.462 | -0.66, 1.9 | 0.2 |  |
| 80+ \* 4 hours per week and more | 0.97 | 0.765 | -1.2, 3.1 | 0.3 |  |
| Age \* BIK community size (categorized) |  |  |  |  | 0.077 |
| 30-39 yrs \* BIK region 20,000 to <50,000 inhabitants OR surroundings 50,000 to <500,000 inhabitants | -0.18 | 0.355 | -1.2, 0.81 | 0.6 |  |
| 40-49 yrs \* BIK region 20,000 to <50,000 inhabitants OR surroundings 50,000 to <500,000 inhabitants | -0.74 | 0.346 | -1.7, 0.22 | 0.10 |  |
| 50-59 yrs \* BIK region 20,000 to <50,000 inhabitants OR surroundings 50,000 to <500,000 inhabitants | -0.10 | 0.291 | -0.91, 0.70 | 0.7 |  |
| 60-69 yrs \* BIK region 20,000 to <50,000 inhabitants OR surroundings 50,000 to <500,000 inhabitants | -0.23 | 0.299 | -1.1, 0.60 | 0.5 |  |
| 70-79 yrs \* BIK region 20,000 to <50,000 inhabitants OR surroundings 50,000 to <500,000 inhabitants | -0.61 | 0.455 | -1.9, 0.65 | 0.3 |  |
| 80+ \* BIK region 20,000 to <50,000 inhabitants OR surroundings 50,000 to <500,000 inhabitants | -0.38 | 0.616 | -2.1, 1.3 | 0.6 |  |
| 30-39 yrs \* Core city 50,000 to <500,000 inhabitants OR surroundings 500,000+ inhabitants | -0.36 | 0.356 | -1.3, 0.63 | 0.4 |  |
| 40-49 yrs \* Core city 50,000 to <500,000 inhabitants OR surroundings 500,000+ inhabitants | -0.72 | 0.368 | -1.7, 0.30 | 0.12 |  |
| 50-59 yrs \* Core city 50,000 to <500,000 inhabitants OR surroundings 500,000+ inhabitants | 0.19 | 0.311 | -0.67, 1.1 | 0.6 |  |
| 60-69 yrs \* Core city 50,000 to <500,000 inhabitants OR surroundings 500,000+ inhabitants | -0.19 | 0.348 | -1.2, 0.77 | 0.6 |  |
| 70-79 yrs \* Core city 50,000 to <500,000 inhabitants OR surroundings 500,000+ inhabitants | -0.84 | 0.491 | -2.2, 0.52 | 0.2 |  |
| 80+ \* Core city 50,000 to <500,000 inhabitants OR surroundings 500,000+ inhabitants | 0.05 | 0.625 | -1.7, 1.8 | >0.9 |  |
| 30-39 yrs \* Core city 500,000+ inhabitants | -0.56 | 0.359 | -1.6, 0.44 | 0.2 |  |
| 40-49 yrs \* Core city 500,000+ inhabitants | -0.52 | 0.357 | -1.5, 0.47 | 0.2 |  |
| 50-59 yrs \* Core city 500,000+ inhabitants | -0.08 | 0.310 | -0.94, 0.78 | 0.8 |  |
| 60-69 yrs \* Core city 500,000+ inhabitants | -0.16 | 0.308 | -1.0, 0.70 | 0.6 |  |
| 70-79 yrs \* Core city 500,000+ inhabitants | -1.1 | 0.492 | -2.5, 0.28 | 0.092 |  |
| 80+ \* Core city 500,000+ inhabitants | -0.75 | 0.622 | -2.5, 0.98 | 0.3 |  |
| Age \* Regions |  |  |  |  | 0.61 |
| 30-39 yrs \* Northwest | -0.07 | 0.302 | -0.91, 0.77 | 0.8 |  |
| 40-49 yrs \* Northwest | 0.15 | 0.320 | -0.74, 1.0 | 0.7 |  |
| 50-59 yrs \* Northwest | -0.06 | 0.328 | -0.98, 0.85 | 0.9 |  |
| 60-69 yrs \* Northwest | -0.14 | 0.353 | -1.1, 0.84 | 0.7 |  |
| 70-79 yrs \* Northwest | -0.49 | 0.436 | -1.7, 0.72 | 0.3 |  |
| 80+ \* Northwest | 0.23 | 0.527 | -1.2, 1.7 | 0.7 |  |
| 30-39 yrs \* Central-East | -0.69 | 0.335 | -1.6, 0.24 | 0.11 |  |
| 40-49 yrs \* Central-East | -0.49 | 0.383 | -1.6, 0.57 | 0.3 |  |
| 50-59 yrs \* Central-East | -0.29 | 0.419 | -1.5, 0.87 | 0.5 |  |
| 60-69 yrs \* Central-East | 0.15 | 0.392 | -0.94, 1.2 | 0.7 |  |
| 70-79 yrs \* Central-East | -0.45 | 0.499 | -1.8, 0.94 | 0.4 |  |
| 80+ \* Central-East | -0.40 | 0.615 | -2.1, 1.3 | 0.6 |  |
| 30-39 yrs \* Central-West | -0.38 | 0.282 | -1.2, 0.41 | 0.3 |  |
| 40-49 yrs \* Central-West | -0.06 | 0.301 | -0.90, 0.77 | 0.8 |  |
| 50-59 yrs \* Central-West | -0.22 | 0.312 | -1.1, 0.65 | 0.5 |  |
| 60-69 yrs \* Central-West | -0.03 | 0.321 | -0.92, 0.87 | >0.9 |  |
| 70-79 yrs \* Central-West | -0.29 | 0.426 | -1.5, 0.89 | 0.5 |  |
| 80+ \* Central-West | -0.38 | 0.497 | -1.8, 1.0 | 0.5 |  |
| 30-39 yrs \* South | -0.10 | 0.306 | -0.95, 0.75 | 0.8 |  |
| 40-49 yrs \* South | -0.01 | 0.303 | -0.85, 0.83 | >0.9 |  |
| 50-59 yrs \* South | -0.02 | 0.317 | -0.90, 0.85 | >0.9 |  |
| 60-69 yrs \* South | -0.15 | 0.321 | -1.0, 0.74 | 0.7 |  |
| 70-79 yrs \* South | -0.52 | 0.419 | -1.7, 0.64 | 0.3 |  |
| 80+ \* South | -0.52 | 0.510 | -1.9, 0.90 | 0.4 |  |
| Age \* Country of birth |  |  |  |  | 0.237 |
| 30-39 yrs \* In another country | 0.53 | 0.415 | -0.62, 1.7 | 0.3 |  |
| 40-49 yrs \* In another country | -0.30 | 0.358 | -1.3, 0.69 | 0.4 |  |
| 50-59 yrs \* In another country | -0.16 | 0.387 | -1.2, 0.91 | 0.7 |  |
| 60-69 yrs \* In another country | -0.20 | 0.404 | -1.3, 0.92 | 0.6 |  |
| 70-79 yrs \* In another country | 0.48 | 0.526 | -0.98, 1.9 | 0.4 |  |
| 80+ \* In another country | -0.01 | 0.440 | -1.2, 1.2 | >0.9 |  |
| Age \* German nationality |  |  |  |  | 0.39 |
| 30-39 yrs \* No | -0.24 | 0.465 | -1.5, 1.1 | 0.6 |  |
| 40-49 yrs \* No | 0.14 | 0.414 | -1.0, 1.3 | 0.7 |  |
| 50-59 yrs \* No | -0.30 | 0.390 | -1.4, 0.78 | 0.5 |  |
| 60-69 yrs \* No | -1.1 | 0.574 | -2.7, 0.51 | 0.13 |  |
| 70-79 yrs \* No | -0.13 | 0.761 | -2.2, 2.0 | 0.9 |  |
| 80+ \* No | 0.39 | 1.12 | -2.7, 3.5 | 0.7 |  |
| Age \* Household size |  |  |  |  | 0.505 |
| 30-39 yrs \* Multi-person household | 0.08 | 0.254 | -0.62, 0.79 | 0.8 |  |
| 40-49 yrs \* Multi-person household | 0.12 | 0.248 | -0.57, 0.81 | 0.7 |  |
| 50-59 yrs \* Multi-person household | 0.12 | 0.255 | -0.59, 0.83 | 0.7 |  |
| 60-69 yrs \* Multi-person household | 0.42 | 0.221 | -0.19, 1.0 | 0.13 |  |
| 70-79 yrs \* Multi-person household | -0.09 | 0.295 | -0.91, 0.73 | 0.8 |  |
| 80+ \* Multi-person household | 0.29 | 0.307 | -0.56, 1.1 | 0.4 |  |
| Age \* Education: ISCED (2011) |  |  |  |  | 0.649 |
| 30-39 yrs \* Medium | -0.43 | 0.355 | -1.4, 0.56 | 0.3 |  |
| 40-49 yrs \* Medium | -0.28 | 0.338 | -1.2, 0.66 | 0.5 |  |
| 50-59 yrs \* Medium | 0.08 | 0.361 | -0.93, 1.1 | 0.8 |  |
| 60-69 yrs \* Medium | -0.05 | 0.377 | -1.1, 0.99 | 0.9 |  |
| 70-79 yrs \* Medium | 0.19 | 0.344 | -0.76, 1.1 | 0.6 |  |
| 80+ \* Medium | 0.09 | 0.376 | -0.95, 1.1 | 0.8 |  |
| 30-39 yrs \* High | -0.66 | 0.377 | -1.7, 0.39 | 0.2 |  |
| 40-49 yrs \* High | -0.73 | 0.353 | -1.7, 0.25 | 0.11 |  |
| 50-59 yrs \* High | -0.14 | 0.369 | -1.2, 0.89 | 0.7 |  |
| 60-69 yrs \* High | -0.33 | 0.402 | -1.4, 0.79 | 0.5 |  |
| 70-79 yrs \* High | -0.07 | 0.407 | -1.2, 1.1 | 0.9 |  |
| 80+ \* High | 0.03 | 0.452 | -1.2, 1.3 | >0.9 |  |
| Sex \* Chronic diseases |  |  |  |  | 0.051 |
| Female \* No | -0.22 | 0.115 | -0.54, 0.09 | 0.12 |  |
| Sex \* Sausage products |  |  |  |  | 0.007 |
| Female \* 4 to 6 times per week | 0.07 | 0.261 | -0.66, 0.79 | 0.8 |  |
| Female \* 1 to 3 times per week | -0.48 | 0.231 | -1.1, 0.16 | 0.11 |  |
| Female \* Less than once per week | -0.14 | 0.240 | -0.81, 0.53 | 0.6 |  |
| Female \* Never | -0.06 | 0.277 | -0.83, 0.71 | 0.8 |  |
| Sex \* Sport |  |  |  |  | 0.534 |
| Female \* Less than 1 hour per week | 0.01 | 0.184 | -0.50, 0.52 | >0.9 |  |
| Female \* 1 to less than 2 hours per week | 0.22 | 0.180 | -0.28, 0.72 | 0.3 |  |
| Female \* 2 to less than 4 hours per week | 0.25 | 0.203 | -0.32, 0.81 | 0.3 |  |
| Female \* 4 hours per week and more | 0.08 | 0.203 | -0.48, 0.65 | 0.7 |  |
| Sex \* BIK community size (categorized) |  |  |  |  | 0.273 |
| Female \* BIK region 20,000 to <50,000 inhabitants OR surroundings 50,000 to <500,000 inhabitants | 0.03 | 0.236 | -0.62, 0.69 | 0.9 |  |
| Female \* Core city 50,000 to <500,000 inhabitants OR surroundings 500,000+ inhabitants | -0.25 | 0.236 | -0.91, 0.40 | 0.3 |  |
| Female \* Core city 500,000+ inhabitants | -0.15 | 0.232 | -0.79, 0.49 | 0.5 |  |
| Sex \* Regions |  |  |  |  | 0.131 |
| Female \* Northwest | 0.06 | 0.219 | -0.55, 0.67 | 0.8 |  |
| Female \* Central-East | -0.21 | 0.244 | -0.88, 0.47 | 0.4 |  |
| Female \* Central-West | -0.35 | 0.195 | -0.89, 0.19 | 0.15 |  |
| Female \* South | -0.11 | 0.199 | -0.67, 0.44 | 0.6 |  |
| Sex \* German nationality |  |  |  |  | 0.067 |
| Female \* No | -0.39 | 0.215 | -0.99, 0.20 | 0.14 |  |
| Sex \* Education: ISCED (2011) |  |  |  |  | 0.004 |
| Female \* Medium | 0.06 | 0.199 | -0.49, 0.62 | 0.8 |  |
| Female \* High | -0.37 | 0.209 | -0.95, 0.21 | 0.2 |  |
| Obesity \* Education: CASMIN |  |  |  |  | 0.883 |
| No \* Medium | -0.10 | 0.202 | -0.66, 0.46 | 0.6 |  |
| No \* High | -0.05 | 0.222 | -0.67, 0.56 | 0.8 |  |
| Self-rated health \* Education: CASMIN |  |  |  |  | 0.161 |
| Bad/very bad \* Medium | -0.49 | 0.324 | -1.4, 0.41 | 0.2 |  |
| Bad/very bad \* High | -0.62 | 0.362 | -1.6, 0.38 | 0.2 |  |
| Satisfaction: Life in general \* Education: CASMIN |  |  |  |  | 0.268 |
| Scale value 4 to 7 \* Medium | -0.64 | 0.331 | -1.6, 0.28 | 0.12 |  |
| Scale value 8 to 10 \* Medium | -0.47 | 0.352 | -1.5, 0.50 | 0.2 |  |
| Scale value 4 to 7 \* High | -0.33 | 0.364 | -1.3, 0.68 | 0.4 |  |
| Scale value 8 to 10 \* High | -0.10 | 0.376 | -1.1, 0.95 | 0.8 |  |
| Health risk due to climate change \* Education: CASMIN |  |  |  |  | 0.182 |
| Scale value 4 to 7 \* Medium | 0.37 | 0.180 | -0.13, 0.87 | 0.11 |  |
| Scale value 8 to 10 \* Medium | 0.52 | 0.256 | -0.19, 1.2 | 0.11 |  |
| Scale value 4 to 7 \* High | 0.36 | 0.203 | -0.20, 0.93 | 0.15 |  |
| Scale value 8 to 10 \* High | 0.40 | 0.253 | -0.30, 1.1 | 0.2 |  |
| Education: CASMIN \* Paying attention to health |  |  |  |  | 0.229 |
| Medium \* Strong/very strong | 0.19 | 0.150 | -0.23, 0.61 | 0.3 |  |
| High \* Strong/very strong | -0.03 | 0.161 | -0.47, 0.42 | 0.9 |  |
| Self-rated mental health \* Education: CASMIN |  |  |  |  | 0.649 |
| fair/poor \* Medium | 0.02 | 0.212 | -0.57, 0.61 | >0.9 |  |
| fair/poor \* High | 0.19 | 0.235 | -0.47, 0.84 | 0.5 |  |
| Smoking \* Education: CASMIN |  |  |  |  | 0.403 |
| Occasional smoking \* Medium | -0.49 | 0.355 | -1.5, 0.49 | 0.2 |  |
| Non-smoker \* Medium | -0.23 | 0.181 | -0.73, 0.28 | 0.3 |  |
| Occasional smoking \* High | -0.59 | 0.413 | -1.7, 0.56 | 0.2 |  |
| Non-smoker \* High | -0.41 | 0.251 | -1.1, 0.29 | 0.2 |  |
| Sausage products \* Education: CASMIN |  |  |  |  | 0.518 |
| 4 to 6 times per week \* Medium | -0.36 | 0.318 | -1.2, 0.52 | 0.3 |  |
| 1 to 3 times per week \* Medium | 0.07 | 0.293 | -0.74, 0.88 | 0.8 |  |
| Less than once per week \* Medium | -0.03 | 0.297 | -0.85, 0.80 | >0.9 |  |
| Never \* Medium | 0.31 | 0.395 | -0.79, 1.4 | 0.5 |  |
| 4 to 6 times per week \* High | -0.35 | 0.346 | -1.3, 0.61 | 0.4 |  |
| 1 to 3 times per week \* High | 0.02 | 0.321 | -0.87, 0.91 | >0.9 |  |
| Less than once per week \* High | 0.12 | 0.334 | -0.81, 1.0 | 0.7 |  |
| Never \* High | 0.11 | 0.410 | -1.0, 1.3 | 0.8 |  |
| Red meat \* Education: CASMIN |  |  |  |  | 0.821 |
| 4 to 6 times per week \* Medium | -0.24 | 0.471 | -1.5, 1.1 | 0.6 |  |
| 1 to 3 times per week \* Medium | -0.35 | 0.437 | -1.6, 0.86 | 0.5 |  |
| Less than once per week \* Medium | -0.49 | 0.445 | -1.7, 0.75 | 0.3 |  |
| Never \* Medium | -0.46 | 0.576 | -2.1, 1.1 | 0.5 |  |
| 4 to 6 times per week \* High | -0.09 | 0.528 | -1.6, 1.4 | 0.9 |  |
| 1 to 3 times per week \* High | -0.06 | 0.512 | -1.5, 1.4 | >0.9 |  |
| Less than once per week \* High | -0.39 | 0.511 | -1.8, 1.0 | 0.5 |  |
| Never \* High | -0.17 | 0.626 | -1.9, 1.6 | 0.8 |  |
| Sport \* Education: CASMIN |  |  |  |  | 0.419 |
| Less than 1 hour per week \* Medium | -0.32 | 0.224 | -0.95, 0.30 | 0.2 |  |
| 1 to less than 2 hours per week \* Medium | -0.48 | 0.223 | -1.1, 0.14 | 0.10 |  |
| 2 to less than 4 hours per week \* Medium | -0.24 | 0.260 | -0.97, 0.48 | 0.4 |  |
| 4 hours per week and more \* Medium | -0.64 | 0.310 | -1.5, 0.22 | 0.11 |  |
| Less than 1 hour per week \* High | -0.31 | 0.275 | -1.1, 0.46 | 0.3 |  |
| 1 to less than 2 hours per week \* High | -0.25 | 0.272 | -1.0, 0.51 | 0.4 |  |
| 2 to less than 4 hours per week \* High | -0.23 | 0.290 | -1.0, 0.57 | 0.5 |  |
| 4 hours per week and more \* High | -0.57 | 0.322 | -1.5, 0.33 | 0.2 |  |
| Education: CASMIN \* Waited for medical examination date in the last 12 months |  |  |  |  | 0.249 |
| Medium \* No | 0.03 | 0.158 | -0.40, 0.47 | 0.8 |  |
| High \* No | -0.13 | 0.180 | -0.63, 0.37 | 0.5 |  |
| Medium \* No need for examination or treatment | -0.37 | 0.269 | -1.1, 0.37 | 0.2 |  |
| High \* No need for examination or treatment | -0.64 | 0.299 | -1.5, 0.19 | 0.10 |  |
| Education: CASMIN \* Interview type |  |  |  |  | 0.535 |
| Medium \* Paper and Pencil Interview | -0.01 | 0.180 | -0.51, 0.49 | >0.9 |  |
| High \* Paper and Pencil Interview | -0.23 | 0.228 | -0.86, 0.40 | 0.4 |  |
| BIK community size (categorized) \* Education: CASMIN |  |  |  |  | 0.011 |
| BIK region 20,000 to <50,000 inhabitants OR surroundings 50,000 to <500,000 inhabitants \* Medium | 0.13 | 0.240 | -0.53, 0.80 | 0.6 |  |
| Core city 50,000 to <500,000 inhabitants OR surroundings 500,000+ inhabitants \* Medium | 0.14 | 0.249 | -0.55, 0.84 | 0.6 |  |
| Core city 500,000+ inhabitants \* Medium | 0.01 | 0.264 | -0.73, 0.74 | >0.9 |  |
| BIK region 20,000 to <50,000 inhabitants OR surroundings 50,000 to <500,000 inhabitants \* High | 0.87 | 0.256 | 0.16, 1.6 | 0.028 |  |
| Core city 50,000 to <500,000 inhabitants OR surroundings 500,000+ inhabitants \* High | 0.83 | 0.271 | 0.08, 1.6 | 0.037 |  |
| Core city 500,000+ inhabitants \* High | 0.65 | 0.278 | -0.13, 1.4 | 0.081 |  |
| Regions \* Education: CASMIN |  |  |  |  | 0.49 |
| Northwest \* Medium | -0.34 | 0.288 | -1.1, 0.46 | 0.3 |  |
| Central-East \* Medium | 0.23 | 0.346 | -0.73, 1.2 | 0.5 |  |
| Central-West \* Medium | 0.02 | 0.270 | -0.73, 0.77 | >0.9 |  |
| South \* Medium | 0.05 | 0.274 | -0.71, 0.81 | 0.9 |  |
| Northwest \* High | -0.44 | 0.335 | -1.4, 0.49 | 0.3 |  |
| Central-East \* High | 0.36 | 0.379 | -0.70, 1.4 | 0.4 |  |
| Central-West \* High | -0.05 | 0.322 | -0.95, 0.84 | 0.9 |  |
| South \* High | -0.11 | 0.323 | -1.0, 0.79 | 0.8 |  |
| Current living situation \* Education: CASMIN |  |  |  |  | 0.587 |
| Part-time employed \* Medium | -0.02 | 0.235 | -0.67, 0.63 | >0.9 |  |
| Unemployed \* Medium | -0.19 | 0.336 | -1.1, 0.75 | 0.6 |  |
| Retired or early retired \* Medium | 0.28 | 0.230 | -0.35, 0.92 | 0.3 |  |
| Not employed for other reasons (student, volunteer service, homemaker) \* Medium | 0.55 | 0.291 | -0.26, 1.4 | 0.13 |  |
| Part-time employed \* High | -0.01 | 0.252 | -0.72, 0.69 | >0.9 |  |
| Unemployed \* High | -0.23 | 0.430 | -1.4, 0.96 | 0.6 |  |
| Retired or early retired \* High | 0.25 | 0.291 | -0.56, 1.1 | 0.4 |  |
| Not employed for other reasons (student, volunteer service, homemaker) \* High | 0.48 | 0.312 | -0.39, 1.3 | 0.2 |  |
| N.Obs | 11,845 |  |  |  |  |
| N.Cluster | 359 |  |  |  |  |
|  |  |  |  |  |  |
| --- | --- | --- | --- | --- | --- |
| Abbreviations: CI = Confidence Interval, OR = Odds Ratio, SE = Standard Error | | | | | |

### Quarter 4

| Characteristic | log(OR) | SE | 95% CI | p-value | p-value (global) |
| --- | --- | --- | --- | --- | --- |
| Sex |  |  |  |  | 0.007 |
| Male | — | — | — |  |  |
| Female | 0.17 | 0.065 | 0.05, 0.30 | 0.008 |  |
| Age |  |  |  |  | 0.001 |
| 16-29 yrs | — | — | — |  |  |
| 30-39 yrs | 0.21 | 0.431 | -0.64, 1.1 | 0.6 |  |
| 40-49 yrs | 0.40 | 0.474 | -0.54, 1.3 | 0.4 |  |
| 50-59 yrs | 0.68 | 0.431 | -0.17, 1.5 | 0.12 |  |
| 60-69 yrs | 1.6 | 0.513 | 0.61, 2.6 | 0.002 |  |
| 70-79 yrs | 2.1 | 0.699 | 0.69, 3.5 | 0.003 |  |
| 80+ | -1.8 | 1.00 | -3.8, 0.20 | 0.079 |  |
| Smoking |  |  |  |  | 0.042 |
| Daily smoking | — | — | — |  |  |
| Occasional smoking | 0.76 | 0.352 | 0.07, 1.5 | 0.031 |  |
| Non-smoker | 0.56 | 0.243 | 0.08, 1.0 | 0.023 |  |
| Sausage products |  |  |  |  | 0.07 |
| Daily or several times a day | — | — | — |  |  |
| 4 to 6 times per week | 0.39 | 0.252 | -0.10, 0.89 | 0.12 |  |
| 1 to 3 times per week | 0.13 | 0.226 | -0.31, 0.58 | 0.6 |  |
| Less than once per week | 0.23 | 0.235 | -0.24, 0.69 | 0.3 |  |
| Never | 0.57 | 0.244 | 0.09, 1.1 | 0.021 |  |
| Country of birth |  |  |  |  | 0.084 |
| In Germany (within current borders) | — | — | — |  |  |
| In another country | -0.44 | 0.254 | -0.94, 0.06 | 0.085 |  |
| German nationality |  |  |  |  | <0.001 |
| Yes | — | — | — |  |  |
| No | -0.55 | 0.161 | -0.86, -0.23 | <0.001 |  |
| Obesity |  |  |  |  | >0.9 |
| Yes | — | — | — |  |  |
| No | 0.02 | 0.261 | -0.50, 0.54 | >0.9 |  |
| Self-rated health |  |  |  |  | 0.289 |
| Very good/good/fair | — | — | — |  |  |
| Bad/very bad | -0.55 | 0.515 | -1.6, 0.47 | 0.3 |  |
| Sport |  |  |  |  | >0.9 |
| No sporting activities | — | — | — |  |  |
| Less than 1 hour per week | -0.09 | 0.214 | -0.52, 0.33 | 0.7 |  |
| 1 to less than 2 hours per week | -0.01 | 0.218 | -0.44, 0.42 | >0.9 |  |
| 2 to less than 4 hours per week | 0.02 | 0.209 | -0.40, 0.43 | >0.9 |  |
| 4 hours per week and more | 0.00 | 0.204 | -0.40, 0.40 | >0.9 |  |
| Interview type |  |  |  |  | 0.106 |
| Computer Assisted Web Interview | — | — | — |  |  |
| Paper and Pencil Interview | -0.42 | 0.258 | -0.93, 0.09 | 0.11 |  |
| Regions |  |  |  |  | 0.449 |
| Northeast | — | — | — |  |  |
| Northwest | 0.03 | 0.204 | -0.37, 0.43 | 0.9 |  |
| Central-East | 0.23 | 0.252 | -0.27, 0.72 | 0.4 |  |
| Central-West | -0.12 | 0.194 | -0.50, 0.26 | 0.5 |  |
| South | 0.15 | 0.211 | -0.27, 0.56 | 0.5 |  |
| Education: CASMIN |  |  |  |  | 0.692 |
| Low | — | — | — |  |  |
| Medium | 0.14 | 0.271 | -0.40, 0.67 | 0.6 |  |
| High | -0.11 | 0.365 | -0.83, 0.61 | 0.8 |  |
| Overweight |  |  |  |  | >0.9 |
| Yes | — | — | — |  |  |
| No | 0.01 | 0.135 | -0.26, 0.27 | >0.9 |  |
| Waited for medical examination date in the last 12 months |  |  |  |  | 0.59 |
| Yes | — | — | — |  |  |
| No | 0.08 | 0.142 | -0.20, 0.36 | 0.6 |  |
| No need for examination or treatment | -0.13 | 0.214 | -0.55, 0.29 | 0.6 |  |
| BIK community size (categorized) |  |  |  |  | 0.232 |
| BIK region <20,000 inhabitants | — | — | — |  |  |
| BIK region 20,000 to <50,000 inhabitants OR surroundings 50,000 to <500,000 inhabitants | -0.32 | 0.168 | -0.65, 0.01 | 0.057 |  |
| Core city 50,000 to <500,000 inhabitants OR surroundings 500,000+ inhabitants | -0.22 | 0.183 | -0.58, 0.14 | 0.2 |  |
| Core city 500,000+ inhabitants | -0.13 | 0.195 | -0.51, 0.26 | 0.5 |  |
| Current living situation |  |  |  |  | 0.032 |
| Full-time employed | — | — | — |  |  |
| Part-time employed | -0.31 | 0.217 | -0.74, 0.12 | 0.2 |  |
| Unemployed | -0.17 | 0.273 | -0.71, 0.37 | 0.5 |  |
| Retired or early retired | 0.30 | 0.195 | -0.08, 0.69 | 0.12 |  |
| Not employed for other reasons (student, volunteer service, homemaker) | -0.26 | 0.231 | -0.71, 0.20 | 0.3 |  |
| Age \* Obesity |  |  |  |  | 0.788 |
| 30-39 yrs \* No | -0.01 | 0.243 | -0.49, 0.47 | >0.9 |  |
| 40-49 yrs \* No | 0.09 | 0.274 | -0.46, 0.63 | 0.8 |  |
| 50-59 yrs \* No | -0.16 | 0.257 | -0.67, 0.35 | 0.5 |  |
| 60-69 yrs \* No | -0.26 | 0.290 | -0.83, 0.32 | 0.4 |  |
| 70-79 yrs \* No | -0.13 | 0.332 | -0.78, 0.53 | 0.7 |  |
| 80+ \* No | 0.27 | 0.389 | -0.50, 1.0 | 0.5 |  |
| Age \* Self-rated health |  |  |  |  | 0.026 |
| 30-39 yrs \* Bad/very bad | 0.76 | 0.641 | -0.50, 2.0 | 0.2 |  |
| 40-49 yrs \* Bad/very bad | 1.2 | 0.696 | -0.13, 2.6 | 0.075 |  |
| 50-59 yrs \* Bad/very bad | 0.68 | 0.555 | -0.42, 1.8 | 0.2 |  |
| 60-69 yrs \* Bad/very bad | -0.33 | 0.561 | -1.4, 0.78 | 0.6 |  |
| 70-79 yrs \* Bad/very bad | 0.35 | 0.626 | -0.89, 1.6 | 0.6 |  |
| 80+ \* Bad/very bad | 0.16 | 0.595 | -1.0, 1.3 | 0.8 |  |
| Age \* Smoking |  |  |  |  | 0.058 |
| 30-39 yrs \* Occasional smoking | 0.43 | 0.370 | -0.30, 1.2 | 0.2 |  |
| 40-49 yrs \* Occasional smoking | 0.27 | 0.439 | -0.60, 1.1 | 0.5 |  |
| 50-59 yrs \* Occasional smoking | -0.21 | 0.407 | -1.0, 0.59 | 0.6 |  |
| 60-69 yrs \* Occasional smoking | -0.34 | 0.458 | -1.2, 0.57 | 0.5 |  |
| 70-79 yrs \* Occasional smoking | 1.7 | 1.19 | -0.67, 4.0 | 0.2 |  |
| 80+ \* Occasional smoking | 0.62 | 0.923 | -1.2, 2.4 | 0.5 |  |
| 30-39 yrs \* Non-smoker | 0.03 | 0.246 | -0.45, 0.52 | 0.9 |  |
| 40-49 yrs \* Non-smoker | 0.05 | 0.282 | -0.51, 0.60 | 0.9 |  |
| 50-59 yrs \* Non-smoker | -0.41 | 0.253 | -0.91, 0.09 | 0.11 |  |
| 60-69 yrs \* Non-smoker | -0.01 | 0.258 | -0.52, 0.50 | >0.9 |  |
| 70-79 yrs \* Non-smoker | -0.60 | 0.405 | -1.4, 0.20 | 0.14 |  |
| 80+ \* Non-smoker | 1.3 | 0.750 | -0.14, 2.8 | 0.075 |  |
| Age \* Sausage products |  |  |  |  | 0.006 |
| 30-39 yrs \* 4 to 6 times per week | -0.50 | 0.376 | -1.2, 0.24 | 0.2 |  |
| 40-49 yrs \* 4 to 6 times per week | 0.13 | 0.350 | -0.56, 0.82 | 0.7 |  |
| 50-59 yrs \* 4 to 6 times per week | 0.43 | 0.333 | -0.23, 1.1 | 0.2 |  |
| 60-69 yrs \* 4 to 6 times per week | -0.07 | 0.377 | -0.82, 0.67 | 0.8 |  |
| 70-79 yrs \* 4 to 6 times per week | -0.93 | 0.449 | -1.8, -0.04 | 0.041 |  |
| 80+ \* 4 to 6 times per week | 0.62 | 0.494 | -0.36, 1.6 | 0.2 |  |
| 30-39 yrs \* 1 to 3 times per week | -0.02 | 0.332 | -0.67, 0.64 | >0.9 |  |
| 40-49 yrs \* 1 to 3 times per week | 0.06 | 0.320 | -0.57, 0.69 | 0.9 |  |
| 50-59 yrs \* 1 to 3 times per week | 0.33 | 0.315 | -0.30, 0.95 | 0.3 |  |
| 60-69 yrs \* 1 to 3 times per week | 0.15 | 0.337 | -0.52, 0.81 | 0.7 |  |
| 70-79 yrs \* 1 to 3 times per week | -0.68 | 0.425 | -1.5, 0.16 | 0.11 |  |
| 80+ \* 1 to 3 times per week | -0.16 | 0.419 | -0.98, 0.67 | 0.7 |  |
| 30-39 yrs \* Less than once per week | -0.27 | 0.351 | -0.96, 0.43 | 0.5 |  |
| 40-49 yrs \* Less than once per week | 0.01 | 0.349 | -0.68, 0.70 | >0.9 |  |
| 50-59 yrs \* Less than once per week | 0.59 | 0.320 | -0.04, 1.2 | 0.067 |  |
| 60-69 yrs \* Less than once per week | 0.16 | 0.375 | -0.58, 0.90 | 0.7 |  |
| 70-79 yrs \* Less than once per week | -0.86 | 0.456 | -1.8, 0.04 | 0.062 |  |
| 80+ \* Less than once per week | 0.34 | 0.479 | -0.61, 1.3 | 0.5 |  |
| 30-39 yrs \* Never | -0.73 | 0.393 | -1.5, 0.04 | 0.064 |  |
| 40-49 yrs \* Never | -0.88 | 0.414 | -1.7, -0.06 | 0.036 |  |
| 50-59 yrs \* Never | 0.08 | 0.383 | -0.68, 0.83 | 0.8 |  |
| 60-69 yrs \* Never | -0.27 | 0.438 | -1.1, 0.59 | 0.5 |  |
| 70-79 yrs \* Never | -1.0 | 0.571 | -2.2, 0.08 | 0.068 |  |
| 80+ \* Never | 0.23 | 0.680 | -1.1, 1.6 | 0.7 |  |
| Age \* Sport |  |  |  |  | 0.275 |
| 30-39 yrs \* Less than 1 hour per week | 0.33 | 0.283 | -0.23, 0.89 | 0.2 |  |
| 40-49 yrs \* Less than 1 hour per week | 0.02 | 0.298 | -0.57, 0.60 | >0.9 |  |
| 50-59 yrs \* Less than 1 hour per week | 0.41 | 0.286 | -0.16, 0.97 | 0.2 |  |
| 60-69 yrs \* Less than 1 hour per week | -0.01 | 0.330 | -0.66, 0.64 | >0.9 |  |
| 70-79 yrs \* Less than 1 hour per week | -0.11 | 0.336 | -0.77, 0.55 | 0.7 |  |
| 80+ \* Less than 1 hour per week | 0.27 | 0.408 | -0.53, 1.1 | 0.5 |  |
| 30-39 yrs \* 1 to less than 2 hours per week | 0.09 | 0.282 | -0.47, 0.65 | 0.7 |  |
| 40-49 yrs \* 1 to less than 2 hours per week | 0.19 | 0.289 | -0.38, 0.76 | 0.5 |  |
| 50-59 yrs \* 1 to less than 2 hours per week | 0.31 | 0.283 | -0.25, 0.87 | 0.3 |  |
| 60-69 yrs \* 1 to less than 2 hours per week | 0.24 | 0.330 | -0.41, 0.89 | 0.5 |  |
| 70-79 yrs \* 1 to less than 2 hours per week | 0.06 | 0.343 | -0.62, 0.73 | 0.9 |  |
| 80+ \* 1 to less than 2 hours per week | 0.42 | 0.404 | -0.38, 1.2 | 0.3 |  |
| 30-39 yrs \* 2 to less than 4 hours per week | 0.44 | 0.314 | -0.18, 1.1 | 0.2 |  |
| 40-49 yrs \* 2 to less than 4 hours per week | -0.03 | 0.303 | -0.63, 0.57 | >0.9 |  |
| 50-59 yrs \* 2 to less than 4 hours per week | 0.02 | 0.292 | -0.56, 0.60 | >0.9 |  |
| 60-69 yrs \* 2 to less than 4 hours per week | 0.03 | 0.329 | -0.62, 0.68 | >0.9 |  |
| 70-79 yrs \* 2 to less than 4 hours per week | 0.83 | 0.426 | -0.01, 1.7 | 0.052 |  |
| 80+ \* 2 to less than 4 hours per week | 0.31 | 0.460 | -0.60, 1.2 | 0.5 |  |
| 30-39 yrs \* 4 hours per week and more | -0.11 | 0.288 | -0.68, 0.46 | 0.7 |  |
| 40-49 yrs \* 4 hours per week and more | 0.14 | 0.348 | -0.55, 0.82 | 0.7 |  |
| 50-59 yrs \* 4 hours per week and more | 0.35 | 0.347 | -0.34, 1.0 | 0.3 |  |
| 60-69 yrs \* 4 hours per week and more | -0.17 | 0.369 | -0.89, 0.56 | 0.7 |  |
| 70-79 yrs \* 4 hours per week and more | 0.94 | 0.434 | 0.09, 1.8 | 0.031 |  |
| 80+ \* 4 hours per week and more | 0.83 | 0.677 | -0.50, 2.2 | 0.2 |  |
| Age \* Interview type |  |  |  |  | 0.01 |
| 30-39 yrs \* Paper and Pencil Interview | 0.62 | 0.376 | -0.12, 1.4 | 0.10 |  |
| 40-49 yrs \* Paper and Pencil Interview | 1.0 | 0.384 | 0.29, 1.8 | 0.007 |  |
| 50-59 yrs \* Paper and Pencil Interview | 0.28 | 0.305 | -0.32, 0.88 | 0.4 |  |
| 60-69 yrs \* Paper and Pencil Interview | 0.31 | 0.326 | -0.34, 0.95 | 0.3 |  |
| 70-79 yrs \* Paper and Pencil Interview | 0.90 | 0.330 | 0.25, 1.6 | 0.007 |  |
| 80+ \* Paper and Pencil Interview | 1.1 | 0.425 | 0.24, 1.9 | 0.012 |  |
| Age \* Regions |  |  |  |  | 0.723 |
| 30-39 yrs \* Northwest | -0.01 | 0.264 | -0.53, 0.51 | >0.9 |  |
| 40-49 yrs \* Northwest | 0.03 | 0.321 | -0.61, 0.66 | >0.9 |  |
| 50-59 yrs \* Northwest | 0.30 | 0.287 | -0.27, 0.86 | 0.3 |  |
| 60-69 yrs \* Northwest | -0.22 | 0.329 | -0.87, 0.43 | 0.5 |  |
| 70-79 yrs \* Northwest | 0.29 | 0.399 | -0.50, 1.1 | 0.5 |  |
| 80+ \* Northwest | 0.13 | 0.499 | -0.85, 1.1 | 0.8 |  |
| 30-39 yrs \* Central-East | -0.05 | 0.325 | -0.69, 0.59 | 0.9 |  |
| 40-49 yrs \* Central-East | -0.08 | 0.373 | -0.81, 0.66 | 0.8 |  |
| 50-59 yrs \* Central-East | -0.16 | 0.406 | -0.96, 0.64 | 0.7 |  |
| 60-69 yrs \* Central-East | -0.36 | 0.429 | -1.2, 0.49 | 0.4 |  |
| 70-79 yrs \* Central-East | -0.67 | 0.487 | -1.6, 0.29 | 0.2 |  |
| 80+ \* Central-East | -0.32 | 0.535 | -1.4, 0.73 | 0.5 |  |
| 30-39 yrs \* Central-West | 0.31 | 0.250 | -0.18, 0.81 | 0.2 |  |
| 40-49 yrs \* Central-West | 0.32 | 0.307 | -0.29, 0.93 | 0.3 |  |
| 50-59 yrs \* Central-West | 0.33 | 0.273 | -0.21, 0.87 | 0.2 |  |
| 60-69 yrs \* Central-West | 0.17 | 0.335 | -0.49, 0.83 | 0.6 |  |
| 70-79 yrs \* Central-West | 0.13 | 0.348 | -0.55, 0.82 | 0.7 |  |
| 80+ \* Central-West | 0.38 | 0.452 | -0.51, 1.3 | 0.4 |  |
| 30-39 yrs \* South | 0.12 | 0.293 | -0.46, 0.69 | 0.7 |  |
| 40-49 yrs \* South | 0.10 | 0.312 | -0.51, 0.72 | 0.7 |  |
| 50-59 yrs \* South | 0.48 | 0.298 | -0.10, 1.1 | 0.11 |  |
| 60-69 yrs \* South | -0.19 | 0.366 | -0.91, 0.53 | 0.6 |  |
| 70-79 yrs \* South | -0.13 | 0.379 | -0.88, 0.61 | 0.7 |  |
| 80+ \* South | 0.48 | 0.489 | -0.48, 1.4 | 0.3 |  |
| Age \* Country of birth |  |  |  |  | 0.042 |
| 30-39 yrs \* In another country | 0.15 | 0.272 | -0.38, 0.69 | 0.6 |  |
| 40-49 yrs \* In another country | -0.29 | 0.291 | -0.86, 0.28 | 0.3 |  |
| 50-59 yrs \* In another country | -0.24 | 0.278 | -0.79, 0.31 | 0.4 |  |
| 60-69 yrs \* In another country | -0.44 | 0.371 | -1.2, 0.29 | 0.2 |  |
| 70-79 yrs \* In another country | -0.34 | 0.339 | -1.0, 0.33 | 0.3 |  |
| 80+ \* In another country | 0.75 | 0.394 | -0.03, 1.5 | 0.059 |  |
| Sex \* Country of birth |  |  |  |  | >0.9 |
| Female \* In another country | -0.02 | 0.178 | -0.37, 0.34 | >0.9 |  |
| Obesity \* Education: CASMIN |  |  |  |  | 0.099 |
| No \* Medium | 0.08 | 0.191 | -0.30, 0.45 | 0.7 |  |
| No \* High | 0.45 | 0.240 | -0.02, 0.93 | 0.060 |  |
| Education: CASMIN \* Overweight |  |  |  |  | 0.68 |
| Medium \* No | 0.10 | 0.160 | -0.21, 0.42 | 0.5 |  |
| High \* No | 0.00 | 0.176 | -0.35, 0.34 | >0.9 |  |
| Smoking \* Education: CASMIN |  |  |  |  | 0.16 |
| Occasional smoking \* Medium | -0.63 | 0.320 | -1.3, 0.00 | 0.049 |  |
| Non-smoker \* Medium | -0.02 | 0.189 | -0.39, 0.35 | >0.9 |  |
| Occasional smoking \* High | -0.95 | 0.396 | -1.7, -0.16 | 0.018 |  |
| Non-smoker \* High | -0.22 | 0.250 | -0.71, 0.28 | 0.4 |  |
| Education: CASMIN \* Waited for medical examination date in the last 12 months |  |  |  |  | 0.09 |
| Medium \* No | -0.19 | 0.164 | -0.52, 0.13 | 0.2 |  |
| High \* No | 0.17 | 0.179 | -0.18, 0.52 | 0.3 |  |
| Medium \* No need for examination or treatment | 0.16 | 0.249 | -0.33, 0.66 | 0.5 |  |
| High \* No need for examination or treatment | 0.23 | 0.261 | -0.28, 0.74 | 0.4 |  |
| Education: CASMIN \* BIK community size (categorized) |  |  |  |  | 0.577 |
| Medium \* BIK region 20,000 to <50,000 inhabitants OR surroundings 50,000 to <500,000 inhabitants | 0.21 | 0.196 | -0.18, 0.60 | 0.3 |  |
| High \* BIK region 20,000 to <50,000 inhabitants OR surroundings 50,000 to <500,000 inhabitants | 0.52 | 0.264 | 0.00, 1.0 | 0.050 |  |
| Medium \* Core city 50,000 to <500,000 inhabitants OR surroundings 500,000+ inhabitants | 0.06 | 0.207 | -0.35, 0.47 | 0.8 |  |
| High \* Core city 50,000 to <500,000 inhabitants OR surroundings 500,000+ inhabitants | 0.24 | 0.284 | -0.32, 0.80 | 0.4 |  |
| Medium \* Core city 500,000+ inhabitants | 0.13 | 0.221 | -0.30, 0.57 | 0.5 |  |
| High \* Core city 500,000+ inhabitants | 0.38 | 0.281 | -0.18, 0.93 | 0.2 |  |
| Education: CASMIN \* Current living situation |  |  |  |  | 0.272 |
| Medium \* Part-time employed | 0.49 | 0.230 | 0.04, 0.95 | 0.034 |  |
| High \* Part-time employed | 0.28 | 0.254 | -0.22, 0.78 | 0.3 |  |
| Medium \* Unemployed | -0.07 | 0.320 | -0.71, 0.56 | 0.8 |  |
| High \* Unemployed | 0.44 | 0.417 | -0.39, 1.3 | 0.3 |  |
| Medium \* Retired or early retired | 0.09 | 0.196 | -0.30, 0.47 | 0.7 |  |
| High \* Retired or early retired | 0.22 | 0.239 | -0.25, 0.70 | 0.4 |  |
| Medium \* Not employed for other reasons (student, volunteer service, homemaker) | 0.38 | 0.251 | -0.11, 0.87 | 0.13 |  |
| High \* Not employed for other reasons (student, volunteer service, homemaker) | 0.20 | 0.299 | -0.39, 0.79 | 0.5 |  |
| N.Obs | 11,800 |  |  |  |  |
| N.Cluster | 359 |  |  |  |  |
|  |  |  |  |  |  |
| --- | --- | --- | --- | --- | --- |
| Abbreviations: CI = Confidence Interval, OR = Odds Ratio, SE = Standard Error | | | | | |
