# Supplementary material for: Weighting strategy and selection analysis in the panel ‘Health in Germany‘: methods and results for the 2024 annual survey
Source: BMC Med Res Methodol. 2025 Dec 15;26:8. doi: 10.1186/s12874-025-02740-w (PMC12822093; doi:10.1186/s12874-025-02740-w)
Supplement: Supplementary file 2 — Supplementary Material 2. [file 12874_2025_2740_MOESM2_ESM.html]

Additional File 2 - Standardized Diffferences Questionnaire A to D


- Additional File 2 - Standardized
  Diffferences Questionnaire A to D

- Questionnaire A
  - Female
    and male
  - Female
  - Male
- Questionnaire B
  - Female and male
  - Female
  - Male
- Questionnaire C
  - Female and male
  - Female
  - Male
- Questionnaire D
  - Female and male
  - Female
  - Male

- 2025-11-24

# Additional File 2 - Standardized Diffferences Questionnaire A to D

# Questionnaire A

## Female and male

Table 1.1: Standardized differences in percentage points compared to reference values for the recruitment survey, registration sample, and the sample of participants in Questionnaire A; calculated unweighted and weighted according to the respective weighting factors.

|  |  | Recruitment Survey | | | Registration | | | | Participation Questionnaire A | | | | | |
| --- | --- | --- | --- | --- | --- | --- | --- | --- | --- | --- | --- | --- | --- | --- |
| Parameter | Reference [%] | Unweighted | Design weight | CSa weight | Unweighted | Design weight | CSa weight | Drop-out Weight | Unweighted | Design weight | CSa weight (Recruitment Study) | Drop-out weight (Registration) | Drop-out weight | CSa weight |
| sex |  |  |  |  |  |  |  |  |  |  |  |  |  |  |
| Male | 48.9 | -2.0 | -1.9 | 0.0 | -2.2 | -2.1 | 0.0 | 0.0 | -3.4 | -3.2 | -1.3 | -1.4 | -0.1 | 0.0 |
| Female | 51.1 | 2.0 | 1.9 | 0.0 | 2.2 | 2.1 | 0.0 | 0.0 | 3.4 | 3.2 | 1.3 | 1.4 | 0.1 | 0.0 |
| Agegrp |  |  |  |  |  |  |  |  |  |  |  |  |  |  |
| 18-29 yrs | 16.0 | 0.6 | -0.3 | -0.1 | 1.7 | 0.7 | 0.9 | -0.3 | -2.4 | -2.9 | -3.2 | -4.4 | -0.7 | 0.2 |
| 30-39 yrs | 15.8 | -0.9 | -0.1 | 0.0 | 0.3 | 1.1 | 1.4 | 0.1 | -2.1 | -1.4 | -1.3 | -2.6 | -0.1 | 0.1 |
| 40-49 yrs | 14.7 | -3.8 | -0.1 | 0.1 | -3.3 | 0.4 | 0.7 | 0.1 | -4.1 | -0.3 | -0.1 | -0.9 | 0.1 | -0.2 |
| 50-59 yrs | 17.6 | 0.4 | 0.6 | 0.4 | 0.8 | 0.9 | 0.7 | 0.4 | 2.0 | 2.3 | 2.2 | 1.8 | 0.8 | 0.4 |
| 60-69 yrs | 16.5 | -0.1 | -0.1 | -0.1 | -0.1 | -0.2 | -0.2 | 0.0 | 2.3 | 2.2 | 2.7 | 2.8 | 0.1 | -0.2 |
| 70-79 yrs | 10.7 | 3.5 | 1.7 | -0.2 | 2.4 | 0.4 | -1.4 | -0.1 | 5.1 | 3.0 | 1.2 | 2.5 | 0.0 | -0.1 |
| 80+ yrs | 8.7 | 0.6 | -1.8 | -0.1 | -2.3 | -4.6 | -3.2 | -0.1 | -1.4 | -3.8 | -2.3 | 0.8 | -0.2 | -0.4 |
| Federal state |  |  |  |  |  |  |  |  |  |  |  |  |  |  |
| Schleswig-Holstein | 3.5 | 12.5 | 0.0 | 0.0 | 12.6 | 0.1 | 0.2 | 0.1 | 12.9 | 0.2 | 0.2 | 0.1 | 0.5 | 0.0 |
| Hamburg | 2.2 | 3.9 | -0.1 | 0.0 | 4.3 | 0.3 | 0.4 | 0.2 | 4.0 | 0.1 | 0.1 | -0.1 | 0.3 | 0.0 |
| Lower Saxony | 9.6 | -3.8 | 0.1 | 0.1 | -3.9 | 0.0 | 0.0 | -0.1 | -4.0 | -0.2 | -0.4 | -0.5 | -0.4 | 0.0 |
| Bremen | 0.8 | 8.3 | -0.1 | 0.0 | 8.6 | 0.1 | 0.1 | 0.1 | 8.5 | 0.0 | -0.1 | -0.1 | 0.2 | 0.0 |
| North Rhine-Westphalia | 21.4 | -7.2 | 0.0 | 0.0 | -7.2 | 0.2 | 0.1 | -0.2 | -7.1 | 0.2 | 0.0 | -0.3 | -0.3 | 0.0 |
| Hesse | 7.6 | -4.4 | 0.0 | 0.0 | -4.5 | -0.2 | -0.2 | -0.2 | -5.6 | -1.6 | -1.6 | -1.7 | -1.4 | 0.0 |
| Rhineland-Palatinate | 4.9 | -3.5 | 0.0 | 0.0 | -3.2 | 0.5 | 0.5 | 0.5 | -2.9 | 0.9 | 0.9 | 1.0 | 1.0 | 0.0 |
| Baden-Württemberg | 13.3 | -5.9 | -0.1 | 0.0 | -5.6 | 0.1 | 0.1 | -0.1 | -5.8 | -0.1 | -0.2 | -0.3 | -0.8 | 0.0 |
| Bavaria | 15.9 | -5.7 | 0.0 | 0.0 | -5.8 | -0.1 | -0.2 | 0.0 | -5.2 | 0.8 | 0.7 | 0.7 | 0.4 | 0.0 |
| Saarland | 1.2 | 7.1 | 0.0 | 0.0 | 7.0 | 0.0 | 0.0 | 0.1 | 7.2 | 0.2 | 0.2 | 0.3 | 0.3 | 0.0 |
| Berlin | 4.5 | 10.5 | 0.0 | 0.0 | 10.9 | 0.3 | 0.4 | 0.3 | 10.4 | -0.1 | 0.3 | 0.1 | 0.9 | 0.0 |
| Brandenburg | 3.1 | 0.8 | 0.0 | -0.1 | 0.5 | -0.2 | -0.2 | -0.1 | 0.4 | -0.4 | -0.3 | -0.1 | 0.0 | 0.0 |
| Mecklenburg-Western Pomerania | 2.0 | 4.0 | 0.0 | 0.0 | 3.5 | -0.3 | -0.3 | -0.2 | 3.5 | -0.3 | -0.3 | -0.2 | 0.0 | 0.0 |
| Saxony | 4.9 | -2.6 | 0.0 | 0.0 | -2.9 | -0.4 | -0.3 | 0.0 | -2.4 | 0.2 | 0.4 | 0.8 | 0.3 | 0.0 |
| Saxony-Anhalt | 2.6 | 1.8 | 0.0 | 0.0 | 1.4 | -0.3 | -0.2 | 0.2 | 1.7 | -0.1 | 0.0 | 0.5 | 0.3 | 0.0 |
| Thuringia | 2.5 | 2.0 | 0.0 | 0.0 | 1.4 | -0.5 | -0.5 | -0.1 | 1.8 | -0.1 | -0.1 | 0.4 | 0.3 | 0.0 |
| German |  |  |  |  |  |  |  |  |  |  |  |  |  |  |
| Yes | 85.0 | 14.2 | 13.6 | 12.5 | 16.1 | 15.7 | 15.0 | 12.6 | 19.0 | 18.7 | 18.2 | 16.4 | 13.7 | 13.6 |
| No | 15.0 | -14.2 | -13.6 | -12.5 | -16.1 | -15.7 | -15.0 | -12.6 | -19.0 | -18.7 | -18.2 | -16.4 | -13.7 | -13.6 |
| BIK |  |  |  |  |  |  |  |  |  |  |  |  |  |  |
| BIK 1 | 10.8 | -0.8 | -0.4 | 0.0 | -1.4 | -0.9 | -0.5 | 0.0 | -1.7 | -1.1 | -0.6 | 0.0 | 0.3 | 0.0 |
| BIK 2 | 34.6 | -1.2 | 1.1 | 0.0 | -1.8 | 0.6 | -0.6 | 0.1 | -1.1 | 1.5 | 0.2 | 0.9 | 0.1 | 0.0 |
| BIK 3 | 26.8 | -2.4 | -0.6 | 0.0 | -2.2 | -0.4 | 0.2 | -0.1 | -2.4 | -0.8 | -0.2 | -0.5 | -0.2 | 0.0 |
| BIK 4 | 27.9 | 4.1 | -0.3 | 0.0 | 4.9 | 0.3 | 0.7 | 0.0 | 4.6 | 0.0 | 0.3 | -0.5 | -0.1 | 0.0 |
| Education |  |  |  |  |  |  |  |  |  |  |  |  |  |  |
| Low | 34.4 | -12.8 | -12.8 | -1.3 | -16.8 | -16.8 | -5.8 | -1.5 | -17.4 | -17.6 | -7.3 | -3.1 | -3.0 | -1.1 |
| Medium | 45.3 | 2.3 | 2.5 | 1.4 | 3.3 | 3.5 | 3.4 | 1.5 | 2.5 | 2.8 | 3.4 | 1.6 | 2.4 | 1.0 |
| High | 20.3 | 10.7 | 10.5 | -0.1 | 13.2 | 12.9 | 2.3 | 0.0 | 14.6 | 14.4 | 4.1 | 1.7 | 0.6 | 0.0 |
| Household size |  |  |  |  |  |  |  |  |  |  |  |  |  |  |
| Single-person household | 25.4 | -3.5 | -4.8 | 0.2 | -4.6 | -5.8 | -0.9 | 0.1 | -4.7 | -5.6 | -0.9 | 0.1 | 0.0 | 0.5 |
| Multi-person household | 74.6 | 3.5 | 4.8 | -0.2 | 4.6 | 5.8 | 0.9 | -0.1 | 4.7 | 5.6 | 0.9 | -0.1 | 0.0 | -0.5 |
| PAbmi\_k2 |  |  |  |  |  |  |  |  |  |  |  |  |  |  |
| Normal weight (18.5 <= BMI < 25) | 40.7 | 2.0 | 1.9 | 0.0 | 2.5 | 2.5 | 0.4 | -0.1 | 2.3 | 2.4 | 0.4 | -0.1 | -0.1 | -0.2 |
| Underweight (BMI < 18.5) | 2.0 | 0.2 | -0.1 | 0.0 | 0.2 | -0.1 | -0.1 | -0.1 | 0.1 | -0.4 | -0.4 | -0.4 | -0.1 | 0.1 |
| Overweight (25 <= BMI < 30) | 35.2 | -0.3 | -0.3 | 0.0 | -0.7 | -0.7 | -0.4 | 0.1 | 0.0 | -0.2 | 0.2 | 0.7 | 0.1 | 0.0 |
| Obesity (BMI >= 30) | 22.1 | -2.2 | -1.9 | 0.0 | -2.3 | -2.1 | -0.1 | 0.0 | -2.8 | -2.5 | -0.6 | -0.5 | 0.1 | 0.2 |
| Self-rated health |  |  |  |  |  |  |  |  |  |  |  |  |  |  |
| Very good/good/fair | 93.1 | 0.9 | 1.3 | 0.0 | 2.4 | 2.7 | 1.5 | 0.0 | 2.8 | 3.1 | 2.0 | 0.7 | 0.5 | 0.7 |
| Bad/very bad | 6.9 | -0.9 | -1.3 | 0.0 | -2.4 | -2.7 | -1.5 | 0.0 | -2.8 | -3.1 | -2.0 | -0.7 | -0.5 | -0.7 |
| Self-rated mental health |  |  |  |  |  |  |  |  |  |  |  |  |  |  |
| Excellent/very good/good | 78.5 | 1.3 | 1.6 | 0.0 | 1.7 | 1.9 | 0.3 | 0.0 | 2.3 | 2.7 | 1.1 | 0.7 | 0.0 | 0.0 |
| fair/poor | 21.5 | -1.3 | -1.6 | 0.0 | -1.7 | -1.9 | -0.3 | 0.0 | -2.3 | -2.7 | -1.1 | -0.7 | 0.0 | 0.0 |
| Paying attention to health |  |  |  |  |  |  |  |  |  |  |  |  |  |  |
| Not at all/less strong/moderate | 51.6 | -1.7 | -1.6 | 0.0 | -2.2 | -2.2 | -0.5 | 0.0 | -3.7 | -3.7 | -2.1 | -1.4 | -0.1 | 0.2 |
| Strong/very strong | 48.4 | 1.7 | 1.6 | 0.0 | 2.2 | 2.2 | 0.5 | 0.0 | 3.7 | 3.7 | 2.1 | 1.4 | 0.1 | -0.2 |
| Satisfaction: Life in general |  |  |  |  |  |  |  |  |  |  |  |  |  |  |
| Scale value 1 to 3 | 8.5 | -1.4 | -1.5 | 0.0 | -2.0 | -2.1 | -0.5 | -0.1 | -3.1 | -3.2 | -1.7 | -1.3 | -0.3 | 0.0 |
| Scale value 4 to 7 | 45.1 | -0.9 | -1.0 | 0.0 | -1.3 | -1.4 | -0.5 | 0.1 | -2.3 | -2.4 | -1.5 | -1.0 | 0.1 | 0.1 |
| Scale value 8 to 10 | 46.4 | 1.7 | 1.8 | 0.0 | 2.4 | 2.5 | 0.7 | 0.0 | 3.9 | 4.1 | 2.4 | 1.7 | 0.1 | -0.1 |
| Red meat |  |  |  |  |  |  |  |  |  |  |  |  |  |  |
| Never | 9.3 | 1.2 | 0.6 | 0.0 | 1.7 | 1.0 | 0.4 | 0.0 | 1.7 | 1.1 | 0.5 | 0.0 | -0.3 | -0.1 |
| Daily or several times a day | 2.8 | -1.6 | -1.1 | 0.0 | -2.1 | -1.7 | -0.4 | 0.0 | -3.3 | -3.0 | -1.9 | -1.7 | -0.4 | -0.2 |
| 4 to 6 times per week | 12.2 | -1.4 | -0.4 | 0.0 | -1.6 | -0.5 | 0.1 | 0.0 | -2.8 | -1.7 | -1.0 | -1.0 | -0.2 | 0.0 |
| 1 to 3 times per week | 46.1 | -0.6 | -0.1 | 0.0 | -1.1 | -0.5 | -0.4 | 0.0 | -0.8 | -0.3 | -0.3 | 0.0 | 0.0 | -0.2 |
| Less than once per week | 29.6 | 1.4 | 0.4 | 0.0 | 1.9 | 0.9 | 0.3 | 0.0 | 2.7 | 1.7 | 1.4 | 1.2 | 0.5 | 0.3 |
| Sausage products |  |  |  |  |  |  |  |  |  |  |  |  |  |  |
| Never | 10.3 | 1.5 | 1.1 | 0.0 | 2.1 | 1.6 | 0.4 | -0.1 | 1.7 | 1.2 | -0.1 | -0.7 | -0.5 | -0.5 |
| Daily or several times a day | 10.0 | -0.7 | -1.0 | 0.0 | -1.2 | -1.4 | -0.3 | 0.0 | -1.8 | -2.1 | -0.9 | -0.6 | -0.1 | -0.1 |
| 4 to 6 times per week | 19.4 | -0.6 | -0.2 | 0.0 | -0.3 | 0.1 | 0.4 | 0.0 | -0.2 | 0.3 | 0.5 | 0.2 | 0.2 | 0.1 |
| 1 to 3 times per week | 35.6 | -0.7 | -0.3 | 0.0 | -1.3 | -0.8 | -0.4 | 0.0 | -0.9 | -0.4 | -0.1 | 0.4 | 0.0 | 0.0 |
| Less than once per week | 24.8 | 0.8 | 0.4 | 0.0 | 1.0 | 0.6 | 0.1 | 0.1 | 1.2 | 0.7 | 0.3 | 0.3 | 0.2 | 0.3 |
| Smoking |  |  |  |  |  |  |  |  |  |  |  |  |  |  |
| Non-smoker | 76.2 | 3.5 | 3.2 | 0.0 | 4.1 | 3.8 | 0.5 | 0.0 | 7.4 | 7.1 | 4.4 | 4.1 | 0.8 | 0.6 |
| Daily smoking | 17.9 | -3.9 | -3.5 | 0.0 | -4.5 | -4.1 | -0.5 | -0.1 | -7.5 | -7.1 | -4.2 | -3.9 | -0.9 | -0.7 |
| Occasional smoking | 5.9 | -0.1 | -0.1 | 0.0 | -0.1 | -0.2 | -0.1 | 0.0 | -1.4 | -1.5 | -1.3 | -1.1 | -0.1 | 0.0 |
| Chronic diseases |  |  |  |  |  |  |  |  |  |  |  |  |  |  |
| No | 45.0 | -0.1 | 0.7 | 0.0 | -0.3 | 0.4 | -0.4 | -0.1 | -2.2 | -1.3 | -2.1 | -1.8 | -0.3 | -0.1 |
| Yes | 55.0 | 0.1 | -0.7 | 0.0 | 0.3 | -0.4 | 0.4 | 0.1 | 2.2 | 1.3 | 2.1 | 1.8 | 0.3 | 0.1 |
| Sport |  |  |  |  |  |  |  |  |  |  |  |  |  |  |
| No sporting activities | 23.0 | -2.6 | -3.1 | 0.0 | -4.7 | -5.2 | -2.3 | -0.1 | -5.7 | -6.4 | -3.7 | -1.5 | -0.3 | -0.2 |
| Less than 1 hour per week | 19.7 | -0.9 | -0.5 | 0.0 | -1.0 | -0.7 | -0.1 | 0.0 | -1.8 | -1.3 | -0.9 | -0.7 | -0.1 | -0.1 |
| 1 to less than 2 hours per week | 26.5 | 1.0 | 1.0 | 0.0 | 1.4 | 1.4 | 0.5 | 0.1 | 2.4 | 2.5 | 1.6 | 1.2 | 0.4 | 0.2 |
| 2 to less than 4 hours per week | 18.9 | 1.7 | 1.8 | 0.0 | 2.8 | 3.0 | 1.2 | 0.0 | 3.6 | 3.7 | 2.3 | 1.1 | 0.1 | 0.0 |
| 4 hours per week and more | 11.9 | 0.8 | 0.9 | 0.0 | 1.6 | 1.8 | 0.9 | 0.0 | 1.3 | 1.5 | 0.8 | -0.2 | -0.1 | 0.0 |
| KWsubj\_k |  |  |  |  |  |  |  |  |  |  |  |  |  |  |
| Scale value 1 to 3 | 34.8 | -0.3 | -0.4 | 0.0 | -0.8 | -0.9 | -0.4 | 0.0 | -0.9 | -1.0 | -0.9 | -0.6 | 0.0 | -0.1 |
| Scale value 4 to 7 | 49.0 | 0.1 | 0.1 | 0.0 | 0.1 | 0.2 | 0.2 | 0.0 | 0.6 | 0.5 | 0.8 | 0.7 | 0.1 | 0.3 |
| Scale value 8 to 10 | 16.1 | 0.3 | 0.4 | 0.0 | 0.8 | 0.8 | 0.3 | -0.1 | 0.4 | 0.6 | 0.2 | -0.2 | -0.1 | -0.2 |
| IAtermin |  |  |  |  |  |  |  |  |  |  |  |  |  |  |
| No | 54.8 | 0.9 | 0.3 | 0.0 | 0.6 | -0.1 | -0.5 | 0.0 | 2.4 | 1.8 | 1.4 | 1.9 | 0.4 | 0.4 |
| Yes | 33.5 | -0.7 | -0.3 | 0.0 | -0.5 | -0.2 | 0.3 | 0.0 | -1.9 | -1.7 | -1.2 | -1.5 | -0.3 | -0.3 |
| No need for examination or treatment | 11.7 | -0.4 | 0.1 | 0.0 | -0.1 | 0.3 | 0.3 | 0.0 | -1.0 | -0.3 | -0.4 | -0.8 | -0.2 | -0.1 |
|  |  |  |  |  |  |  |  |  |  |  |  |  |  |  |
| --- | --- | --- | --- | --- | --- | --- | --- | --- | --- | --- | --- | --- | --- | --- |
| aCS = Cross-Sectional | | | | | | | | | | | | | | |

## Female

Table 1.2: Standardized differences in percentage points compared to reference values for the recruitment survey, registration sample, and the sample of participants in Questionnaire A; calculated unweighted and weighted according to the respective weighting factors.

|  |  | Recruitment Survey | | | Registration | | | | Participation Questionnaire A | | | | | |
| --- | --- | --- | --- | --- | --- | --- | --- | --- | --- | --- | --- | --- | --- | --- |
| Parameter | Reference [%] | Unweighted | Design weight | CSa weight | Unweighted | Design weight | CSa weight | Drop-out Weight | Unweighted | Design weight | CSa weight (Recruitment Study) | Drop-out weight (Registration) | Drop-out weight | CSa weight |
| Agegrp |  |  |  |  |  |  |  |  |  |  |  |  |  |  |
| 18-29 yrs | 14.9 | 2.2 | 1.3 | -0.1 | 3.9 | 2.9 | 1.7 | 0.1 | 0.8 | 0.1 | -1.3 | -2.9 | 0.5 | 0.4 |
| 30-39 yrs | 15.1 | 0.2 | 0.9 | 0.2 | 1.5 | 2.3 | 1.9 | 0.1 | -0.7 | 0.2 | -0.5 | -2.2 | -0.2 | 0.0 |
| 40-49 yrs | 14.4 | -2.9 | 1.0 | 0.3 | -2.4 | 1.6 | 0.9 | 0.1 | -3.1 | 0.8 | 0.0 | -1.1 | -0.2 | -0.3 |
| 50-59 yrs | 17.2 | 1.3 | 1.4 | 0.7 | 1.9 | 1.9 | 1.3 | 0.8 | 3.3 | 3.4 | 2.7 | 2.0 | 1.0 | 0.5 |
| 60-69 yrs | 16.6 | -0.6 | -0.7 | 0.2 | -0.7 | -1.0 | 0.0 | 0.1 | 1.3 | 1.0 | 2.3 | 2.5 | 0.1 | -0.2 |
| 70-79 yrs | 11.4 | 1.5 | -0.4 | 0.0 | 0.1 | -1.9 | -1.5 | 0.3 | 2.5 | 0.3 | 0.8 | 2.7 | 0.4 | 0.1 |
| 80+ yrs | 10.4 | -2.2 | -4.6 | -1.5 | -6.0 | -8.2 | -5.8 | -1.6 | -5.5 | -7.8 | -5.5 | -1.5 | -2.0 | -0.7 |
| Federal state |  |  |  |  |  |  |  |  |  |  |  |  |  |  |
| Schleswig-Holstein | 3.5 | 12.7 | 0.1 | 0.0 | 12.9 | 0.3 | 0.2 | 0.0 | 13.4 | 0.6 | 0.6 | 0.4 | 0.7 | 0.0 |
| Hamburg | 2.3 | 4.4 | 0.3 | 0.0 | 4.8 | 0.7 | 0.3 | 0.1 | 4.5 | 0.5 | 0.2 | 0.0 | 0.4 | 0.0 |
| Lower Saxony | 9.6 | -3.9 | 0.0 | 0.1 | -3.9 | 0.1 | 0.2 | 0.0 | -3.8 | 0.2 | 0.1 | -0.1 | -0.1 | 0.0 |
| Bremen | 0.8 | 8.4 | 0.0 | -0.1 | 8.7 | 0.2 | 0.2 | 0.1 | 8.8 | 0.2 | 0.2 | 0.1 | 0.3 | 0.0 |
| North Rhine-Westphalia | 21.5 | -7.1 | 0.2 | 0.0 | -7.2 | 0.4 | 0.0 | -0.2 | -7.3 | 0.2 | -0.2 | -0.5 | -0.2 | 0.0 |
| Hesse | 7.6 | -4.8 | -0.4 | 0.0 | -5.4 | -1.0 | -0.5 | -0.4 | -6.7 | -2.8 | -2.2 | -2.3 | -1.9 | -0.1 |
| Rhineland-Palatinate | 4.9 | -3.8 | -0.3 | -0.1 | -3.4 | 0.2 | 0.5 | 0.6 | -3.3 | 0.4 | 0.6 | 0.7 | 0.8 | 0.0 |
| Baden-Württemberg | 13.2 | -5.8 | 0.1 | 0.0 | -5.7 | 0.1 | -0.1 | -0.2 | -6.1 | -0.3 | -0.6 | -0.7 | -1.1 | 0.0 |
| Bavaria | 15.8 | -5.6 | 0.1 | -0.1 | -5.6 | 0.2 | 0.0 | 0.1 | -5.0 | 1.1 | 0.8 | 0.8 | 0.4 | 0.0 |
| Saarland | 1.2 | 6.9 | 0.0 | 0.0 | 6.7 | -0.1 | -0.1 | 0.0 | 6.6 | -0.1 | 0.0 | 0.1 | 0.4 | 0.0 |
| Berlin | 4.5 | 10.2 | -0.2 | 0.0 | 10.8 | 0.3 | 0.6 | 0.3 | 10.4 | 0.0 | 0.4 | 0.2 | 1.0 | 0.0 |
| Brandenburg | 3.1 | 0.8 | 0.1 | -0.1 | 0.6 | -0.2 | -0.3 | -0.2 | 0.5 | -0.4 | -0.4 | -0.3 | -0.2 | 0.0 |
| Mecklenburg-Western Pomerania | 2.0 | 4.2 | 0.2 | 0.0 | 3.9 | 0.0 | -0.1 | 0.0 | 3.8 | -0.1 | -0.1 | -0.1 | 0.1 | 0.0 |
| Saxony | 4.9 | -2.5 | 0.0 | 0.1 | -2.8 | -0.5 | -0.4 | 0.0 | -2.0 | 0.5 | 0.8 | 1.3 | 0.6 | 0.0 |
| Saxony-Anhalt | 2.6 | 1.5 | -0.2 | 0.0 | 1.3 | -0.4 | -0.1 | 0.3 | 1.9 | 0.1 | 0.5 | 0.9 | 0.6 | 0.0 |
| Thuringia | 2.5 | 1.7 | -0.1 | 0.0 | 1.1 | -0.6 | -0.4 | 0.0 | 1.3 | -0.3 | -0.1 | 0.4 | 0.0 | 0.0 |
| German |  |  |  |  |  |  |  |  |  |  |  |  |  |  |
| Yes | 85.8 | 12.7 | 12.0 | 11.1 | 14.5 | 14.1 | 13.4 | 11.1 | 17.1 | 17.0 | 16.4 | 14.5 | 11.5 | 11.5 |
| No | 14.2 | -12.7 | -12.0 | -11.1 | -14.5 | -14.1 | -13.4 | -11.1 | -17.1 | -17.0 | -16.4 | -14.5 | -11.5 | -11.5 |
| BIK |  |  |  |  |  |  |  |  |  |  |  |  |  |  |
| BIK 1 | 10.7 | -0.8 | -0.4 | 0.0 | -1.4 | -0.8 | -0.4 | 0.2 | -1.6 | -0.9 | -0.2 | 0.5 | 0.8 | 0.7 |
| BIK 2 | 34.4 | -1.3 | 0.9 | 0.1 | -1.9 | 0.5 | -0.4 | 0.2 | -1.4 | 1.1 | 0.1 | 0.6 | -0.2 | -0.3 |
| BIK 3 | 26.9 | -2.3 | -0.3 | 0.2 | -2.2 | -0.2 | 0.5 | 0.1 | -2.3 | -0.5 | 0.1 | -0.1 | 0.1 | 0.3 |
| BIK 4 | 28.1 | 4.1 | -0.5 | -0.3 | 5.0 | 0.2 | 0.2 | -0.5 | 4.7 | -0.1 | -0.1 | -0.8 | -0.5 | -0.4 |
| Education |  |  |  |  |  |  |  |  |  |  |  |  |  |  |
| Low | 33.8 | -14.0 | -14.2 | -1.2 | -18.7 | -18.9 | -6.5 | -1.7 | -19.3 | -19.9 | -8.3 | -3.4 | -3.2 | -0.4 |
| Medium | 47.6 | 3.4 | 3.6 | 1.4 | 4.8 | 5.1 | 4.2 | 1.7 | 4.6 | 5.3 | 5.0 | 2.6 | 2.8 | 0.7 |
| High | 18.6 | 10.8 | 10.7 | -0.2 | 13.3 | 13.1 | 2.2 | -0.1 | 13.9 | 13.7 | 3.2 | 0.8 | 0.3 | -0.3 |
| Household size |  |  |  |  |  |  |  |  |  |  |  |  |  |  |
| Single-person household | 26.0 | -1.9 | -3.6 | 0.2 | -3.6 | -5.1 | -1.6 | 0.1 | -3.2 | -4.6 | -1.2 | 0.4 | -0.5 | 0.4 |
| Multi-person household | 74.0 | 1.9 | 3.6 | -0.2 | 3.6 | 5.1 | 1.6 | -0.1 | 3.2 | 4.6 | 1.2 | -0.4 | 0.5 | -0.4 |
| PAbmi\_k2 |  |  |  |  |  |  |  |  |  |  |  |  |  |  |
| Normal weight (18.5 <= BMI < 25) | 46.8 | 2.1 | 2.4 | 0.0 | 2.6 | 3.1 | 0.7 | 0.0 | 2.3 | 2.7 | 0.5 | -0.2 | -0.3 | -0.5 |
| Underweight (BMI < 18.5) | 2.9 | 0.2 | -0.1 | 0.0 | 0.2 | -0.1 | 0.1 | 0.0 | 0.1 | -0.3 | -0.2 | -0.3 | 0.0 | 0.1 |
| Overweight (25 <= BMI < 30) | 28.7 | -0.3 | -0.7 | 0.0 | -0.7 | -1.2 | -0.6 | 0.1 | 0.0 | -0.7 | 0.0 | 0.6 | 0.0 | 0.1 |
| Obesity (BMI >= 30) | 21.6 | -2.4 | -2.2 | 0.0 | -2.6 | -2.5 | -0.2 | -0.1 | -2.9 | -2.5 | -0.4 | -0.4 | 0.4 | 0.5 |
| Self-rated health |  |  |  |  |  |  |  |  |  |  |  |  |  |  |
| Very good/good/fair | 92.8 | 1.5 | 2.0 | 0.0 | 3.2 | 3.4 | 1.7 | -0.1 | 3.3 | 3.6 | 2.2 | 0.7 | 0.3 | 0.3 |
| Bad/very bad | 7.2 | -1.5 | -2.0 | 0.0 | -3.2 | -3.4 | -1.7 | 0.1 | -3.3 | -3.6 | -2.2 | -0.7 | -0.3 | -0.3 |
| Self-rated mental health |  |  |  |  |  |  |  |  |  |  |  |  |  |  |
| Excellent/very good/good | 75.6 | 1.3 | 1.7 | 0.0 | 1.7 | 2.1 | 0.6 | 0.2 | 2.3 | 2.8 | 1.5 | 1.1 | 0.2 | 0.2 |
| fair/poor | 24.3 | -1.3 | -1.7 | 0.0 | -1.7 | -2.1 | -0.6 | -0.2 | -2.3 | -2.8 | -1.5 | -1.1 | -0.2 | -0.2 |
| Paying attention to health |  |  |  |  |  |  |  |  |  |  |  |  |  |  |
| Not at all/less strong/moderate | 47.3 | -1.6 | -1.5 | 0.0 | -2.2 | -2.2 | -0.7 | -0.1 | -3.4 | -3.7 | -2.2 | -1.4 | 0.0 | 0.4 |
| Strong/very strong | 52.7 | 1.6 | 1.5 | 0.0 | 2.2 | 2.2 | 0.7 | 0.1 | 3.4 | 3.7 | 2.2 | 1.4 | 0.0 | -0.4 |
| Satisfaction: Life in general |  |  |  |  |  |  |  |  |  |  |  |  |  |  |
| Scale value 1 to 3 | 8.4 | -1.1 | -1.4 | 0.0 | -1.8 | -2.1 | -0.7 | -0.2 | -2.7 | -3.0 | -2.0 | -1.4 | -0.3 | -0.1 |
| Scale value 4 to 7 | 46.0 | -0.6 | -0.6 | 0.0 | -0.8 | -0.7 | -0.2 | 0.2 | -1.5 | -1.6 | -1.2 | -0.7 | 0.2 | 0.3 |
| Scale value 8 to 10 | 45.7 | 1.2 | 1.4 | 0.0 | 1.8 | 1.8 | 0.6 | -0.1 | 2.9 | 3.2 | 2.2 | 1.5 | -0.1 | -0.2 |
| Red meat |  |  |  |  |  |  |  |  |  |  |  |  |  |  |
| Never | 12.6 | 1.3 | 0.8 | 0.0 | 1.9 | 1.2 | 0.5 | 0.0 | 1.8 | 1.4 | 0.6 | 0.0 | -0.1 | -0.2 |
| Daily or several times a day | 1.2 | -1.0 | -1.0 | 0.0 | -1.4 | -1.4 | -0.2 | 0.0 | -2.2 | -2.5 | -1.3 | -1.1 | -0.1 | 0.2 |
| 4 to 6 times per week | 7.3 | -0.7 | 0.1 | 0.0 | -0.8 | -0.1 | 0.1 | 0.1 | -1.4 | -0.7 | -0.6 | -0.5 | 0.0 | 0.2 |
| 1 to 3 times per week | 42.6 | -1.3 | -0.5 | 0.0 | -2.0 | -1.0 | -0.6 | -0.1 | -2.0 | -1.1 | -0.8 | -0.3 | -0.3 | -0.5 |
| Less than once per week | 36.3 | 1.0 | 0.1 | 0.0 | 1.5 | 0.5 | 0.3 | 0.0 | 2.0 | 1.0 | 1.0 | 0.8 | 0.4 | 0.4 |
| Sausage products |  |  |  |  |  |  |  |  |  |  |  |  |  |  |
| Never | 13.2 | 2.3 | 1.7 | 0.0 | 3.1 | 2.4 | 0.6 | -0.1 | 2.7 | 2.3 | 0.5 | -0.4 | -0.3 | -0.4 |
| Daily or several times a day | 6.3 | -0.7 | -0.9 | 0.0 | -0.9 | -1.2 | -0.2 | -0.1 | -1.4 | -1.6 | -0.7 | -0.6 | -0.2 | 0.1 |
| 4 to 6 times per week | 13.9 | -0.4 | 0.1 | 0.0 | -0.1 | 0.4 | 0.5 | 0.1 | -0.2 | 0.3 | 0.3 | -0.1 | 0.0 | 0.0 |
| 1 to 3 times per week | 35.8 | -1.4 | -0.9 | 0.0 | -2.3 | -1.6 | -0.9 | 0.0 | -1.9 | -1.3 | -0.4 | 0.5 | 0.2 | 0.1 |
| Less than once per week | 30.8 | 0.4 | 0.0 | 0.0 | 0.6 | 0.2 | 0.2 | 0.1 | 0.7 | 0.2 | 0.2 | 0.2 | 0.1 | 0.2 |
| Smoking |  |  |  |  |  |  |  |  |  |  |  |  |  |  |
| Non-smoker | 79.4 | 2.4 | 2.3 | 0.0 | 2.5 | 2.3 | -0.2 | -0.4 | 5.6 | 5.5 | 3.8 | 3.6 | 0.8 | 0.4 |
| Daily smoking | 15.5 | -2.9 | -2.7 | 0.0 | -3.1 | -2.8 | 0.2 | 0.3 | -5.9 | -5.7 | -3.6 | -3.5 | -0.7 | -0.2 |
| Occasional smoking | 5.1 | 0.4 | 0.3 | 0.0 | 0.4 | 0.3 | 0.1 | 0.2 | -0.7 | -0.8 | -1.0 | -0.9 | -0.3 | -0.4 |
| Chronic diseases |  |  |  |  |  |  |  |  |  |  |  |  |  |  |
| No | 41.6 | 0.7 | 1.7 | 0.0 | 0.4 | 1.4 | -0.4 | 0.0 | -0.8 | 0.4 | -1.3 | -0.9 | 0.0 | -0.1 |
| Yes | 58.4 | -0.7 | -1.7 | 0.0 | -0.4 | -1.4 | 0.4 | 0.0 | 0.8 | -0.4 | 1.3 | 0.9 | 0.0 | 0.1 |
| Sport |  |  |  |  |  |  |  |  |  |  |  |  |  |  |
| No sporting activities | 22.8 | -3.2 | -3.7 | 0.0 | -5.9 | -6.3 | -3.0 | -0.1 | -7.2 | -7.6 | -4.6 | -1.8 | -0.3 | 0.1 |
| Less than 1 hour per week | 20.1 | -0.8 | -0.2 | 0.0 | -0.8 | -0.4 | 0.1 | 0.0 | -1.8 | -1.4 | -1.1 | -1.0 | -0.1 | 0.0 |
| 1 to less than 2 hours per week | 28.7 | 1.3 | 1.1 | 0.0 | 1.8 | 1.7 | 0.7 | 0.1 | 2.5 | 2.5 | 1.4 | 0.8 | 0.1 | -0.1 |
| 2 to less than 4 hours per week | 19.0 | 1.8 | 2.0 | 0.0 | 3.1 | 3.3 | 1.5 | 0.0 | 4.3 | 4.3 | 3.2 | 1.7 | 0.3 | 0.1 |
| 4 hours per week and more | 9.3 | 1.2 | 1.1 | 0.0 | 2.0 | 2.0 | 0.9 | 0.0 | 2.4 | 2.4 | 1.4 | 0.5 | 0.0 | -0.1 |
| KWsubj\_k |  |  |  |  |  |  |  |  |  |  |  |  |  |  |
| Scale value 1 to 3 | 29.9 | -0.3 | -0.5 | 0.0 | -0.7 | -0.9 | -0.4 | 0.2 | -0.3 | -0.5 | -0.4 | 0.1 | 0.5 | 0.4 |
| Scale value 4 to 7 | 53.3 | -0.1 | 0.0 | 0.0 | -0.1 | 0.1 | 0.2 | -0.1 | -0.1 | 0.0 | 0.4 | 0.3 | -0.2 | 0.1 |
| Scale value 8 to 10 | 16.8 | 0.5 | 0.6 | 0.0 | 1.0 | 1.0 | 0.2 | -0.2 | 0.5 | 0.7 | -0.1 | -0.6 | -0.4 | -0.6 |
| IAtermin |  |  |  |  |  |  |  |  |  |  |  |  |  |  |
| No | 53.9 | 0.1 | -0.4 | 0.0 | -0.6 | -1.1 | -0.9 | -0.1 | 0.9 | 0.4 | 0.7 | 1.5 | 0.4 | 0.6 |
| Yes | 36.1 | -0.4 | -0.1 | 0.0 | 0.2 | 0.4 | 0.7 | 0.2 | -1.3 | -1.1 | -1.0 | -1.5 | -0.1 | -0.3 |
| No need for examination or treatment | 10.0 | 0.4 | 0.8 | 0.0 | 0.7 | 1.1 | 0.3 | -0.1 | 0.5 | 1.1 | 0.4 | -0.1 | -0.4 | -0.5 |
|  |  |  |  |  |  |  |  |  |  |  |  |  |  |  |
| --- | --- | --- | --- | --- | --- | --- | --- | --- | --- | --- | --- | --- | --- | --- |
| aCS = Cross-Sectional | | | | | | | | | | | | | | |

## Male

Table 1.3: Standardized differences in percentage points compared to reference values for the recruitment survey, registration sample, and the sample of participants in Questionnaire A; calculated unweighted and weighted according to the respective weighting factors.

|  |  | Recruitment Survey | | | Registration | | | | Participation Questionnaire A | | | | | |
| --- | --- | --- | --- | --- | --- | --- | --- | --- | --- | --- | --- | --- | --- | --- |
| Parameter | Reference [%] | Unweighted | Design weight | CSa weight | Unweighted | Design weight | CSa weight | Drop-out Weight | Unweighted | Design weight | CSa weight (Recruitment Study) | Drop-out weight (Registration) | Drop-out weight | CSa weight |
| Agegrp |  |  |  |  |  |  |  |  |  |  |  |  |  |  |
| 18-29 yrs | 17.1 | -1.1 | -1.9 | -0.1 | -0.7 | -1.5 | 0.0 | -0.7 | -6.0 | -6.3 | -5.1 | -5.9 | -2.0 | 0.1 |
| 30-39 yrs | 16.6 | -2.0 | -1.2 | -0.1 | -1.0 | -0.1 | 1.0 | 0.1 | -3.6 | -3.2 | -2.1 | -3.1 | -0.1 | 0.1 |
| 40-49 yrs | 15.0 | -4.8 | -1.4 | -0.1 | -4.3 | -0.9 | 0.5 | 0.1 | -5.2 | -1.6 | -0.1 | -0.6 | 0.4 | 0.0 |
| 50-59 yrs | 17.9 | -0.7 | -0.3 | 0.0 | -0.4 | -0.3 | 0.0 | 0.0 | 0.6 | 1.0 | 1.7 | 1.7 | 0.6 | 0.4 |
| 60-69 yrs | 16.4 | 0.5 | 0.4 | -0.5 | 0.6 | 0.6 | -0.4 | -0.2 | 3.5 | 3.6 | 3.0 | 3.1 | 0.1 | -0.2 |
| 70-79 yrs | 10.0 | 5.7 | 3.9 | -0.5 | 4.8 | 3.0 | -1.3 | -0.7 | 8.1 | 6.1 | 1.6 | 2.2 | -0.4 | -0.3 |
| 80+ yrs | 7.0 | 3.7 | 1.5 | 1.7 | 1.9 | -0.5 | -0.3 | 1.8 | 3.4 | 0.9 | 1.3 | 3.5 | 1.9 | -0.2 |
| Federal state |  |  |  |  |  |  |  |  |  |  |  |  |  |  |
| Schleswig-Holstein | 3.5 | 12.2 | -0.1 | 0.0 | 12.3 | -0.1 | 0.1 | 0.2 | 12.2 | -0.3 | -0.2 | -0.2 | 0.2 | 0.0 |
| Hamburg | 2.2 | 3.3 | -0.5 | 0.0 | 3.7 | -0.2 | 0.4 | 0.3 | 3.4 | -0.5 | 0.0 | -0.2 | 0.1 | 0.0 |
| Lower Saxony | 9.6 | -3.6 | 0.1 | 0.1 | -3.9 | -0.2 | -0.1 | -0.1 | -4.2 | -0.6 | -0.8 | -0.8 | -0.7 | 0.0 |
| Bremen | 0.8 | 8.3 | -0.1 | 0.0 | 8.3 | -0.1 | -0.1 | 0.0 | 8.2 | -0.3 | -0.4 | -0.4 | 0.2 | 0.0 |
| North Rhine-Westphalia | 21.2 | -7.3 | -0.2 | 0.0 | -7.3 | 0.0 | 0.2 | -0.2 | -7.0 | 0.1 | 0.2 | -0.1 | -0.5 | 0.0 |
| Hesse | 7.6 | -4.1 | 0.4 | 0.0 | -3.7 | 0.7 | 0.1 | -0.1 | -4.4 | -0.2 | -0.9 | -1.1 | -0.9 | 0.0 |
| Rhineland-Palatinate | 5.0 | -3.3 | 0.3 | 0.0 | -2.9 | 0.7 | 0.5 | 0.4 | -2.5 | 1.3 | 1.3 | 1.2 | 1.1 | 0.0 |
| Baden-Württemberg | 13.4 | -5.9 | -0.3 | 0.0 | -5.5 | 0.0 | 0.2 | 0.1 | -5.4 | 0.1 | 0.2 | 0.1 | -0.5 | 0.0 |
| Bavaria | 16.0 | -5.8 | -0.2 | 0.0 | -5.9 | -0.4 | -0.3 | -0.2 | -5.4 | 0.5 | 0.5 | 0.7 | 0.4 | 0.0 |
| Saarland | 1.2 | 7.2 | 0.1 | 0.0 | 7.3 | 0.1 | 0.0 | 0.1 | 7.8 | 0.5 | 0.4 | 0.4 | 0.2 | 0.0 |
| Berlin | 4.4 | 10.7 | 0.1 | 0.0 | 11.0 | 0.2 | 0.3 | 0.2 | 10.4 | -0.2 | 0.1 | 0.0 | 0.9 | 0.0 |
| Brandenburg | 3.1 | 0.7 | 0.0 | -0.1 | 0.5 | -0.2 | -0.1 | 0.0 | 0.3 | -0.3 | -0.2 | 0.0 | 0.2 | 0.0 |
| Mecklenburg-Western Pomerania | 2.0 | 3.7 | -0.2 | 0.0 | 3.1 | -0.6 | -0.4 | -0.3 | 3.1 | -0.6 | -0.4 | -0.3 | 0.0 | 0.0 |
| Saxony | 4.9 | -2.8 | 0.0 | 0.0 | -3.0 | -0.2 | -0.3 | 0.0 | -2.9 | -0.3 | 0.0 | 0.3 | 0.0 | 0.0 |
| Saxony-Anhalt | 2.6 | 2.2 | 0.3 | 0.0 | 1.6 | -0.1 | -0.3 | 0.1 | 1.5 | -0.2 | -0.4 | 0.0 | 0.1 | 0.0 |
| Thuringia | 2.6 | 2.4 | 0.3 | 0.0 | 1.7 | -0.3 | -0.6 | -0.2 | 2.3 | 0.3 | -0.1 | 0.4 | 0.5 | 0.0 |
| German |  |  |  |  |  |  |  |  |  |  |  |  |  |  |
| Yes | 84.1 | 15.7 | 15.3 | 13.9 | 17.9 | 17.5 | 16.7 | 14.3 | 21.0 | 20.6 | 20.1 | 18.3 | 15.9 | 15.9 |
| No | 15.9 | -15.7 | -15.3 | -13.9 | -17.9 | -17.5 | -16.7 | -14.3 | -21.0 | -20.6 | -20.1 | -18.3 | -15.9 | -15.9 |
| BIK |  |  |  |  |  |  |  |  |  |  |  |  |  |  |
| BIK 1 | 10.9 | -0.9 | -0.4 | 0.0 | -1.5 | -1.0 | -0.6 | -0.1 | -1.9 | -1.4 | -1.0 | -0.6 | -0.3 | -0.7 |
| BIK 2 | 34.7 | -1.1 | 1.2 | -0.1 | -1.6 | 0.7 | -0.7 | 0.0 | -0.7 | 2.0 | 0.4 | 1.3 | 0.5 | 0.3 |
| BIK 3 | 26.6 | -2.5 | -0.9 | -0.3 | -2.2 | -0.6 | 0.0 | -0.3 | -2.7 | -1.3 | -0.5 | -0.9 | -0.6 | -0.4 |
| BIK 4 | 27.7 | 4.1 | -0.1 | 0.4 | 4.8 | 0.5 | 1.2 | 0.5 | 4.5 | 0.1 | 0.7 | -0.1 | 0.3 | 0.5 |
| Education |  |  |  |  |  |  |  |  |  |  |  |  |  |  |
| Low | 34.9 | -11.5 | -11.2 | -1.3 | -14.7 | -14.6 | -5.0 | -1.4 | -15.2 | -15.1 | -6.3 | -2.8 | -2.8 | -1.8 |
| Medium | 43.0 | 0.9 | 1.0 | 1.3 | 1.3 | 1.5 | 2.7 | 1.3 | -0.5 | -0.4 | 1.5 | 0.3 | 2.0 | 1.4 |
| High | 22.1 | 10.8 | 10.5 | 0.0 | 13.3 | 13.0 | 2.5 | 0.1 | 15.6 | 15.4 | 5.1 | 2.7 | 0.9 | 0.4 |
| Household size |  |  |  |  |  |  |  |  |  |  |  |  |  |  |
| Single-person household | 24.8 | -5.4 | -6.3 | 0.1 | -5.8 | -6.7 | -0.2 | 0.2 | -6.6 | -7.0 | -0.6 | -0.2 | 0.4 | 0.6 |
| Multi-person household | 75.2 | 5.4 | 6.3 | -0.1 | 5.8 | 6.7 | 0.2 | -0.2 | 6.6 | 7.0 | 0.6 | 0.2 | -0.4 | -0.6 |
| PAbmi\_k2 |  |  |  |  |  |  |  |  |  |  |  |  |  |  |
| Normal weight (18.5 <= BMI < 25) | 34.4 | 1.4 | 0.8 | 0.0 | 1.8 | 1.3 | 0.2 | -0.1 | 1.4 | 1.1 | 0.1 | -0.3 | 0.1 | 0.2 |
| Underweight (BMI < 18.5) | 1.1 | 0.0 | -0.5 | 0.0 | -0.3 | -0.7 | -0.4 | -0.4 | -0.8 | -1.3 | -0.9 | -0.9 | -0.4 | 0.0 |
| Overweight (25 <= BMI < 30) | 41.9 | 0.2 | 0.6 | 0.0 | -0.1 | 0.4 | -0.2 | 0.1 | 0.9 | 1.3 | 0.8 | 1.1 | 0.2 | -0.2 |
| Obesity (BMI >= 30) | 22.6 | -1.9 | -1.5 | 0.0 | -2.0 | -1.7 | 0.0 | 0.1 | -2.5 | -2.5 | -0.8 | -0.7 | -0.2 | 0.0 |
| Self-rated health |  |  |  |  |  |  |  |  |  |  |  |  |  |  |
| Very good/good/fair | 93.5 | 0.3 | 0.7 | 0.0 | 1.6 | 2.0 | 1.3 | 0.2 | 2.2 | 2.6 | 1.7 | 0.7 | 0.7 | 1.1 |
| Bad/very bad | 6.5 | -0.3 | -0.7 | 0.0 | -1.6 | -2.0 | -1.3 | -0.2 | -2.2 | -2.6 | -1.7 | -0.7 | -0.7 | -1.1 |
| Self-rated mental health |  |  |  |  |  |  |  |  |  |  |  |  |  |  |
| Excellent/very good/good | 81.4 | 1.6 | 1.8 | 0.0 | 2.0 | 2.1 | 0.1 | -0.2 | 2.9 | 3.1 | 0.7 | 0.4 | -0.3 | -0.3 |
| fair/poor | 18.6 | -1.6 | -1.8 | 0.0 | -2.0 | -2.1 | -0.1 | 0.2 | -2.9 | -3.1 | -0.7 | -0.4 | 0.3 | 0.3 |
| Paying attention to health |  |  |  |  |  |  |  |  |  |  |  |  |  |  |
| Not at all/less strong/moderate | 56.1 | -1.4 | -1.3 | 0.0 | -1.9 | -1.9 | -0.4 | 0.1 | -3.4 | -3.1 | -1.7 | -1.2 | -0.1 | -0.1 |
| Strong/very strong | 43.9 | 1.4 | 1.3 | 0.0 | 1.9 | 1.9 | 0.4 | -0.1 | 3.4 | 3.1 | 1.7 | 1.2 | 0.1 | 0.1 |
| Satisfaction: Life in general |  |  |  |  |  |  |  |  |  |  |  |  |  |  |
| Scale value 1 to 3 | 8.6 | -1.8 | -1.6 | 0.0 | -2.2 | -2.0 | -0.3 | 0.0 | -3.6 | -3.4 | -1.5 | -1.3 | -0.3 | 0.1 |
| Scale value 4 to 7 | 44.2 | -1.4 | -1.6 | 0.0 | -2.0 | -2.3 | -0.7 | -0.1 | -3.4 | -3.5 | -1.9 | -1.3 | -0.1 | -0.1 |
| Scale value 8 to 10 | 47.2 | 2.3 | 2.5 | 0.0 | 3.2 | 3.3 | 0.8 | 0.1 | 5.3 | 5.3 | 2.7 | 1.9 | 0.3 | 0.1 |
| Red meat |  |  |  |  |  |  |  |  |  |  |  |  |  |  |
| Never | 5.8 | 0.5 | -0.2 | 0.0 | 0.8 | 0.1 | 0.3 | 0.1 | 0.6 | -0.3 | 0.0 | -0.3 | -0.5 | 0.0 |
| Daily or several times a day | 4.4 | -1.7 | -1.0 | 0.0 | -2.4 | -1.6 | -0.5 | -0.1 | -3.8 | -3.0 | -2.3 | -2.0 | -0.6 | -0.5 |
| 4 to 6 times per week | 17.3 | -1.5 | -0.3 | 0.0 | -1.6 | -0.4 | 0.1 | 0.0 | -3.1 | -1.7 | -1.0 | -1.0 | -0.4 | 0.0 |
| 1 to 3 times per week | 49.9 | 0.5 | 0.6 | 0.0 | 0.3 | 0.4 | -0.2 | 0.0 | 1.2 | 1.1 | 0.4 | 0.5 | 0.3 | 0.1 |
| Less than once per week | 22.6 | 1.3 | 0.2 | 0.0 | 1.7 | 0.6 | 0.2 | 0.0 | 2.7 | 1.7 | 1.5 | 1.4 | 0.6 | 0.2 |
| Sausage products |  |  |  |  |  |  |  |  |  |  |  |  |  |  |
| Never | 7.2 | -0.1 | -0.3 | 0.0 | 0.2 | -0.1 | 0.1 | -0.1 | -0.9 | -1.4 | -1.3 | -1.6 | -0.8 | -0.6 |
| Daily or several times a day | 13.8 | -0.3 | -0.7 | 0.0 | -0.9 | -1.1 | -0.4 | 0.0 | -1.3 | -1.8 | -0.8 | -0.3 | 0.0 | -0.2 |
| 4 to 6 times per week | 25.1 | -0.3 | 0.1 | 0.0 | 0.1 | 0.4 | 0.3 | 0.0 | 0.8 | 1.2 | 1.0 | 0.8 | 0.4 | 0.3 |
| 1 to 3 times per week | 35.4 | 0.0 | 0.3 | 0.0 | -0.1 | 0.1 | 0.0 | 0.0 | 0.2 | 0.5 | 0.3 | 0.3 | -0.2 | -0.2 |
| Less than once per week | 18.4 | 0.7 | 0.3 | 0.0 | 0.8 | 0.4 | 0.0 | 0.0 | 0.6 | 0.5 | 0.0 | 0.0 | 0.3 | 0.4 |
| Smoking |  |  |  |  |  |  |  |  |  |  |  |  |  |  |
| Non-smoker | 72.8 | 4.4 | 3.9 | 0.0 | 5.4 | 5.0 | 1.1 | 0.4 | 9.0 | 8.4 | 5.0 | 4.4 | 0.9 | 0.8 |
| Daily smoking | 20.4 | -4.7 | -4.1 | 0.0 | -5.7 | -5.2 | -1.1 | -0.4 | -8.9 | -8.2 | -4.7 | -4.1 | -1.1 | -1.1 |
| Occasional smoking | 6.8 | -0.3 | -0.4 | 0.0 | -0.5 | -0.6 | -0.3 | -0.1 | -1.9 | -1.9 | -1.4 | -1.2 | 0.2 | 0.3 |
| Chronic diseases |  |  |  |  |  |  |  |  |  |  |  |  |  |  |
| No | 48.4 | -0.6 | -0.1 | 0.0 | -0.8 | -0.4 | -0.5 | -0.2 | -3.3 | -2.9 | -2.8 | -2.5 | -0.6 | 0.0 |
| Yes | 51.6 | 0.6 | 0.1 | 0.0 | 0.8 | 0.4 | 0.5 | 0.2 | 3.3 | 2.9 | 2.8 | 2.5 | 0.6 | 0.0 |
| Sport |  |  |  |  |  |  |  |  |  |  |  |  |  |  |
| No sporting activities | 23.3 | -1.8 | -2.4 | 0.0 | -3.3 | -4.0 | -1.7 | 0.0 | -3.9 | -4.9 | -2.7 | -1.0 | -0.3 | -0.4 |
| Less than 1 hour per week | 19.3 | -1.0 | -0.8 | 0.0 | -1.3 | -1.1 | -0.2 | 0.0 | -1.8 | -1.3 | -0.6 | -0.4 | -0.2 | -0.1 |
| 1 to less than 2 hours per week | 24.1 | 0.5 | 0.6 | 0.0 | 0.7 | 0.8 | 0.3 | 0.1 | 2.0 | 2.1 | 1.7 | 1.5 | 0.6 | 0.4 |
| 2 to less than 4 hours per week | 18.8 | 1.6 | 1.7 | 0.0 | 2.4 | 2.5 | 0.8 | 0.0 | 2.8 | 3.0 | 1.2 | 0.4 | -0.1 | 0.0 |
| 4 hours per week and more | 14.6 | 0.8 | 1.1 | 0.0 | 1.7 | 1.9 | 1.0 | 0.0 | 0.9 | 1.1 | 0.4 | -0.7 | -0.1 | 0.1 |
| KWsubj\_k |  |  |  |  |  |  |  |  |  |  |  |  |  |  |
| Scale value 1 to 3 | 40.0 | 0.1 | 0.0 | 0.0 | -0.3 | -0.4 | -0.4 | -0.2 | -0.8 | -0.9 | -1.2 | -1.0 | -0.4 | -0.6 |
| Scale value 4 to 7 | 44.6 | -0.1 | -0.1 | 0.0 | 0.0 | 0.0 | 0.2 | 0.1 | 0.6 | 0.6 | 0.9 | 0.9 | 0.3 | 0.4 |
| Scale value 8 to 10 | 15.4 | -0.1 | 0.1 | 0.0 | 0.5 | 0.6 | 0.3 | 0.1 | 0.2 | 0.4 | 0.4 | 0.1 | 0.2 | 0.3 |
| IAtermin |  |  |  |  |  |  |  |  |  |  |  |  |  |  |
| No | 55.6 | 2.0 | 1.1 | 0.0 | 2.0 | 1.2 | -0.1 | 0.1 | 4.2 | 3.6 | 2.3 | 2.4 | 0.4 | 0.1 |
| Yes | 30.9 | -1.4 | -0.9 | 0.0 | -1.6 | -1.1 | -0.1 | -0.1 | -2.9 | -2.7 | -1.6 | -1.6 | -0.5 | -0.3 |
| No need for examination or treatment | 13.5 | -1.0 | -0.4 | 0.0 | -0.7 | -0.2 | 0.2 | 0.0 | -2.2 | -1.5 | -1.1 | -1.4 | 0.1 | 0.2 |
|  |  |  |  |  |  |  |  |  |  |  |  |  |  |  |
| --- | --- | --- | --- | --- | --- | --- | --- | --- | --- | --- | --- | --- | --- | --- |
| aCS = Cross-Sectional | | | | | | | | | | | | | | |

# Questionnaire B

## Female and male

Table 2.1: Standardized differences in percentage points compared to reference values for the recruitment survey, registration sample, and the sample of participants in Questionnaire B; calculated unweighted and weighted according to the respective weighting factors.

|  |  | Recruitment Survey | | | Registration | | | | Participation Questionnaire B | | | | | |
| --- | --- | --- | --- | --- | --- | --- | --- | --- | --- | --- | --- | --- | --- | --- |
| Parameter | Reference [%] | Unweighted | Design weight | CSa weight | Unweighted | Design weight | CSa weight | Drop-out Weight | Unweighted | Design weight | CSa weight (Recruitment Study) | Drop-out weight (Registration) | Drop-out weight | CSa weight |
| sex |  |  |  |  |  |  |  |  |  |  |  |  |  |  |
| Male | 48.9 | -2.0 | -1.9 | 0.0 | -2.2 | -2.1 | 0.0 | 0.0 | -3.7 | -3.6 | -1.9 | -2.0 | -0.5 | 0.0 |
| Female | 51.1 | 2.0 | 1.9 | 0.0 | 2.2 | 2.1 | 0.0 | 0.0 | 3.7 | 3.6 | 1.9 | 2.0 | 0.5 | 0.0 |
| Agegrp |  |  |  |  |  |  |  |  |  |  |  |  |  |  |
| 18-29 yrs | 16.0 | 0.6 | -0.3 | -0.1 | 1.7 | 0.7 | 0.9 | -0.3 | -2.7 | -3.3 | -3.4 | -4.5 | -0.6 | 0.0 |
| 30-39 yrs | 15.8 | -0.9 | -0.1 | 0.0 | 0.3 | 1.1 | 1.4 | 0.1 | -1.7 | -0.8 | -0.8 | -2.2 | 0.0 | 0.2 |
| 40-49 yrs | 14.7 | -3.8 | -0.1 | 0.1 | -3.3 | 0.4 | 0.7 | 0.1 | -4.2 | -0.4 | -0.2 | -1.0 | -0.1 | -0.3 |
| 50-59 yrs | 17.6 | 0.4 | 0.6 | 0.4 | 0.8 | 0.9 | 0.7 | 0.4 | 2.0 | 2.1 | 2.1 | 1.6 | 0.6 | 0.5 |
| 60-69 yrs | 16.5 | -0.1 | -0.1 | -0.1 | -0.1 | -0.2 | -0.2 | 0.0 | 2.5 | 2.3 | 2.7 | 2.8 | 0.1 | -0.1 |
| 70-79 yrs | 10.7 | 3.5 | 1.7 | -0.2 | 2.4 | 0.4 | -1.4 | -0.1 | 5.0 | 2.9 | 1.2 | 2.5 | 0.0 | -0.2 |
| 80+ yrs | 8.7 | 0.6 | -1.8 | -0.1 | -2.3 | -4.6 | -3.2 | -0.1 | -1.3 | -3.7 | -2.2 | 0.9 | 0.1 | -0.3 |
| Federal state |  |  |  |  |  |  |  |  |  |  |  |  |  |  |
| Schleswig-Holstein | 3.5 | 12.5 | 0.0 | 0.0 | 12.6 | 0.1 | 0.2 | 0.1 | 12.8 | 0.1 | 0.2 | 0.1 | 0.2 | 0.0 |
| Hamburg | 2.2 | 3.9 | -0.1 | 0.0 | 4.3 | 0.3 | 0.4 | 0.2 | 4.0 | 0.0 | 0.1 | -0.2 | 0.1 | 0.0 |
| Lower Saxony | 9.6 | -3.8 | 0.1 | 0.1 | -3.9 | 0.0 | 0.0 | -0.1 | -3.6 | 0.2 | 0.4 | 0.3 | 0.1 | 0.0 |
| Bremen | 0.8 | 8.3 | -0.1 | 0.0 | 8.6 | 0.1 | 0.1 | 0.1 | 8.6 | 0.0 | -0.1 | -0.2 | 0.1 | 0.0 |
| North Rhine-Westphalia | 21.4 | -7.2 | 0.0 | 0.0 | -7.2 | 0.2 | 0.1 | -0.2 | -7.3 | 0.1 | 0.0 | -0.3 | -0.2 | 0.0 |
| Hesse | 7.6 | -4.4 | 0.0 | 0.0 | -4.5 | -0.2 | -0.2 | -0.2 | -5.4 | -1.3 | -1.4 | -1.4 | -1.1 | 0.0 |
| Rhineland-Palatinate | 4.9 | -3.5 | 0.0 | 0.0 | -3.2 | 0.5 | 0.5 | 0.5 | -2.9 | 0.7 | 1.0 | 1.0 | 0.9 | 0.0 |
| Baden-Württemberg | 13.3 | -5.9 | -0.1 | 0.0 | -5.6 | 0.1 | 0.1 | -0.1 | -5.8 | -0.2 | -0.5 | -0.6 | -1.0 | 0.0 |
| Bavaria | 15.9 | -5.7 | 0.0 | 0.0 | -5.8 | -0.1 | -0.2 | 0.0 | -5.0 | 0.9 | 0.8 | 0.9 | 0.6 | 0.1 |
| Saarland | 1.2 | 7.1 | 0.0 | 0.0 | 7.0 | 0.0 | 0.0 | 0.1 | 7.0 | 0.0 | -0.1 | 0.0 | 0.0 | 0.0 |
| Berlin | 4.5 | 10.5 | 0.0 | 0.0 | 10.9 | 0.3 | 0.4 | 0.3 | 10.4 | -0.2 | -0.1 | -0.3 | 0.5 | 0.0 |
| Brandenburg | 3.1 | 0.8 | 0.0 | -0.1 | 0.5 | -0.2 | -0.2 | -0.1 | 0.3 | -0.5 | -0.4 | -0.3 | -0.1 | 0.0 |
| Mecklenburg-Western Pomerania | 2.0 | 4.0 | 0.0 | 0.0 | 3.5 | -0.3 | -0.3 | -0.2 | 3.5 | -0.4 | -0.3 | -0.2 | -0.1 | 0.0 |
| Saxony | 4.9 | -2.6 | 0.0 | 0.0 | -2.9 | -0.4 | -0.3 | 0.0 | -2.5 | 0.3 | 0.3 | 0.7 | 0.4 | 0.0 |
| Saxony-Anhalt | 2.6 | 1.8 | 0.0 | 0.0 | 1.4 | -0.3 | -0.2 | 0.2 | 1.4 | -0.3 | -0.2 | 0.3 | 0.1 | 0.0 |
| Thuringia | 2.5 | 2.0 | 0.0 | 0.0 | 1.4 | -0.5 | -0.5 | -0.1 | 1.6 | -0.4 | -0.3 | 0.1 | -0.1 | -0.1 |
| German |  |  |  |  |  |  |  |  |  |  |  |  |  |  |
| Yes | 85.0 | 14.2 | 13.6 | 12.5 | 16.1 | 15.7 | 15.0 | 12.6 | 19.1 | 18.6 | 18.4 | 16.7 | 13.9 | 14.2 |
| No | 15.0 | -14.2 | -13.6 | -12.5 | -16.1 | -15.7 | -15.0 | -12.6 | -19.1 | -18.6 | -18.4 | -16.7 | -13.9 | -14.2 |
| BIK |  |  |  |  |  |  |  |  |  |  |  |  |  |  |
| BIK 1 | 10.8 | -0.8 | -0.4 | 0.0 | -1.4 | -0.9 | -0.5 | 0.0 | -1.7 | -1.2 | -0.8 | -0.2 | -0.1 | 0.0 |
| BIK 2 | 34.6 | -1.2 | 1.1 | 0.0 | -1.8 | 0.6 | -0.6 | 0.1 | -1.2 | 1.2 | 0.0 | 0.8 | 0.2 | 0.0 |
| BIK 3 | 26.8 | -2.4 | -0.6 | 0.0 | -2.2 | -0.4 | 0.2 | -0.1 | -2.1 | -0.3 | 0.4 | 0.0 | -0.2 | 0.0 |
| BIK 4 | 27.9 | 4.1 | -0.3 | 0.0 | 4.9 | 0.3 | 0.7 | 0.0 | 4.4 | -0.1 | 0.1 | -0.7 | 0.0 | 0.0 |
| Education |  |  |  |  |  |  |  |  |  |  |  |  |  |  |
| Low | 34.4 | -12.8 | -12.8 | -1.3 | -16.8 | -16.8 | -5.8 | -1.5 | -17.8 | -17.9 | -7.5 | -3.4 | -3.0 | -1.3 |
| Medium | 45.3 | 2.3 | 2.5 | 1.4 | 3.3 | 3.5 | 3.4 | 1.5 | 3.0 | 3.3 | 3.9 | 2.1 | 2.4 | 1.1 |
| High | 20.3 | 10.7 | 10.5 | -0.1 | 13.2 | 12.9 | 2.3 | 0.0 | 14.3 | 14.0 | 3.7 | 1.4 | 0.4 | 0.1 |
| Household size |  |  |  |  |  |  |  |  |  |  |  |  |  |  |
| Single-person household | 25.4 | -3.5 | -4.8 | 0.2 | -4.6 | -5.8 | -0.9 | 0.1 | -4.7 | -6.1 | -1.2 | -0.2 | 0.0 | 0.5 |
| Multi-person household | 74.6 | 3.5 | 4.8 | -0.2 | 4.6 | 5.8 | 0.9 | -0.1 | 4.7 | 6.1 | 1.2 | 0.2 | 0.0 | -0.5 |
| PAbmi\_k2 |  |  |  |  |  |  |  |  |  |  |  |  |  |  |
| Normal weight (18.5 <= BMI < 25) | 40.7 | 2.0 | 1.9 | 0.0 | 2.5 | 2.5 | 0.4 | -0.1 | 2.7 | 2.8 | 1.0 | 0.5 | 0.3 | 0.2 |
| Underweight (BMI < 18.5) | 2.0 | 0.2 | -0.1 | 0.0 | 0.2 | -0.1 | -0.1 | -0.1 | -0.1 | -0.5 | -0.7 | -0.7 | -0.1 | 0.0 |
| Overweight (25 <= BMI < 30) | 35.2 | -0.3 | -0.3 | 0.0 | -0.7 | -0.7 | -0.4 | 0.1 | -0.5 | -0.4 | -0.1 | 0.3 | -0.1 | -0.1 |
| Obesity (BMI >= 30) | 22.1 | -2.2 | -1.9 | 0.0 | -2.3 | -2.1 | -0.1 | 0.0 | -2.7 | -2.7 | -0.8 | -0.7 | -0.1 | 0.0 |
| Self-rated health |  |  |  |  |  |  |  |  |  |  |  |  |  |  |
| Very good/good/fair | 93.1 | 0.9 | 1.3 | 0.0 | 2.4 | 2.7 | 1.5 | 0.0 | 2.8 | 3.1 | 1.8 | 0.5 | 0.1 | 0.4 |
| Bad/very bad | 6.9 | -0.9 | -1.3 | 0.0 | -2.4 | -2.7 | -1.5 | 0.0 | -2.8 | -3.1 | -1.8 | -0.5 | -0.1 | -0.4 |
| Self-rated mental health |  |  |  |  |  |  |  |  |  |  |  |  |  |  |
| Excellent/very good/good | 78.5 | 1.3 | 1.6 | 0.0 | 1.7 | 1.9 | 0.3 | 0.0 | 2.6 | 3.0 | 1.5 | 1.2 | 0.3 | 0.2 |
| fair/poor | 21.5 | -1.3 | -1.6 | 0.0 | -1.7 | -1.9 | -0.3 | 0.0 | -2.6 | -3.0 | -1.5 | -1.2 | -0.3 | -0.2 |
| Paying attention to health |  |  |  |  |  |  |  |  |  |  |  |  |  |  |
| Not at all/less strong/moderate | 51.6 | -1.7 | -1.6 | 0.0 | -2.2 | -2.2 | -0.5 | 0.0 | -3.6 | -3.3 | -2.0 | -1.5 | -0.2 | -0.1 |
| Strong/very strong | 48.4 | 1.7 | 1.6 | 0.0 | 2.2 | 2.2 | 0.5 | 0.0 | 3.6 | 3.3 | 2.0 | 1.5 | 0.2 | 0.1 |
| Satisfaction: Life in general |  |  |  |  |  |  |  |  |  |  |  |  |  |  |
| Scale value 1 to 3 | 8.5 | -1.4 | -1.5 | 0.0 | -2.0 | -2.1 | -0.5 | -0.1 | -2.8 | -3.0 | -1.5 | -1.2 | -0.1 | 0.0 |
| Scale value 4 to 7 | 45.1 | -0.9 | -1.0 | 0.0 | -1.3 | -1.4 | -0.5 | 0.1 | -2.4 | -2.3 | -1.5 | -1.0 | 0.2 | 0.3 |
| Scale value 8 to 10 | 46.4 | 1.7 | 1.8 | 0.0 | 2.4 | 2.5 | 0.7 | 0.0 | 3.9 | 3.8 | 2.4 | 1.6 | -0.1 | -0.2 |
| Red meat |  |  |  |  |  |  |  |  |  |  |  |  |  |  |
| Never | 9.3 | 1.2 | 0.6 | 0.0 | 1.7 | 1.0 | 0.4 | 0.0 | 1.7 | 1.2 | 0.5 | 0.2 | 0.0 | 0.1 |
| Daily or several times a day | 2.8 | -1.6 | -1.1 | 0.0 | -2.1 | -1.7 | -0.4 | 0.0 | -3.5 | -3.1 | -2.0 | -1.7 | -0.6 | -0.3 |
| 4 to 6 times per week | 12.2 | -1.4 | -0.4 | 0.0 | -1.6 | -0.5 | 0.1 | 0.0 | -2.5 | -1.6 | -1.1 | -1.1 | -0.2 | 0.0 |
| 1 to 3 times per week | 46.1 | -0.6 | -0.1 | 0.0 | -1.1 | -0.5 | -0.4 | 0.0 | -0.7 | -0.2 | -0.1 | 0.3 | 0.1 | 0.1 |
| Less than once per week | 29.6 | 1.4 | 0.4 | 0.0 | 1.9 | 0.9 | 0.3 | 0.0 | 2.5 | 1.6 | 1.2 | 0.9 | 0.2 | -0.1 |
| Sausage products |  |  |  |  |  |  |  |  |  |  |  |  |  |  |
| Never | 10.3 | 1.5 | 1.1 | 0.0 | 2.1 | 1.6 | 0.4 | -0.1 | 1.7 | 1.3 | 0.0 | -0.6 | -0.2 | -0.3 |
| Daily or several times a day | 10.0 | -0.7 | -1.0 | 0.0 | -1.2 | -1.4 | -0.3 | 0.0 | -1.6 | -1.8 | -1.1 | -0.9 | -0.5 | -0.4 |
| 4 to 6 times per week | 19.4 | -0.6 | -0.2 | 0.0 | -0.3 | 0.1 | 0.4 | 0.0 | 0.0 | 0.3 | 0.4 | 0.2 | 0.1 | 0.2 |
| 1 to 3 times per week | 35.6 | -0.7 | -0.3 | 0.0 | -1.3 | -0.8 | -0.4 | 0.0 | -1.3 | -0.7 | -0.2 | 0.4 | 0.1 | 0.0 |
| Less than once per week | 24.8 | 0.8 | 0.4 | 0.0 | 1.0 | 0.6 | 0.1 | 0.1 | 1.3 | 0.8 | 0.6 | 0.5 | 0.3 | 0.3 |
| Smoking |  |  |  |  |  |  |  |  |  |  |  |  |  |  |
| Non-smoker | 76.2 | 3.5 | 3.2 | 0.0 | 4.1 | 3.8 | 0.5 | 0.0 | 7.0 | 6.6 | 4.0 | 3.6 | 0.7 | 0.5 |
| Daily smoking | 17.9 | -3.9 | -3.5 | 0.0 | -4.5 | -4.1 | -0.5 | -0.1 | -7.1 | -6.6 | -3.9 | -3.5 | -0.9 | -0.7 |
| Occasional smoking | 5.9 | -0.1 | -0.1 | 0.0 | -0.1 | -0.2 | -0.1 | 0.0 | -1.3 | -1.3 | -1.1 | -0.9 | 0.1 | 0.3 |
| Chronic diseases |  |  |  |  |  |  |  |  |  |  |  |  |  |  |
| No | 45.0 | -0.1 | 0.7 | 0.0 | -0.3 | 0.4 | -0.4 | -0.1 | -2.1 | -1.1 | -2.0 | -1.7 | -0.4 | -0.2 |
| Yes | 55.0 | 0.1 | -0.7 | 0.0 | 0.3 | -0.4 | 0.4 | 0.1 | 2.1 | 1.1 | 2.0 | 1.7 | 0.4 | 0.2 |
| Sport |  |  |  |  |  |  |  |  |  |  |  |  |  |  |
| No sporting activities | 23.0 | -2.6 | -3.1 | 0.0 | -4.7 | -5.2 | -2.3 | -0.1 | -5.9 | -6.4 | -4.1 | -2.1 | -0.9 | -0.9 |
| Less than 1 hour per week | 19.7 | -0.9 | -0.5 | 0.0 | -1.0 | -0.7 | -0.1 | 0.0 | -1.4 | -1.1 | -0.5 | -0.4 | 0.4 | 0.4 |
| 1 to less than 2 hours per week | 26.5 | 1.0 | 1.0 | 0.0 | 1.4 | 1.4 | 0.5 | 0.1 | 2.4 | 2.3 | 1.9 | 1.6 | 0.6 | 0.5 |
| 2 to less than 4 hours per week | 18.9 | 1.7 | 1.8 | 0.0 | 2.8 | 3.0 | 1.2 | 0.0 | 3.8 | 3.9 | 2.2 | 1.1 | 0.2 | 0.0 |
| 4 hours per week and more | 11.9 | 0.8 | 0.9 | 0.0 | 1.6 | 1.8 | 0.9 | 0.0 | 1.0 | 1.3 | 0.5 | -0.5 | -0.3 | 0.0 |
| KWsubj\_k |  |  |  |  |  |  |  |  |  |  |  |  |  |  |
| Scale value 1 to 3 | 34.8 | -0.3 | -0.4 | 0.0 | -0.8 | -0.9 | -0.4 | 0.0 | -0.8 | -1.0 | -0.6 | -0.2 | 0.0 | -0.1 |
| Scale value 4 to 7 | 49.0 | 0.1 | 0.1 | 0.0 | 0.1 | 0.2 | 0.2 | 0.0 | 0.3 | 0.2 | 0.3 | 0.3 | 0.1 | 0.2 |
| Scale value 8 to 10 | 16.1 | 0.3 | 0.4 | 0.0 | 0.8 | 0.8 | 0.3 | -0.1 | 0.7 | 0.9 | 0.4 | -0.1 | -0.1 | -0.1 |
| IAtermin |  |  |  |  |  |  |  |  |  |  |  |  |  |  |
| No | 54.8 | 0.9 | 0.3 | 0.0 | 0.6 | -0.1 | -0.5 | 0.0 | 2.3 | 1.5 | 1.1 | 1.5 | 0.0 | -0.1 |
| Yes | 33.5 | -0.7 | -0.3 | 0.0 | -0.5 | -0.2 | 0.3 | 0.0 | -1.8 | -1.2 | -0.9 | -1.2 | -0.2 | -0.2 |
| No need for examination or treatment | 11.7 | -0.4 | 0.1 | 0.0 | -0.1 | 0.3 | 0.3 | 0.0 | -0.9 | -0.5 | -0.4 | -0.7 | 0.3 | 0.5 |
|  |  |  |  |  |  |  |  |  |  |  |  |  |  |  |
| --- | --- | --- | --- | --- | --- | --- | --- | --- | --- | --- | --- | --- | --- | --- |
| aCS = Cross-Sectional | | | | | | | | | | | | | | |

## Female

Table 2.2: Standardized differences in percentage points compared to reference values for the recruitment survey, registration sample, and the sample of participants in Questionnaire B; calculated unweighted and weighted according to the respective weighting factors.

|  |  | Recruitment Survey | | | Registration | | | | Participation Questionnaire B | | | | | |
| --- | --- | --- | --- | --- | --- | --- | --- | --- | --- | --- | --- | --- | --- | --- |
| Parameter | Reference [%] | Unweighted | Design weight | CSa weight | Unweighted | Design weight | CSa weight | Drop-out Weight | Unweighted | Design weight | CSa weight (Recruitment Study) | Drop-out weight (Registration) | Drop-out weight | CSa weight |
| Agegrp |  |  |  |  |  |  |  |  |  |  |  |  |  |  |
| 18-29 yrs | 14.9 | 2.2 | 1.3 | -0.1 | 3.9 | 2.9 | 1.7 | 0.1 | 0.3 | -0.5 | -1.6 | -3.0 | 0.5 | 0.0 |
| 30-39 yrs | 15.1 | 0.2 | 0.9 | 0.2 | 1.5 | 2.3 | 1.9 | 0.1 | -0.2 | 0.7 | -0.3 | -2.0 | -0.3 | 0.3 |
| 40-49 yrs | 14.4 | -2.9 | 1.0 | 0.3 | -2.4 | 1.6 | 0.9 | 0.1 | -2.7 | 1.2 | 0.5 | -0.6 | 0.3 | -0.2 |
| 50-59 yrs | 17.2 | 1.3 | 1.4 | 0.7 | 1.9 | 1.9 | 1.3 | 0.8 | 2.8 | 2.8 | 2.3 | 1.6 | 0.6 | 0.5 |
| 60-69 yrs | 16.6 | -0.6 | -0.7 | 0.2 | -0.7 | -1.0 | 0.0 | 0.1 | 1.6 | 1.3 | 2.3 | 2.4 | 0.0 | -0.2 |
| 70-79 yrs | 11.4 | 1.5 | -0.4 | 0.0 | 0.1 | -1.9 | -1.5 | 0.3 | 2.5 | 0.2 | 0.8 | 2.7 | 0.5 | -0.1 |
| 80+ yrs | 10.4 | -2.2 | -4.6 | -1.5 | -6.0 | -8.2 | -5.8 | -1.6 | -5.6 | -7.7 | -5.5 | -1.6 | -1.8 | -0.4 |
| Federal state |  |  |  |  |  |  |  |  |  |  |  |  |  |  |
| Schleswig-Holstein | 3.5 | 12.7 | 0.1 | 0.0 | 12.9 | 0.3 | 0.2 | 0.0 | 13.3 | 0.5 | 0.4 | 0.2 | 0.0 | 0.0 |
| Hamburg | 2.3 | 4.4 | 0.3 | 0.0 | 4.8 | 0.7 | 0.3 | 0.1 | 4.6 | 0.6 | 0.2 | -0.2 | 0.1 | 0.0 |
| Lower Saxony | 9.6 | -3.9 | 0.0 | 0.1 | -3.9 | 0.1 | 0.2 | 0.0 | -3.6 | 0.4 | 0.5 | 0.3 | 0.0 | -0.1 |
| Bremen | 0.8 | 8.4 | 0.0 | -0.1 | 8.7 | 0.2 | 0.2 | 0.1 | 8.7 | 0.1 | 0.1 | -0.1 | -0.1 | 0.0 |
| North Rhine-Westphalia | 21.5 | -7.1 | 0.2 | 0.0 | -7.2 | 0.4 | 0.0 | -0.2 | -7.2 | 0.4 | -0.2 | -0.5 | 0.1 | 0.0 |
| Hesse | 7.6 | -4.8 | -0.4 | 0.0 | -5.4 | -1.0 | -0.5 | -0.4 | -6.4 | -2.4 | -2.0 | -2.0 | -1.5 | -0.1 |
| Rhineland-Palatinate | 4.9 | -3.8 | -0.3 | -0.1 | -3.4 | 0.2 | 0.5 | 0.6 | -3.5 | 0.0 | 0.3 | 0.5 | 0.8 | 0.0 |
| Baden-Württemberg | 13.2 | -5.8 | 0.1 | 0.0 | -5.7 | 0.1 | -0.1 | -0.2 | -5.9 | -0.1 | -0.4 | -0.4 | -0.9 | 0.0 |
| Bavaria | 15.8 | -5.6 | 0.1 | -0.1 | -5.6 | 0.2 | 0.0 | 0.1 | -4.9 | 1.2 | 1.0 | 1.2 | 0.9 | 0.1 |
| Saarland | 1.2 | 6.9 | 0.0 | 0.0 | 6.7 | -0.1 | -0.1 | 0.0 | 6.5 | -0.3 | -0.3 | -0.1 | 0.1 | 0.0 |
| Berlin | 4.5 | 10.2 | -0.2 | 0.0 | 10.8 | 0.3 | 0.6 | 0.3 | 10.3 | -0.2 | -0.1 | -0.4 | 0.2 | 0.0 |
| Brandenburg | 3.1 | 0.8 | 0.1 | -0.1 | 0.6 | -0.2 | -0.3 | -0.2 | 0.4 | -0.4 | -0.4 | -0.3 | -0.2 | 0.0 |
| Mecklenburg-Western Pomerania | 2.0 | 4.2 | 0.2 | 0.0 | 3.9 | 0.0 | -0.1 | 0.0 | 4.0 | 0.0 | -0.1 | -0.1 | -0.1 | 0.0 |
| Saxony | 4.9 | -2.5 | 0.0 | 0.1 | -2.8 | -0.5 | -0.4 | 0.0 | -2.3 | 0.1 | 0.6 | 0.8 | 0.3 | 0.1 |
| Saxony-Anhalt | 2.6 | 1.5 | -0.2 | 0.0 | 1.3 | -0.4 | -0.1 | 0.3 | 1.5 | -0.2 | 0.2 | 0.7 | 0.4 | 0.1 |
| Thuringia | 2.5 | 1.7 | -0.1 | 0.0 | 1.1 | -0.6 | -0.4 | 0.0 | 1.2 | -0.5 | -0.2 | 0.3 | 0.0 | -0.1 |
| German |  |  |  |  |  |  |  |  |  |  |  |  |  |  |
| Yes | 85.8 | 12.7 | 12.0 | 11.1 | 14.5 | 14.1 | 13.4 | 11.1 | 17.3 | 16.9 | 16.7 | 15.0 | 12.0 | 12.2 |
| No | 14.2 | -12.7 | -12.0 | -11.1 | -14.5 | -14.1 | -13.4 | -11.1 | -17.3 | -16.9 | -16.7 | -15.0 | -12.0 | -12.2 |
| BIK |  |  |  |  |  |  |  |  |  |  |  |  |  |  |
| BIK 1 | 10.7 | -0.8 | -0.4 | 0.0 | -1.4 | -0.8 | -0.4 | 0.2 | -2.0 | -1.5 | -1.1 | -0.6 | -0.2 | -0.1 |
| BIK 2 | 34.4 | -1.3 | 0.9 | 0.1 | -1.9 | 0.5 | -0.4 | 0.2 | -1.4 | 1.1 | 0.3 | 1.1 | 0.5 | 0.1 |
| BIK 3 | 26.9 | -2.3 | -0.3 | 0.2 | -2.2 | -0.2 | 0.5 | 0.1 | -1.9 | 0.0 | 0.8 | 0.5 | 0.2 | 0.4 |
| BIK 4 | 28.1 | 4.1 | -0.5 | -0.3 | 5.0 | 0.2 | 0.2 | -0.5 | 4.5 | -0.2 | -0.4 | -1.2 | -0.6 | -0.5 |
| Education |  |  |  |  |  |  |  |  |  |  |  |  |  |  |
| Low | 33.8 | -14.0 | -14.2 | -1.2 | -18.7 | -18.9 | -6.5 | -1.7 | -19.5 | -19.6 | -8.0 | -3.4 | -3.0 | -0.5 |
| Medium | 47.6 | 3.4 | 3.6 | 1.4 | 4.8 | 5.1 | 4.2 | 1.7 | 4.9 | 5.2 | 4.9 | 2.6 | 2.5 | 0.6 |
| High | 18.6 | 10.8 | 10.7 | -0.2 | 13.3 | 13.1 | 2.2 | -0.1 | 13.9 | 13.6 | 3.0 | 0.7 | 0.5 | -0.2 |
| Household size |  |  |  |  |  |  |  |  |  |  |  |  |  |  |
| Single-person household | 26.0 | -1.9 | -3.6 | 0.2 | -3.6 | -5.1 | -1.6 | 0.1 | -3.3 | -4.8 | -1.2 | 0.5 | 0.0 | 0.9 |
| Multi-person household | 74.0 | 1.9 | 3.6 | -0.2 | 3.6 | 5.1 | 1.6 | -0.1 | 3.3 | 4.8 | 1.2 | -0.5 | 0.0 | -0.9 |
| PAbmi\_k2 |  |  |  |  |  |  |  |  |  |  |  |  |  |  |
| Normal weight (18.5 <= BMI < 25) | 46.8 | 2.1 | 2.4 | 0.0 | 2.6 | 3.1 | 0.7 | 0.0 | 2.6 | 3.2 | 1.0 | 0.3 | 0.1 | -0.2 |
| Underweight (BMI < 18.5) | 2.9 | 0.2 | -0.1 | 0.0 | 0.2 | -0.1 | 0.1 | 0.0 | 0.1 | -0.3 | -0.3 | -0.3 | 0.3 | 0.6 |
| Overweight (25 <= BMI < 30) | 28.7 | -0.3 | -0.7 | 0.0 | -0.7 | -1.2 | -0.6 | 0.1 | -0.7 | -1.2 | -0.6 | 0.1 | -0.4 | -0.4 |
| Obesity (BMI >= 30) | 21.6 | -2.4 | -2.2 | 0.0 | -2.6 | -2.5 | -0.2 | -0.1 | -2.5 | -2.5 | -0.4 | -0.4 | 0.2 | 0.4 |
| Self-rated health |  |  |  |  |  |  |  |  |  |  |  |  |  |  |
| Very good/good/fair | 92.8 | 1.5 | 2.0 | 0.0 | 3.2 | 3.4 | 1.7 | -0.1 | 3.8 | 3.9 | 2.2 | 0.7 | 0.0 | 0.1 |
| Bad/very bad | 7.2 | -1.5 | -2.0 | 0.0 | -3.2 | -3.4 | -1.7 | 0.1 | -3.8 | -3.9 | -2.2 | -0.7 | 0.0 | -0.1 |
| Self-rated mental health |  |  |  |  |  |  |  |  |  |  |  |  |  |  |
| Excellent/very good/good | 75.6 | 1.3 | 1.7 | 0.0 | 1.7 | 2.1 | 0.6 | 0.2 | 2.8 | 3.3 | 1.9 | 1.6 | 0.3 | 0.1 |
| fair/poor | 24.3 | -1.3 | -1.7 | 0.0 | -1.7 | -2.1 | -0.6 | -0.2 | -2.8 | -3.3 | -1.9 | -1.6 | -0.3 | -0.1 |
| Paying attention to health |  |  |  |  |  |  |  |  |  |  |  |  |  |  |
| Not at all/less strong/moderate | 47.3 | -1.6 | -1.5 | 0.0 | -2.2 | -2.2 | -0.7 | -0.1 | -3.4 | -3.4 | -2.3 | -1.8 | -0.5 | -0.4 |
| Strong/very strong | 52.7 | 1.6 | 1.5 | 0.0 | 2.2 | 2.2 | 0.7 | 0.1 | 3.4 | 3.4 | 2.3 | 1.8 | 0.5 | 0.4 |
| Satisfaction: Life in general |  |  |  |  |  |  |  |  |  |  |  |  |  |  |
| Scale value 1 to 3 | 8.4 | -1.1 | -1.4 | 0.0 | -1.8 | -2.1 | -0.7 | -0.2 | -2.7 | -2.8 | -1.8 | -1.4 | -0.4 | -0.3 |
| Scale value 4 to 7 | 46.0 | -0.6 | -0.6 | 0.0 | -0.8 | -0.7 | -0.2 | 0.2 | -1.9 | -1.7 | -1.4 | -0.9 | 0.1 | 0.3 |
| Scale value 8 to 10 | 45.7 | 1.2 | 1.4 | 0.0 | 1.8 | 1.8 | 0.6 | -0.1 | 3.3 | 3.2 | 2.4 | 1.6 | 0.1 | -0.2 |
| Red meat |  |  |  |  |  |  |  |  |  |  |  |  |  |  |
| Never | 12.6 | 1.3 | 0.8 | 0.0 | 1.9 | 1.2 | 0.5 | 0.0 | 1.8 | 1.4 | 0.7 | 0.2 | 0.0 | 0.2 |
| Daily or several times a day | 1.2 | -1.0 | -1.0 | 0.0 | -1.4 | -1.4 | -0.2 | 0.0 | -2.5 | -2.4 | -1.6 | -1.3 | -0.7 | -0.3 |
| 4 to 6 times per week | 7.3 | -0.7 | 0.1 | 0.0 | -0.8 | -0.1 | 0.1 | 0.1 | -0.9 | -0.5 | -0.2 | -0.2 | -0.1 | 0.0 |
| 1 to 3 times per week | 42.6 | -1.3 | -0.5 | 0.0 | -2.0 | -1.0 | -0.6 | -0.1 | -1.8 | -0.9 | -0.4 | 0.1 | 0.2 | 0.1 |
| Less than once per week | 36.3 | 1.0 | 0.1 | 0.0 | 1.5 | 0.5 | 0.3 | 0.0 | 1.6 | 0.7 | 0.4 | 0.1 | 0.0 | -0.1 |
| Sausage products |  |  |  |  |  |  |  |  |  |  |  |  |  |  |
| Never | 13.2 | 2.3 | 1.7 | 0.0 | 3.1 | 2.4 | 0.6 | -0.1 | 2.8 | 2.3 | 0.5 | -0.4 | -0.1 | -0.2 |
| Daily or several times a day | 6.3 | -0.7 | -0.9 | 0.0 | -0.9 | -1.2 | -0.2 | -0.1 | -1.4 | -1.6 | -0.8 | -0.8 | -0.7 | -0.6 |
| 4 to 6 times per week | 13.9 | -0.4 | 0.1 | 0.0 | -0.1 | 0.4 | 0.5 | 0.1 | 0.2 | 0.4 | 0.4 | 0.1 | -0.2 | -0.2 |
| 1 to 3 times per week | 35.8 | -1.4 | -0.9 | 0.0 | -2.3 | -1.6 | -0.9 | 0.0 | -2.2 | -1.6 | -0.8 | 0.2 | 0.3 | 0.3 |
| Less than once per week | 30.8 | 0.4 | 0.0 | 0.0 | 0.6 | 0.2 | 0.2 | 0.1 | 0.8 | 0.4 | 0.6 | 0.4 | 0.2 | 0.3 |
| Smoking |  |  |  |  |  |  |  |  |  |  |  |  |  |  |
| Non-smoker | 79.4 | 2.4 | 2.3 | 0.0 | 2.5 | 2.3 | -0.2 | -0.4 | 4.9 | 4.5 | 2.5 | 2.3 | 0.0 | -0.1 |
| Daily smoking | 15.5 | -2.9 | -2.7 | 0.0 | -3.1 | -2.8 | 0.2 | 0.3 | -5.1 | -4.6 | -2.2 | -2.1 | 0.1 | 0.2 |
| Occasional smoking | 5.1 | 0.4 | 0.3 | 0.0 | 0.4 | 0.3 | 0.1 | 0.2 | -0.7 | -0.9 | -1.0 | -0.8 | -0.1 | -0.3 |
| Chronic diseases |  |  |  |  |  |  |  |  |  |  |  |  |  |  |
| No | 41.6 | 0.7 | 1.7 | 0.0 | 0.4 | 1.4 | -0.4 | 0.0 | -0.7 | 0.3 | -1.2 | -0.8 | 0.0 | -0.2 |
| Yes | 58.4 | -0.7 | -1.7 | 0.0 | -0.4 | -1.4 | 0.4 | 0.0 | 0.7 | -0.3 | 1.2 | 0.8 | 0.0 | 0.2 |
| Sport |  |  |  |  |  |  |  |  |  |  |  |  |  |  |
| No sporting activities | 22.8 | -3.2 | -3.7 | 0.0 | -5.9 | -6.3 | -3.0 | -0.1 | -7.4 | -7.8 | -5.1 | -2.6 | -1.2 | -1.0 |
| Less than 1 hour per week | 20.1 | -0.8 | -0.2 | 0.0 | -0.8 | -0.4 | 0.1 | 0.0 | -1.2 | -0.9 | -0.5 | -0.5 | 0.5 | 0.7 |
| 1 to less than 2 hours per week | 28.7 | 1.3 | 1.1 | 0.0 | 1.8 | 1.7 | 0.7 | 0.1 | 2.6 | 2.6 | 2.0 | 1.6 | 0.6 | 0.5 |
| 2 to less than 4 hours per week | 19.0 | 1.8 | 2.0 | 0.0 | 3.1 | 3.3 | 1.5 | 0.0 | 4.1 | 4.3 | 2.7 | 1.3 | 0.3 | 0.0 |
| 4 hours per week and more | 9.3 | 1.2 | 1.1 | 0.0 | 2.0 | 2.0 | 0.9 | 0.0 | 1.8 | 1.8 | 0.9 | -0.1 | -0.3 | -0.3 |
| KWsubj\_k |  |  |  |  |  |  |  |  |  |  |  |  |  |  |
| Scale value 1 to 3 | 29.9 | -0.3 | -0.5 | 0.0 | -0.7 | -0.9 | -0.4 | 0.2 | -0.7 | -0.9 | -0.3 | 0.2 | 0.2 | 0.3 |
| Scale value 4 to 7 | 53.3 | -0.1 | 0.0 | 0.0 | -0.1 | 0.1 | 0.2 | -0.1 | -0.1 | -0.1 | 0.1 | 0.0 | -0.2 | -0.1 |
| Scale value 8 to 10 | 16.8 | 0.5 | 0.6 | 0.0 | 1.0 | 1.0 | 0.2 | -0.2 | 1.0 | 1.1 | 0.2 | -0.3 | 0.0 | -0.2 |
| IAtermin |  |  |  |  |  |  |  |  |  |  |  |  |  |  |
| No | 53.9 | 0.1 | -0.4 | 0.0 | -0.6 | -1.1 | -0.9 | -0.1 | 0.7 | 0.0 | 0.1 | 0.9 | -0.3 | -0.1 |
| Yes | 36.1 | -0.4 | -0.1 | 0.0 | 0.2 | 0.4 | 0.7 | 0.2 | -1.1 | -0.6 | -0.5 | -1.1 | 0.1 | -0.1 |
| No need for examination or treatment | 10.0 | 0.4 | 0.8 | 0.0 | 0.7 | 1.1 | 0.3 | -0.1 | 0.6 | 1.0 | 0.6 | 0.3 | 0.3 | 0.3 |
|  |  |  |  |  |  |  |  |  |  |  |  |  |  |  |
| --- | --- | --- | --- | --- | --- | --- | --- | --- | --- | --- | --- | --- | --- | --- |
| aCS = Cross-Sectional | | | | | | | | | | | | | | |

## Male

Table 2.3: Standardized differences in percentage points compared to reference values for the recruitment survey, registration sample, and the sample of participants in Questionnaire B; calculated unweighted and weighted according to the respective weighting factors.

|  |  | Recruitment Survey | | | Registration | | | | Participation Questionnaire B | | | | | |
| --- | --- | --- | --- | --- | --- | --- | --- | --- | --- | --- | --- | --- | --- | --- |
| Parameter | Reference [%] | Unweighted | Design weight | CSa weight | Unweighted | Design weight | CSa weight | Drop-out Weight | Unweighted | Design weight | CSa weight (Recruitment Study) | Drop-out weight (Registration) | Drop-out weight | CSa weight |
| Agegrp |  |  |  |  |  |  |  |  |  |  |  |  |  |  |
| 18-29 yrs | 17.1 | -1.1 | -1.9 | -0.1 | -0.7 | -1.5 | 0.0 | -0.7 | -6.1 | -6.5 | -5.3 | -6.1 | -1.8 | 0.0 |
| 30-39 yrs | 16.6 | -2.0 | -1.2 | -0.1 | -1.0 | -0.1 | 1.0 | 0.1 | -3.4 | -2.3 | -1.3 | -2.4 | 0.3 | 0.2 |
| 40-49 yrs | 15.0 | -4.8 | -1.4 | -0.1 | -4.3 | -0.9 | 0.5 | 0.1 | -5.9 | -2.2 | -1.0 | -1.5 | -0.5 | -0.3 |
| 50-59 yrs | 17.9 | -0.7 | -0.3 | 0.0 | -0.4 | -0.3 | 0.0 | 0.0 | 1.0 | 1.2 | 1.9 | 1.7 | 0.6 | 0.6 |
| 60-69 yrs | 16.4 | 0.5 | 0.4 | -0.5 | 0.6 | 0.6 | -0.4 | -0.2 | 3.6 | 3.5 | 3.1 | 3.2 | 0.3 | -0.1 |
| 70-79 yrs | 10.0 | 5.7 | 3.9 | -0.5 | 4.8 | 3.0 | -1.3 | -0.7 | 7.8 | 5.8 | 1.5 | 2.2 | -0.6 | -0.2 |
| 80+ yrs | 7.0 | 3.7 | 1.5 | 1.7 | 1.9 | -0.5 | -0.3 | 1.8 | 3.7 | 0.9 | 1.4 | 3.7 | 2.3 | -0.2 |
| Federal state |  |  |  |  |  |  |  |  |  |  |  |  |  |  |
| Schleswig-Holstein | 3.5 | 12.2 | -0.1 | 0.0 | 12.3 | -0.1 | 0.1 | 0.2 | 12.1 | -0.4 | -0.1 | 0.0 | 0.4 | 0.0 |
| Hamburg | 2.2 | 3.3 | -0.5 | 0.0 | 3.7 | -0.2 | 0.4 | 0.3 | 3.2 | -0.7 | 0.0 | -0.2 | 0.1 | 0.0 |
| Lower Saxony | 9.6 | -3.6 | 0.1 | 0.1 | -3.9 | -0.2 | -0.1 | -0.1 | -3.5 | 0.0 | 0.2 | 0.2 | 0.2 | 0.0 |
| Bremen | 0.8 | 8.3 | -0.1 | 0.0 | 8.3 | -0.1 | -0.1 | 0.0 | 8.4 | -0.2 | -0.2 | -0.3 | 0.2 | 0.0 |
| North Rhine-Westphalia | 21.2 | -7.3 | -0.2 | 0.0 | -7.3 | 0.0 | 0.2 | -0.2 | -7.3 | -0.2 | 0.2 | -0.1 | -0.4 | 0.0 |
| Hesse | 7.6 | -4.1 | 0.4 | 0.0 | -3.7 | 0.7 | 0.1 | -0.1 | -4.2 | 0.0 | -0.7 | -0.8 | -0.7 | -0.1 |
| Rhineland-Palatinate | 5.0 | -3.3 | 0.3 | 0.0 | -2.9 | 0.7 | 0.5 | 0.4 | -2.2 | 1.5 | 1.7 | 1.6 | 1.1 | 0.0 |
| Baden-Württemberg | 13.4 | -5.9 | -0.3 | 0.0 | -5.5 | 0.0 | 0.2 | 0.1 | -5.6 | -0.2 | -0.5 | -0.7 | -1.2 | 0.1 |
| Bavaria | 16.0 | -5.8 | -0.2 | 0.0 | -5.9 | -0.4 | -0.3 | -0.2 | -5.0 | 0.7 | 0.6 | 0.6 | 0.3 | 0.0 |
| Saarland | 1.2 | 7.2 | 0.1 | 0.0 | 7.3 | 0.1 | 0.0 | 0.1 | 7.5 | 0.2 | -0.1 | 0.0 | -0.2 | 0.0 |
| Berlin | 4.4 | 10.7 | 0.1 | 0.0 | 11.0 | 0.2 | 0.3 | 0.2 | 10.4 | -0.3 | -0.1 | -0.3 | 0.9 | 0.0 |
| Brandenburg | 3.1 | 0.7 | 0.0 | -0.1 | 0.5 | -0.2 | -0.1 | 0.0 | 0.1 | -0.6 | -0.4 | -0.3 | 0.0 | 0.0 |
| Mecklenburg-Western Pomerania | 2.0 | 3.7 | -0.2 | 0.0 | 3.1 | -0.6 | -0.4 | -0.3 | 2.9 | -0.8 | -0.4 | -0.3 | -0.1 | 0.0 |
| Saxony | 4.9 | -2.8 | 0.0 | 0.0 | -3.0 | -0.2 | -0.3 | 0.0 | -2.8 | 0.4 | 0.0 | 0.5 | 0.6 | 0.0 |
| Saxony-Anhalt | 2.6 | 2.2 | 0.3 | 0.0 | 1.6 | -0.1 | -0.3 | 0.1 | 1.3 | -0.4 | -0.6 | -0.2 | -0.2 | -0.1 |
| Thuringia | 2.6 | 2.4 | 0.3 | 0.0 | 1.7 | -0.3 | -0.6 | -0.2 | 2.0 | -0.2 | -0.5 | -0.1 | -0.2 | 0.0 |
| German |  |  |  |  |  |  |  |  |  |  |  |  |  |  |
| Yes | 84.1 | 15.7 | 15.3 | 13.9 | 17.9 | 17.5 | 16.7 | 14.3 | 21.0 | 20.5 | 20.2 | 18.6 | 16.0 | 16.2 |
| No | 15.9 | -15.7 | -15.3 | -13.9 | -17.9 | -17.5 | -16.7 | -14.3 | -21.0 | -20.5 | -20.2 | -18.6 | -16.0 | -16.2 |
| BIK |  |  |  |  |  |  |  |  |  |  |  |  |  |  |
| BIK 1 | 10.9 | -0.9 | -0.4 | 0.0 | -1.5 | -1.0 | -0.6 | -0.1 | -1.3 | -0.9 | -0.4 | 0.2 | 0.1 | 0.1 |
| BIK 2 | 34.7 | -1.1 | 1.2 | -0.1 | -1.6 | 0.7 | -0.7 | 0.0 | -1.1 | 1.2 | -0.3 | 0.4 | 0.0 | -0.1 |
| BIK 3 | 26.6 | -2.5 | -0.9 | -0.3 | -2.2 | -0.6 | 0.0 | -0.3 | -2.3 | -0.7 | -0.1 | -0.4 | -0.6 | -0.4 |
| BIK 4 | 27.7 | 4.1 | -0.1 | 0.4 | 4.8 | 0.5 | 1.2 | 0.5 | 4.2 | 0.0 | 0.7 | -0.2 | 0.6 | 0.5 |
| Education |  |  |  |  |  |  |  |  |  |  |  |  |  |  |
| Low | 34.9 | -11.5 | -11.2 | -1.3 | -14.7 | -14.6 | -5.0 | -1.4 | -15.7 | -15.7 | -6.9 | -3.3 | -2.9 | -2.0 |
| Medium | 43.0 | 0.9 | 1.0 | 1.3 | 1.3 | 1.5 | 2.7 | 1.3 | 0.4 | 0.7 | 2.5 | 1.3 | 2.3 | 1.6 |
| High | 22.1 | 10.8 | 10.5 | 0.0 | 13.3 | 13.0 | 2.5 | 0.1 | 15.2 | 14.8 | 4.7 | 2.2 | 0.5 | 0.4 |
| Household size |  |  |  |  |  |  |  |  |  |  |  |  |  |  |
| Single-person household | 24.8 | -5.4 | -6.3 | 0.1 | -5.8 | -6.7 | -0.2 | 0.2 | -6.5 | -7.8 | -1.4 | -1.0 | -0.2 | 0.0 |
| Multi-person household | 75.2 | 5.4 | 6.3 | -0.1 | 5.8 | 6.7 | 0.2 | -0.2 | 6.5 | 7.8 | 1.4 | 1.0 | 0.2 | 0.0 |
| PAbmi\_k2 |  |  |  |  |  |  |  |  |  |  |  |  |  |  |
| Normal weight (18.5 <= BMI < 25) | 34.4 | 1.4 | 0.8 | 0.0 | 1.8 | 1.3 | 0.2 | -0.1 | 1.8 | 1.3 | 0.4 | 0.1 | 0.4 | 0.5 |
| Underweight (BMI < 18.5) | 1.1 | 0.0 | -0.5 | 0.0 | -0.3 | -0.7 | -0.4 | -0.4 | -1.4 | -1.9 | -1.8 | -1.9 | -1.1 | -1.2 |
| Overweight (25 <= BMI < 30) | 41.9 | 0.2 | 0.6 | 0.0 | -0.1 | 0.4 | -0.2 | 0.1 | 0.9 | 1.4 | 0.9 | 1.1 | 0.2 | 0.1 |
| Obesity (BMI >= 30) | 22.6 | -1.9 | -1.5 | 0.0 | -2.0 | -1.7 | 0.0 | 0.1 | -2.8 | -2.8 | -1.1 | -1.0 | -0.5 | -0.5 |
| Self-rated health |  |  |  |  |  |  |  |  |  |  |  |  |  |  |
| Very good/good/fair | 93.5 | 0.3 | 0.7 | 0.0 | 1.6 | 2.0 | 1.3 | 0.2 | 1.7 | 2.3 | 1.5 | 0.4 | 0.2 | 0.8 |
| Bad/very bad | 6.5 | -0.3 | -0.7 | 0.0 | -1.6 | -2.0 | -1.3 | -0.2 | -1.7 | -2.3 | -1.5 | -0.4 | -0.2 | -0.8 |
| Self-rated mental health |  |  |  |  |  |  |  |  |  |  |  |  |  |  |
| Excellent/very good/good | 81.4 | 1.6 | 1.8 | 0.0 | 2.0 | 2.1 | 0.1 | -0.2 | 3.0 | 3.4 | 1.4 | 1.0 | 0.3 | 0.3 |
| fair/poor | 18.6 | -1.6 | -1.8 | 0.0 | -2.0 | -2.1 | -0.1 | 0.2 | -3.0 | -3.4 | -1.4 | -1.0 | -0.3 | -0.3 |
| Paying attention to health |  |  |  |  |  |  |  |  |  |  |  |  |  |  |
| Not at all/less strong/moderate | 56.1 | -1.4 | -1.3 | 0.0 | -1.9 | -1.9 | -0.4 | 0.1 | -3.1 | -2.6 | -1.3 | -0.9 | 0.3 | 0.3 |
| Strong/very strong | 43.9 | 1.4 | 1.3 | 0.0 | 1.9 | 1.9 | 0.4 | -0.1 | 3.1 | 2.6 | 1.3 | 0.9 | -0.3 | -0.3 |
| Satisfaction: Life in general |  |  |  |  |  |  |  |  |  |  |  |  |  |  |
| Scale value 1 to 3 | 8.6 | -1.8 | -1.6 | 0.0 | -2.2 | -2.0 | -0.3 | 0.0 | -2.9 | -3.1 | -1.2 | -0.8 | 0.1 | 0.3 |
| Scale value 4 to 7 | 44.2 | -1.4 | -1.6 | 0.0 | -2.0 | -2.3 | -0.7 | -0.1 | -3.2 | -3.1 | -1.8 | -1.2 | 0.2 | 0.2 |
| Scale value 8 to 10 | 47.2 | 2.3 | 2.5 | 0.0 | 3.2 | 3.3 | 0.8 | 0.1 | 4.7 | 4.8 | 2.4 | 1.6 | -0.3 | -0.3 |
| Red meat |  |  |  |  |  |  |  |  |  |  |  |  |  |  |
| Never | 5.8 | 0.5 | -0.2 | 0.0 | 0.8 | 0.1 | 0.3 | 0.1 | 0.5 | -0.2 | -0.3 | -0.4 | -0.1 | -0.1 |
| Daily or several times a day | 4.4 | -1.7 | -1.0 | 0.0 | -2.4 | -1.6 | -0.5 | -0.1 | -3.8 | -3.3 | -2.1 | -1.8 | -0.5 | -0.3 |
| 4 to 6 times per week | 17.3 | -1.5 | -0.3 | 0.0 | -1.6 | -0.4 | 0.1 | 0.0 | -2.8 | -1.5 | -1.2 | -1.3 | -0.3 | 0.1 |
| 1 to 3 times per week | 49.9 | 0.5 | 0.6 | 0.0 | 0.3 | 0.4 | -0.2 | 0.0 | 1.1 | 1.1 | 0.6 | 0.7 | 0.1 | 0.1 |
| Less than once per week | 22.6 | 1.3 | 0.2 | 0.0 | 1.7 | 0.6 | 0.2 | 0.0 | 2.6 | 1.6 | 1.5 | 1.4 | 0.4 | 0.0 |
| Sausage products |  |  |  |  |  |  |  |  |  |  |  |  |  |  |
| Never | 7.2 | -0.1 | -0.3 | 0.0 | 0.2 | -0.1 | 0.1 | -0.1 | -1.1 | -1.2 | -1.1 | -1.5 | -0.4 | -0.5 |
| Daily or several times a day | 13.8 | -0.3 | -0.7 | 0.0 | -0.9 | -1.1 | -0.4 | 0.0 | -1.0 | -1.3 | -1.0 | -0.6 | -0.2 | -0.2 |
| 4 to 6 times per week | 25.1 | -0.3 | 0.1 | 0.0 | 0.1 | 0.4 | 0.3 | 0.0 | 1.0 | 1.2 | 1.0 | 0.8 | 0.5 | 0.4 |
| 1 to 3 times per week | 35.4 | 0.0 | 0.3 | 0.0 | -0.1 | 0.1 | 0.0 | 0.0 | -0.2 | 0.3 | 0.4 | 0.5 | -0.2 | -0.3 |
| Less than once per week | 18.4 | 0.7 | 0.3 | 0.0 | 0.8 | 0.4 | 0.0 | 0.0 | 0.7 | 0.2 | -0.1 | 0.0 | 0.2 | 0.3 |
| Smoking |  |  |  |  |  |  |  |  |  |  |  |  |  |  |
| Non-smoker | 72.8 | 4.4 | 3.9 | 0.0 | 5.4 | 5.0 | 1.1 | 0.4 | 8.8 | 8.3 | 5.4 | 4.7 | 1.3 | 1.0 |
| Daily smoking | 20.4 | -4.7 | -4.1 | 0.0 | -5.7 | -5.2 | -1.1 | -0.4 | -8.9 | -8.4 | -5.4 | -4.8 | -1.8 | -1.6 |
| Occasional smoking | 6.8 | -0.3 | -0.4 | 0.0 | -0.5 | -0.6 | -0.3 | -0.1 | -1.7 | -1.4 | -1.0 | -0.7 | 0.5 | 0.8 |
| Chronic diseases |  |  |  |  |  |  |  |  |  |  |  |  |  |  |
| No | 48.4 | -0.6 | -0.1 | 0.0 | -0.8 | -0.4 | -0.5 | -0.2 | -3.1 | -2.2 | -2.6 | -2.4 | -0.8 | -0.3 |
| Yes | 51.6 | 0.6 | 0.1 | 0.0 | 0.8 | 0.4 | 0.5 | 0.2 | 3.1 | 2.2 | 2.6 | 2.4 | 0.8 | 0.3 |
| Sport |  |  |  |  |  |  |  |  |  |  |  |  |  |  |
| No sporting activities | 23.3 | -1.8 | -2.4 | 0.0 | -3.3 | -4.0 | -1.7 | 0.0 | -4.1 | -4.8 | -3.0 | -1.5 | -0.5 | -0.8 |
| Less than 1 hour per week | 19.3 | -1.0 | -0.8 | 0.0 | -1.3 | -1.1 | -0.2 | 0.0 | -1.7 | -1.4 | -0.6 | -0.3 | 0.2 | 0.1 |
| 1 to less than 2 hours per week | 24.1 | 0.5 | 0.6 | 0.0 | 0.7 | 0.8 | 0.3 | 0.1 | 1.7 | 1.6 | 1.4 | 1.4 | 0.5 | 0.5 |
| 2 to less than 4 hours per week | 18.8 | 1.6 | 1.7 | 0.0 | 2.4 | 2.5 | 0.8 | 0.0 | 3.4 | 3.4 | 1.7 | 0.9 | 0.0 | 0.0 |
| 4 hours per week and more | 14.6 | 0.8 | 1.1 | 0.0 | 1.7 | 1.9 | 1.0 | 0.0 | 0.7 | 1.4 | 0.5 | -0.6 | -0.2 | 0.2 |
| KWsubj\_k |  |  |  |  |  |  |  |  |  |  |  |  |  |  |
| Scale value 1 to 3 | 40.0 | 0.1 | 0.0 | 0.0 | -0.3 | -0.4 | -0.4 | -0.2 | -0.2 | -0.3 | -0.4 | -0.2 | -0.2 | -0.5 |
| Scale value 4 to 7 | 44.6 | -0.1 | -0.1 | 0.0 | 0.0 | 0.0 | 0.2 | 0.1 | 0.2 | -0.1 | 0.1 | 0.2 | 0.3 | 0.5 |
| Scale value 8 to 10 | 15.4 | -0.1 | 0.1 | 0.0 | 0.5 | 0.6 | 0.3 | 0.1 | 0.1 | 0.5 | 0.5 | 0.0 | -0.2 | 0.0 |
| IAtermin |  |  |  |  |  |  |  |  |  |  |  |  |  |  |
| No | 55.6 | 2.0 | 1.1 | 0.0 | 2.0 | 1.2 | -0.1 | 0.1 | 4.3 | 3.5 | 2.3 | 2.4 | 0.3 | -0.2 |
| Yes | 30.9 | -1.4 | -0.9 | 0.0 | -1.6 | -1.1 | -0.1 | -0.1 | -3.1 | -2.5 | -1.5 | -1.5 | -0.5 | -0.3 |
| No need for examination or treatment | 13.5 | -1.0 | -0.4 | 0.0 | -0.7 | -0.2 | 0.2 | 0.0 | -2.1 | -1.7 | -1.2 | -1.5 | 0.3 | 0.7 |
|  |  |  |  |  |  |  |  |  |  |  |  |  |  |  |
| --- | --- | --- | --- | --- | --- | --- | --- | --- | --- | --- | --- | --- | --- | --- |
| aCS = Cross-Sectional | | | | | | | | | | | | | | |

# Questionnaire C

## Female and male

Table 3.1: Standardized differences in percentage points compared to reference values for the recruitment survey, registration sample, and the sample of participants in Questionnaire C; calculated unweighted and weighted according to the respective weighting factors.

|  |  | Recruitment Survey | | | Registration | | | | Participation Questionnaire C | | | | | |
| --- | --- | --- | --- | --- | --- | --- | --- | --- | --- | --- | --- | --- | --- | --- |
| Parameter | Reference [%] | Unweighted | Design weight | CSa weight | Unweighted | Design weight | CSa weight | Drop-out Weight | Unweighted | Design weight | CSa weight (Recruitment Study) | Drop-out weight (Registration) | Drop-out weight | CSa weight |
| sex |  |  |  |  |  |  |  |  |  |  |  |  |  |  |
| Male | 48.9 | -2.0 | -1.9 | 0.0 | -2.2 | -2.1 | 0.0 | 0.0 | -3.4 | -3.2 | -1.4 | -1.5 | -0.3 | -0.1 |
| Female | 51.1 | 2.0 | 1.9 | 0.0 | 2.2 | 2.1 | 0.0 | 0.0 | 3.4 | 3.2 | 1.4 | 1.5 | 0.3 | 0.1 |
| Agegrp |  |  |  |  |  |  |  |  |  |  |  |  |  |  |
| 18-29 yrs | 16.0 | 0.6 | -0.3 | -0.1 | 1.7 | 0.7 | 0.9 | -0.3 | -2.5 | -3.4 | -3.3 | -4.4 | -0.8 | 0.0 |
| 30-39 yrs | 15.8 | -0.9 | -0.1 | 0.0 | 0.3 | 1.1 | 1.4 | 0.1 | -1.6 | -0.7 | -0.8 | -2.2 | -0.2 | 0.3 |
| 40-49 yrs | 14.7 | -3.8 | -0.1 | 0.1 | -3.3 | 0.4 | 0.7 | 0.1 | -4.2 | -0.6 | -0.4 | -1.1 | 0.0 | -0.3 |
| 50-59 yrs | 17.6 | 0.4 | 0.6 | 0.4 | 0.8 | 0.9 | 0.7 | 0.4 | 1.7 | 2.1 | 1.8 | 1.4 | 0.4 | 0.5 |
| 60-69 yrs | 16.5 | -0.1 | -0.1 | -0.1 | -0.1 | -0.2 | -0.2 | 0.0 | 2.5 | 2.4 | 2.9 | 2.9 | 0.3 | -0.2 |
| 70-79 yrs | 10.7 | 3.5 | 1.7 | -0.2 | 2.4 | 0.4 | -1.4 | -0.1 | 5.0 | 2.9 | 1.1 | 2.4 | 0.3 | 0.0 |
| 80+ yrs | 8.7 | 0.6 | -1.8 | -0.1 | -2.3 | -4.6 | -3.2 | -0.1 | -1.2 | -3.7 | -2.1 | 1.0 | 0.0 | -0.4 |
| Federal state |  |  |  |  |  |  |  |  |  |  |  |  |  |  |
| Schleswig-Holstein | 3.5 | 12.5 | 0.0 | 0.0 | 12.6 | 0.1 | 0.2 | 0.1 | 12.8 | 0.2 | 0.2 | 0.2 | 0.3 | 0.0 |
| Hamburg | 2.2 | 3.9 | -0.1 | 0.0 | 4.3 | 0.3 | 0.4 | 0.2 | 4.1 | 0.0 | 0.3 | 0.1 | 0.3 | 0.0 |
| Lower Saxony | 9.6 | -3.8 | 0.1 | 0.1 | -3.9 | 0.0 | 0.0 | -0.1 | -3.7 | 0.3 | 0.2 | 0.1 | -0.1 | 0.0 |
| Bremen | 0.8 | 8.3 | -0.1 | 0.0 | 8.6 | 0.1 | 0.1 | 0.1 | 8.6 | 0.1 | -0.1 | -0.1 | 0.1 | 0.0 |
| North Rhine-Westphalia | 21.4 | -7.2 | 0.0 | 0.0 | -7.2 | 0.2 | 0.1 | -0.2 | -7.0 | 0.4 | 0.2 | -0.1 | 0.1 | 0.0 |
| Hesse | 7.6 | -4.4 | 0.0 | 0.0 | -4.5 | -0.2 | -0.2 | -0.2 | -5.8 | -1.8 | -2.0 | -2.0 | -1.8 | -0.1 |
| Rhineland-Palatinate | 4.9 | -3.5 | 0.0 | 0.0 | -3.2 | 0.5 | 0.5 | 0.5 | -2.8 | 0.8 | 0.9 | 0.9 | 0.9 | 0.0 |
| Baden-Württemberg | 13.3 | -5.9 | -0.1 | 0.0 | -5.6 | 0.1 | 0.1 | -0.1 | -5.8 | 0.0 | -0.2 | -0.4 | -0.9 | 0.0 |
| Bavaria | 15.9 | -5.7 | 0.0 | 0.0 | -5.8 | -0.1 | -0.2 | 0.0 | -5.4 | 0.5 | 0.7 | 0.8 | 0.7 | 0.0 |
| Saarland | 1.2 | 7.1 | 0.0 | 0.0 | 7.0 | 0.0 | 0.0 | 0.1 | 7.2 | 0.1 | 0.1 | 0.2 | 0.4 | 0.0 |
| Berlin | 4.5 | 10.5 | 0.0 | 0.0 | 10.9 | 0.3 | 0.4 | 0.3 | 10.4 | -0.1 | 0.0 | -0.2 | 0.5 | 0.0 |
| Brandenburg | 3.1 | 0.8 | 0.0 | -0.1 | 0.5 | -0.2 | -0.2 | -0.1 | 0.8 | 0.0 | 0.1 | 0.3 | 0.3 | 0.0 |
| Mecklenburg-Western Pomerania | 2.0 | 4.0 | 0.0 | 0.0 | 3.5 | -0.3 | -0.3 | -0.2 | 3.6 | -0.3 | -0.3 | -0.3 | -0.2 | 0.0 |
| Saxony | 4.9 | -2.6 | 0.0 | 0.0 | -2.9 | -0.4 | -0.3 | 0.0 | -2.8 | -0.2 | -0.1 | 0.3 | 0.0 | 0.0 |
| Saxony-Anhalt | 2.6 | 1.8 | 0.0 | 0.0 | 1.4 | -0.3 | -0.2 | 0.2 | 1.5 | -0.3 | -0.2 | 0.2 | 0.1 | 0.0 |
| Thuringia | 2.5 | 2.0 | 0.0 | 0.0 | 1.4 | -0.5 | -0.5 | -0.1 | 1.7 | -0.2 | -0.3 | 0.1 | 0.0 | 0.0 |
| German |  |  |  |  |  |  |  |  |  |  |  |  |  |  |
| Yes | 85.0 | 14.2 | 13.6 | 12.5 | 16.1 | 15.7 | 15.0 | 12.6 | 19.2 | 18.9 | 18.5 | 16.9 | 14.0 | 14.0 |
| No | 15.0 | -14.2 | -13.6 | -12.5 | -16.1 | -15.7 | -15.0 | -12.6 | -19.2 | -18.9 | -18.5 | -16.9 | -14.0 | -14.0 |
| BIK |  |  |  |  |  |  |  |  |  |  |  |  |  |  |
| BIK 1 | 10.8 | -0.8 | -0.4 | 0.0 | -1.4 | -0.9 | -0.5 | 0.0 | -1.9 | -1.3 | -0.7 | 0.0 | 0.3 | 0.0 |
| BIK 2 | 34.6 | -1.2 | 1.1 | 0.0 | -1.8 | 0.6 | -0.6 | 0.1 | -1.2 | 1.4 | 0.0 | 0.7 | 0.0 | 0.0 |
| BIK 3 | 26.8 | -2.4 | -0.6 | 0.0 | -2.2 | -0.4 | 0.2 | -0.1 | -2.2 | -0.4 | 0.2 | -0.2 | -0.1 | 0.0 |
| BIK 4 | 27.9 | 4.1 | -0.3 | 0.0 | 4.9 | 0.3 | 0.7 | 0.0 | 4.5 | -0.2 | 0.2 | -0.5 | -0.1 | 0.0 |
| Education |  |  |  |  |  |  |  |  |  |  |  |  |  |  |
| Low | 34.4 | -12.8 | -12.8 | -1.3 | -16.8 | -16.8 | -5.8 | -1.5 | -17.5 | -17.6 | -7.2 | -3.1 | -2.6 | -1.2 |
| Medium | 45.3 | 2.3 | 2.5 | 1.4 | 3.3 | 3.5 | 3.4 | 1.5 | 2.6 | 2.9 | 3.4 | 1.7 | 2.2 | 1.1 |
| High | 20.3 | 10.7 | 10.5 | -0.1 | 13.2 | 12.9 | 2.3 | 0.0 | 14.5 | 14.3 | 3.9 | 1.5 | 0.3 | 0.0 |
| Household size |  |  |  |  |  |  |  |  |  |  |  |  |  |  |
| Single-person household | 25.4 | -3.5 | -4.8 | 0.2 | -4.6 | -5.8 | -0.9 | 0.1 | -4.7 | -5.8 | -1.2 | -0.1 | 0.1 | 0.5 |
| Multi-person household | 74.6 | 3.5 | 4.8 | -0.2 | 4.6 | 5.8 | 0.9 | -0.1 | 4.7 | 5.8 | 1.2 | 0.1 | -0.1 | -0.5 |
| PAbmi\_k2 |  |  |  |  |  |  |  |  |  |  |  |  |  |  |
| Normal weight (18.5 <= BMI < 25) | 40.7 | 2.0 | 1.9 | 0.0 | 2.5 | 2.5 | 0.4 | -0.1 | 2.5 | 2.5 | 0.6 | 0.1 | -0.2 | -0.2 |
| Underweight (BMI < 18.5) | 2.0 | 0.2 | -0.1 | 0.0 | 0.2 | -0.1 | -0.1 | -0.1 | -0.3 | -0.7 | -0.7 | -0.7 | -0.3 | -0.1 |
| Overweight (25 <= BMI < 30) | 35.2 | -0.3 | -0.3 | 0.0 | -0.7 | -0.7 | -0.4 | 0.1 | -0.3 | -0.4 | 0.0 | 0.5 | 0.2 | 0.1 |
| Obesity (BMI >= 30) | 22.1 | -2.2 | -1.9 | 0.0 | -2.3 | -2.1 | -0.1 | 0.0 | -2.5 | -2.3 | -0.4 | -0.4 | 0.1 | 0.2 |
| Self-rated health |  |  |  |  |  |  |  |  |  |  |  |  |  |  |
| Very good/good/fair | 93.1 | 0.9 | 1.3 | 0.0 | 2.4 | 2.7 | 1.5 | 0.0 | 3.2 | 3.6 | 2.4 | 1.1 | 0.0 | 0.5 |
| Bad/very bad | 6.9 | -0.9 | -1.3 | 0.0 | -2.4 | -2.7 | -1.5 | 0.0 | -3.2 | -3.6 | -2.4 | -1.1 | 0.0 | -0.5 |
| Self-rated mental health |  |  |  |  |  |  |  |  |  |  |  |  |  |  |
| Excellent/very good/good | 78.5 | 1.3 | 1.6 | 0.0 | 1.7 | 1.9 | 0.3 | 0.0 | 2.9 | 3.1 | 1.7 | 1.3 | 0.0 | 0.2 |
| fair/poor | 21.5 | -1.3 | -1.6 | 0.0 | -1.7 | -1.9 | -0.3 | 0.0 | -2.9 | -3.1 | -1.7 | -1.3 | 0.0 | -0.2 |
| Paying attention to health |  |  |  |  |  |  |  |  |  |  |  |  |  |  |
| Not at all/less strong/moderate | 51.6 | -1.7 | -1.6 | 0.0 | -2.2 | -2.2 | -0.5 | 0.0 | -3.5 | -3.4 | -1.8 | -1.2 | 0.1 | 0.3 |
| Strong/very strong | 48.4 | 1.7 | 1.6 | 0.0 | 2.2 | 2.2 | 0.5 | 0.0 | 3.5 | 3.4 | 1.8 | 1.2 | -0.1 | -0.3 |
| Satisfaction: Life in general |  |  |  |  |  |  |  |  |  |  |  |  |  |  |
| Scale value 1 to 3 | 8.5 | -1.4 | -1.5 | 0.0 | -2.0 | -2.1 | -0.5 | -0.1 | -2.9 | -2.8 | -1.5 | -1.1 | -0.2 | -0.2 |
| Scale value 4 to 7 | 45.1 | -0.9 | -1.0 | 0.0 | -1.3 | -1.4 | -0.5 | 0.1 | -2.6 | -2.6 | -1.8 | -1.3 | -0.2 | -0.1 |
| Scale value 8 to 10 | 46.4 | 1.7 | 1.8 | 0.0 | 2.4 | 2.5 | 0.7 | 0.0 | 4.1 | 4.1 | 2.6 | 1.9 | 0.3 | 0.2 |
| Red meat |  |  |  |  |  |  |  |  |  |  |  |  |  |  |
| Never | 9.3 | 1.2 | 0.6 | 0.0 | 1.7 | 1.0 | 0.4 | 0.0 | 1.7 | 1.1 | 0.7 | 0.4 | 0.6 | 0.6 |
| Daily or several times a day | 2.8 | -1.6 | -1.1 | 0.0 | -2.1 | -1.7 | -0.4 | 0.0 | -3.3 | -2.8 | -1.8 | -1.6 | -0.7 | -0.3 |
| 4 to 6 times per week | 12.2 | -1.4 | -0.4 | 0.0 | -1.6 | -0.5 | 0.1 | 0.0 | -2.6 | -1.6 | -1.0 | -1.0 | 0.1 | 0.2 |
| 1 to 3 times per week | 46.1 | -0.6 | -0.1 | 0.0 | -1.1 | -0.5 | -0.4 | 0.0 | -0.7 | -0.1 | -0.2 | 0.1 | -0.3 | -0.4 |
| Less than once per week | 29.6 | 1.4 | 0.4 | 0.0 | 1.9 | 0.9 | 0.3 | 0.0 | 2.5 | 1.4 | 1.1 | 0.9 | 0.1 | 0.0 |
| Sausage products |  |  |  |  |  |  |  |  |  |  |  |  |  |  |
| Never | 10.3 | 1.5 | 1.1 | 0.0 | 2.1 | 1.6 | 0.4 | -0.1 | 1.7 | 1.1 | -0.1 | -0.7 | -0.1 | -0.1 |
| Daily or several times a day | 10.0 | -0.7 | -1.0 | 0.0 | -1.2 | -1.4 | -0.3 | 0.0 | -1.2 | -1.5 | -0.5 | -0.3 | 0.0 | 0.1 |
| 4 to 6 times per week | 19.4 | -0.6 | -0.2 | 0.0 | -0.3 | 0.1 | 0.4 | 0.0 | 0.1 | 0.6 | 0.9 | 0.7 | 0.2 | 0.4 |
| 1 to 3 times per week | 35.6 | -0.7 | -0.3 | 0.0 | -1.3 | -0.8 | -0.4 | 0.0 | -1.3 | -0.7 | -0.5 | -0.1 | 0.1 | -0.1 |
| Less than once per week | 24.8 | 0.8 | 0.4 | 0.0 | 1.0 | 0.6 | 0.1 | 0.1 | 1.0 | 0.5 | 0.2 | 0.1 | -0.3 | -0.2 |
| Smoking |  |  |  |  |  |  |  |  |  |  |  |  |  |  |
| Non-smoker | 76.2 | 3.5 | 3.2 | 0.0 | 4.1 | 3.8 | 0.5 | 0.0 | 7.4 | 7.0 | 4.4 | 4.1 | 0.6 | 0.5 |
| Daily smoking | 17.9 | -3.9 | -3.5 | 0.0 | -4.5 | -4.1 | -0.5 | -0.1 | -7.3 | -6.9 | -4.2 | -3.9 | -0.8 | -0.7 |
| Occasional smoking | 5.9 | -0.1 | -0.1 | 0.0 | -0.1 | -0.2 | -0.1 | 0.0 | -1.7 | -1.6 | -1.3 | -1.0 | 0.1 | 0.2 |
| Chronic diseases |  |  |  |  |  |  |  |  |  |  |  |  |  |  |
| No | 45.0 | -0.1 | 0.7 | 0.0 | -0.3 | 0.4 | -0.4 | -0.1 | -2.1 | -1.2 | -2.0 | -1.7 | -0.4 | -0.1 |
| Yes | 55.0 | 0.1 | -0.7 | 0.0 | 0.3 | -0.4 | 0.4 | 0.1 | 2.1 | 1.2 | 2.0 | 1.7 | 0.4 | 0.1 |
| Sport |  |  |  |  |  |  |  |  |  |  |  |  |  |  |
| No sporting activities | 23.0 | -2.6 | -3.1 | 0.0 | -4.7 | -5.2 | -2.3 | -0.1 | -5.6 | -6.3 | -3.7 | -1.6 | -0.6 | -0.5 |
| Less than 1 hour per week | 19.7 | -0.9 | -0.5 | 0.0 | -1.0 | -0.7 | -0.1 | 0.0 | -1.5 | -1.2 | -0.6 | -0.5 | 0.1 | 0.3 |
| 1 to less than 2 hours per week | 26.5 | 1.0 | 1.0 | 0.0 | 1.4 | 1.4 | 0.5 | 0.1 | 2.1 | 2.0 | 1.3 | 0.9 | 0.2 | 0.0 |
| 2 to less than 4 hours per week | 18.9 | 1.7 | 1.8 | 0.0 | 2.8 | 3.0 | 1.2 | 0.0 | 3.8 | 4.2 | 2.5 | 1.4 | 0.3 | 0.2 |
| 4 hours per week and more | 11.9 | 0.8 | 0.9 | 0.0 | 1.6 | 1.8 | 0.9 | 0.0 | 1.2 | 1.4 | 0.7 | -0.3 | -0.1 | 0.0 |
| KWsubj\_k |  |  |  |  |  |  |  |  |  |  |  |  |  |  |
| Scale value 1 to 3 | 34.8 | -0.3 | -0.4 | 0.0 | -0.8 | -0.9 | -0.4 | 0.0 | -0.9 | -1.0 | -0.8 | -0.4 | -0.1 | -0.2 |
| Scale value 4 to 7 | 49.0 | 0.1 | 0.1 | 0.0 | 0.1 | 0.2 | 0.2 | 0.0 | 0.4 | 0.3 | 0.5 | 0.5 | 0.3 | 0.4 |
| Scale value 8 to 10 | 16.1 | 0.3 | 0.4 | 0.0 | 0.8 | 0.8 | 0.3 | -0.1 | 0.6 | 0.8 | 0.2 | -0.2 | -0.2 | -0.3 |
| IAtermin |  |  |  |  |  |  |  |  |  |  |  |  |  |  |
| No | 54.8 | 0.9 | 0.3 | 0.0 | 0.6 | -0.1 | -0.5 | 0.0 | 2.3 | 1.5 | 1.0 | 1.5 | 0.2 | -0.1 |
| Yes | 33.5 | -0.7 | -0.3 | 0.0 | -0.5 | -0.2 | 0.3 | 0.0 | -1.8 | -1.4 | -0.8 | -1.2 | -0.2 | 0.0 |
| No need for examination or treatment | 11.7 | -0.4 | 0.1 | 0.0 | -0.1 | 0.3 | 0.3 | 0.0 | -0.9 | -0.2 | -0.3 | -0.7 | -0.1 | 0.1 |
|  |  |  |  |  |  |  |  |  |  |  |  |  |  |  |
| --- | --- | --- | --- | --- | --- | --- | --- | --- | --- | --- | --- | --- | --- | --- |
| aCS = Cross-Sectional | | | | | | | | | | | | | | |

## Female

Table 3.2: Standardized differences in percentage points compared to reference values for the recruitment survey, registration sample, and the sample of participants in Questionnaire C; calculated unweighted and weighted according to the respective weighting factors.

|  |  | Recruitment Survey | | | Registration | | | | Participation Questionnaire C | | | | | |
| --- | --- | --- | --- | --- | --- | --- | --- | --- | --- | --- | --- | --- | --- | --- |
| Parameter | Reference [%] | Unweighted | Design weight | CSa weight | Unweighted | Design weight | CSa weight | Drop-out Weight | Unweighted | Design weight | CSa weight (Recruitment Study) | Drop-out weight (Registration) | Drop-out weight | CSa weight |
| Agegrp |  |  |  |  |  |  |  |  |  |  |  |  |  |  |
| 18-29 yrs | 14.9 | 2.2 | 1.3 | -0.1 | 3.9 | 2.9 | 1.7 | 0.1 | 0.5 | -0.5 | -1.5 | -3.0 | 0.1 | 0.1 |
| 30-39 yrs | 15.1 | 0.2 | 0.9 | 0.2 | 1.5 | 2.3 | 1.9 | 0.1 | -0.2 | 0.7 | -0.1 | -1.9 | -0.3 | 0.2 |
| 40-49 yrs | 14.4 | -2.9 | 1.0 | 0.3 | -2.4 | 1.6 | 0.9 | 0.1 | -3.0 | 0.9 | 0.1 | -1.0 | 0.1 | -0.2 |
| 50-59 yrs | 17.2 | 1.3 | 1.4 | 0.7 | 1.9 | 1.9 | 1.3 | 0.8 | 3.0 | 3.3 | 2.6 | 1.9 | 1.1 | 0.6 |
| 60-69 yrs | 16.6 | -0.6 | -0.7 | 0.2 | -0.7 | -1.0 | 0.0 | 0.1 | 1.6 | 1.3 | 2.6 | 2.7 | 0.3 | -0.3 |
| 70-79 yrs | 11.4 | 1.5 | -0.4 | 0.0 | 0.1 | -1.9 | -1.5 | 0.3 | 2.2 | 0.0 | 0.5 | 2.3 | 0.5 | 0.1 |
| 80+ yrs | 10.4 | -2.2 | -4.6 | -1.5 | -6.0 | -8.2 | -5.8 | -1.6 | -5.6 | -8.0 | -5.6 | -1.6 | -2.2 | -0.6 |
| Federal state |  |  |  |  |  |  |  |  |  |  |  |  |  |  |
| Schleswig-Holstein | 3.5 | 12.7 | 0.1 | 0.0 | 12.9 | 0.3 | 0.2 | 0.0 | 13.4 | 0.6 | 0.5 | 0.4 | 0.3 | 0.0 |
| Hamburg | 2.3 | 4.4 | 0.3 | 0.0 | 4.8 | 0.7 | 0.3 | 0.1 | 4.7 | 0.6 | 0.6 | 0.3 | 0.4 | 0.0 |
| Lower Saxony | 9.6 | -3.9 | 0.0 | 0.1 | -3.9 | 0.1 | 0.2 | 0.0 | -3.6 | 0.6 | 0.6 | 0.4 | 0.1 | 0.0 |
| Bremen | 0.8 | 8.4 | 0.0 | -0.1 | 8.7 | 0.2 | 0.2 | 0.1 | 8.7 | 0.2 | 0.1 | 0.0 | 0.1 | 0.0 |
| North Rhine-Westphalia | 21.5 | -7.1 | 0.2 | 0.0 | -7.2 | 0.4 | 0.0 | -0.2 | -7.2 | 0.2 | -0.3 | -0.5 | -0.1 | 0.1 |
| Hesse | 7.6 | -4.8 | -0.4 | 0.0 | -5.4 | -1.0 | -0.5 | -0.4 | -6.8 | -2.8 | -2.1 | -1.9 | -1.5 | -0.1 |
| Rhineland-Palatinate | 4.9 | -3.8 | -0.3 | -0.1 | -3.4 | 0.2 | 0.5 | 0.6 | -3.1 | 0.5 | 0.9 | 1.1 | 1.2 | 0.0 |
| Baden-Württemberg | 13.2 | -5.8 | 0.1 | 0.0 | -5.7 | 0.1 | -0.1 | -0.2 | -5.9 | 0.0 | -0.4 | -0.6 | -0.9 | 0.0 |
| Bavaria | 15.8 | -5.6 | 0.1 | -0.1 | -5.6 | 0.2 | 0.0 | 0.1 | -5.6 | 0.5 | 0.3 | 0.3 | 0.2 | 0.0 |
| Saarland | 1.2 | 6.9 | 0.0 | 0.0 | 6.7 | -0.1 | -0.1 | 0.0 | 6.7 | -0.1 | 0.0 | 0.1 | 0.4 | 0.0 |
| Berlin | 4.5 | 10.2 | -0.2 | 0.0 | 10.8 | 0.3 | 0.6 | 0.3 | 10.4 | 0.1 | 0.3 | 0.0 | 0.4 | 0.0 |
| Brandenburg | 3.1 | 0.8 | 0.1 | -0.1 | 0.6 | -0.2 | -0.3 | -0.2 | 0.8 | 0.1 | 0.1 | 0.2 | 0.2 | 0.0 |
| Mecklenburg-Western Pomerania | 2.0 | 4.2 | 0.2 | 0.0 | 3.9 | 0.0 | -0.1 | 0.0 | 4.1 | 0.0 | -0.1 | -0.1 | 0.0 | 0.0 |
| Saxony | 4.9 | -2.5 | 0.0 | 0.1 | -2.8 | -0.5 | -0.4 | 0.0 | -2.7 | -0.4 | -0.1 | 0.3 | -0.1 | 0.0 |
| Saxony-Anhalt | 2.6 | 1.5 | -0.2 | 0.0 | 1.3 | -0.4 | -0.1 | 0.3 | 1.7 | -0.1 | 0.2 | 0.6 | 0.3 | 0.0 |
| Thuringia | 2.5 | 1.7 | -0.1 | 0.0 | 1.1 | -0.6 | -0.4 | 0.0 | 1.6 | -0.1 | 0.1 | 0.6 | 0.3 | 0.0 |
| German |  |  |  |  |  |  |  |  |  |  |  |  |  |  |
| Yes | 85.8 | 12.7 | 12.0 | 11.1 | 14.5 | 14.1 | 13.4 | 11.1 | 17.4 | 17.1 | 16.7 | 15.0 | 12.0 | 12.1 |
| No | 14.2 | -12.7 | -12.0 | -11.1 | -14.5 | -14.1 | -13.4 | -11.1 | -17.4 | -17.1 | -16.7 | -15.0 | -12.0 | -12.1 |
| BIK |  |  |  |  |  |  |  |  |  |  |  |  |  |  |
| BIK 1 | 10.7 | -0.8 | -0.4 | 0.0 | -1.4 | -0.8 | -0.4 | 0.2 | -1.8 | -1.2 | -0.6 | 0.1 | 0.3 | 0.1 |
| BIK 2 | 34.4 | -1.3 | 0.9 | 0.1 | -1.9 | 0.5 | -0.4 | 0.2 | -1.3 | 1.4 | 0.3 | 0.9 | 0.4 | 0.1 |
| BIK 3 | 26.9 | -2.3 | -0.3 | 0.2 | -2.2 | -0.2 | 0.5 | 0.1 | -2.2 | -0.4 | 0.2 | -0.1 | 0.0 | 0.1 |
| BIK 4 | 28.1 | 4.1 | -0.5 | -0.3 | 5.0 | 0.2 | 0.2 | -0.5 | 4.6 | -0.3 | -0.1 | -0.9 | -0.6 | -0.2 |
| Education |  |  |  |  |  |  |  |  |  |  |  |  |  |  |
| Low | 33.8 | -14.0 | -14.2 | -1.2 | -18.7 | -18.9 | -6.5 | -1.7 | -19.4 | -19.9 | -8.4 | -3.7 | -3.1 | -0.8 |
| Medium | 47.6 | 3.4 | 3.6 | 1.4 | 4.8 | 5.1 | 4.2 | 1.7 | 5.2 | 5.8 | 5.5 | 3.2 | 3.1 | 1.1 |
| High | 18.6 | 10.8 | 10.7 | -0.2 | 13.3 | 13.1 | 2.2 | -0.1 | 13.5 | 13.2 | 2.7 | 0.4 | -0.3 | -0.4 |
| Household size |  |  |  |  |  |  |  |  |  |  |  |  |  |  |
| Single-person household | 26.0 | -1.9 | -3.6 | 0.2 | -3.6 | -5.1 | -1.6 | 0.1 | -3.2 | -4.7 | -1.1 | 0.6 | 0.2 | 1.0 |
| Multi-person household | 74.0 | 1.9 | 3.6 | -0.2 | 3.6 | 5.1 | 1.6 | -0.1 | 3.2 | 4.7 | 1.1 | -0.6 | -0.2 | -1.0 |
| PAbmi\_k2 |  |  |  |  |  |  |  |  |  |  |  |  |  |  |
| Normal weight (18.5 <= BMI < 25) | 46.8 | 2.1 | 2.4 | 0.0 | 2.6 | 3.1 | 0.7 | 0.0 | 2.6 | 3.0 | 0.7 | -0.1 | -0.4 | -0.5 |
| Underweight (BMI < 18.5) | 2.9 | 0.2 | -0.1 | 0.0 | 0.2 | -0.1 | 0.1 | 0.0 | -0.3 | -0.6 | -0.7 | -0.6 | -0.3 | -0.2 |
| Overweight (25 <= BMI < 30) | 28.7 | -0.3 | -0.7 | 0.0 | -0.7 | -1.2 | -0.6 | 0.1 | -0.3 | -0.9 | 0.0 | 0.8 | 0.5 | 0.6 |
| Obesity (BMI >= 30) | 21.6 | -2.4 | -2.2 | 0.0 | -2.6 | -2.5 | -0.2 | -0.1 | -2.8 | -2.4 | -0.5 | -0.5 | 0.0 | 0.0 |
| Self-rated health |  |  |  |  |  |  |  |  |  |  |  |  |  |  |
| Very good/good/fair | 92.8 | 1.5 | 2.0 | 0.0 | 3.2 | 3.4 | 1.7 | -0.1 | 4.0 | 4.4 | 2.9 | 1.4 | 0.0 | 0.2 |
| Bad/very bad | 7.2 | -1.5 | -2.0 | 0.0 | -3.2 | -3.4 | -1.7 | 0.1 | -4.0 | -4.4 | -2.9 | -1.4 | 0.0 | -0.2 |
| Self-rated mental health |  |  |  |  |  |  |  |  |  |  |  |  |  |  |
| Excellent/very good/good | 75.6 | 1.3 | 1.7 | 0.0 | 1.7 | 2.1 | 0.6 | 0.2 | 2.9 | 3.4 | 2.1 | 1.8 | 0.1 | 0.2 |
| fair/poor | 24.3 | -1.3 | -1.7 | 0.0 | -1.7 | -2.1 | -0.6 | -0.2 | -2.9 | -3.4 | -2.1 | -1.8 | -0.1 | -0.2 |
| Paying attention to health |  |  |  |  |  |  |  |  |  |  |  |  |  |  |
| Not at all/less strong/moderate | 47.3 | -1.6 | -1.5 | 0.0 | -2.2 | -2.2 | -0.7 | -0.1 | -3.4 | -3.3 | -2.0 | -1.4 | 0.0 | 0.1 |
| Strong/very strong | 52.7 | 1.6 | 1.5 | 0.0 | 2.2 | 2.2 | 0.7 | 0.1 | 3.4 | 3.3 | 2.0 | 1.4 | 0.0 | -0.1 |
| Satisfaction: Life in general |  |  |  |  |  |  |  |  |  |  |  |  |  |  |
| Scale value 1 to 3 | 8.4 | -1.1 | -1.4 | 0.0 | -1.8 | -2.1 | -0.7 | -0.2 | -2.5 | -2.7 | -1.7 | -1.3 | -0.5 | -0.4 |
| Scale value 4 to 7 | 46.0 | -0.6 | -0.6 | 0.0 | -0.8 | -0.7 | -0.2 | 0.2 | -1.8 | -2.0 | -1.6 | -1.2 | 0.0 | 0.1 |
| Scale value 8 to 10 | 45.7 | 1.2 | 1.4 | 0.0 | 1.8 | 1.8 | 0.6 | -0.1 | 3.2 | 3.4 | 2.5 | 1.9 | 0.2 | 0.1 |
| Red meat |  |  |  |  |  |  |  |  |  |  |  |  |  |  |
| Never | 12.6 | 1.3 | 0.8 | 0.0 | 1.9 | 1.2 | 0.5 | 0.0 | 1.6 | 1.0 | 0.4 | 0.0 | 0.2 | 0.2 |
| Daily or several times a day | 1.2 | -1.0 | -1.0 | 0.0 | -1.4 | -1.4 | -0.2 | 0.0 | -2.2 | -2.1 | -1.2 | -1.1 | -0.4 | 0.0 |
| 4 to 6 times per week | 7.3 | -0.7 | 0.1 | 0.0 | -0.8 | -0.1 | 0.1 | 0.1 | -1.5 | -0.7 | -0.7 | -0.6 | 0.2 | 0.1 |
| 1 to 3 times per week | 42.6 | -1.3 | -0.5 | 0.0 | -2.0 | -1.0 | -0.6 | -0.1 | -1.6 | -0.5 | -0.3 | 0.2 | 0.0 | 0.0 |
| Less than once per week | 36.3 | 1.0 | 0.1 | 0.0 | 1.5 | 0.5 | 0.3 | 0.0 | 1.8 | 0.6 | 0.6 | 0.4 | -0.2 | -0.2 |
| Sausage products |  |  |  |  |  |  |  |  |  |  |  |  |  |  |
| Never | 13.2 | 2.3 | 1.7 | 0.0 | 3.1 | 2.4 | 0.6 | -0.1 | 2.7 | 1.9 | 0.3 | -0.4 | 0.1 | -0.1 |
| Daily or several times a day | 6.3 | -0.7 | -0.9 | 0.0 | -0.9 | -1.2 | -0.2 | -0.1 | -0.7 | -0.8 | -0.1 | -0.1 | 0.3 | 0.3 |
| 4 to 6 times per week | 13.9 | -0.4 | 0.1 | 0.0 | -0.1 | 0.4 | 0.5 | 0.1 | 0.3 | 0.9 | 0.9 | 0.6 | 0.1 | 0.3 |
| 1 to 3 times per week | 35.8 | -1.4 | -0.9 | 0.0 | -2.3 | -1.6 | -0.9 | 0.0 | -2.6 | -1.8 | -1.3 | -0.5 | 0.0 | -0.3 |
| Less than once per week | 30.8 | 0.4 | 0.0 | 0.0 | 0.6 | 0.2 | 0.2 | 0.1 | 0.8 | 0.2 | 0.5 | 0.4 | -0.3 | -0.1 |
| Smoking |  |  |  |  |  |  |  |  |  |  |  |  |  |  |
| Non-smoker | 79.4 | 2.4 | 2.3 | 0.0 | 2.5 | 2.3 | -0.2 | -0.4 | 4.7 | 4.4 | 2.4 | 2.2 | -0.9 | -0.9 |
| Daily smoking | 15.5 | -2.9 | -2.7 | 0.0 | -3.1 | -2.8 | 0.2 | 0.3 | -4.7 | -4.5 | -2.3 | -2.1 | 0.8 | 0.8 |
| Occasional smoking | 5.1 | 0.4 | 0.3 | 0.0 | 0.4 | 0.3 | 0.1 | 0.2 | -0.9 | -0.8 | -0.7 | -0.5 | 0.4 | 0.3 |
| Chronic diseases |  |  |  |  |  |  |  |  |  |  |  |  |  |  |
| No | 41.6 | 0.7 | 1.7 | 0.0 | 0.4 | 1.4 | -0.4 | 0.0 | -0.6 | 0.6 | -1.1 | -0.7 | 0.2 | 0.1 |
| Yes | 58.4 | -0.7 | -1.7 | 0.0 | -0.4 | -1.4 | 0.4 | 0.0 | 0.6 | -0.6 | 1.1 | 0.7 | -0.2 | -0.1 |
| Sport |  |  |  |  |  |  |  |  |  |  |  |  |  |  |
| No sporting activities | 22.8 | -3.2 | -3.7 | 0.0 | -5.9 | -6.3 | -3.0 | -0.1 | -6.9 | -7.6 | -4.7 | -2.0 | -0.6 | -0.3 |
| Less than 1 hour per week | 20.1 | -0.8 | -0.2 | 0.0 | -0.8 | -0.4 | 0.1 | 0.0 | -1.2 | -0.8 | -0.5 | -0.4 | 0.5 | 0.7 |
| 1 to less than 2 hours per week | 28.7 | 1.3 | 1.1 | 0.0 | 1.8 | 1.7 | 0.7 | 0.1 | 2.2 | 2.0 | 1.2 | 0.7 | -0.1 | -0.2 |
| 2 to less than 4 hours per week | 19.0 | 1.8 | 2.0 | 0.0 | 3.1 | 3.3 | 1.5 | 0.0 | 4.1 | 4.5 | 2.9 | 1.5 | 0.4 | 0.0 |
| 4 hours per week and more | 9.3 | 1.2 | 1.1 | 0.0 | 2.0 | 2.0 | 0.9 | 0.0 | 2.0 | 2.0 | 1.2 | 0.3 | -0.1 | -0.2 |
| KWsubj\_k |  |  |  |  |  |  |  |  |  |  |  |  |  |  |
| Scale value 1 to 3 | 29.9 | -0.3 | -0.5 | 0.0 | -0.7 | -0.9 | -0.4 | 0.2 | -0.7 | -1.0 | -0.7 | -0.1 | -0.1 | -0.3 |
| Scale value 4 to 7 | 53.3 | -0.1 | 0.0 | 0.0 | -0.1 | 0.1 | 0.2 | -0.1 | 0.1 | 0.2 | 0.6 | 0.4 | 0.4 | 0.6 |
| Scale value 8 to 10 | 16.8 | 0.5 | 0.6 | 0.0 | 1.0 | 1.0 | 0.2 | -0.2 | 0.7 | 0.9 | 0.0 | -0.4 | -0.4 | -0.5 |
| IAtermin |  |  |  |  |  |  |  |  |  |  |  |  |  |  |
| No | 53.9 | 0.1 | -0.4 | 0.0 | -0.6 | -1.1 | -0.9 | -0.1 | 0.8 | 0.0 | 0.1 | 1.0 | -0.1 | -0.1 |
| Yes | 36.1 | -0.4 | -0.1 | 0.0 | 0.2 | 0.4 | 0.7 | 0.2 | -1.1 | -0.7 | -0.3 | -0.9 | 0.0 | 0.1 |
| No need for examination or treatment | 10.0 | 0.4 | 0.8 | 0.0 | 0.7 | 1.1 | 0.3 | -0.1 | 0.5 | 1.1 | 0.4 | -0.1 | 0.1 | 0.0 |
|  |  |  |  |  |  |  |  |  |  |  |  |  |  |  |
| --- | --- | --- | --- | --- | --- | --- | --- | --- | --- | --- | --- | --- | --- | --- |
| aCS = Cross-Sectional | | | | | | | | | | | | | | |

## Male

Table 3.3: Standardized differences in percentage points compared to reference values for the recruitment survey, registration sample, and the sample of participants in Questionnaire C; calculated unweighted and weighted according to the respective weighting factors.

|  |  | Recruitment Survey | | | Registration | | | | Participation Questionnaire C | | | | | |
| --- | --- | --- | --- | --- | --- | --- | --- | --- | --- | --- | --- | --- | --- | --- |
| Parameter | Reference [%] | Unweighted | Design weight | CSa weight | Unweighted | Design weight | CSa weight | Drop-out Weight | Unweighted | Design weight | CSa weight (Recruitment Study) | Drop-out weight (Registration) | Drop-out weight | CSa weight |
| Agegrp |  |  |  |  |  |  |  |  |  |  |  |  |  |  |
| 18-29 yrs | 17.1 | -1.1 | -1.9 | -0.1 | -0.7 | -1.5 | 0.0 | -0.7 | -6.0 | -6.6 | -5.1 | -5.8 | -1.6 | -0.1 |
| 30-39 yrs | 16.6 | -2.0 | -1.2 | -0.1 | -1.0 | -0.1 | 1.0 | 0.1 | -3.2 | -2.3 | -1.4 | -2.4 | -0.1 | 0.4 |
| 40-49 yrs | 15.0 | -4.8 | -1.4 | -0.1 | -4.3 | -0.9 | 0.5 | 0.1 | -5.7 | -2.3 | -0.8 | -1.3 | -0.2 | -0.3 |
| 50-59 yrs | 17.9 | -0.7 | -0.3 | 0.0 | -0.4 | -0.3 | 0.0 | 0.0 | 0.2 | 0.6 | 1.0 | 0.8 | -0.2 | 0.4 |
| 60-69 yrs | 16.4 | 0.5 | 0.4 | -0.5 | 0.6 | 0.6 | -0.4 | -0.2 | 3.7 | 3.7 | 3.2 | 3.2 | 0.3 | -0.1 |
| 70-79 yrs | 10.0 | 5.7 | 3.9 | -0.5 | 4.8 | 3.0 | -1.3 | -0.7 | 8.1 | 6.1 | 1.8 | 2.5 | 0.0 | -0.2 |
| 80+ yrs | 7.0 | 3.7 | 1.5 | 1.7 | 1.9 | -0.5 | -0.3 | 1.8 | 3.9 | 1.3 | 1.8 | 4.0 | 2.6 | -0.2 |
| Federal state |  |  |  |  |  |  |  |  |  |  |  |  |  |  |
| Schleswig-Holstein | 3.5 | 12.2 | -0.1 | 0.0 | 12.3 | -0.1 | 0.1 | 0.2 | 12.1 | -0.4 | -0.1 | 0.0 | 0.4 | 0.0 |
| Hamburg | 2.2 | 3.3 | -0.5 | 0.0 | 3.7 | -0.2 | 0.4 | 0.3 | 3.4 | -0.7 | -0.1 | -0.2 | 0.2 | 0.0 |
| Lower Saxony | 9.6 | -3.6 | 0.1 | 0.1 | -3.9 | -0.2 | -0.1 | -0.1 | -3.7 | 0.0 | -0.2 | -0.2 | -0.3 | 0.0 |
| Bremen | 0.8 | 8.3 | -0.1 | 0.0 | 8.3 | -0.1 | -0.1 | 0.0 | 8.4 | -0.2 | -0.2 | -0.3 | 0.1 | 0.0 |
| North Rhine-Westphalia | 21.2 | -7.3 | -0.2 | 0.0 | -7.3 | 0.0 | 0.2 | -0.2 | -6.8 | 0.5 | 0.7 | 0.3 | 0.3 | 0.0 |
| Hesse | 7.6 | -4.1 | 0.4 | 0.0 | -3.7 | 0.7 | 0.1 | -0.1 | -4.8 | -0.7 | -1.9 | -2.0 | -2.2 | -0.1 |
| Rhineland-Palatinate | 5.0 | -3.3 | 0.3 | 0.0 | -2.9 | 0.7 | 0.5 | 0.4 | -2.6 | 1.1 | 0.8 | 0.7 | 0.6 | 0.0 |
| Baden-Württemberg | 13.4 | -5.9 | -0.3 | 0.0 | -5.5 | 0.0 | 0.2 | 0.1 | -5.6 | -0.1 | 0.0 | -0.2 | -0.8 | 0.0 |
| Bavaria | 16.0 | -5.8 | -0.2 | 0.0 | -5.9 | -0.4 | -0.3 | -0.2 | -5.2 | 0.5 | 1.1 | 1.4 | 1.1 | 0.0 |
| Saarland | 1.2 | 7.2 | 0.1 | 0.0 | 7.3 | 0.1 | 0.0 | 0.1 | 7.7 | 0.4 | 0.2 | 0.2 | 0.3 | 0.0 |
| Berlin | 4.4 | 10.7 | 0.1 | 0.0 | 11.0 | 0.2 | 0.3 | 0.2 | 10.4 | -0.3 | -0.3 | -0.4 | 0.7 | 0.0 |
| Brandenburg | 3.1 | 0.7 | 0.0 | -0.1 | 0.5 | -0.2 | -0.1 | 0.0 | 0.7 | -0.1 | 0.2 | 0.4 | 0.5 | 0.1 |
| Mecklenburg-Western Pomerania | 2.0 | 3.7 | -0.2 | 0.0 | 3.1 | -0.6 | -0.4 | -0.3 | 3.1 | -0.7 | -0.5 | -0.3 | -0.4 | 0.0 |
| Saxony | 4.9 | -2.8 | 0.0 | 0.0 | -3.0 | -0.2 | -0.3 | 0.0 | -3.0 | 0.0 | -0.1 | 0.2 | 0.1 | 0.0 |
| Saxony-Anhalt | 2.6 | 2.2 | 0.3 | 0.0 | 1.6 | -0.1 | -0.3 | 0.1 | 1.3 | -0.4 | -0.6 | -0.1 | -0.1 | 0.0 |
| Thuringia | 2.6 | 2.4 | 0.3 | 0.0 | 1.7 | -0.3 | -0.6 | -0.2 | 1.8 | -0.4 | -0.8 | -0.4 | -0.4 | 0.0 |
| German |  |  |  |  |  |  |  |  |  |  |  |  |  |  |
| Yes | 84.1 | 15.7 | 15.3 | 13.9 | 17.9 | 17.5 | 16.7 | 14.3 | 21.1 | 20.9 | 20.4 | 18.9 | 16.1 | 16.0 |
| No | 15.9 | -15.7 | -15.3 | -13.9 | -17.9 | -17.5 | -16.7 | -14.3 | -21.1 | -20.9 | -20.4 | -18.9 | -16.1 | -16.0 |
| BIK |  |  |  |  |  |  |  |  |  |  |  |  |  |  |
| BIK 1 | 10.9 | -0.9 | -0.4 | 0.0 | -1.5 | -1.0 | -0.6 | -0.1 | -1.9 | -1.3 | -0.7 | -0.1 | 0.3 | -0.1 |
| BIK 2 | 34.7 | -1.1 | 1.2 | -0.1 | -1.6 | 0.7 | -0.7 | 0.0 | -1.0 | 1.4 | -0.2 | 0.5 | -0.3 | -0.1 |
| BIK 3 | 26.6 | -2.5 | -0.9 | -0.3 | -2.2 | -0.6 | 0.0 | -0.3 | -2.1 | -0.5 | 0.1 | -0.3 | -0.3 | -0.1 |
| BIK 4 | 27.7 | 4.1 | -0.1 | 0.4 | 4.8 | 0.5 | 1.2 | 0.5 | 4.3 | -0.1 | 0.6 | -0.1 | 0.5 | 0.3 |
| Education |  |  |  |  |  |  |  |  |  |  |  |  |  |  |
| Low | 34.9 | -11.5 | -11.2 | -1.3 | -14.7 | -14.6 | -5.0 | -1.4 | -15.2 | -14.9 | -5.9 | -2.3 | -2.0 | -1.5 |
| Medium | 43.0 | 0.9 | 1.0 | 1.3 | 1.3 | 1.5 | 2.7 | 1.3 | -0.9 | -1.0 | 1.0 | -0.2 | 1.1 | 1.0 |
| High | 22.1 | 10.8 | 10.5 | 0.0 | 13.3 | 13.0 | 2.5 | 0.1 | 16.0 | 15.8 | 5.3 | 2.8 | 1.0 | 0.5 |
| Household size |  |  |  |  |  |  |  |  |  |  |  |  |  |  |
| Single-person household | 24.8 | -5.4 | -6.3 | 0.1 | -5.8 | -6.7 | -0.2 | 0.2 | -6.7 | -7.3 | -1.3 | -0.9 | -0.1 | 0.0 |
| Multi-person household | 75.2 | 5.4 | 6.3 | -0.1 | 5.8 | 6.7 | 0.2 | -0.2 | 6.7 | 7.3 | 1.3 | 0.9 | 0.1 | 0.0 |
| PAbmi\_k2 |  |  |  |  |  |  |  |  |  |  |  |  |  |  |
| Normal weight (18.5 <= BMI < 25) | 34.4 | 1.4 | 0.8 | 0.0 | 1.8 | 1.3 | 0.2 | -0.1 | 1.5 | 1.0 | 0.2 | -0.2 | 0.0 | 0.1 |
| Underweight (BMI < 18.5) | 1.1 | 0.0 | -0.5 | 0.0 | -0.3 | -0.7 | -0.4 | -0.4 | -1.3 | -1.7 | -1.2 | -1.3 | -0.3 | 0.0 |
| Overweight (25 <= BMI < 30) | 41.9 | 0.2 | 0.6 | 0.0 | -0.1 | 0.4 | -0.2 | 0.1 | 0.7 | 1.0 | 0.3 | 0.5 | -0.1 | -0.4 |
| Obesity (BMI >= 30) | 22.6 | -1.9 | -1.5 | 0.0 | -2.0 | -1.7 | 0.0 | 0.1 | -2.2 | -2.0 | -0.3 | -0.2 | 0.2 | 0.4 |
| Self-rated health |  |  |  |  |  |  |  |  |  |  |  |  |  |  |
| Very good/good/fair | 93.5 | 0.3 | 0.7 | 0.0 | 1.6 | 2.0 | 1.3 | 0.2 | 2.2 | 2.7 | 1.9 | 0.9 | 0.0 | 0.8 |
| Bad/very bad | 6.5 | -0.3 | -0.7 | 0.0 | -1.6 | -2.0 | -1.3 | -0.2 | -2.2 | -2.7 | -1.9 | -0.9 | 0.0 | -0.8 |
| Self-rated mental health |  |  |  |  |  |  |  |  |  |  |  |  |  |  |
| Excellent/very good/good | 81.4 | 1.6 | 1.8 | 0.0 | 2.0 | 2.1 | 0.1 | -0.2 | 3.4 | 3.3 | 1.5 | 1.1 | -0.1 | 0.2 |
| fair/poor | 18.6 | -1.6 | -1.8 | 0.0 | -2.0 | -2.1 | -0.1 | 0.2 | -3.4 | -3.3 | -1.5 | -1.1 | 0.1 | -0.2 |
| Paying attention to health |  |  |  |  |  |  |  |  |  |  |  |  |  |  |
| Not at all/less strong/moderate | 56.1 | -1.4 | -1.3 | 0.0 | -1.9 | -1.9 | -0.4 | 0.1 | -3.1 | -3.0 | -1.3 | -0.7 | 0.3 | 0.6 |
| Strong/very strong | 43.9 | 1.4 | 1.3 | 0.0 | 1.9 | 1.9 | 0.4 | -0.1 | 3.1 | 3.0 | 1.3 | 0.7 | -0.3 | -0.6 |
| Satisfaction: Life in general |  |  |  |  |  |  |  |  |  |  |  |  |  |  |
| Scale value 1 to 3 | 8.6 | -1.8 | -1.6 | 0.0 | -2.2 | -2.0 | -0.3 | 0.0 | -3.4 | -2.9 | -1.3 | -1.0 | 0.2 | -0.1 |
| Scale value 4 to 7 | 44.2 | -1.4 | -1.6 | 0.0 | -2.0 | -2.3 | -0.7 | -0.1 | -3.7 | -3.6 | -2.1 | -1.5 | -0.4 | -0.2 |
| Scale value 8 to 10 | 47.2 | 2.3 | 2.5 | 0.0 | 3.2 | 3.3 | 0.8 | 0.1 | 5.4 | 5.1 | 2.8 | 2.1 | 0.3 | 0.2 |
| Red meat |  |  |  |  |  |  |  |  |  |  |  |  |  |  |
| Never | 5.8 | 0.5 | -0.2 | 0.0 | 0.8 | 0.1 | 0.3 | 0.1 | 1.1 | 0.4 | 0.8 | 0.7 | 1.1 | 1.3 |
| Daily or several times a day | 4.4 | -1.7 | -1.0 | 0.0 | -2.4 | -1.6 | -0.5 | -0.1 | -3.8 | -3.0 | -2.2 | -1.8 | -0.9 | -0.6 |
| 4 to 6 times per week | 17.3 | -1.5 | -0.3 | 0.0 | -1.6 | -0.4 | 0.1 | 0.0 | -2.6 | -1.5 | -0.9 | -1.0 | 0.1 | 0.4 |
| 1 to 3 times per week | 49.9 | 0.5 | 0.6 | 0.0 | 0.3 | 0.4 | -0.2 | 0.0 | 0.8 | 1.0 | 0.1 | 0.3 | -0.6 | -0.8 |
| Less than once per week | 22.6 | 1.3 | 0.2 | 0.0 | 1.7 | 0.6 | 0.2 | 0.0 | 2.4 | 1.3 | 1.2 | 1.0 | 0.4 | 0.1 |
| Sausage products |  |  |  |  |  |  |  |  |  |  |  |  |  |  |
| Never | 7.2 | -0.1 | -0.3 | 0.0 | 0.2 | -0.1 | 0.1 | -0.1 | -0.7 | -1.0 | -1.0 | -1.5 | -0.4 | -0.1 |
| Daily or several times a day | 13.8 | -0.3 | -0.7 | 0.0 | -0.9 | -1.1 | -0.4 | 0.0 | -0.9 | -1.4 | -0.6 | -0.1 | -0.2 | -0.1 |
| 4 to 6 times per week | 25.1 | -0.3 | 0.1 | 0.0 | 0.1 | 0.4 | 0.3 | 0.0 | 0.8 | 1.2 | 1.3 | 1.2 | 0.3 | 0.4 |
| 1 to 3 times per week | 35.4 | 0.0 | 0.3 | 0.0 | -0.1 | 0.1 | 0.0 | 0.0 | 0.2 | 0.6 | 0.3 | 0.4 | 0.3 | 0.0 |
| Less than once per week | 18.4 | 0.7 | 0.3 | 0.0 | 0.8 | 0.4 | 0.0 | 0.0 | 0.2 | -0.3 | -0.7 | -0.8 | -0.3 | -0.4 |
| Smoking |  |  |  |  |  |  |  |  |  |  |  |  |  |  |
| Non-smoker | 72.8 | 4.4 | 3.9 | 0.0 | 5.4 | 5.0 | 1.1 | 0.4 | 9.9 | 9.4 | 6.4 | 5.8 | 2.1 | 1.9 |
| Daily smoking | 20.4 | -4.7 | -4.1 | 0.0 | -5.7 | -5.2 | -1.1 | -0.4 | -9.7 | -9.2 | -6.0 | -5.6 | -2.3 | -2.1 |
| Occasional smoking | 6.8 | -0.3 | -0.4 | 0.0 | -0.5 | -0.6 | -0.3 | -0.1 | -2.2 | -2.2 | -1.8 | -1.5 | 0.0 | 0.1 |
| Chronic diseases |  |  |  |  |  |  |  |  |  |  |  |  |  |  |
| No | 48.4 | -0.6 | -0.1 | 0.0 | -0.8 | -0.4 | -0.5 | -0.2 | -3.4 | -2.8 | -2.9 | -2.7 | -1.0 | -0.3 |
| Yes | 51.6 | 0.6 | 0.1 | 0.0 | 0.8 | 0.4 | 0.5 | 0.2 | 3.4 | 2.8 | 2.9 | 2.7 | 1.0 | 0.3 |
| Sport |  |  |  |  |  |  |  |  |  |  |  |  |  |  |
| No sporting activities | 23.3 | -1.8 | -2.4 | 0.0 | -3.3 | -4.0 | -1.7 | 0.0 | -4.0 | -4.8 | -2.6 | -1.1 | -0.5 | -0.7 |
| Less than 1 hour per week | 19.3 | -1.0 | -0.8 | 0.0 | -1.3 | -1.1 | -0.2 | 0.0 | -1.9 | -1.7 | -0.9 | -0.6 | -0.3 | -0.2 |
| 1 to less than 2 hours per week | 24.1 | 0.5 | 0.6 | 0.0 | 0.7 | 0.8 | 0.3 | 0.1 | 1.5 | 1.6 | 1.1 | 1.0 | 0.5 | 0.3 |
| 2 to less than 4 hours per week | 18.8 | 1.6 | 1.7 | 0.0 | 2.4 | 2.5 | 0.8 | 0.0 | 3.4 | 3.7 | 1.9 | 1.2 | 0.3 | 0.5 |
| 4 hours per week and more | 14.6 | 0.8 | 1.1 | 0.0 | 1.7 | 1.9 | 1.0 | 0.0 | 1.0 | 1.3 | 0.5 | -0.6 | 0.0 | 0.2 |
| KWsubj\_k |  |  |  |  |  |  |  |  |  |  |  |  |  |  |
| Scale value 1 to 3 | 40.0 | 0.1 | 0.0 | 0.0 | -0.3 | -0.4 | -0.4 | -0.2 | -0.3 | -0.4 | -0.6 | -0.3 | 0.0 | 0.0 |
| Scale value 4 to 7 | 44.6 | -0.1 | -0.1 | 0.0 | 0.0 | 0.0 | 0.2 | 0.1 | 0.1 | -0.1 | 0.2 | 0.3 | 0.1 | 0.1 |
| Scale value 8 to 10 | 15.4 | -0.1 | 0.1 | 0.0 | 0.5 | 0.6 | 0.3 | 0.1 | 0.4 | 0.7 | 0.4 | 0.0 | -0.1 | -0.1 |
| IAtermin |  |  |  |  |  |  |  |  |  |  |  |  |  |  |
| No | 55.6 | 2.0 | 1.1 | 0.0 | 2.0 | 1.2 | -0.1 | 0.1 | 4.3 | 3.4 | 2.1 | 2.2 | 0.5 | 0.0 |
| Yes | 30.9 | -1.4 | -0.9 | 0.0 | -1.6 | -1.1 | -0.1 | -0.1 | -3.1 | -2.7 | -1.6 | -1.6 | -0.4 | -0.1 |
| No need for examination or treatment | 13.5 | -1.0 | -0.4 | 0.0 | -0.7 | -0.2 | 0.2 | 0.0 | -2.1 | -1.4 | -0.9 | -1.1 | -0.2 | 0.2 |
|  |  |  |  |  |  |  |  |  |  |  |  |  |  |  |
| --- | --- | --- | --- | --- | --- | --- | --- | --- | --- | --- | --- | --- | --- | --- |
| aCS = Cross-Sectional | | | | | | | | | | | | | | |

# Questionnaire D

## Female and male

Table 4.1: Standardized differences in percentage points compared to reference values for the recruitment survey, registration sample, and the sample of participants in Questionnaire D; calculated unweighted and weighted according to the respective weighting factors.

|  |  | Recruitment Survey | | | Registration | | | | Participation Questionnaire D | | | | | |
| --- | --- | --- | --- | --- | --- | --- | --- | --- | --- | --- | --- | --- | --- | --- |
| Parameter | Reference [%] | Unweighted | Design weight | CSa weight | Unweighted | Design weight | CSa weight | Drop-out Weight | Unweighted | Design weight | CSa weight (Recruitment Study) | Drop-out weight (Registration) | Drop-out weight | CSa weight |
| sex |  |  |  |  |  |  |  |  |  |  |  |  |  |  |
| Male | 48.9 | -2.0 | -1.9 | 0.0 | -2.2 | -2.1 | 0.0 | 0.0 | -3.6 | -3.5 | -1.9 | -2.0 | -0.5 | 0.0 |
| Female | 51.1 | 2.0 | 1.9 | 0.0 | 2.2 | 2.1 | 0.0 | 0.0 | 3.6 | 3.5 | 1.9 | 2.0 | 0.5 | 0.0 |
| Agegrp |  |  |  |  |  |  |  |  |  |  |  |  |  |  |
| 18-29 yrs | 16.0 | 0.6 | -0.3 | -0.1 | 1.7 | 0.7 | 0.9 | -0.3 | -2.1 | -2.7 | -2.9 | -4.1 | -0.7 | 0.0 |
| 30-39 yrs | 15.8 | -0.9 | -0.1 | 0.0 | 0.3 | 1.1 | 1.4 | 0.1 | -1.7 | -0.9 | -0.8 | -2.2 | 0.0 | 0.3 |
| 40-49 yrs | 14.7 | -3.8 | -0.1 | 0.1 | -3.3 | 0.4 | 0.7 | 0.1 | -4.2 | -0.5 | -0.3 | -1.1 | 0.0 | -0.3 |
| 50-59 yrs | 17.6 | 0.4 | 0.6 | 0.4 | 0.8 | 0.9 | 0.7 | 0.4 | 1.6 | 1.7 | 1.7 | 1.3 | 0.4 | 0.4 |
| 60-69 yrs | 16.5 | -0.1 | -0.1 | -0.1 | -0.1 | -0.2 | -0.2 | 0.0 | 2.5 | 2.3 | 2.7 | 2.8 | 0.2 | -0.1 |
| 70-79 yrs | 10.7 | 3.5 | 1.7 | -0.2 | 2.4 | 0.4 | -1.4 | -0.1 | 4.8 | 2.8 | 1.0 | 2.3 | 0.0 | 0.0 |
| 80+ yrs | 8.7 | 0.6 | -1.8 | -0.1 | -2.3 | -4.6 | -3.2 | -0.1 | -1.1 | -3.5 | -2.0 | 1.2 | 0.1 | -0.4 |
| Federal state |  |  |  |  |  |  |  |  |  |  |  |  |  |  |
| Schleswig-Holstein | 3.5 | 12.5 | 0.0 | 0.0 | 12.6 | 0.1 | 0.2 | 0.1 | 12.9 | 0.2 | 0.2 | 0.2 | 0.4 | 0.0 |
| Hamburg | 2.2 | 3.9 | -0.1 | 0.0 | 4.3 | 0.3 | 0.4 | 0.2 | 4.3 | 0.4 | 0.6 | 0.4 | 0.6 | 0.0 |
| Lower Saxony | 9.6 | -3.8 | 0.1 | 0.1 | -3.9 | 0.0 | 0.0 | -0.1 | -3.9 | -0.2 | -0.2 | -0.3 | -0.3 | 0.0 |
| Bremen | 0.8 | 8.3 | -0.1 | 0.0 | 8.6 | 0.1 | 0.1 | 0.1 | 8.3 | -0.1 | -0.1 | -0.2 | -0.1 | 0.0 |
| North Rhine-Westphalia | 21.4 | -7.2 | 0.0 | 0.0 | -7.2 | 0.2 | 0.1 | -0.2 | -7.4 | 0.0 | -0.2 | -0.5 | -0.6 | 0.0 |
| Hesse | 7.6 | -4.4 | 0.0 | 0.0 | -4.5 | -0.2 | -0.2 | -0.2 | -5.2 | -1.0 | -1.0 | -1.0 | -0.9 | 0.0 |
| Rhineland-Palatinate | 4.9 | -3.5 | 0.0 | 0.0 | -3.2 | 0.5 | 0.5 | 0.5 | -2.8 | 1.0 | 1.1 | 1.2 | 1.2 | 0.0 |
| Baden-Württemberg | 13.3 | -5.9 | -0.1 | 0.0 | -5.6 | 0.1 | 0.1 | -0.1 | -5.9 | -0.2 | -0.3 | -0.4 | -0.9 | 0.0 |
| Bavaria | 15.9 | -5.7 | 0.0 | 0.0 | -5.8 | -0.1 | -0.2 | 0.0 | -5.1 | 0.8 | 0.7 | 0.9 | 0.7 | 0.0 |
| Saarland | 1.2 | 7.1 | 0.0 | 0.0 | 7.0 | 0.0 | 0.0 | 0.1 | 7.2 | 0.1 | 0.1 | 0.1 | 0.1 | 0.0 |
| Berlin | 4.5 | 10.5 | 0.0 | 0.0 | 10.9 | 0.3 | 0.4 | 0.3 | 10.5 | 0.0 | 0.2 | 0.0 | 0.8 | 0.0 |
| Brandenburg | 3.1 | 0.8 | 0.0 | -0.1 | 0.5 | -0.2 | -0.2 | -0.1 | 0.6 | -0.2 | -0.1 | -0.1 | 0.1 | 0.0 |
| Mecklenburg-Western Pomerania | 2.0 | 4.0 | 0.0 | 0.0 | 3.5 | -0.3 | -0.3 | -0.2 | 3.5 | -0.4 | -0.5 | -0.5 | -0.4 | 0.0 |
| Saxony | 4.9 | -2.6 | 0.0 | 0.0 | -2.9 | -0.4 | -0.3 | 0.0 | -2.6 | -0.1 | -0.1 | 0.3 | 0.0 | 0.0 |
| Saxony-Anhalt | 2.6 | 1.8 | 0.0 | 0.0 | 1.4 | -0.3 | -0.2 | 0.2 | 1.4 | -0.4 | -0.1 | 0.3 | 0.3 | 0.0 |
| Thuringia | 2.5 | 2.0 | 0.0 | 0.0 | 1.4 | -0.5 | -0.5 | -0.1 | 1.5 | -0.5 | -0.5 | 0.0 | -0.1 | 0.0 |
| German |  |  |  |  |  |  |  |  |  |  |  |  |  |  |
| Yes | 85.0 | 14.2 | 13.6 | 12.5 | 16.1 | 15.7 | 15.0 | 12.6 | 18.9 | 18.5 | 18.1 | 16.3 | 13.4 | 13.5 |
| No | 15.0 | -14.2 | -13.6 | -12.5 | -16.1 | -15.7 | -15.0 | -12.6 | -18.9 | -18.5 | -18.1 | -16.3 | -13.4 | -13.5 |
| BIK |  |  |  |  |  |  |  |  |  |  |  |  |  |  |
| BIK 1 | 10.8 | -0.8 | -0.4 | 0.0 | -1.4 | -0.9 | -0.5 | 0.0 | -1.7 | -1.3 | -0.7 | -0.1 | 0.0 | 0.0 |
| BIK 2 | 34.6 | -1.2 | 1.1 | 0.0 | -1.8 | 0.6 | -0.6 | 0.1 | -1.4 | 1.1 | -0.2 | 0.5 | 0.1 | 0.0 |
| BIK 3 | 26.8 | -2.4 | -0.6 | 0.0 | -2.2 | -0.4 | 0.2 | -0.1 | -2.2 | -0.5 | 0.0 | -0.4 | -0.3 | 0.0 |
| BIK 4 | 27.9 | 4.1 | -0.3 | 0.0 | 4.9 | 0.3 | 0.7 | 0.0 | 4.7 | 0.2 | 0.7 | 0.0 | 0.2 | 0.0 |
| Education |  |  |  |  |  |  |  |  |  |  |  |  |  |  |
| Low | 34.4 | -12.8 | -12.8 | -1.3 | -16.8 | -16.8 | -5.8 | -1.5 | -17.5 | -17.5 | -7.1 | -2.9 | -2.7 | -1.2 |
| Medium | 45.3 | 2.3 | 2.5 | 1.4 | 3.3 | 3.5 | 3.4 | 1.5 | 2.5 | 2.9 | 3.4 | 1.5 | 2.1 | 1.1 |
| High | 20.3 | 10.7 | 10.5 | -0.1 | 13.2 | 12.9 | 2.3 | 0.0 | 14.5 | 14.2 | 3.8 | 1.4 | 0.5 | 0.1 |
| Household size |  |  |  |  |  |  |  |  |  |  |  |  |  |  |
| Single-person household | 25.4 | -3.5 | -4.8 | 0.2 | -4.6 | -5.8 | -0.9 | 0.1 | -4.4 | -5.5 | -0.9 | 0.3 | 0.1 | 0.5 |
| Multi-person household | 74.6 | 3.5 | 4.8 | -0.2 | 4.6 | 5.8 | 0.9 | -0.1 | 4.4 | 5.5 | 0.9 | -0.3 | -0.1 | -0.5 |
| PAbmi\_k2 |  |  |  |  |  |  |  |  |  |  |  |  |  |  |
| Normal weight (18.5 <= BMI < 25) | 40.7 | 2.0 | 1.9 | 0.0 | 2.5 | 2.5 | 0.4 | -0.1 | 2.9 | 3.0 | 1.1 | 0.6 | 0.0 | -0.1 |
| Underweight (BMI < 18.5) | 2.0 | 0.2 | -0.1 | 0.0 | 0.2 | -0.1 | -0.1 | -0.1 | 0.2 | -0.1 | -0.2 | -0.2 | 0.1 | 0.2 |
| Overweight (25 <= BMI < 30) | 35.2 | -0.3 | -0.3 | 0.0 | -0.7 | -0.7 | -0.4 | 0.1 | -0.6 | -0.7 | -0.3 | 0.1 | 0.2 | 0.2 |
| Obesity (BMI >= 30) | 22.1 | -2.2 | -1.9 | 0.0 | -2.3 | -2.1 | -0.1 | 0.0 | -2.9 | -2.8 | -0.9 | -0.7 | -0.3 | -0.2 |
| Self-rated health |  |  |  |  |  |  |  |  |  |  |  |  |  |  |
| Very good/good/fair | 93.1 | 0.9 | 1.3 | 0.0 | 2.4 | 2.7 | 1.5 | 0.0 | 2.6 | 2.9 | 1.7 | 0.3 | 0.1 | 0.3 |
| Bad/very bad | 6.9 | -0.9 | -1.3 | 0.0 | -2.4 | -2.7 | -1.5 | 0.0 | -2.6 | -2.9 | -1.7 | -0.3 | -0.1 | -0.3 |
| Self-rated mental health |  |  |  |  |  |  |  |  |  |  |  |  |  |  |
| Excellent/very good/good | 78.5 | 1.3 | 1.6 | 0.0 | 1.7 | 1.9 | 0.3 | 0.0 | 2.5 | 2.6 | 1.0 | 0.6 | -0.1 | 0.0 |
| fair/poor | 21.5 | -1.3 | -1.6 | 0.0 | -1.7 | -1.9 | -0.3 | 0.0 | -2.5 | -2.6 | -1.0 | -0.6 | 0.1 | 0.0 |
| Paying attention to health |  |  |  |  |  |  |  |  |  |  |  |  |  |  |
| Not at all/less strong/moderate | 51.6 | -1.7 | -1.6 | 0.0 | -2.2 | -2.2 | -0.5 | 0.0 | -3.6 | -3.7 | -2.1 | -1.5 | -0.2 | 0.1 |
| Strong/very strong | 48.4 | 1.7 | 1.6 | 0.0 | 2.2 | 2.2 | 0.5 | 0.0 | 3.6 | 3.7 | 2.1 | 1.5 | 0.2 | -0.1 |
| Satisfaction: Life in general |  |  |  |  |  |  |  |  |  |  |  |  |  |  |
| Scale value 1 to 3 | 8.5 | -1.4 | -1.5 | 0.0 | -2.0 | -2.1 | -0.5 | -0.1 | -2.9 | -2.9 | -1.5 | -1.1 | -0.4 | -0.3 |
| Scale value 4 to 7 | 45.1 | -0.9 | -1.0 | 0.0 | -1.3 | -1.4 | -0.5 | 0.1 | -2.7 | -2.8 | -1.9 | -1.4 | -0.4 | -0.5 |
| Scale value 8 to 10 | 46.4 | 1.7 | 1.8 | 0.0 | 2.4 | 2.5 | 0.7 | 0.0 | 4.2 | 4.3 | 2.7 | 1.9 | 0.6 | 0.7 |
| Red meat |  |  |  |  |  |  |  |  |  |  |  |  |  |  |
| Never | 9.3 | 1.2 | 0.6 | 0.0 | 1.7 | 1.0 | 0.4 | 0.0 | 1.7 | 1.1 | 0.5 | 0.2 | 0.2 | 0.2 |
| Daily or several times a day | 2.8 | -1.6 | -1.1 | 0.0 | -2.1 | -1.7 | -0.4 | 0.0 | -3.4 | -2.8 | -2.0 | -1.7 | -0.7 | -0.6 |
| 4 to 6 times per week | 12.2 | -1.4 | -0.4 | 0.0 | -1.6 | -0.5 | 0.1 | 0.0 | -2.4 | -1.4 | -0.7 | -0.6 | 0.0 | 0.3 |
| 1 to 3 times per week | 46.1 | -0.6 | -0.1 | 0.0 | -1.1 | -0.5 | -0.4 | 0.0 | -1.2 | -0.5 | -0.6 | -0.3 | -0.3 | -0.4 |
| Less than once per week | 29.6 | 1.4 | 0.4 | 0.0 | 1.9 | 0.9 | 0.3 | 0.0 | 2.9 | 1.7 | 1.5 | 1.3 | 0.4 | 0.3 |
| Sausage products |  |  |  |  |  |  |  |  |  |  |  |  |  |  |
| Never | 10.3 | 1.5 | 1.1 | 0.0 | 2.1 | 1.6 | 0.4 | -0.1 | 1.7 | 1.2 | 0.0 | -0.6 | -0.3 | -0.4 |
| Daily or several times a day | 10.0 | -0.7 | -1.0 | 0.0 | -1.2 | -1.4 | -0.3 | 0.0 | -1.4 | -1.7 | -0.8 | -0.6 | -0.1 | 0.1 |
| 4 to 6 times per week | 19.4 | -0.6 | -0.2 | 0.0 | -0.3 | 0.1 | 0.4 | 0.0 | -0.1 | 0.3 | 0.5 | 0.2 | 0.0 | 0.0 |
| 1 to 3 times per week | 35.6 | -0.7 | -0.3 | 0.0 | -1.3 | -0.8 | -0.4 | 0.0 | -1.3 | -0.8 | -0.5 | 0.0 | -0.1 | -0.1 |
| Less than once per week | 24.8 | 0.8 | 0.4 | 0.0 | 1.0 | 0.6 | 0.1 | 0.1 | 1.3 | 0.9 | 0.6 | 0.6 | 0.3 | 0.4 |
| Smoking |  |  |  |  |  |  |  |  |  |  |  |  |  |  |
| Non-smoker | 76.2 | 3.5 | 3.2 | 0.0 | 4.1 | 3.8 | 0.5 | 0.0 | 7.4 | 7.0 | 4.3 | 3.9 | 0.9 | 0.8 |
| Daily smoking | 17.9 | -3.9 | -3.5 | 0.0 | -4.5 | -4.1 | -0.5 | -0.1 | -7.4 | -7.1 | -4.1 | -3.8 | -1.1 | -1.0 |
| Occasional smoking | 5.9 | -0.1 | -0.1 | 0.0 | -0.1 | -0.2 | -0.1 | 0.0 | -1.6 | -1.4 | -1.2 | -1.0 | 0.1 | 0.1 |
| Chronic diseases |  |  |  |  |  |  |  |  |  |  |  |  |  |  |
| No | 45.0 | -0.1 | 0.7 | 0.0 | -0.3 | 0.4 | -0.4 | -0.1 | -1.9 | -1.2 | -1.9 | -1.6 | -0.4 | -0.2 |
| Yes | 55.0 | 0.1 | -0.7 | 0.0 | 0.3 | -0.4 | 0.4 | 0.1 | 1.9 | 1.2 | 1.9 | 1.6 | 0.4 | 0.2 |
| Sport |  |  |  |  |  |  |  |  |  |  |  |  |  |  |
| No sporting activities | 23.0 | -2.6 | -3.1 | 0.0 | -4.7 | -5.2 | -2.3 | -0.1 | -5.5 | -6.0 | -3.4 | -1.2 | -0.2 | -0.1 |
| Less than 1 hour per week | 19.7 | -0.9 | -0.5 | 0.0 | -1.0 | -0.7 | -0.1 | 0.0 | -1.6 | -1.4 | -0.8 | -0.7 | -0.1 | -0.1 |
| 1 to less than 2 hours per week | 26.5 | 1.0 | 1.0 | 0.0 | 1.4 | 1.4 | 0.5 | 0.1 | 2.1 | 2.2 | 1.4 | 1.1 | 0.3 | 0.3 |
| 2 to less than 4 hours per week | 18.9 | 1.7 | 1.8 | 0.0 | 2.8 | 3.0 | 1.2 | 0.0 | 3.5 | 3.5 | 2.0 | 0.8 | -0.1 | -0.2 |
| 4 hours per week and more | 11.9 | 0.8 | 0.9 | 0.0 | 1.6 | 1.8 | 0.9 | 0.0 | 1.5 | 1.8 | 1.0 | 0.0 | 0.1 | 0.1 |
| KWsubj\_k |  |  |  |  |  |  |  |  |  |  |  |  |  |  |
| Scale value 1 to 3 | 34.8 | -0.3 | -0.4 | 0.0 | -0.8 | -0.9 | -0.4 | 0.0 | -0.5 | -1.0 | -0.6 | -0.2 | -0.1 | -0.1 |
| Scale value 4 to 7 | 49.0 | 0.1 | 0.1 | 0.0 | 0.1 | 0.2 | 0.2 | 0.0 | 0.1 | 0.3 | 0.4 | 0.4 | 0.3 | 0.4 |
| Scale value 8 to 10 | 16.1 | 0.3 | 0.4 | 0.0 | 0.8 | 0.8 | 0.3 | -0.1 | 0.5 | 0.8 | 0.2 | -0.2 | -0.4 | -0.5 |
| IAtermin |  |  |  |  |  |  |  |  |  |  |  |  |  |  |
| No | 54.8 | 0.9 | 0.3 | 0.0 | 0.6 | -0.1 | -0.5 | 0.0 | 2.2 | 1.5 | 1.0 | 1.5 | 0.0 | -0.1 |
| Yes | 33.5 | -0.7 | -0.3 | 0.0 | -0.5 | -0.2 | 0.3 | 0.0 | -1.7 | -1.3 | -0.8 | -1.1 | 0.0 | 0.0 |
| No need for examination or treatment | 11.7 | -0.4 | 0.1 | 0.0 | -0.1 | 0.3 | 0.3 | 0.0 | -1.0 | -0.4 | -0.3 | -0.7 | 0.0 | 0.1 |
|  |  |  |  |  |  |  |  |  |  |  |  |  |  |  |
| --- | --- | --- | --- | --- | --- | --- | --- | --- | --- | --- | --- | --- | --- | --- |
| aCS = Cross-Sectional | | | | | | | | | | | | | | |

## Female

Table 4.2: Standardized differences in percentage points compared to reference values for the recruitment survey, registration sample, and the sample of participants in Questionnaire D; calculated unweighted and weighted according to the respective weighting factors.

|  |  | Recruitment Survey | | | Registration | | | | Participation Questionnaire D | | | | | |
| --- | --- | --- | --- | --- | --- | --- | --- | --- | --- | --- | --- | --- | --- | --- |
| Parameter | Reference [%] | Unweighted | Design weight | CSa weight | Unweighted | Design weight | CSa weight | Drop-out Weight | Unweighted | Design weight | CSa weight (Recruitment Study) | Drop-out weight (Registration) | Drop-out weight | CSa weight |
| Agegrp |  |  |  |  |  |  |  |  |  |  |  |  |  |  |
| 18-29 yrs | 14.9 | 2.2 | 1.3 | -0.1 | 3.9 | 2.9 | 1.7 | 0.1 | 0.9 | -0.1 | -1.6 | -3.1 | -0.2 | 0.1 |
| 30-39 yrs | 15.1 | 0.2 | 0.9 | 0.2 | 1.5 | 2.3 | 1.9 | 0.1 | -0.4 | 0.8 | 0.2 | -1.7 | 0.3 | 0.3 |
| 40-49 yrs | 14.4 | -2.9 | 1.0 | 0.3 | -2.4 | 1.6 | 0.9 | 0.1 | -3.1 | 0.8 | 0.0 | -1.0 | 0.0 | -0.3 |
| 50-59 yrs | 17.2 | 1.3 | 1.4 | 0.7 | 1.9 | 1.9 | 1.3 | 0.8 | 2.6 | 2.6 | 2.1 | 1.5 | 0.8 | 0.5 |
| 60-69 yrs | 16.6 | -0.6 | -0.7 | 0.2 | -0.7 | -1.0 | 0.0 | 0.1 | 1.7 | 1.4 | 2.6 | 2.6 | 0.2 | -0.2 |
| 70-79 yrs | 11.4 | 1.5 | -0.4 | 0.0 | 0.1 | -1.9 | -1.5 | 0.3 | 2.1 | -0.1 | 0.4 | 2.4 | 0.4 | 0.1 |
| 80+ yrs | 10.4 | -2.2 | -4.6 | -1.5 | -6.0 | -8.2 | -5.8 | -1.6 | -5.2 | -7.5 | -5.1 | -1.0 | -1.7 | -0.6 |
| Federal state |  |  |  |  |  |  |  |  |  |  |  |  |  |  |
| Schleswig-Holstein | 3.5 | 12.7 | 0.1 | 0.0 | 12.9 | 0.3 | 0.2 | 0.0 | 13.3 | 0.5 | 0.4 | 0.2 | 0.3 | 0.0 |
| Hamburg | 2.3 | 4.4 | 0.3 | 0.0 | 4.8 | 0.7 | 0.3 | 0.1 | 4.8 | 0.9 | 0.5 | 0.3 | 0.5 | 0.0 |
| Lower Saxony | 9.6 | -3.9 | 0.0 | 0.1 | -3.9 | 0.1 | 0.2 | 0.0 | -4.0 | 0.0 | 0.0 | -0.2 | -0.3 | 0.0 |
| Bremen | 0.8 | 8.4 | 0.0 | -0.1 | 8.7 | 0.2 | 0.2 | 0.1 | 8.4 | 0.0 | -0.2 | -0.3 | -0.3 | 0.0 |
| North Rhine-Westphalia | 21.5 | -7.1 | 0.2 | 0.0 | -7.2 | 0.4 | 0.0 | -0.2 | -7.4 | 0.1 | -0.5 | -0.8 | -0.6 | 0.0 |
| Hesse | 7.6 | -4.8 | -0.4 | 0.0 | -5.4 | -1.0 | -0.5 | -0.4 | -6.2 | -2.1 | -1.4 | -1.3 | -1.0 | -0.1 |
| Rhineland-Palatinate | 4.9 | -3.8 | -0.3 | -0.1 | -3.4 | 0.2 | 0.5 | 0.6 | -3.2 | 0.4 | 0.6 | 0.9 | 1.0 | -0.1 |
| Baden-Württemberg | 13.2 | -5.8 | 0.1 | 0.0 | -5.7 | 0.1 | -0.1 | -0.2 | -5.8 | 0.0 | -0.1 | -0.2 | -0.8 | 0.0 |
| Bavaria | 15.8 | -5.6 | 0.1 | -0.1 | -5.6 | 0.2 | 0.0 | 0.1 | -4.9 | 1.1 | 1.0 | 1.2 | 0.9 | 0.0 |
| Saarland | 1.2 | 6.9 | 0.0 | 0.0 | 6.7 | -0.1 | -0.1 | 0.0 | 6.7 | -0.1 | 0.0 | 0.0 | 0.2 | 0.0 |
| Berlin | 4.5 | 10.2 | -0.2 | 0.0 | 10.8 | 0.3 | 0.6 | 0.3 | 10.4 | 0.0 | 0.2 | 0.0 | 0.6 | 0.0 |
| Brandenburg | 3.1 | 0.8 | 0.1 | -0.1 | 0.6 | -0.2 | -0.3 | -0.2 | 0.7 | 0.0 | 0.0 | 0.0 | 0.0 | 0.0 |
| Mecklenburg-Western Pomerania | 2.0 | 4.2 | 0.2 | 0.0 | 3.9 | 0.0 | -0.1 | 0.0 | 3.9 | -0.1 | -0.3 | -0.3 | -0.3 | 0.0 |
| Saxony | 4.9 | -2.5 | 0.0 | 0.1 | -2.8 | -0.5 | -0.4 | 0.0 | -2.6 | -0.1 | -0.2 | 0.1 | 0.0 | 0.0 |
| Saxony-Anhalt | 2.6 | 1.5 | -0.2 | 0.0 | 1.3 | -0.4 | -0.1 | 0.3 | 1.1 | -0.6 | -0.1 | 0.3 | 0.2 | 0.0 |
| Thuringia | 2.5 | 1.7 | -0.1 | 0.0 | 1.1 | -0.6 | -0.4 | 0.0 | 1.5 | -0.3 | 0.0 | 0.5 | 0.4 | 0.0 |
| German |  |  |  |  |  |  |  |  |  |  |  |  |  |  |
| Yes | 85.8 | 12.7 | 12.0 | 11.1 | 14.5 | 14.1 | 13.4 | 11.1 | 17.4 | 17.0 | 16.9 | 15.0 | 11.8 | 11.9 |
| No | 14.2 | -12.7 | -12.0 | -11.1 | -14.5 | -14.1 | -13.4 | -11.1 | -17.4 | -17.0 | -16.9 | -15.0 | -11.8 | -11.9 |
| BIK |  |  |  |  |  |  |  |  |  |  |  |  |  |  |
| BIK 1 | 10.7 | -0.8 | -0.4 | 0.0 | -1.4 | -0.8 | -0.4 | 0.2 | -1.5 | -1.2 | -0.5 | 0.2 | 0.1 | 0.1 |
| BIK 2 | 34.4 | -1.3 | 0.9 | 0.1 | -1.9 | 0.5 | -0.4 | 0.2 | -1.6 | 1.0 | -0.1 | 0.4 | 0.0 | -0.3 |
| BIK 3 | 26.9 | -2.3 | -0.3 | 0.2 | -2.2 | -0.2 | 0.5 | 0.1 | -2.0 | -0.3 | 0.5 | 0.1 | 0.4 | 0.7 |
| BIK 4 | 28.1 | 4.1 | -0.5 | -0.3 | 5.0 | 0.2 | 0.2 | -0.5 | 4.7 | 0.0 | 0.1 | -0.7 | -0.5 | -0.4 |
| Education |  |  |  |  |  |  |  |  |  |  |  |  |  |  |
| Low | 33.8 | -14.0 | -14.2 | -1.2 | -18.7 | -18.9 | -6.5 | -1.7 | -19.1 | -19.3 | -7.5 | -2.8 | -2.6 | -0.5 |
| Medium | 47.6 | 3.4 | 3.6 | 1.4 | 4.8 | 5.1 | 4.2 | 1.7 | 4.8 | 5.2 | 4.7 | 2.3 | 2.3 | 0.7 |
| High | 18.6 | 10.8 | 10.7 | -0.2 | 13.3 | 13.1 | 2.2 | -0.1 | 13.6 | 13.3 | 2.8 | 0.5 | 0.2 | -0.3 |
| Household size |  |  |  |  |  |  |  |  |  |  |  |  |  |  |
| Single-person household | 26.0 | -1.9 | -3.6 | 0.2 | -3.6 | -5.1 | -1.6 | 0.1 | -2.9 | -4.3 | -1.0 | 0.9 | 0.2 | 0.7 |
| Multi-person household | 74.0 | 1.9 | 3.6 | -0.2 | 3.6 | 5.1 | 1.6 | -0.1 | 2.9 | 4.3 | 1.0 | -0.9 | -0.2 | -0.7 |
| PAbmi\_k2 |  |  |  |  |  |  |  |  |  |  |  |  |  |  |
| Normal weight (18.5 <= BMI < 25) | 46.8 | 2.1 | 2.4 | 0.0 | 2.6 | 3.1 | 0.7 | 0.0 | 2.8 | 3.2 | 0.9 | 0.3 | -0.1 | -0.5 |
| Underweight (BMI < 18.5) | 2.9 | 0.2 | -0.1 | 0.0 | 0.2 | -0.1 | 0.1 | 0.0 | 0.1 | -0.1 | -0.3 | -0.4 | -0.1 | 0.2 |
| Overweight (25 <= BMI < 30) | 28.7 | -0.3 | -0.7 | 0.0 | -0.7 | -1.2 | -0.6 | 0.1 | -0.4 | -1.0 | -0.3 | 0.3 | 0.2 | 0.5 |
| Obesity (BMI >= 30) | 21.6 | -2.4 | -2.2 | 0.0 | -2.6 | -2.5 | -0.2 | -0.1 | -3.0 | -2.7 | -0.7 | -0.7 | -0.1 | 0.0 |
| Self-rated health |  |  |  |  |  |  |  |  |  |  |  |  |  |  |
| Very good/good/fair | 92.8 | 1.5 | 2.0 | 0.0 | 3.2 | 3.4 | 1.7 | -0.1 | 3.4 | 3.5 | 2.0 | 0.3 | -0.1 | 0.0 |
| Bad/very bad | 7.2 | -1.5 | -2.0 | 0.0 | -3.2 | -3.4 | -1.7 | 0.1 | -3.4 | -3.5 | -2.0 | -0.3 | 0.1 | 0.0 |
| Self-rated mental health |  |  |  |  |  |  |  |  |  |  |  |  |  |  |
| Excellent/very good/good | 75.6 | 1.3 | 1.7 | 0.0 | 1.7 | 2.1 | 0.6 | 0.2 | 2.8 | 3.0 | 1.6 | 1.2 | 0.4 | 0.2 |
| fair/poor | 24.3 | -1.3 | -1.7 | 0.0 | -1.7 | -2.1 | -0.6 | -0.2 | -2.8 | -3.0 | -1.6 | -1.2 | -0.4 | -0.2 |
| Paying attention to health |  |  |  |  |  |  |  |  |  |  |  |  |  |  |
| Not at all/less strong/moderate | 47.3 | -1.6 | -1.5 | 0.0 | -2.2 | -2.2 | -0.7 | -0.1 | -3.4 | -3.4 | -2.1 | -1.6 | -0.3 | -0.1 |
| Strong/very strong | 52.7 | 1.6 | 1.5 | 0.0 | 2.2 | 2.2 | 0.7 | 0.1 | 3.4 | 3.4 | 2.1 | 1.6 | 0.3 | 0.1 |
| Satisfaction: Life in general |  |  |  |  |  |  |  |  |  |  |  |  |  |  |
| Scale value 1 to 3 | 8.4 | -1.1 | -1.4 | 0.0 | -1.8 | -2.1 | -0.7 | -0.2 | -2.6 | -2.7 | -1.6 | -1.1 | -0.5 | -0.3 |
| Scale value 4 to 7 | 46.0 | -0.6 | -0.6 | 0.0 | -0.8 | -0.7 | -0.2 | 0.2 | -2.0 | -1.9 | -1.5 | -1.1 | -0.2 | -0.3 |
| Scale value 8 to 10 | 45.7 | 1.2 | 1.4 | 0.0 | 1.8 | 1.8 | 0.6 | -0.1 | 3.3 | 3.3 | 2.4 | 1.8 | 0.5 | 0.4 |
| Red meat |  |  |  |  |  |  |  |  |  |  |  |  |  |  |
| Never | 12.6 | 1.3 | 0.8 | 0.0 | 1.9 | 1.2 | 0.5 | 0.0 | 1.5 | 1.1 | 0.4 | 0.0 | 0.2 | 0.2 |
| Daily or several times a day | 1.2 | -1.0 | -1.0 | 0.0 | -1.4 | -1.4 | -0.2 | 0.0 | -2.4 | -2.4 | -2.0 | -1.8 | -1.5 | -1.4 |
| 4 to 6 times per week | 7.3 | -0.7 | 0.1 | 0.0 | -0.8 | -0.1 | 0.1 | 0.1 | -1.2 | -0.6 | -0.3 | -0.2 | 0.0 | 0.2 |
| 1 to 3 times per week | 42.6 | -1.3 | -0.5 | 0.0 | -2.0 | -1.0 | -0.6 | -0.1 | -2.0 | -0.9 | -0.8 | -0.4 | -0.3 | -0.4 |
| Less than once per week | 36.3 | 1.0 | 0.1 | 0.0 | 1.5 | 0.5 | 0.3 | 0.0 | 2.1 | 1.0 | 1.1 | 0.9 | 0.4 | 0.5 |
| Sausage products |  |  |  |  |  |  |  |  |  |  |  |  |  |  |
| Never | 13.2 | 2.3 | 1.7 | 0.0 | 3.1 | 2.4 | 0.6 | -0.1 | 2.6 | 1.9 | 0.3 | -0.5 | -0.1 | -0.2 |
| Daily or several times a day | 6.3 | -0.7 | -0.9 | 0.0 | -0.9 | -1.2 | -0.2 | -0.1 | -1.2 | -1.4 | -0.5 | -0.5 | -0.2 | -0.2 |
| 4 to 6 times per week | 13.9 | -0.4 | 0.1 | 0.0 | -0.1 | 0.4 | 0.5 | 0.1 | 0.2 | 0.8 | 0.8 | 0.4 | -0.2 | -0.2 |
| 1 to 3 times per week | 35.8 | -1.4 | -0.9 | 0.0 | -2.3 | -1.6 | -0.9 | 0.0 | -2.6 | -2.0 | -1.4 | -0.6 | -0.3 | -0.4 |
| Less than once per week | 30.8 | 0.4 | 0.0 | 0.0 | 0.6 | 0.2 | 0.2 | 0.1 | 1.1 | 0.8 | 0.9 | 0.9 | 0.6 | 0.8 |
| Smoking |  |  |  |  |  |  |  |  |  |  |  |  |  |  |
| Non-smoker | 79.4 | 2.4 | 2.3 | 0.0 | 2.5 | 2.3 | -0.2 | -0.4 | 5.3 | 4.9 | 2.9 | 2.8 | -0.1 | -0.2 |
| Daily smoking | 15.5 | -2.9 | -2.7 | 0.0 | -3.1 | -2.8 | 0.2 | 0.3 | -5.4 | -5.1 | -2.6 | -2.5 | 0.1 | 0.3 |
| Occasional smoking | 5.1 | 0.4 | 0.3 | 0.0 | 0.4 | 0.3 | 0.1 | 0.2 | -0.9 | -0.7 | -1.0 | -0.9 | 0.1 | 0.0 |
| Chronic diseases |  |  |  |  |  |  |  |  |  |  |  |  |  |  |
| No | 41.6 | 0.7 | 1.7 | 0.0 | 0.4 | 1.4 | -0.4 | 0.0 | -0.7 | 0.1 | -1.6 | -1.1 | -0.2 | -0.4 |
| Yes | 58.4 | -0.7 | -1.7 | 0.0 | -0.4 | -1.4 | 0.4 | 0.0 | 0.7 | -0.1 | 1.6 | 1.1 | 0.2 | 0.4 |
| Sport |  |  |  |  |  |  |  |  |  |  |  |  |  |  |
| No sporting activities | 22.8 | -3.2 | -3.7 | 0.0 | -5.9 | -6.3 | -3.0 | -0.1 | -7.3 | -7.5 | -4.6 | -1.8 | -0.5 | -0.3 |
| Less than 1 hour per week | 20.1 | -0.8 | -0.2 | 0.0 | -0.8 | -0.4 | 0.1 | 0.0 | -1.4 | -1.1 | -0.6 | -0.7 | -0.1 | 0.1 |
| 1 to less than 2 hours per week | 28.7 | 1.3 | 1.1 | 0.0 | 1.8 | 1.7 | 0.7 | 0.1 | 2.6 | 2.5 | 1.8 | 1.3 | 0.6 | 0.6 |
| 2 to less than 4 hours per week | 19.0 | 1.8 | 2.0 | 0.0 | 3.1 | 3.3 | 1.5 | 0.0 | 3.8 | 3.7 | 2.2 | 0.8 | -0.2 | -0.4 |
| 4 hours per week and more | 9.3 | 1.2 | 1.1 | 0.0 | 2.0 | 2.0 | 0.9 | 0.0 | 2.4 | 2.4 | 1.4 | 0.4 | 0.1 | -0.1 |
| KWsubj\_k |  |  |  |  |  |  |  |  |  |  |  |  |  |  |
| Scale value 1 to 3 | 29.9 | -0.3 | -0.5 | 0.0 | -0.7 | -0.9 | -0.4 | 0.2 | -0.4 | -1.0 | -0.2 | 0.4 | 0.3 | 0.3 |
| Scale value 4 to 7 | 53.3 | -0.1 | 0.0 | 0.0 | -0.1 | 0.1 | 0.2 | -0.1 | -0.1 | 0.2 | 0.2 | 0.1 | 0.2 | 0.4 |
| Scale value 8 to 10 | 16.8 | 0.5 | 0.6 | 0.0 | 1.0 | 1.0 | 0.2 | -0.2 | 0.6 | 1.0 | 0.0 | -0.6 | -0.6 | -0.9 |
| IAtermin |  |  |  |  |  |  |  |  |  |  |  |  |  |  |
| No | 53.9 | 0.1 | -0.4 | 0.0 | -0.6 | -1.1 | -0.9 | -0.1 | 0.9 | 0.1 | 0.3 | 1.1 | -0.1 | -0.1 |
| Yes | 36.1 | -0.4 | -0.1 | 0.0 | 0.2 | 0.4 | 0.7 | 0.2 | -1.3 | -0.8 | -0.5 | -1.1 | 0.1 | 0.1 |
| No need for examination or treatment | 10.0 | 0.4 | 0.8 | 0.0 | 0.7 | 1.1 | 0.3 | -0.1 | 0.5 | 1.1 | 0.3 | -0.1 | 0.1 | 0.0 |
|  |  |  |  |  |  |  |  |  |  |  |  |  |  |  |
| --- | --- | --- | --- | --- | --- | --- | --- | --- | --- | --- | --- | --- | --- | --- |
| aCS = Cross-Sectional | | | | | | | | | | | | | | |

## Male

Table 4.3: Standardized differences in percentage points compared to reference values for the recruitment survey, registration sample, and the sample of participants in Questionnaire D; calculated unweighted and weighted according to the respective weighting factors.

|  |  | Recruitment Survey | | | Registration | | | | Participation Questionnaire D | | | | | |
| --- | --- | --- | --- | --- | --- | --- | --- | --- | --- | --- | --- | --- | --- | --- |
| Parameter | Reference [%] | Unweighted | Design weight | CSa weight | Unweighted | Design weight | CSa weight | Drop-out Weight | Unweighted | Design weight | CSa weight (Recruitment Study) | Drop-out weight (Registration) | Drop-out weight | CSa weight |
| Agegrp |  |  |  |  |  |  |  |  |  |  |  |  |  |  |
| 18-29 yrs | 17.1 | -1.1 | -1.9 | -0.1 | -0.7 | -1.5 | 0.0 | -0.7 | -5.5 | -5.7 | -4.2 | -5.1 | -1.2 | -0.1 |
| 30-39 yrs | 16.6 | -2.0 | -1.2 | -0.1 | -1.0 | -0.1 | 1.0 | 0.1 | -3.2 | -2.7 | -1.8 | -2.8 | -0.4 | 0.4 |
| 40-49 yrs | 15.0 | -4.8 | -1.4 | -0.1 | -4.3 | -0.9 | 0.5 | 0.1 | -5.5 | -2.2 | -0.6 | -1.1 | 0.0 | -0.3 |
| 50-59 yrs | 17.9 | -0.7 | -0.3 | 0.0 | -0.4 | -0.3 | 0.0 | 0.0 | 0.4 | 0.7 | 1.3 | 1.1 | 0.1 | 0.4 |
| 60-69 yrs | 16.4 | 0.5 | 0.4 | -0.5 | 0.6 | 0.6 | -0.4 | -0.2 | 3.3 | 3.4 | 2.8 | 2.9 | 0.2 | -0.1 |
| 70-79 yrs | 10.0 | 5.7 | 3.9 | -0.5 | 4.8 | 3.0 | -1.3 | -0.7 | 7.8 | 6.0 | 1.5 | 2.1 | -0.5 | -0.2 |
| 80+ yrs | 7.0 | 3.7 | 1.5 | 1.7 | 1.9 | -0.5 | -0.3 | 1.8 | 3.7 | 1.0 | 1.4 | 3.7 | 2.3 | -0.2 |
| Federal state |  |  |  |  |  |  |  |  |  |  |  |  |  |  |
| Schleswig-Holstein | 3.5 | 12.2 | -0.1 | 0.0 | 12.3 | -0.1 | 0.1 | 0.2 | 12.3 | -0.2 | 0.1 | 0.1 | 0.4 | 0.0 |
| Hamburg | 2.2 | 3.3 | -0.5 | 0.0 | 3.7 | -0.2 | 0.4 | 0.3 | 3.8 | -0.2 | 0.6 | 0.4 | 0.7 | 0.0 |
| Lower Saxony | 9.6 | -3.6 | 0.1 | 0.1 | -3.9 | -0.2 | -0.1 | -0.1 | -3.9 | -0.3 | -0.3 | -0.3 | -0.3 | 0.0 |
| Bremen | 0.8 | 8.3 | -0.1 | 0.0 | 8.3 | -0.1 | -0.1 | 0.0 | 8.2 | -0.2 | -0.1 | -0.1 | 0.2 | 0.0 |
| North Rhine-Westphalia | 21.2 | -7.3 | -0.2 | 0.0 | -7.3 | 0.0 | 0.2 | -0.2 | -7.3 | -0.1 | 0.2 | -0.2 | -0.5 | -0.1 |
| Hesse | 7.6 | -4.1 | 0.4 | 0.0 | -3.7 | 0.7 | 0.1 | -0.1 | -4.1 | 0.2 | -0.6 | -0.8 | -0.8 | 0.0 |
| Rhineland-Palatinate | 5.0 | -3.3 | 0.3 | 0.0 | -2.9 | 0.7 | 0.5 | 0.4 | -2.3 | 1.7 | 1.5 | 1.4 | 1.4 | 0.0 |
| Baden-Württemberg | 13.4 | -5.9 | -0.3 | 0.0 | -5.5 | 0.0 | 0.2 | 0.1 | -5.9 | -0.4 | -0.5 | -0.7 | -1.0 | 0.0 |
| Bavaria | 16.0 | -5.8 | -0.2 | 0.0 | -5.9 | -0.4 | -0.3 | -0.2 | -5.4 | 0.4 | 0.5 | 0.6 | 0.4 | 0.0 |
| Saarland | 1.2 | 7.2 | 0.1 | 0.0 | 7.3 | 0.1 | 0.0 | 0.1 | 7.7 | 0.5 | 0.2 | 0.2 | 0.0 | 0.0 |
| Berlin | 4.4 | 10.7 | 0.1 | 0.0 | 11.0 | 0.2 | 0.3 | 0.2 | 10.6 | 0.0 | 0.2 | 0.1 | 1.0 | 0.0 |
| Brandenburg | 3.1 | 0.7 | 0.0 | -0.1 | 0.5 | -0.2 | -0.1 | 0.0 | 0.4 | -0.4 | -0.2 | -0.1 | 0.1 | 0.0 |
| Mecklenburg-Western Pomerania | 2.0 | 3.7 | -0.2 | 0.0 | 3.1 | -0.6 | -0.4 | -0.3 | 2.9 | -0.7 | -0.8 | -0.8 | -0.6 | 0.0 |
| Saxony | 4.9 | -2.8 | 0.0 | 0.0 | -3.0 | -0.2 | -0.3 | 0.0 | -2.6 | -0.1 | 0.0 | 0.5 | 0.1 | 0.0 |
| Saxony-Anhalt | 2.6 | 2.2 | 0.3 | 0.0 | 1.6 | -0.1 | -0.3 | 0.1 | 1.6 | -0.2 | -0.1 | 0.3 | 0.4 | 0.0 |
| Thuringia | 2.6 | 2.4 | 0.3 | 0.0 | 1.7 | -0.3 | -0.6 | -0.2 | 1.5 | -0.7 | -1.1 | -0.5 | -0.6 | 0.0 |
| German |  |  |  |  |  |  |  |  |  |  |  |  |  |  |
| Yes | 84.1 | 15.7 | 15.3 | 13.9 | 17.9 | 17.5 | 16.7 | 14.3 | 20.4 | 20.0 | 19.4 | 17.6 | 15.1 | 15.2 |
| No | 15.9 | -15.7 | -15.3 | -13.9 | -17.9 | -17.5 | -16.7 | -14.3 | -20.4 | -20.0 | -19.4 | -17.6 | -15.1 | -15.2 |
| BIK |  |  |  |  |  |  |  |  |  |  |  |  |  |  |
| BIK 1 | 10.9 | -0.9 | -0.4 | 0.0 | -1.5 | -1.0 | -0.6 | -0.1 | -1.8 | -1.4 | -0.8 | -0.3 | -0.1 | 0.0 |
| BIK 2 | 34.7 | -1.1 | 1.2 | -0.1 | -1.6 | 0.7 | -0.7 | 0.0 | -1.1 | 1.4 | -0.3 | 0.5 | 0.2 | 0.3 |
| BIK 3 | 26.6 | -2.5 | -0.9 | -0.3 | -2.2 | -0.6 | 0.0 | -0.3 | -2.5 | -0.9 | -0.6 | -1.0 | -1.0 | -0.8 |
| BIK 4 | 27.7 | 4.1 | -0.1 | 0.4 | 4.8 | 0.5 | 1.2 | 0.5 | 4.7 | 0.4 | 1.4 | 0.7 | 0.8 | 0.5 |
| Education |  |  |  |  |  |  |  |  |  |  |  |  |  |  |
| Low | 34.9 | -11.5 | -11.2 | -1.3 | -14.7 | -14.6 | -5.0 | -1.4 | -15.5 | -15.3 | -6.6 | -2.9 | -2.7 | -2.0 |
| Medium | 43.0 | 0.9 | 1.0 | 1.3 | 1.3 | 1.5 | 2.7 | 1.3 | -0.7 | -0.3 | 1.8 | 0.5 | 1.8 | 1.5 |
| High | 22.1 | 10.8 | 10.5 | 0.0 | 13.3 | 13.0 | 2.5 | 0.1 | 16.0 | 15.5 | 5.1 | 2.7 | 0.9 | 0.5 |
| Household size |  |  |  |  |  |  |  |  |  |  |  |  |  |  |
| Single-person household | 24.8 | -5.4 | -6.3 | 0.1 | -5.8 | -6.7 | -0.2 | 0.2 | -6.4 | -7.2 | -0.9 | -0.4 | 0.1 | 0.2 |
| Multi-person household | 75.2 | 5.4 | 6.3 | -0.1 | 5.8 | 6.7 | 0.2 | -0.2 | 6.4 | 7.2 | 0.9 | 0.4 | -0.1 | -0.2 |
| PAbmi\_k2 |  |  |  |  |  |  |  |  |  |  |  |  |  |  |
| Normal weight (18.5 <= BMI < 25) | 34.4 | 1.4 | 0.8 | 0.0 | 1.8 | 1.3 | 0.2 | -0.1 | 2.2 | 1.9 | 0.8 | 0.4 | 0.1 | 0.3 |
| Underweight (BMI < 18.5) | 1.1 | 0.0 | -0.5 | 0.0 | -0.3 | -0.7 | -0.4 | -0.4 | -0.4 | -0.9 | -0.4 | -0.4 | 0.2 | 0.2 |
| Overweight (25 <= BMI < 30) | 41.9 | 0.2 | 0.6 | 0.0 | -0.1 | 0.4 | -0.2 | 0.1 | 0.2 | 0.6 | 0.2 | 0.4 | 0.3 | -0.1 |
| Obesity (BMI >= 30) | 22.6 | -1.9 | -1.5 | 0.0 | -2.0 | -1.7 | 0.0 | 0.1 | -2.7 | -2.8 | -1.0 | -0.8 | -0.6 | -0.3 |
| Self-rated health |  |  |  |  |  |  |  |  |  |  |  |  |  |  |
| Very good/good/fair | 93.5 | 0.3 | 0.7 | 0.0 | 1.6 | 2.0 | 1.3 | 0.2 | 1.7 | 2.2 | 1.5 | 0.4 | 0.3 | 0.6 |
| Bad/very bad | 6.5 | -0.3 | -0.7 | 0.0 | -1.6 | -2.0 | -1.3 | -0.2 | -1.7 | -2.2 | -1.5 | -0.4 | -0.3 | -0.6 |
| Self-rated mental health |  |  |  |  |  |  |  |  |  |  |  |  |  |  |
| Excellent/very good/good | 81.4 | 1.6 | 1.8 | 0.0 | 2.0 | 2.1 | 0.1 | -0.2 | 2.7 | 2.7 | 0.6 | 0.2 | -0.5 | -0.3 |
| fair/poor | 18.6 | -1.6 | -1.8 | 0.0 | -2.0 | -2.1 | -0.1 | 0.2 | -2.7 | -2.7 | -0.6 | -0.2 | 0.5 | 0.3 |
| Paying attention to health |  |  |  |  |  |  |  |  |  |  |  |  |  |  |
| Not at all/less strong/moderate | 56.1 | -1.4 | -1.3 | 0.0 | -1.9 | -1.9 | -0.4 | 0.1 | -3.2 | -3.4 | -1.7 | -1.1 | 0.1 | 0.3 |
| Strong/very strong | 43.9 | 1.4 | 1.3 | 0.0 | 1.9 | 1.9 | 0.4 | -0.1 | 3.2 | 3.4 | 1.7 | 1.1 | -0.1 | -0.3 |
| Satisfaction: Life in general |  |  |  |  |  |  |  |  |  |  |  |  |  |  |
| Scale value 1 to 3 | 8.6 | -1.8 | -1.6 | 0.0 | -2.2 | -2.0 | -0.3 | 0.0 | -3.2 | -3.1 | -1.3 | -0.9 | -0.2 | -0.3 |
| Scale value 4 to 7 | 44.2 | -1.4 | -1.6 | 0.0 | -2.0 | -2.3 | -0.7 | -0.1 | -3.7 | -3.9 | -2.4 | -1.7 | -0.7 | -0.8 |
| Scale value 8 to 10 | 47.2 | 2.3 | 2.5 | 0.0 | 3.2 | 3.3 | 0.8 | 0.1 | 5.4 | 5.5 | 3.1 | 2.2 | 0.8 | 0.9 |
| Red meat |  |  |  |  |  |  |  |  |  |  |  |  |  |  |
| Never | 5.8 | 0.5 | -0.2 | 0.0 | 0.8 | 0.1 | 0.3 | 0.1 | 0.9 | 0.2 | 0.3 | 0.0 | 0.1 | 0.2 |
| Daily or several times a day | 4.4 | -1.7 | -1.0 | 0.0 | -2.4 | -1.6 | -0.5 | -0.1 | -3.6 | -2.7 | -1.8 | -1.6 | -0.3 | -0.3 |
| 4 to 6 times per week | 17.3 | -1.5 | -0.3 | 0.0 | -1.6 | -0.4 | 0.1 | 0.0 | -2.4 | -1.1 | -0.5 | -0.5 | 0.2 | 0.3 |
| 1 to 3 times per week | 49.9 | 0.5 | 0.6 | 0.0 | 0.3 | 0.4 | -0.2 | 0.0 | 0.4 | 0.6 | -0.1 | 0.0 | -0.3 | -0.4 |
| Less than once per week | 22.6 | 1.3 | 0.2 | 0.0 | 1.7 | 0.6 | 0.2 | 0.0 | 2.8 | 1.4 | 1.4 | 1.1 | 0.2 | 0.2 |
| Sausage products |  |  |  |  |  |  |  |  |  |  |  |  |  |  |
| Never | 7.2 | -0.1 | -0.3 | 0.0 | 0.2 | -0.1 | 0.1 | -0.1 | -0.7 | -0.8 | -0.9 | -1.4 | -0.8 | -0.9 |
| Daily or several times a day | 13.8 | -0.3 | -0.7 | 0.0 | -0.9 | -1.1 | -0.4 | 0.0 | -0.8 | -1.2 | -0.7 | -0.2 | 0.2 | 0.2 |
| 4 to 6 times per week | 25.1 | -0.3 | 0.1 | 0.0 | 0.1 | 0.4 | 0.3 | 0.0 | 0.6 | 0.9 | 0.7 | 0.6 | 0.3 | 0.2 |
| 1 to 3 times per week | 35.4 | 0.0 | 0.3 | 0.0 | -0.1 | 0.1 | 0.0 | 0.0 | 0.2 | 0.7 | 0.6 | 0.6 | 0.1 | 0.2 |
| Less than once per week | 18.4 | 0.7 | 0.3 | 0.0 | 0.8 | 0.4 | 0.0 | 0.0 | 0.2 | -0.2 | -0.3 | -0.3 | -0.2 | -0.1 |
| Smoking |  |  |  |  |  |  |  |  |  |  |  |  |  |  |
| Non-smoker | 72.8 | 4.4 | 3.9 | 0.0 | 5.4 | 5.0 | 1.1 | 0.4 | 9.2 | 8.9 | 5.5 | 4.8 | 1.9 | 1.9 |
| Daily smoking | 20.4 | -4.7 | -4.1 | 0.0 | -5.7 | -5.2 | -1.1 | -0.4 | -9.1 | -8.8 | -5.4 | -4.8 | -2.2 | -2.3 |
| Occasional smoking | 6.8 | -0.3 | -0.4 | 0.0 | -0.5 | -0.6 | -0.3 | -0.1 | -2.0 | -1.8 | -1.2 | -0.9 | 0.2 | 0.3 |
| Chronic diseases |  |  |  |  |  |  |  |  |  |  |  |  |  |  |
| No | 48.4 | -0.6 | -0.1 | 0.0 | -0.8 | -0.4 | -0.5 | -0.2 | -2.7 | -2.1 | -2.1 | -1.9 | -0.5 | 0.0 |
| Yes | 51.6 | 0.6 | 0.1 | 0.0 | 0.8 | 0.4 | 0.5 | 0.2 | 2.7 | 2.1 | 2.1 | 1.9 | 0.5 | 0.0 |
| Sport |  |  |  |  |  |  |  |  |  |  |  |  |  |  |
| No sporting activities | 23.3 | -1.8 | -2.4 | 0.0 | -3.3 | -4.0 | -1.7 | 0.0 | -3.4 | -4.3 | -2.2 | -0.5 | 0.1 | 0.1 |
| Less than 1 hour per week | 19.3 | -1.0 | -0.8 | 0.0 | -1.3 | -1.1 | -0.2 | 0.0 | -2.0 | -1.8 | -1.0 | -0.7 | -0.2 | -0.3 |
| 1 to less than 2 hours per week | 24.1 | 0.5 | 0.6 | 0.0 | 0.7 | 0.8 | 0.3 | 0.1 | 1.1 | 1.3 | 0.7 | 0.5 | -0.1 | 0.0 |
| 2 to less than 4 hours per week | 18.8 | 1.6 | 1.7 | 0.0 | 2.4 | 2.5 | 0.8 | 0.0 | 3.2 | 3.2 | 1.6 | 0.8 | 0.0 | 0.0 |
| 4 hours per week and more | 14.6 | 0.8 | 1.1 | 0.0 | 1.7 | 1.9 | 1.0 | 0.0 | 1.2 | 1.8 | 1.0 | -0.1 | 0.2 | 0.3 |
| KWsubj\_k |  |  |  |  |  |  |  |  |  |  |  |  |  |  |
| Scale value 1 to 3 | 40.0 | 0.1 | 0.0 | 0.0 | -0.3 | -0.4 | -0.4 | -0.2 | 0.1 | -0.2 | -0.7 | -0.4 | -0.3 | -0.5 |
| Scale value 4 to 7 | 44.6 | -0.1 | -0.1 | 0.0 | 0.0 | 0.0 | 0.2 | 0.1 | -0.3 | -0.1 | 0.4 | 0.4 | 0.4 | 0.5 |
| Scale value 8 to 10 | 15.4 | -0.1 | 0.1 | 0.0 | 0.5 | 0.6 | 0.3 | 0.1 | 0.3 | 0.4 | 0.5 | 0.0 | -0.1 | 0.0 |
| IAtermin |  |  |  |  |  |  |  |  |  |  |  |  |  |  |
| No | 55.6 | 2.0 | 1.1 | 0.0 | 2.0 | 1.2 | -0.1 | 0.1 | 4.0 | 3.3 | 1.8 | 2.0 | 0.2 | -0.1 |
| Yes | 30.9 | -1.4 | -0.9 | 0.0 | -1.6 | -1.1 | -0.1 | -0.1 | -2.7 | -2.3 | -1.3 | -1.3 | -0.1 | 0.0 |
| No need for examination or treatment | 13.5 | -1.0 | -0.4 | 0.0 | -0.7 | -0.2 | 0.2 | 0.0 | -2.2 | -1.6 | -0.9 | -1.1 | -0.1 | 0.1 |
|  |  |  |  |  |  |  |  |  |  |  |  |  |  |  |
| --- | --- | --- | --- | --- | --- | --- | --- | --- | --- | --- | --- | --- | --- | --- |
| aCS = Cross-Sectional | | | | | | | | | | | | | | |
